# Identifying a causal link between prolactin signaling pathways and COVID-19 vaccine-induced menstrual changes

**DOI:** 10.1038/s41541-023-00719-6

**Published:** 2023-09-01

**Authors:** Rima Hajjo, Ensaf Momani, Dima A. Sabbah, Nancy Baker, Alexander Tropsha

**Affiliations:** 1 Department of Pharmacy, Faculty of Pharmacy, Al-Zaytoonah University of Jordan, P.O. Box 130, Amman, 11733 Jordan; 2 Laboratory for Molecular Modeling, Division of Chemical Biology and Medicinal Chemistry, Eshelman School of Pharmacy, The University of North Carolina at Chapel Hill, Chapel Hill, NC USA; 3 Jordan CDC, Amman, Jordan; 4 Department of Basic Medical sciences, Faculty of Medicine, Al Balqa’ Applied University, Al-Salt, Jordan; 5 Applied Science Research Center, Applied Science Private University, Amman, Jordan; 6 ParlezChem, 123 W Union St., Hillsborough, NC 27278 USA

**Keywords:** Reproductive signs and symptoms, RNA vaccines

## Abstract

COVID-19 vaccines have been instrumental tools in the fight against SARS-CoV-2 helping to reduce disease severity and mortality. At the same time, just like any other therapeutic, COVID-19 vaccines were associated with adverse events. Women have reported menstrual cycle irregularity after receiving COVID-19 vaccines, and this led to renewed fears concerning COVID-19 vaccines and their effects on fertility. Herein we devised an informatics workflow to explore the causal drivers of menstrual cycle irregularity in response to vaccination with mRNA COVID-19 vaccine BNT162b2. Our methods relied on gene expression analysis in response to vaccination, followed by network biology analysis to derive testable hypotheses regarding the causal links between BNT162b2 and menstrual cycle irregularity. Five high-confidence transcription factors were identified as causal drivers of BNT162b2-induced menstrual irregularity, namely: IRF1, STAT1, RelA (p65 NF-kB subunit), STAT2 and IRF3. Furthermore, some biomarkers of menstrual irregularity, including TNF, IL6R, IL6ST, LIF, BIRC3, FGF2, ARHGDIB, RPS3, RHOU, MIF, were identified as topological genes and predicted as causal drivers of menstrual irregularity. Our network-based mechanism reconstruction results indicated that BNT162b2 exerted biological effects similar to those resulting from prolactin signaling. However, these effects were short-lived and didn’t raise concerns about long-term infertility issues. This approach can be applied to interrogate the functional links between drugs/vaccines and other side effects.

## Introduction

Corona virus disease 19 (COVID-19) pandemic, caused by the severe acute respiratory syndrome corona virus 2 (SARS-CoV-2), is still sweeping the world, causing more fatalities and threatening with dangerous viral variants and more economic losses^1–4^. The virus has infected hundreds of millions and caused millions of deaths worldwide. COVID-19 vaccines have been instrumental tools in the fight against the virus, and they helped reduce disease severity and mortality^5–10^. At the same time, COVID-19 vaccines were associated with adverse events, just like any other therapeutic^11–24^.

The occurrence of post-vaccine menstrual cycle disturbances has gone unnoticed during clinical trials phase of COVID-19 vaccines. Then, thousands of reports pointing to menstrual changes started to surface following worldwide vaccination campaigns^14,16,25^. Healthcare authorities dismissed these claims at the beginning and considered them unjustified. But such reports continued to appear from various countries around the world which led to increased vaccine hesitancy in young women^26–29^.

Recently, serious concerns have been raised about the effects of COVID-19 vaccines on menstruation^27–31^, and these fears keep escalating. Thousands of women reported post-COVID-19-vaccine menstrual changes to health care authorities around the world, and several published studies indicated an association between menstrual abnormalities and COVID-19 vaccines^32–34^. Women feared that menstrual changes suggest long-term adverse effects on fertility and pregnancy, which led to hesitation against vaccination among women. In fact, menstrual changes have been reported after receiving both mRNA and adenovirus vectored COVID-19 vaccines which led to the speculation that it is the nature of the immune response to vaccines, rather than vaccine components, that led to these adverse events^13,14,16,31,35–37^. Furthermore, previous reports suggested that vaccine-associated period changes occur due to transient perturbations to the hypothalamic-pituitary-ovarian (HPO) axis^38–40^. In fact, changes in menstrual cycles have been reported for non-COVID-19 vaccines including the human papillomavirus and typhoid vaccines^41,42^.

Herein, we devised a workflow to assess menstrual adverse events in response to treatment with mRNA vaccine BNT162b2. Our results revealed a causal link implicating prolactin signaling and hormone-induced effects on the menstrual cycle and endometrium resulting from post-vaccine gene expression perturbances. Fortunately, gene expression perturbations were short-term and therefore are not expected to cause long-term menstrual irregularities. The approach devised and implemented herein can be applied to assess other vaccines and other vaccine-induced biological effects.

## Results

### Systems biology findings

**Fig. 1:**
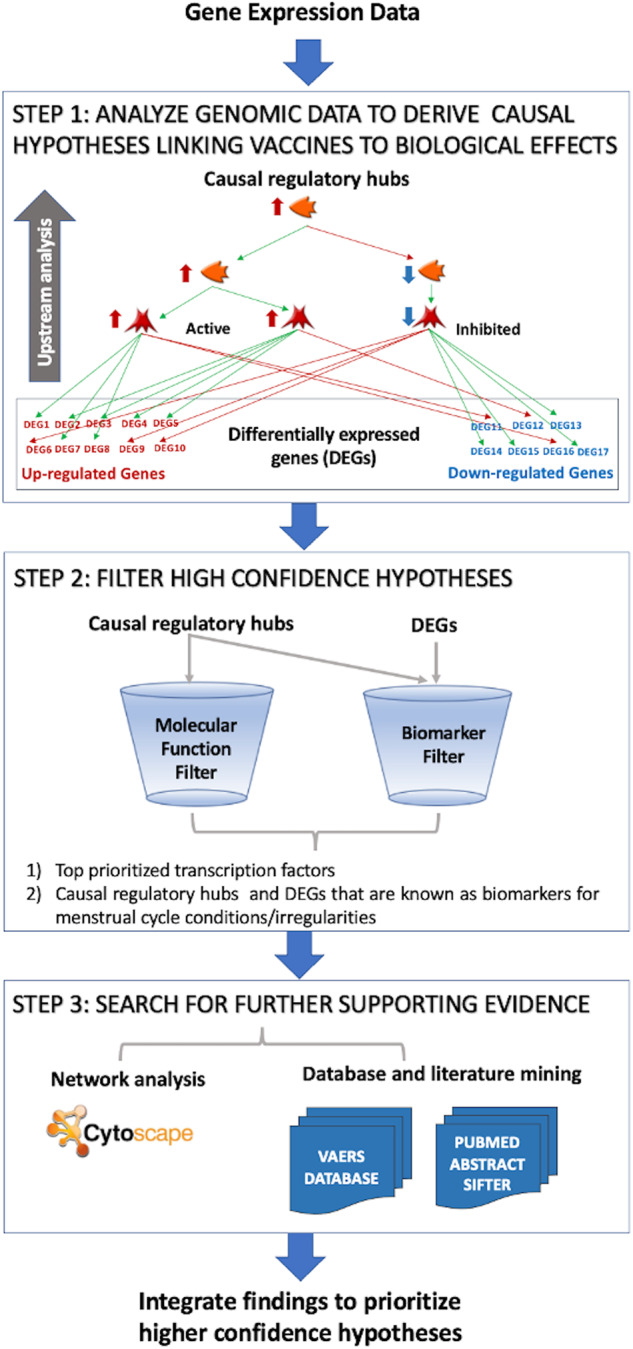
Informatics systems biology workflow. A devised workflow for studying the mechanism(s) underlying the biological effects of vaccines.

We undertook a systems biology approach to derive transcriptional signatures for COVID-19 mRNA vaccines relying on BNT162b2 transcriptomics data. Our workflow is shown in Fig. 1. Previously, we applied similar approaches to explore the network pharmacology of drugs and vaccines^43^, as well as investigating disease pathogenesis pathways for prioritizing biomarkers and drug targets^11,12^. Each study workflow is tweaked to suit the scientific questions we are asking as well as the types of data we have. In this study, the analysis of transcriptional raw data extracted from GSE169159 indicated that gene expression alterations on day 22 of receiving the first vaccine dose (i.e., the day after receiving the second vaccine dose) affected genes that are known biomarkers or drug targets for menstrual cycle disturbances. All details on deriving gene signatures from transcriptomics data of GSE169159 are described elsewhere^12^. Additionally, no significant DEGs were observed on day 28 after receiving the first vaccine dose.

### Vaccine Gene Signatures

Two transcriptomic gene signatures (GS) for BNT162b2 vaccine were derived from gene expression profiling experiments in response to treatment with vaccine (GSE169159)^44^. The first gene signature (GS1) consisted of 1853 differentially expressed genes (DEGs), 884 upregulated and 969 downregulated DEGs that satisfied the prioritization criteria of a false discovery rate (FDR) ≤ 0.05, and a log2 fold change (log_2_FC) ≥ 2.00 or ≤ −2.00. The second gene signature (GS2) consisted of 108 DEGs, 74 upregulated and 18 downregulated DEGs that satisfied the prioritization criteria of an FDR ≤ 0.05, and a log_2_FC ≥ 5.00 or ≤ −5.00.

In order to get a better idea about the biological significance of the DEGs in response to treatment with BNT162b2, we applied a bioinformatics workflow relying on both downstream enrichment analysis, and upstream analysis for putative regulators responsible for causing the gene expression changes observed in transcriptomics data.

### Upstream regulation analysis

Upstream analysis was performed using the DEGs in GS1 (applying a threshold of log_2_FC) and GS2 using causal reasoning^45^. GS1 consisted of 1853 DEGs and therefore was trimmed by our upstream analysis algorithm to reduce the complexity of generated results. The algorithm automatically applied log_2_FC ≥ +2.62 or ≤ −2.62 threshold which reduced the number of DEGs to 1107 (743 upregulated and 612 downregulated); since the applied causal reasoning algorithm requires a query list of about thousand genes on average. This analysis resulted in the prediction of 826 activated and 480 inactivated upstream regulators including transcription factors, kinases, phosphatases, and microRNAs (Supplementary Table 1). All prioritized upstream regulators have prediction activities with *p*-values ≤ 0.05 and a calculation distance = 1–3.

To focus on upstream regulators of genes with the maximal differential expression in response to vaccination, we applied the same analysis to GS2 consisting of 92 DEGs (74 upregulated and 18 downregulated) that had log_2_FC ≥ +5.00 or ≤ −5.00. Our analysis resulted in predicting 625 activated proteins 389 inactivated (Supplementary Table 2).

All prioritized upstream regulators were scored based on the number of differentially expressed genes that can be reached via the shortest paths, and the correctness of the regulation. The activity prediction correctness is assessed based on the activation and inhibition edges along the paths and the expected and observed directionality of fold changes of the DEGs. It should be noted that the calculation distance is one of the most important parameters that can distinguish direct regulation effects from indirect effects. For example, a calculation distance of one means that the upstream regulator is one step away from the transcriptional event indicating that the regulation event in direct.

### Filtering upstream regulators by molecular function and distance

The changes in gene expression that we observe in response to perturbagens (e.g., vaccines), often result from interactions between gene regulatory regions and regulatory proteins such as transcription factors, kinases, phosphatase, RNA molecules and others. Transcription factors (TFs) are considered topologically more important than DEGs especially for purposes of mechanism reconstruction; or at least complementary to DEGs for reconstructing the biological pathways and networks responsible for a phenotype of interest (e.g., menstrual irregularities in response to vaccination). Although transcription factors are not the only master regulators, but they are probably one of the easiest to validate experimentally using in vitro assays.

Hence, we sought filtering the predicted causal regulatory proteins by “molecular function” = “transcription factor” and “calculation distance” = 1. A calculation distance=1, indicates direct effects on transcription. This filtering step resulted in 13 transcription factors (IRF1, STAT1, STAT2, RELA, IRF9, SPI, NFKB1, IRF3, IRF7, BCL6, PRDM1, GATA3) for DEGs in GS1 with log_2_FC ≥ +2.62 or ≤ −2.62 (Supplementary Table 3). Applying the same filters on the causal regulatory proteins predicted for GS2 resulted in the prediction of five transcription factors (IRF1, STAT1, RELA, IRF3 and STAT 2), with a calculation distance = 1 (Supplementary Table 4). The overlapping TFs resulting for the previous two causal reasoning analyses are listed in Table 1. Such hits can be considered higher confidence transcription factors for causing the observed phenotype.

**Table 1:** Top commonly predicted upstream regulatory hubs at a distance of 1 for DEGs in GS1 and GS2.

Molecular entity	Molecular function	Gene	Predicted activity^a^	Correct/total^b^	Prediction *p*-value^c^	Distance^d^	Evidence^e^
IRF1	Transcription factor	IRF1	+	60/62* 17/17**	4.24E-16^f^ 7.63E-06^g^	1	Yes^59,60^
STAT1	Transcription factor	STAT1	+	64/70* 15/16**	1.22E-13^f^ 2.59E-04^g^	1	Yes^118–122^
RelA (p65 NF-kB subunit)	Transcription factor	RELA	+	59/78* 14/15**	3.21E-06^f^ 4.88E-04^g^	1	Yes^123–132^
STAT2	Transcription factor	STAT2	+	23/24* 8/8**	1.49E-06^f^ 3.91E-03^g^	1	Yes^117,133–137^
IRF3	Transcription factor	IRF3	+	19/22* 9/9**	4.28E-04^f^ 1.95E-03^g^	1	Yes^138–148^

^a^Predicted activity of the key hub by causal reasoning is denoted by – if the hub is inhibited, and denoted by + if the hub is activated.

^b^Correct/total network predictions show the number of genes in the dataset predicted correctly over the total number of genes in the causal reasoning network.

^c^Calculation distance from the upstream regulatory key hubs and downstream genes.

For example, distances of 2 and 3 identify key hubs that are distant key hubs, while a distance of 1 identify closest one-step away transcriptional factors.

^d^*p*-value of the predicted protein activity calculated using the polynomial test.

^e^Evidence for an existing experimental link between the transcription factor and menstrual irregularity.

^f^*p*-value of the predicted protein activity calculated using the polynomial test for GS1.

^g^*p*-value of the predicted protein activity calculated using the polynomial test for GS2.

*For GS1.

**For GS2.

Two of the five higher confidence transcription factors (IRF1 and IRF3) belong to the interferon regulatory factors, and other two transcription factors (STAT1 and STAT2) belong to the signal transducers and transcription activators which mediate cellular responses to interferons. The fifth transcription factor, RelA, belongs to the Rel homology domain/immunoglobulin-like fold and is a regulator of NF-kB activity. As a validation of these findings, we reported examples of supporting evidence in the biomedical literature linking these five predicted higher-confidence transcription factors to menstrual cycle irregularities (Table 1). In fact, supporting studies cited in Table 1 brought our attention to significant interactions between these transcription factors and prolactin/PRL gene.

**Fig. 2:**
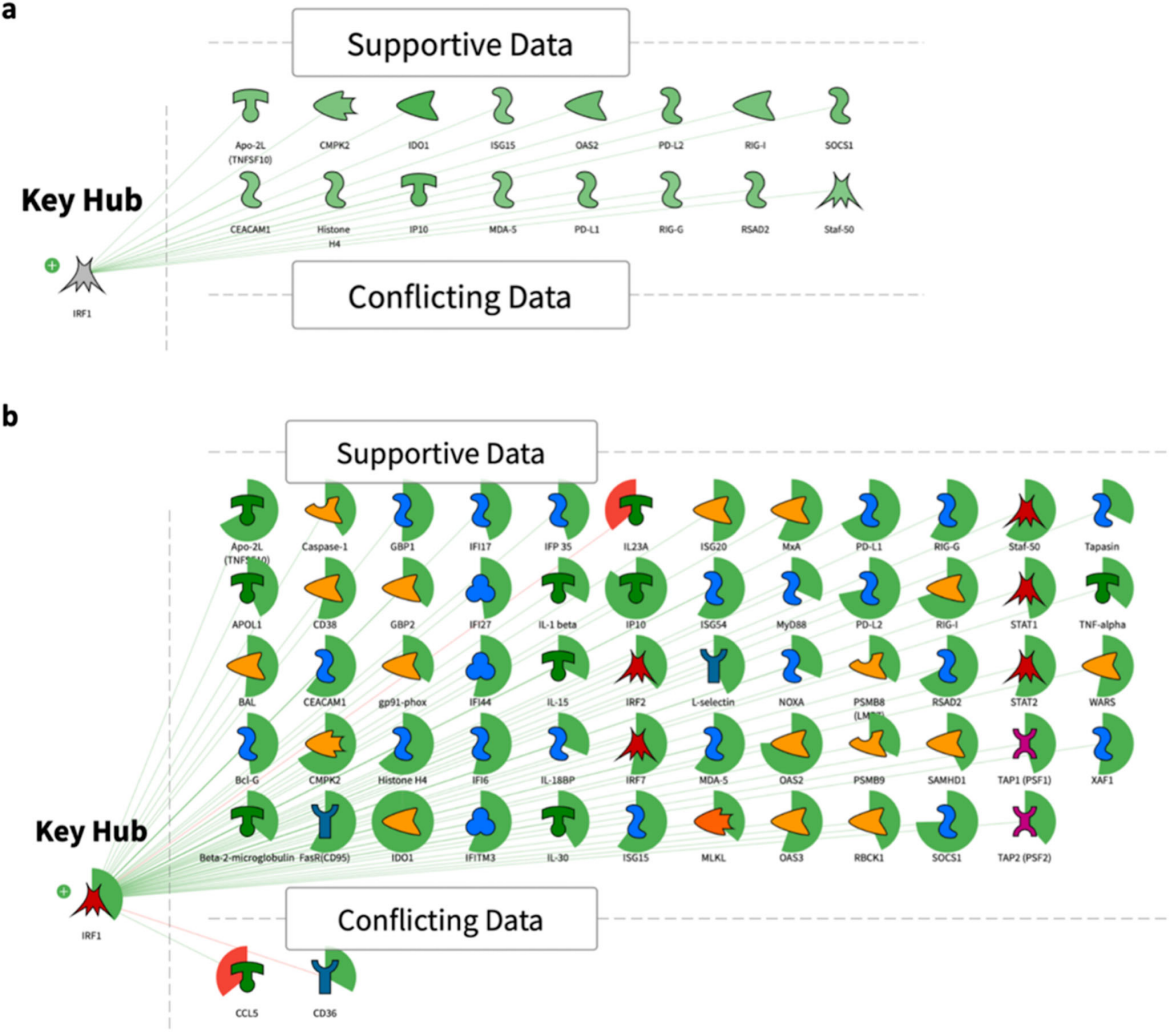
Causal reasoning networks. **a** Causal reasoning network of highest confidence transcription factor IRF1 using DEGs in GS1. **b** Causal reasoning network of highest confidence transcription factor IRF1 using DEGs in GS2. Gene expression changes are shown in green and red sectors around each molecule. Increased expression value corresponds to the green sector which size increases clockwise around the molecule icon. Decreased expression value corresponds to the red sector which size increases counterclockwise. Supportive data panel contains over and under-expressed genes from the experimental data set which support a hypothesis that IRF1 is in a predicted predominant “active” state. Conflicting data panel contains over and under-expressed genes from the experimental data set which are discordant with the hypothesis that IRF1 is in predicted predominant “active” state.

Among all predicted transcription factors for GS1 and GS2, IRF1 had the smallest activity prediction *p*-values values (4.237E-16 for GS1, and 7.63E-06 for GS2). Therefore, IRF’s causal reasoning network shown in Fig. 2a, b. This network serves as an example of the causal reasoning networks we relied in this work. Additional networks for STAT1, RELA, IRF3 and STAT 2 are provided in Supplementary Material (Supplementary Fig. 1–4).

### Identifying important RNA molecules as upstream regulators

Many RNA molecules, including microRNAs and long non-coding RNAs, haven been predicted as upstream molecular regulators that worth further analysis by experimental scientists. DEGs in GS1, with log_2_FC ≥ +2.62 or ≤ −2.62 and FDR ≤ 0.05, led to the prioritization of 182 RNA molecules including miR-502-5p, miR-345-5p, miR-548x-3p, miR-548x-3p, miR-935, miR-383-5p, miR-450a-5p, miR-450a-5p, miR-4674 and miR-3941 which topped the list based on their activity prediction *p*-values. However, DEGs in GS2 led to the prioritization of 28 RNA molecules including LINC02605, miR-221-5p, NBAT1, RMRP, miR-378b, MIR31HG, HOXB-AS1, miR-514b-5p, LINC00277 and CASC9 as top hits. Additionally, we identified 18 overlapping RNA molecules between the 182 (from the first DEGs in GS1) and 28 (from GS2) that are considered high confidence RNA upstream regulators. The overlapping RNA molecules were in order of their activity prediction *p*-values, from smaller to larger values, were: LINC02605, miR-221-5p, NBAT1, RMRP, miR-378b, MIR31HG, HOXB-AS1, miR-514b-5p, LINC00277, CASC9, ZFPM2-AS1, miR-219-1-3p, miR-3941, LINC02605, miR-210-5p, miR-3134, MIR31HG, LOC101929517, LINC-PINT, SBF2-AS1.

### Filtering upstream regulators and downstream DEGs by biomarker uses

Menstrual irregularity biomarkers were extracted from the Cortellis Drug Discovery Intelligence (CDDI) database^46^ using the following search terms: biomarker type = “gene” or “protein”; condition = “menstrual cycle”, “menstrual abnormalities” or “premenstrual syndrome”; biomarker role = “diagnosis”, “disease profiling”, “prognosis” or “prognosis–risk stratification”. Finally, we retrieved 213 biomarkers in total. We also extracted 177 biomarkers for prolactin.

**Fig. 3:**
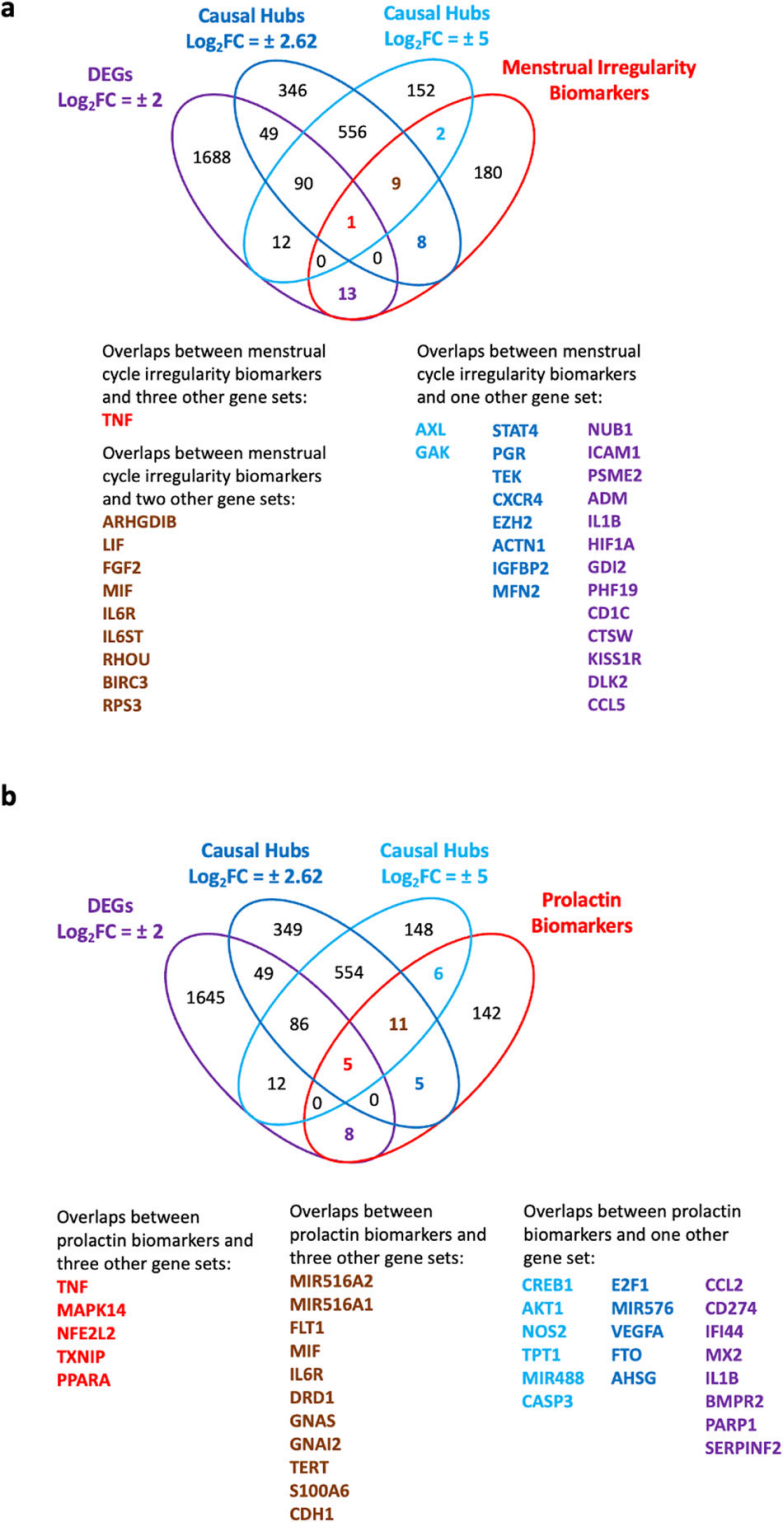
Overlapping DEGs, causal upstream hubs and biomarkers. **a** Venn diagram showing overlaps between DEGs, predicted causal upstream regulatory hubs using DEGs in GS1 and GS2, and menstrual irregularity biomarkers. **b** Venn diagram showing overlaps between DEGs, predicted causal upstream regulatory hubs using DEGs in GS1 and GS2, and prolactin signaling biomarkers.

Next, we checked overlaps between the 213-biomarker set and 3 gene sets of interest: 1) DEGs with log_2_FC ≥ +2.00 or ≤ −2.00, 2) causal hubs predicted using DEGs with log_2_FC ≥ +2.00 or ≤ −2.00, and 3) causal hubs predicted using DEGs with log_2_FC ≥ +5 or ≤ −5. Our results indicated that TNF was the only common gene between our biomarkers list and all other three gene lists. Nine genes (ARHGDIB, LIF, FGF2, MIF, IL6R, IL6ST, RHOU, BIRC3 and RPS3) were common between the biomarkers list and two gene sets. Finally, there were 33 common genes between menstrual cycle biomarkers and vaccine-induced DEGs and/or predicted causal proteins (Table 2), and 35 gene overlaps between prolactin biomarkers and vaccine-induced DEGs and/or predicted causal proteins (Table 3). All gene overlaps are shown in Fig. 3a, b. We could look at overlaps with more gene lists as a method of filtering higher confidence “topological” genes that may be driving the menstrual irregularities in response to COVID-19 vaccines (i.e., increased confidence due to biological relevance).

**Table 2:** List of menstrual irregularity biomarkers which overlap with BNT162b2 vaccine-induced DEGs and/or predicted causal proteins.

#	Gene	Confidence Score	Activity	Activity Prediction *p*-value GS1	Activity Prediction *p*-value GS2	DEG
1	TNF	4	+	3.80E-11	2.96E-03	Yes
2	IL6R	3	+	3.08E-12	1.37E-04	No
3	IL6ST	3	+	3.08E-12	9.00E-05	No
4	LIF	3	+	5.24E-12	2.59E-04	No
5	BIRC3	3	+	8.89E-12	7.63E-06	No
6	FGF2	3	+	3.00E-11	1.18E-03	No
7	ARHGDIB	3	-	7.26E-11	7.63E-06	No
8	RPS3	3	+	1.25E-04	6.27E-03	No
9	RHOU	3	+	4.76E-04	3.60E-03	No
10	MIF	3	+	9.94E-03	3.60E-03	No
11	STAT4	2	+	8.48E-08	NA	No
12	TEK	2	-	4.92E-06	NA	No
13	CXCR4	2	+	9.06E-05	NA	No
14	GAK	2	+	2.67E-04	NA	No
15	ACTN1	2	+	9.62E-04	NA	No
16	PGR	2	-	5.31E-03	NA	No
17	MFN2	2	-	7.00E-03	NA	No
18	EZH2	2	+	7.06E-03	NA	No
19	AXL	2	+	NA	3.69E-03	No
20	IGFBP2	2	+	NA	3.81E-03	No
21	NUB1	1	NA	NA	NA	Yes
22	ICAM1	1	NA	NA	NA	Yes
23	PSME2	1	NA	NA	NA	Yes
24	ADM	1	NA	NA	NA	Yes
25	IL1B	1	NA	NA	NA	Yes
26	HIF1A	1	NA	NA	NA	Yes
27	GDI2	1	NA	NA	NA	Yes
28	PHF19	1	NA	NA	NA	Yes
29	CD1C	1	NA	NA	NA	Yes
30	CTSW	1	NA	NA	NA	Yes
31	KISS1R	1	NA	NA	NA	Yes
32	DLK2	1	NA	NA	NA	Yes
33	CCL5	1	NA	NA	NA	Yes

All molecules were ranked based on their activity prediction *p*-values as well their overlap confidence score. An overlap confidence score of 3 indicates that a specific gene/protein is overlapping between the biomarker set and 3 other gene sets, while a score of 1 indicates that gene/protein is overlapping between the biomarker list and one other gene set.

**Table 3:** List of prolactin biomarkers which overlap with BNT162b2 vaccine-induced DEGs and/or predicted causal proteins.

#	Gene	Overlap confidence score	Activity	Activity prediction *p*-value GS1	Activity prediction *p*-value GS2	DEG
1	TNF	3	+	3.80E-11	2.96E-03	Yes
2	MAPK14	3	+	5.46E-07	4.39E-04	Yes
3	PPARA	3	-	9.69E-06	2.97E-05	Yes
4	NFE2L2	3	-	2.32E-03	4.68E-03	Yes
5	TXNIP	3	-	3.81E-03	1.29E-03	Yes
6	IL6R	2	+	3.08E-12	1.37E-04	No
7	GNAS	2	+	9.79E-12	1.79E-05	No
8	GNAI2	2	+	1.53E-10	6.06E-05	No
9	DRD1	2	+	3.56E-09	4.88E-04	No
10	TERT	2	+	9.22E-07	5.48E-06	No
11	S100A6	2	-	1.36E-05	7.83E-05	No
12	FLT1	2	+	2.01E-05	1.63E-04	No
13	CDH1	2	-	3.28E-05	3.64E-04	No
14	MIR516A2	2	+	9.57E-04	8.45E-03	No
15	MIR516A1	2	-	9.57E-04	8.45E-03	No
16	MIF	2	+	9.94E-03	3.60E-03	No
17	VEGFA	1	+	9.58E-10	NA	No
18	E2F1	1	+	1.48E-04	NA	No
19	MIR576	1	-	4.01E-04	NA	No
20	FTO	1	-	3.30E-03	NA	No
21	AHSG	1	+	4.74E-03	NA	No
22	MIR488	1	-	NA	9.72E-06	No
23	CREB1	1	+	NA	2.59E-04	No
24	AKT1	1	+	NA	2.17E-03	No
25	NOS2	1	+	NA	5.91E-03	No
26	TPT1	1	+	NA	6.27E-03	No
27	CASP3	1	-	NA	6.47E-03	No
28	CCL2	1	NA	NA	NA	Yes
29	CD274	1	NA	NA	NA	Yes
30	IFI44	1	NA	NA	NA	Yes
31	MX2	1	NA	NA	NA	Yes
32	IL1B	1	NA	NA	NA	Yes
33	BMPR2	1	NA	NA	NA	Yes
34	PARP1	1	NA	NA	NA	Yes
35	SERPINF2	1	NA	NA	NA	Yes

All molecules were ranked based on their activity prediction *p*-values as well their overlap confidence score. An overlap confidence score of 3 indicates that a specific gene/protein is overlapping between the biomarker set and 3 other gene sets, while a score of 1 indicates that gene/protein is overlapping between the biomarker list and one other gene set.

### Pathway enrichment and interconnectivity analysis

**Fig. 4:**
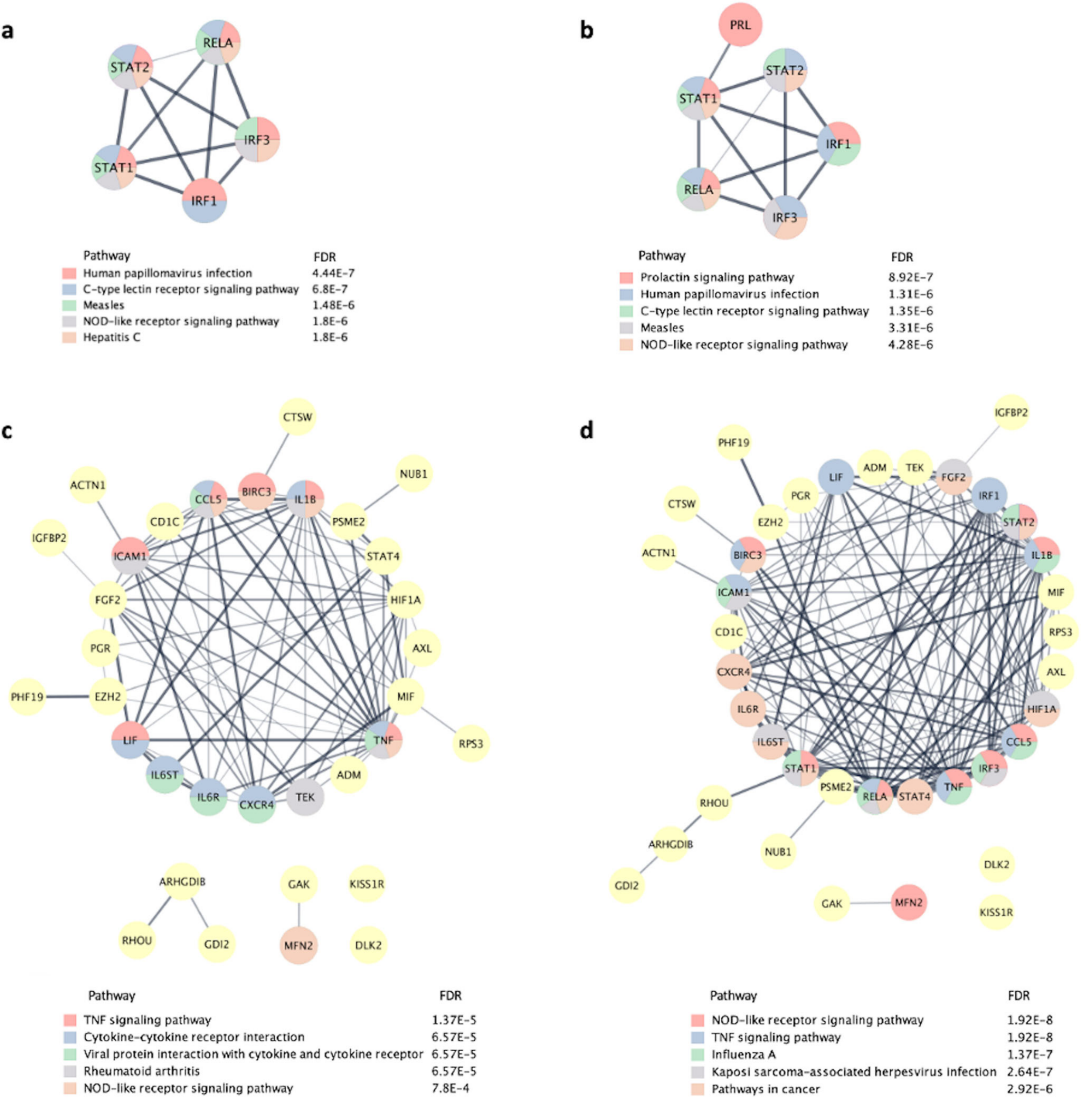
Interconnectivity between prioritized high confidence transcription factors. **a** Direct interactions network of five higher confidence causal transcription factors. **b** Direct interactions network of five higher confidence causal transcription factors in addition to prolactin (PRL). **c** Direct interactions network of 33 causal upstream regulators that are known biomarkers for menstrual disturbances. **d** Direct interactions network of 33 causal upstream regulators that are known biomarkers for menstrual disturbances, in addition to prioritized 5 topological genes and PRL. Thick edges correspond to confidence score $\ge$ 0.70 (i.e., high confidence score), while the thin edge corresponds to a confidence level $\le$ 0.50 (i.e., low confidence score). Nodes are color-coded using a split pie chart coloring scheme indicating pathway/gene set contribution to each node from the top 5 most enriched pathways/gene lists. FDR values represent he significance of the predicted pathway. Generated based on STRING data on 27 September 2022.

We used pathway enrichment analysis to assess whether the identified causal regulators work collectively to affect certain biological pathways. Pathway enrichment results using five higher confidence upstream causal regulators as a query gene list (IRF1, STAT1, RELA, IRF3 and STAT 2), highlighted the prolactin signaling pathway as one of the significantly enriched pathways with an enrichment *p*-value of 1.60E-05 (Fig. 4a). Next, we included PRL in the query list (PRL, IRF1, STAT1, RELA, IRF3 and STAT 2) for pathway enrichment which led to the prioritization of prolactin singling pathway as the top enriched pathway with an enrichment *p*-value of 8.92E-07 (Fig. 4b). Similar analyses were performed on the 33 filtered biomarker genes/proteins (Fig. 4c), and biomarker proteins in addition to PRL, IRF1, STAT1, RELA, IRF3 and STAT 2 (Fig. 4d).

**Fig. 5:**
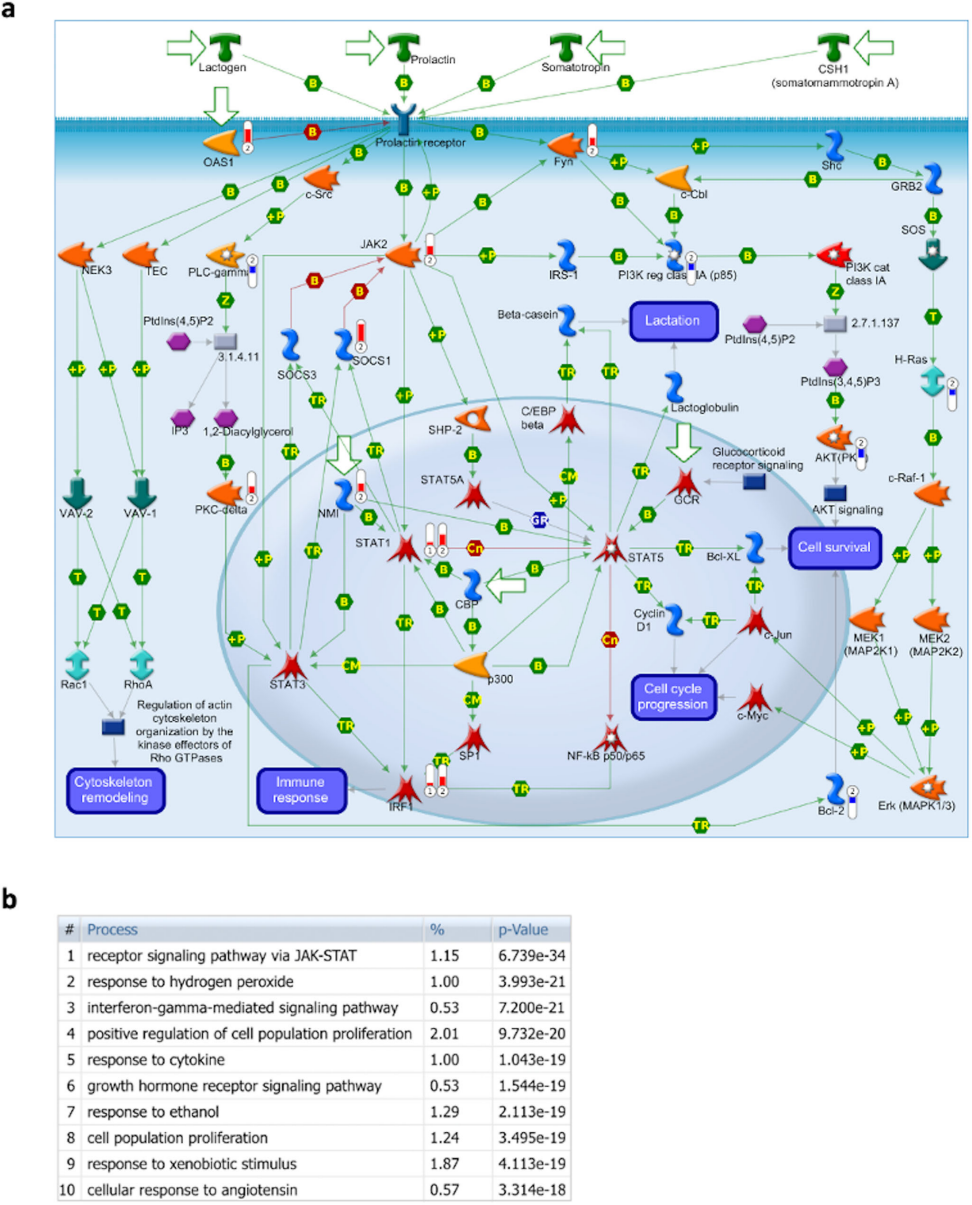
Prolactin signaling pathway. **a** Prolactin signaling pathway map. A node (or object) on the map could be a gene, protein or chemical compound. Query genes from experimental data which intersect with pathway protein or chemical compound. Query genes from experimental data which intersect with pathway objects are denoted by thermometers. Thermometer 1 represents causal transcription factors. Thermometer 2 represents DEGs in response to treatment with vaccine, applying thresholds of log_2_FC ≥ 2.00 or ≤ −2.00, and FDR ≤ 0.05. **b** Biological processes involved in prolactin signaling pathway. The % refers to the percentage of network objects in the pathway map. The *p*-value is the process prediction *p*-value.

It is well known that changes in prolactin signaling can result in menstrual cycle irregularities^47–51^. Searching PubMed using terms “prolactin” AND “menstrual” returned 1950 results, while search terms “prolactin” AND “menstrual cycle” returned 1270 results, and search terms “prolactin” AND “menstrual irregularity” returned 32 results. This is a clear indication that the studied mRNA COVID-19 vaccine BNT162b2 has the potential to cause menstrual irregularities by inducing perturbations in the genes and/or proteins involved in prolactin signaling pathways (i.e., through affecting the activity of key transcription factors involved in this pathway). Prolactin signaling pathway affects a wide range of physiological processes ranging from reproduction and lactation to growth and development, from endocrinology and metabolism to brain and behavior, as well as immune regulation (Fig. 5a, b).

Prolactin is a polypeptide hormone encoded by the PRL gene and secreted by the anterior pituitary gland. It is known as a growth regulator for many tissues, including cells of the immune system. It functions as a cytokine with immunomodulatory activities. It may also play a role in cell survival by suppressing apoptosis, and it is essential for lactation. Chemically, prolactin’s structure is similar to those of the growth hormone and the placental lactogen hormone, which form together what is known as the “prolactin/growth hormone/placental lactogen” family, and they all originated from one ancestral gene.

### Signaling pathway impact analysis (SPIA)

SPIA was performed on the combined list consisting of the DEGs and the predicted causal hubs. Enrichment analyses performed on the combined list of DEGs, and key hubs have previously been shown to highlight more biologically relevant results^52^. The top five enriched pathway maps were: 1) immune response interferon-alpha/beta (IFN-alpha/beta) signaling via JAK/STAT, 2) regulation of antiviral response by SARS-CoV-2, 3) immune response antiviral actions of interferons, 4) immune response induction of apoptosis and inhibition of proliferation mediated by interferon-gamma (IFN-gamma), and 5) immune response IFN-­gamma in macrophages activation with impact *p*-values of 4.31E-25, 1.24E-11, 1.04E-10, 5.61E-10, and 1.59E-9 respectively. All predicted pathways are highlighting the role of interferons. Hence, SPIA results revealed a prominent role of interferon signaling in the signaling pathways impacted by BNT162b2 vaccines.

### Supporting evidence

We mined the VAERS database, PubMed using Abstract Sifter, and the Connectivity Map to gather supporting evidence for the prioritized hypotheses regarding vaccine-induced menstrual irregularities and prolactin or HPO mimicking effects.

#### VAERS database

We searched the VAERS database for vaccine adverse events that are relevant to menstrual irregularities. First, we extracted all COVID-19 vaccine adverse events and ranked them according to their proportions of the overall reported adverse events for COVID-19 vaccines. VAERS annotates menstrual irregularity under ‘menstruation irregular’, in addition to using more specific “symptom” terms, including heavy menstrual bleeding, dysmenorrhea, intermenstrual bleeding, amenorrhea, postmenopausal hemorrhage, premenstrual pain, menstrual discomfort, menstruation normal, premenstrual dysphoric disorder, menstrual cycle management, premenstrual headache, retrograde menstruation, premenstrual syndrome and menstrual disorder.

Our analysis indicated that none of the menstruation disturbances listed above were among the most frequently reported adverse events for COVID-19 vaccines that we detailed before^12^. An updated adverse event report for COVID-19 vaccines is provided in Supplementary Table 5. However, some cross-sectional studies reported high frequencies of these side effects. There could be many explanations for this discrepancy between adverse event databases and cross-sectional studies. Women tend to neglect seeking medical attention for what they perceive as a mild non-threatening short-term menstruation irregularity^53–55^. In fact, women participating in cross-sectional studies are unlikely to report changes to periods unless specifically asked^16^.

In fact, mining VAERS data for menstruation irregularities resulted in 35,386 adverse events that were not restricted to COVID-19 vaccines (Supplementary Table 6). The top five vaccines that had the highest share in these events were COVID-19 vaccine (26,714 events comprising 85.82%), human papillomavirus recombinant vaccine (1198 events comprising 3.85%), hepatitis B vaccine (1013 comprising 3.25%), trivalent influenza virus vaccine (581 events comprising 1.87%) and the zoster vaccine (566 events comprising 1.82%). It is noteworthy that all these vaccines are given later rather than the first few years of life, permitting adverse event reporting by menstruating women.

#### PubMed Abstract Sifter

**Fig. 6:**
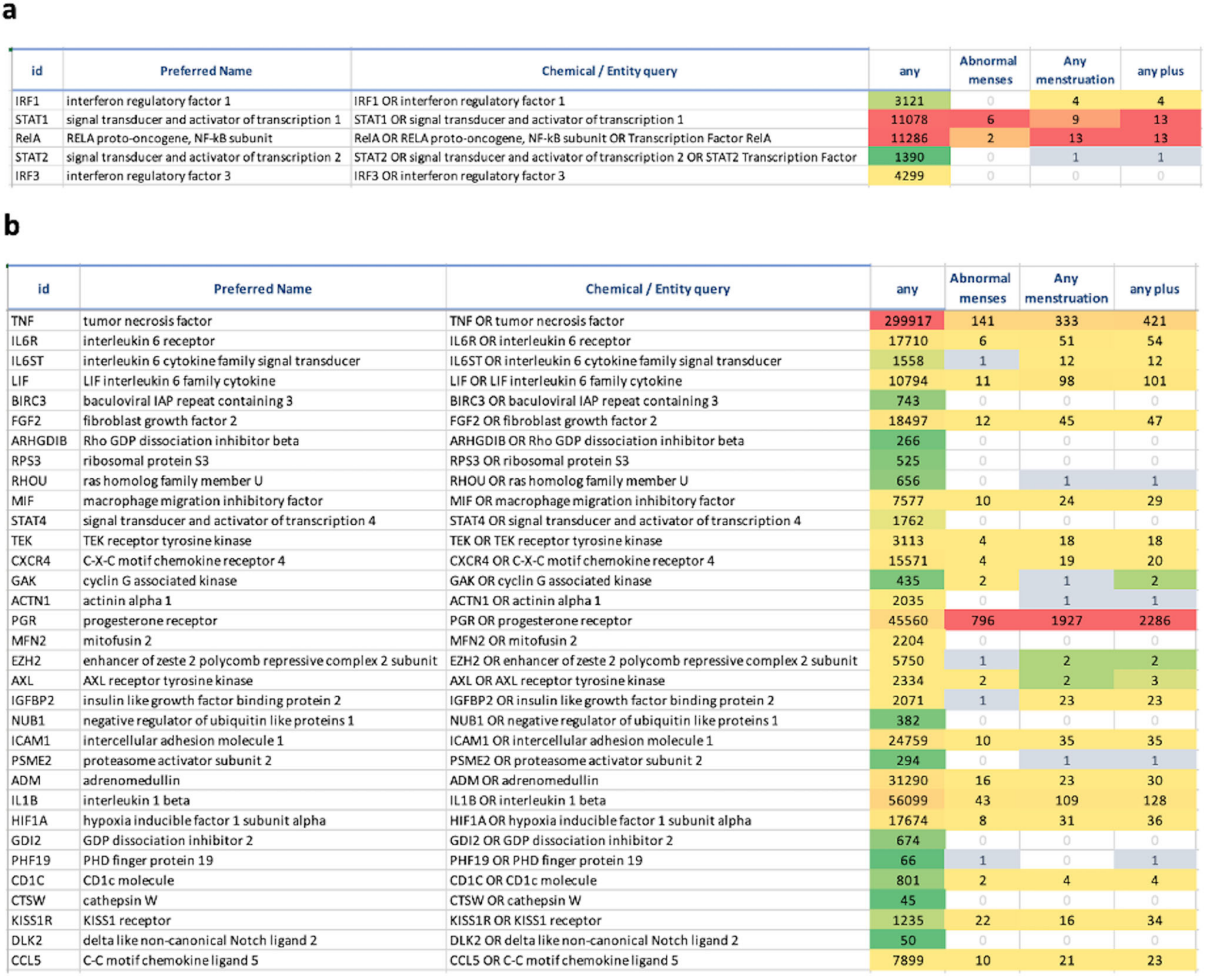
Screenshots from PubMed Abstract Sifter. **a** Landscape sheet of the PubMed Abstract Sifter showing relationships, in the form of article counts, between biological concepts highlighted in this study. The first column “id” lists the gene symbols of prioritized top five causal transcription factors. **b** Landscape sheet of the PubMed Abstract Sifter showing relationships, in the form of article counts, between causal biological concepts highlighted in this study. The first column “id” lists the gene symbols of prioritized causal genes and vaccine-induced DEGs that are known as also biomarkers for menstrual cycle according to the CDDI database^46^.

To increase the confidence in the prioritized causal hits, we examined the relationship(s) between the prioritized genes and menstruation irregularities in more depth and complexity using the PubMed Abstract Sifter^56^. On the Landscape sheet we built queries that revealed the number of articles satisfying a variety of queries related to menstrual cycle, abnormal menstruation, vaccines, and prioritized upstream causal regulators. These results (Supplementary Table 7) revealed a sizable publication record for the relationship between menstruation and genes affected by COVID-19 vaccines (DEGs and/or causal hubs). The results of two queries consisting of the higher confidence list of prioritized causal transcription factors, and biomarkers causal hubs and DEGs are shown in Fig. 6a, b.

#### The Connectivity Map (CMap)

The Connectivity Map analysis suggests that drugs capable of inducing transcriptomics effects opposite to those induced by mRNA vaccines could reverse vaccine side effects^57,58^. To identify small-molecule drugs that could prevent or reverse vaccine’s side effects, we ranked all DEGs in response to vaccination by BNT162b2 according to their expression levels using log_2_FC values, to query the Connectivity Map database^59^. The CMap query gene signature consisted of the 50 most upregulated genes and the 50 most downregulated genes in response to vaccination with BNT162b2. Compound hits that produced opposite transcriptional signatures to the mRNA vaccine BNT162b2 are listed in Table 4. These compounds can reverse the transcriptomic signature of the vaccine, which will prevent or reduce side effects. In this study, we wanted to increase the confidence in the computational hypotheses derived from the enrichment and network analyses described earlier.

**Table 4:** Small-molecule drugs and chemical compounds that regulate gene expression in an opposite manner to BNT162b2.

Compound	Score	Description	Links to hypothalamic-pituitary-ovarian function
Droxinostat	−94.51	HDAC inhibitor	^149^
Metyrapone	−93.62	Cytochrome P450 inhibitor	^150,151^
Perospirone	−92.07	Dopamine receptor antagonist	^152^
Nabumetone	−88.8	Cyclooxygenase inhibitor	^153^
Salbutamol	−87.35	Adrenergic receptor agonist	^154,155^
VU-0415374-1	−86.32	Glutamate receptor modulator	^156^
Bromfenac	−83.64	Cyclooxygenase inhibitor	^153,157^
PF-3845	−82.22	FAAH inhibitor	^158^
Hexylresorcinol	−80.48	Local anesthetic	^159^
PPT	−80.07	Estrogen receptor agonist	^160^

## Discussion

This study describes the first attempt to provide a mechanistic insight for vaccine-induced menstrual cycle irregularities. Our approach combined the analysis of vaccine gene expression profiles with upstream predictions of causal regulatory proteins and RNAs, and downstream analysis of enriched biological pathways to provide a causal mechanistic insight for vaccine-induced menstrual irregularities.

Our analysis led to the prioritization of topologically significant genes, such as transcription factors and important enzymes (i.e., kinases) that were largely missed in the gene expression profiling experiment, and therefore were not among the prioritized DEGs. We used the ‘Causal Reasoning’ methodology to identify candidate proteins (i.e., hypotheses) in the network that can be reached through a pre-defined distance (i.e., maximum shortest path length) from the DEGs. Thus, this analysis was crucial for the reconstruction networks responsible for vaccine-induced menstrual irregularities. The top five transcription factors, listed from highest confidence to lower confidence based on their prediction *p*-values, were: IRF1, STAT1, RELA, IRF3 and STAT 2 (Table 1). All were predicted to be activated, in response to BNT162b2 vaccination, based on the directionality of differential gene expression in GS1 and GS2.

IRF1 was ranked first as the highest confidence predicted activated transcription factor. To assess whether changes in IRF1 activation can affect menstrual cycle, we checked whether IRF1 is biomarker for “menstrual cycle irregularity” but we didn’t find evidence to support that. Next, we reviewed the biomedical literature to search for possible links between IRF1 and the menstrual cycle. We used the PubMed’s advanced search using query terms “IRF1” and “menstrual cycle” and found evidence that IRF1 is upregulated by prolactin during the secretory phase of the menstrual cycle^59,60^. Additionally, evidence pointed that IRF1 upregulation in the endometrium was linked to prolactin and is localized predominantly to the granular epithelial cells^59^. Network reconstruction using PLR in addition to seed nodes IRF1, STAT1, RELA, IRF3 and STAT led to the direct interactions network in Fig. 3a. Downstream enrichment analysis in biological pathways, highlighted the prolactin signaling pathway as the most significantly enriched pathway with the six network seeds mentioned above.

Thus, upstream causal reasoning followed by downstream pathways analysis highlighted a putative role for prolactin signaling in modulating post-COVID-19-vaccine adverse events on the menstrual cycle. Prolactin is a multi-functional molecule; it is a transcription factor hormone, secreted from the pituitary glands, and it regulates diverse biological functions including female menstruation^61–69^. For example, high prolactin levels can interfere with the production of sex hormones including estrogen and progesterone which can further impact menstruation regulation^61–69^. In fact, women who experience menstrual cycle irregularities often have higher prolactin levels than others, a condition known as hyperprolactinemia^47^. Hyperprolactinemia, is the most prevalent endocrine dysfunction of the hypothalamic-pituitary axis in young females, accompanied with ovulatory disorder and leading to menstrual irregularities^70,71^. High levels of prolactin in the body prevent the release of (luteinizing hormone) LH and follicle-stimulating hormone (FSH), leading to ovulation disturbances^62,65,69^. Symptoms of hyperprolactinemia include long or irregular cycles, anovulation, amenorrhea, oligomenorrhea, polycystic ovarian syndrome or amenorrhea^72–78^. In fact, hyperprolactinemia can be caused by some drugs, stress, and some conditions like prolactinoma (noncancerous tumor of the pituitary gland)^74,79–81^. All these factors were found to cause inconsistencies in menstrual cyclicity^51,82–84^.

Although we perceive menstrual changes as adverse events, prolactin-mimicking effects of vaccine are not necessarily a negative consequence of vaccines. Recently, prolactin has been suggested as a promising immunomodulator for the treatment of COVID-19^85^. However, we caution that prolactin mimicking effects may worsen auto-immune disease symptoms in patients suffering from systemic lupus erythematosus (SLE), multiple sclerosis, rheumatoid arthritis, psoriatic arthritis, and AIDS. Caution should be also practiced in patients undergoing organ transplantation. Elevated PRL levels have been reported in the previous conditions^86,87^. Furthermore, a recent study showed that prolactin hormones in addition to FSH and LH of healthy vaccinated males were higher than non-vaccinated males or COVID-19 male patients, indicating that changes in prolactin signaling are not limited to females^88^. Prolactin levels were 27.86 ± 4.35 ng/L in vaccinated males, 5.35 ± 1.59 ng/L in non-vaccinated males, and 16.65 ± 6.15 ng/L in COVID-19 male patients^88^.

To identify biomolecules that are implicated in menstrual changes, or the pathological processes that underlie the observed vaccine-induced menstrual symptoms, we filtered all predicted causal molecules and DEGs based on their overlaps with “menstruation irregularity”/“menstruation abnormality” diagnostic and prognostic biomarkers found in CDDI (Fig. 2a). We had four gene lists: 1) all DEGs in GS1 and GS2, 2) causal hubs for DEGs predicted for GS1, 3) causal hubs predicted for GS2, and 4) known diagnostic biomarkers for menstrual irregularity. TNF was identified as a high-confidence hit, i.e., a causal protein leading to the observed changes in gene expression and the predicted prolactin mimicking effects of the vaccine. TNF was an overlapping gene among four gene lists: 1) a DEG (log_2_FC = 3.07), 2) a causal key hub considering DEGs in both GS1, 3) a causal hub considering DEGs in GS2, and 4) a diagnostic biomarker for menstrual irregularity. Furthermore, our causal reasoning results predicted TNF-alpha activation in response to vaccination with BNT162b2. It should be noted that all causal predictions (Supplementary Tables 1 & 2) are based on experimental gene expression data.

Finally, SPIA results combined the enrichment results of DEGs with the actual amount of perturbation which highlighted the role of interferons on the signaling pathways influenced by BNT162b2. In fact, mRNA and vector-based COVID-19 vaccines result in the formation of neutralizing antibodies and activation of immune cells via the release of pro-inflammatory markers like cytokines and interferons^89^. There is evidence indicating that the treatment of multiple sclerosis with beta interferons causes menstrual irregularities associated with increased levels of luteinizing hormone (LH) and/or hyperprolactinemia^90^. Furthermore, the upregulation of interferon-gamma perturbs calcium signaling pathways which can in turn impact hormonal balance^12^.

But what is the relationship between prolactin signaling, TNF-alpha activation and interferons? In fact, TNF-alpha activates the human prolactin gene promoter via NF-κB signaling^91^. TNF-alpha activation also stimulates the hypothalamic-pituitary-adrenal axis while suppressing the hypothalamic-pituitary-thyroid and gonadal axes, and growth hormone release^92^. Menstrual bleeding (menses) is known to be regulated by hypothalamic and pituitary hormones, and even the slightest changes in hormone levels, e.g., in response to medication or stress, can result in menstrual cycle abnormalities^93^. There is evidence that TNF-alpha and interleukin 1 beta (IL-1B), both are upregulated DEGs in this analysis, exert significant inhibitory effects on the GnRH-LH system in rats^94^, which may be the case in humans too. Moreover, the occurrence of reproductive disorders in poultry is highly correlated with the HPO axis and neuro–endocrine–immune network molecules, such as TNF-alpha and interferon-gamma (IFN-γ, IFNG)^95^. Thus, integrating enrichment and causal reasoning results with SPIA findings uncovered causal relationships between BNT162b2-induced menstrual changes and all the following pathways: prolactin signaling pathways, TNF-alpha activation, interferons the hypothalamic-pituitary-gonadal/ovarian/testicular axis. These results agree with previous studies suggesting that stabilizing the hypothalamic-pituitary-ovarian (HPO) axis with combined hormonal contraception reduces the chance of experiencing vaccine-associated menstrual changes^38,96^.

Different lines of supporting evidence increased the confidence in the derived causal hypothesis implicating menstrual changes with prolactin signaling, TNF-alpha and the HPO axis. First, VAERS data showed that post COVID-19 menstrual changes occurred in response all COVID-19 vaccines included in the databases including mRNA and vector-based vaccines and were not tied to the vaccine platform. The menstrual changes reported in VAERS included wide range of symptoms and were not limited to the length of menstrual cycle or menses period. Secondly, PubMed Abstract Sifter results highlighted 299,927 articles linking DEG TNF to any menstrual symptoms and 141 articles linking TNF to abnormal menses. Other high-confidence causal DEGs were progesterone receptor (PGR) with 45,560 and 796 articles linking it to any menstrual symptoms or abnormal menses subsequently, IL-1B with 56,099 and 43 articles linking it to any menstrual symptoms or abnormal menses subsequently. Finally, chemogenomics evidence from the CMap highlighted significant links to the HPO axis per results shown is Table 4.

It should be noted that the transcriptomics perturbations in response to treatment with BNT162b2 diminished on day 28 after receiving the second vaccine dose of BNT162b2. This suggests that vaccine effects on gonadal hormones, for females and males, and the predicted prolactin-mimicking effects, TNF-alpha activation, and HPO signaling changes, were temporary. However, we cannot rule out long-term effects without clinical studies comparing vaccinated and non-vaccinated individuals. Moreover, because our bioinformatics analysis relied on BNT162b2 transcriptomics data, we emphasize that these findings primarily apply to the BNT162b2 vaccines. However, data mined from VAERS, and the biomedical literature indicated that vaccine-induced menstrual cycle changes were reported for other COVID-19 vaccines (e.g., mRNA-1273, and Janssen’s) and non-COVID-19 vaccines (e.g., HPV and typhoid).

In conclusion, our integrative computational network biology approach revealed that BNT162b2 can induce transcriptomics changes which may induce menstrual cycle changes by several mechanisms including prolactin-mimicking effects resulting from changes in interferon signaling and associated hormonal imbalance particularly in the HPO axis. This remains a high-confidence biological hypothesis supported by different lines of computational evidence derived from transcriptomics studies, causal reasoning analysis, downstream pathway enrichment results, and additional supporting evidence from vaccine adverse event databases (e.g., VAERS) and the biomedical literature. Further experimental validation is warranted to assess whether post-vaccine prolactin-mimicking effects are due to increased levels of prolactin or due to other networking biology events mimicking prolactinemia. These effects may not be restricted to COVID-19 vaccines and should be assessed for other vaccines as well.

This study sheds the light on post-vaccine menstrual irregularity by revealing short-term post-COVID-19 vaccine prolactin mimicking effects resulting from the transcriptomics irregularities induced by COVID-19 vaccines. Most women associate menstruation irregularities with infertility which is one of the leading causes of vaccine hesitancy among females^97^. By providing a mechanistic insight into post-vaccine menstrual irregularities, this study is promised to correct misinformation about the relationship between vaccine-induced period irregularities and infertility. Thus, it is expected to decrease vaccine hesitancy.

This study establishes a causal relationship between COVID-19 vaccine and menstruation regulation by highlighting perturbed gene expression or dysregulated transcription of known diagnostic or prognostic biomarkers for menstruation and menstruation irregularities. Additionally, top scoring key hubs provide valuable hypotheses explaining gene expression and can be explored further in laboratory tests.

This study explores the causal links between COVID-19 vaccines and menstruation regulation based on an integrative bioinformatics approach that analyzed vaccine-induced transcriptomics irregularities. Integrating COVID-19 vaccine transcriptomics data with menstruation biomarkers, reinforced the selection of biologically relevant hypotheses from an overwhelming number of statistically significant hypotheses by increasing the confidence in computational hypotheses predicted by several methods. The fact that our computational hypotheses were supported by multiple lines of evidence is considered a major strength for this study. In fact, our integrative informatics workflow has several advantages over relying solely on conventional enrichment analyses for identifying the biological mechanisms that underlie vaccine side effects. Our approach integrates hypotheses derived independently from pathway and network enrichments, causal reasoning, SPIA, and the CMap to prioritize high confidence computational hypotheses predicted independently by various computational approaches and using different data types. The CMap, for example, is considered a unique chemogenomics database capable of connecting genes, drugs, and diseases based on genes expression similarities between polypharmacologic drugs and studied vaccines. This permits the prediction of vaccine side effects as well as underling causal mechanisms based on gene expression similarities with well-studied drugs. Finally, mining VAERS and PubMed for adverse event reports and vaccine-relevant data, serves as a validation step for the computationally-derived hypotheses. Thus, computational hypotheses prioritized using our integrative informatics approach are inherently high-confidence hypotheses with potentially improved clinical outcome.

Conversely, the applied methodologies or public databases have a few limitations that should be pointed out. First, reports from VAERS may not be conclusive or sufficient to establish causal relationships adverse events and specific vaccines. Due to the voluntary nature of VAERS reporting system, the information provided about an adverse event can be imperfect, imprecise, coincidental, or unconfirmed, limiting the scientific use of such reports^11,12^. Secondly, bioinformatics techniques relying on gene expression, pathway over-representation and network biology have some limitations and biases that we reviewed previously elsewhere^43^. Herein, the main limitation for the generalizability of the bioinformatics results to other COVID-19 vaccines, was the reliance on transcriptional data for the mRNA COVID-19 vaccine BNT162b2, which was the only publicly available COVID-19 transcriptomics data in humans at the time of conducting this research. As a result, our bioinformatics results apply directly to BNT162b2 or and may be extended to other COVID-19 mRNA vaccines (e.g., mRNA-1273) since COVID-19 mRNA vaccines share common features of the nature, strength, and timing of the immune responses as well as similar vaccine compositions^7,8,12,18,44^. The dosing regimens of vaccines may affect the results as well^30,89^. Our integrative workflow can be used to assess the safety of other vaccines using their transcriptional signatures in vaccinated individuals.

## Methods

### Systems biology informatics workflow

We have developed a network biology workflow to identify causal links between COVID-19 Vaccines and menstruation irregularities. This workflow (Fig. 1) incorporates three major components: (1) a module for mining and prioritizing gene signatures representative of a condition or a biological state; (2) a causal reasoning network module to identify upstream regulators of gene expression and (3) a pathway enrichment module to understand the biological processes regulated by DEGs and predicted causal regulators of gene expression. The resulting hypotheses are then evaluated based on existing evidence in vaccine reporting system databases and the biomedical literature.

### Vaccine-induced differential gene expression

We searched the gene expression omnibus (GEO)^98–101^ for transcriptional studies performed in response to treatment COVID-19 vaccines and we were able to identify one whole transcriptomics RNA-seq dataset (GSE169159) for COVID-19 vaccines in response to treatment with BNT162b2 at different time points. Our transcriptomics data analysis of GSE169159 raw data indicated that gene expression alterations from baseline were more prominent on day 22, which is the day after receiving the vaccine second dose. None of the genes analyzed at other time points (e.g., day 7, day 21, day, day 22.23, day 28) passed the applied thresholds for the prioritization of DEGs in this study (i.e., log_2_ fold change (log_2_FC) of +2 or –2, and false discovery rate (FDR) $\le$ 0.05. Therefore, we relied on differential gene expressions on day 22 for all our bioinformatics analyses.

Gene expression profiles on day 22 were used to generate two query gene signatures to study the systems biology effects of BNT162b2: GS1 and GS2. GS1 consisted of all differentially expressed genes (DEGs)^102–104^ that passed our prioritization criteria for DEGs: 1) log_2_ FC $\ge$ 2.00 for differentially upregulated genes, and $\le \,$−2.00 for differentially downregulated genes; 2) FDRs $\le \,$0.05. GS2 consisted of all differentially expressed genes (DEGs) that passed our prioritization criteria for DEGs: 1) log_2_FC $\ge$ 5.00 for differentially upregulated genes, and $\le \,$−5.00 for differentially downregulated genes; 2) FDRs $\le \,$0.05. The DEGs used to derive GS1 and GS2 are provided in Supplementary Table 8.

### Upstream transcriptomics analysis

Causal Reasoning^45,105^ was used to identify upstream regulators (transcription factors, RNA molecules, kinases, phosphatases, and others proteins) that could cause/explain the observed post-vaccine gene expression changes in transcriptomics experiments. We relied on MetaBase^106,107^ as an interactions database, and the causal reasoning algorithm implemented in Clarivate’s Key Pathway Advisor^103,108^. This method relies on a directed network which is annotated with activation and inhibition edges as well as biological mechanisms (transcription regulation). The significance of the predictions made by a particular hypothesis is assessed using a binomial test and calculating *p*-values as probabilities to get k successes in n predictions using binomial tests with *p*-value = 0.50 according to the following equation:

1 $$p{{\mbox{-}}}{value}=\left(\frac{n}{k}\right){p}^{k}\,{(1-p)}^{n-k}$$

Here, k is the sum of correct predictions and n is the sum of correct and incorrect predictions.

Finally, *p*-values are assigned in the score matrix and hypotheses above the *p*-value threshold are filtered out of the score matrix.

### Downstream pathway analysis

Pathway enrichment analyses were conducted in Cytoscape version 3.9.1^109^ and MetaCore^TM^^45^ to interpret the consequences of vaccine-induced differential gene expression on biological processes. The significance of the enrichment was determined by calculating hypergeometric *p*-values^110^. All terms from the ontology were ranked based on their calculated *p* values. Ontology terms with *p*-values less than the *p*-value threshold 0.05 are defined as statistically significant and therefore relevant to the studied list of genes. All terms from the ontology were ranked according to their calculated *p*-values.

### Signaling pathway impact analysis (SPIA)

SPIA^111,112^ was performed to identify the impact of the DEGs on the activity of the enriched pathway. This method aids in the identification of the most biologically relevant pathways and candidate causal genes. Herein, we identified perturbed pathways in response to vaccination by performing the enrichment analysis on the union gene list consisting of the experimentally derived DEGs in response to vaccination with BNT162b2, and the list of key hubs (e.g., activated, or inhibited proteins) using causal reasoning.

### Vaccine adverse events database

Raw data files were downloaded in comma-separated value (CSV) files from the CDC website^106,107^. CDC WONDER online search tool was used to mine VAERS data by vaccine type and symptoms^108^. The COVID-19 vaccines included in the databases were: BNT162b2, mRNA-1273 ’s and Janssen’s^113,114^.

### PubMed Abstract Sifter

The advanced literature retrieval tool PubMed Abstract Sifter was used to explore relationships between the biological concepts and molecular concepts that play roles in this research area. The steps in using the Abstract Sifter are described in the user guide. The tool and the user guide are available from the US EPA and downloadable from this webpage: https://comptox.epa.gov/dashboard/downloads^56,115–117^.

### The Connectivity Map (CMap)

The Connectivity Map analysis suggests that drugs capable of inducing transcriptomics effects opposite to those induced by mRNA vaccines could reverse vaccine side effects^57,58^. To identify small-molecule drugs that could prevent or reverse vaccine’s side effects, we ranked all DEGs in response to vaccination by BNT162b2 according to their expression levels using log_2_FC values, to query the Connectivity Map database^59^. In fact, our transcriptomics data analysis of GSE169159 indicated that gene expression alterations from baseline were more prominent on day 22, which is the day after receiving the vaccine second dose. None of the genes analyzed at other time points passed the applied thresholds for identifying DEGs (i.e., log2 fold change (log2FC) of +2 or –2, and false discovery rate (FDR) $\le$ 0.05. Therefore, we relied on differential gene expressions on day 22 for all our bioinformatics analyses.

### Reporting summary

Further information on research design is available in the Nature Research Reporting Summary linked to this article.

### Supplementary information

Supplemental Information

Reporting Summary

## Data Availability

All supplementary data files can be accessed through the journal’s website and are available on GitHub (https://github.com/rhajjo/npjVaccines).

## Code availability

All used codes for CMap, Cytoscape, and VAERS, are publicly available and can be publicly accessed by any user according to the provided details and citations in the methods section. All codes related to CDDI, MetaCore^TM^, and SPIA, are commercial codes that are licensed by Clarivate Analytics. The code for PubMed Abstract Sifter and the user guide is available from the US EPA and downloadable from this webpage: https://comptox.epa.gov/dashboard/downloads.

## References

[1] Astuti I, Ysrafil (2020). Severe Acute Respiratory Syndrome Coronavirus 2 (SARS-CoV-2): An overview of viral structure and host response. Diabetes Metab. Syndr..

[2] Sabbah DA, Hajjo R, Bardaweel SK, Zhong HA (2021). An updated review on SARS-CoV-2 main Proteinase (MPro): protein structure and small-molecule inhibitors. Curr. Top. Med. Chem..

[3] Teijaro JR, Farber DL (2021). COVID-19 vaccines: modes of immune activation and future challenges. Nat. Rev. Immunol..

[4] Al-Qerem W (2022). Parents’ attitudes, knowledge and practice towards vaccinating their children against COVID-19: a cross-sectional study. Hum. Vaccin. Immunother..

[5] Zhang C, Maruggi G, Shan H, Li J (2019). Advances in mRNA vaccines for infectious diseases. Front. Immunol..

[6] Wang J, Peng Y, Xu H, Cui Z, Williams RO (2020). The COVID-19 vaccine race: challenges and opportunities in vaccine formulation. AAPS PharmSciTech.

[7] Verbeke R, Lentacker I, De Smedt SC, Dewitte H (2021). The dawn of mRNA vaccines: The COVID-19 case. J. Control. Release.

[8] Teo SP (2021). Review of COVID-19 mRNA Vaccines: BNT162b2 and mRNA-1273. J. Pharm. Pract..

[9] Sumirtanurdin R, Barliana MI (2021). Coronavirus disease 2019 vaccine development: an overview. Viral Immunol..

[10] Rawat K, Kumari P, Saha L (2020). COVID-19 vaccine: A recent update in pipeline vaccines, their design and development strategies. Eur. J. Pharmacol..

[11] Hajjo R, Sabbah DA, Bardaweel SK, Tropsha A (2021). Shedding the Light on Post-Vaccine Myocarditis and Pericarditis in COVID-19 and Non-COVID-19 Vaccine Recipients. Vaccines.

[12] Hajjo R, Sabbah DA, Tropsha A (2022). Analyzing the Systems Biology Effects of COVID-19 mRNA Vaccines to Assess Their Safety and Putative Side Effects. Pathogens.

[13] Saleem A, Javed SO, Malik F (2022). COVID-19 vaccine related menstrual irregularities: A cause of vaccine hesitation. J. Pak. Med. Assoc..

[14] Male V (2022). Menstruation and covid-19 vaccination. BMJ.

[15] Hunter PR (2021). Thrombosis after covid-19 vaccination. BMJ.

[16] Male V (2021). Menstrual changes after covid-19 vaccination. BMJ.

[17] Temiz SA (2022). Cutaneous and allergic reactions due to COVID‐19 vaccinations: a review. J. Cosmet. Dermatol..

[18] Warren CM (2021). Assessment of allergic and anaphylactic reactions to mRNA COVID-19 vaccines with confirmatory testing in a US regional health system. JAMA Netw. Open.

[19] Ünsal H, Şekerel BE, Şahiner ÜM (2021). Allergic reactions against Covıd-19 vaccines. Turk. J. Med. Sci..

[20] Ring J (2021). & de Bruin‐Weller, M. Risk of severe allergic reactions to COVID‐19 vaccines among patients with allergic skin diseases–practical recommendations. A position statement of ETFAD with external experts. J. Eur. Acad. Dermatol. Venereol..

[21] McMahon DE (2021). Cutaneous reactions reported after Moderna and Pfizer COVID-19 vaccination: A registry-based study of 414 cases. J. Am. Acad. Dermatol..

[22] Harrison SL, Buckley BJR, Rivera-Caravaca JM, Zhang J, Lip GYH (2021). Cardiovascular risk factors, cardiovascular disease, and COVID-19: an umbrella review of systematic reviews. Eur. Heart J..

[23] García JB (2021). Acute myocarditis after administration of the BNT162b2 vaccine against COVID-19. Rev. Esp. Cardiol..

[24] Ong EZ (2022). Immune gene expression analysis indicates the potential of a self-amplifying Covid-19 mRNA vaccine. npj Vaccines.

[25] Ozimek N (2021). Impact of stress on menstrual cyclicity during the COVID-19 pandemic: a survey study. J. Women’s Health.

[26] Jing Y (2020). Potential influence of COVID-19/ACE2 on the female reproductive system. Mol. Hum. Reprod..

[27] Manzoor K (2022). Oxidative Stress and Menstrual Complications Caused by Vaccination of COVID-19 Among Females Athletes. Cumhur. Med. J..

[28] Sharp GC (2022). The COVID-19 pandemic and the menstrual cycle: research gaps and opportunities. Int. J. Epidemiol..

[29] Li K (2021). Analysis of sex hormones and menstruation in COVID-19 women of child-bearing age. Reprod. Biomed. Online.

[30] Edelman A (2022). Association Between Menstrual Cycle Length and Coronavirus Disease 2019 (COVID-19) Vaccination: A U.S. Cohort. Obstet. Gynecol..

[31] Prado RCR, Silveira R, Asano RY (2021). SARS-CoV-2 (COVID-19) pandemic and a possible impact in the future of menstrual cycle research. Health Sci. Rep..

[32] Farland LV (2023). COVID-19 vaccination and changes in the menstrual cycle among vaccinated persons. Fertil. Steril..

[33] Edelman, A., et al. Association between menstrual cycle length and covid-19 vaccination: global, retrospective cohort study of prospectively collected data. *BMJ Med*. **1**, 10.1136/bmjmed-2022-000297 (2022). 10.1136/bmjmed-2022-000297 PMC9665108 36381261

[34] Wang S (2022). A prospective study of the association between SARS-CoV-2 infection and COVID-19 vaccination with changes in usual menstrual cycle characteristics. Am. J. Obstet. Gynecol..

[35] Kurdoğlu Z (2021). Do the COVID-19 vaccines cause menstrual irregularities. Int. J. Women’s Health Reprod. Sci..

[36] Lee KMN (2022). Investigating trends in those who experience menstrual bleeding changes after SARS-CoV-2 vaccination. Sci. Adv..

[37] Phelan N, Behan LA, Owens L (2021). The Impact of the COVID-19 Pandemic on Women’s Reproductive Health. Front. Endocrinol. (Lausanne).

[38] Alvergne A, Woon EV, Male V (2022). Effect of COVID-19 vaccination on the timing and flow of menstrual periods in two cohorts. Front. Reprod. Health.

[39] Turnbull AV, Rivier CL (1999). Regulation of the hypothalamic-pituitary-adrenal axis by cytokines: actions and mechanisms of action. Physiol. Rev..

[40] Karagiannis A, Harsoulis F (2005). Gonadal dysfunction in systemic diseases. Eur. J. Endocrinol..

[41] Gong L (2020). Human papillomavirus vaccine-associated premature ovarian insufficiency and related adverse events: data mining of Vaccine Adverse Event Reporting System. Sci. Rep..

[42] Lamb, A. R. Experiences with Prophylactic Typhoid Vaccination: It’s Effect on Menstruation. *Arch. Intern. Med*. **XII**, 565-577, 10.1001/archinte.1913.00070050082008 (1913).

[43] Hajjo R, Tropsha A (2020). A Systems Biology Workflow for Drug and Vaccine Repurposing: Identifying Small-Molecule BCG Mimics to Reduce or Prevent COVID-19 Mortality. Pharm. Res..

[44] Arunachalam PS (2021). Systems vaccinology of the BNT162b2 mRNA vaccine in humans. Nature.

[45] Chindelevitch L (2012). Causal reasoning on biological networks: interpreting transcriptional changes. Bioinformatics.

[46] *Clarivate. Introducing Cortellis Drug Discovery Intelligence*. https://clarivate.com/cortellis/campaigns/introducing-cortellis-drug-discovery-intelligence/#.

[47] Kulshreshtha B (2017). Menstrual Cycle Abnormalities in Patients with Prolactinoma and Drug-induced Hyperprolactinemia. Indian J. Endocrinol. Metab..

[48] Farland LV (2017). Menstrual cycle characteristics and steroid hormone, prolactin, and growth factor levels in premenopausal women. Cancer Causes Control.

[49] Bargiota S, Bonotis K, Messinis I, Angelopoulos N (2013). The effects of antipsychotics on prolactin levels and women’s menstruation. Schizophr. Res..

[50] Mishra R, Baveja R, Gupta V, Gupta P (2002). Prolactin level in infertility with menstrual irregularities. J. Obstet. Gynecol. India.

[51] Yazigi RA, Quintero CH, Salameh WA (1997). Prolactin disorders. Fertil. Steril..

[52] Riley BE (2014). Systems-based analyses of brain regions functionally impacted in Parkinson’s disease reveals underlying causal mechanisms. PLoS One.

[53] Li RX (2016). Perimenopausal syndrome and mood disorders in perimenopause: prevalence, severity, relationships, and risk factors. Med. (Baltim.).

[54] Warren MP, Shantha S (2000). The female athlete. Baillieres Best. Pract. Res. Clin. Endocrinol. Metab..

[55] Bae J, Park S, Kwon JW (2018). Factors associated with menstrual cycle irregularity and menopause. BMC Women’s Health.

[56] Baker N, Knudsen T, Williams A (2017). Abstract Sifter: a comprehensive front-end system to PubMed. F1000Res.

[57] Lamb J (2007). The Connectivity Map: a new tool for biomedical research. Nat. Rev. Cancer.

[58] Lamb J (2006). The Connectivity Map: using gene-expression signatures to connect small molecules, genes, and disease. Science.

[59] Jabbour HN, Critchley HO, Yu-Lee LY, Boddy SC (1999). Localization of interferon regulatory factor-1 (IRF-1) in nonpregnant human endometrium: expression of IRF-1 is up-regulated by prolactin during the secretory phase of the menstrual cycle. J. Clin. Endocrinol. Metab..

[60] Lobo SC (2004). The immune environment in human endometrium during the window of implantation. Am. J. Reprod. Immunol..

[61] Maslar IA, Riddick DH (1979). Prolactin production by human endometrium during the normal menstrual cycle. Am. J. Obstet. Gynecol..

[62] Bäckström C, McNeilly A, Leask R, Baird D (1982). Pulsatile secretion of LH, FSH, prolactin, oestradiol and progesterone during the human menstrual cycle. Clin. Endocrinol..

[63] Vekemans M, Delvoye P, Lhermite M, Robyn C (1977). Serum prolactin levels during the menstrual cycle. J. Clin. Endocrinol. Metab..

[64] Franchimont P (1976). Prolactin levels during the menstrual cycle. Clin. Endocrinol..

[65] Lenton EA, Sulaiman R, Sobowale O, Cooke I (1982). The human menstrual cycle: plasma concentrations of prolactin, LH, FSH, oestradiol and progesterone in conceiving and non-conceiving women. Reproduction.

[66] Tyson J, Hwang P, Guyda H, Friesen HG (1972). Studies of prolactin secretion in human pregnancy. Am. J. Obstet. Gynecol..

[67] Hwang P, Guyda H, Friesen H (1971). A radioimmunoassay for human prolactin. Proc. Natl Acad. Sci. USA.

[68] McNeilly A, Chard T (1974). Circulating levels of prolactin during the menstrual cycle. Clin. Endocrinol..

[69] Tanner MJ, Hadlow NC, Wardrop R (2011). Variation of female prolactin levels with menopausal status and phase of menstrual cycle. Aust. N. Z. J. Obstet. Gynaecol..

[70] Lee D-Y, Oh Y-K, Yoon B-K, Choi D (2012). Prevalence of hyperprolactinemia in adolescents and young women with menstruation-related problems. Am. J. Obstet. Gynecol..

[71] Casanueva FF (2006). Guidelines of the Pituitary Society for the diagnosis and management of prolactinomas. Clin. Endocrinol. (Oxf.).

[72] Biller BM (1999). Guidelines for the diagnosis and treatment of hyperprolactinemia. J. Reprod. Med..

[73] Halperin Rabinovich I, Cámara Gómez R, García Mouriz M, Ollero García-Agulló D (2013). [Clinical guidelines for diagnosis and treatment of prolactinoma and hyperprolactinemia]. Endocrinol. Nutr..

[74] Melmed S (2011). Diagnosis and treatment of hyperprolactinemia: an Endocrine Society clinical practice guideline. J. Clin. Endocrinol. Metab..

[75] Vilar L, Vilar CF, Lyra R, Freitas MDC (2019). Pitfalls in the Diagnostic Evaluation of Hyperprolactinemia. Neuroendocrinology.

[76] Capozzi A, Scambia G, Pontecorvi A, Lello S (2015). Hyperprolactinemia: pathophysiology and therapeutic approach. Gynecol. Endocrinol..

[77] Matalliotakis M, Koliarakis I, Matalliotaki C, Trivli A, Hatzidaki E (2019). Clinical manifestations, evaluation and management of hyperprolactinemia in adolescent and young girls: a brief review. Acta Biomed..

[78] Chahal J, Schlechte J (2008). Hyperprolactinemia. Pituitary.

[79] Dickson RA, Glazer WM (1999). Neuroleptic-induced hyperprolactinemia. Schizophr. Res..

[80] Luciano AA (1999). Clinical presentation of hyperprolactinemia. J. Reprod. Med..

[81] Mah PM, Webster J (2002). Hyperprolactinemia: etiology, diagnosis, and management. Semin. Reprod. Med..

[82] Schlechte J (1980). Prolactin-secreting pituitary tumors in amenorrheic women: a comprehensive study. Endocr. Rev..

[83] Berinder K, Stackenäs I, Akre O, Hirschberg AL, Hulting AL (2005). Hyperprolactinaemia in 271 women: up to three decades of clinical follow-up. Clin. Endocrinol. (Oxf.).

[84] Christodoulopoulou V (2016). Clinical and Biochemical Characteristics in PCOS Women With Menstrual Abnormalities. J. Fam. Reprod. Health.

[85] Sen A (2020). Repurposing prolactin as a promising immunomodulator for the treatment of COVID-19: Are common Antiemetics the wonder drug to fight coronavirus?. Med. Hypotheses.

[86] Kanik KS, Wilder RL (2000). Hormonal alterations in rheumatoid arthritis, including the effects of pregnancy. Rheum. Dis. Clin. North Am..

[87] Alvarez-Nemegyei J (1998). Bromocriptine in systemic lupus erythematosus: a double-blind, randomized, placebo-controlled study. Lupus.

[88] Najeb HB (2022). The Study of Effect the Covid-19 Virus and the Vaccine Against it on Men’s Sex Hormones. HIV Nurs..

[89] Nazir M (2022). Menstrual abnormalities after COVID-19 vaccines: A systematic review. Vacunas.

[90] Nabavi SM, Koupai SA, Nejati MR, Garshasbi E, Jalali MR (2010). Menstrual irregularities and related plasma hormone levels in multiple sclerosis patients treated with beta interferone. Acta Med. Iran..

[91] Friedrichsen S (2006). Tumor necrosis factor-alpha activates the human prolactin gene promoter via nuclear factor-kappaB signaling. Endocrinology.

[92] Jones TH, Kennedy RL (1993). Cytokines and hypothalamic-pituitary function. Cytokine.

[93] *AMBOSS. The menstrual cycle and menstrual cycle abnormalities; available from*: https://www.amboss.com/us/knowledge/The_menstrual_cycle_and_menstrual_cycle_abnormalities/ (accessed on 15 May 2023).

[94] Watanobe H, Hayakawa Y (2003). Hypothalamic Interleukin-1β and Tumor Necrosis Factor-α, But Not Interleukin-6, Mediate the Endotoxin-Induced Suppression of the Reproductive Axis in Rats. Endocrinology.

[95] Zhao J (2023). Interacting Networks of the Hypothalamic–Pituitary–Ovarian Axis Regulate Layer Hens Performance. Genes.

[96] Alvergne, A. et al. COVID-19 vaccination and menstrual cycle changes: A United Kingdom (UK) retrospective case-control study. *medRxiv*, 2021.2011.2023.21266709, 10.1101/2021.11.23.21266709 (2021).

[97] Abbasi J (2022). Widespread Misinformation About Infertility Continues to Create COVID-19 Vaccine Hesitancy. JAMA.

[98] Barrett T, Edgar R (2006). [19] Gene Expression Omnibus: microarray data storage, submission, retrieval, and analysis. Meth. Enzymol..

[99] Davis S, Meltzer PS (2007). GEOquery: a bridge between the Gene Expression Omnibus (GEO) and BioConductor. Bioinformatics.

[100] Clough, E. & Barrett, T. In *Statistical genomics* 93–110 (Springer, 2016).

[101] Barrett, T. & Edgar, R. In *Gene Mapping, Discovery, and Expression* 175–190 (Springer, 2006).

[102] Kim Y-J, Kwak C-I, Gu Y-Y, Hwang I-T, Chun J-Y (2004). Annealing control primer system for identification of differentially expressed genes on agarose gels. Biotechniques.

[103] Clark NR (2014). The characteristic direction: a geometrical approach to identify differentially expressed genes. BMC Bioinform.

[104] Broberg P (2003). Statistical methods for ranking differentially expressed genes. Genome Biol..

[105] Pollard J (2005). A computational model to define the molecular causes of type 2 diabetes mellitus. Diabetes Technol. Ther..

[106] Bolser DM (2012). MetaBase—the wiki-database of biological databases. Nucl. Acids Res..

[107] Bernasconi A, Canakoglu A, Masseroli M, Ceri S (2020). META-BASE: a novel architecture for large-scale genomic metadata integration. IEEE/ACM Trans. Comput. Biol. Bioinforma..

[108] Dubovenko, A., Nikolsky, Y., Rakhmatulin, E. & Nikolskaya, T. In *Biological Networks and Pathway Analysis* Vol. New York, USA. 101-124 (Springer, 2017). 10.1007/978-1-4939-7027-8_6 28849560

[109] Shannon P (2003). Cytoscape: a software environment for integrated models of biomolecular interaction networks. Genome Res..

[110] Dubovenko A, Nikolsky Y, Rakhmatulin E, Nikolskaya T (2017). Functional Analysis of OMICs Data and Small Molecule Compounds in an Integrated “Knowledge-Based” Platform. Methods Mol. Biol..

[111] Tarca AL (2009). A novel signaling pathway impact analysis. Bioinformatics.

[112] Fang, H. et al. Signaling pathway impact analysis by incorporating the importance and specificity of genes (SPIA-IS). Comput. Biol. Chem. 71, 236–244 (2017). 10.1016/j.compbiolchem.2017.09.009 28988640

[113] Rudan I, Adeloye D, Sheikh A (2022). COVID-19: vaccines, efficacy and effects on variants. Curr. Opin. Pulm. Med..

[114] Krashias G (2022). SARS CoV-2 vaccination induces antibodies against cardiolipin. BMC Res. Notes.

[115] Kavlock R, Dix D (2010). Computational toxicology as implemented by the U.S. EPA: providing high throughput decision support tools for screening and assessing chemical exposure, hazard and risk. J. Toxicol. Environ. Health B Crit. Rev..

[116] Grulke CM, Williams AJ, Thillanadarajah I, Richard AM (2019). EPA’s DSSTox database: History of development of a curated chemistry resource supporting computational toxicology research. Comput. Toxicol..

[117] Blaszczyk K (2016). The unique role of STAT2 in constitutive and IFN-induced transcription and antiviral responses. Cytokine Growth Factor Rev..

[118] Butts CL (2010). Progesterone regulation of uterine dendritic cell function in rodents is dependent on the stage of estrous cycle. Mucosal. Immunol..

[119] Paravati R (2020). Differential regulation of osteopontin and CD44 correlates with infertility status in PCOS patients. J. Mol. Med. (Berl.).

[120] Song P (2021). Integrated analysis of miRNA-mRNA interaction in ovaries of Turpan Black Sheep during follicular and luteal phases. Reprod. Domest. Anim..

[121] Jabbour HN, Critchley HO, Boddy SC (1998). Expression of functional prolactin receptors in nonpregnant human endometrium: janus kinase-2, signal transducer and activator of transcription-1 (STAT1), and STAT5 proteins are phosphorylated after stimulation with prolactin. J. Clin. Endocrinol. Metab..

[122] Dalrymple A, Jabbour HN (2000). Localization and signaling of the prolactin receptor in the uterus of the common marmoset monkey. J. Clin. Endocrinol. Metab..

[123] Wu B (2014). ROS are critical for endometrial breakdown via NF-κB–COX-2 signaling in a female mouse menstrual-like model. Endocrinology.

[124] Fortes MRS (2018). Pre- and post-puberty expression of genes and proteins in the uterus of Bos indicus heifers: the luteal phase effect post-puberty. Anim. Genet..

[125] King AE (2010). An additive interaction between the NFkappaB and estrogen receptor signalling pathways in human endometrial epithelial cells. Hum. Reprod..

[126] Ponce C (2009). Nuclear factor kappaB pathway and interleukin-6 are affected in eutopic endometrium of women with endometriosis. Reproduction.

[127] Saegusa M, Hashimura M, Suzuki E, Yoshida T, Kuwata T (2012). Transcriptional up-regulation of Sox9 by NF-κB in endometrial carcinoma cells, modulating cell proliferation through alteration in the p14(ARF)/p53/p21(WAF1) pathway. Am. J. Pathol..

[128] Faustmann G (2020). Activation of nuclear factor-kappa B subunits c-Rel, p65 and p50 by plasma lipids and fatty acids across the menstrual cycle. Free Radic. Biol. Med..

[129] González-Ramos R (2012). Physiologic activation of nuclear factor kappa-B in the endometrium during the menstrual cycle is altered in endometriosis patients. Fertil. Steril..

[130] Faustmann G (2018). Progesterone-associated arginine decline at luteal phase of menstrual cycle and associations with related amino acids and nuclear factor kB activation. PLoS One.

[131] Faustmann G (2016). Circulating leptin and NF-κB activation in peripheral blood mononuclear cells across the menstrual cycle. Biofactors.

[132] McKinley-Barnard SK, Andre TL, Gann JJ, Hwang PS, Willoughby DS (2018). Effectiveness of Fish Oil Supplementation in Attenuating Exercise-Induced Muscle Damage in Women During Midfollicular and Midluteal Menstrual Phases. J. Strength Cond. Res..

[133] Slattery ML (2014). Genetic variation in the JAK/STAT/SOCS signaling pathway influences breast cancer-specific mortality through interaction with cigarette smoking and use of aspirin/NSAIDs: the Breast Cancer Health Disparities Study. Breast Cancer Res. Treat..

[134] Lopez-Caraballo L, Martorell-Marugan J, Carmona-Saez P, Gonzalez-Muñoz E (2020). Analysis of menstrual blood stromal cells reveals SOX15 triggers oocyte-based human cell reprogramming. iScience.

[135] Joyce MM (2007). Pig conceptuses increase uterine interferon-regulatory factor 1 (IRF1), but restrict expression to stroma through estrogen-induced IRF2 in luminal epithelium. Biol. Reprod..

[136] Chen XD (2010). Gene expression profiling in monocytes and SNP association suggest the importance of the STAT1 gene for osteoporosis in both Chinese and Caucasians. J. Bone Miner. Res..

[137] Aznaurova YB, Zhumataev MB, Roberts TK, Aliper AM, Zhavoronkov AA (2014). Molecular aspects of development and regulation of endometriosis. Reprod. Biol. Endocrinol..

[138] Almasi MZ (2021). Evaluation of Toll-like receptor 3 (TLR3) signaling pathway genes and its genetic polymorphisms in ectopic and eutopic endometrium of women with endometriosis. J. Gynecol. Obstet. Hum. Reprod..

[139] Soltani N (2020). Assessment of the effect of short-term combined high-intensity interval training on TLR4, NF-κB and IRF3 expression in young overweight and obese girls. Public Health Genomics.

[140] Snijders AM (2014). An interferon signature identified by RNA-sequencing of mammary tissues varies across the estrous cycle and is predictive of metastasis-free survival. Oncotarget.

[141] Fernandez-Gonzalo R, De Paz JA, Rodriguez-Miguelez P, Cuevas MJ, González-Gallego J (2014). TLR4-mediated blunting of inflammatory responses to eccentric exercise in young women. Mediators inflamm..

[142] Kusama K (2022). Toll-like receptor signaling pathway triggered by inhibition of serpin A1 stimulates production of inflammatory cytokines by endometrial stromal cells. Front. Endocrinol..

[143] Cumming HE, Bourke NM (2019). Type I IFNs in the female reproductive tract: The first line of defense in an ever‐changing battleground. J. Leukoc. Biol..

[144] Yang Z, Kong B, Mosser DM, Zhang X (2011). TLRs, macrophages, and NK cells: our understandings of their functions in uterus and ovary. Int. Immunopharmacol..

[145] Amirchaghmaghi E (2013). The role of toll like receptors in pregnancy. Int. J. Fertil. Steril..

[146] Jafari R (2020). Detailed investigation of downstream TLR signaling in the follicular cells of women with endometriosis. J. Reprod. Infertil..

[147] Soltani N, Marandi SM, Kazemi M, Esmaeil N (2020). Combined all-extremity high-intensity interval training regulates immunometabolic responses through toll-like receptor 4 adaptors and A20 downregulation in obese young females. Obes. Facts.

[148] Khan KN (2009). Toll-like receptors in innate immunity: role of bacterial endotoxin and toll-like receptor 4 in endometrium and endometriosis. Gynecol. Obstet. Invest..

[149] Olaniyi KS (2021). Repression of HDAC5 by acetate restores hypothalamic-pituitary-ovarian function in type 2 diabetes mellitus. Reprod. Toxicol..

[150] Givens JR, Andersen RN, Ragland JB, Umstot ES (1976). Effects of norgestrel and metyrapone on pituitary-adrenal-ovarian function. Obstet. Gynecol..

[151] Clayton RN, Bailey LC (1984). Dopamine agonist- and antagonist-induced modulation of pituitary gonadotrophin releasing hormone receptors are independent of changes in serum prolactin. J. Endocrinol..

[152] Kumakura N, Okuzawa K, Gen K, Kagawa H (2003). Effects of gonadotropin-releasing hormone agonist and dopamine antagonist on hypothalamus-pituitary-gonadal axis of pre-pubertal female red seabream (Pagrus major). Gen. Comp. Endocrinol..

[153] Matzkin ME (2012). Prolactin (PRL) induction of cyclooxygenase 2 (COX2) expression and prostaglandin (PG) production in hamster Leydig cells. Mol. Cell. Endocrinol..

[154] Toufexis D, Rivarola MA, Lara H, Viau V (2014). Stress and the reproductive axis. J. Neuroendocrinol..

[155] Arrais RF, Dib SA (2006). The hypothalamus-pituitary-ovary axis and type 1 diabetes mellitus: a mini review. Hum. Reprod..

[156] Maffucci JA, Gore AC (2009). Chapter 2: hypothalamic neural systems controlling the female reproductive life cycle gonadotropin-releasing hormone, glutamate, and GABA. Int. Rev. Cell Mol. Biol..

[157] Walker OS, Holloway AC, Raha S (2019). The role of the endocannabinoid system in female reproductive tissues. J. Ovarian Res..

[158] Lim J, Squire E, Jung KM (2023). Phytocannabinoids, the Endocannabinoid System and Male Reproduction. World J. Mens. Health.

[159] Kim J-Y, Seok H (2020). Role of 4-Hexylresorcinol in the Field of Tissue Engineering. Appl. Sci..

[160] Laws MJ (2014). Dysregulated estrogen receptor signaling in the hypothalamic-pituitary-ovarian axis leads to ovarian epithelial tumorigenesis in mice. PLoS Genet.

