# Supplementary material for: Identifying a causal link between prolactin signaling pathways and COVID-19 vaccine-induced menstrual changes
Source: NPJ Vaccines. 2023 Sep 1;8:129. doi: 10.1038/s41541-023-00719-6 (PMC10474200; doi:10.1038/s41541-023-00719-6)
Supplement: Supplementary file 1 — Supplemental Information [file 41541_2023_719_MOESM1_ESM.pdf]

SUPPLEMENTAL MATERIAL – Supplemental Figures

Identifying a Causal link between Prolactin Signaling Pathways and COVID-19 Vaccine-induced Menstrual Changes

Rima Hajjo[1,2,3*], Ensaf Momani[4], Dima A. Sabbah[1], Nancy Baker[2], Alexander Tropsha[2]

[1]Department of Pharmacy, Faculty of Pharmacy, Al-Zaytoonah University of Jordan, P.O. Box 130 Amman 11733 Jordan.

[2]Laboratory for Molecular Modeling, Division of Chemical Biology and Medicinal Chemistry, Eshelman School of Pharmacy, The University of North Carolina at Chapel Hill

[3]Jordan CDC, Amman, Jordan.

[4]Al Balqa' Applied University, Faculty of Medicine, Al-Salt, Jordan

***Correspondence**

Rima Hajjo, Assistant Professor in Pharmacoinformatics and Pharmacogenetics, Al-Zaytoonah University of Jordan, Amman, Jordan; Adjunct Associate Professor, UNC-Chapel Hill, North Carolina, USA.

Email: r.hajjo@zuj.edu.jo ; hajjo@unc.edu

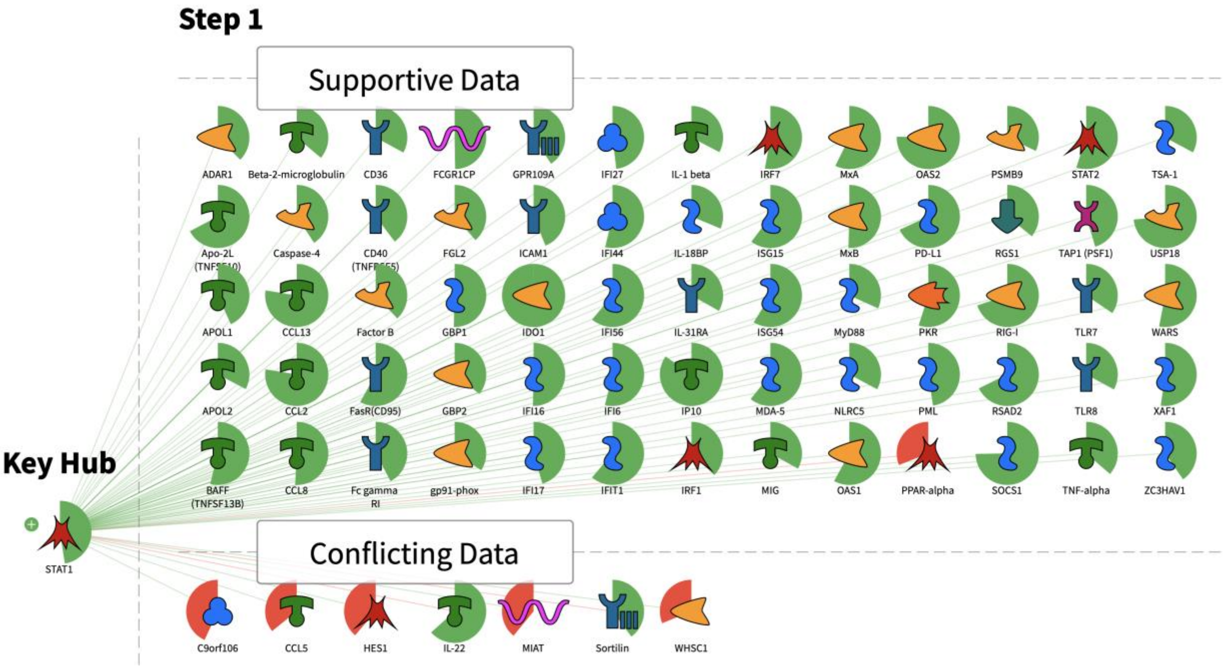

Supplementary Fig. 1: Causal reasoning network of high confidence transcription factor STAT1 using DEGs in GS2. Gene expression changes are shown in green and red sectors around each molecule. Increased expression value corresponds to the green sector which size increases clockwise around the molecule icon. Decreased expression value corresponds to the red sector which size increases counterclockwise. Supportive data panel contains over and under-expressed genes from the experimental data set which support a hypothesis that STAT1 is in a predicted predominant “active” state. Conflicting data panel contains over and under-expressed genes from the experimental data set which are discordant with the hypothesis that STAT1 is in predicted predominant “active” state.

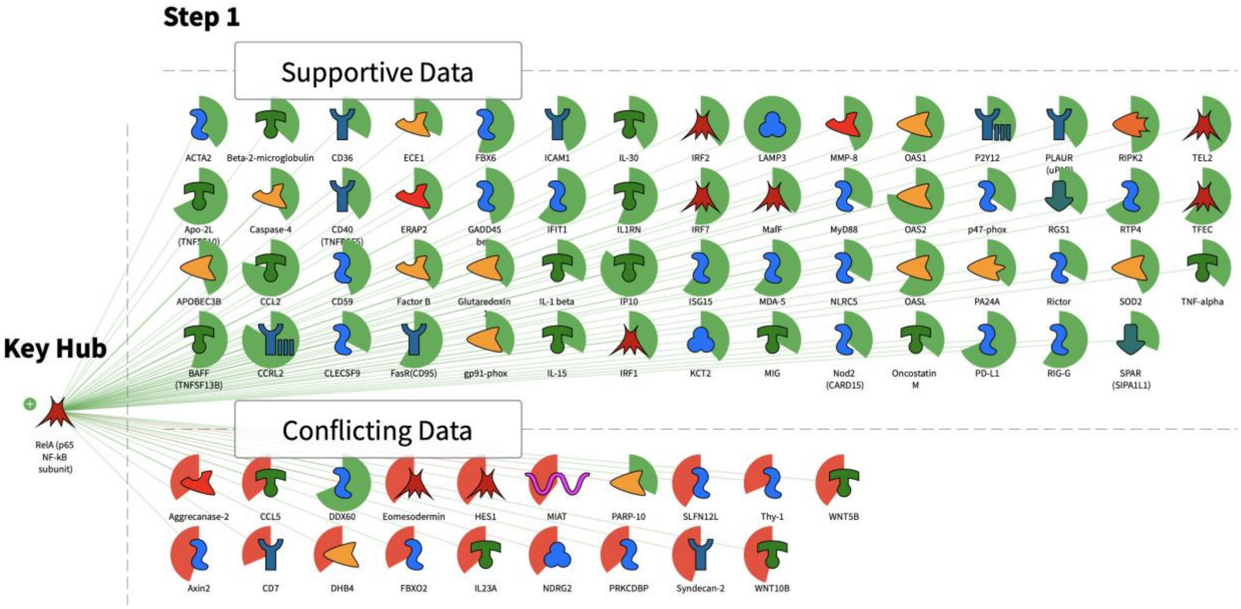

Supplementary Fig. 2: Causal reasoning network of high confidence transcription factor RelA using DEGs in GS2. Gene expression changes are shown in green and red sectors around each molecule. Increased expression value corresponds to the green sector which size increases clockwise around the molecule icon. Decreased expression value corresponds to the red sector which size increases counterclockwise. Supportive data panel contains over and under-expressed genes from the experimental data set which support a hypothesis that RelA is in a predicted predominant "active" state. Conflicting data panel contains over and under-expressed genes from the experimental data set which are discordant with the hypothesis that RelA is in predicted predominant "active" state.

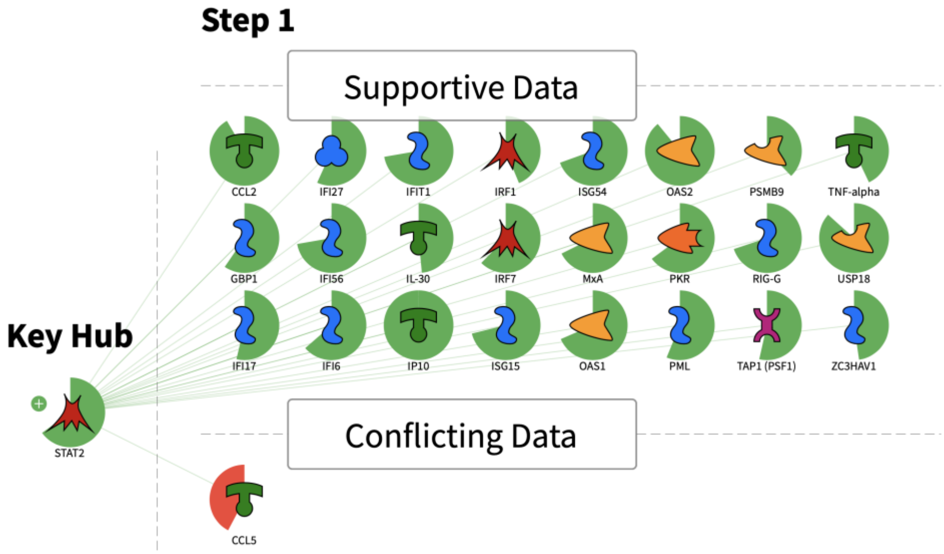

Supplementary Fig. 3: Causal reasoning network of high confidence transcription factor STAT2 using DEGs in GS2. Gene expression changes are shown in green and red sectors around each molecule. Increased expression value corresponds to the green sector which size increases clockwise around the molecule icon. Decreased expression value corresponds to the red sector which size increases counterclockwise. Supportive data panel contains over and under-expressed genes from the experimental data set which support a hypothesis that STAT2 is in a predicted predominant "active" state. Conflicting data panel contains over and under-expressed genes from the experimental data set which are discordant with the hypothesis that STAT2 is in predicted predominant "active" state.

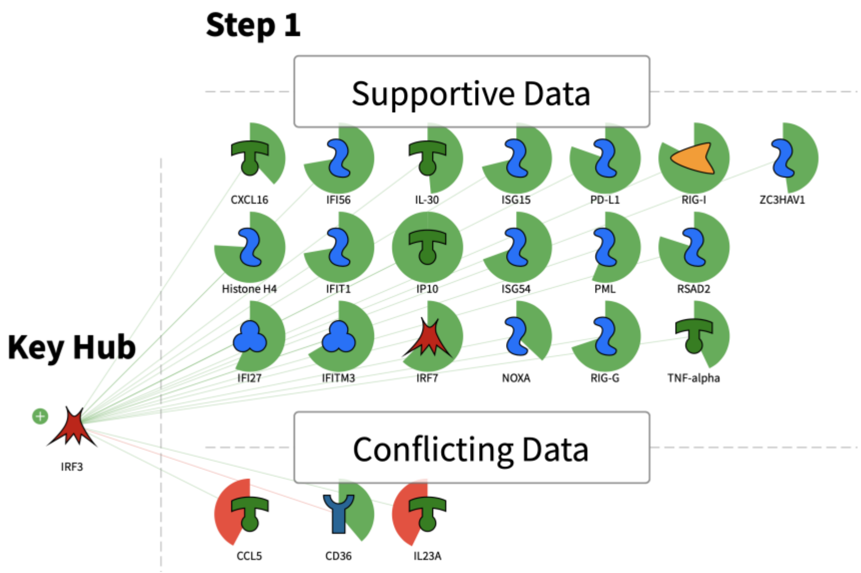

Supplementary Fig. 4: Causal reasoning network of high confidence transcription factor IRF3 using DEGs in GS2. Gene expression changes are shown in green and red sectors around each molecule. Increased expression value corresponds to the green sector which size increases clockwise around the molecule icon. Decreased expression value corresponds to the red sector which size increases counterclockwise. Supportive data panel contains over and under-expressed genes from the experimental data set which support a hypothesis that IRF3 is in a predicted predominant “active” state. Conflicting data panel contains over and under-expressed genes from the experimental data set which are discordant with the hypothesis that IRF3 is in predicted predominant “active” state.

SUPPLEMENTAL MATERIAL – Supplemental Tables

Identifying a Causal link between Prolactin Signaling Pathways and COVID-19 Vaccine-induced Menstrual Changes

Rima Hajjo[1,2,3*], Ensaf Momani[4], Dima A. Sabbah[1], Nancy Baker[2], Alexander Tropsha[2]

[1]Department of Pharmacy, Faculty of Pharmacy, Al-Zaytoonah University of Jordan, P.O. Box 130 Amman 11733 Jordan.

[2]Laboratory for Molecular Modeling, Division of Chemical Biology and Medicinal Chemistry, Eshelman School of Pharmacy, The University of North Carolina at Chapel Hill

[3]Jordan CDC, Amman, Jordan.

[4]Al Balqa' Applied University, Faculty of Medicine, Al-Salt, Jordan

***Correspondence**

Rima Hajjo, Assistant Professor of Pharmacoinformatics and Pharmacogenetics, Al-Zaytoonah University of Jordan, Amman, Jordan; Adjunct Associate Professor, UNC-Chapel

Email: r.hajjo@zuj.edu.jo ; hajjo@unc.edu

Supplementary Table 1. Causal reasoning results using DEGs that has log2FC ≥ 2 or ≤ −2 and FDR ≤ 0.05.

Key Hub						
Rank	Molecular Entity	Gene	Predicted Activity	Correct/Total network predictions	Activity Prediction p-value	Calcul. Distance
1	Siglec-10	SIGLEC10	-	95/105	7.927E-19	3
2	SHP-1	PTPN6	-	93/103	2.599E-18	2
3	IRp60	CD300A	-	93/104	1.243E-17	3
4	IRF1	IRF1	+	60/62	4.237E-16	1
5	miR-502-5p	MIR502	-	66/70	8.251E-16	2
6	ILT2	LILRB1	-	112/135	1.514E-15	3
7	Siglec-7	SIGLEC7	-	112/135	1.514E-15	3
8	BTLA	BTLA	-	112/135	1.514E-15	3
9	SIGLEC5	SIGLEC5	-	112/135	1.514E-15	3
10	CD244	CD244	-	112/135	1.514E-15	3
11	KIR2DL2	KIR2DL2	-	112/135	1.514E-15	3
12	CD33	CD33	-	112/135	1.514E-15	3
13	PIRB	LILRB3	-	112/135	1.514E-15	3
14	PIRB	LOC10798746	-	112/135	1.514E-15	3
16	Siglec-E	SIGLEC9	-	112/135	1.514E-15	3
17	KIR3DL1	KIR3DL1	-	112/135	1.514E-15	3
18	LIRB		-	112/135	1.514E-15	3
19	NKG2A	KLRC1	-	112/135	1.514E-15	3
20	LAIR1	LAIR1	-	112/135	1.514E-15	3
21	KIR2DL1	KIR2DL1	-	112/135	1.514E-15	3
22	ILT4	LILRB2	-	112/135	1.514E-15	3
15	CD84	CD84	-	112/135	1.514E-15	3
23	IFNAR1	IFNAR1	+	73/80	2.898E-15	3
24	Fc gamma RII beta	FCGR2B	-	112/136	4.338E-15	3
25	USP17	USP17L26	+	71/78	9.666E-15	3
26	USP17	USP17L24	+	71/78	9.666E-15	3
27	USP17	USP17L30	+	71/78	9.666E-15	3
28	USP17	USP17L25	+	71/78	9.666E-15	3
29	USP17	USP17L29	+	71/78	9.666E-15	3
30	USP17	USP17L27	+	71/78	9.666E-15	3
31	USP17	USP17L28	+	71/78	9.666E-15	3
32	RIG-I	DDX58	+	71/78	9.666E-15	2
33	IFN-alpha/beta	IFNAR2	+	68/74	1.072E-14	2
34	IFN-alpha/beta	IFNAR1	+	68/74	1.072E-14	2
35	IFN-alpha		+	73/81	1.489E-14	3
36	IFN-beta	IFNB1	+	73/81	1.489E-14	3
37	IL-18R1	IL18R1	+	70/77	1.762E-14	3
38	IL-1RI	IL1R1	+	70/77	1.762E-14	3
39	miR-345-5p	MIR345	-	104/126	3.036E-14	2
41	IRAK1	IRAK1	+	69/76	3.209E-14	2
40	ISG15	ISG15	+	69/76	3.209E-14	2
44	RIG-G	IFIT3	+	66/72	3.628E-14	2

42	miR-548x-3p	MIR548X2	-	66/72	3.628E-14	2
43	miR-548x-3p	MIR548X	-	66/72	3.628E-14	2
45	CTLA-4	CTLA4	-	109/134	5.267E-14	3
47	IL-35	EBI3	+	65/71	6.661E-14	3
48	IL-35	IL12A	+	65/71	6.661E-14	3
46	miR-935	MIR935	-	65/71	6.661E-14	2
49	SHPS-1	SIRPA	-	143/187	1.022E-13	3
50	IRAKM	IRAK3	-	67/74	1.06E-13	3
52	STAT1	STAT1	+	64/70	1.221E-13	1
51	miR-383-5p	MIR383	-	64/70	1.221E-13	2
53	DOT1	DOT1L	+	103/126	1.393E-13	2
55	IRF9	IRF9	+	69/77	1.569E-13	2
54	UBE1L	UBA7	+	69/77	1.569E-13	3
57	CD69	CD69	+	66/73	1.923E-13	2
56	Polycystin	PKD1	+	66/73	1.923E-13	2
59	MGF	KITLG	+	63/69	2.237E-13	3
61	AGTR1	AGTR1	+	63/69	2.237E-13	2
58	CNOT7	CNOT7	-	63/69	2.237E-13	2
60	FasR(CD95)	FAS	-	63/69	2.237E-13	2
62	CBP/P300	EP300	+	73/83	2.9E-13	2
63	CBP/P300	CREBBP	+	73/83	2.9E-13	2
64	miR-450a-5p	MIR450A2	-	82/96	3.621E-13	2
65	miR-450a-5p	MIR450A1	-	82/96	3.621E-13	2
66	PLC-beta3	PLCB3	-	103/127	3.73E-13	3
67	PTTG3P	PTTG3P	+	88/105	4.976E-13	3
68	BCDIN3D	BCDIN3D	+	67/75	5.049E-13	3
69	SLAP	SLA	-	72/82	5.112E-13	3
70	miR-4674	MIR4674	-	90/108	5.329E-13	3
71	SNX5	SNX5	-	103/128	9.667E-13	3
72	ANKRD17	ANKRD17	+	97/119	9.959E-13	3
73	BAL	PARP9	+	63/70	1.138E-12	2
74	GHR	GHR	+	100/124	1.597E-12	3
75	IL3RA	IL3RA	-	65/73	1.615E-12	3
76	RET	RET	+	83/99	2.195E-12	2
77	IRF1	IRF1	+	105/132	2.328E-12	2
78	Rap1GAP1	RAP1GAP	-	85/102	2.349E-12	3
80	FGFR3	FGFR3	+	78/92	3.081E-12	2
81	IL-6 receptor	IL6R	+	78/92	3.081E-12	2
82	IL-6 receptor	IL6ST	+	78/92	3.081E-12	2
83	gp130	IL6ST	+	78/92	3.081E-12	2
84	IL-22 receptor	IL22RA1	+	78/92	3.081E-12	2
85	IL-22 receptor	IL10RB	+	78/92	3.081E-12	2
79	PTPN2	PTPN2	-	78/92	3.081E-12	2
86	Tyk2	TYK2	+	82/98	3.69E-12	2
87	XAF1	XAF1	+	86/104	4.11E-12	2
88	PPP2R2C	PPP2R2C	-	134/178	4.527E-12	3
89	CNTF	CNTF	+	77/91	5.238E-12	3
90	LIF	LIF	+	77/91	5.238E-12	3

91	KLF5	KLF5	+	121/158	6.494E-12	2
92	c-IAP2	BIRC3	+	76/90	8.889E-12	2
93	EG1	MED28	+	134/179	9.101E-12	3
94	G-protein alpha-s	GNAS	+	127/168	9.79E-12	3
95	alpha-4/beta-1	ITGA4	+	114/148	1.346E-11	3
96	alpha-4/beta-1	ITGB1	+	114/148	1.346E-11	3
97	miR-3941	MIR3941	+	66/76	1.48E-11	3
98	APG12	ATG12	-	110/142	1.615E-11	3
99	STAT2	STAT2	+	97/122	1.662E-11	2
100	PSMC3	PSMC3	+	79/95	1.734E-11	2
101	SGK1	SGK1	+	89/110	1.883E-11	2
102	alpha-6/beta-1	ITGB1	+	72/85	2.314E-11	3
103	alpha-6/beta-1	ITGA6	+	72/85	2.314E-11	3
104	OGG1	OGG1	+	98/124	2.512E-11	2
105	KIAA1914	AFAP1L2	+	128/171	2.65E-11	3
106	FGF21	FGF21	+	80/97	3.004E-11	3
107	miR-483-3p	MIR483	-	82/100	3.074E-11	2
108	JAK2	JAK2	+	84/103	3.104E-11	2
109	MAP2	MAP2	+	124/165	3.401E-11	3
110	NR2A	GRIN2A	+	124/165	3.401E-11	3
111	Actin		+	110/143	3.543E-11	3
112	TNF-alpha	TNF	+	142/194	3.796E-11	3
113	HSPC142	BABAM1	+	57/64	3.819E-11	3
114	PSTPIP2	PSTPIP2	+	64/74	4.479E-11	2
115	IL6RA	IL6R	+	77/93	4.815E-11	3
116	Lnk	SH2B3	-	87/108	4.952E-11	3
117	FISH	SH3PXD2A	+	123/164	5.13E-11	3
118	LRG-47	IRGM	-	100/128	5.476E-11	3
119	miR-502-5p	MIR502	-	142/195	7.058E-11	3
120	GEFT	ARHGEF25	+	103/133	7.26E-11	3
121	PLEKHG4	PLEKHG4	+	103/133	7.26E-11	3
124	DBL	MCF2	+	103/133	7.26E-11	3
122	RhoGAP1	ARHGAP1	-	103/133	7.26E-11	3
123	RhoGDI beta	ARHGDIB	-	103/133	7.26E-11	3
125	MSN (moesin)	MSN	+	90/113	7.442E-11	3
126	alpha-IIb/beta-3	ITGA2B	+	120/160	8.762E-11	3
127	alpha-IIb/beta-3	ITGB3	+	120/160	8.762E-11	3
128	c-Src	SRC	+	123/165	1.02E-10	2
129	AF-10	MLLT10	+	131/178	1.144E-10	3
130	Plexin A2	PLXNA2	+	73/88	1.28E-10	3
131	PDGF-R-beta	PDGFRB	+	75/91	1.326E-10	2
133	ALPHA-PIX	ARHGEF6	+	122/164	1.526E-10	3
132	G-protein alpha-i2	GNAI2	+	122/164	1.526E-10	3
134	Kalirin	KALRN	+	103/134	1.59E-10	3
135	GGTase-I	FNTA	+	103/134	1.59E-10	3
136	GGTase-I	PGGT1B	+	103/134	1.59E-10	3
137	SHP-2	PTPN11	-	90/114	1.793E-10	2
138	Ephrin-B receptor 2	EPHB2	+	68/81	1.904E-10	3

139	Csk	CSK	-	144/200	2.008E-10	3
140	miR-345-5p	MIR345	-	201/295	2.196E-10	3
141	PHAP1 (pp32)	ANP32A	+	78/96	2.223E-10	2
142	miR-23a-3p	MIR23A	-	95/122	2.282E-10	2
143	TFG	TFG	+	171/245	2.579E-10	3
144	IL-11 receptor	IL11RA	+	124/168	2.58E-10	3
145	IL-11 receptor	IL6ST	+	124/168	2.58E-10	3
146	JNK2(MAPK9)	MAPK9	+	112/149	2.897E-10	2
147	miR-496-3p	MIR496	-	117/157	2.974E-10	3
148	MAG	MAG	+	67/80	3.208E-10	3
149	NRB54	NONO	+	81/101	3.465E-10	2
150	Intersectin	ITSN1	-	92/118	3.874E-10	3
151	KLRG1	KLRG1	-	90/115	4.185E-10	3
152	CD63	CD63	-	99/129	4.187E-10	3
153	G-protein alpha-i		+	121/164	4.401E-10	3
154	METTL3	METTL3	+	104/137	4.655E-10	2
155	PD-1	PDCD1	-	140/195	4.904E-10	3
157	p114-RhoGEF	ARHGEF18	+	114/153	5.082E-10	3
156	Ephrin-B2 (CTF2)	EFNB2	+	114/153	5.082E-10	3
158	GM-CSF receptor	CSF2RB	+	82/103	5.348E-10	3
159	GM-CSF receptor	CSF2RA	+	82/103	5.348E-10	3
160	Fyn	FYN	+	66/79	5.391E-10	2
162	Syk	SYK	+	78/97	5.767E-10	2
161	DAP12	TYROBP	+	78/97	5.767E-10	3
163	Tyro3	TYRO3	+	76/94	5.9E-10	3
164	DAB1	DAB1	+	76/94	5.9E-10	3
165	FRS2	FRS2	-	89/114	6.576E-10	3
166	PEAR1	PEAR1	+	87/111	7.09E-10	3
169	PLEKHG2	PLEKHG2	+	96/125	7.146E-10	3
173	DOCK6	DOCK6	+	96/125	7.146E-10	3
176	FARP2	FARP2	+	96/125	7.146E-10	3
168	TNFAIP2	TNFAIP2	+	96/125	7.146E-10	3
172	BIN3	BIN3	+	96/125	7.146E-10	3
167	CDGAP	ARHGAP31	-	96/125	7.146E-10	3
174	p200RhoGAP	ARHGAP32	-	96/125	7.146E-10	3
175	ABR	ABR	-	96/125	7.146E-10	3
170	SH3BP1	SH3BP1	-	96/125	7.146E-10	3
171	Porf-2	ARHGAP39	-	96/125	7.146E-10	3
177	NP14	NOLC1	+	126/173	7.907E-10	3
178	CD151	CD151	+	99/130	8.902E-10	3
179	miR-3607-5p	SNORD138	-	146/206	9.313E-10	3
180	VEGF-A	VEGFA	+	75/93	9.577E-10	3
181	LCoR (MLR2)	LCOR	-	173/251	9.609E-10	3
182	Cardiotrophin-1	CTF1	+	109/146	9.902E-10	3
183	SAD1 (USP39)	USP39	+	95/124	1.102E-09	2
184	Unc-119	UNC119	+	100/132	1.214E-09	3
185	SAP	SH2D1A	+	100/132	1.214E-09	3
186	miR-3188	MIR3188	-	110/148	1.3E-09	3

187	NUR77	NR4A1	-	198/294	1.347E-09	3
188	PREX1	PREX1	+	103/137	1.45E-09	3
189	Rac1	RAC1	+	96/126	1.522E-09	2
190	RhoGDI gamma	ARHGDIG	-	96/126	1.522E-09	3
191	USP1	USP1	+	74/92	1.551E-09	2
192	miR-141-5p	MIR141	-	66/80	1.568E-09	3
193	Calgranulin A	S100A8	+	101/134	1.639E-09	3
194	C/EBPbeta	CEBPB	+	124/171	1.689E-09	2
195	TSLC1	CADM1	+	99/131	1.848E-09	3
196	Cathepsin G	CTSG	-	83/106	1.894E-09	3
197	G-protein alpha-i1	GNAI1	-	83/106	1.894E-09	3
198	LARP1	LARP1	+	104/139	1.928E-09	3
199	Calgranulin B	S100A9	+	181/266	1.956E-09	3
200	FBXL7	FBXL7	-	102/136	2.19E-09	3
201	Rab-1A	RAB1A	+	107/144	2.222E-09	3
202	PTPR-zeta	PTPRZ1	+	157/226	2.307E-09	3
203	MFHAS1	MFHAS1	+	75/94	2.409E-09	3
204	Biglycan	BGN	+	75/94	2.409E-09	3
205	Glycoprotein VI	GP6	+	100/133	2.481E-09	3
206	Thy-1	THY1	+	100/133	2.481E-09	3
207	PP2A cat (alpha)	PPP2CA	-	67/82	2.629E-09	2
208	UBE3C	UBE3C	-	132/185	2.892E-09	3
209	p38beta (MAPK11)	MAPK11	+	103/138	2.898E-09	2
210	miR-146a-5p	MIR146A	-	91/119	2.901E-09	2
211	CNK1	CNKSR1	+	141/200	3.154E-09	3
212	DOCK3	DOCK3	+	96/127	3.164E-09	3
213	miR-146b-5p	MIR146B	-	80/102	3.212E-09	2
214	mature miR-4429	MIR4429	-	119/164	3.339E-09	3
215	SMURF1	SMURF1	-	78/99	3.441E-09	2
216	Dopamine D1A	DRD1	-	87/113	3.559E-09	3
217	TRIM26	TRIM26	-	136/192	3.636E-09	3
218	mTOR	MTOR	+	85/110	3.916E-09	2
219	ErbB4	ERBB4	+	70/87	4.175E-09	2
220	APPL2	APPL2	+	97/129	4.249E-09	3
221	SYNE2	SYNE2	-	97/129	4.249E-09	3
222	RagB	RRAGB	+	90/118	4.457E-09	3
223	RagD	RRAGD	+	90/118	4.457E-09	3
224	SH3RF	SH3RF1	+	95/126	4.805E-09	3
225	DDR1	DDR1	-	118/163	4.868E-09	3
226	Fc gamma RI	FCGR1A	+	100/134	4.96E-09	3
227	Fc gamma RI	FCGR1A	+	100/134	4.96E-09	3
228	CD79 complex	CD79A	+	100/134	4.96E-09	3
229	CD79 complex	CD79B	+	100/134	4.96E-09	3
230	IKK-alpha	CHUK	+	141/201	5.345E-09	2
231	C6orf176	LINC00473	+	144/206	5.382E-09	3
232	PDGF-B	PDGFB	+	108/147	5.524E-09	3
234	Migfilin	FBLIM1	+	116/160	5.672E-09	3
233	miR-412-5p	MIR412	-	116/160	5.672E-09	3

235	ADAM17	ADAM17	+	103/139	5.673E-09	3
236	c-Fos	FOS	+	176/260	5.92E-09	3
238	miR-99a-3p	MIR99A	-	84/109	6.08E-09	3
237	hPEM-2 (collybistin)	ARHGEF9	-	84/109	6.08E-09	3
239	eIF3S3	EIF3H	+	145/208	6.384E-09	3
240	NMI	NMI	+	71/89	6.484E-09	2
241	ActRIIA	ACVR2A	+	69/86	6.748E-09	3
242	PTAFR	PTAFR	+	89/117	6.831E-09	3
243	PLC-beta2	PLCB2	+	94/125	7.28E-09	3
244	miR-4698	MIR4698	-	134/190	7.352E-09	3
245	Pacsin 2	PACSIN2	-	99/133	7.439E-09	3
246	BLNK	BLNK	+	50/58	7.851E-09	3
247	G-protein		+	92/122	8.234E-09	3
248	Ragulator	LAMTOR2	+	85/111	8.493E-09	3
249	Ragulator	LAMTOR5	+	85/111	8.493E-09	3
250	Ragulator	LAMTOR4	+	85/111	8.493E-09	3
251	Ragulator	LAMTOR1	+	85/111	8.493E-09	3
252	Ragulator	LAMTOR3	+	85/111	8.493E-09	3
253	Galectin-1	LGALS1	+	97/130	8.494E-09	3
254	MATK	MATK	-	141/202	8.954E-09	3
255	Cyclin C	CCNC	+	163/239	9.336E-09	3
256	APG5	ATG5	-	119/166	1.085E-08	3
257	RHEB2	RHEB	+	93/124	1.1E-08	3
258	NF-AT4(NFATC3)	NFATC3	+	111/153	1.123E-08	3
259	Calmyrin	CIB1	+	122/171	1.134E-08	3
260	BRMS1	BRMS1	-	86/113	1.17E-08	2
261	COMMD1 (MURR1)	COMMD1	-	175/260	1.245E-08	3
262	DEPTOR	DEPTOR	-	84/110	1.307E-08	3
263	RIG-G	IFIT3	+	112/155	1.403E-08	3
264	miR-1225-5p	MIR1225	-	112/155	1.403E-08	3
265	IRS-2	IRS2	-	112/155	1.403E-08	3
266	MECOM	MECOM	-	94/126	1.455E-08	2
267	STAT2	STAT2	+	132/188	1.474E-08	3
268	MEF2D	MEF2D	+	180/269	1.521E-08	3
269	TSLPR	CRLF2	+	87/115	1.592E-08	3
270	TSLPR	IL7R	+	87/115	1.592E-08	3
271	KLF5	KLF5	+	184/276	1.624E-08	3
272	miR-539-3p	MIR539	-	121/170	1.627E-08	3
273	c-Fos	FOS	+	97/131	1.661E-08	2
274	miR-548x-3p	MIR548X2	-	105/144	1.766E-08	3
275	miR-548x-3p	MIR548X	-	105/144	1.766E-08	3
276	Syndecan-3	SDC3	+	116/162	1.835E-08	3
278	IL4RA	IL4R	+	76/98	1.948E-08	3
277	CD3 zeta	CD247	+	76/98	1.948E-08	3
279	VAV-3	VAV3	+	128/182	2.06E-08	3
280	GFRalpha1	GFRA1	+	137/197	2.086E-08	3
281	PHAP1 (pp32)	ANP32A	+	134/192	2.09E-08	3
282	PPP2R2B	PPP2R2B	-	88/117	2.14E-08	3

283	miR-548ah-5p	MIR548AH	-	98/133	2.146E-08	3
284	QIK	SIK2	-	156/229	2.158E-08	3
285	JAK1	JAK1	+	81/106	2.24E-08	2
286	LAGY	HOPX	+	117/164	2.248E-08	3
287	miR-330-5p	MIR330	-	101/138	2.375E-08	2
288	TGM4	TGM4	+	126/179	2.434E-08	3
289	RNF6	RNF6	+	154/226	2.569E-08	3
290	SHP-1	PTPN6	-	148/216	2.723E-08	3
291	MafK	MAFK	+	185/279	2.739E-08	3
292	TPL2(MAP3K8)	MAP3K8	+	77/100	2.757E-08	2
293	BAP1	BAP1	+	130/186	2.931E-08	3
294	TBK1	TBK1	+	75/97	3.037E-08	2
295	LINC00963	LINC00963	+	82/108	3.071E-08	3
296	CPEB3	CPEB3	-	113/158	3.102E-08	3
297	Connexin 43	GJA1	-	159/235	3.26E-08	3
298	PIAS4	PIAS4	-	176/264	3.273E-08	3
299	P52rIPK	THAP12	+	80/105	3.44E-08	3
300	PLD2	PLD2	+	100/137	3.495E-08	3
301	NR1	GRIN1	+	147/215	3.751E-08	3
302	HNF1-alpha	HNF1A	+	114/160	3.794E-08	3
303	NET1	NET1	+	117/165	3.917E-08	3
304	PIAS4	PIAS4	-	141/205	3.94E-08	2
305	Fc epsilon RI	FCER1A	+	98/134	4.055E-08	3
306	Fc epsilon RI	MS4A2	+	98/134	4.055E-08	3
307	Fc epsilon RI	FCER1G	+	98/134	4.055E-08	3
308	PAK1	PAK1	+	76/99	4.267E-08	2
309	MOZ	KAT6A	+	101/139	4.409E-08	2
310	DOCK1	DOCK1	+	91/123	4.869E-08	3
311	MIR205HG	MIR205HG	-	130/187	4.87E-08	3
312	TLR6	TLR6	+	37/41	5.129E-08	3
313	miR-30b-5p	MIR30B	-	99/136	5.13E-08	2
314	IFNAR2	IFNAR2	+	184/279	5.418E-08	3
315	STRA6	STRA6	+	94/128	5.436E-08	3
316	miR-129-1-3p	MIR129-1	-	137/199	5.569E-08	3
317	LINC02575	LINC02575	+	134/194	5.665E-08	3
318	CMIP	CMIP	-	119/169	5.693E-08	3
319	DOCK2	DOCK2	+	92/125	6.28E-08	3
320	HSP70L1	HSPA14	+	82/109	6.304E-08	3
321	CENTA2	ADAP2	+	100/138	6.442E-08	3
322	miR-23a-3p	MIR23A	-	129/186	6.809E-08	3
323	IL-3 receptor	CSF2RB	+	106/148	7.231E-08	3
324	IL-3 receptor	IL3RA	+	106/148	7.231E-08	3
325	RagC	RRAGC	+	85/114	7.338E-08	3
326	ABL2	ABL2	+	162/242	7.373E-08	3
327	c-Src	SRC	+	152/225	7.504E-08	3
328	ZPR9	ZNF622	+	133/193	7.87E-08	3
329	Nucleophosmin	NPM1	+	133/193	7.87E-08	2
330	LIFR	LIFR	+	93/127	8.028E-08	3

331	NCAM1	NCAM1	+	127/183	8.083E-08	3
332	ATP7A	ATP7A	+	71/92	8.084E-08	3
334	STAT4	STAT4	+	104/145	8.484E-08	3
333	miR-518d-5p	MIR518D	-	104/145	8.484E-08	3
335	miR-224-3p	MIR224	-	91/124	9.296E-08	3
336	Caspase-12	CASP12	-	113/160	9.385E-08	3
337	CNR1	CNR1	+	67/86	9.705E-08	3
338	Karyopherin alpha 1	KPNA1	+	79/105	1.083E-07	2
339	NANOS2	NANOS2	-	72/94	1.13E-07	3
340	IFNAR2	IFNAR2	+	33/36	1.136E-07	2
341	FOXP3	FOXP3	-	100/139	1.166E-07	3
342	c-Jun	JUN	+	103/144	1.224E-07	2
343	DTX4	DTX4	-	77/102	1.226E-07	3
344	CBP	CREBBP	+	181/276	1.257E-07	2
345	IL-9 receptor	IL2RG	+	82/110	1.26E-07	3
346	IL-9 receptor	IL9R	+	82/110	1.26E-07	3
347	IL-2R gamma chain	IL2RG	+	82/110	1.26E-07	3
348	Tyk2	TYK2	+	146/216	1.27E-07	3
349	USP13	USP13	+	95/131	1.28E-07	2
350	Fbxl19	FBXL19	-	185/283	1.293E-07	3
351	Contactin 2	CNTN2	+	112/159	1.333E-07	3
352	Karyopherin alpha 3	KPNA3	+	150/223	1.382E-07	3
353	DPF3	DPF3	+	68/88	1.385E-07	2
354	OSM receptor	IL6ST	+	85/115	1.43E-07	3
355	OSM receptor	OSMR	+	85/115	1.43E-07	3
356	CAS-L	NEDD9	+	194/299	1.483E-07	3
357	NUR77	NR4A1	-	110/156	1.576E-07	2
358	miR-101-3p	MIR101-2	-	116/166	1.605E-07	2
359	miR-101-3p	MIR101-1	-	116/166	1.605E-07	2
360	ATF-5	ATF5	+	195/301	1.627E-07	3
361	LYRIC	MTDH	+	99/138	1.687E-07	2
362	miR-520b-3p	MIR520B	-	71/93	1.741E-07	2
363	DTX1	DTX1	-	192/296	1.763E-07	3
364	WDR48	WDR48	+	159/239	1.789E-07	3
365	RAI	RNH1	-	129/188	1.796E-07	3
366	alpha-6/beta-4	ITGA6	+	105/148	1.82E-07	3
367	alpha-6/beta-4	ITGB4	+	105/148	1.82E-07	3
368	p90Rsk		+	123/178	1.868E-07	3
369	miR-33a-3p	MIR33A	-	174/265	1.915E-07	3
370	CIA/ASF1	ASF1B	+	182/279	2.024E-07	3
371	CC2D1A	CC2D1A	+	171/260	2.054E-07	3
372	NP220	ZNF638	+	161/243	2.249E-07	3
373	miR-548a-3p	MIR548A1	-	95/132	2.319E-07	3
374	miR-548a-3p	MIR548A3	-	95/132	2.319E-07	3
375	miR-548a-3p	MIR548A2	-	95/132	2.319E-07	3
377	FOXP3	FOXP3	-	65/84	2.374E-07	2
376	hsa-miR-4756-5p	MIR4756	-	65/84	2.374E-07	3
379	miR-128-1-5p	MIR128-1	-	131/192	2.406E-07	3

378	Homer 3	HOMER3	-	131/192	2.406E-07	3
380	ASF1A	ASF1A	+	180/276	2.409E-07	3
381	Fc epsilon RI beta	MS4A2	+	98/137	2.435E-07	3
382	LARS1	LARS1	+	101/142	2.528E-07	3
383	IRF1	IRF1	+	208/325	2.533E-07	3
384	miR-365b-3p	MIR365B	-	116/167	2.666E-07	3
385	JSAP1	MAPK8IP3	+	110/157	2.671E-07	3
386	CAP1	CAP1	+	135/199	2.671E-07	3
387	NIK(MAP3K14)	MAP3K14	+	70/92	2.674E-07	2
388	ARHGAP21	ARHGAP21	-	113/162	2.678E-07	3
389	p300	EP300	+	193/299	2.758E-07	2
390	DEP-1	PTPRJ	+	99/139	2.978E-07	3
392	MUC1-CT	MUC1	+	68/89	2.993E-07	2
394	IFRD1	IFRD1	+	68/89	2.993E-07	2
391	CLIM1	PDLIM1	-	68/89	2.993E-07	2
393	FAF1	FAF1	-	68/89	2.993E-07	2
395	c-IAP2	BIRC3	+	198/308	3.021E-07	3
396	IL-7 receptor	IL2RG	+	102/144	3.066E-07	3
397	IL-7 receptor	IL7R	+	102/144	3.066E-07	3
398	HTR2A	HTR2A	+	105/149	3.128E-07	3
399	PTMA	PTMA	+	179/275	3.161E-07	3
400	HOXB-AS1	HOXB-AS1	+	108/154	3.164E-07	3
401	AUP1	AUP1	+	73/97	3.202E-07	3
402	TRIP6	TRIP6	+	66/86	3.335E-07	2
403	SMC1	SMC1A	+	94/131	3.359E-07	3
404	GFI-1	GFI1	-	94/131	3.359E-07	2
405	miR-3173-3p	MIR3173	-	127/186	3.415E-07	3
406	BAF170	SMARCC2	+	151/227	3.618E-07	3
407	PDLIM2	PDLIM2	-	51/63	3.73E-07	2
408	KIF26A	KIF26A	-	112/161	3.759E-07	3
409	Neurogenin 3	NEUROG3	+	193/300	3.908E-07	3
410	FKBP4	FKBP4	+	84/115	4.014E-07	2
411	FMIP	THOC5	+	95/133	4.125E-07	3
412	MEGF10	MEGF10	+	74/99	4.253E-07	3
413	D52	TPD52	+	156/236	4.262E-07	3
414	TBP	TBP	+	116/168	4.37E-07	3
415	FAK1	PTK2	+	113/163	4.429E-07	2
416	miR-378g	MIR378G	+	104/148	4.437E-07	3
417	TRIO	TRIO	+	129/190	4.525E-07	3
418	BDKRB2	BDKRB2	+	67/88	4.602E-07	3
419	cPKC (conventional)		+	126/185	4.692E-07	3
420	NIX	BNIP3L	-	93/130	4.851E-07	3
421	EAP30	SNF8	+	161/245	4.938E-07	3
422	LOC101929517	ETS1-AS1	+	114/165	5.197E-07	3
423	Keratin 18	KRT18	-	111/160	5.263E-07	3
424	TRIM72	TRIM72	-	105/150	5.294E-07	3
425	C/EBPbeta	CEBPB	+	177/273	5.411E-07	3
426	p38alpha (MAPK14)	MAPK14	+	144/216	5.46E-07	2

427	Esrra	ESRRA	-	75/101	5.577E-07	3
428	KLF6	KLF6	+	63/82	5.734E-07	2
429	RhoB	RHOB	-	83/114	5.902E-07	3
430	cKrox	ZBTB7B	+	94/132	5.931E-07	3
431	HES6	HES6	+	178/275	5.935E-07	3
432	Septin 9	SEPTIN9	+	163/249	6.07E-07	3
433	JunD	JUND	+	97/137	6.101E-07	3
434	miR-551a	MIR551A	-	112/162	6.18E-07	3
435	PSMD10 (Gankyrin)	PSMD10	-	100/142	6.215E-07	2
436	DLEU1	DLEU1	+	109/157	6.253E-07	3
437	NAP2	NAP1L4	+	156/237	6.313E-07	3
438	IL-2 receptor	IL2RB	+	86/119	6.334E-07	3
439	IL-2 receptor	IL2RA	+	86/119	6.334E-07	3
440	IL-2 receptor	IL2RG	+	86/119	6.334E-07	3
442	PRDX1	PRDX1	+	73/98	6.398E-07	2
441	D52	TPD52	+	73/98	6.398E-07	2
443	DET1	DET1	-	135/201	6.426E-07	3
444	A2M	A2M	+	81/111	6.869E-07	3
445	ErbB4	ERBB4	+	139/208	6.895E-07	3
446	BAT3	BAG6	+	196/307	7.051E-07	3
447	MEF2A	MEF2A	+	192/300	7.103E-07	3
448	CASC9	CASC9	+	188/293	7.132E-07	3
449	MSK1	RPS6KA5	+	95/134	7.204E-07	2
452	miR-1298-5p	MIR1298	-	113/164	7.228E-07	3
450	PPP4C	PPP4C	-	113/164	7.228E-07	3
451	SIN1	MAPKAP1	-	113/164	7.228E-07	3
454	miR-448-3p	MIR448	-	150/227	7.229E-07	3
453	DDX19L	DDX19A	-	76/103	7.229E-07	3
455	SUR-8	SHOC2	-	101/144	7.437E-07	3
456	CDK5RAP3	CDK5RAP3	-	147/222	7.716E-07	3
457	ART-27	UXT	+	151/229	8.062E-07	3
458	LGP2	DHX58	-	74/100	8.337E-07	3
459	PI3K cat class III	PIK3C3	+	69/92	8.352E-07	3
460	miR-1323	MIR1323	-	127/188	8.43E-07	3
461	LINC02605	LINC02605	+	203/320	8.806E-07	3
462	PRDX1	PRDX1	+	163/250	8.827E-07	3
463	MafK	MAFK	+	62/81	8.865E-07	2
464	c-Rel (NF-kB	REL	+	39/46	9.158E-07	2
465	TERT	TERT	+	85/118	9.215E-07	2
466	cKrox	ZBTB7B	+	60/78	9.865E-07	2
467	PSMD10 (Gankyrin)	PSMD10	-	168/259	9.882E-07	3
468	IEX1	IER3	-	112/163	1.003E-06	3
469	BRG1	SMARCA4	+	146/221	1.029E-06	2
471	Arrestin 3	ARR3	+	103/148	1.048E-06	3
470	MKP-2	DUSP4	-	103/148	1.048E-06	3
472	LOC145837	DRAIC	-	161/247	1.051E-06	3
473	miR-520c-3p	MIR520C	-	75/102	1.074E-06	2
474	CCR2	CCR2	+	143/216	1.098E-06	3

475	SIX1	SIX1	+	162/249	1.158E-06	3
476	IL-4R type I	IL4R	+	113/165	1.164E-06	3
477	IL-4R type I	IL2RG	+	113/165	1.164E-06	3
478	Dystrophin	DMD	+	113/165	1.164E-06	3
479	PRK1	PKN1	+	140/211	0.00000117	3
480	OGG1	OGG1	+	186/291	1.187E-06	3
481	alpha-V/beta-1	ITGAV	+	110/160	1.194E-06	3
482	alpha-V/beta-1	ITGB1	+	110/160	1.194E-06	3
483	CPEB4	CPEB4	+	123/182	0.0000012	3
484	IL-2R beta chain	IL2RB	+	107/155	1.218E-06	3
485	Ephrin-A receptor 4	EPHA4	+	141/213	0.00000131	3
486	M-CSF receptor	CSF1R	+	152/232	1.318E-06	3
487	PPP4R1	PPP4R1	-	134/201	1.321E-06	3
488	miR-138-2-3p	MIR138-2	-	84/117	1.337E-06	3
489	miR-4319	MIR4319	+	76/104	1.368E-06	3
490	EPC1	EPC1	+	180/281	1.418E-06	3
491	Fra-1	FOSL1	+	66/88	0.00000144	3
492	RelA (p65 NF-kB	RELA	+	93/132	1.464E-06	2
493	Dexras1	RASD1	-	96/137	1.477E-06	3
494	STAT2	STAT2	+	23/24	0.00000149	1
495	PAK2	PAK2	+	189/297	1.509E-06	3
496	SLM-2	KHDRBS3	+	115/169	1.552E-06	3
497	TH1L	NELFCD	-	139/210	1.564E-06	3
498	FasL(TNFSF6)	FASLG	-	82/114	1.569E-06	3
499	KIS	UHMK1	+	136/205	1.667E-06	3
500	COMMD1 (MURR1)	COMMD1	-	77/106	1.726E-06	2
501	MAP3K2 (MEKK2)	MAP3K2	+	151/231	1.739E-06	3
502	TIRAP (Mal)	TIRAP	+	94/134	1.752E-06	3
503	MKK7 (MAP2K7)	MAP2K7	+	159/245	1.807E-06	3
504	miR-302d-5p	MIR302D	-	72/98	1.844E-06	3
505	I-kB		-	67/90	1.898E-06	2
506	p38gamma	MAPK12	+	188/296	1.933E-06	3
507	Nova1	NOVA1	+	86/121	2.002E-06	3
508	IKBZ	NFKBIZ	-	75/103	2.014E-06	2
509	Agrin	AGRN	+	117/173	0.00000204	3
510	FBXL15	FBXL15	+	89/126	0.00000205	3
511	DDX1	DDX1	+	95/136	2.085E-06	3
512	MECOM	MECOM	-	198/314	2.155E-06	3
513	CaMK II gamma	CAMK2G	+	142/216	2.165E-06	3
514	PSMC3	PSMC3	+	146/223	2.233E-06	3
515	AZI2	AZI2	+	73/100	2.346E-06	3
516	RARbeta	RARB	-	105/153	2.348E-06	3
517	Paxillin	PXN	+	166/258	2.387E-06	3
518	ACKR3	ACKR3	+	143/218	2.404E-06	3
519	SH2B	SH2B1	+	143/218	2.404E-06	3
520	CUX1	CUX1	-	68/92	2.468E-06	2
521	IRAK1BP1	IRAK1BP1	+	63/84	2.484E-06	2
522	Pyrin (MEFV)	MEFV	+	119/177	2.647E-06	3

523	COASY	COASY	-	148/227	2.727E-06	3
524	NPAT	NPAT	+	71/97	2.729E-06	3
525	DPF2	DPF2	+	197/313	2.736E-06	3
526	SFRS3	SRSF3	+	116/172	2.779E-06	3
527	UBF	UBTF	+	61/81	2.828E-06	3
530	SOX11	SOX11	+	61/81	2.828E-06	2
528	TESC	TESC	+	61/81	2.828E-06	2
531	CRSP8 (CRSP34)	MED27	+	61/81	2.828E-06	2
532	ZFP91	ZFP91	+	61/81	2.828E-06	2
529	UACA	UACA	-	61/81	2.828E-06	2
533	AF-6	AFDN	-	66/89	2.845E-06	3
534	SNFT	BATF3	-	100/145	2.862E-06	3
536	USP44	USP44	-	91/130	2.938E-06	3
535	FMNL1	FMNL1	-	91/130	2.938E-06	3
537	NFKBIE	NFKBIE	-	145/222	2.945E-06	3
538	TIM-3	HAVCR2	+	110/162	3.029E-06	3
539	BAF60c	SMARCD3	+	153/236	0.00000305	3
540	DOT1	DOT1L	+	173/271	3.056E-06	3
541	USP1	USP1	+	169/264	0.00000308	3
542	MIR31HG	MIR31HG	+	77/107	3.139E-06	2
543	LPAR2	LPAR2	+	107/157	3.143E-06	3
544	GATA-2	GATA2	-	131/198	3.173E-06	3
545	MKP-5	DUSP10	-	142/217	3.176E-06	3
547	RelA (p65 NF-kB	RELA	+	59/78	3.208E-06	1
546	WIP1	PPM1D	-	59/78	3.208E-06	2
548	LTBR(TNFRSF3)	LTBR	+	52/67	0.00000323	3
549	ERK1/2		+	195/310	3.233E-06	3
550	ABIN-2	TNIP2	+	150/231	3.307E-06	3
551	CPSF4	CPSF4	+	92/132	3.487E-06	3
552	FasR(CD95)	FAS	-	132/200	3.543E-06	3
553	NLRC5	NLRC5	-	125/188	3.604E-06	3
554	miR-542-3p	MIR542	-	136/207	3.677E-06	3
555	TIP41	TIPRL	+	75/104	3.683E-06	3
556	PLC-gamma 1	PLCG1	+	115/171	3.776E-06	3
557	APP	APP	+	129/195	3.789E-06	3
558	SAM68	KHDRBS1	+	102/149	3.872E-06	2
559	p16INK4	CDKN2A	-	99/144	3.976E-06	2
560	CaMK II gamma	CAMK2G	+	81/114	4.002E-06	2
561	miR-297	MIR297	-	156/242	4.006E-06	3
562	G-protein alpha-12	GNA12	+	84/119	4.091E-06	3
563	ITGB3	ITGB3	+	141/216	4.186E-06	3
564	UACA	UACA	-	130/197	0.00000423	3
566	NFAT-90	ILF3	+	65/88	4.251E-06	2
565	NFKBIB	NFKBIB	-	65/88	4.251E-06	2
569	TBP	TBP	+	60/80	0.00000429	2
571	JunD	JUND	+	60/80	0.00000429	2
567	DDX9	DHX9	+	60/80	0.00000429	2
568	CPSF4	CPSF4	+	60/80	0.00000429	2

570	Clusterin	CLU	-	60/80	0.00000429	2
572	Makorin-2	MKRN2	-	186/295	0.00000432	3
573	NR2E3	NR2E3	+	157/244	4.373E-06	3
574	LINC02605	LINC02605	+	42/52	4.532E-06	2
575	TLR2	TLR2	+	120/180	4.572E-06	3
576	SMAD1	SMAD1	+	174/274	4.593E-06	3
577	Cyclin T1	CCNT1	+	131/199	4.711E-06	3
578	Nucleophosmin	NPM1	+	166/260	4.714E-06	3
579	MSK1	RPS6KA5	+	183/290	4.755E-06	3
580	ITGB8	ITGB8	+	124/187	4.828E-06	3
582	HES1	HES1	+	117/175	4.844E-06	3
581	DIS3L2	DIS3L2	+	117/175	4.844E-06	3
583	POT1	POT1	+	85/121	4.907E-06	3
584	TIE2	TEK	-	88/126	4.924E-06	3
585	miR-27a-5p	MIR27A	-	147/227	0.00000515	3
586	c-Jun	JUN	+	151/234	5.188E-06	3
587	BTG3	BTG3	-	159/248	5.191E-06	3
588	p38beta (MAPK11)	MAPK11	+	176/278	5.345E-06	3
589	alpha-3/beta-1	ITGB1	+	125/189	5.402E-06	3
590	alpha-3/beta-1	ITGA3	+	125/189	5.402E-06	3
591	alpha-8/beta-1	ITGA8	+	125/189	5.402E-06	3
592	alpha-8/beta-1	ITGB1	+	125/189	5.402E-06	3
593	alpha-10/beta-1	ITGB1	+	125/189	5.402E-06	3
594	alpha-10/beta-1	ITGA10	+	125/189	5.402E-06	3
595	alpha-11/beta-1	ITGA11	+	125/189	5.402E-06	3
596	alpha-11/beta-1	ITGB1	+	125/189	5.402E-06	3
597	BTEB1	KLF9	-	118/177	5.462E-06	3
599	PHF20	PHF20	+	61/82	5.648E-06	2
598	miR-520e-3p	MIR520E	-	61/82	5.648E-06	2
601	ERK1/2		+	156/243	5.668E-06	2
600	miR-646	MIR646	-	156/243	5.668E-06	3
602	Syndecan-1	SDC1	+	92/133	5.758E-06	3
603	G-protein beta-1	GNB1	+	89/128	5.827E-06	3
604	DCTN2	DCTN2	-	86/123	0.00000585	2
605	BRD4/NUT fusion		+	182/289	6.043E-06	3
606	USP12	USP12	+	165/259	6.062E-06	3
607	microRNA 214	MIR214	-	109/162	6.437E-06	3
608	ART-27	UXT	+	75/105	6.577E-06	2
609	PHF20	PHF20	+	127/193	6.714E-06	3
610	LOC102724571	LNCTAM34A	+	154/240	6.739E-06	3
611	SP1	SP1	+	211/341	6.762E-06	3
612	TFIIB	GTF2B	+	139/214	7.223E-06	3
613	PTPR-epsilon	PTPRE	-	155/242	7.331E-06	3
614	LINC00470	LINC00470	+	128/195	7.459E-06	3
615	IRF9	IRF9	+	97/142	0.00000761	3
616	TRIF (TICAM1)	TICAM1	+	26/29	7.618E-06	2
617	IL-23 receptor	IL23R	+	132/202	7.666E-06	3
618	IL-23 receptor	IL12RB1	+	132/202	7.666E-06	3

619	mTOR	MTOR	+	181/288	7.666E-06	3
620	JunB	JUNB	+	177/281	7.875E-06	3
621	IFN-alpha/beta	IFNAR2	+	140/216	7.935E-06	3
622	IFN-alpha/beta	IFNAR1	+	140/216	7.935E-06	3
623	PIAS1	PIAS1	-	140/216	7.935E-06	2
624	FBXO24	FBXO24	-	91/132	8.029E-06	3
625	Alpha-catenin		+	85/122	8.245E-06	3
626	miR-302a-3p	MIR302A	-	165/260	8.403E-06	3
627	TPL2(MAP3K8)	MAP3K8	+	178/283	8.451E-06	3
628	Adenosine A2b	ADORA2B	-	55/73	8.457E-06	2
629	miR-221-5p	MIR221	+	133/204	8.465E-06	3
630	IKK-alpha	CHUK	+	187/299	8.515E-06	3
631	SHP-2	PTPN11	-	137/211	8.608E-06	3
632	CDK9	CDK9	+	68/94	8.658E-06	2
633	p38alpha (MAPK14)	MAPK14	+	174/276	8.675E-06	3
634	miR-485-3p	MIR485	-	145/225	8.747E-06	3
635	CIZ1	CIZ1	+	115/173	8.769E-06	3
636	NKRF	NKRF	-	126/192	8.904E-06	3
637	ELKS	ERC1	+	134/206	9.329E-06	3
638	CRIP2	CRIP2	-	92/134	9.353E-06	3
639	SRF	SRF	+	146/227	9.548E-06	3
640	Dcc	DCC	+	123/187	9.571E-06	3
641	p15	CDKN2B	-	102/151	9.644E-06	3
642	p18	CDKN2C	-	102/151	9.644E-06	3
643	EAP30	SNF8	+	28/32	9.651E-06	2
644	PPAR-alpha	PPARA	-	77/109	0.00000969	2
645	NBAT1	NBAT1	+	127/194	9.868E-06	3
646	MCT-1	MCTS1	+	127/194	9.868E-06	3
647	miR-146b-5p	MIR146B	-	120/182	0.00001027	3
648	TIM-4	TIMD4	+	106/158	0.00001043	3
650	Elongin C	ELOC	-	96/141	0.00001048	3
649	SCCA-1	SERPINB3	-	113/170	0.00001048	3
651	miR-210-5p	MIR210	+	93/136	0.00001084	3
652	Par-4	PAWR	-	61/83	0.0000109	2
653	RBM8 (Y14)	RBM8A	+	156/245	0.00001111	3
654	miR-105-5p	MIR105-1	-	156/245	0.00001111	3
655	miR-105-5p	MIR105-2	-	156/245	0.00001111	3
656	TRAF7	TRAF7	-	90/131	0.00001116	2
657	SH3BP-2	SH3BP2	-	90/131	0.00001116	3
658	IRX3	IRX3	-	72/101	0.00001118	3
659	ANGPTL4	ANGPTL4	+	136/210	0.00001127	3
660	NFKBIA	NFKBIA	-	148/231	0.00001132	3
661	SGK1	SGK1	+	140/217	0.00001134	3
662	PTPR-alpha	PTPRA	+	84/121	0.00001158	3
664	SNHG6	SNHG6	+	161/254	0.00001181	3
663	PDCD10	PDCD10	-	107/160	0.00001181	3
666	PRMT6	PRMT6	+	64/88	0.00001186	2
665	MAD2L1BP	MAD2L1BP	+	64/88	0.00001186	3

667	NFKBIE	NFKBIE	-	64/88	0.00001186	2
668	LRIG2	LRIG2	+	129/198	0.00001204	3
669	Betacellulin	BTC	+	133/205	0.00001223	3
672	Epiregulin	EREG	+	133/205	0.00001223	3
670	nAChR alpha-7	CHRFAM7A	+	133/205	0.00001223	3
671	nAChR alpha-7	CHRNA7	+	133/205	0.00001223	3
673	Granzyme M	GZMM	-	145/226	0.00001238	3
674	TRIM40	TRIM40	-	145/226	0.00001238	3
675	ATF-5	ATF5	+	38/47	0.00001245	2
676	CRK	CRK	+	111/167	0.00001253	3
677	IKK (cat)	IKBKB	+	54/72	0.00001284	2
678	IKK (cat)	CHUK	+	54/72	0.00001284	2
680	alpha-5/beta-1	ITGB1	+	126/193	0.00001302	3
681	alpha-5/beta-1	ITGA5	+	126/193	0.00001302	3
679	MIB1	MIB1	+	126/193	0.00001302	3
682	G-protein alpha-13	GNA13	+	88/128	0.00001331	3
683	LINC00277	EWSAT1	+	108/162	0.00001332	3
684	NF-kB1 (p105)	NFKB1	-	146/228	0.00001346	3
685	DPF3	DPF3	+	142/221	0.00001352	3
686	Calcyclin	S100A6	-	85/123	0.0000136	3
687	Alpha crystallin B	CRYAB	-	85/123	0.0000136	2
688	DEAF	DEAF1	+	25/28	0.00001372	2
689	Menin	MEN1	-	76/108	0.0000138	2
690	OASL	OASL	+	62/85	0.00001386	3
691	miR-194-5p	MIR194-1	-	79/113	0.00001387	2
692	miR-194-5p	MIR194-2	-	79/113	0.00001387	2
694	JNK1(MAPK8)	MAPK8	+	112/169	0.00001404	2
693	Gemin5	GEMIN5	+	112/169	0.00001404	3
695	NOTCH2 (2ICD)	NOTCH2	+	105/157	0.00001412	3
696	DUSP13	DUSP13	-	127/195	0.00001437	3
697	Karyopherin alpha 2	KPNA2	+	92/135	0.00001495	2
698	PAK		+	109/164	0.00001497	3
699	IRF9	IRF9	+	16/16	0.00001526	1
700	SNX8	SNX8	+	106/159	0.00001592	3
702	Neuregulin 2	NRG2	+	132/204	0.000016	3
701	PDIA3	PDIA3	+	132/204	0.000016	3
703	hnRNP F	HNRNPF	+	144/225	0.00001601	3
704	LIG-1	LRIG1	-	136/211	0.00001608	3
705	RelB (NF-kB	RELB	+	60/82	0.00001616	2
707	CaMK IV	CAMK4	+	74/105	0.00001638	2
706	ANAPC4	ANAPC4	+	74/105	0.00001638	3
708	FGF9	FGF9	+	80/115	0.00001642	3
710	FGF2	FGF2	+	80/115	0.00001642	3
711	FGF8	FGF8	+	80/115	0.00001642	3
709	Klotho beta	KLB	+	80/115	0.00001642	3
712	ZAK	MAP3K20	+	96/142	0.00001647	3
713	Ankyrin-G	ANK3	-	96/142	0.00001647	3
714	GSTP1	GSTP1	-	157/248	0.0000166	3

715	miR-653-5p	MIR653	-	27/31	0.00001698	2
716	miR-130b-3p	MIR130B	-	125/192	0.00001714	2
717	IL-10 receptor	IL10RB	+	129/199	0.00001738	3
718	IL-10 receptor	IL10RA	+	129/199	0.00001738	3
719	FAM120A	FAM120A	+	154/243	0.00001826	3
721	ECT2	ECT2	+	111/168	0.00001877	3
720	PL scramblase 1	PLSCR1	-	111/168	0.00001877	3
722	miR-409-5p	MIR409	+	78/112	0.00001955	3
723	FGF17	FGF17	+	75/107	0.00001959	3
724	JNK2(MAPK9)	MAPK9	+	182/293	0.00001994	3
725	VEGFR-1	FLT1	+	164/261	0.00002011	3
726	TBK1	TBK1	+	164/261	0.00002011	3
727	BAT3	BAG6	+	37/46	0.00002028	2
728	Dystroglycan	DAG1	+	123/189	0.00002045	3
730	c-Kit	KIT	+	131/203	0.00002087	3
729	mGluR5	GRM5	+	131/203	0.00002087	3
731	OTUD1	OTUD1	-	135/210	0.0000209	3
732	MSK2	RPS6KA4	-	35/43	0.00002097	2
733	NFKBIB	NFKBIB	-	98/146	0.00002133	3
734	KLF11 (TIEG2)	KLF11	-	64/89	0.00002161	2
735	PDLIM2	PDLIM2	-	116/177	0.00002161	3
736	CtBP		-	148/233	0.00002205	3
737	UBB	UBB	-	170/272	0.00002231	2
738	Myosin VI	MYO6	-	76/109	0.00002328	3
739	CtBP1	CTBP1	-	153/242	0.00002332	3
740	Golgin-95	GOLGA2	-	92/136	0.00002355	3
741	GRIPAP1	GRIPAP1	+	99/148	0.00002415	3
742	NFKBID	NFKBID	+	121/186	0.0000244	3
743	IRAK1BP1	IRAK1BP1	+	125/193	0.00002471	3
744	CDC20	CDC20	-	62/86	0.00002542	2
745	YTHDF1	YTHDF1	+	177/285	0.00002602	3
746	miR-520d-5p	MIR520D	-	146/230	0.00002617	3
747	HB-EGF	HBEGF	+	142/223	0.00002658	3
748	KDM2A	KDM2A	-	80/116	0.000027	2
749	CNTN1 (F3)	CNTN1	+	68/96	0.0000273	3
750	RGC32	RGCC	+	68/96	0.0000273	2
751	SFK		+	151/239	0.00002764	3
752	SFK		+	151/239	0.00002764	3
753	miR-548d-3p	MIR548D2	-	90/133	0.00002816	3
754	miR-548d-3p	MIR548D1	-	90/133	0.00002816	3
755	miR-let-7a-2-3p	MIRLET7A2	-	156/248	0.00002896	3
756	Sirtuin6	SIRT6	-	119/183	0.00002912	2
757	PRMT6	PRMT6	+	179/289	0.00002939	3
758	AF-9	MLLT3	+	135/211	0.00002946	3
759	PDCD4	PDCD4	-	60/83	0.00002987	2
760	MYST1	KAT8	+	108/164	0.0000299	2
761	Cullin 2	CUL2	-	84/123	0.0000305	2
762	Nectin-1	NECTIN1	+	81/118	0.00003146	3

763	miR-4728-5p	MIR4728	-	105/159	0.00003207	3
764	miR-1277-5p	MIR1277	+	78/113	0.00003223	3
765	HOXA-AS3	HOXA-AS3	+	66/93	0.00003234	2
766	LINC-PINT	LINC-PINT	+	66/93	0.00003234	2
767	ANAPC1	ANAPC1	+	128/199	0.00003235	3
768	E-cadherin	CDH1	-	75/108	0.00003275	2
769	UBE3C	UBE3C	-	28/33	0.00003309	2
770	PIAS1	PIAS1	-	186/302	0.00003346	3
771	ASPP2	TP53BP2	+	88/130	0.00003369	2
772	FKBP8	FKBP8	-	113/173	0.00003413	3
773	p300	EP300	+	34/42	0.00003439	1
774	hsa-miR-3182	MIR3182	-	95/142	0.00003457	3
775	NF-kB2 (p100)	NFKB2	-	146/231	0.00003609	3
776	LRRK2	LRRK2	+	173/279	0.0000361	3
777	PKA-cat beta	PRKACB	+	61/85	0.00003698	2
778	SMARCA5	SMARCA5	+	53/72	0.00003778	3
779	MCM5	MCM5	+	64/90	0.00003829	2
780	F16P	FBP1	-	174/281	0.00003835	3
781	TrkB	NTRK2	-	103/156	0.00003836	3
782	CD36	CD36	+	76/110	0.00003846	3
783	miR-301a-3p	MIR301A	-	89/132	0.00003846	2
784	RIG-I	DDX58	+	126/196	0.00003853	3
785	ZNF451	ZNF451	-	156/249	0.00003932	3
786	RANBP7	IPO7	-	143/226	0.00003973	3
787	CBP	CREBBP	+	215/355	0.00004057	3
788	ADAM12	ADAM12	+	111/170	0.00004076	3
789	DOCK8	DOCK8	+	100/151	0.00004106	3
790	miR-1470	MIR1470	-	135/212	0.00004118	3
791	BRG1	SMARCA4	+	185/301	0.00004154	3
792	ITGAV	ITGAV	+	131/205	0.00004167	3
793	FGFR4	FGFR4	-	83/122	0.00004204	3
794	SAMM50	SAMM50	-	104/158	0.00004274	3
795	Sequestosome	SQSTM1	-	104/158	0.00004274	2
796	miR-520c-5p	MIR520C	-	59/82	0.00004358	2
797	TFIIA gamma chain	GTF2A2	+	80/117	0.00004359	3
799	PCBP-2	PCBP2	+	90/134	0.00004373	2
798	Leukocyte elastase	ELANE	-	140/221	0.00004373	3
800	PU.1	SPI1	+	23/26	0.00004399	1
801	c-Rel (NF-kB	REL	+	136/214	0.00004455	3
802	XIST	XIST	-	77/112	0.0000449	2
803	miR-302a-3p	MIR302A	-	77/112	0.0000449	2
804	miR-875-3p	MIR875	-	62/87	0.0000453	3
805	BVRA	BLVRA	+	128/200	0.00004565	3
806	C1orf86	FAAP20	+	74/107	0.00004591	3
807	SOCS1	SOCS1	-	74/107	0.00004591	2
808	SP7	SP7	+	145/230	0.000046	3
809	TORC2	CRTC2	+	182/296	0.00004612	3
810	UTX	KDM6A	+	197/323	0.00004626	3

811	HSP70		+	65/92	0.00004632	2
812	ATF-4	ATF4	+	173/280	0.00004788	3
813	miR-320d	MIR320D1	-	109/167	0.0000487	3
814	miR-320d	MIR320D2	-	109/167	0.0000487	3
815	TTI1	TTI1	+	142/225	0.00005072	3
816	SAMHD1	SAMHD1	-	25/29	0.00005186	2
817	CREB3	CREB3	-	126/197	0.00005432	3
818	miR-211-3p	MIR211	-	99/150	0.00005489	3
820	NF-AT1(NFATC2)	NFATC2	+	152/243	0.00005494	3
819	MSK2	RPS6KA4	-	152/243	0.00005494	3
821	TFIIB	GTF2B	+	66/94	0.00005556	2
822	miR-493-5p	MIR493	-	171/277	0.00005631	3
823	TLR4	TLR4	+	27/32	0.00005654	2
824	p38gamma	MAPK12	+	82/121	0.00005774	2
826	alpha-V/beta-5	ITGB5	+	131/206	0.00005815	3
827	alpha-V/beta-5	ITGAV	+	131/206	0.00005815	3
825	UCHL3	UCHL3	+	131/206	0.00005815	3
828	GBP1	GBP1	-	107/164	0.00005818	3
829	DDEF1	ASAP1	+	47/63	0.00005853	3
830	miR-1248	MIR1248	-	153/245	0.0000587	3
831	Rad52	RAD52	+	111/171	0.00005911	3
832	miR-130a-3p	MIR130A	-	154/247	0.00006264	2
833	ERG	ERG	+	183/299	0.00006366	3
834	TRAF5	TRAF5	+	73/106	0.00006411	3
835	AKR1C3	AKR1C3	+	141/224	0.00006458	3
836	TELO2	TELO2	+	141/224	0.00006458	3
837	NF-kB1 (p50)	NFKB1	+	164/265	0.00006535	3
838	C11orf51	ANAPC15	+	64/91	0.00006608	3
839	miR-483-3p	MIR483	-	137/217	0.00006628	3
840	UBB	UBB	-	194/319	0.00006648	3
841	miR-26b-3p	MIR26B	-	146/233	0.00006716	3
842	ANAPC11	ANAPC11	-	101/154	0.0000679	3
843	Miz-1	ZBTB17	-	170/276	0.00007011	3
844	ABCA1	ABCA1	+	125/196	0.00007012	3
845	ASB2	ASB2	-	109/168	0.00007057	3
846	Ankyrin-B	ANK2	-	53/73	0.00007082	3
847	MCAM	MCAM	-	53/73	0.00007082	3
848	SOCS5	SOCS5	-	134/212	0.00007305	3
849	JMJD2D	KDM4D	+	186/305	0.00007439	3
850	DR6(TNFRSF21)	TNFRSF21	+	74/108	0.00007455	3
851	miR1a-1-5p	MIR1-1	-	74/108	0.00007455	3
852	NMI	NMI	+	157/253	0.00007564	3
853	NRB54	NONO	+	126/198	0.00007593	3
854	ISG15	ISG15	+	139/221	0.0000765	3
855	NF-AT5	NFAT5	+	71/103	0.00007662	2
856	CRIP2	CRIP2	-	59/83	0.00007728	2
857	TRFP	MED20	+	95/144	0.0000786	3
858	CDK6	CDK6	+	81/120	0.00007902	2

859	HBXAP	RSF1	+	51/70	0.00008302	2
860	PIKE(CENTG1)	AGAP2	+	123/193	0.00008339	3
861	GRK6	GRK6	+	92/139	0.00008426	3
862	FLJ20625	LAMTOR1	+	111/172	0.00008482	3
863	LRRFIP1	LRRFIP1	+	34/43	0.00008508	2
864	Neurogenin 3	NEUROG3	+	34/43	0.00008508	2
865	TRAF3	TRAF3	-	96/146	0.00008752	3
866	APEX	APEX1	+	124/195	0.00009026	2
868	CXCR4	CXCR4	+	137/218	0.00009061	3
867	PICT-1	NOP53	-	137/218	0.00009061	3
869	MSR1	MSR1	-	104/160	0.00009155	3
870	PTP4A3	PTP4A3	+	86/129	0.00009597	3
871	MIR31HG	MIR31HG	+	186/306	0.00009613	3
872	mTORC2	PRR5	+	147/236	0.0000967	3
873	mTORC2	MLST8	+	147/236	0.0000967	3
874	mTORC2	MTOR	+	147/236	0.0000967	3
875	mTORC2	MAPKAP1	+	147/236	0.0000967	3
876	mTORC2	RICTOR	+	147/236	0.0000967	3
877	HMG14	HMGN1	+	125/197	0.00009754	3
878	NF-kB p50/p65	RELA	+	28/34	0.00009756	2
879	NF-kB p50/p65	NFKB1	+	28/34	0.00009756	2
880	ARNT	ARNT	+	197/326	0.0000984	3
881	gp130	IL6ST	+	152/245	0.00009888	3
882	TRIM35	TRIM35	-	121/190	0.00009919	3
883	VGLL4	VGLL4	-	101/155	0.00009931	3
884	UBAP2L	UBAP2L	+	90/136	0.0001009	3
885	miR-1299	MIR1299	-	44/59	0.0001019	2
886	Usp24	USP24	+	167/272	0.0001023	3
887	Lysyl oxidase	LOX	-	73/107	0.0001032	3
888	Malin	NHLRC1	+	94/143	0.0001047	3
889	MKP-1	DUSP1	-	149/240	0.0001096	3
890	PHLPPL	PHLPP2	-	131/208	0.0001105	3
891	miR-1304-5p	MIR1304	-	64/92	0.0001113	3
892	OR3A4	OR3A4P	+	91/138	0.0001127	3
893	NFKBIA	NFKBIA	-	91/138	0.0001127	2
894	DDA3	PSRC1	-	84/126	0.000115	3
895	I-kB		-	132/210	0.0001186	3
896	Karyopherin alpha 4	KPNA4	+	103/159	0.0001202	2
897	SIGIRR	SIGIRR	-	103/159	0.0001202	3
898	CRSP8 (CRSP34)	MED27	+	128/203	0.0001219	3
899	miR-541-3p	MIR541	-	137/219	0.000123	3
900	MEF2A	MEF2A	+	35/45	0.0001235	2
901	RPS3	RPS3	+	92/140	0.0001254	2
902	miR-21-5p	MIR21	-	156/253	0.0001254	2
903	TRUSS	TRPC4AP	+	96/147	0.0001286	3
904	miR-506-3p	MIR506	-	116/182	0.0001296	2
905	Polycystin	PKD1	+	108/168	0.0001318	3
906	CDK5R1 (p35)	CDK5R1	+	157/255	0.0001327	3

907	RhoE	RND3	+	59/84	0.0001332	2
908	BRD9	BRD9	+	162/264	0.0001335	3
909	EPC1	EPC1	+	40/53	0.0001343	2
910	WDR62	WDR62	+	89/135	0.0001349	3
911	Karyopherin alpha 3	KPNA3	+	21/24	0.0001386	2
912	BRSK2	BRSK2	+	93/142	0.0001391	3
913	Tuberin	TSC2	-	93/142	0.0001391	3
914	miR-641	MIR641	-	130/207	0.0001405	3
915	CALCOCO2	CALCOCO2	-	97/149	0.0001419	3
916	hsa-miR-770-5p	MIR770	+	109/170	0.0001436	3
917	TIFA	TIFA	+	105/163	0.0001441	3
918	miR-766-5p	MIR766	-	179/295	0.000146	3
919	E2F1	E2F1	+	159/259	0.0001484	3
920	RACK1	RACK1	+	90/137	0.0001501	2
921	miR-582-5p	MIR582	-	131/209	0.0001505	3
922	ZAP70	ZAP70	+	145/234	0.0001525	3
923	CHFR	CHFR	-	127/202	0.0001553	3
924	c-FLIP	CFLAR	+	98/151	0.0001562	3
925	NOTCH2 (2ICD)	NOTCH2	+	60/86	0.0001585	2
926	EYA4	EYA4	+	57/81	0.0001587	3
927	BCR	BCR	-	141/227	0.0001591	3
928	COP1	COP1	-	87/132	0.0001616	2
929	JAK1	JAK1	+	146/236	0.0001619	3
930	RIPK4	RIPK4	+	73/108	0.0001629	3
931	Adenosine A1	ADORA1	+	137/220	0.0001656	3
932	NET1(TSPAN1)	TSPAN1	+	99/153	0.0001715	3
933	Granzyme B	GZMB	-	147/238	0.0001718	3
934	CIDEC	CIDEC	-	103/160	0.0001719	3
935	miR-589-5p	MIR589	+	133/213	0.000172	3
936	CDK9	CDK9	+	188/312	0.0001731	3
937	IRF7	IRF7	+	49/68	0.000179	3
938	CHFR	CHFR	-	64/93	0.0001829	2
939	TARBP2	TARBP2	-	92/141	0.0001841	3
940	USP25	USP25	-	100/155	0.0001878	3
942	Fyn	FYN	+	121/192	0.0001895	3
941	DUB3	USP17L2	+	121/192	0.0001895	3
943	Karyopherin alpha 4	KPNA4	+	164/269	0.0001932	3
945	miR-3928-3p	MIR3928	-	78/117	0.0001981	3
944	KPC1	RNF123	-	78/117	0.0001981	3
946	NF-kB p50/p65	RELA	+	18/20	0.0002012	1
947	NF-kB p50/p65	NFKB1	+	18/20	0.0002012	1
950	BAF60A	SMARCD1	+	165/271	0.0002033	3
948	miR-450a-5p	MIR450A2	-	165/271	0.0002033	3
949	miR-450a-5p	MIR450A1	-	165/271	0.0002033	3
951	FAM83B	FAM83B	+	136/219	0.0002083	3
952	STYK1	STYK1	+	161/264	0.0002151	3
953	SOX9	SOX9	+	156/255	0.0002157	3
955	RBCK1	RBCK1	-	59/85	0.0002236	2

954	DVL-1	DVL1	-	59/85	0.0002236	2
957	Bid	BID	+	142/230	0.0002247	3
956	miR-330-5p	MIR330	-	142/230	0.0002247	3
958	ZFPM2-AS1	ZFPM2-AS1	+	119/189	0.0002248	3
959	RAD1	RAD1	+	133/214	0.0002311	3
960	Tip60	KAT5	+	173/286	0.0002324	3
962	TAF4B	TAF4B	+	69/102	0.0002343	2
961	NRBP	NRBP1	-	69/102	0.0002343	3
963	SHIP	INPP5D	-	111/175	0.000236	3
964	ECSIT	ECSIT	+	87/133	0.0002386	3
965	RAIDD	CRADD	+	12/12	0.0002441	2
966	NLRX1	NLRX1	-	95/147	0.0002448	3
967	NDR2	STK38L	-	134/216	0.0002459	3
968	miR-597-5p	MIR597	-	80/121	0.0002493	3
969	CBP/P300	EP300	+	125/200	0.0002497	3
970	CBP/P300	CREBBP	+	125/200	0.0002497	3
971	miR-514b-5p	MIR514B	+	73/109	0.0002525	3
972	STING (TMEM173)	STING1	+	28/35	0.0002541	2
973	PKA-cat beta	PRKACB	+	121/193	0.0002585	3
974	NRIF3	ITGB3BP	-	22/26	0.0002668	2
975	MKL2	MRTFB	-	57/82	0.0002674	2
976	RASSF6	RASSF6	+	96/149	0.0002678	3
977	IRF6	IRF6	+	81/123	0.000278	3
979	ARF6	ARF6	+	101/158	0.0002901	3
978	HNF1A-AS1	HNF1A-AS1	-	101/158	0.0002901	3
980	miR-373-3p	MIR373	-	89/137	0.0002905	2
981	Lck	LCK	+	114/181	0.0002948	3
982	miR-3134	MIR3134	+	123/197	0.0002955	3
983	BRD7	BRD7	+	152/249	0.0002967	3
984	Kallikrein 6	KLK6	+	82/125	0.000309	3
985	UNRIP	STRAP	+	82/125	0.000309	2
986	miR-4717-5p	MIR4717	+	38/51	0.0003105	3
988	DACT3	DACT3	+	58/84	0.000314	3
987	URI	URI1	+	58/84	0.000314	2
989	miR-223-3p	MIR223	-	164/271	0.0003213	3
990	TOB2	TOB2	-	139/226	0.0003298	3
991	A20	TNFAIP3	-	149/244	0.0003304	3
992	UNRIP	STRAP	+	170/282	0.0003308	3
993	IL-22 receptor	IL22RA1	+	144/235	0.0003309	3
994	IL-22 receptor	IL10RB	+	144/235	0.0003309	3
995	LEF1-AS1	LEF1-AS1	+	116/185	0.0003391	3
996	FGFR3	FGFR3	+	160/264	0.0003414	3
997	GFI-1	GFI1	-	160/264	0.0003414	3
998	Emerin	EMD	-	41/56	0.0003428	2
999	miR-7-5p	MIR7-1	-	99/155	0.0003458	2
1000	miR-7-5p	MIR7-2	-	99/155	0.0003458	2
1001	miR-7-5p	MIR7-3	-	99/155	0.0003458	2
1002	miR-520e-3p	MIR520E	-	131/212	0.0003634	3

1003	MyD88	MYD88	+	17/19	0.0003643	2
1004	JAK2	JAK2	+	146/239	0.0003679	3
1005	miR-383-5p	MIR383	-	141/230	0.0003682	3
1006	BATF2	BATF2	+	80/122	0.0003704	2
1007	PPAR-gamma	PPARG	-	80/122	0.0003704	2
1008	MT-TRX	TXN2	-	56/81	0.0003761	3
1009	VISA	MAVS	+	53/76	0.0003823	2
1010	TAFII55	TAF7	-	109/173	0.0003868	3
1011	CDC42	CDC42	+	105/166	0.000397	3
1012	CFTR	CFTR	-	123/198	0.0003972	3
1013	miR-576-3p	MIR576	-	39/53	0.0004012	2
1014	SOCS2	SOCS2	+	101/159	0.0004057	3
1015	IL-6 receptor	IL6R	+	148/243	0.0004076	3
1016	IL-6 receptor	IL6ST	+	148/243	0.0004076	3
1017	p90RSK2(RPS6KA3	RPS6KA3	+	133/216	0.000408	3
1018	PTPN2	PTPN2	-	143/234	0.0004096	3
1019	miR-148a-3p	MIR148A	-	81/124	0.0004101	2
1020	miR-212-5p	MIR212	+	27/34	0.0004107	2
1021	Alpha-actinin 4	ACTN4	+	164/272	0.0004109	3
1022	Symplekin	SYMPK	+	85/131	0.000416	3
1023	LXN	LXN	-	89/138	0.000418	3
1024	BRD4	BRD4	+	106/168	0.0004274	2
1025	miR-29c-5p	MIR29C	-	106/168	0.0004274	3
1027	IRF3	IRF3	+	19/22	0.0004277	1
1026	Myotrophin	MTPN	-	19/22	0.0004277	2
1028	miR-653-5p	MIR653	-	129/209	0.0004284	3
1029	TRPM7	TRPM7	-	57/83	0.0004388	3
1030	DVL-2	DVL2	-	57/83	0.0004388	2
1031	BRD3	BRD3	+	25/31	0.000439	2
1032	miR-615-5p	MIR615	-	111/177	0.0004452	3
1033	IPMK	IPMK	+	150/247	0.0004501	3
1034	MKP-1	DUSP1	-	23/28	0.0004561	2
1035	miR-637	MIR637	-	135/220	0.0004562	3
1036	ATF-7	ATF7	+	86/133	0.0004566	3
1037	TRAM	TICAM2	+	116/186	0.000459	3
1038	Adenosine A2b	ADORA2B	-	121/195	0.0004694	3
1039	Neogenin	NEO1	+	32/42	0.0004703	3
1040	STAT1	STAT1	+	103/163	0.0004721	2
1041	WRCH-1	RHOU	+	112/179	0.0004764	3
1042	Karyopherin alpha 1	KPNA1	+	141/231	0.0004806	3
1043	IRF7	IRF7	+	11/11	0.0004883	1
1044	KIR2DL4	KIR2DL4	-	122/197	0.0004989	3
1045	miR-378b	MIR378B	+	118/190	0.0005213	3
1046	RASSF1	RASSF1	+	164/273	0.0005227	3
1047	NF-AT2(NFATC1)	NFATC1	+	96/151	0.0005324	2
1048	DAPK1	DAPK1	+	30/39	0.0005325	2
1049	RPL11	RPL11	-	128/208	0.0005346	3
1051	PTPD1	PTPN21	+	80/123	0.0005422	3

1050	Rab-8	RAB8A	-	80/123	0.0005422	3
1052	Annexin VI	ANXA6	+	114/183	0.0005433	3
1053	UTX	KDM6A	+	46/65	0.000545	2
1054	C6orf106	ILRUN	+	88/137	0.0005455	3
1055	NXF1	NXF1	-	62/92	0.0005555	3
1056	MAP1	MOAP1	+	101/160	0.0005615	3
1057	miR-138-1-3p	MIR138-1	-	110/176	0.0005647	3
1058	EG624219	ANGPTL8	-	129/210	0.0005654	3
1059	JNK3(MAPK10)	MAPK10	+	155/257	0.0005693	3
1060	LAT	LAT	+	97/153	0.0005752	3
1063	HSF1	HSF1	+	73/111	0.0005755	2
1062	Nod2 (CARD15)	NOD2	+	73/111	0.0005755	3
1061	Alpha-actinin 4	ACTN4	+	73/111	0.0005755	2
1064	NIPBL	NIPBL	+	106/169	0.0005849	3
1065	MAP1LC3A	MAP1LC3A	+	106/169	0.0005849	3
1066	Trabid	ZRANB1	+	125/203	0.0005949	3
1067	miR-199a-5p	MIR199A1	-	111/178	0.000603	2
1068	miR-199a-5p	MIR199A2	-	111/178	0.000603	2
1069	NF-AT2(NFATC1)	NFATC1	+	151/250	0.0006065	3
1070	USP13	USP13	+	141/232	0.0006233	3
1071	DRIP130	MED23	+	33/44	0.00063	2
1072	Plakophilin 3	PKP3	-	131/214	0.0006305	3
1073	Ubiquilin-1	UBQLN1	-	44/62	0.0006495	3
1074	NALP4	NLRP4	-	78/120	0.0006497	3
1075	CD69	CD69	+	118/191	0.0006951	3
1076	RMRP	RMRP	+	91/143	0.0006991	3
1077	SOX10	SOX10	+	123/200	0.0007008	3
1078	HSP60	HSPD1	+	36/49	0.0007013	2
1079	T3JAM	TRAF3IP3	-	87/136	0.0007093	3
1080	NF-kB2 (p100)	NFKB2	-	64/96	0.0007122	2
1081	Smac/Diablo	DIABLO	-	79/122	0.000714	3
1082	GIT1	GIT1	+	114/184	0.0007282	3
1085	TACI(TNFRSF13B)	TNFRSF13B	+	96/152	0.0007381	3
1083	GIT2	GIT2	-	96/152	0.0007381	3
1084	NUMBL	NUMBL	-	96/152	0.0007381	3
1086	BMX	BMX	+	145/240	0.0007544	3
1087	NF-kB p65/p65	RELA	+	22/27	0.0007569	2
1088	HOXA10	HOXA10	+	22/27	0.0007569	2
1089	SHANK2	SHANK2	+	61/91	0.0007583	3
1090	PTPN12	PTPN12	-	101/161	0.0007692	3
1091	miR-194-5p	MIR194-1	-	125/204	0.0007826	3
1092	miR-194-5p	MIR194-2	-	125/204	0.0007826	3
1093	NF-kB1 (p50)	NFKB1	+	106/170	0.0007928	2
1094	HPK1(MAP4K1)	MAP4K1	+	58/86	0.0008028	3
1095	HSP20	HSPB6	+	93/147	0.0008157	3
1096	BAL	PARP9	+	102/163	0.0008234	3
1097	PEDF (serpinF1)	SERPINF1	-	121/197	0.0008254	3
1098	Par-4	PAWR	-	142/235	0.0008416	3

1099	IFN-gamma receptor	IFNGR1	+	112/181	0.0008603	3
1100	IFN-gamma receptor	IFNGR2	+	112/181	0.0008603	3
1101	FAM105B	OTULIN	+	103/165	0.0008801	3
1102	CNOT7	CNOT7	-	103/165	0.0008801	3
1103	miR-148a-3p	MIR148A	-	143/237	0.0008811	3
1104	miR-138-5p	MIR138-2	-	138/228	0.000896	2
1105	miR-138-5p	MIR138-1	-	138/228	0.000896	2
1106	miR-935	MIR935	-	108/174	0.0009001	3
1107	SIX4	SIX4	+	46/66	0.0009291	3
1108	ZIN	RNF216	-	78/121	0.0009365	3
1109	TRAF6	TRAF6	+	95/151	0.0009439	2
1110	Piccolo	PCLO	+	109/176	0.000957	3
1111	miR-516a-5p	MIR516A2	-	109/176	0.000957	3
1112	miR-516a-5p	MIR516A1	-	109/176	0.000957	3
1114	Alpha-actinin 1	ACTN1	+	63/95	0.0009622	3
1113	NF-kB1 (p105)	NFKB1	-	63/95	0.0009622	2
1115	STO	NSD1	+	56/83	0.0009657	2
1116	ASXL1	ASXL1	+	100/160	0.0009769	3
1117	TFIID	TAF10	+	67/102	0.0009964	3
1118	TFIID	TAF7L	+	67/102	0.0009964	3
1119	TFIID	TAF15	+	67/102	0.0009964	3
1120	TFIID	TAF7	+	67/102	0.0009964	3
1121	TFIID	TAF8	+	67/102	0.0009964	3
1122	TFIID	TAF4B	+	67/102	0.0009964	3
1123	TFIID	TAF1	+	67/102	0.0009964	3
1124	TFIID	TAF13	+	67/102	0.0009964	3
1125	TFIID	TAF9B	+	67/102	0.0009964	3
1126	TFIID	TAF12	+	67/102	0.0009964	3
1127	TFIID	TAF2	+	67/102	0.0009964	3
1128	TFIID	TAF9	+	67/102	0.0009964	3
1129	TFIID	TBP	+	67/102	0.0009964	3
1130	TFIID	TAF4	+	67/102	0.0009964	3
1131	TFIID	TAF6	+	67/102	0.0009964	3
1132	TFIID	TAF3	+	67/102	0.0009964	3
1133	TFIID	TAF5	+	67/102	0.0009964	3
1134	TFIID	TAF11	+	67/102	0.0009964	3
1135	IRF2	IRF2	-	67/102	0.0009964	2
1136	RANBP10	RANBP10	+	130/214	0.001013	3
1137	PSTPIP2	PSTPIP2	+	125/205	0.001022	3
1139	SAD1 (USP39)	USP39	+	147/245	0.001051	3
1138	miR-155-5p	MIR155	-	147/245	0.001051	2
1140	CDK3	CDK3	+	142/236	0.001075	3
1141	SCP1	CTDSP1	-	116/189	0.001083	3
1142	miR-1301-3p	MIR1301	-	38/53	0.001095	2
1143	miR-518b	MIR518B	-	84/132	0.001097	3
1144	XAF1	XAF1	+	132/218	0.001116	3
1145	DR4(TNFRSF10A)	TNFRSF10A	+	72/111	0.001119	3
1146	IKK-beta	IKBKB	+	72/111	0.001119	2

1147	Connexin 40	GJA5	+	93/148	0.001122	3
1149	Alpha-1B	ADRA1B	+	61/92	0.001157	3
1150	Alpha-1D	ADRA1D	+	61/92	0.001157	3
1148	Keratin 8	KRT8	+	61/92	0.001157	3
1151	PDK1	PDK1	+	144/240	0.001173	3
1152	PHF8	PHF8	+	123/202	0.001199	3
1153	miR-223-3p	MIR223	-	108/175	0.0012	2
1154	MyD88	MYD88	+	129/213	0.001245	3
1155	SENP3	SENP3	+	140/233	0.001254	3
1156	PML	PML	-	114/186	0.001275	2
1157	MKP-5	DUSP10	-	19/23	0.0013	2
1158	VDAC 1	VDAC1	-	100/161	0.001317	3
1159	ELF3	ELF3	+	66/101	0.001327	2
1160	Galectin-9	LGALS9	+	91/145	0.001334	3
1161	miR-370-5p	MIR370	-	70/108	0.001342	3
1162	CFTR	CFTR	-	59/89	0.001393	2
1163	miR-219-1-3p	MIR219A1	+	106/172	0.001417	3
1164	AGTR1	AGTR1	+	116/190	0.001419	3
1165	PITPNA-AS1	PITPNA-AS1	+	111/181	0.001422	3
1166	PKC-epsilon	PRKCE	+	63/96	0.001439	2
1167	CD45	PTPRC	-	79/124	0.001444	3
1168	miR-576-3p	MIR576	-	127/210	0.001457	3
1169	LGR4	LGR4	-	97/156	0.001465	3
1170	TGM2	TGM2	+	60/91	0.001556	2
1171	NEUR1	NEU1	-	113/185	0.001583	3
1172	IGBP1	IGBP1	-	108/176	0.001586	3
1173	ULK2	ULK2	+	53/79	0.001592	3
1174	SFRS2 (SC-35)	SRSF2	+	135/225	0.001632	3
1175	APPBP2	APPBP2	+	119/196	0.001651	3
1176	Bcl-6	BCL6	-	12/13	0.001709	1
1177	BMI-1	BMI1	-	12/13	0.001709	1
1178	BMI-1	COMMD3-	-	12/13	0.001709	1
1179	RORET	TRIM38	-	95/153	0.001736	3
1180	miR-3140-3p	MIR3140	-	131/218	0.001746	3
1181	DARPP-32	PPP1R1B	+	121/200	0.001817	3
1182	4EHP	EIF4E2	+	132/220	0.001823	3
1184	MAPKBP1	MAPKBP1	+	96/155	0.001847	3
1183	CaMK II alpha	CAMK2A	-	96/155	0.001847	3
1185	CBLC	CBLC	-	111/182	0.001861	3
1186	HSP40	DNAJB1	-	111/182	0.001861	3
1187	CHP1	CHORDC1	+	78/123	0.001866	3
1188	CHMP4B	CHMP4B	+	58/88	0.001873	3
1189	BRD7	BRD7	+	51/76	0.001918	2
1190	IRF4	IRF4	+	38/54	0.001919	2
1191	PKM2	PKM	+	108/177	0.002078	2
1193	AP complex 2	AP2B1	+	59/90	0.002083	3
1194	AP complex 2	AP2A1	+	59/90	0.002083	3
1195	AP complex 2	AP2A2	+	59/90	0.002083	3

1196	AP complex 2	AP2M1	+	59/90	0.002083	3
1197	AP complex 2	AP2S1	+	59/90	0.002083	3
1192	A20	TNFAIP3	-	59/90	0.002083	2
1198	NFAT-90	ILF3	+	124/206	0.002085	3
1199	miR-202-5p	MIR202	-	33/46	0.002267	2
1200	AAMP	AAMP	+	110/181	0.0023	3
1201	SSTR5	SSTR5	-	110/181	0.0023	3
1202	UNC5B	UNC5B	+	36/51	0.002301	3
1203	TLE2	TLE2	+	95/154	0.002319	3
1204	NRF2	NFE2L2	-	105/172	0.002321	3
1205	miR-194-3p	MIR194-2	+	81/129	0.002325	3
1206	PRMT1	PRMT1	+	81/129	0.002325	2
1207	microRNA 21	MIR21	-	117/194	0.00249	3
1208	Annexin V	ANXA5	-	69/108	0.002512	3
1209	Profilin		+	61/94	0.002539	3
1210	miR-766-5p	MIR766	-	28/38	0.002549	2
1211	MBNL1	MBNL1	+	102/167	0.002592	3
1212	miR-205-3p	MIR205	-	118/196	0.002604	3
1213	TLR3	TLR3	+	103/169	0.002731	3
1214	AdipoR1	ADIPOR1	+	34/48	0.002758	3
1217	RIOK3	RIOK3	-	62/96	0.002786	3
1215	HSPA1B	HSPA1A	-	62/96	0.002786	2
1216	HSPA1B	HSPA1B	-	62/96	0.002786	2
1218	TrkA	NTRK1	-	109/180	0.002834	3
1219	miR-1276	MIR1276	-	120/200	0.002843	3
1220	miR-4288	MIR4288	-	104/171	0.002873	3
1221	NF-kB p52/RelB	NFKB2	+	21/27	0.002962	2
1222	NF-kB p52/RelB	RELB	+	21/27	0.002962	2
1223	NF-kB p65/c-Rel	RELA	+	21/27	0.002962	2
1224	NF-kB p65/c-Rel	REL	+	21/27	0.002962	2
1225	EGFR	EGFR	+	110/182	0.002971	2
1226	JMJD6	JMJD6	-	55/84	0.003019	2
1228	TAK1(MAP3K7)	MAP3K7	+	116/193	0.003043	3
1227	IMP1(ZBP1)	IGF2BP1	+	116/193	0.003043	2
1229	RSAD2	RSAD2	+	63/98	0.003046	3
1231	NGAL	LCN2	+	111/184	0.003112	3
1230	PRMT8	PRMT8	-	111/184	0.003112	3
1232	BLIMP1 (PRDI-BF1)	PRDM1	-	11/12	0.003174	1
1233	miR-25-5p	MIR25	-	117/195	0.003178	3
1234	FTO	FTO	-	35/50	0.0033	2
1236	NF-kB		+	19/24	0.003305	1
1237	TLR9	TLR9	+	19/24	0.003305	3
1235	RI58	IFIT5	-	19/24	0.003305	2
1238	RRAD	RRAD	-	60/93	0.003348	2
1239	HSP70		+	102/168	0.003376	3
1240	Mucin 13	MUC13	+	113/188	0.003404	3
1241	Aif	AIFM1	+	87/141	0.003415	3
1242	TRIM35	TRIM35	-	17/21	0.003599	2

1243	Tensin 4	TNS4	+	109/181	0.003642	3
1244	IKK-epsilon	IKBKE	-	57/88	0.003672	2
1245	TMEM16A	ANO1	+	110/183	0.003808	3
1246	IBP2	IGFBP2	+	110/183	0.003808	3
1247	TXNIP (VDUP1)	TXNIP	-	84/136	0.003812	2
1249	VAV-2	VAV2	+	94/154	0.003814	3
1248	miR-345-3p	MIR345	-	94/154	0.003814	3
1250	NF-AT4(NFATC3)	NFATC3	+	36/52	0.003894	2
1251	HGF receptor (Met)	MET	+	36/52	0.003894	2
1252	TLR8	TLR8	-	66/104	0.0039	3
1253	SAHH2	AHCYL1	-	58/90	0.004023	3
1254	RUVBL2	RUVBL2	-	85/138	0.004042	2
1255	Cullin 1	CUL1	-	22/29	0.004065	2
1256	BRD3	BRD3	+	43/64	0.004073	3
1257	miR-26a-1-3p	MIR26A1	+	101/167	0.004159	3
1258	SBF2-AS1	SBF2-AS1	+	81/131	0.004256	3
1259	HtrA2	HTRA2	-	81/131	0.004256	3
1260	Prickle-1	PRICKLE1	+	63/99	0.004317	3
1261	PKC-delta	PRKCD	+	102/169	0.004358	2
1262	MafB	MAFB	-	25/34	0.004521	2
1263	HIF-prolyl		-	37/54	0.004537	2
1264	TFE3	TFE3	+	44/66	0.004605	3
1265	NF-kB p50/c-Rel	NFKB1	+	20/26	0.004678	2
1266	NF-kB p50/c-Rel	REL	+	20/26	0.004678	2
1267	PI3K reg class IA		+	93/153	0.004727	3
1268	Fetuin-A	AHSG	+	28/39	0.004738	3
1269	SH3PXD2A-AS1	SH3PXD2A-	+	83/135	0.004782	3
1270	MafB	MAFB	-	56/87	0.004837	3
1271	MAP1LC3B	MAP1LC3B	-	99/164	0.004878	3
1272	DEAF	DEAF1	+	41/61	0.004927	3
1273	PYGO2	PYGO2	-	105/175	0.004982	3
1274	CD79A	CD79A	+	100/166	0.005106	3
1275	Nibrin	NBN	+	45/68	0.005169	2
1276	TRIM47	TRIM47	+	61/96	0.005173	3
1277	PR (nuclear)	PGR	-	75/121	0.005306	2
1278	IRF7	IRF7	+	18/23	0.005311	2
1279	BRG1	SMARCA4	+	18/23	0.005311	1
1280	FEM1A	FEM1A	-	101/168	0.00534	3
1281	DVL-3	DVL3	-	62/98	0.005587	2
1283	Nod1	NOD1	+	32/46	0.005676	3
1282	BRD4/NUT fusion		+	32/46	0.005676	2
1284	NRIF3	ITGB3BP	-	82/134	0.005976	3
1285	MITF	MITF	+	43/65	0.006251	2
1286	GATA-3	GATA3	-	12/14	0.00647	1
1287	P4HB	P4HB	-	89/147	0.006539	3
1288	eEF1D	EEF1D	+	74/120	0.006688	3
1290	SEH1L	SEH1L	+	52/81	0.007	3
1289	Mitofusin 2	MFN2	-	52/81	0.007	3

1291	EZH2	EZH2	+	80/131	0.007059	2
1292	microRNA 21	MIR21	-	19/25	0.007317	2
1293	Calcineurin A		-	57/90	0.007433	2
1294	NPL4	NPLOC4	+	87/144	0.007685	3
1295	SRRF	HNRNPLL	+	7/7	0.007813	2
1296	PIAS2	PIAS2	-	67/108	0.007875	2
1297	DUB3	USP17L2	+	31/45	0.008047	2
1298	miR-20a-3p	MIR20A	+	89/148	0.008421	3
1300	PU.1	SPI1	+	42/64	0.008429	2
1299	RASA3	RASA3	-	42/64	0.008429	3
1301	CIRBP	CIRBP	+	35/52	0.008767	2
1302	Cyclophilin A	PPIA	+	74/121	0.008865	2
1303	ATOH8	ATOH8	-	39/59	0.009169	2
1304	WHISTLE	NSD3	-	80/132	0.009215	3
1305	miR-600	MIR600	-	36/54	0.009917	2
1306	MIF	MIF	+	71/116	0.00994	2

Supplementary Table 2. Causal reasoning results using DEGs that has log2FC ≥ 5 or ≤ −5 and FDR ≤ 0.05.

Key Hub

Rank	Molecular Entity	Gene	Predicted Activity	Correct/Total network predictions	Activity Prediction p-value	Calc. Distance
1	MAP3K2 (MEKK2)	MAP3K2	+	30/31	1.49E-08	3
2	PIAS4	PIAS4	-	31/33	6.54E-08	3
4	ErbB4	ERBB4	+	26/27	2.09E-07	3
3	ELKS	ERC1	+	26/27	2.09E-07	3
5	PD-1	PDCD1	-	26/27	2.09E-07	3
6	NCOA2	NCOA2	+	22/22	2.38E-07	2
8	c-Fos	FOS	+	25/26	4.02E-07	2
7	BTEB1	KLF9	-	25/26	4.02E-07	3
10	HNF1-alpha	HNF1A	+	28/30	4.34E-07	3
9	KIS	UHMK1	+	28/30	4.34E-07	3
11	IRF1	IRF1	+	33/37	5.42E-07	3
12	Fbxl19	FBXL19	-	30/33	7.01E-07	3
13	MIR205HG	MIR205HG	-	24/25	7.75E-07	3
14	NCOA3	NCOA3	+	27/29	8.12E-07	2
15	PIAS4	PIAS4	-	27/29	8.12E-07	2
18	IFNAR2	IFNAR2	+	29/32	1.28E-06	3
16	LOC145837	DRAIC	-	29/32	1.28E-06	3
17	miR-541-3p	MIR541	-	29/32	1.28E-06	3
19	TAFII70	TAF6	+	23/24	1.49E-06	3
24	NF-AT4(NFATC3)	NFATC3	+	23/24	1.49E-06	3
25	IRF1	IRF1	+	23/24	1.49E-06	2
20	Alpha-actinin 2	ACTN2	+	23/24	1.49E-06	3
22	Contactin 2	CNTN2	+	23/24	1.49E-06	3
21	miR-345-5p	MIR345	-	23/24	1.49E-06	2
23	Caspase-12	CASP12	-	23/24	1.49E-06	3
26	TRIM40	TRIM40	-	26/28	1.52E-06	3
28	ATF-5	ATF5	+	31/35	1.73E-06	3
27	LINC02605	LINC02605	+	31/35	1.73E-06	3
29	miR-383-5p	MIR383	-	19/19	1.91E-06	2
34	c-Src	SRC	+	28/31	2.33E-06	3
30	USP12	USP12	+	28/31	2.33E-06	3
32	CBP	CREBBP	+	28/31	2.33E-06	2
33	SHP-1	PTPN6	-	28/31	2.33E-06	3
31	LCoR (MLR2)	LCOR	-	28/31	2.33E-06	3
37	SRF	SRF	+	25/27	2.82E-06	3
36	BAF60c	SMARCD3	+	25/27	2.82E-06	3
35	miR-646	MIR646	-	25/27	2.82E-06	3
40	ALPHA-PIX	ARHGEF6	+	22/23	2.86E-06	3
38	PTTG3P	PTTG3P	+	22/23	2.86E-06	3
39	RIG-G	IFIT3	+	22/23	2.86E-06	3
41	p300	EP300	+	30/34	3.08E-06	2
44	PREX1	PREX1	+	18/18	3.82E-06	3

43	Calgranulin A	S100A8	+	18/18	3.82E-06	3
42	miR-502-5p	MIR502	-	18/18	3.82E-06	2
46	TBK1	TBK1	+	27/30	4.22E-06	3
45	miR-383-5p	MIR383	-	27/30	4.22E-06	3
47	PIG8	EI24	-	27/30	4.22E-06	3
54	BRG1	SMARCA4	+	24/26	5.25E-06	2
52	EG1	MED28	+	24/26	5.25E-06	3
48	miR-130a-3p	MIR130A	-	24/26	5.25E-06	2
49	PHLPPL	PHLPP2	-	24/26	5.25E-06	3
51	PPP2R2C	PPP2R2C	-	24/26	5.25E-06	3
50	NLRC5	NLRC5	-	24/26	5.25E-06	3
53	SHPS-1	SIRPA	-	24/26	5.25E-06	3
57	MSK1	RPS6KA5	+	29/33	5.46E-06	3
55	DOT1	DOT1L	+	29/33	5.46E-06	3
56	ARPP- 21	ARPP21	+	29/33	5.46E-06	3
64	c-Jun	JUN	+	21/22	5.48E-06	2
65	TERT	TERT	+	21/22	5.48E-06	2
58	TSLC1	CADM1	+	21/22	5.48E-06	3
62	MECOM	MECOM	-	21/22	5.48E-06	2
59	miR-548a-3p	MIR548A1	-	21/22	5.48E-06	3
60	miR-548a-3p	MIR548A3	-	21/22	5.48E-06	3
61	miR-548a-3p	MIR548A2	-	21/22	5.48E-06	3
63	SHP-1	PTPN6	-	21/22	5.48E-06	2
66	USP1	USP1	+	31/36	6.46E-06	3
67	Calgranulin B	S100A9	+	31/36	6.46E-06	3
70	CDK3	CDK3	+	26/29	7.62E-06	3
71	IKK-alpha	CHUK	+	26/29	7.62E-06	2
68	WDR48	WDR48	+	26/29	7.62E-06	3
69	PHF8	PHF8	+	26/29	7.62E-06	3
72	CUX1	CUX1	-	26/29	7.62E-06	3
83	IRF1	IRF1	+	17/17	7.63E-06	1
73	Kalirin	KALRN	+	17/17	7.63E-06	3
75	GEFT	ARHGEF25	+	17/17	7.63E-06	3
76	PLEKHG4	PLEKHG4	+	17/17	7.63E-06	3
82	DBL	MCF2	+	17/17	7.63E-06	3
79	GGTase-I	FNTA	+	17/17	7.63E-06	3
80	GGTase-I	PGGT1B	+	17/17	7.63E-06	3
78	c-IAP2	BIRC3	+	17/17	7.63E-06	2
74	miR-4674	MIR4674	-	17/17	7.63E-06	3
77	RhoGAP1	ARHGAP1	-	17/17	7.63E-06	3
81	RhoGDI beta	ARHGDIB	-	17/17	7.63E-06	3
93	KLF5	KLF5	+	23/25	9.72E-06	2
89	miR-221-5p	MIR221	+	23/25	9.72E-06	3
84	KIAA1914	AFAP1L2	+	23/25	9.72E-06	3
87	BAF170	SMARCC2	+	23/25	9.72E-06	3
92	Actin		+	23/25	9.72E-06	3
90	HEYL	HEYL	-	23/25	9.72E-06	3
86	miR-488-5p	MIR488	-	23/25	9.72E-06	3

85	PPP4C	PPP4C	-	23/25	9.72E-06	3
91	Sirtuin6	SIRT6	-	23/25	9.72E-06	2
88	TH1L	NELFCD	-	23/25	9.72E-06	3
114	VAV-3	VAV3	+	20/21	1.05E-05	3
109	GHR	GHR	+	20/21	1.05E-05	3
94	DOT1	DOT1L	+	20/21	1.05E-05	2
113	HSP27	HSPB1	+	20/21	1.05E-05	2
115	MSN (moesin)	MSN	+	20/21	1.05E-05	3
95	ILT2	LILRB1	-	20/21	1.05E-05	3
96	Siglec-7	SIGLEC7	-	20/21	1.05E-05	3
97	BTLA	BTLA	-	20/21	1.05E-05	3
98	SIGLEC5	SIGLEC5	-	20/21	1.05E-05	3
99	CD244	CD244	-	20/21	1.05E-05	3
100	KIR2DL2	KIR2DL2	-	20/21	1.05E-05	3
101	CD33	CD33	-	20/21	1.05E-05	3
102	PIRB	LILRB3	-	20/21	1.05E-05	3
103	PIRB	LOC107987462	-	20/21	1.05E-05	3
105	Siglec-E	SIGLEC9	-	20/21	1.05E-05	3
106	Siglec-10	SIGLEC10	-	20/21	1.05E-05	3
107	KIR3DL1	KIR3DL1	-	20/21	1.05E-05	3
108	LIRB		-	20/21	1.05E-05	3
110	NKG2A	KLRC1	-	20/21	1.05E-05	3
112	Fc gamma RII beta	FCGR2B	-	20/21	1.05E-05	3
116	LAIR1	LAIR1	-	20/21	1.05E-05	3
117	KIR2DL1	KIR2DL1	-	20/21	1.05E-05	3
118	ILT4	LILRB2	-	20/21	1.05E-05	3
104	CD84	CD84	-	20/21	1.05E-05	3
111	p16INK4	CDKN2A	-	20/21	1.05E-05	2
120	CBP	CREBBP	+	32/38	1.22E-05	3
119	miR-149-5p	MIR149	-	32/38	1.22E-05	3
122	Karyopherin alpha 3	KPNA3	+	25/28	1.37E-05	3
126	c-Jun	JUN	+	25/28	1.37E-05	3
128	C/EBPbeta	CEBPB	+	25/28	1.37E-05	2
125	MURC	CAVIN4	+	25/28	1.37E-05	3
123	CIRBP	CIRBP	+	25/28	1.37E-05	3
124	EAP30	SNF8	+	25/28	1.37E-05	3
127	Cyclin C	CCNC	+	25/28	1.37E-05	3
121	miR-502-5p	MIR502	-	25/28	1.37E-05	3
129	NPAT	NPAT	+	16/16	1.53E-05	3
130	NFAT-90	ILF3	+	16/16	1.53E-05	2
131	Rac1	RAC1	+	16/16	1.53E-05	2
132	MEF2D	MEF2D	+	27/31	1.70E-05	3
134	M-CSF receptor	CSF1R	+	27/31	1.70E-05	3
137	IFN-alpha/beta	IFNAR2	+	27/31	1.70E-05	3
138	IFN-alpha/beta	IFNAR1	+	27/31	1.70E-05	3
135	NIK(MAP3K14)	MAP3K14	+	27/31	1.70E-05	3
136	Paxillin	PXN	+	27/31	1.70E-05	3
133	miR-520c-3p	MIR520C	-	27/31	1.70E-05	3

147	STAT2	STAT2	+	22/24	1.79E-05	2
148	alpha-4/beta-1	ITGA4	+	22/24	1.79E-05	3
149	alpha-4/beta-1	ITGB1	+	22/24	1.79E-05	3
146	c-Src	SRC	+	22/24	1.79E-05	2
150	MSK1	RPS6KA5	+	22/24	1.79E-05	2
144	G-protein alpha-s	GNAS	+	22/24	1.79E-05	3
141	ANAPC1	ANAPC1	+	22/24	1.79E-05	3
145	PHAP1 (pp32)	ANP32A	+	22/24	1.79E-05	3
142	DDR1	DDR1	-	22/24	1.79E-05	3
139	NDPK B	NME1-NME2	-	22/24	1.79E-05	3
140	NDPK B	NME2	-	22/24	1.79E-05	3
143	APG5	ATG5	-	22/24	1.79E-05	3
151	MECOM	MECOM	-	29/34	1.93E-05	3
155	PAK1	PAK1	+	19/20	2.00E-05	2
156	TBK1	TBK1	+	19/20	2.00E-05	2
152	UBE1L	UBA7	+	19/20	2.00E-05	3
153	miR-330-5p	MIR330	-	19/20	2.00E-05	2
154	CTLA-4	CTLA4	-	19/20	2.00E-05	3
157	UTX	KDM6A	+	31/37	2.06E-05	3
165	Tyk2	TYK2	+	24/27	2.46E-05	3
158	D52	TPD52	+	24/27	2.46E-05	3
161	CAP1	CAP1	+	24/27	2.46E-05	3
162	CREB3	CREB3	-	24/27	2.46E-05	3
159	miR-1470	MIR1470	-	24/27	2.46E-05	3
160	miR-330-5p	MIR330	-	24/27	2.46E-05	3
163	miR-448-3p	MIR448	-	24/27	2.46E-05	3
164	PEDF (serpinF1)	SERPINF1	-	24/27	2.46E-05	3
169	c-Fos	FOS	+	26/30	2.97E-05	3
170	STAT2	STAT2	+	26/30	2.97E-05	3
167	COMMD1 (MURR1)	COMMD1	-	26/30	2.97E-05	3
168	PPAR-alpha	PPARA	-	26/30	2.97E-05	3
166	DET1	DET1	-	26/30	2.97E-05	3
171	PLC-beta2	PLCB2	+	15/15	3.05E-05	3
172	NBAT1	NBAT1	+	21/23	3.30E-05	3
178	alpha-IIb/beta-3	ITGA2B	+	21/23	3.30E-05	3
179	alpha-IIb/beta-3	ITGB3	+	21/23	3.30E-05	3
173	ZAK	MAP3K20	+	21/23	3.30E-05	3
175	OGG1	OGG1	+	21/23	3.30E-05	2
174	LYRIC	MTDH	+	21/23	3.30E-05	2
176	Nova1	NOVA1	+	21/23	3.30E-05	3
177	miR-542-3p	MIR542	-	21/23	3.30E-05	3
180	Connexin 43	GJA1	-	28/33	3.31E-05	3
181	FAK1	PTK2	+	30/36	3.48E-05	3
185	p38beta (MAPK11)	MAPK11	+	18/19	3.82E-05	2
182	PEAR1	PEAR1	+	18/19	3.82E-05	3
183	USP13	USP13	+	18/19	3.82E-05	2
184	Biglycan	BGN	+	18/19	3.82E-05	3
186	FKBP4	FKBP4	+	18/19	3.82E-05	2

187	Lnk	SH2B3	-	18/19	3.82E-05	3
188	Karyopherin alpha 2	KPNA2	+	23/26	4.40E-05	2
192	RMRP	RMRP	+	23/26	4.40E-05	3
189	CCR2	CCR2	+	23/26	4.40E-05	3
198	ACKR3	ACKR3	+	23/26	4.40E-05	3
195	4EHP	EIF4E2	+	23/26	4.40E-05	3
196	AF-9	MLLT3	+	23/26	4.40E-05	3
199	Cyclin T1	CCNT1	+	23/26	4.40E-05	3
191	miR-128-1-5p	MIR128-1	-	23/26	4.40E-05	3
193	mature miR-4429	MIR4429	-	23/26	4.40E-05	3
197	Csk	CSK	-	23/26	4.40E-05	3
190	EG624219	ANGPTL8	-	23/26	4.40E-05	3
194	CDK5RAP3	CDK5RAP3	-	23/26	4.40E-05	3
202	CC2D1A	CC2D1A	+	25/29	5.19E-05	3
204	TRIO	TRIO	+	25/29	5.19E-05	3
201	TGM4	TGM4	+	25/29	5.19E-05	3
200	Homer 3	HOMER3	-	25/29	5.19E-05	3
203	PPP4R1	PPP4R1	-	25/29	5.19E-05	3
205	NFKBIE	NFKBIE	-	25/29	5.19E-05	3
209	KLF5	KLF5	+	27/32	5.65E-05	3
210	SMAD1	SMAD1	+	27/32	5.65E-05	3
206	BRD4/NUT fusion		+	27/32	5.65E-05	3
207	EPC1	EPC1	+	27/32	5.65E-05	3
208	miR-766-5p	MIR766	-	27/32	5.65E-05	3
211	Protein kinase G1	PRKG1	+	29/35	5.84E-05	3
212	G-protein alpha-i2	GNAI2	+	20/22	6.06E-05	3
215	Migfilin	FBLIM1	+	20/22	6.06E-05	3
213	FXR	NR1H4	-	20/22	6.06E-05	2
214	SNX5	SNX5	-	20/22	6.06E-05	3
216	SMRT	NCOR2	-	20/22	6.06E-05	2
217	CDK9	CDK9	+	14/14	6.10E-05	2
231	RET	RET	+	17/18	7.25E-05	2
230	JAK2	JAK2	+	17/18	7.25E-05	2
232	Tyk2	TYK2	+	17/18	7.25E-05	2
226	SAD1 (USP39)	USP39	+	17/18	7.25E-05	2
222	CBP/P300	EP300	+	17/18	7.25E-05	2
223	CBP/P300	CREBBP	+	17/18	7.25E-05	2
225	RUVBL1	RUVBL1	+	17/18	7.25E-05	2
228	MUC1-CT	MUC1	+	17/18	7.25E-05	2
235	ISG15	ISG15	+	17/18	7.25E-05	2
236	IFRD1	IFRD1	+	17/18	7.25E-05	2
227	COMMD1 (MURR1)	COMMD1	-	17/18	7.25E-05	2
219	microRNA 103-1	MIR103A1	-	17/18	7.25E-05	3
220	miR-450a-5p	MIR450A2	-	17/18	7.25E-05	2
221	miR-450a-5p	MIR450A1	-	17/18	7.25E-05	2
224	miR-520c-3p	MIR520C	-	17/18	7.25E-05	2
229	miR-146a-5p	MIR146A	-	17/18	7.25E-05	2
218	CLIM1	PDLIM1	-	17/18	7.25E-05	2

233	CDC20	CDC20	-	17/18	7.25E-05	2
234	FAF1	FAF1	-	17/18	7.25E-05	2
238	miR-378b	MIR378B	+	22/25	7.83E-05	3
240	Nucleophosmin	NPM1	+	22/25	7.83E-05	2
242	CNK1	CNKSR1	+	22/25	7.83E-05	3
237	miR-3607-5p	SNORD138	-	22/25	7.83E-05	3
239	Ankyrin-G	ANK3	-	22/25	7.83E-05	3
241	DAXX	DAXX	-	22/25	7.83E-05	2
243	Calcyclin	S100A6	-	22/25	7.83E-05	3
246	NP220	ZNF638	+	24/28	9.00E-05	3
258	NET1	NET1	+	24/28	9.00E-05	3
244	TFG	TFG	+	24/28	9.00E-05	3
250	alpha-V/beta-5	ITGB5	+	24/28	9.00E-05	3
251	alpha-V/beta-5	ITGAV	+	24/28	9.00E-05	3
256	gp130	IL6ST	+	24/28	9.00E-05	3
253	BRD9	BRD9	+	24/28	9.00E-05	3
249	MIB1	MIB1	+	24/28	9.00E-05	3
247	RANBP7	IPO7	-	24/28	9.00E-05	3
245	miR-26b-3p	MIR26B	-	24/28	9.00E-05	3
248	miR-520b-3p	MIR520B	-	24/28	9.00E-05	3
252	miR-520d-5p	MIR520D	-	24/28	9.00E-05	3
254	CtBP1	CTBP1	-	24/28	9.00E-05	3
255	GANP	MCM3AP	-	24/28	9.00E-05	3
257	CtBP		-	24/28	9.00E-05	3
262	NF-AT1(NFATC2)	NFATC2	+	26/31	9.61E-05	3
261	Rac3	RAC3	+	26/31	9.61E-05	3
263	p90RSK2(RPS6KA3	RPS6KA3	+	26/31	9.61E-05	3
259	miR-300-3p	MIR300	-	26/31	9.61E-05	3
260	miR-33a-3p	MIR33A	-	26/31	9.61E-05	3
264	C/EBPbeta	CEBPB	+	28/34	9.76E-05	3
272	Pim-1	PIM1	+	19/21	1.11E-04	2
273	TPL2(MAP3K8)	MAP3K8	+	19/21	1.11E-04	2
274	p90RSK3(RPS6KA2	RPS6KA2	+	19/21	1.11E-04	3
269	G-protein alpha-12	GNA12	+	19/21	1.11E-04	3
267	ANKRD17	ANKRD17	+	19/21	1.11E-04	3
265	SNFT	BATF3	-	19/21	1.11E-04	3
271	PPAR-alpha	PPARA	-	19/21	1.11E-04	2
268	XIST	XIST	-	19/21	1.11E-04	2
266	BRMS1	BRMS1	-	19/21	1.11E-04	2
270	DTX4	DTX4	-	19/21	1.11E-04	3
276	ATP7A	ATP7A	+	13/13	1.22E-04	3
277	RPRD1B	RPRD1B	+	13/13	1.22E-04	2
278	HSPC142	BABAM1	+	13/13	1.22E-04	3
275	MBL2	MBL2	-	13/13	1.22E-04	3
279	PDLIM2	PDLIM2	-	13/13	1.22E-04	2
296	Fra-1	FOSL1	+	16/17	1.37E-04	3
285	MIR31HG	MIR31HG	+	16/17	1.37E-04	2
288	FGFR3	FGFR3	+	16/17	1.37E-04	2

280	A2M	A2M	+	16/17	1.37E-04	3
290	IL-6 receptor	IL6R	+	16/17	1.37E-04	2
291	IL-6 receptor	IL6ST	+	16/17	1.37E-04	2
293	gp130	IL6ST	+	16/17	1.37E-04	2
294	IFN-alpha/beta	IFNAR2	+	16/17	1.37E-04	2
295	IFN-alpha/beta	IFNAR1	+	16/17	1.37E-04	2
301	IL-22 receptor	IL10RB	+	16/17	1.37E-04	2
302	IL-22 receptor	IL22RA1	+	16/17	1.37E-04	2
292	Syk	SYK	+	16/17	1.37E-04	2
300	IRAK1	IRAK1	+	16/17	1.37E-04	2
297	PTAFR	PTAFR	+	16/17	1.37E-04	3
281	PSMC3	PSMC3	+	16/17	1.37E-04	2
284	RIG-I	DDX58	+	16/17	1.37E-04	2
286	ANAPC4	ANAPC4	+	16/17	1.37E-04	3
287	RIG-G	IFIT3	+	16/17	1.37E-04	2
298	CUX1	CUX1	-	16/17	1.37E-04	2
289	FasL(TNFSF6)	FASLG	-	16/17	1.37E-04	3
282	PTPN2	PTPN2	-	16/17	1.37E-04	2
299	G-protein alpha-i1	GNAI1	-	16/17	1.37E-04	3
283	SLAP	SLA	-	16/17	1.37E-04	3
303	NFKBIE	NFKBIE	-	16/17	1.37E-04	2
304	HOXB-AS1	HOXB-AS1	+	21/24	1.39E-04	3
313	Cardiotrophin-1	CTF1	+	21/24	1.39E-04	3
309	GFRalpha1	GFRA1	+	21/24	1.39E-04	3
310	IL-3 receptor	CSF2RB	+	21/24	1.39E-04	3
311	IL-3 receptor	IL3RA	+	21/24	1.39E-04	3
308	Calmyrin	CIB1	+	21/24	1.39E-04	3
312	NCAM1	NCAM1	+	21/24	1.39E-04	3
305	NKRF	NKRF	-	21/24	1.39E-04	3
306	CHFR	CHFR	-	21/24	1.39E-04	3
307	CPEB3	CPEB3	-	21/24	1.39E-04	3
318	IL-23 receptor	IL23R	+	23/27	1.55E-04	3
319	IL-23 receptor	IL12RB1	+	23/27	1.55E-04	3
321	FAK1	PTK2	+	23/27	1.55E-04	2
322	SGK1	SGK1	+	23/27	1.55E-04	3
314	eIF3S3	EIF3H	+	23/27	1.55E-04	3
316	SLM-2	KHDRBS3	+	23/27	1.55E-04	3
315	miR-3173-3p	MIR3173	-	23/27	1.55E-04	3
317	TRIM35	TRIM35	-	23/27	1.55E-04	3
320	PDLIM2	PDLIM2	-	23/27	1.55E-04	3
323	PRMT6	PRMT6	+	29/36	1.56E-04	3
324	OGG1	OGG1	+	29/36	1.56E-04	3
326	ERG	ERG	+	27/33	1.62E-04	3
325	ABL2	ABL2	+	27/33	1.62E-04	3
327	Karyopherin alpha 1	KPNA1	+	25/30	1.63E-04	3
333	VEGFR-1	FLT1	+	25/30	1.63E-04	3
328	NDR1 (STK38)	STK38	+	25/30	1.63E-04	3
331	CaMK IV	CAMK4	+	25/30	1.63E-04	3

329	Semenogelin I	SEMG1	+	25/30	1.63E-04	3
330	DPF3	DPF3	+	25/30	1.63E-04	3
334	GATA-2	GATA2	-	25/30	1.63E-04	3
332	I-kB		-	25/30	1.63E-04	3
335	KCTD20	KCTD20	+	18/20	2.01E-04	3
343	Karyopherin alpha 1	KPNA1	+	18/20	2.01E-04	2
360	IRF9	IRF9	+	18/20	2.01E-04	2
339	TSLPR	CRLF2	+	18/20	2.01E-04	3
340	TSLPR	IL7R	+	18/20	2.01E-04	3
341	OSM receptor	IL6ST	+	18/20	2.01E-04	3
342	OSM receptor	OSMR	+	18/20	2.01E-04	3
350	Fc gamma RI	FCGR1A	+	18/20	2.01E-04	3
351	CD79 complex	CD79A	+	18/20	2.01E-04	3
352	CD79 complex	CD79B	+	18/20	2.01E-04	3
355	IL-7 receptor	IL2RG	+	18/20	2.01E-04	3
356	IL-7 receptor	IL7R	+	18/20	2.01E-04	3
357	Fc epsilon RI	FCER1A	+	18/20	2.01E-04	3
358	Fc epsilon RI	MS4A2	+	18/20	2.01E-04	3
359	Fc epsilon RI	FCER1G	+	18/20	2.01E-04	3
362	IL-2R beta chain	IL2RB	+	18/20	2.01E-04	3
363	SGK1	SGK1	+	18/20	2.01E-04	2
344	MTCP1(p13)	MTCP1	+	18/20	2.01E-04	3
345	CLIP3	CLIP3	+	18/20	2.01E-04	3
336	HTR2A	HTR2A	+	18/20	2.01E-04	3
338	SMC1	SMC1A	+	18/20	2.01E-04	3
348	TCL1b	TCL1B	+	18/20	2.01E-04	3
361	Esrra	ESRRA	-	18/20	2.01E-04	3
337	miR-520b-3p	MIR520B	-	18/20	2.01E-04	2
353	miR-211-3p	MIR211	-	18/20	2.01E-04	3
346	KDM2A	KDM2A	-	18/20	2.01E-04	2
347	APG12	ATG12	-	18/20	2.01E-04	3
349	CD63	CD63	-	18/20	2.01E-04	3
354	FBXL7	FBXL7	-	18/20	2.01E-04	3
380	TLR6	TLR6	+	12/12	2.44E-04	3
377	p90Rsk		+	20/23	2.44E-04	3
378	JNK2(MAPK9)	MAPK9	+	20/23	2.44E-04	2
367	BRD9	BRD9	+	12/12	2.44E-04	2
372	mGluR5	GRM5	+	20/23	2.44E-04	3
369	STING (TMEM173)	STING1	+	12/12	2.44E-04	2
370	XAF1	XAF1	+	20/23	2.44E-04	2
374	NFKBID	NFKBID	+	20/23	2.44E-04	3
379	FKBP5	FKBP5	+	12/12	2.44E-04	2
364	miR-1299	MIR1299	-	12/12	2.44E-04	2
376	FGFR4	FGFR4	-	20/23	2.44E-04	3
368	Chymase	CMA1	-	12/12	2.44E-04	3
373	PLC-beta3	PLCB3	-	20/23	2.44E-04	3
365	MGEA5	OGA	-	20/23	2.44E-04	3
375	UBE3C	UBE3C	-	20/23	2.44E-04	3

366	SHARP (SPEN)	SPEN	-	20/23	2.44E-04	3
371	IRAS	NISCH	-	20/23	2.44E-04	3
382	IKK-alpha	CHUK	+	28/35	2.54E-04	3
381	DACH1	DACH1	-	28/35	2.54E-04	3
383	UBF	UBTF	+	15/16	2.59E-04	3
399	KLF6	KLF6	+	15/16	2.59E-04	2
404	CREB1	CREB1	+	15/16	2.59E-04	2
409	STAT1	STAT1	+	15/16	2.59E-04	1
410	Tyro3	TYRO3	+	15/16	2.59E-04	3
403	CNTF	CNTF	+	15/16	2.59E-04	3
415	LIF	LIF	+	15/16	2.59E-04	3
397	Plexin A2	PLXNA2	+	15/16	2.59E-04	3
413	IL6RA	IL6R	+	15/16	2.59E-04	3
414	IL4RA	IL4R	+	15/16	2.59E-04	3
384	USP17	USP17L26	+	15/16	2.59E-04	3
385	USP17	USP17L24	+	15/16	2.59E-04	3
386	USP17	USP17L30	+	15/16	2.59E-04	3
387	USP17	USP17L25	+	15/16	2.59E-04	3
388	USP17	USP17L29	+	15/16	2.59E-04	3
389	USP17	USP17L27	+	15/16	2.59E-04	3
390	USP17	USP17L28	+	15/16	2.59E-04	3
395	G-protein alpha-13	GNA13	+	15/16	2.59E-04	3
394	PRDX1	PRDX1	+	15/16	2.59E-04	2
393	D52	TPD52	+	15/16	2.59E-04	2
400	PSTPIP2	PSTPIP2	+	15/16	2.59E-04	2
401	DPF3	DPF3	+	15/16	2.59E-04	2
402	CD3 zeta	CD247	+	15/16	2.59E-04	3
405	DAP12	TYROBP	+	15/16	2.59E-04	3
407	PHAP1 (pp32)	ANP32A	+	15/16	2.59E-04	2
412	DAB1	DAB1	+	15/16	2.59E-04	3
396	FOXP3	FOXP3	-	15/16	2.59E-04	2
411	NF-kB2 (p100)	NFKB2	-	15/16	2.59E-04	2
408	SHP-2	PTPN11	-	15/16	2.59E-04	2
391	IRAKM	IRAK3	-	15/16	2.59E-04	3
392	SMURF1	SMURF1	-	15/16	2.59E-04	2
398	NFKBIB	NFKBIB	-	15/16	2.59E-04	2
406	I-kB		-	15/16	2.59E-04	2
420	alpha-V/beta-1	ITGAV	+	22/26	2.67E-04	3
421	alpha-V/beta-1	ITGB1	+	22/26	2.67E-04	3
430	ITGB8	ITGB8	+	22/26	2.67E-04	3
434	Dcc	DCC	+	22/26	2.67E-04	3
435	IL-10 receptor	IL10RB	+	22/26	2.67E-04	3
436	IL-10 receptor	IL10RA	+	22/26	2.67E-04	3
416	GAK	GAK	+	22/26	2.67E-04	3
429	SFK		+	22/26	2.67E-04	3
423	BAP1	BAP1	+	22/26	2.67E-04	3
432	PHF20	PHF20	+	22/26	2.67E-04	3
437	HMG14	HMGN1	+	22/26	2.67E-04	3

418	miR-130b-3p	MIR130B	-	22/26	2.67E-04	2
425	miR-551a	MIR551A	-	22/26	2.67E-04	3
426	miR-548x-3p	MIR548X2	-	22/26	2.67E-04	3
427	miR-548x-3p	MIR548X	-	22/26	2.67E-04	3
428	miR-22-5p	MIR22	-	22/26	2.67E-04	3
433	miR-129-1-3p	MIR129-1	-	22/26	2.67E-04	3
422	KIR2DL4	KIR2DL4	-	22/26	2.67E-04	3
431	QIK	SIK2	-	22/26	2.67E-04	3
424	Granzyme M	GZMM	-	22/26	2.67E-04	3
417	M33	CBX2	-	22/26	2.67E-04	3
419	KIF26A	KIF26A	-	22/26	2.67E-04	3
442	TPL2(MAP3K8)	MAP3K8	+	26/32	2.68E-04	3
439	Usp24	USP24	+	26/32	2.68E-04	3
438	Bcl-6	BCL6	-	26/32	2.68E-04	3
441	miR-297	MIR297	-	26/32	2.68E-04	3
440	IKBZ	NFKBIZ	-	26/32	2.68E-04	3
444	PELP1	PELP1	+	24/29	2.73E-04	3
453	c-Rel (NF-kB	REL	+	24/29	2.73E-04	3
451	c-Kit	KIT	+	24/29	2.73E-04	3
452	CD69	CD69	+	24/29	2.73E-04	3
447	MOZ	KAT6A	+	24/29	2.73E-04	3
446	CPEB4	CPEB4	+	24/29	2.73E-04	3
448	CRSP8 (CRSP34)	MED27	+	24/29	2.73E-04	3
443	HIPK1	HIPK1	-	24/29	2.73E-04	3
445	UACA	UACA	-	24/29	2.73E-04	3
449	TRIM26	TRIM26	-	24/29	2.73E-04	3
450	Par-4	PAWR	-	24/29	2.73E-04	3
458	Glycoprotein VI	GP6	+	17/19	3.64E-04	3
461	Fc epsilon RI beta	MS4A2	+	17/19	3.64E-04	3
468	alpha-6/beta-4	ITGA6	+	17/19	3.64E-04	3
469	alpha-6/beta-4	ITGB4	+	17/19	3.64E-04	3
454	CaMK II gamma	CAMK2G	+	17/19	3.64E-04	2
464	CaMK IV	CAMK4	+	17/19	3.64E-04	2
465	JAK1	JAK1	+	17/19	3.64E-04	2
466	NIK(MAP3K14)	MAP3K14	+	17/19	3.64E-04	2
455	Unc-119	UNC119	+	17/19	3.64E-04	3
460	SAP	SH2D1A	+	17/19	3.64E-04	3
462	Thy-1	THY1	+	17/19	3.64E-04	3
456	Menin	MEN1	-	17/19	3.64E-04	2
457	miR-224-3p	MIR224	-	17/19	3.64E-04	3
459	IKBZ	NFKBIZ	-	17/19	3.64E-04	2
463	Cullin 2	CUL2	-	17/19	3.64E-04	2
467	E-cadherin	CDH1	-	17/19	3.64E-04	2
472	CDK9	CDK9	+	27/34	4.11E-04	3
471	RBM8 (Y14)	RBM8A	+	27/34	4.11E-04	3
470	XIST	XIST	-	27/34	4.11E-04	3
477	MOZ	KAT6A	+	19/22	4.28E-04	2
476	Ephrin-B2 (CTF2)	EFNB2	+	19/22	4.28E-04	3

479	GFI-1	GFI1	-	19/22	4.28E-04	2
473	miR-7-5p	MIR7-1	-	19/22	4.28E-04	2
474	miR-7-5p	MIR7-2	-	19/22	4.28E-04	2
475	miR-7-5p	MIR7-3	-	19/22	4.28E-04	2
478	ING4	ING4	-	19/22	4.28E-04	2
480	Sequestosome	SQSTM1	-	19/22	4.28E-04	2
485	NR2E3	NR2E3	+	25/31	4.39E-04	3
487	p38alpha (MAPK14)	MAPK14	+	25/31	4.39E-04	3
488	Cyclophilin A	PPIA	+	25/31	4.39E-04	3
486	Nucleophosmin	NPM1	+	25/31	4.39E-04	3
481	NF-kB1 (p105)	NFKB1	-	25/31	4.39E-04	3
482	miR-512-5p	MIR512-2	-	25/31	4.39E-04	3
483	miR-512-5p	MIR512-1	-	25/31	4.39E-04	3
484	miR-653-5p	MIR653	-	25/31	4.39E-04	3
503	GABP alpha	GABPA	+	21/25	4.55E-04	3
495	CENTA2	ADAP2	+	21/25	4.55E-04	3
502	ECT2	ECT2	+	21/25	4.55E-04	3
492	LRP1	LRP1	+	21/25	4.55E-04	3
498	IL-4R type I	IL4R	+	21/25	4.55E-04	3
499	IL-4R type I	IL2RG	+	21/25	4.55E-04	3
501	ADAM12	ADAM12	+	21/25	4.55E-04	3
500	PKM2	PKM	+	21/25	4.55E-04	2
489	IWS1	IWS1	+	21/25	4.55E-04	3
493	METTL3	METTL3	+	21/25	4.55E-04	2
494	SFRS3	SRSF3	+	21/25	4.55E-04	3
497	PBXIP1	PBXIP1	+	21/25	4.55E-04	3
490	miR-1298-5p	MIR1298	-	21/25	4.55E-04	3
491	miR-3188	MIR3188	-	21/25	4.55E-04	3
496	RPL11	RPL11	-	21/25	4.55E-04	3
505	AF-10	MLLT10	+	23/28	4.56E-04	3
504	Calpain 3	CAPN3	+	23/28	4.56E-04	3
507	Bid	BID	+	23/28	4.56E-04	3
508	MATK	MATK	-	23/28	4.56E-04	3
506	Adenosine A2b	ADORA2B	-	23/28	4.56E-04	3
510	DEAF	DEAF1	+	11/11	4.88E-04	2
534	RelA (p65 NF-kB	RELA	+	14/15	4.88E-04	1
530	DOCK1	DOCK1	+	14/15	4.88E-04	3
526	ErbB4	ERBB4	+	14/15	4.88E-04	2
532	ActRIIA	ACVR2A	+	14/15	4.88E-04	3
533	PDGF-R-beta	PDGFRB	+	14/15	4.88E-04	2
517	FGF21	FGF21	+	14/15	4.88E-04	3
521	MGF	KITLG	+	14/15	4.88E-04	3
527	IKK (cat)	IKBKB	+	14/15	4.88E-04	2
528	IKK (cat)	CHUK	+	14/15	4.88E-04	2
535	AGTR1	AGTR1	+	14/15	4.88E-04	2
516	BCDIN3D	BCDIN3D	+	14/15	4.88E-04	3
518	Polycystin	PKD1	+	14/15	4.88E-04	2
509	Alpha-actinin 4	ACTN4	+	14/15	4.88E-04	2

511	MFHAS1	MFHAS1	+	14/15	4.88E-04	3
513	TESC	TESC	+	14/15	4.88E-04	2
520	CRSP8 (CRSP34)	MED27	+	14/15	4.88E-04	2
523	TRIF (TICAM1)	TICAM1	+	11/11	4.88E-04	2
512	CNOT7	CNOT7	-	14/15	4.88E-04	2
519	miR-483-3p	MIR483	-	14/15	4.88E-04	2
525	Adenosine A2b	ADORA2B	-	14/15	4.88E-04	2
529	Dopamine D1A	DRD1	-	14/15	4.88E-04	3
524	UBE3C	UBE3C	-	11/11	4.88E-04	2
514	UACA	UACA	-	14/15	4.88E-04	2
515	CCDC6	CCDC6	-	14/15	4.88E-04	3
522	Par-4	PAWR	-	14/15	4.88E-04	2
531	Cullin 1	CUL1	-	11/11	4.88E-04	2
536	STAT1	STAT1	+	28/36	5.97E-04	3
543	TAF4B	TAF4B	+	16/18	6.56E-04	2
545	RelB (NF-kB subunit)	RELB	+	16/18	6.56E-04	2
538	miR-514b-5p	MIR514B	+	16/18	6.56E-04	3
541	LINC00277	EWSAT1	+	16/18	6.56E-04	3
546	PLD2	PLD2	+	16/18	6.56E-04	3
544	ADAM17	ADAM17	+	16/18	6.56E-04	3
539	CNOT3	CNOT3	+	16/18	6.56E-04	3
542	TRIP6	TRIP6	+	16/18	6.56E-04	2
540	miR-373-3p	MIR373	-	16/18	6.56E-04	2
537	Myosin VI	MYO6	-	16/18	6.56E-04	3
552	Zac1	PLAGL1	+	26/33	6.59E-04	3
549	CASC9	CASC9	+	26/33	6.59E-04	3
550	IL-6 receptor	IL6R	+	26/33	6.59E-04	3
551	IL-6 receptor	IL6ST	+	26/33	6.59E-04	3
547	miR-1299	MIR1299	-	26/33	6.59E-04	3
548	miR-345-5p	MIR345	-	26/33	6.59E-04	3
553	miR-185-3p	MIR185	-	24/30	7.16E-04	3
554	Sequestosome	SQSTM1	-	24/30	7.16E-04	3
556	Syndecan-3	SDC3	+	18/21	7.45E-04	3
560	IL-2 receptor	IL2RB	+	18/21	7.45E-04	3
561	IL-2 receptor	IL2RA	+	18/21	7.45E-04	3
562	IL-2 receptor	IL2RG	+	18/21	7.45E-04	3
564	STRA6	STRA6	+	18/21	7.45E-04	3
565	PLC-gamma 1	PLCG1	+	18/21	7.45E-04	3
555	HSP70L1	HSPA14	+	18/21	7.45E-04	3
557	miR-412-5p	MIR412	-	18/21	7.45E-04	3
558	miR-539-3p	MIR539	-	18/21	7.45E-04	3
559	miR-1225-5p	MIR1225	-	18/21	7.45E-04	3
563	PTPN12	PTPN12	-	18/21	7.45E-04	3
575	SOX11	SOX11	+	22/27	7.57E-04	3
580	TWIST1	TWIST1	+	22/27	7.57E-04	3
570	HB-EGF	HBEGF	+	22/27	7.57E-04	3
566	FAM83B	FAM83B	+	22/27	7.57E-04	3
567	C6orf176	LINC00473	+	22/27	7.57E-04	3

568	UCHL3	UCHL3	+	22/27	7.57E-04	3
572	DUB3	USP17L2	+	22/27	7.57E-04	3
582	NR1	GRIN1	+	22/27	7.57E-04	3
576	PSTPIP2	PSTPIP2	+	22/27	7.57E-04	3
578	TRIP6	TRIP6	+	22/27	7.57E-04	3
581	IFRD1	IFRD1	+	22/27	7.57E-04	3
573	miR-3140-3p	MIR3140	-	22/27	7.57E-04	3
574	miR-1266-5p	MIR1266	-	22/27	7.57E-04	3
577	miR-496-3p	MIR496	-	22/27	7.57E-04	3
579	PIAS3	PIAS3	-	22/27	7.57E-04	2
569	VPS11	VPS11	-	22/27	7.57E-04	3
571	VPS18	VPS18	-	22/27	7.57E-04	3
592	IRF9	IRF9	+	20/24	7.72E-04	3
583	ZFPM2-AS1	ZFPM2-AS1	+	20/24	7.72E-04	3
587	miR-219-1-3p	MIR219A1	+	20/24	7.72E-04	3
591	cPKC (conventional)		+	20/24	7.72E-04	3
589	nAChR alpha-7	CHRFAM7A	+	20/24	7.72E-04	3
590	nAChR alpha-7	CHRNA7	+	20/24	7.72E-04	3
586	BRD4	BRD4	+	20/24	7.72E-04	2
584	PRMT5	PRMT5	+	20/24	7.72E-04	2
588	LRIG2	LRIG2	+	20/24	7.72E-04	3
585	miR-506-3p	MIR506	-	20/24	7.72E-04	2
594	CD36	CD36	+	13/14	9.16E-04	3
596	RhoE	RND3	+	13/14	9.16E-04	2
600	HSP60	HSPD1	+	13/14	9.16E-04	2
603	ZFP91	ZFP91	+	13/14	9.16E-04	2
605	HEY2	HEY2	-	13/14	9.16E-04	3
593	miR-26b-3p	MIR26B	-	13/14	9.16E-04	2
598	miR-146b-5p	MIR146B	-	13/14	9.16E-04	2
604	RhoB	RHOB	-	13/14	9.16E-04	3
597	PP2A cat (alpha)	PPP2CA	-	13/14	9.16E-04	2
599	PP2A cat (beta)	PPP2CB	-	13/14	9.16E-04	3
602	WIP1	PPM1D	-	13/14	9.16E-04	2
601	Cathepsin G	CTSG	-	13/14	9.16E-04	3
595	Ankyrin-B	ANK2	-	13/14	9.16E-04	3
607	Karyopherin alpha 4	KPNA4	+	27/35	9.39E-04	3
606	TORC2	CRTC2	+	27/35	9.39E-04	3
608	PIAS1	PIAS1	-	27/35	9.39E-04	3
611	BARX2	BARX2	+	10/10	9.77E-04	2
612	IRF4	IRF4	+	10/10	9.77E-04	2
613	HMGB1	HMGB1	+	10/10	9.77E-04	2
614	IFNAR2	IFNAR2	+	10/10	9.77E-04	2
609	EAP30	SNF8	+	10/10	9.77E-04	2
610	TLE4	TLE4	-	10/10	9.77E-04	3
620	RelA (p65 NF-kB	RELA	+	25/32	1.05E-03	3
621	IL-22 receptor	IL10RB	+	25/32	1.05E-03	3
622	IL-22 receptor	IL22RA1	+	25/32	1.05E-03	3
616	CBP/P300	EP300	+	25/32	1.05E-03	3

617	CBP/P300	CREBBP	+	25/32	1.05E-03	3
615	PTPN2	PTPN2	-	25/32	1.05E-03	3
618	RNF22	TRIM3	-	25/32	1.05E-03	3
619	SMRT	NCOR2	-	25/32	1.05E-03	3
628	RelB (NF-kB subunit)	RELB	+	23/29	1.16E-03	3
623	CaMK II gamma	CAMK2G	+	23/29	1.16E-03	3
624	ABIN-2	TNIP2	+	23/29	1.16E-03	3
625	miR-105-5p	MIR105-1	-	23/29	1.16E-03	3
626	miR-105-5p	MIR105-2	-	23/29	1.16E-03	3
627	ING4	ING4	-	23/29	1.16E-03	3
633	miR-3941	MIR3941	+	15/17	1.18E-03	3
629	FGF9	FGF9	+	15/17	1.18E-03	3
634	FGF17	FGF17	+	15/17	1.18E-03	3
635	FGF2	FGF2	+	15/17	1.18E-03	3
638	FGF8	FGF8	+	15/17	1.18E-03	3
632	Syndecan-1	SDC1	+	15/17	1.18E-03	3
637	Nectin-1	NECTIN1	+	15/17	1.18E-03	3
630	PCBP-2	PCBP2	+	15/17	1.18E-03	2
631	Klotho beta	KLB	+	15/17	1.18E-03	3
636	PPAR-gamma	PPARG	-	15/17	1.18E-03	2
657	JunD	JUND	+	21/26	1.25E-03	3
644	FEZF1-AS1	FEZF1-AS1	+	21/26	1.25E-03	3
646	KIAA1429	VIRMA	+	21/26	1.25E-03	3
656	USP46	USP46	+	21/26	1.25E-03	3
639	DDX1	DDX1	+	21/26	1.25E-03	3
647	RIG-I	DDX58	+	21/26	1.25E-03	3
640	DND1	DND1	+	21/26	1.25E-03	3
643	RAD1	RAD1	+	21/26	1.25E-03	3
645	hnRNP F	HNRNPF	+	21/26	1.25E-03	3
650	SNX8	SNX8	+	21/26	1.25E-03	3
654	Calnexin	CANX	+	21/26	1.25E-03	3
655	LAGY	HOPX	+	21/26	1.25E-03	3
641	miR-520e-3p	MIR520E	-	21/26	1.25E-03	3
653	miR-517c-3p	MIR517C	-	21/26	1.25E-03	3
651	CMIP	CMIP	-	21/26	1.25E-03	3
642	PDCD10	PDCD10	-	21/26	1.25E-03	3
648	BTG3	BTG3	-	21/26	1.25E-03	3
649	SOCS5	SOCS5	-	21/26	1.25E-03	3
652	NFKBIB	NFKBIB	-	21/26	1.25E-03	3
666	IL-9 receptor	IL2RG	+	17/20	1.29E-03	3
667	IL-9 receptor	IL9R	+	17/20	1.29E-03	3
670	IL-2R gamma chain	IL2RG	+	17/20	1.29E-03	3
665	RHEB2	RHEB	+	17/20	1.29E-03	3
660	C6orf106	ILRUN	+	17/20	1.29E-03	3
661	TSP50	PRSS50	+	17/20	1.29E-03	3
662	USP1	USP1	+	17/20	1.29E-03	2
669	Cyclophilin A	PPIA	+	17/20	1.29E-03	2
668	Aif	AIFM1	+	17/20	1.29E-03	3

663	miR-302a-3p	MIR302A	-	17/20	1.29E-03	2
664	miR-23a-3p	MIR23A	-	17/20	1.29E-03	2
659	PP2A cat (alpha)	PPP2CA	-	17/20	1.29E-03	3
658	LRG-47	IRGM	-	17/20	1.29E-03	3
671	TXNIP (VDUP1)	TXNIP	-	17/20	1.29E-03	2
686	ATF-7	ATF7	+	19/23	1.30E-03	3
677	Betacellulin	BTC	+	19/23	1.30E-03	3
682	Neuregulin 2	NRG2	+	19/23	1.30E-03	3
688	Epiregulin	EREG	+	19/23	1.30E-03	3
672	Dystroglycan	DAG1	+	19/23	1.30E-03	3
679	PAK		+	19/23	1.30E-03	3
680	BRSK2	BRSK2	+	19/23	1.30E-03	3
687	GOLPH2	GOLM1	+	19/23	1.30E-03	3
674	MAML2	MAML2	+	19/23	1.30E-03	3
684	MAP1	MOAP1	+	19/23	1.30E-03	3
673	miR-660-5p	MIR660	-	19/23	1.30E-03	3
675	miR-199a-5p	MIR199A1	-	19/23	1.30E-03	2
676	miR-199a-5p	MIR199A2	-	19/23	1.30E-03	2
678	miR-641	MIR641	-	19/23	1.30E-03	3
681	PSMD10 (Gankyrin)	PSMD10	-	19/23	1.30E-03	2
683	IRS-2	IRS2	-	19/23	1.30E-03	3
685	Keratin 18	KRT18	-	19/23	1.30E-03	3
689	Karyopherin alpha 2	KPNA2	+	26/34	1.47E-03	3
691	p90RSK1	RPS6KA1	+	24/31	1.66E-03	3
690	PSMC3	PSMC3	+	24/31	1.66E-03	3
694	miR-200b-5p	MIR200B	+	12/13	1.71E-03	3
695	LTBR(TNFRSF3)	LTBR	+	12/13	1.71E-03	3
693	BRD3	BRD3	+	12/13	1.71E-03	2
692	CRIP2	CRIP2	-	12/13	1.71E-03	2
700	KLF6	KLF6	+	22/28	1.86E-03	3
705	TBP	TBP	+	22/28	1.86E-03	3
703	IFN-gamma receptor	IFNGR1	+	22/28	1.86E-03	3
704	IFN-gamma receptor	IFNGR2	+	22/28	1.86E-03	3
706	alpha-5/beta-1	ITGB1	+	22/28	1.86E-03	3
707	alpha-5/beta-1	ITGA5	+	22/28	1.86E-03	3
698	SAD1 (USP39)	USP39	+	22/28	1.86E-03	3
701	MUC1-CT	MUC1	+	22/28	1.86E-03	3
696	miR-190-5p	MIR190A	-	22/28	1.86E-03	3
697	miR-1248	MIR1248	-	22/28	1.86E-03	3
699	miR-655-3p	MIR655	-	22/28	1.86E-03	3
702	miR-29b-1-5p	MIR29B1	-	22/28	1.86E-03	3
708	Karyopherin alpha 3	KPNA3	+	9/9	1.95E-03	2
712	ENL	MLLT1	+	9/9	1.95E-03	3
718	NF-AT4(NFATC3)	NFATC3	+	9/9	1.95E-03	2
719	IRF3	IRF3	+	9/9	1.95E-03	1
720	c-Rel (NF-kB	REL	+	9/9	1.95E-03	2
714	LINC02605	LINC02605	+	9/9	1.95E-03	2
709	SND1	SND1	+	9/9	1.95E-03	2

716	EPC1	EPC1	+	9/9	1.95E-03	2
710	miR-302d-3p	MIR302D	-	9/9	1.95E-03	2
717	miR-541-3p	MIR541	-	9/9	1.95E-03	2
711	QKI	QKI	-	9/9	1.95E-03	2
713	TRIM33	TRIM33	-	9/9	1.95E-03	2
715	Ubiquilin-1	UBQLN1	-	9/9	1.95E-03	3
734	SOX10	SOX10	+	20/25	2.04E-03	3
735	BAPX1	NKX3-2	+	20/25	2.04E-03	3
736	RelA (p65 NF-kB	RELA	+	20/25	2.04E-03	2
722	LINC00662	LINC00662	+	20/25	2.04E-03	3
728	FGF1	FGF1	+	20/25	2.04E-03	3
731	Syk	SYK	+	20/25	2.04E-03	3
730	Rad52	RAD52	+	20/25	2.04E-03	3
732	SH2B	SH2B1	+	20/25	2.04E-03	3
726	TAFII55	TAF7	-	20/25	2.04E-03	3
721	miR-4698	MIR4698	-	20/25	2.04E-03	3
723	miR-1323	MIR1323	-	20/25	2.04E-03	3
724	miR-29c-5p	MIR29C	-	20/25	2.04E-03	3
727	miR-27a-5p	MIR27A	-	20/25	2.04E-03	3
729	FasR(CD95)	FAS	-	20/25	2.04E-03	3
733	PIAS1	PIAS1	-	20/25	2.04E-03	2
725	LIG-1	LRIG1	-	20/25	2.04E-03	3
737	SMARCA5	SMARCA5	+	14/16	2.09E-03	3
754	TFIIB	GTF2B	+	14/16	2.09E-03	2
748	EYA4	EYA4	+	14/16	2.09E-03	3
751	PKA-cat beta	PRKACB	+	14/16	2.09E-03	2
753	Fyn	FYN	+	14/16	2.09E-03	2
745	OR3A4	OR3A4P	+	14/16	2.09E-03	3
741	NOTCH2 (2ICD)	NOTCH2	+	14/16	2.09E-03	2
742	CPSF4	CPSF4	+	14/16	2.09E-03	2
743	IRAK1BP1	IRAK1BP1	+	14/16	2.09E-03	2
746	CCM2	CCM2	+	14/16	2.09E-03	3
750	PHF20	PHF20	+	14/16	2.09E-03	2
738	miR-520e-3p	MIR520E	-	14/16	2.09E-03	2
744	miR-875-3p	MIR875	-	14/16	2.09E-03	3
749	miR-372-3p	MIR372	-	14/16	2.09E-03	2
752	miR-148a-3p	MIR148A	-	14/16	2.09E-03	2
747	PPP2R2B	PPP2R2B	-	14/16	2.09E-03	3
739	Lysyl oxidase	LOX	-	14/16	2.09E-03	3
740	CHFR	CHFR	-	14/16	2.09E-03	2
757	NF-kB1 (p50)	NFKB1	+	18/22	2.17E-03	2
762	AKT1	AKT1	+	18/22	2.17E-03	2
763	IKK-beta	IKBKB	+	18/22	2.17E-03	2
759	WHSC1	NSD2	+	18/22	2.17E-03	2
755	MCA2(p38)	AIMP2	+	18/22	2.17E-03	3
758	STING (TMEM173)	STING1	+	18/22	2.17E-03	3
756	miR-564	MIR564	-	18/22	2.17E-03	3
760	YTHDF2	YTHDF2	-	18/22	2.17E-03	2

761	GBP1	GBP1	-	18/22	2.17E-03	3
765	miR-210-5p	MIR210	+	16/19	2.21E-03	3
772	miR-1261	MIR1261	+	16/19	2.21E-03	3
774	DOCK8	DOCK8	+	16/19	2.21E-03	3
764	Rab-1A	RAB1A	+	16/19	2.21E-03	3
768	AZI2	AZI2	+	16/19	2.21E-03	3
767	DEP-1	PTPRJ	+	16/19	2.21E-03	3
773	LARS1	LARS1	+	16/19	2.21E-03	3
766	OTUD4	OTUD4	+	16/19	2.21E-03	3
769	RGC32	RGCC	+	16/19	2.21E-03	2
770	Flotillin-2	FLOT2	+	16/19	2.21E-03	3
771	P52rIPK	THAP12	+	16/19	2.21E-03	3
775	Nod2 (CARD15)	NOD2	+	16/19	2.21E-03	3
776	SPRY4-IT1	SPRY4-IT1	+	25/33	2.28E-03	3
777	FGFR3	FGFR3	+	25/33	2.28E-03	3
783	Ran	RAN	+	23/30	2.61E-03	3
781	PKA-cat beta	PRKACB	+	23/30	2.61E-03	3
784	WDR5	WDR5	+	23/30	2.61E-03	3
780	Polycystin	PKD1	+	23/30	2.61E-03	3
782	Pyrin (MEFV)	MEFV	+	23/30	2.61E-03	3
778	miR-493-5p	MIR493	-	23/30	2.61E-03	3
779	miR-302a-3p	MIR302A	-	23/30	2.61E-03	3
795	NFAT-90	ILF3	+	21/27	2.96E-03	3
798	TFIIB	GTF2B	+	21/27	2.96E-03	3
787	miR-3134	MIR3134	+	21/27	2.96E-03	3
807	TNF-alpha	TNF	+	21/27	2.96E-03	3
799	alpha-3/beta-1	ITGB1	+	21/27	2.96E-03	3
800	alpha-3/beta-1	ITGA3	+	21/27	2.96E-03	3
801	alpha-8/beta-1	ITGA8	+	21/27	2.96E-03	3
802	alpha-8/beta-1	ITGB1	+	21/27	2.96E-03	3
803	alpha-10/beta-1	ITGB1	+	21/27	2.96E-03	3
804	alpha-10/beta-1	ITGA10	+	21/27	2.96E-03	3
805	alpha-11/beta-1	ITGA11	+	21/27	2.96E-03	3
806	alpha-11/beta-1	ITGB1	+	21/27	2.96E-03	3
797	PAK1	PAK1	+	21/27	2.96E-03	3
785	Protein p8	NUPR1	+	21/27	2.96E-03	3
788	IRAK1BP1	IRAK1BP1	+	21/27	2.96E-03	3
790	VISA	MAVS	+	21/27	2.96E-03	3
789	PL scramblase 1	PLSCR1	-	21/27	2.96E-03	3
791	FOXP3	FOXP3	-	21/27	2.96E-03	3
794	PAX6	PAX6	-	21/27	2.96E-03	3
796	RRAD	RRAD	-	21/27	2.96E-03	3
792	KDM2A	KDM2A	-	21/27	2.96E-03	3
793	ALKBH5	ALKBH5	-	21/27	2.96E-03	3
786	GNIP (TRIM7)	TRIM7	-	21/27	2.96E-03	3
808	IEX1	IER3	-	21/27	2.96E-03	3
810	MIR31HG	MIR31HG	+	26/35	2.99E-03	3
809	METTL3	METTL3	+	26/35	2.99E-03	3

811	PSMD10 (Gankyrin)	PSMD10	-	26/35	2.99E-03	3
814	MKL1	MRTFA	+	11/12	3.17E-03	2
815	DAPK1	DAPK1	+	11/12	3.17E-03	2
812	PAX7	PAX7	-	11/12	3.17E-03	2
813	miR-141-5p	MIR141	-	11/12	3.17E-03	3
817	Karyopherin alpha 4	KPNA4	+	19/24	3.31E-03	2
818	miR-605-5p	MIR605	+	19/24	3.31E-03	3
820	APP	APP	+	19/24	3.31E-03	3
816	CaMKK1	CAMKK1	+	19/24	3.31E-03	3
821	WT1	WT1	-	19/24	3.31E-03	3
819	miR-96-3p	MIR96	-	19/24	3.31E-03	3
824	YTHDF1	YTHDF1	+	24/32	3.50E-03	3
822	HBXAP	RSF1	+	24/32	3.50E-03	3
823	RBM35A	ESRP1	-	24/32	3.50E-03	3
832	LOC101929517	ETS1-AS1	+	17/21	3.60E-03	3
837	Ephrin-B receptor 1	EPHB1	+	17/21	3.60E-03	3
834	MIF	MIF	+	17/21	3.60E-03	2
836	PDGF-B	PDGFB	+	17/21	3.60E-03	3
827	WRCH-1	RHOU	+	17/21	3.60E-03	3
830	STRAD	STRADA	+	17/21	3.60E-03	3
835	SAM68	KHDRBS1	+	17/21	3.60E-03	2
828	DSIPI (GILZ)	TSC22D3	-	17/21	3.60E-03	2
826	miR-4728-5p	MIR4728	-	17/21	3.60E-03	3
833	miR-615-5p	MIR615	-	17/21	3.60E-03	3
829	LGR4	LGR4	-	17/21	3.60E-03	3
831	DDX19L	DDX19A	-	17/21	3.60E-03	3
825	RAI	RNH1	-	17/21	3.60E-03	3
841	Importin 9	IPO9	+	13/15	3.69E-03	3
838	ELF3	ELF3	+	13/15	3.69E-03	2
844	UFO	AXL	+	13/15	3.69E-03	3
845	HPK1(MAP4K1)	MAP4K1	+	13/15	3.69E-03	3
839	PGAM1	PGAM1	+	13/15	3.69E-03	3
847	CNR1	CNR1	+	13/15	3.69E-03	3
846	RACK1	RACK1	+	13/15	3.69E-03	2
842	NRIF	ZNF274	-	13/15	3.69E-03	3
840	miR-520c-5p	MIR520C	-	13/15	3.69E-03	2
843	NRBP	NRBP1	-	13/15	3.69E-03	3
855	NF-AT5	NFAT5	+	15/18	3.77E-03	2
856	NF-AT2(NFATC1)	NFATC1	+	15/18	3.77E-03	2
854	mTOR	MTOR	+	15/18	3.77E-03	2
849	ART-27	UXT	+	15/18	3.77E-03	2
850	TIRAP (Mal)	TIRAP	+	15/18	3.77E-03	3
851	LARP1	LARP1	+	15/18	3.77E-03	3
852	miR-186-5p	MIR186	-	15/18	3.77E-03	2
853	Dexras1	RASD1	-	15/18	3.77E-03	3
857	Sortilin	SORT1	-	15/18	3.77E-03	3
848	Annexin V	ANXA5	-	15/18	3.77E-03	3
858	IRAK1	IRAK1	+	27/37	3.82E-03	3

862	ATF-5	ATF5	+	8/8	3.91E-03	2
864	STAT2	STAT2	+	8/8	3.91E-03	1
859	MEG3	MEG3	-	8/8	3.91E-03	2
863	miR-328-3p	MIR328	-	8/8	3.91E-03	2
860	DACT1	DACT1	-	8/8	3.91E-03	2
861	EID1	EID1	-	8/8	3.91E-03	2
868	LINC01138	LINC01138	+	22/29	4.07E-03	3
870	JNK3(MAPK10)	MAPK10	+	22/29	4.07E-03	3
866	BRD4	BRD4	+	22/29	4.07E-03	3
867	KLF11 (TIEG2)	KLF11	-	22/29	4.07E-03	3
865	miR-490-5p	MIR490	-	22/29	4.07E-03	3
869	DCTN2	DCTN2	-	22/29	4.07E-03	3
871	MYST1	KAT8	+	25/34	4.52E-03	3
872	XAF1	XAF1	+	25/34	4.52E-03	3
876	NRB54	NONO	+	20/26	4.68E-03	3
881	STAT1	STAT1	+	20/26	4.68E-03	2
884	MEF2C	MEF2C	+	20/26	4.68E-03	3
875	Ephrin-A receptor 4	EPHA4	+	20/26	4.68E-03	3
885	LPAR2	LPAR2	+	20/26	4.68E-03	3
886	AGTR1	AGTR1	+	20/26	4.68E-03	3
873	NOTCH2 (2ICD)	NOTCH2	+	20/26	4.68E-03	3
880	eIF3S5	EIF3F	+	20/26	4.68E-03	3
874	CNOT7	CNOT7	-	20/26	4.68E-03	3
883	NRF2	NFE2L2	-	20/26	4.68E-03	3
877	miR-320d	MIR320D1	-	20/26	4.68E-03	3
878	miR-320d	MIR320D2	-	20/26	4.68E-03	3
879	Casein kinase I	CSNK1G1	-	20/26	4.68E-03	3
882	CDC20	CDC20	-	20/26	4.68E-03	3
895	TLR2	TLR2	+	18/23	5.31E-03	3
889	MYST1	KAT8	+	18/23	5.31E-03	2
887	ASXL1	ASXL1	+	18/23	5.31E-03	3
888	RNF144	RNF144A	+	18/23	5.31E-03	3
891	NET1(TSPAN1)	TSPAN1	+	18/23	5.31E-03	3
892	TRAM	TICAM2	+	18/23	5.31E-03	3
893	MCT-1	MCTS1	+	18/23	5.31E-03	3
890	SHIP	INPP5D	-	18/23	5.31E-03	3
894	FKBP8	FKBP8	-	18/23	5.31E-03	3
896	JAK1	JAK1	+	23/31	5.34E-03	3
897	PCNA	PCNA	-	23/31	5.34E-03	3
898	IKK-beta	IKBKB	+	26/36	5.67E-03	3
902	IRF7	IRF7	+	10/11	5.86E-03	3
904	Nod1	NOD1	+	10/11	5.86E-03	3
905	TLR4	TLR4	+	10/11	5.86E-03	2
900	STK36	STK36	+	10/11	5.86E-03	3
901	ULK3	ULK3	+	10/11	5.86E-03	3
899	DRIP130	MED23	+	10/11	5.86E-03	2
903	RASA3	RASA3	-	10/11	5.86E-03	3
911	NRB54	NONO	+	16/20	5.91E-03	2

906	miR-330-3p	MIR330	+	16/20	5.91E-03	3
907	DR6(TNFRSF21)	TNFRSF21	+	16/20	5.91E-03	3
916	iNOS	NOS2	+	16/20	5.91E-03	3
908	DLEU1	DLEU1	+	16/20	5.91E-03	3
910	C1orf86	FAAP20	+	16/20	5.91E-03	3
912	BUB3	BUB3	+	16/20	5.91E-03	3
913	miR-4288	MIR4288	-	16/20	5.91E-03	3
914	TRAF7	TRAF7	-	16/20	5.91E-03	2
915	Elongin C	ELOC	-	16/20	5.91E-03	3
909	NALP4	NLRP4	-	16/20	5.91E-03	3
917	Alpha crystallin B	CRYAB	-	16/20	5.91E-03	2
918	SOCS1	SOCS1	-	16/20	5.91E-03	2
926	GATA-4	GATA4	+	21/28	6.27E-03	3
927	NF-AT5	NFAT5	+	21/28	6.27E-03	3
924	IKK (cat)	IKBKB	+	21/28	6.27E-03	3
925	IKK (cat)	CHUK	+	21/28	6.27E-03	3
921	BVRA	BLVRA	+	21/28	6.27E-03	3
922	RPS3	RPS3	+	21/28	6.27E-03	3
923	TPT1	TPT1	+	21/28	6.27E-03	3
919	miR-935	MIR935	-	21/28	6.27E-03	3
920	PICT-1	NOP53	-	21/28	6.27E-03	3
928	IGBP1	IGBP1	-	21/28	6.27E-03	3
937	IRF4	IRF4	+	14/17	6.36E-03	3
934	LIFR	LIFR	+	14/17	6.36E-03	3
938	G-protein		+	14/17	6.36E-03	3
930	PRMT6	PRMT6	+	14/17	6.36E-03	2
936	ARD1	NAA10	+	14/17	6.36E-03	2
929	NF-kB1 (p105)	NFKB1	-	14/17	6.36E-03	2
935	KLF11 (TIEG2)	KLF11	-	14/17	6.36E-03	2
931	miR-99a-3p	MIR99A	-	14/17	6.36E-03	3
933	hsa-miR-4756-5p	MIR4756	-	14/17	6.36E-03	3
939	CD45	PTPRC	-	14/17	6.36E-03	3
932	TRIM72	TRIM72	-	14/17	6.36E-03	3
942	MCM5	MCM5	+	12/14	6.47E-03	2
944	HBXAP	RSF1	+	12/14	6.47E-03	2
945	TIF1-beta	TRIM28	-	12/14	6.47E-03	2
943	miR-192-3p	MIR192	-	12/14	6.47E-03	3
946	miR-302d-5p	MIR302D	-	12/14	6.47E-03	3
947	Caspase-3	CASP3	-	12/14	6.47E-03	2
940	HSPA1B	HSPA1A	-	12/14	6.47E-03	2
941	HSPA1B	HSPA1B	-	12/14	6.47E-03	2
948	SMYD3	SMYD3	+	24/33	6.77E-03	3
949	DAXX	DAXX	-	24/33	6.77E-03	3
955	NOTCH1 (NICD)	NOTCH1	+	19/25	7.32E-03	2
953	SPRED2	SPRED2	+	19/25	7.32E-03	3
957	Cyclin E	CCNE1	+	19/25	7.32E-03	3
950	miR-1285-3p	MIR1285-2	-	19/25	7.32E-03	3
951	miR-1285-3p	MIR1285-1	-	19/25	7.32E-03	3

952	miR-582-5p	MIR582	-	19/25	7.32E-03	3
954	miR-182-5p	MIR182	-	19/25	7.32E-03	2
956	miR-454-3p	MIR454	-	19/25	7.32E-03	3
960	NF-kB p65/p65	RELA	+	7/7	7.81E-03	2
962	NF-kB p50/p65	RELA	+	7/7	7.81E-03	2
963	NF-kB p50/p65	NFKB1	+	7/7	7.81E-03	2
965	p300	EP300	+	7/7	7.81E-03	1
964	MyD88	MYD88	+	7/7	7.81E-03	2
966	PEG3	PEG3	-	7/7	7.81E-03	2
961	miR-766-5p	MIR766	-	7/7	7.81E-03	2
958	CORO1B	CORO1B	-	7/7	7.81E-03	3
959	TRIM35	TRIM35	-	7/7	7.81E-03	2
967	Myocardin	MYOCD	+	22/30	8.06E-03	3
970	MIF	MIF	+	22/30	8.06E-03	3
968	Pim-2	PIM2	+	22/30	8.06E-03	3
969	miR-let-7g-3p	MIRLET7G	-	22/30	8.06E-03	3
971	ISG15	ISG15	+	25/35	8.34E-03	3
990	Fyn	FYN	+	17/22	8.45E-03	3
972	Kallikrein 6	KLK6	+	17/22	8.45E-03	3
975	RagB	RRAGB	+	17/22	8.45E-03	3
978	Mucin 13	MUC13	+	17/22	8.45E-03	3
979	Piccolo	PCLO	+	17/22	8.45E-03	3
984	RPS3	RPS3	+	17/22	8.45E-03	2
988	RagD	RRAGD	+	17/22	8.45E-03	3
985	FOXK1	FOXK1	-	17/22	8.45E-03	3
992	NUR77	NR4A1	-	17/22	8.45E-03	2
976	miR-301a-3p	MIR301A	-	17/22	8.45E-03	2
980	miR-516a-5p	MIR516A2	-	17/22	8.45E-03	3
981	miR-516a-5p	MIR516A1	-	17/22	8.45E-03	3
993	MSR1	MSR1	-	17/22	8.45E-03	3
974	DRAK1	STK17A	-	17/22	8.45E-03	3
986	USP44	USP44	-	17/22	8.45E-03	3
991	SSTR5	SSTR5	-	17/22	8.45E-03	3
987	NEUR1	NEU1	-	17/22	8.45E-03	3
973	CRIP2	CRIP2	-	17/22	8.45E-03	3
977	SIGIRR	SIGIRR	-	17/22	8.45E-03	3
982	Kendrin	PCNT	-	17/22	8.45E-03	3
983	FRS2beta	FRS3	-	17/22	8.45E-03	3
989	DCTN2	DCTN2	-	17/22	8.45E-03	2
995	CIZ1	CIZ1	+	20/27	9.58E-03	3
1000	TAF4B	TAF4B	+	20/27	9.58E-03	3
998	LINC-PINT	LINC-PINT	+	20/27	9.58E-03	3
994	BS69	ZMYND11	+	20/27	9.58E-03	3
1001	Importin		-	20/27	9.58E-03	3
997	miR-23a-3p	MIR23A	-	20/27	9.58E-03	3
996	EBBP	TRIM16	-	20/27	9.58E-03	3
999	Sestrin 2	SESN2	-	20/27	9.58E-03	3
1011	NF-kB		+	15/19	9.61E-03	3

1005	SBF2-AS1	SBF2-AS1	+	15/19	9.61E-03	3
1008	RagC	RRAGC	+	15/19	9.61E-03	3
1014	PTPD1	PTPN21	+	15/19	9.61E-03	3
1010	CDK6	CDK6	+	15/19	9.61E-03	2
1004	JSAP1	MAPK8IP3	+	15/19	9.61E-03	3
1009	MILI	PIWIL2	+	15/19	9.61E-03	3
1002	PPAR-beta(delta)	PPARD	-	15/19	9.61E-03	2
1003	VENTX	VENTX	-	15/19	9.61E-03	3
1007	RRAD	RRAD	-	15/19	9.61E-03	2
1013	Rab-8	RAB8A	-	15/19	9.61E-03	3
1006	RBCK1	RBCK1	-	15/19	9.61E-03	2
1012	SH3BP-2	SH3BP2	-	15/19	9.61E-03	3

Supplementary Table 3. All predicted causal reasoning transcription factors using DEGs that has log2FC ≥ 2 or ≤ −2 and FDR ≤ 0.05.

Rank	Molecular Entity	Gene	Predicted Activity	Correct/Total network predictions	Activity Prediction p-value	Calculation Distance
4	IRF1	IRF1	+	60/62	4.24E-16	1
52	STAT1	STAT1	+	64/70	1.22E-13	1
55	IRF9	IRF9	+	69/77	1.57E-13	2
58	CNOT7	CNOT7	-	63/69	2.24E-13	2
77	IRF1	IRF1	+	105/132	2.33E-12	2
91	KLF5	KLF5	+	121/158	6.49E-12	2
99	STAT2	STAT2	+	97/122	1.66E-11	2
129	AF-10	MLLT10	+	131/178	1.14E-10	3
149	NRB54	NONO	+	81/101	3.47E-10	2
187	NUR77	NR4A1	-	198/294	1.35E-09	3
194	C/EBPbeta	CEBPB	+	124/171	1.69E-09	2
236	c-Fos	FOS	+	176/260	5.92E-09	3
258	NF-AT4(NFATC3)	NFATC3	+	111/153	1.12E-08	3
266	MECOM	MECOM	-	94/126	1.46E-08	2
267	STAT2	STAT2	+	132/188	1.47E-08	3
268	MEF2D	MEF2D	+	180/269	1.52E-08	3
271	KLF5	KLF5	+	184/276	1.62E-08	3
273	c-Fos	FOS	+	97/131	1.66E-08	2
291	MafK	MAFK	+	185/279	2.74E-08	3
302	HNF1-alpha	HNF1A	+	114/160	3.79E-08	3
334	STAT4	STAT4	+	104/145	8.48E-08	3
341	FOXP3	FOXP3	-	100/139	1.17E-07	3
342	c-Jun	JUN	+	103/144	1.22E-07	2
357	NUR77	NR4A1	-	110/156	1.58E-07	2
360	ATF-5	ATF5	+	195/301	1.63E-07	3
371	CC2D1A	CC2D1A	+	171/260	2.05E-07	3
372	NP220	ZNF638	+	161/243	2.25E-07	3
377	FOXP3	FOXP3	-	65/84	2.37E-07	2
383	IRF1	IRF1	+	208/325	2.53E-07	3
404	GFI-1	GFI1	-	94/131	3.36E-07	2
409	Neurogenin 3	NEUROG3	+	193/300	3.91E-07	3
414	TBP	TBP	+	116/168	4.37E-07	3
425	C/EBPbeta	CEBPB	+	177/273	5.41E-07	3
427	Esrra	ESRRA	-	75/101	5.58E-07	3
428	KLF6	KLF6	+	63/82	5.73E-07	2
430	cKrox	ZBTB7B	+	94/132	5.93E-07	3
431	HES6	HES6	+	178/275	5.94E-07	3
433	JunD	JUND	+	97/137	6.10E-07	3
447	MEF2A	MEF2A	+	192/300	7.10E-07	3
463	MafK	MAFK	+	62/81	8.87E-07	2
464	c-Rel (NF-kB subunit)	REL	+	39/46	9.16E-07	2
466	cKrox	ZBTB7B	+	60/78	9.87E-07	2

475	SIX1	SIX1	+	162/249	1.16E-06	3
491	Fra-1	FOSL1	+	66/88	1.44E-06	3
492	RelA (p65 NF-kB	RELA	+	93/132	1.46E-06	2
494	STAT2	STAT2	+	23/24	1.49E-06	1
512	MECOM	MECOM	-	198/314	2.16E-06	3
516	RARbeta	RARB	-	105/153	2.35E-06	3
520	CUX1	CUX1	-	68/92	2.47E-06	2
524	NPAT	NPAT	+	71/97	2.73E-06	3
525	DPF2	DPF2	+	197/313	2.74E-06	3
530	SOX11	SOX11	+	61/81	2.83E-06	2
527	UBF	UBTF	+	61/81	2.83E-06	3
534	SNFT	BATF3	-	100/145	2.86E-06	3
544	GATA-2	GATA2	-	131/198	3.17E-06	3
547	RelA (p65 NF-kB	RELA	+	59/78	3.21E-06	1
566	NFAT-90	ILF3	+	65/88	4.25E-06	2
569	TBP	TBP	+	60/80	4.29E-06	2
571	JunD	JUND	+	60/80	4.29E-06	2
573	NR2E3	NR2E3	+	157/244	4.37E-06	3
576	SMAD1	SMAD1	+	174/274	4.59E-06	3
582	HES1	HES1	+	117/175	4.84E-06	3
586	c-Jun	JUN	+	151/234	5.19E-06	3
597	BTEB1	KLF9	-	118/177	5.46E-06	3
611	SP1	SP1	+	211/341	6.76E-06	3
612	TFIIB	GTF2B	+	139/214	7.22E-06	3
615	IRF9	IRF9	+	97/142	7.61E-06	3
620	JunB	JUNB	+	177/281	7.88E-06	3
635	CIZ1	CIZ1	+	115/173	8.77E-06	3
636	NKRF	NKRF	-	126/192	8.90E-06	3
639	SRF	SRF	+	146/227	9.55E-06	3
644	PPAR-alpha	PPARA	-	77/109	9.69E-06	2
658	IRX3	IRX3	-	72/101	1.12E-05	3
675	ATF-5	ATF5	+	38/47	1.25E-05	2
684	NF-kB1 (p105)	NFKB1	-	146/228	1.35E-05	3
688	DEAF	DEAF1	+	25/28	1.37E-05	2
689	Menin	MEN1	-	76/108	1.38E-05	2
699	IRF9	IRF9	+	16/16	1.53E-05	1
705	RelB (NF-kB subunit)	RELB	+	60/82	1.62E-05	2
734	KLF11 (TIEG2)	KLF11	-	64/89	2.16E-05	2
775	NF-kB2 (p100)	NFKB2	-	146/231	3.61E-05	3
778	SMARCA5	SMARCA5	+	53/72	3.78E-05	3
797	TFIIA gamma chain	GTF2A2	+	80/117	4.36E-05	3
800	PU.1	SPI1	+	23/26	4.40E-05	1
801	c-Rel (NF-kB subunit)	REL	+	136/214	4.46E-05	3
808	SP7	SP7	+	145/230	4.60E-05	3
812	ATF-4	ATF4	+	173/280	4.79E-05	3
817	CREB3	CREB3	-	126/197	5.43E-05	3
820	NF-AT1(NFATC2)	NFATC2	+	152/243	5.49E-05	3
821	TFIIB	GTF2B	+	66/94	5.56E-05	2

833	ERG	ERG	+	183/299	6.37E-05	3
837	NF-kB1 (p50)	NFKB1	+	164/265	6.54E-05	3
843	Miz-1	ZBTB17	-	170/276	7.01E-05	3
853	NRB54	NONO	+	126/198	7.59E-05	3
855	NF-AT5	NFAT5	+	71/103	7.66E-05	2
863	LRRFIP1	LRRFIP1	+	34/43	8.51E-05	2
864	Neurogenin 3	NEUROG3	+	34/43	8.51E-05	2
878	NF-kB p50/p65	RELA	+	28/34	9.76E-05	2
879	NF-kB p50/p65	NFKB1	+	28/34	9.76E-05	2
880	ARNT	ARNT	+	197/326	9.84E-05	3
900	MEF2A	MEF2A	+	35/45	1.24E-04	2
919	E2F1	E2F1	+	159/259	1.48E-04	3
937	IRF7	IRF7	+	49/68	1.79E-04	3
946	NF-kB p50/p65	RELA	+	18/20	2.01E-04	1
947	NF-kB p50/p65	NFKB1	+	18/20	2.01E-04	1
953	SOX9	SOX9	+	156/255	2.16E-04	3
962	TAF4B	TAF4B	+	69/102	2.34E-04	2
977	IRF6	IRF6	+	81/123	2.78E-04	3
997	GFI-1	GFI1	-	160/264	3.41E-04	3
1006	BATF2	BATF2	+	80/122	3.70E-04	2
1007	PPAR-gamma	PPARG	-	80/122	3.70E-04	2
1010	TAFII55	TAF7	-	109/173	3.87E-04	3
1027	IRF3	IRF3	+	19/22	4.28E-04	1
1036	ATF-7	ATF7	+	86/133	4.57E-04	3
1040	STAT1	STAT1	+	103/163	4.72E-04	2
1043	IRF7	IRF7	+	11/11	4.88E-04	1
1047	NF-AT2(NFATC1)	NFATC1	+	96/151	5.32E-04	2
1063	HSF1	HSF1	+	73/111	5.76E-04	2
1069	NF-AT2(NFATC1)	NFATC1	+	151/250	6.07E-04	3
1077	SOX10	SOX10	+	123/200	7.01E-04	3
1080	NF-kB2 (p100)	NFKB2	-	64/96	7.12E-04	2
1087	NF-kB p65/p65	RELA	+	22/27	7.57E-04	2
1088	HOXA10	HOXA10	+	22/27	7.57E-04	2
1093	NF-kB1 (p50)	NFKB1	+	106/170	7.93E-04	2
1102	CNOT7	CNOT7	-	103/165	8.80E-04	3
1107	SIX4	SIX4	+	46/66	9.29E-04	3
1113	NF-kB1 (p105)	NFKB1	-	63/95	9.62E-04	2
1135	IRF2	IRF2	-	67/102	9.96E-04	2
1117	TFIID	TAF10	+	67/102	9.96E-04	3
1118	TFIID	TAF7L	+	67/102	9.96E-04	3
1119	TFIID	TAF15	+	67/102	9.96E-04	3
1120	TFIID	TAF7	+	67/102	9.96E-04	3
1121	TFIID	TAF8	+	67/102	9.96E-04	3
1122	TFIID	TAF4B	+	67/102	9.96E-04	3
1123	TFIID	TAF1	+	67/102	9.96E-04	3
1124	TFIID	TAF13	+	67/102	9.96E-04	3
1125	TFIID	TAF9B	+	67/102	9.96E-04	3
1126	TFIID	TAF12	+	67/102	9.96E-04	3

1127	TFIID	TAF2	+	67/102	9.96E-04	3
1128	TFIID	TAF9	+	67/102	9.96E-04	3
1129	TFIID	TBP	+	67/102	9.96E-04	3
1130	TFIID	TAF4	+	67/102	9.96E-04	3
1131	TFIID	TAF6	+	67/102	9.96E-04	3
1132	TFIID	TAF3	+	67/102	9.96E-04	3
1133	TFIID	TAF5	+	67/102	9.96E-04	3
1134	TFIID	TAF11	+	67/102	9.96E-04	3
1159	ELF3	ELF3	+	66/101	1.33E-03	2
1176	Bcl-6	BCL6	-	12/13	1.71E-03	1
1190	IRF4	IRF4	+	38/54	1.92E-03	2
1198	NFAT-90	ILF3	+	124/206	2.09E-03	3
1204	NRF2	NFE2L2	-	105/172	2.32E-03	3
1221	NF-kB p52/RelB	NFKB2	+	21/27	2.96E-03	2
1222	NF-kB p52/RelB	RELB	+	21/27	2.96E-03	2
1223	NF-kB p65/c-Rel	RELA	+	21/27	2.96E-03	2
1224	NF-kB p65/c-Rel	REL	+	21/27	2.96E-03	2
1232	BLIMP1 (PRDI-BF1)	PRDM1	-	11/12	3.17E-03	1
1236	NF-kB		+	19/24	3.31E-03	1
1250	NF-AT4(NFATC3)	NFATC3	+	36/52	3.89E-03	2
1262	MafB	MAFB	-	25/34	4.52E-03	2
1264	TFE3	TFE3	+	44/66	4.61E-03	3
1265	NF-kB p50/c-Rel	NFKB1	+	20/26	4.68E-03	2
1266	NF-kB p50/c-Rel	REL	+	20/26	4.68E-03	2
1270	MafB	MAFB	-	56/87	4.84E-03	3
1272	DEAF	DEAF1	+	41/61	4.93E-03	3
1275	Nibrin	NBN	+	45/68	5.17E-03	2
1277	PR (nuclear)	PGR	-	75/121	5.31E-03	2
1278	IRF7	IRF7	+	18/23	5.31E-03	2
1285	MITF	MITF	+	43/65	6.25E-03	2
1286	GATA-3	GATA3	-	12/14	6.47E-03	1
1300	PU.1	SPI1	+	42/64	8.43E-03	2
1303	ATOH8	ATOH8	-	39/59	9.17E-03	2

Supplementary Table 4. Causal reasoning results using DEGs that has log2FC ≥ 5 or ≤ −5 and FDR ≤ 0.05.

Rank	Molecular Entity	Gene	Predicted Activity	Correct/Total network predictions	Activity Prediction p-value	Calculation Distance
83	IRF1	IRF1	+	17/17	7.63E-06	1
409	STAT1	STAT1	+	15/16	2.59E-04	1
534	RelA (p65 NF-kB	RELA	+	14/15	4.88E-04	1
719	IRF3	IRF3	+	9/9	1.95E-03	1
864	STAT2	STAT2	+	8/8	3.91E-03	1
25	IRF1	IRF1	+	23/24	1.49E-06	2
62	MECOM	MECOM	-	21/22	5.48E-06	2
64	c-Jun	JUN	+	21/22	5.48E-06	2
93	KLF5	KLF5	+	23/25	9.72E-06	2
128	C/EBPbeta	CEBPB	+	25/28	1.37E-05	2
130	NFAT-90	ILF3	+	16/16	1.53E-05	2
147	STAT2	STAT2	+	22/24	1.79E-05	2
213	FXR	NR1H4	-	20/22	6.06E-05	2
271	PPAR-alpha	PPARA	-	19/21	1.11E-04	2
298	CUX1	CUX1	-	16/17	1.37E-04	2
360	IRF9	IRF9	+	18/20	2.01E-04	2
396	FOXP3	FOXP3	-	15/16	2.59E-04	2
399	KLF6	KLF6	+	15/16	2.59E-04	2
404	CREB1	CREB1	+	15/16	2.59E-04	2
411	NF-kB2 (p100)	NFKB2	-	15/16	2.59E-04	2
456	Menin	MEN1	-	17/19	3.64E-04	2
479	GFI-1	GFI1	-	19/22	4.28E-04	2
510	DEAF	DEAF1	+	11/11	4.88E-04	2
512	CNOT7	CNOT7	-	14/15	4.88E-04	2
543	TAF4B	TAF4B	+	16/18	6.56E-04	2
545	RelB (NF-kB subunit)	RELB	+	16/18	6.56E-04	2
611	BARX2	BARX2	+	10/10	9.77E-04	2
612	IRF4	IRF4	+	10/10	9.77E-04	2
613	HMGB1	HMGB1	+	10/10	9.77E-04	2
636	PPAR-gamma	PPARG	-	15/17	1.18E-03	2
718	NF-AT4(NFATC3)	NFATC3	+	9/9	1.95E-03	2
720	c-Rel (NF-kB	REL	+	9/9	1.95E-03	2
736	RelA (p65 NF-kB	RELA	+	20/25	2.04E-03	2
754	TFIIB	GTF2B	+	14/16	2.09E-03	2
757	NF-kB1 (p50)	NFKB1	+	18/22	2.17E-03	2
812	PAX7	PAX7	-	11/12	3.17E-03	2
814	MKL1	MRTFA	+	11/12	3.17E-03	2
828	DSIPI (GILZ)	TSC22D3	-	17/21	3.60E-03	2
838	ELF3	ELF3	+	13/15	3.69E-03	2
855	NF-AT5	NFAT5	+	15/18	3.77E-03	2
856	NF-AT2(NFATC1)	NFATC1	+	15/18	3.77E-03	2
862	ATF-5	ATF5	+	8/8	3.91E-03	2

881	STAT1	STAT1	+	20/26	4.68E-03	2
911	NRB54	NONO	+	16/20	5.91E-03	2
929	NF-kB1 (p105)	NFKB1	-	14/17	6.36E-03	2
935	KLF11 (TIEG2)	KLF11	-	14/17	6.36E-03	2
945	TIF1-beta	TRIM28	-	12/14	6.47E-03	2
955	NOTCH1 (NICD)	NOTCH1	+	19/25	7.32E-03	2
960	NF-kB p65/p65	RELA	+	7/7	7.81E-03	2
962	NF-kB p50/p65	RELA	+	7/7	7.81E-03	2
963	NF-kB p50/p65	NFKB1	+	7/7	7.81E-03	2
966	PEG3	PEG3	-	7/7	7.81E-03	2
992	NUR77	NR4A1	-	17/22	8.45E-03	2
1002	PPAR-beta(delta)	PPARD	-	15/19	9.61E-03	2
10	HNF1-alpha	HNF1A	+	28/30	4.34E-07	3
11	IRF1	IRF1	+	33/37	5.42E-07	3
19	TAFII70	TAF6	+	23/24	1.49E-06	3
24	NF-AT4(NFATC3)	NFATC3	+	23/24	1.49E-06	3
28	ATF-5	ATF5	+	31/35	1.73E-06	3
37	SRF	SRF	+	25/27	2.82E-06	3
72	CUX1	CUX1	-	26/29	7.62E-06	3
90	HEYL	HEYL	-	23/25	9.72E-06	3
126	c-Jun	JUN	+	25/28	1.37E-05	3
129	NPAT	NPAT	+	16/16	1.53E-05	3
132	MEF2D	MEF2D	+	27/31	1.70E-05	3
151	MECOM	MECOM	-	29/34	1.93E-05	3
162	CREB3	CREB3	-	24/27	2.46E-05	3
168	PPAR-alpha	PPARA	-	26/30	2.97E-05	3
169	c-Fos	FOS	+	26/30	2.97E-05	3
170	STAT2	STAT2	+	26/30	2.97E-05	3
202	CC2D1A	CC2D1A	+	25/29	5.19E-05	3
209	KLF5	KLF5	+	27/32	5.65E-05	3
210	SMAD1	SMAD1	+	27/32	5.65E-05	3
246	NP220	ZNF638	+	24/28	9.00E-05	3
262	NF-AT1(NFATC2)	NFATC2	+	26/31	9.61E-05	3
264	C/EBPbeta	CEBPB	+	28/34	9.76E-05	3
265	SNFT	BATF3	-	19/21	1.11E-04	3
296	Fra-1	FOSL1	+	16/17	1.37E-04	3
305	NKRF	NKRF	-	21/24	1.39E-04	3
326	ERG	ERG	+	27/33	1.62E-04	3
334	GATA-2	GATA2	-	25/30	1.63E-04	3
361	Esrra	ESRRA	-	18/20	2.01E-04	3
381	DACH1	DACH1	-	28/35	2.54E-04	3
383	UBF	UBTF	+	15/16	2.59E-04	3
438	Bcl-6	BCL6	-	26/32	2.68E-04	3
444	PELP1	PELP1	+	24/29	2.73E-04	3
453	c-Rel (NF-kB	REL	+	24/29	2.73E-04	3
481	NF-kB1 (p105)	NFKB1	-	25/31	4.39E-04	3
485	NR2E3	NR2E3	+	25/31	4.39E-04	3
503	GABP alpha	GABPA	+	21/25	4.55E-04	3

505	AF-10	MLLT10	+	23/28	4.56E-04	3
536	STAT1	STAT1	+	28/36	5.97E-04	3
552	Zac1	PLAGL1	+	26/33	6.59E-04	3
575	SOX11	SOX11	+	22/27	7.57E-04	3
580	TWIST1	TWIST1	+	22/27	7.57E-04	3
592	IRF9	IRF9	+	20/24	7.72E-04	3
605	HEY2	HEY2	-	13/14	9.16E-04	3
610	TLE4	TLE4	-	10/10	9.77E-04	3
620	RelA (p65 NF-kB	RELA	+	25/32	1.05E-03	3
628	RelB (NF-kB subunit)	RELB	+	23/29	1.16E-03	3
657	JunD	JUND	+	21/26	1.25E-03	3
686	ATF-7	ATF7	+	19/23	1.30E-03	3
700	KLF6	KLF6	+	22/28	1.86E-03	3
705	TBP	TBP	+	22/28	1.86E-03	3
712	ENL	MLLT1	+	9/9	1.95E-03	3
726	TAFII55	TAF7	-	20/25	2.04E-03	3
734	SOX10	SOX10	+	20/25	2.04E-03	3
735	BAPX1	NKX3-2	+	20/25	2.04E-03	3
737	SMARCA5	SMARCA5	+	14/16	2.09E-03	3
791	FOXP3	FOXP3	-	21/27	2.96E-03	3
794	PAX6	PAX6	-	21/27	2.96E-03	3
795	NFAT-90	ILF3	+	21/27	2.96E-03	3
798	TFIIB	GTF2B	+	21/27	2.96E-03	3
821	WT1	WT1	-	19/24	3.31E-03	3
842	NRIF	ZNF274	-	13/15	3.69E-03	3
867	KLF11 (TIEG2)	KLF11	-	22/29	4.07E-03	3
874	CNOT7	CNOT7	-	20/26	4.68E-03	3
876	NRB54	NONO	+	20/26	4.68E-03	3
883	NRF2	NFE2L2	-	20/26	4.68E-03	3
884	MEF2C	MEF2C	+	20/26	4.68E-03	3
902	IRF7	IRF7	+	10/11	5.86E-03	3
926	GATA-4	GATA4	+	21/28	6.27E-03	3
927	NF-AT5	NFAT5	+	21/28	6.27E-03	3
937	IRF4	IRF4	+	14/17	6.36E-03	3
967	Myocardin	MYOCD	+	22/30	8.06E-03	3
985	FOXK1	FOXK1	-	17/22	8.45E-03	3
995	CIZ1	CIZ1	+	20/27	9.58E-03	3
1000	TAF4B	TAF4B	+	20/27	9.58E-03	3
1003	VENTX	VENTX	-	15/19	9.61E-03	3
1011	NF-kB		+	15/19	9.61E-03	3

Supplementary Table 5. All adverse events in females for COVID-19 vaccines reported in VAERS database as of 22 November 2021.

Symptoms	Symptoms Code	Events Reported
HEADACHE	10019211	104639
FATIGUE	10016256	88113
PYREXIA	10037660	84563
PAIN	10033371	76962
CHILLS	10008531	71840
NAUSEA	10028813	60339
PAIN IN EXTREMITY	10033425	58773
DIZZINESS	10013573	55089
COVID-19	10084268	37390
MYALGIA	10028411	34673
ARTHRALGIA	10003239	34022
INJECTION SITE PAIN	10022086	33474
DYSPNOEA	10013968	31980
RASH	10037844	31388
PRURITUS	10037087	31208
INJECTION SITE ERYTHEMA	10022061	26402
SARS-COV-2 TEST POSITIVE	10084271	25423
VOMITING	10047700	24063
ASTHENIA	10003549	22671
INJECTION SITE SWELLING	10053425	21565
ERYTHEMA	10015150	21144
DIARRHOEA	10012735	20075
URTICARIA	10046735	19911
LYMPHADENOPATHY	10025197	19794
FEELING ABNORMAL	10016322	19726
PARAESTHESIA	10033775	19690
COUGH	10011224	19449
INJECTION SITE PRURITUS	10022093	18197
HYPOAESTHESIA	10020937	18105
MALAISE	10025482	17644
SARS-COV-2 TEST	10084354	16068
PERIPHERAL SWELLING	10048959	15662
CHEST PAIN	10008479	15524
HYPERHIDROSIS	10020642	14499
EXPIRED PRODUCT ADMINISTERED	10074902	13789
INJECTION SITE WARMTH	10022112	13608
VACCINATION SITE PAIN	10068879	13151
BLOOD TEST	10061726	12959

PRODUCT STORAGE ERROR	10079843	12821
PALPITATIONS	10033557	12541
CHEST DISCOMFORT	10008469	12404
NO ADVERSE EVENT	10067482	12183
CONDITION AGGRAVATED	10010264	11950
OROPHARYNGEAL PAIN	10068319	11777
BACK PAIN	10003988	11372
HEART RATE INCREASED	10019303	11344
SWELLING	10042674	10660
TREMOR	10044565	10600
FEELING HOT	10016334	10003
INJECTION SITE RASH	10022094	9834
DECREASED APPETITE	10061428	9650
TINNITUS	10043882	9449
NECK PAIN	10028836	9363
SYNCOPE	10042772	9065
RASH ERYTHEMATOUS	10037855	8902
MOBILITY DECREASED	10048334	8567
ELECTROCARDIOGRAM	10014362	8464
MIGRAINE	10027599	8401
SKIN WARM	10040952	8132
BODY TEMPERATURE	10005906	7910
RASH PRURITIC	10037884	7670
INFLUENZA LIKE ILLNESS	10022004	7445
SARS-COV-2 TEST NEGATIVE	10084273	7140
FLUSHING	10016825	6969
MUSCLE SPASMS	10028334	6961
PRODUCT ADMINISTERED TO PATIENT OF INAPPROPRIATE AGI	10081578	6748
SLEEP DISORDER	10040984	6725
INAPPROPRIATE SCHEDULE OF PRODUCT ADMINISTRATION	10081572	6698
LOSS OF CONSCIOUSNESS	10024855	6608
ANXIETY	10002855	6583
INSOMNIA	10022437	6557
VACCINATION SITE ERYTHEMA	10059079	6549
VERTIGO	10047340	6468
LABORATORY TEST	10059938	6440
ABDOMINAL PAIN UPPER	10000087	6438
SWELLING FACE	10042682	6396
BURNING SENSATION	10006784	6376
RHINORRHOEA	10039101	6326
GAIT DISTURBANCE	10017577	6262
COMPUTERISED TOMOGRAM	10010234	6026
PARAESTHESIA ORAL	10057372	5954

ABDOMINAL PAIN	10000081	5906
THROAT IRRITATION	10043521	5778
THROAT TIGHTNESS	10043528	5730
FEELING COLD	10016326	5635
MUSCULAR WEAKNESS	10028372	5605
IMPAIRED WORK ABILITY	10052302	5583
HYPERTENSION	10020772	5429
MUSCULOSKELETAL STIFFNESS	10052904	5423
LETHARGY	10024264	5415
BODY TEMPERATURE INCREASED	10005911	5405
VACCINATION SITE SWELLING	10069620	5373
DRUG INEFFECTIVE	10013709	5360
VISION BLURRED	10047513	5360
AXILLARY PAIN	10048750	5335
SOMNOLENCE	10041349	5333
HERPES ZOSTER	10019974	5247
TACHYCARDIA	10043071	5166
INJECTION SITE INDURATION	10022075	5092
HEAVY MENSTRUAL BLEEDING	10085423	5070
BLOOD PRESSURE INCREASED	10005750	5056
INCORRECT DOSE ADMINISTERED	10064355	5034
DEATH	10011906	5031
INTERCHANGE OF VACCINE PRODUCTS	10070574	5028
FULL BLOOD COUNT	10017411	5023
EXTRA DOSE ADMINISTERED	10064366	4806
ABDOMINAL DISCOMFORT	10000059	4767
ILLNESS	10080284	4750
LYMPH NODE PAIN	10025182	4743
NASAL CONGESTION	10028735	4743
IMMEDIATE POST-INJECTION REACTION	10067142	4712
CHEST X-RAY	10008498	4691
FALL	10016173	4590
LIP SWELLING	10024570	4499
MAGNETIC RESONANCE IMAGING	10078223	4438
TENDERNESS	10043224	4369
LOSS OF PERSONAL INDEPENDENCE IN DAILY ACTIVITIES	10079487	4367
LIMB DISCOMFORT	10061224	4273
METABOLIC FUNCTION TEST	10062191	4215
DYSPHAGIA	10013950	4187
VACCINATION SITE PRURITUS	10068881	4182
INJECTION SITE REACTION	10022095	4174
RESPIRATORY TRACT CONGESTION	10052251	4154
HYPOAESTHESIA ORAL	10057371	4110

UNEVALUABLE EVENT	10062355	3983
SWOLLEN TONGUE	10042727	3975
DISCOMFORT	10013082	3906
DYSGEUSIA	10013911	3898
CONFUSIONAL STATE	10010305	3868
MENSTRUATION IRREGULAR	10027339	3860
ELECTROCARDIOGRAM NORMAL	10014373	3834
PHARYNGEAL SWELLING	10082270	3775
EXPOSURE DURING PREGNANCY	10073513	3772
HYPERSENSITIVITY	10020751	3765
AGEUSIA	10001480	3685
RASH MACULAR	10037867	3632
BLOOD TEST NORMAL	10050540	3617
CONTUSION	10050584	3551
EAR PAIN	10014020	3486
NASOPHARYNGITIS	10028810	3404
ULTRASOUND SCAN	10045434	3403
VACCINATION SITE WARMTH	10069624	3399
INFLAMMATION	10061218	3395
VACCINE BREAKTHROUGH INFECTION	10067923	3318
HOT FLUSH	10060800	3279
X-RAY	10048064	3231
VISUAL IMPAIRMENT	10047571	3225
ECHOCARDIOGRAM	10014113	3188
BLOOD PRESSURE MEASUREMENT	10076581	3125
PALLOR	10033546	3082
ANOSMIA	10002653	2995
BALANCE DISORDER	10049848	2967
JOINT SWELLING	10023232	2955
MENSTRUAL DISORDER	10027327	2928
VACCINATION COMPLICATION	10046861	2865
THROMBOSIS	10043607	2864
RASH PAPULAR	10037876	2841
URINE ANALYSIS	10046614	2820
SEIZURE	10039906	2775
HYPOTENSION	10021097	2773
HEART RATE	10019299	2755
UNDERDOSE	10057362	2751
VACCINATION FAILURE	10046862	2726
COLD SWEAT	10009866	2710
CHEST X-RAY NORMAL	10008500	2703
INJECTION SITE MASS	10022081	2692
EYE PAIN	10015958	2689

EYE SWELLING	10015967	2669
WHEEZING	10047924	2664
HEAD DISCOMFORT	10019194	2655
VACCINATION SITE RASH	10069482	2570
UNRESPONSIVE TO STIMULI	10045555	2503
PRODUCT PREPARATION ISSUE	10081301	2469
INCORRECT PRODUCT FORMULATION ADMINISTERED	10074946	2457
IMMUNISATION	10021430	2436
POOR QUALITY PRODUCT ADMINISTERED	10081478	2427
FULL BLOOD COUNT NORMAL	10017414	2260
HYPERSOMNIA	10020765	2259
NEURALGIA	10029223	2234
COVID-19 PNEUMONIA	10084380	2224
CHEST X-RAY ABNORMAL	10008499	2210
EAR DISCOMFORT	10052137	2208
PRESYNCOPE	10036653	2191
PRODUCT TEMPERATURE EXCURSION ISSUE	10083571	2191
BONE PAIN	10006002	2189
DYSSTASIA	10050256	2154
SKIN DISCOLOURATION	10040829	2149
DEHYDRATION	10012174	2141
DYSMENORRHOEA	10013935	2139
BREAST PAIN	10006298	2081
INJECTION SITE BRUISING	10022052	2064
BLISTER	10005191	2043
NIGHT SWEATS	10029410	2037
BELL'S PALSY	10004223	2001
PNEUMONIA	10035664	2001
LABORATORY TEST NORMAL	10054052	1977
CEREBROVASCULAR ACCIDENT	10008190	1955
SNEEZING	10041232	1941
INDURATION	10060708	1915
PULMONARY EMBOLISM	10037377	1900
MUSCLE TWITCHING	10028347	1873
PAIN IN JAW	10033433	1864
URINARY TRACT INFECTION	10046571	1858
GAIT INABILITY	10017581	1843
NERVOUSNESS	10029216	1832
ATRIAL FIBRILLATION	10003658	1826
TASTE DISORDER	10082490	1825
ANTICOAGULANT THERAPY	10053468	1823
HYPOXIA	10021143	1803
EXPOSURE TO SARS-COV-2	10084456	1784

DRY MOUTH	10013781	1777
VAGINAL HAEMORRHAGE	10046910	1750
DYSPHONIA	10013952	1742
DISTURBANCE IN ATTENTION	10013496	1738
MUSCLE TIGHTNESS	10049816	1737
EPISTAXIS	10015090	1734
ELECTROCARDIOGRAM ABNORMAL	10014363	1693
FACIAL PARALYSIS	10016062	1693
PHOTOPHOBIA	10034960	1659
ANAPHYLACTIC REACTION	10002198	1650
OEDEMA PERIPHERAL	10030124	1636
ALOPECIA	10001760	1626
COMPUTERISED TOMOGRAM HEAD	10054003	1621
INJECTED LIMB MOBILITY DECREASED	10057664	1615
COMPUTERISED TOMOGRAM NORMAL	10010236	1614
DISORIENTATION	10013395	1604
ASTHMA	10003553	1590
PAIN OF SKIN	10033474	1547
PRODUCT DOSE OMISSION ISSUE	10084406	1546
JOINT RANGE OF MOTION DECREASED	10048706	1539
VACCINE POSITIVE RECHALLENGE	10066903	1505
OFF LABEL USE	10053762	1500
INTERMENSTRUAL BLEEDING	10022559	1497
INTENSIVE CARE	10022519	1493
BLOOD GLUCOSE NORMAL	10005558	1492
PRODUCTIVE COUGH	10036790	1492
WEIGHT DECREASED	10047895	1490
MEMORY IMPAIRMENT	10027175	1486
ARTHRITIS	10003246	1479
DYSPEPSIA	10013946	1478
MOVEMENT DISORDER	10028035	1470
WRONG PRODUCT ADMINISTERED	10081579	1467
PERIPHERAL COLDNESS	10034568	1466
WHITE BLOOD CELL COUNT INCREASED	10047943	1457
ACUTE RESPIRATORY FAILURE	10001053	1438
SPEECH DISORDER	10041466	1432
CELLULITIS	10007882	1414
ABDOMINAL DISTENSION	10000060	1413
HEART RATE IRREGULAR	10019304	1408
OXYGEN SATURATION DECREASED	10033318	1406
MAGNETIC RESONANCE IMAGING HEAD	10085255	1401
APHASIA	10002948	1393
RETCHING	10038776	1393

INJECTION SITE URTICARIA	10022107	1378
BLOOD GLUCOSE INCREASED	10005557	1374
OROPHARYNGEAL DISCOMFORT	10068318	1362
BLOOD TEST ABNORMAL	10061016	1339
SKIN BURNING SENSATION	10054786	1336
DEEP VEIN THROMBOSIS	10051055	1293
FIBRIN D DIMER	10016577	1291
ABORTION SPONTANEOUS	10000234	1263
SINUSITIS	10040753	1260
NODULE	10054107	1260
FACIAL PAIN	10016059	1249
MENSTRUATION DELAYED	10027336	1248
EYE PRURITUS	10052140	1248
VACCINATION SITE REACTION	10059080	1247
FEEDING DISORDER	10061148	1245
OCULAR HYPERAEMIA	10030041	1241
VACCINATION SITE MASS	10076182	1235
C-REACTIVE PROTEIN INCREASED	10006825	1225
FIBRIN D DIMER INCREASED	10016581	1222
ORAL HERPES	10067152	1221
SUSPECTED COVID-19	10084451	1214
VACCINATION SITE INDURATION	10065117	1209
MATERNAL EXPOSURE DURING PREGNANCY	10071408	1196
INFLUENZA	10022000	1191
THIRST	10043458	1184
SARS-COV-2 ANTIBODY TEST	10084501	1172
FEELING OF BODY TEMPERATURE CHANGE	10061458	1166
TROPONIN	10061576	1166
HEAD INJURY	10019196	1158
ULTRASOUND DOPPLER	10045412	1155
DYSPNOEA EXERTIONAL	10013971	1130
SENSITIVE SKIN	10081765	1119
BLOOD THYROID STIMULATING HORMONE	10005829	1111
PAROSMIA	10034018	1099
AMNESIA	10001949	1096
MUSCULOSKELETAL DISCOMFORT	10053156	1088
JOINT STIFFNESS	10023230	1086
DYSARTHRIA	10013887	1083
HAEMOGLOBIN DECREASED	10018884	1076
INJECTION SITE NODULE	10057880	1074
SINUS CONGESTION	10040742	1067
COMPUTERISED TOMOGRAM THORAX ABNORMAL	10057799	1064
DEPRESSION	10012378	1053

MUSCULOSKELETAL CHEST PAIN	10050819	1053
HYPOACUSIS	10048865	1051
PANIC ATTACK	10033664	1049
COMPUTERISED TOMOGRAM THORAX	10053875	1049
LACRIMATION INCREASED	10023644	1044
NEUROPATHY PERIPHERAL	10029331	1043
ACUTE KIDNEY INJURY	10069339	1042
MASS	10026865	1038
PLATELET COUNT DECREASED	10035528	1025
SWELLING OF EYELID	10042690	1025
CARDIAC FLUTTER	10052840	1011
MAGNETIC RESONANCE IMAGING NORMAL	10078225	1002
CARDIAC STRESS TEST	10061027	997
SENSORY DISTURBANCE	10040026	994
INFLUENZA VIRUS TEST NEGATIVE	10070718	989
BLOOD PRESSURE DECREASED	10005734	984
ECHOCARDIOGRAM NORMAL	10014115	984
IMPAIRED DRIVING ABILITY	10049564	984
INVESTIGATION	10062026	984
SENSATION OF FOREIGN BODY	10061549	982
DEAFNESS	10011878	980
CARDIAC MONITORING	10053438	980
RHEUMATOID ARTHRITIS	10039073	976
BLOOD GLUCOSE	10005553	974
COMPUTERISED TOMOGRAM ABNORMAL	10010235	972
DYSKINESIA	10013916	965
EYE IRRITATION	10015946	961
VITAL SIGNS MEASUREMENT	10072952	961
BIOPSY	10004720	959
ENDOTRACHEAL INTUBATION	10067450	950
INJECTION SITE INFLAMMATION	10022078	945
MENTAL STATUS CHANGES	10048294	939
CRYING	10011469	933
COMPUTERISED TOMOGRAM HEAD NORMAL	10072167	922
TROPONIN INCREASED	10058267	922
SKIN EXFOLIATION	10040844	917
URINE ANALYSIS NORMAL	10061578	913
HAEMORRHAGE	10055798	904
CONSTIPATION	10010774	897
INCORRECT ROUTE OF PRODUCT ADMINISTRATION	10081202	891
C-REACTIVE PROTEIN	10006824	887
LUNG OPACITY	10081792	882
ANGIOGRAM	10061637	872

MAGNETIC RESONANCE IMAGING HEAD NORMAL	10085257	862
DIFFERENTIAL WHITE BLOOD CELL COUNT	10012784	857
AMENORRHOEA	10001928	852
PARANASAL SINUS DISCOMFORT	10052438	848
TOOTHACHE	10044055	841
PHARYNGEAL PARAESTHESIA	10076737	834
LABORATORY TEST ABNORMAL	10023547	831
METABOLIC FUNCTION TEST NORMAL	10062192	827
MOUTH SWELLING	10075203	812
MAGNETIC RESONANCE IMAGING HEAD ABNORMAL	10085256	811
PLATELET COUNT NORMAL	10035530	810
ECHOCARDIOGRAM ABNORMAL	10061593	802
BREAST SWELLING	10006312	798
DRY SKIN	10013786	795
DRY THROAT	10013789	790
ASYMPTOMATIC COVID-19	10084459	789
OXYGEN SATURATION	10033316	787
PERICARDITIS	10034484	787
GASTROOESOPHAGEAL REFLUX DISEASE	10017885	771
INFECTION	10021789	770
POLYMENORRHOEA	10036086	767
ELECTROCARDIOGRAM AMBULATORY	10014368	765
STOMATITIS	10042128	764
AXILLARY MASS	10049021	763
SEPSIS	10040047	760
ELECTRIC SHOCK SENSATION	10014358	759
BLINDNESS	10005169	756
OCULAR DISCOMFORT	10052143	755
CARDIAC DISORDER	10061024	748
HEART RATE DECREASED	10019301	747
FEAR	10016275	744
MYOCARDITIS	10028606	744
BLOOD CREATININE INCREASED	10005483	741
STREPTOCOCCUS TEST NEGATIVE	10070415	741
MYOCARDIAL INFARCTION	10028596	739
ORAL DISCOMFORT	10030973	731
ULTRASOUND SCAN NORMAL	10061607	730
TROPONIN NORMAL	10071322	729
ANGIOEDEMA	10002424	728
PREGNANCY TEST	10036572	728
RESTLESSNESS	10038743	724
DEAFNESS UNILATERAL	10048812	721
ANAEMIA	10002034	720

WEIGHT	10047890	719
DIPLOPIA	10013036	717
ANGINA PECTORIS	10002383	715
ARRHYTHMIA	10003119	711
ACOUSTIC STIMULATION TESTS	10000525	704
IMMUNISATION REACTION	10021432	700
BEDRIDDEN	10048948	700
FLATULENCE	10016766	699
URINARY INCONTINENCE	10046543	693
ABDOMINAL PAIN LOWER	10000084	686
ULTRASOUND SCAN ABNORMAL	10061606	684
COMPUTERISED TOMOGRAM ABDOMEN	10053876	683
ULTRASOUND DOPPLER ABNORMAL	10045413	681
VENTRICULAR EXTRASYSTOLES	10047289	681
SCAN WITH CONTRAST	10059696	680
OLIGOMENORRHOEA	10030295	675
GENERAL PHYSICAL HEALTH DETERIORATION	10049438	674
PRODUCT ADMINISTERED AT INAPPROPRIATE SITE	10080753	674
SKIN REACTION	10040914	672
MAMMOGRAM	10026735	667
GASTROINTESTINAL DISORDER	10017944	666
HYPOKINESIA	10021021	665
POLLAKIURIA	10036018	665
HYPOPHAGIA	10063743	664
SYRINGE ISSUE	10069218	657
HALLUCINATION	10019063	655
ADVERSE REACTION	10067484	653
PRODUCT ADMINISTRATION ERROR	10081576	652
SKIN LESION	10040882	649
SPINAL PAIN	10072005	649
COGNITIVE DISORDER	10057668	646
ANTIBODY TEST	10060979	645
WHITE BLOOD CELL COUNT NORMAL	10047944	644
SKIN SWELLING	10053262	640
POSTMENOPAUSAL HAEMORRHAGE	10055870	632
COMPUTERISED TOMOGRAM ABDOMEN ABNORMAL	10057798	627
MAGNETIC RESONANCE IMAGING ABNORMAL	10078224	625
CARDIAC ARREST	10007515	622
COMPUTERISED TOMOGRAM HEAD ABNORMAL	10072168	622
MECHANICAL URTICARIA	10068773	620
BLOOD POTASSIUM DECREASED	10005724	617
HEMIPARESIS	10019465	617
INFLUENZA B VIRUS TEST	10071544	617

RED BLOOD CELL SEDIMENTATION RATE INCREASED	10049187	610
NEUROLOGICAL SYMPTOM	10060860	610
PERIORBITAL SWELLING	10056647	609
PRODUCT USE ISSUE	10076309	608
IRRITABILITY	10022998	606
ORAL PAIN	10031009	604
RESPIRATORY FAILURE	10038695	603
DIZZINESS POSTURAL	10013578	601
PLEURAL EFFUSION	10035598	601
X-RAY NORMAL	10048067	601
HAEMOGLOBIN NORMAL	10018890	600
BRONCHITIS	10006451	597
RED BLOOD CELL SEDIMENTATION RATE	10049184	596
UPPER-AIRWAY COUGH SYNDROME	10070488	593
PAINFUL RESPIRATION	10033517	588
PRODUCT PREPARATION ERROR	10076869	588
ANTINUCLEAR ANTIBODY	10002807	584
ECZEMA	10014184	583
BLOOD CREATININE NORMAL	10005484	582
RED BLOOD CELL SEDIMENTATION RATE NORMAL	10049408	582
BREAST TENDERNESS	10006313	580
FEELING JITTERY	10016338	579
INJECTION SITE HYPOAESTHESIA	10074586	579
PETECHIAE	10034754	578
SLUGGISHNESS	10041052	578
MUSCULOSKELETAL PAIN	10028391	576
ANGIOGRAM PULMONARY ABNORMAL	10002441	573
WHITE BLOOD CELL COUNT	10047939	566
EYE MOVEMENT DISORDER	10061129	565
URINE ANALYSIS ABNORMAL	10062226	563
HYPERACUSIS	10020559	560
SINUS DISORDER	10062244	558
BLOOD URINE PRESENT	10018870	556
LUMBAR PUNCTURE	10024999	555
WHITE BLOOD CELL COUNT DECREASED	10047942	555
PSORIASIS	10037153	554
HYPERVENTILATION	10020910	553
TONGUE DISCOMFORT	10077855	552
ADVERSE EVENT	10060933	552
DRY EYE	10013774	548
THERAPEUTIC RESPONSE UNEXPECTED	10043417	548
APHONIA	10002953	546
LUNG INFILTRATION	10025102	543

INJECTION SITE DISCOMFORT	10054266	540
VACCINATION SITE BRUISING	10069484	536
WEIGHT INCREASED	10047899	533
INJECTION SITE PARAESTHESIA	10022088	531
MENTAL IMPAIRMENT	10027374	530
SKIN IRRITATION	10040880	530
FLANK PAIN	10016750	529
BLOOD SODIUM DECREASED	10005802	526
RASH VESICULAR	10037898	524
BLEPHAROSPASM	10005159	523
INJECTION SITE DISCOLOURATION	10051572	522
C-REACTIVE PROTEIN NORMAL	10006826	521
PLATELET COUNT	10035525	520
BLOOD POTASSIUM NORMAL	10005726	519
INFLUENZA A VIRUS TEST NEGATIVE	10070417	509
ASPARTATE AMINOTRANSFERASE INCREASED	10003481	505
SKIN TIGHTNESS	10050637	504
ALANINE AMINOTRANSFERASE INCREASED	10001551	500
RENAL PAIN	10038490	496
GLOSSODYNIA	10018388	495
HYPERAESTHESIA	10020568	495
BREAST MASS	10006272	494
TONGUE DISORDER	10043951	494
ELECTROENCEPHALOGRAM	10014407	493
BLOOD THYROID STIMULATING HORMONE NORMAL	10005834	488
DYSURIA	10013990	487
APPENDICITIS	10003011	482
BLOOD SODIUM NORMAL	10005804	478
STRESS	10042209	478
TRANSIENT ISCHAEMIC ATTACK	10044390	478
LUNG DISORDER	10025082	476
AUTOIMMUNE DISORDER	10061664	476
OEDEMA	10030095	475
CHRONIC OBSTRUCTIVE PULMONARY DISEASE	10009033	468
EAR SWELLING	10014025	467
EYE DISORDER	10015916	467
SINUS TACHYCARDIA	10040752	462
LIMB INJURY	10061225	459
SINUS PAIN	10040747	458
SECRETION DISCHARGE	10053459	454
BLOOD LACTIC ACID	10005632	452
GUILLAIN-BARRE SYNDROME	10018767	449
BRADYCARDIA	10006093	448

ORAL PRURITUS	10052894	448
PULMONARY THROMBOSIS	10037437	446
THROMBOCYTOPENIA	10043554	446
COLONOSCOPY	10010007	445
PARALYSIS	10033799	445
PELVIC PAIN	10034263	445
TONGUE PRURITUS	10070072	445
HAEMATOCRIT NORMAL	10018842	444
RESPIRATORY DISTRESS	10038687	444
PERICARDIAL EFFUSION	10034474	443
PHYSICAL EXAMINATION	10034986	443
EXERCISE TOLERANCE DECREASED	10051301	443
INJECTION SITE HAEMORRHAGE	10022067	441
ULTRASOUND ABDOMEN	10052038	441
TACHYPNOEA	10043089	439
CARDIAC FAILURE CONGESTIVE	10007559	438
VITREOUS FLOATERS	10047654	436
WALKING AID USER	10050778	435
DEPRESSED MOOD	10012374	434
ACUTE MYOCARDIAL INFARCTION	10000891	432
GRIP STRENGTH DECREASED	10062556	431
BLOOD UREA INCREASED	10005851	430
POOR QUALITY SLEEP	10062519	430
CARDIOMEGALY	10007632	429
POSITIVE AIRWAY PRESSURE THERAPY	10086397	429
FIBROMYALGIA	10048439	428
MUSCLE FATIGUE	10049565	428
CYANOSIS	10011703	427
EAR INFECTION	10014011	426
HAEMATOCRIT DECREASED	10018838	426
EXTRASYSTOLES	10015856	425
PREGNANCY TEST NEGATIVE	10036574	425
SENSORY LOSS	10040030	423
PULMONARY PAIN	10074693	423
AGITATION	10001497	422
ELECTROMYOGRAM	10014430	420
FIBRIN D DIMER NORMAL	10016583	420
BLOOD GLUCOSE DECREASED	10005555	419
PHARYNGEAL HYPOAESTHESIA	10059923	419
THYROID FUNCTION TEST	10043729	418
MECHANICAL VENTILATION	10067221	416
FULL BLOOD COUNT ABNORMAL	10017412	408
HAEMATOCHEZIA	10018836	404

IMMUNODEFICIENCY	10061598	403
BLOOD MAGNESIUM	10005651	398
ULTRASOUND DOPPLER NORMAL	10045414	397
BLOOD PRESSURE ABNORMAL	10005728	393
HYPOPNOEA	10021079	392
SKIN DISORDER	10040831	391
PERIARTHRITIS	10034464	390
SUPRAVENTRICULAR TACHYCARDIA	10042604	389
OPHTHALMOLOGICAL EXAMINATION	10050320	388
ABNORMAL DREAMS	10000125	386
CHROMATURIA	10008796	384
BLOOD CHLORIDE NORMAL	10005421	383
PULMONARY CONGESTION	10037368	382
INFLUENZA VIRUS TEST	10070715	381
BIOPSY SKIN	10004873	380
THINKING ABNORMAL	10043431	380
ATELECTASIS	10003598	379
DERMATITIS	10012431	379
SCAN WITH CONTRAST ABNORMAL	10062152	379
HEART RATE ABNORMAL	10019300	376
PULMONARY OEDEMA	10037423	375
CULTURE URINE	10011638	374
INJECTION SITE CELLULITIS	10050057	372
ACNE	10000496	371
RESPIRATORY RATE INCREASED	10038712	370
SCIATICA	10039674	370
SURGERY	10042609	369
MEAN CELL VOLUME NORMAL	10027006	368
ANTINUCLEAR ANTIBODY POSITIVE	10060055	366
FORMICATION	10017062	365
RESUSCITATION	10038749	365
ACOUSTIC STIMULATION TESTS ABNORMAL	10000526	364
ALANINE AMINOTRANSFERASE NORMAL	10001552	364
BLOOD UREA NORMAL	10005857	363
BODY TEMPERATURE DECREASED	10005910	363
THROAT CLEARING	10080125	362
HEPATIC ENZYME INCREASED	10060795	362
NIGHTMARE	10029412	360
IMMUNOGLOBULIN THERAPY	10069534	360
BRAIN NATRIURETIC PEPTIDE INCREASED	10053405	360
BLOOD ALKALINE PHOSPHATASE NORMAL	10005310	357
MAGNETIC RESONANCE IMAGING NECK	10083140	357
ALLERGY TEST	10053462	355

DELIRIUM	10012218	353
EMOTIONAL DISTRESS	10049119	353
POLYMERASE CHAIN REACTION	10050967	353
EATING DISORDER	10014062	352
GLYCOSYLATED HAEMOGLOBIN	10018480	351
VACCINATION SITE URTICARIA	10069622	350
NERVOUS SYSTEM DISORDER	10029202	349
NEUROLOGICAL EXAMINATION	10050318	346
RESPIRATORY SYNCYTIAL VIRUS TEST NEGATIVE	10068564	346
COMPUTERISED TOMOGRAM NECK	10082961	346
INTERNATIONAL NORMALISED RATIO	10022591	345
X-RAY LIMB	10061585	345
BLOOD CULTURE	10005485	344
RED BLOOD CELL COUNT DECREASED	10038153	344
GROIN PAIN	10018735	343
PHOTOPSIA	10034962	342
DELIVERY	10067647	342
LIP PRURITUS	10070721	342
BURSITIS	10006811	341
HYPONATRAEMIA	10021036	340
ANION GAP	10002522	339
INCOMPLETE COURSE OF VACCINATION	10072103	337
VACCINATION SITE DISCOLOURATION	10069474	336
LIMB MASS	10078503	336
LYMPHADENITIS	10025188	335
ORAL MUCOSAL BLISTERING	10030995	335
SCAB	10039509	335
APPENDICECTOMY	10003010	334
ASTHENOPIA	10003552	333
BLOOD MAGNESIUM NORMAL	10005656	331
BLOOD PRESSURE FLUCTUATION	10005746	331
STREPTOCOCCUS TEST	10070414	330
GASTROINTESTINAL PAIN	10017999	328
VACCINATION SITE MOVEMENT IMPAIRMENT	10076183	327
SINUS HEADACHE	10040744	326
LIVER FUNCTION TEST	10060105	325
HAEMOGLOBIN	10018876	323
ALLERGY TO VACCINE	10055048	323
PROTHROMBIN TIME	10037056	322
TRANSFUSION	10066152	319
SKIN MASS	10067868	317
VITAMIN D	10050713	316
BLOOD BILIRUBIN NORMAL	10005367	313

MATERNAL EXPOSURE DURING BREAST FEEDING	10080752	311
ASPARTATE AMINOTRANSFERASE NORMAL	10003482	309
RESPIRATORY DISORDER	10038683	309
SEIZURE LIKE PHENOMENA	10071048	308
POST-ACUTE COVID-19 SYNDROME	10085503	308
RED BLOOD CELL COUNT NORMAL	10038157	307
TENSION HEADACHE	10043269	307
THYROID FUNCTION TEST NORMAL	10043731	307
ANTINUCLEAR ANTIBODY NEGATIVE	10002809	305
ENDOSCOPY	10014805	305
NASAL DISCOMFORT	10052437	305
GINGIVAL PAIN	10018286	304
CARDIAC FAILURE	10007554	303
GLOMERULAR FILTRATION RATE DECREASED	10018358	303
COMPUTERISED TOMOGRAM THORAX NORMAL	10057801	303
MOUTH ULCERATION	10028034	301
FLUID RETENTION	10016807	298
LYMPHOEDEMA	10025282	298
WRONG TECHNIQUE IN PRODUCT USAGE PROCESS	10076573	298
MEAN CELL HAEMOGLOBIN CONCENTRATION NORMAL	10026994	294
HYPOKALAEMIA	10021015	293
NERVE INJURY	10052897	293
APHTHOUS ULCER	10002959	292
CATHETERISATION CARDIAC	10007815	292
DISEASE RECURRENCE	10061819	292
CEREBRAL HAEMORRHAGE	10008111	290
EYE HAEMORRHAGE	10015926	290
FEELING DRUNK	10016330	290
TUNNEL VISION	10045178	290
FACIAL PARESIS	10051267	290
LEUKOCYTOSIS	10024378	289
BLOOD CULTURE NEGATIVE	10005486	288
INJECTION SITE VESICLES	10022111	288
RESPIRATION ABNORMAL	10038647	288
BLOOD CREATINE PHOSPHOKINASE	10005467	287
COLITIS	10009887	287
HAEMOPTYSIS	10018964	287
SYSTEMIC LUPUS ERYTHEMATOSUS	10042945	287
UPPER RESPIRATORY TRACT INFECTION	10046306	287
RED CELL DISTRIBUTION WIDTH NORMAL	10053922	287
SCRATCH	10039737	285
ACCIDENTAL UNDERDOSE	10074904	285
ERUCTATION	10015137	284

MATERNAL EXPOSURE BEFORE PREGNANCY	10071406	284
ULTRASOUND SCAN VAGINA	10045438	281
PULMONARY MASS	10056342	280
PREGNANCY	10036556	279
IMMUNE SYSTEM DISORDER	10021425	278
RHEUMATOID FACTOR	10039079	278
NEPHROLITHIASIS	10029148	277
SARS-COV-2 ANTIBODY TEST NEGATIVE	10084509	276
ACCIDENTAL OVERDOSE	10000381	275
DIFFERENTIAL WHITE BLOOD CELL COUNT NORMAL	10012787	275
NECK MASS	10049146	275
RESPIRATORY SYMPTOM	10075535	274
LOCAL REACTION	10024769	273
GENERALISED TONIC-CLONIC SEIZURE	10018100	272
JOINT INJURY	10060820	272
ADVERSE DRUG REACTION	10061623	272
ARTHROPATHY	10003285	271
ACTIVATED PARTIAL THROMBOPLASTIN TIME	10000630	270
INJECTION SITE IRRITATION	10022079	270
TROPONIN I	10050397	270
PLATELET COUNT INCREASED	10051608	270
FACIAL DISCOMFORT	10083537	270
CARBON DIOXIDE NORMAL	10007228	269
CYSTITIS	10011781	269
VACCINATION SITE DISCOMFORT	10069478	268
BLOOD CALCIUM NORMAL	10005397	267
VITAMIN B12	10047602	267
PROTEIN TOTAL NORMAL	10037017	266
X-RAY ABNORMAL	10048065	266
EAR CONGESTION	10052136	266
BLOOD ALKALINE PHOSPHATASE INCREASED	10059570	266
PULMONARY FUNCTION TEST	10059914	264
DEVICE CONNECTION ISSUE	10065066	264
BLINDNESS UNILATERAL	10005186	263
FAECES DISCOLOURED	10016100	263
HAEMATURIA	10018867	263
OVERDOSE	10033295	263
LIP BLISTER	10049307	263
DEPRESSED LEVEL OF CONSCIOUSNESS	10012373	262
TENDONITIS	10043255	262
MAGNETIC RESONANCE IMAGING SPINAL	10083133	261
ODYNOPHAGIA	10030094	259
SARS-COV-2 ANTIBODY TEST POSITIVE	10084491	259

ENCEPHALOPATHY	10014625	258
BLOOD CALCIUM DECREASED	10005395	257
MAMMOGRAM ABNORMAL	10026736	257
SCAR	10039580	256
RESTLESS LEGS SYNDROME	10058920	256
BLOOD THYROID STIMULATING HORMONE DECREASED	10005832	255
DIVERTICULITIS	10013538	255
OSTEOARTHRITIS	10031161	255
CHEILITIS	10008417	254
HUMAN CHORIONIC GONADOTROPIN	10071325	254
SCAN	10061498	254
HERPES SIMPLEX	10019948	253
MEAN CELL HAEMOGLOBIN NORMAL	10026997	253
RECTAL HAEMORRHAGE	10038063	253
RENAL FAILURE	10038435	253
RENAL IMPAIRMENT	10062237	253
SUICIDAL IDEATION	10042458	252
COORDINATION ABNORMAL	10010947	251
PROCALCITONIN	10064051	251
DRUG HYPERSENSITIVITY	10013700	249
LYMPHOCYTE PERCENTAGE DECREASED	10052231	249
MONOCYTE PERCENTAGE	10059473	249
SHOULDER INJURY RELATED TO VACCINE ADMINISTRATION	10081038	247
POSTURAL ORTHOSTATIC TACHYCARDIA SYNDROME	10063080	247
LIP PAIN	10024561	245
PAIN ASSESSMENT	10050533	244
IMMUNE THROMBOCYTOPENIA	10083842	244
MYDRIASIS	10028521	243
NEUTROPHIL PERCENTAGE INCREASED	10052224	243
INCONTINENCE	10021639	242
VACCINATION SITE HAEMORRHAGE	10069475	242
LIPASE	10050659	241
CIRCUMSTANCE OR INFORMATION CAPABLE OF LEADING TO M	10064385	240
EAR PRURITUS	10052138	239
BLOOD ALBUMIN DECREASED	10005287	238
VACCINATION SITE INFLAMMATION	10068878	237
RESPIRATORY VIRAL PANEL	10075165	237
NEUTROPHIL COUNT	10029363	236
BIOPSY SKIN ABNORMAL	10004874	235
MUSCLE STRAIN	10050031	235
CARDIAC DISCOMFORT	10054211	233
DIABETES MELLITUS	10012601	232
INTERNATIONAL NORMALISED RATIO NORMAL	10022596	232

BLOOD CHOLESTEROL	10005422	230
RHEUMATOID FACTOR NEGATIVE	10048574	230
MAGNETIC RESONANCE IMAGING HEART	10083127	230
FUNGAL INFECTION	10017533	229
INFLAMMATORY MARKER INCREASED	10069826	229
HYPOTHYROIDISM	10021114	228
LIPIDS	10024587	228
SCAN WITH CONTRAST NORMAL	10062153	228
VIRAL INFECTION	10047461	227
UTERINE DILATION AND CURETTAGE	10057304	227
IMAGING PROCEDURE	10068979	226
ISCHAEMIC STROKE	10061256	226
MICTURITION URGENCY	10027566	225
CARDIAC STRESS TEST NORMAL	10055063	225
BLOOD CREATININE	10005480	224
BODY HEIGHT	10005891	224
CONJUNCTIVITIS	10010741	224
MONOPLEGIA	10027926	224
NEUTROPHIL COUNT INCREASED	10029368	224
BLOOD ALBUMIN NORMAL	10005289	223
BLOOD CHOLESTEROL INCREASED	10005425	223
INTERNATIONAL NORMALISED RATIO INCREASED	10022595	223
PANIC REACTION	10033670	222
VERTIGO POSITIONAL	10047348	222
ANAL INCONTINENCE	10077605	222
CULTURE	10061447	222
GLOMERULAR FILTRATION RATE	10018355	221
VITAMIN B12 NORMAL	10047611	221
BRAIN NATRIURETIC PEPTIDE	10053406	221
HAEMATOMA	10018852	219
PHOTOSENSITIVITY REACTION	10034972	219
GLOMERULAR FILTRATION RATE NORMAL	10018361	217
HYPERGLYCAEMIA	10020635	217
SUDDEN HEARING LOSS	10061373	216
EJECTION FRACTION DECREASED	10050528	215
NERVE CONDUCTION STUDIES	10053318	215
BLOOD IMMUNOGLOBULIN G	10005593	214
BLOOD IRON DECREASED	10005619	214
EMOTIONAL DISORDER	10014551	214
GASTROINTESTINAL HAEMORRHAGE	10017955	214
ELECTROENCEPHALOGRAM NORMAL	10014409	213
GINGIVAL SWELLING	10018291	213
RASH MACULO-PAPULAR	10037868	213

BLOOD GASES	10005537	212
HYPERTHYROIDISM	10020850	212
GASTRITIS	10017853	211
CROHN'S DISEASE	10011401	210
PITYRIASIS ROSEA	10035114	210
POSTURE ABNORMAL	10036436	210
TEMPERATURE INTOLERANCE	10057040	210
MOTOR DYSFUNCTION	10061296	210
SINUS OPERATION	10062245	210
MULTIPLE SCLEROSIS	10028245	209
MUSCLE CONTRACTIONS INVOLUNTARY	10028293	209
MAGNETIC RESONANCE IMAGING SPINAL ABNORMAL	10083139	209
LIVER FUNCTION TEST NORMAL	10060106	209
ABNORMAL BEHAVIOUR	10061422	209
BLOOD CREATINE PHOSPHOKINASE NORMAL	10005479	208
CULTURE URINE NEGATIVE	10011639	208
GINGIVAL BLEEDING	10018276	208
SALIVARY HYPERSECRETION	10039424	208
HERPES VIRUS INFECTION	10019973	207
PULSE ABNORMAL	10037466	207
SKIN LACERATION	10058818	207
FREQUENT BOWEL MOVEMENTS	10017367	206
GLYCOSYLATED HAEMOGLOBIN INCREASED	10018484	206
HYPOTONIA	10021118	206
RESPIRATORY ARREST	10038669	206
TRISMUS	10044684	206
DERMATITIS ALLERGIC	10012434	205
CARDIOVERSION	10007661	204
GAZE PALSY	10056696	204
ACOUSTIC STIMULATION TESTS NORMAL	10000527	203
EYE DISCHARGE	10015915	203
SPUTUM DISCOLOURED	10041807	203
MEAN PLATELET VOLUME NORMAL	10055070	203
CEREBRAL INFARCTION	10008118	202
IRRITABLE BOWEL SYNDROME	10023003	202
NEEDLE ISSUE	10069217	202
CARDIAC MONITORING ABNORMAL	10053440	202
TRYPTASE	10063240	202
HYPOGLYCAEMIA	10020993	201
INJECTION SITE OEDEMA	10022085	201
MUSCULOSKELETAL DISORDER	10048592	200
EUPHORIC MOOD	10015535	199
HYPOMENORRHOEA	10021033	199

THROMBECTOMY	10043530	199
TRIGEMINAL NEURALGIA	10044652	199
CARDIO-RESPIRATORY ARREST	10007617	198
COSTOCHONDRITIS	10011219	197
CYST	10011732	197
ULTRASOUND ANTENATAL SCREEN ABNORMAL	10045400	197
EYELID PTOSIS	10015995	196
LYMPHOCYTE COUNT DECREASED	10025256	196
BLOOD POTASSIUM	10005721	195
HEMIPLEGIA	10019468	195
INCOHERENT	10021630	195
VACCINATION SITE LYMPHADENOPATHY	10069480	195
INJURY	10022116	194
LYMPHOCYTE COUNT	10025251	194
LIVER FUNCTION TEST INCREASED	10077692	194
BODY TEMPERATURE FLUCTUATION	10063488	193
BORRELIA TEST NEGATIVE	10070280	191
INFLAMMATORY MARKER TEST	10074736	191
LARYNGITIS	10023874	189
NERVE COMPRESSION	10029174	189
SKIN INDURATION	10051837	189
IMMUNOLOGY TEST	10062297	189
TONSILLAR HYPERTROPHY	10044003	188
BLOOD BILIRUBIN INCREASED	10005364	187
SPINAL X-RAY	10041604	187
BLOOD IRON	10005616	186
CAESAREAN SECTION	10006924	186
CHAPPED LIPS	10049047	186
FACE INJURY	10050392	186
CARBON DIOXIDE DECREASED	10007223	185
FOOD ALLERGY	10016946	185
SUPRAVENTRICULAR EXTRASYSTOLES	10042602	185
ERYTHEMA MULTIFORME	10015218	184
MUSCLE RIGIDITY	10028330	184
POLYMYALGIA RHEUMATICA	10036099	184
INFLUENZA A VIRUS TEST	10070416	184
MIDDLE EAR EFFUSION	10062545	184
MUSCLE SWELLING	10064470	183
CHRONIC KIDNEY DISEASE	10064848	183
DERMATITIS CONTACT	10012442	182
BED REST	10050118	182
FLUID INTAKE REDUCED	10056291	182
BLOOD CREATINE PHOSPHOKINASE INCREASED	10005470	181

MOOD SWINGS	10027951	181
STOOL ANALYSIS	10059536	181
CHOKING	10008589	180
CULTURE URINE POSITIVE	10011640	180
DEMENTIA	10012267	180
PULSE ABSENT	10037469	180
X-RAY LIMB NORMAL	10061587	180
SEPTIC SHOCK	10040070	179
VISUAL FIELD DEFECT	10047555	179
ANGER	10002368	178
ULTRASOUND BREAST	10045409	178
TENSION	10043268	177
VITAMIN D DECREASED	10062189	177
BLOOD THYROID STIMULATING HORMONE INCREASED	10005833	176
CONCUSSION	10010254	176
PNEUMONITIS	10035742	176
PURPURA	10037549	176
EOSINOPHIL PERCENTAGE	10059464	176
MAMMOGRAM NORMAL	10026737	175
GASTRIC DISORDER	10056819	175
X-RAY LIMB ABNORMAL	10061586	175
MIGRAINE WITH AURA	10027607	174
POSITRON EMISSION TOMOGRAM	10036220	174
EYE INFLAMMATION	10015943	173
RAYNAUD'S PHENOMENON	10037912	173
PLEURISY	10035618	172
PREMENSTRUAL SYNDROME	10036618	172
WHEELCHAIR USER	10047920	172
CHEMOTHERAPY	10061758	172
AUDIOGRAM	10003760	171
ELECTROCARDIOGRAM AMBULATORY ABNORMAL	10014369	171
LIP DRY	10024552	171
MUSCLE DISORDER	10028300	171
ANGIOGRAM NORMAL	10061638	171
POLYURIA	10036142	170
SHOCK	10040560	170
IMMATURE GRANULOCYTE COUNT	10085122	170
RED CELL DISTRIBUTION WIDTH INCREASED	10053920	170
MENTAL DISORDER	10061284	170
OVARIAN CYST	10033132	169
PHARYNGEAL ERYTHEMA	10057009	169
COMPUTERISED TOMOGRAM ABDOMEN NORMAL	10057800	169
ANAPHYLACTIC SHOCK	10002199	168

THYROID DISORDER	10043709	168
THERAPY NON-RESPONDER	10051082	168
THYROXINE FREE	10055157	168
PNEUMONIA BACTERIAL	10060946	168
COLITIS ULCERATIVE	10009900	167
VAGINAL DISCHARGE	10046901	167
MOOD ALTERED	10027940	166
ROTATOR CUFF SYNDROME	10039227	166
DRAINAGE	10084562	166
BOWEL MOVEMENT IRREGULARITY	10063541	166
PLEURITIC PAIN	10035623	165
URINARY RETENTION	10046555	165
ULTRASOUND PELVIS	10052041	165
BASOPHIL PERCENTAGE DECREASED	10052219	165
ANGIOGRAM CEREBRAL ABNORMAL	10052906	165
DEAFNESS NEUROSENSORY	10011891	164
PSORIATIC ARTHROPATHY	10037162	164
SERUM FERRITIN	10040246	164
ORAL DISORDER	10067621	164
BODY TEMPERATURE ABNORMAL	10075265	164
LYMPHOCYTE PERCENTAGE	10059905	163
LIVEDO RETICULARIS	10024648	162
RESPIRATORY RATE	10038709	162
VASCULITIS	10047115	162
BLOOD ELECTROLYTES NORMAL	10061015	162
LYMPHOCYTE COUNT NORMAL	10025260	161
MENOPAUSE	10027308	161
PROTHROMBIN TIME NORMAL	10037062	161
BREATH SOUNDS ABNORMAL	10064780	161
MONOCYTE COUNT	10027876	160
MOTION SICKNESS	10027990	160
ORAL MUCOSAL ERUPTION	10030997	160
TONGUE DISCOLOURATION	10043949	160
ULTRASOUND ABDOMEN ABNORMAL	10052039	160
ALLERGY TEST NEGATIVE	10056362	160
ANGIOGRAM ABNORMAL	10060956	160
CATHETERISATION CARDIAC ABNORMAL	10007816	159
LIPASE NORMAL	10024575	159
COVID-19 IMMUNISATION	10084457	159
BLOOD CALCIUM	10005392	158
TROPONIN I NORMAL	10073406	158
ANGIOGRAM CEREBRAL NORMAL	10052907	157
OPHTHALMOLOGICAL EXAMINATION ABNORMAL	10056836	157

METABOLIC FUNCTION TEST ABNORMAL	10061286	157
VACCINATION ERROR	10063972	157
HUNGER	10020466	156
COMPUTERISED TOMOGRAM PELVIS	10075023	156
WOUND	10052428	156
MUSCLE DISCOMFORT	10028299	155
MAGNETIC RESONANCE IMAGING SPINAL NORMAL	10083138	155
ADNEXA UTERI PAIN	10058841	155
ANGIOGRAM PULMONARY NORMAL	10002442	154
PANCREATITIS	10033645	154
RENAL DISORDER	10038428	154
EJECTION FRACTION	10050527	154
WEIGHT BEARING DIFFICULTY	10066454	154
URTICARIA CHRONIC	10052568	154
NEUTROPHIL COUNT NORMAL	10029370	153
SERUM FERRITIN INCREASED	10040250	153
EOSINOPHIL PERCENTAGE DECREASED	10052221	153
MYELITIS TRANSVERSE	10028527	152
BRAIN NATRIURETIC PEPTIDE NORMAL	10053409	152
RENAL FUNCTION TEST	10061490	152
HAEMATEMESIS	10018830	151
ENERGY INCREASED	10048779	151
ALANINE AMINOTRANSFERASE	10001546	150
BLOOD LACTIC ACID INCREASED	10005635	150
DROOLING	10013642	150
EOSINOPHIL COUNT NORMAL	10014946	150
SEASONAL ALLERGY	10048908	150
VULVOVAGINAL PAIN	10069055	150
FEAR OF INJECTION	10073753	150
VIRAL TEST	10062358	150
EJECTION FRACTION NORMAL	10064144	150
HORMONE LEVEL ABNORMAL	10061210	149
BLOOD CHLORIDE INCREASED	10005420	148
LUNG CONSOLIDATION	10025080	148
ORTHOSTATIC HYPOTENSION	10031127	148
GOUT	10018627	147
SUPPRESSED LACTATION	10042576	147
BASOPHIL COUNT DECREASED	10004167	146
BLOOD GLUCOSE ABNORMAL	10005554	146
HORDEOLUM	10020377	146
MASTICATION DISORDER	10026882	146
VOMITING PROJECTILE	10047708	146
COMPUTERISED TOMOGRAM SPINE	10081777	146

ASPARTATE AMINOTRANSFERASE	10003476	145
GOITRE	10018498	145
SITTING DISABILITY	10081807	145
ANGIOGRAM CEREBRAL	10052905	145
EYELID FUNCTION DISORDER	10061145	145
BASEDOW'S DISEASE	10004161	144
CARPAL TUNNEL SYNDROME	10007697	144
CHOLELITHIASIS	10008629	144
MYOCARDIAL NECROSIS MARKER	10075210	144
DYSPHEMIA	10054964	144
CEREBRAL THROMBOSIS	10008132	143
RED BLOOD CELL COUNT	10038150	143
HUMAN CHORIONIC GONADOTROPIN NEGATIVE	10071333	143
BACTERIAL INFECTION	10060945	143
EAR DISORDER	10014004	142
MONOCYTE COUNT INCREASED	10027880	142
UVEITIS	10046851	142
AUTOIMMUNE THYROIDITIS	10049046	142
NEAR DEATH EXPERIENCE	10068111	142
INFUSION	10060345	142
SKIN ABRASION	10064990	142
BRADYKINESIA	10006100	141
BRONCHOSPASM	10006482	141
INJECTION SITE HYPERSENSITIVITY	10022071	141
KIDNEY INFECTION	10023424	141
MONOCYTE COUNT NORMAL	10027882	141
BLOOD POTASSIUM INCREASED	10005725	140
MITRAL VALVE INCOMPETENCE	10027727	140
PUSTULE	10037578	140
VENTRICULAR TACHYCARDIA	10047302	140
FOETAL DEATH	10055690	140
ACUTE RESPIRATORY DISTRESS SYNDROME	10001052	139
LUMBAR PUNCTURE ABNORMAL	10025000	139
SCREAMING	10039740	139
SMEAR CERVIX	10041205	139
AUTOPSY	10050117	139
BACTERIAL TEST	10068074	139
BASOPHIL PERCENTAGE	10059471	139
CARDIAC PACEMAKER INSERTION	10007598	138
NEUTROPHIL COUNT DECREASED	10029366	138
PAPULE	10033733	138
SKIN SENSITISATION	10070835	138
CEREBRAL VENOUS SINUS THROMBOSIS	10083037	138

SARS-COV-2 RNA	10085493	138
BLOOD CHLORIDE DECREASED	10005419	137
HYPERAESTHESIA TEETH	10082426	137
BREAST CANCER FEMALE	10057654	137
COAGULOPATHY	10009802	136
THYROXINE	10043813	136
TONGUE BLISTERING	10043942	136
EOSINOPHIL COUNT DECREASED	10014943	135
MYOSITIS	10028653	135
VACCINATION SITE NODULE	10069616	135
BLOOD IMMUNOGLOBULIN M	10005598	134
HAEMATOCRIT	10018837	134
MEDICATION ERROR	10027091	134
VASODILATATION	10047141	134
NUCLEIC ACID TEST	10083356	134
DIABETIC KETOACIDOSIS	10012671	133
UTERINE SPASM	10046823	133
WHITE BLOOD CELLS URINE POSITIVE	10047967	133
SINUS RHYTHM	10048815	133
ATAXIA	10003591	132
AUDIOGRAM ABNORMAL	10003761	132
ELECTROMYOGRAM NORMAL	10014432	132
HYPOAESTHESIA EYE	10020939	132
INTERVERTEBRAL DISC PROTRUSION	10050296	132
LIP ERYTHEMA	10080124	132
THYROXINE FREE NORMAL	10055159	132
EAR, NOSE AND THROAT EXAMINATION	10056807	132
PILOERECTION	10035039	131
UTERINE LEIOMYOMA	10046798	131
SYSTEMIC INFLAMMATORY RESPONSE SYNDROME	10051379	131
CARDIAC MONITORING NORMAL	10053439	131
METABOLIC ENCEPHALOPATHY	10062190	131
STRIDOR	10042241	130
BORRELIA TEST	10070279	130
PROTEIN URINE PRESENT	10053123	130
NEUTROPHIL PERCENTAGE	10059467	130
ABNORMAL SENSATION IN EYE	10000173	129
GLYCOSYLATED HAEMOGLOBIN NORMAL	10018485	129
NYSTAGMUS	10029864	129
RASH MORBILLIFORM	10037870	129
SKIN TEST	10040929	129
TEARFULNESS	10043169	129
ULTRASOUND ABDOMEN NORMAL	10052040	129

REFUSAL OF TREATMENT BY PATIENT	10056407	129
NEUROLOGICAL EXAMINATION NORMAL	10056831	129
RENAL FUNCTION TEST NORMAL	10061491	129
BLOOD SODIUM	10005799	128
BREAST DISCOMFORT	10049872	128
AUTONOMIC NERVOUS SYSTEM IMBALANCE	10003840	127
BLOOD UREA	10005845	127
MONOCYTE PERCENTAGE INCREASED	10052230	127
VIRAL TEST NEGATIVE	10062362	127
ATRIAL FLUTTER	10003662	126
DECREASED ACTIVITY	10011953	126
DIARRHOEA HAEMORRHAGIC	10012741	126
ROAD TRAFFIC ACCIDENT	10039203	126
SLOW SPEECH	10071299	126
UPPER RESPIRATORY TRACT CONGESTION	10052252	126
CARDIAC FUNCTION TEST	10058470	126
BREAST FEEDING	10006247	125
GLOSSITIS	10018386	125
CATHETERISATION CARDIAC NORMAL	10007817	124
PNEUMOTHORAX	10035759	124
PATHOLOGY TEST	10068056	124
RESPIRATORY SYNCYTIAL VIRUS TEST	10068562	124
CHRONIC SPONTANEOUS URTICARIA	10072757	124
THYROID MASS	10058900	124
BLOOD UREA NITROGEN/CREATININE RATIO	10059899	124
BLOOD BILIRUBIN	10005362	123
COLONOSCOPY ABNORMAL	10010008	123
ERYTHEMA OF EYELID	10015237	123
FACE OEDEMA	10016029	123
STARING	10041953	123
VENOGRAM	10047208	123
DERMATITIS ACNEIFORM	10012432	122
FOETAL HEART RATE ABNORMAL	10051139	122
INTENTIONAL PRODUCT USE ISSUE	10076308	122
ARTHROPOD BITE	10003399	121
ELECTROMYOGRAM ABNORMAL	10014431	121
LACTIC ACIDOSIS	10023676	121
SUNBURN	10042496	121
EXPOSURE VIA SKIN CONTACT	10071430	121
MASTITIS	10026883	120
ULTRASOUND FOETAL	10053140	120
INJECTION SITE INFECTION	10022076	119
TONGUE ERYTHEMA	10079075	119

INJECTION SITE INDENTATION	10079277	119
LUMBAR PUNCTURE NORMAL	10025002	118
TONGUE ULCERATION	10043991	118
VEIN DISORDER	10047184	118
MILIARIA	10027627	117
IMPAIRED QUALITY OF LIFE	10076936	117
EXPOSURE VIA BREAST MILK	10080751	117
STAPHYLOCOCCAL INFECTION	10058080	117
TROPONIN I INCREASED	10058268	117
NITRITE URINE ABSENT	10060799	117
TENDON PAIN	10066371	117
GENITAL HERPES	10018150	116
INTERSTITIAL LUNG DISEASE	10022611	116
MEAN CELL HAEMOGLOBIN CONCENTRATION DECREASED	10026991	116
RHABDOMYOLYSIS	10039020	116
SUBARACHNOID HAEMORRHAGE	10042316	116
URINE LEUKOCYTE ESTERASE POSITIVE	10050795	116
EYELIDS PRURITUS	10051627	116
EYELID RASH	10074620	116
COAGULATION TEST	10063556	116
ARTERIOGRAM CAROTID NORMAL	10003196	115
BLOOD LACTIC ACID NORMAL	10005636	115
NEOPLASM MALIGNANT	10028997	115
BRAIN OEDEMA	10048962	115
PROCALCITONIN INCREASED	10067081	115
ANTINUCLEAR ANTIBODY INCREASED	10064726	115
CHOLECYSTECTOMY	10008611	114
HIV TEST NEGATIVE	10020187	114
INITIAL INSOMNIA	10022035	114
PROTHROMBIN TIME PROLONGED	10037063	114
TRIGGER FINGER	10044654	114
MEAN PLATELET VOLUME INCREASED	10055052	114
ANTI-THYROID ANTIBODY	10060325	114
DIALYSIS	10061105	114
ALOPECIA AREATA	10001761	113
BLINDNESS TRANSIENT	10005184	113
CARDIOMYOPATHY	10007636	113
LYME DISEASE	10025169	113
MONONUCLEOSIS HETEROPHILE TEST NEGATIVE	10027921	113
COMMUNICATION DISORDER	10061046	113
EYE INFECTION	10015929	112
HYPERKALAEMIA	10020646	112
MUSCLE ATROPHY	10028289	112

CANDIDA INFECTION	10074170	112
TYPE IV HYPERSENSITIVITY REACTION	10053613	112
SCAN BRAIN	10061500	112
ALTERED STATE OF CONSCIOUSNESS	10001854	111
BLOOD FOLATE	10005524	111
HIATUS HERNIA	10020028	111
PLATELET TRANSFUSION	10035543	111
CORONARY ARTERIAL STENT INSERTION	10052086	111
TYPE 2 DIABETES MELLITUS	10067585	111
FRUSTRATION TOLERANCE DECREASED	10077753	111
BASOPHIL COUNT NORMAL	10004170	110
HEPATIC STEATOSIS	10019708	110
ROSACEA	10039218	110
TEMPOROMANDIBULAR JOINT SYNDROME	10043220	110
EYE CONTUSION	10073354	110
FURUNCLE	10017553	109
HALLUCINATION, VISUAL	10019075	109
OPHTHALMIC MIGRAINE	10030867	109
PREGNANCY TEST POSITIVE	10036575	109
RASH PUSTULAR	10037888	109
THROMBOPHLEBITIS SUPERFICIAL	10043595	109
STENT PLACEMENT	10048561	109
HERPES SIMPLEX TEST NEGATIVE	10077970	109
SKIN WEEPING	10080560	109
METAMORPHOPSIA	10063341	109
HIV TEST	10020185	108
VARICOSE VEIN	10046996	108
ABNORMAL FAECES	10000133	107
AURA	10003791	107
BLOOD CULTURE POSITIVE	10005488	107
CATARACT	10007739	107
MYASTHENIA GRAVIS	10028417	107
PREMATURE LABOUR	10036600	107
PROTEIN TOTAL DECREASED	10037014	107
URINE LEUKOCYTE ESTERASE	10050413	107
COMPLEMENT FACTOR C4	10050963	107
CEREBRAL DISORDER	10054938	107
PULSELESS ELECTRICAL ACTIVITY	10058151	107
EYELID DISORDER	10061130	107
BACTERIAL TEST NEGATIVE	10065004	107
BODY MASS INDEX	10005894	106
CARDIAC FAILURE ACUTE	10007556	106
DELUSION	10012239	106

DISSOCIATION	10013457	106
ELECTROENCEPHALOGRAM ABNORMAL	10014408	106
NIPPLE PAIN	10029421	106
POOR PERIPHERAL CIRCULATION	10036155	106
PSYCHOMOTOR HYPERACTIVITY	10037211	106
JOINT NOISE	10074329	106
INTERVERTEBRAL DISC DEGENERATION	10061246	106
ABDOMINAL X-RAY	10061612	106
BLOOD ALKALINE PHOSPHATASE	10005298	105
CORONARY ARTERY DISEASE	10011078	105
PHARYNGITIS	10034835	105
TRICUSPID VALVE INCOMPETENCE	10044640	105
VIBRATORY SENSE INCREASED	10068327	105
HYPORESPONSIVE TO STIMULI	10071552	105
ULTRASOUND FOETAL ABNORMAL	10077578	105
RED BLOOD CELL NUCLEATED MORPHOLOGY	10080979	105
EYELID PAIN	10059208	105
APPETITE DISORDER	10060961	105
AGGRESSION	10001488	104
BLADDER CATHETERISATION	10005028	104
BLOOD LACTATE DEHYDROGENASE INCREASED	10005630	104
MEAN CELL VOLUME INCREASED	10027004	104
PREMATURE DELIVERY	10036595	104
SARS-COV-1 TEST	10084444	104
BLOOD PHOSPHORUS	10005717	103
LICHEN PLANUS	10024429	103
NASAL DRYNESS	10028740	103
SJOGREN'S SYNDROME	10040767	103
TIC	10043833	103
FINE MOTOR SKILL DYSFUNCTION	10076288	103
CARDIAC STRESS TEST ABNORMAL	10055014	103
BACTERIAL TEST POSITIVE	10059421	103
GENERAL SYMPTOM	10060891	103
BLOOD MAGNESIUM DECREASED	10005654	102
METABOLIC ACIDOSIS	10027417	102
MOANING	10027783	102
PULMONARY HYPERTENSION	10037400	102
LIP DISORDER	10048470	102
PROTEIN TOTAL	10050537	102
MULTIPLE ORGAN DYSFUNCTION SYNDROME	10077361	102
CARDIAC MURMUR	10007586	101
CONJUNCTIVAL HAEMORRHAGE	10010719	101
ESSENTIAL HYPERTENSION	10015488	101

SEDATION	10039897	101
SPECIFIC GRAVITY URINE NORMAL	10041440	101
PROCALCITONIN NORMAL	10077831	101
DEAFNESS BILATERAL	10052556	101
HOSPITALISATION	10054112	101
CLUSTER HEADACHE	10059133	101
IMMOBILE	10021417	100
SERUM FERRITIN NORMAL	10040251	100
SPLENOMEGALY	10041660	100
VASCULAR PAIN	10047095	100
DISCHARGE	10080907	100
GENERAL PHYSICAL CONDITION ABNORMAL	10058911	100
BLOOD ELECTROLYTES	10061013	100
JAW DISORDER	10061257	100
OBSTRUCTIVE AIRWAYS DISORDER	10061877	100
GLUCOSE TOLERANCE TEST	10018432	99
OPTIC NEURITIS	10030942	99
SKIN ULCER	10040943	99
ARTERIOGRAM CAROTID	10003194	98
DISABILITY	10013050	98
ELECTROLYTE IMBALANCE	10014418	98
HYPOGEUSIA	10020989	98
INJECTION SITE EXTRAVASATION	10022062	98
ORAL CANDIDIASIS	10030963	98
BRADYPHRENIA	10050012	98
TREPONEMA TEST NEGATIVE	10070403	98
PARANASAL SINUS HYPERSECRETION	10057392	98
MUSCLE CONTRACTURE	10062575	98
BLOOD UREA DECREASED	10005850	97
IMPAIRED HEALING	10021519	97
LEUKOPENIA	10024384	97
PHARYNGITIS STREPTOCOCCAL	10034839	97
PULMONARY FIBROSIS	10037383	97
COMPLEMENT FACTOR C3	10050962	97
RHINALGIA	10051496	97
CHLAMYDIA TEST NEGATIVE	10070273	97
SMALL FIBRE NEUROPATHY	10073928	97
DEFAECATION URGENCY	10012110	96
LIPASE INCREASED	10024574	96
THYROID FUNCTION TEST ABNORMAL	10043730	96
URINE ABNORMALITY	10046607	96
ULTRASOUND THYROID	10049501	96
HEPATIC ENZYME	10060793	96

BIOPSY BONE MARROW	10004737	95
ELECTROCARDIOGRAM AMBULATORY NORMAL	10014370	95
LOCALISED INFECTION	10024774	95
PANCREATITIS ACUTE	10033647	95
PARANOIA	10033864	95
WHITE MATTER LESION	10072731	95
URINE OUTPUT DECREASED	10059895	95
PH URINE NORMAL	10034797	94
ADENOVIRUS TEST	10050991	94
SYMPTOM RECURRENCE	10080080	94
ABDOMINAL TENDERNESS	10000097	93
BLOOD BLISTER	10005372	93
LIGAMENT SPRAIN	10024453	93
LIVER DISORDER	10024670	93
LYMPHOCYTE COUNT INCREASED	10025258	93
PNEUMONIA VIRAL	10035737	93
UTERINE CONTRACTIONS DURING PREGNANCY	10049975	93
GYNAECOLOGICAL EXAMINATION	10050317	93
CENTRAL VENOUS CATHETERISATION	10053377	93
ANTIBODY TEST NEGATIVE	10061426	93
CULTURE NEGATIVE	10061448	93
BLOOD BICARBONATE DECREASED	10005359	92
CHOKING SENSATION	10008590	92
HYPERPYREXIA	10020741	92
MOUTH HAEMORRHAGE	10028024	92
VENTILATION/PERFUSION SCAN	10047264	92
VESTIBULAR NEURONITIS	10047393	92
CENTRAL NERVOUS SYSTEM LESION	10051290	92
CARDIOVASCULAR EVALUATION	10053046	92
CARDIAC ABLATION	10059864	92
ACTIVATED PARTIAL THROMBOPLASTIN TIME NORMAL	10000635	91
CULTURE STOOL	10011629	91
EOSINOPHIL COUNT INCREASED	10014945	91
GLOBULIN	10018342	91
HYPERVOLAEMIA	10020919	91
UTERINE HAEMORRHAGE	10046788	91
STREPTOCOCCUS TEST POSITIVE	10070055	91
BILIRUBIN URINE	10053113	91
INJECTION SITE MOVEMENT IMPAIRMENT	10056250	91
URINE ODOUR ABNORMAL	10057135	91
ENLARGED UVULA	10066192	91
ALBUMIN GLOBULIN RATIO	10001562	90
BLOOD LACTATE DEHYDROGENASE	10005626	90

GASTROENTERITIS VIRAL	10017918	90
SINUS BRADYCARDIA	10040741	90
ULTRASOUND ANTENATAL SCREEN	10045399	90
INJECTION SITE JOINT PAIN	10049261	90
IMAGING PROCEDURE ABNORMAL	10077446	90
ACUPUNCTURE	10000646	89
BLADDER PAIN	10005063	89
HIP FRACTURE	10020100	89
LIPIDS NORMAL	10024593	89
LOWER RESPIRATORY TRACT INFECTION	10024968	89
BRAIN INJURY	10067967	89
VACCINATION SITE VESICLES	10069623	89
CARDIAC TELEMETRY	10053448	89
SUPERFICIAL VEIN THROMBOSIS	10086210	89
GLUCOSE URINE ABSENT	10018474	88
HYPOTHERMIA	10021113	88
RED BLOOD CELL COUNT INCREASED	10038155	88
RED BLOOD CELLS URINE POSITIVE	10038182	88
SLEEP APNOEA SYNDROME	10040979	88
ULCER	10045285	88
ULTRASOUND BREAST ABNORMAL	10045410	88
INCREASED UPPER AIRWAY SECRETION	10062717	88
BLOOD ALBUMIN	10005285	87
BUNDLE BRANCH BLOCK RIGHT	10006582	87
EMPHYSEMA	10014561	87
GALLBLADDER DISORDER	10017626	87
HEPATIC PAIN	10019705	87
NONINFECTIVE GINGIVITIS	10074863	87
JOINT LOCK	10061258	87
ABNORMAL UTERINE BLEEDING	10085424	87
BREAST CYST	10006220	86
DIFFERENTIAL WHITE BLOOD CELL COUNT ABNORMAL	10012785	86
INFECTIOUS MONONUCLEOSIS	10021914	86
PANCYTOPENIA	10033661	86
X-RAY OF PELVIS AND HIP	10048159	86
TYPE 1 DIABETES MELLITUS	10067584	86
MYOCARDIAL NECROSIS MARKER NORMAL	10075212	86
NASAL PRURITUS	10076406	86
ECCHYMOSIS	10014080	85
HANGOVER	10019133	85
HYSTERECTOMY	10021151	85
NAIL DISCOLOURATION	10028692	85
TREPONEMA TEST	10070401	85

SPECIALIST CONSULTATION	10076498	85
MYCOPLASMA TEST NEGATIVE	10078590	85
NEUROLOGICAL EXAMINATION ABNORMAL	10056832	85
PSYCHOTIC DISORDER	10061920	85
SKIN HAEMORRHAGE	10064265	85
HEPATOMEGALY	10019842	84
LEFT VENTRICULAR FAILURE	10024119	84
RALES	10037833	84
SKIN DISCOMFORT	10040830	84
SKIN INFECTION	10040872	84
VESTIBULAR DISORDER	10047386	84
ALLERGY TEST POSITIVE	10056352	84
URINE KETONE BODY ABSENT	10057596	84
STOOL ANALYSIS NORMAL	10059537	84
APPENDICITIS PERFORATED	10003012	83
BLOOD FOLATE NORMAL	10005529	83
BLOOD PRESSURE NORMAL	10005754	83
BRUXISM	10006514	83
ERYTHEMA NODOSUM	10015226	83
HAEMORRHOIDS	10019022	83
JAUNDICE	10023126	83
MEAN CELL HAEMOGLOBIN INCREASED	10026996	83
NEURALGIC AMYOTROPHY	10029229	83
PRE-ECLAMPSIA	10036485	83
ACTIVATED PARTIAL THROMBOPLASTIN TIME SHORTENED	10000637	82
BREAST CANCER	10006187	82
GRANULOMA ANNULARE	10018692	82
HYPOMAGNESAEMIA	10021027	82
ULTRASOUND PELVIS NORMAL	10052043	82
GLASSY EYES	10072465	82
FAECES SOFT	10074859	82
THERMAL BURN	10053615	82
HAEMORRHAGE URINARY TRACT	10055847	82
DYSGRAPHIA	10058319	82
BILEVEL POSITIVE AIRWAY PRESSURE	10064530	82
ABSCESS	10000269	81
ATRIOVENTRICULAR BLOCK	10003671	81
BACK DISORDER	10003983	81
CSF PROTEIN INCREASED	10011575	81
INCREASED APPETITE	10021654	81
INDUCED LABOUR	10021718	81
VITREOUS DETACHMENT	10047650	81
ESCHERICHIA TEST POSITIVE	10070090	81

ENTEROVIRUS TEST NEGATIVE	10070397	81
GASTROINTESTINAL TUBE INSERTION	10053050	81
TRANSAMINASES INCREASED	10054889	81
LAPAROSCOPIC SURGERY	10058468	81
FOOD INTOLERANCE	10061958	81
HYPERLIPIDAEMIA	10062060	81
ARTERIOSCLEROSIS	10003210	80
OBESITY	10029883	80
ULTRASOUND KIDNEY	10045421	80
MULTIPLE SCLEROSIS RELAPSE	10048393	80
ANTIPHOSPHOLIPID ANTIBODIES	10058341	80
BLOOD GASES ABNORMAL	10005539	79
IRON DEFICIENCY ANAEMIA	10022972	79
LIP DISCOLOURATION	10024549	79
PO2 DECREASED	10035768	79
SLOW RESPONSE TO STIMULI	10041045	79
SPINAL OSTEOARTHRITIS	10041591	79
SYNOVIAL CYST	10042858	79
THYROIDITIS	10043778	79
HUMAN METAPNEUMOVIRUS TEST	10072858	79
HUMAN RHINOVIRUS TEST	10075163	79
CARDIAC IMAGING PROCEDURE ABNORMAL	10053453	79
ABNORMAL LOSS OF WEIGHT	10000159	78
ASPIRATION	10003504	78
BIOPSY BREAST	10004744	78
HALLUCINATION, AUDITORY	10019070	78
HIDRADENITIS	10020040	78
INJECTION SITE HAEMATOMA	10022066	78
JOINT EFFUSION	10023215	78
MEAN CELL HAEMOGLOBIN DECREASED	10026995	78
MIDDLE INSOMNIA	10027590	78
NUCLEATED RED CELLS	10029825	78
SMALL INTESTINAL OBSTRUCTION	10041101	78
VITILIGO	10047642	78
PRENATAL SCREENING TEST	10072060	78
INTENTIONAL DOSE OMISSION	10079221	78
URINE KETONE BODY PRESENT	10057597	78
ANTIBODY TEST POSITIVE	10061427	78
FACIAL SPASM	10063006	78
ASCITES	10003445	77
BLOOD IRON NORMAL	10005621	77
BLOOD PH DECREASED	10005706	77
BREAST ENLARGEMENT	10006242	77

HAEMOGLOBIN INCREASED	10018888	77
SKIN ODOUR ABNORMAL	10040904	77
TRI-IODOTHYRONINE	10044591	77
VITAMIN B12 DECREASED	10047608	77
TONGUE DRY	10049713	77
ANTI-CYCLIC CITRULLINATED PEPTIDE ANTIBODY	10068810	77
MAST CELL ACTIVATION SYNDROME	10075217	77
UROBILINOGEN URINE	10059506	77
BLOOD COUNT	10064196	77
BIOPSY BREAST ABNORMAL	10004745	76
BIOPSY LYMPH GLAND	10004797	76
CORONARY ARTERY OCCLUSION	10011086	76
ELECTROCARDIOGRAM ST SEGMENT ELEVATION	10014392	76
INTRACRANIAL ANEURYSM	10022758	76
JOINT DISLOCATION	10023204	76
PNEUMONIA ASPIRATION	10035669	76
STRESS ECHOCARDIOGRAM	10050241	76
DOUBLE STRANDED DNA ANTIBODY	10050418	76
QUALITY OF LIFE DECREASED	10067620	76
INJURY ASSOCIATED WITH DEVICE	10069803	76
CEREBRAL SMALL VESSEL ISCHAEMIC DISEASE	10070878	76
N-TERMINAL PROHORMONE BRAIN NATRIURETIC PEPTIDE INC	10071662	76
BLADDER DISORDER	10061011	76
BLOOD BICARBONATE NORMAL	10005361	75
BUNDLE BRANCH BLOCK LEFT	10006580	75
INJECTION SITE ABSCESS	10022044	75
RIB FRACTURE	10039117	75
STRESS CARDIOMYOPATHY	10066286	75
COLONOSCOPY NORMAL	10010011	74
EOSINOPHIL COUNT	10014941	74
EPSTEIN-BARR VIRUS TEST	10050681	74
LIP INJURY	10055082	74
ASPIRATION PLEURAL CAVITY	10003522	73
ATYPICAL PNEUMONIA	10003757	73
EPILEPSY	10015037	73
PERONEAL NERVE PALSY	10034701	73
PROTEIN URINE	10037018	73
LEFT VENTRICULAR HYPERTROPHY	10049773	73
ORAL MUCOSAL ERYTHEMA	10067418	73
BREAST DISCHARGE	10006223	72
ELECTROCARDIOGRAM T WAVE INVERSION	10014395	72
INCREASED TENDENCY TO BRUISE	10021688	72
MYOCLONUS	10028622	72

NEOPLASM	10028980	72
PERIORBITAL OEDEMA	10034545	72
READING DISORDER	10037999	72
SPINAL X-RAY ABNORMAL	10041605	72
SUBDURAL HAEMATOMA	10042361	72
VAGINAL ULCERATION	10046943	72
BORDETELLA TEST NEGATIVE	10070278	72
OPHTHALMOLOGICAL EXAMINATION NORMAL	10056835	72
ENDOSCOPY ABNORMAL	10061838	72
GASTROINTESTINAL INFLAMMATION	10064147	72
CARBON DIOXIDE	10007220	71
DEMYELINATION	10012305	71
LISTLESS	10024642	71
RETINAL VEIN OCCLUSION	10038907	71
VITAMIN D DEFICIENCY	10047626	71
ELECTROCARDIOGRAM T WAVE ABNORMAL	10050380	71
DRUG SCREEN NEGATIVE	10050895	71
VACCINATION SITE CELLULITIS	10069559	71
AUTOSCOPY	10074080	71
LOWER RESPIRATORY TRACT CONGESTION	10075565	71
DISORGANISED SPEECH	10076227	71
BLOOD CHLORIDE	10005416	70
CARBON DIOXIDE INCREASED	10007225	70
MENIERE'S DISEASE	10027183	70
PACKED RED BLOOD CELL TRANSFUSION	10033359	70
THYROIDITIS SUBACUTE	10043784	70
TOOTH DISORDER	10044034	70
TONGUE ERUPTION	10052002	70
VACCINATION SITE PARAESTHESIA	10069481	70
SEXUALLY TRANSMITTED DISEASE TEST	10084201	70
OESOPHAGOGASTRODUODENOSCOPY	10053057	70
INJECTION SITE LYMPHADENOPATHY	10057665	70
ESCHERICHIA INFECTION	10061126	70
TEMPERATURE REGULATION DISORDER	10061376	70
HEAD TITUBATION	10064950	70
BLOOD URIC ACID	10005858	69
EPSTEIN-BARR VIRUS INFECTION	10015108	69
HAEMORRHAGIC STROKE	10019016	69
PROTEINURIA	10037032	69
SLEEP DEFICIT	10080881	69
COMPUTERISED TOMOGRAM PELVIS ABNORMAL	10081333	69
VULVOVAGINAL PRURITUS	10056530	69
RHEUMATOID FACTOR INCREASED	10057962	69

BLOOD IMMUNOGLOBULIN A	10005583	68
GENERALISED OEDEMA	10018092	68
HAEMODIALYSIS	10018875	68
IMPAIRED GASTRIC EMPTYING	10021518	68
PREGNANCY TEST URINE NEGATIVE	10036577	68
SUPERINFECTION	10042566	68
TRANSIENT GLOBAL AMNESIA	10044380	68
LEFT VENTRICULAR DYSFUNCTION	10049694	68
BLOOD GLUCOSE FLUCTUATION	10049803	68
ANTINEUTROPHIL CYTOPLASMIC ANTIBODY	10050552	68
DRUG SCREEN	10050837	68
BLOOD DISORDER	10061590	68
EXFOLIATIVE RASH	10064579	68
CARDIOGENIC SHOCK	10007625	67
HAEMATOCRIT INCREASED	10018840	67
HYPERTENSIVE CRISIS	10020802	67
LIVER FUNCTION TEST ABNORMAL	10024690	67
THYROXINE NORMAL	10043820	67
EYELID MARGIN CRUSTING	10052132	67
STREPTOCOCCAL INFECTION	10061372	67
ULTRASOUND BILIARY TRACT	10064146	67
APATHY	10002942	66
DIPLEGIA	10013033	66
SKIN EROSION	10040840	66
UTERINE PAIN	10046809	66
URINARY SEDIMENT PRESENT	10049821	66
HYPOSMIA	10050515	66
NEUTROPHIL PERCENTAGE DECREASED	10052223	66
SLEEP STUDY	10053319	66
TROPONIN T	10058266	66
ULTRASOUND THYROID ABNORMAL	10060987	66
INJECTION SITE SCAB	10066210	66
ANGIOGRAM PULMONARY	10002440	65
BLOOD URINE	10005863	65
STILLBIRTH	10042062	65
TOOTH INFECTION	10048762	65
VACCINATION SITE IRRITATION	10069479	65
COLITIS MICROSCOPIC	10056979	65
MALNUTRITION	10061273	65
ARTERIOSCLEROSIS CORONARY ARTERY	10003211	64
BLOOD CALCIUM INCREASED	10005396	64
DYSTONIA	10013983	64
ELECTROCARDIOGRAM QT PROLONGED	10014387	64

HYPERCAPNIA	10020591	64
OSTEOPOROSIS	10031282	64
PROTEIN TOTAL INCREASED	10037016	64
PROTEIN URINE ABSENT	10037033	64
RESPIRATORY RATE DECREASED	10038710	64
RETINAL ARTERY OCCLUSION	10038827	64
TONGUE BITING	10050467	64
GASTROINTESTINAL SOUNDS ABNORMAL	10067715	64
PALATAL SWELLING	10074403	64
TILT TABLE TEST	10052700	64
HYDRONEPHROSIS	10020524	63
NERVE CONDUCTION STUDIES NORMAL	10029176	63
NEUTROPENIA	10029354	63
VULVAL ULCERATION	10047768	63
CONSCIOUSNESS FLUCTUATING	10050093	63
BLOOD LOSS ANAEMIA	10082297	63
MAGNETIC RESONANCE IMAGING BRAIN	10083128	63
FOAMING AT MOUTH	10062654	63
BLOOD PH NORMAL	10005709	62
CARDIOLIPIN ANTIBODY	10007628	62
CHOLECYSTITIS	10008612	62
FEMUR FRACTURE	10016454	62
RADIOTHERAPY	10037794	62
N-TERMINAL PROHORMONE BRAIN NATRIURETIC PEPTIDE	10067803	62
IMPAIRED SELF-CARE	10052404	62
LYMPH NODE PALPABLE	10057470	62
HEPATIC ENZYME ABNORMAL	10062685	62
AORTIC ARTERIOSCLEROSIS	10065558	62
BACTERAEMIA	10003997	61
KNEE ARTHROPLASTY	10023469	61
MANIA	10026749	61
MEAN CELL VOLUME DECREASED	10027002	61
NORMAL LABOUR	10029767	61
VULVOVAGINAL DISCOMFORT	10047786	61
CHEST TUBE INSERTION	10050522	61
PROHORMONE BRAIN NATRIURETIC PEPTIDE INCREASED	10077781	61
BONE SWELLING	10053631	61
CLOSTRIDIUM DIFFICILE INFECTION	10054236	61
COAGULATION TEST NORMAL	10063558	61
ANION GAP DECREASED	10002526	60
ARTERIOGRAM CAROTID ABNORMAL	10003195	60
BIOPSY KIDNEY	10004782	60
BLOOD FIBRINOGEN	10005517	60

BLOOD FOLLICLE STIMULATING HORMONE	10005531	60
BLOOD IMMUNOGLOBULIN E	10005588	60
CREPITATIONS	10011376	60
INTESTINAL OBSTRUCTION	10022687	60
LIFE SUPPORT	10024447	60
LYMPHOMA	10025310	60
MEAN CELL HAEMOGLOBIN	10026989	60
NASAL OEDEMA	10028750	60
OESOPHAGITIS	10030216	60
OTORRHOEA	10033101	60
POSITRON EMISSION TOMOGRAM ABNORMAL	10036221	60
TONSILLITIS	10044008	60
INTERNAL HAEMORRHAGE	10075192	60
EYELID IRRITATION	10057385	60
CULTURE POSITIVE	10061449	60
SCAN NORMAL	10061505	60
PULMONARY FUNCTION TEST NORMAL	10061923	60
RESPIRATORY TRACT INFECTION	10062352	60
LIP EXFOLIATION	10064482	60
TACHYPHRENIA	10064805	60
INJECTION SITE PAPULE	10066044	60
BLOOD CREATINE	10005461	59
BLOOD OSMOLARITY DECREASED	10005696	59
BLOOD SODIUM INCREASED	10005803	59
BREAST INFLAMMATION	10006262	59
CARDIOVASCULAR DISORDER	10007649	59
FAILURE TO THRIVE	10016165	59
HAEMORRHAGE IN PREGNANCY	10018981	59
HIP ARTHROPLASTY	10020096	59
MUSCLE SPASTICITY	10028335	59
ORTHOPNOEA	10031123	59
PLANTAR FASCIITIS	10035155	59
SERUM FERRITIN DECREASED	10040249	59
SMEAR CERVIX NORMAL	10041207	59
CLOSTRIDIUM TEST	10070270	59
LABOUR INDUCTION	10052856	59
ABDOMINAL RIGIDITY	10000090	58
ANKLE FRACTURE	10002544	58
ANKYLOSING SPONDYLITIS	10002556	58
CLUMSINESS	10009696	58
LABYRINTHITIS	10023567	58
NERVE CONDUCTION STUDIES ABNORMAL	10029175	58
OPHTHALMIC HERPES ZOSTER	10030865	58

PCO2 INCREASED	10034183	58
REACTION TO PREVIOUS EXPOSURE TO ANY VACCINE	10066904	58
ACCIDENTAL EXPOSURE TO PRODUCT	10073317	58
MYOCARDIAL NECROSIS MARKER INCREASED	10075211	58
VACCINATION SITE HYPOAESTHESIA	10076168	58
MULTISYSTEM INFLAMMATORY SYNDROME IN CHILDREN	10084767	58
ULTRASOUND CHEST	10052962	58
CARDIAC FUNCTION TEST NORMAL	10058478	58
ENDOSCOPY NORMAL	10061840	58
HEPATOBILIARY SCAN	10061946	58
RED BLOOD CELL TRANSFUSION	10087283	58
ANION GAP NORMAL	10002530	57
BACK INJURY	10003986	57
BONE DISORDER	10005956	57
HEART RATE NORMAL	10019306	57
HYPOVOLAEMIA	10021137	57
IRITIS	10022955	57
MEAN CELL HAEMOGLOBIN CONCENTRATION	10026990	57
PHLEBITIS	10034879	57
RENAL CYST	10038423	57
TENDON DISORDER	10043239	57
INTRACARDIAC THROMBUS	10048620	57
LOCALISED OEDEMA	10048961	57
BLOOD BILIRUBIN DECREASED	10049869	57
NO REACTION ON PREVIOUS EXPOSURE TO DRUG	10052053	57
OCCUPATIONAL EXPOSURE TO SARS-COV-2	10084394	57
PARTIAL SEIZURES	10061334	57
CYTOMEGALOVIRUS TEST	10061806	57
CARDITIS	10062746	57
BLOOD CORTISOL	10005455	56
BLOOD IMMUNOGLOBULIN G NORMAL	10005597	56
BLOOD PRESSURE SYSTOLIC INCREASED	10005760	56
DRUG INTERACTION	10013710	56
MONONUCLEOSIS HETEROPHILE TEST	10027920	56
MUCOUS STOOLS	10028140	56
OXYGEN SATURATION NORMAL	10033322	56
VARICELLA	10046980	56
VENOGRAM NORMAL	10047210	56
GRANULOCYTE PERCENTAGE	10068913	56
FOETAL GROWTH RESTRICTION	10070531	56
OESOPHAGOGASTRODUODENOSCOPY ABNORMAL	10072163	56
HEPATITIS VIRAL TEST NEGATIVE	10072747	56
CORONAVIRUS TEST NEGATIVE	10084269	56

HAEMATOLOGY TEST	10053076	56
SEROLOGY TEST	10062163	56
BLOOD TRIGLYCERIDES INCREASED	10005839	55
CHRONIC FATIGUE SYNDROME	10008874	55
COLITIS ISCHAEMIC	10009895	55
COMA	10010071	55
CONTRACEPTION	10010808	55
ELECTROCARDIOGRAM ST SEGMENT DEPRESSION	10014391	55
ELECTROPHORESIS PROTEIN	10014467	55
GENITAL ULCERATION	10018180	55
LOW DENSITY LIPOPROTEIN INCREASED	10024910	55
OTITIS MEDIA	10033078	55
PAPILLOEDEMA	10033712	55
PERICARDIAL DRAINAGE	10034471	55
PLASMAPHERESIS	10035486	55
RHINITIS	10039083	55
SUDDEN DEATH	10042434	55
NEW DAILY PERSISTENT HEADACHE	10076678	55
SPINAL STENOSIS	10082214	55
DENTAL DISCOMFORT	10054217	55
ANGIOPATHY	10059245	55
SPONDYLITIS	10061371	55
NASAL DISORDER	10062209	55
DARK CIRCLES UNDER EYES	10064729	55
BLOOD MAGNESIUM INCREASED	10005655	54
GIANT CELL ARTERITIS	10018250	54
HEPATITIS	10019717	54
MELAENA	10027141	54
PULMONARY INFARCTION	10037410	54
RESPIRATORY TRACT IRRITATION	10038731	54
SINUS ARRHYTHMIA	10040739	54
SKIN FISSURES	10040849	54
SPINAL X-RAY NORMAL	10041606	54
VENOGRAM ABNORMAL	10047209	54
YAWNING	10048232	54
POSTICTAL STATE	10048727	54
BONE DENSITOMETRY	10050973	54
ULTRASOUND PELVIS ABNORMAL	10052042	54
FACIAL ASYMMETRY	10068737	54
ANTI-CYCLIC CITRULLINATED PEPTIDE ANTIBODY NEGATIVE	10068799	54
HERPES VIRUS TEST	10077971	54
INJECTION SITE MUSCLE WEAKNESS	10084588	54
DIASTOLIC DYSFUNCTION	10052337	54

INTRAOCULAR PRESSURE TEST	10060950	54
TOOTH FRACTURE	10062544	54
AMYLASE	10002013	53
DERMATITIS ATOPIC	10012438	53
EXOPHTHALMOS	10015683	53
HYPERNATRAEMIA	10020679	53
LOW DENSITY LIPOPROTEIN NORMAL	10024911	53
MEAN CELL VOLUME	10026999	53
OXYGEN SATURATION ABNORMAL	10033317	53
POLYARTHRITIS	10036030	53
PREMATURE RUPTURE OF MEMBRANES	10036603	53
SKIN HYPERPIGMENTATION	10040865	53
SPINAL COMPRESSION FRACTURE	10041541	53
BASOPHIL COUNT	10049695	53
STAPHYLOCOCCUS TEST POSITIVE	10070052	53
THERAPEUTIC PRODUCT EFFECT DECREASED	10082201	53
JOINT WARMTH	10054106	53
ANTI-THYROID ANTIBODY POSITIVE	10060310	53
PIGMENTATION DISORDER	10062080	53
ALBUMIN GLOBULIN RATIO NORMAL	10001569	52
ARTHRITIS REACTIVE	10003267	52
AUTOIMMUNE HEPATITIS	10003827	52
CHILLBLAINS	10008528	52
COCCYDYNIA	10009829	52
FOOT FRACTURE	10016970	52
JOINT CONTRACTURE	10023201	52
LYMPHOPENIA	10025327	52
OESOPHAGEAL PAIN	10030180	52
ULTRASOUND KIDNEY NORMAL	10045423	52
CYTOMEGALOVIRUS TEST NEGATIVE	10051622	52
OCCIPITAL NEURALGIA	10068106	52
VULVOVAGINAL SWELLING	10071211	52
SKIN TEXTURE ABNORMAL	10075267	52
HERPES SIMPLEX TEST	10077968	52
PRODUCT ADMINISTRATION INTERRUPTED	10081479	52
DERMATITIS BULLOUS	10012441	51
EYELID OEDEMA	10015993	51
GENITAL RASH	10018175	51
MYOCARDIAL ISCHAEMIA	10028600	51
OESOPHAGEAL SPASM	10030184	51
ULTRASOUND SCAN VAGINA ABNORMAL	10045439	51
YELLOW SKIN	10048245	51
GASTROSTOMY	10048978	51

INJECTION SITE STREAKING	10066778	51
FOETAL HYPOKINESIA	10068461	51
FUNCTIONAL GASTROINTESTINAL DISORDER	10071275	51
HUMAN CHORIONIC GONADOTROPIN DECREASED	10071331	51
MENTAL FATIGUE	10076757	51
CORONAVIRUS TEST	10084353	51
BLOOD PHOSPHORUS NORMAL	10054887	51
EPSTEIN-BARR VIRUS TEST POSITIVE	10064545	51
ACIDOSIS	10000486	50
AORTIC VALVE INCOMPETENCE	10002915	50
APNOEA	10002974	50
BLOOD LACTIC ACID DECREASED	10005634	50
FACIAL BONES FRACTURE	10016042	50
LYMPHADENOPATHY MEDIASTINAL	10025205	50
PROTHROMBIN LEVEL	10037047	50
RED CELL DISTRIBUTION WIDTH	10051168	50
HEART VALVE INCOMPETENCE	10067660	50
STAPHYLOCOCCUS TEST NEGATIVE	10070413	50
GINGIVAL DISCOMFORT	10077854	50
OXYGEN THERAPY	10078798	50
MAGNETIC RESONANCE IMAGING THORACIC	10083142	50
HYPERTENSIVE URGENCY	10058181	50
BRAIN SCAN NORMAL	10061944	50
ULTRASOUND UTERUS ABNORMAL	10064876	50
ANEURYSM	10002329	49
AREFLEXIA	10003084	49
BLOOD URIC ACID NORMAL	10005862	49
MENOPAUSAL SYMPTOMS	10027304	49
NEURITIS	10029240	49
POST HERPETIC NEURALGIA	10036376	49
RETINAL DETACHMENT	10038848	49
PERIPHERAL VENOUS DISEASE	10075049	49
ALLODYNIA	10053552	49
LACTATION DISORDER	10061261	49
SPINAL DISORDER	10061368	49
UPPER LIMB FRACTURE	10061394	49
BLOOD CHOLESTEROL NORMAL	10005426	48
BLOOD PRESSURE SYSTOLIC	10005756	48
CEREBRAL ATROPHY	10008096	48
DIVERTICULUM	10013554	48
HICCUPS	10020039	48
MONOCYTE COUNT DECREASED	10027878	48
PEMPHIGOID	10034277	48

TRANSVERSE SINUS THROMBOSIS	10044457	48
VAGINAL INFECTION	10046914	48
VISUAL ACUITY TESTS	10047533	48
X-RAY OF PELVIS AND HIP NORMAL	10048161	48
NITRITE URINE PRESENT	10051469	48
PHYSICAL DECONDITIONING	10051588	48
CHEST SCAN	10076373	48
ANGIOCARDIOGRAM	10080743	48
ACUTE LEFT VENTRICULAR FAILURE	10063081	48
ABSCESS DRAINAGE	10000279	47
BLOOD CREATINE PHOSPHOKINASE MB	10005471	47
BLOOD CREATININE DECREASED	10005482	47
CEREBRAL ARTERY OCCLUSION	10008089	47
CYSTITIS INTERSTITIAL	10011796	47
DYSPNOEA AT REST	10013969	47
ENCEPHALITIS	10014581	47
LIPOMA	10024612	47
MUSCLE INJURY	10028314	47
RHEUMATOID FACTOR POSITIVE	10039080	47
RHONCHI	10039109	47
SICK RELATIVE	10040637	47
SKIN WRINKLING	10040954	47
EOSINOPHIL PERCENTAGE INCREASED	10052222	47
LYMPHOCYTE PERCENTAGE INCREASED	10052232	47
LEGIONELLA TEST	10070410	47
POLYMERASE CHAIN REACTION POSITIVE	10075628	47
IMMUNE-MEDIATED ADVERSE REACTION	10077665	47
THERAPEUTIC RESPONSE SHORTENED	10078575	47
BALANCE TEST	10084567	47
ULTRASOUND UTERUS	10052601	47
BODY HEIGHT DECREASED	10056812	47
HERPES ZOSTER OTICUS	10063491	47
ACTIVATED PARTIAL THROMBOPLASTIN TIME PROLONGED	10000636	46
BILIARY COLIC	10004663	46
BIOPSY BONE MARROW ABNORMAL	10004738	46
BIOPSY ENDOMETRIUM	10004769	46
BLOOD BICARBONATE INCREASED	10005360	46
CULTURE STOOL NEGATIVE	10011630	46
ENDOMETRIOSIS	10014778	46
LIP OEDEMA	10024558	46
PREGNANCY TEST URINE	10036576	46
PYELONEPHRITIS	10037596	46
ULTRASOUND LIVER	10045427	46

WHITE BLOOD CELLS URINE NEGATIVE	10047965	46
LIVER INJURY	10067125	46
SOFT TISSUE SWELLING	10076991	46
CSF TEST	10059690	46
ILL-DEFINED DISORDER	10061520	46
ARTERIOGRAM CORONARY ABNORMAL	10003201	45
ARTERIOGRAM CORONARY NORMAL	10003202	45
BRONCHOSCOPY	10006479	45
CLOSTRIDIUM DIFFICILE COLITIS	10009657	45
COELIAC DISEASE	10009839	45
CUTANEOUS VASCULITIS	10011686	45
FEELING OF DESPAIR	10016344	45
BLOOD URINE ABSENT	10018868	45
IRON BINDING CAPACITY TOTAL	10022960	45
LOW DENSITY LIPOPROTEIN	10024900	45
MACULE	10025421	45
MENOMETRORRHAGIA	10027295	45
PORTAL VEIN THROMBOSIS	10036206	45
TREATMENT NONCOMPLIANCE	10049414	45
LACUNAR INFARCTION	10051078	45
HEPATITIS C VIRUS TEST	10068416	45
ANTIPHOSPHOLIPID ANTIBODIES NEGATIVE	10058342	45
INNER EAR DISORDER	10061524	45
RED BLOOD CELL ABNORMALITY	10061548	45
BLOOD TRIGLYCERIDES	10005836	44
BLOOD TRIGLYCERIDES NORMAL	10005840	44
DIABETES MELLITUS INADEQUATE CONTROL	10012607	44
DISSEMINATED INTRAVASCULAR COAGULATION	10013442	44
EMBOLIC STROKE	10014498	44
GASTROENTERITIS	10017888	44
HAEMORRHAGE INTRACRANIAL	10018985	44
IRON DEFICIENCY	10022970	44
MENINGITIS	10027199	44
PUPIL FIXED	10037515	44
TOOTH ABSCESS	10044016	44
CEREBRAL MASS EFFECT	10067086	44
SKIN PLAQUE	10067723	44
VACCINATION SITE DISCHARGE	10069560	44
BLISTER RUPTURE	10073385	44
IRREGULAR BREATHING	10076213	44
CIRCUMORAL SWELLING	10081703	44
CATHETER PLACEMENT	10052915	44
AFFECT LABILITY	10054196	44

ENDOMETRIAL THICKENING	10056432	44
LUNG NEOPLASM MALIGNANT	10058467	44
FOOT DEFORMITY	10061159	44
ISCHAEMIA	10061255	44
SCAN ABNORMAL	10061499	44
HEPATITIS C TEST NEGATIVE	10065001	44
EXERCISE ELECTROCARDIOGRAM	10015644	43
FRACTURE	10017076	43
INTRACRANIAL PRESSURE INCREASED	10022773	43
SCOLIOSIS	10039722	43
SMEAR TEST	10041210	43
THROMBOPHLEBITIS	10043570	43
ULTRASOUND BREAST NORMAL	10045411	43
VENTRICULAR FIBRILLATION	10047290	43
CORONARY ARTERY DISSECTION	10048631	43
FOOD AVERSION	10049238	43
BLOOD PHOSPHORUS DECREASED	10049471	43
HEPARIN-INDUCED THROMBOCYTOPENIA TEST	10050829	43
VULVOVAGINAL BURNING SENSATION	10067641	43
END STAGE RENAL DISEASE	10077512	43
VESTIBULAR MIGRAINE	10077920	43
IDIOPATHIC INTRACRANIAL HYPERTENSION	10078904	43
MAGNETIC RESONANCE IMAGING BRAIN NORMAL	10083129	43
SUTURE INSERTION	10052665	43
BLOOD KETONE BODY	10057593	43
GENITAL PAIN	10061979	43
IMMUNOSUPPRESSION	10062016	43
PREMENSTRUAL PAIN	10065347	43
ARTHROPOD STING	10003402	42
BONE SCAN	10006009	42
CAPILLARY FRAGILITY	10007191	42
CULTURE THROAT	10011632	42
GLUCOSE TOLERANCE IMPAIRED	10018429	42
PHARYNGEAL OEDEMA	10034829	42
PROCTALGIA	10036772	42
SKIN PAPILLOMA	10040907	42
ULTRASOUND SCAN VAGINA NORMAL	10045440	42
VEIN DISCOLOURATION	10047183	42
ANGIOTENSIN CONVERTING ENZYME	10050289	42
SPUTUM CULTURE	10050419	42
RED BLOOD CELLS URINE	10050676	42
CORONAVIRUS TEST POSITIVE	10070255	42
MAGNETIC RESONANCE IMAGING ABDOMINAL	10083126	42

MAGNETIC RESONANCE IMAGING JOINT	10083134	42
EPIGASTRIC DISCOMFORT	10053155	42
MAJOR DEPRESSION	10057840	42
PULMONARY FUNCTION TEST ABNORMAL	10061602	42
VULVOVAGINAL MYCOTIC INFECTION	10064899	42
ANION GAP INCREASED	10002528	41
BLEPHARITIS	10005148	41
BLOOD BICARBONATE	10005357	41
CORNEAL REFLEX DECREASED	10011042	41
DRUG ERUPTION	10013687	41
EUSTACHIAN TUBE DYSFUNCTION	10015543	41
EXOSTOSIS	10015688	41
NORMOCYTIC ANAEMIA	10029784	41
PCO2 DECREASED	10034181	41
THROMBOTIC THROMBOCYTOPENIC PURPURA	10043648	41
FOREIGN BODY SENSATION IN EYES	10051116	41
EPSTEIN-BARR VIRUS TEST NEGATIVE	10067926	41
INFLUENZA VIRUS TEST POSITIVE	10070717	41
GASTROINTESTINAL WALL THICKENING	10075724	41
INCREASED VISCOSITY OF UPPER RESPIRATORY SECRETION	10076745	41
WRONG TECHNIQUE IN DEVICE USAGE PROCESS	10077040	41
SKIN INDENTATION	10079274	41
MAGNETIC RESONANCE IMAGING BRAIN ABNORMAL	10083130	41
INJECTION SITE SCAR	10059009	41
SIMILAR REACTION ON PREVIOUS EXPOSURE TO DRUG	10063672	41
MYOSCLEROSIS	10064584	41
BLOOD CREATINE INCREASED	10005464	40
DEMENTIA ALZHEIMER'S TYPE	10012271	40
DERMATOMYOSITIS	10012503	40
EPISCLERITIS	10015084	40
FOLLICULITIS	10016936	40
HEPATIC CIRRHOSIS	10019641	40
JUGULAR VEIN THROMBOSIS	10023237	40
MIOSIS	10027646	40
PANIC DISORDER	10033666	40
PREMATURE SEPARATION OF PLACENTA	10036608	40
LYMPHADENECTOMY	10048956	40
LIP HAEMORRHAGE	10049297	40
LEFT ATRIAL ENLARGEMENT	10051860	40
TROPONIN T NORMAL	10073407	40
EPSTEIN-BARR VIRUS ANTIBODY POSITIVE	10052324	40
RIGHT VENTRICULAR DYSFUNCTION	10058597	40
LOWER LIMB FRACTURE	10061599	40

TOOTH EXTRACTION	10062132	40
NON-CARDIAC CHEST PAIN	10062501	40
LIMB IMMOBILISATION	10062615	40
CHRONIC LEFT VENTRICULAR FAILURE	10063083	40
BIOPSY LIVER	10004791	39
BIOPSY LIVER ABNORMAL	10004792	39
BLOOD LACTATE DEHYDROGENASE NORMAL	10005631	39
CYSTOSCOPY	10011814	39
ELECTROCARDIOGRAM ST SEGMENT ABNORMAL	10014390	39
GESTATIONAL DIABETES	10018209	39
GLOBULINS INCREASED	10018350	39
GRAM STAIN POSITIVE	10018656	39
HENOCH-SCHONLEIN PURPURA	10019617	39
INAPPROPRIATE AFFECT	10021588	39
NASAL INFLAMMATION	10028741	39
STRABISMUS	10042159	39
SUFFOCATION FEELING	10042444	39
TOXIC ENCEPHALOPATHY	10044221	39
TRACHEOSTOMY	10044320	39
VISUAL FIELD TESTS	10047566	39
GINGIVAL BLISTER	10049304	39
VENTRICULAR HYPOKINESIA	10050510	39
GINGIVAL ERYTHEMA	10067419	39
OROPHARYNGEAL BLISTERING	10067950	39
PERIPHERAL ARTERY THROMBOSIS	10072564	39
DEPERSONALISATION/DEREALISATION DISORDER	10077805	39
DISCOLOURED VOMIT	10079120	39
ATTENTION DEFICIT HYPERACTIVITY DISORDER	10083622	39
X-RAY DENTAL	10052955	39
INJECTION	10052995	39
DERMATOLOGIC EXAMINATION	10058379	39
ANTIBODY TEST ABNORMAL	10061425	39
RESPIRATORY SYNCYTIAL VIRUS INFECTION	10061603	39
OCCULT BLOOD POSITIVE	10061880	39
HEPATITIS VIRAL TEST	10061999	39
COMPLEX REGIONAL PAIN SYNDROME	10064332	39
FEAR OF DEATH	10066392	39
AFFECTIVE DISORDER	10001443	38
AORTIC STENOSIS	10002906	38
ATRIAL SEPTAL DEFECT	10003664	38
DYSCHROMATOPSIA	10013892	38
EAR HAEMORRHAGE	10014009	38
EXTUBATION	10015894	38

IRON BINDING CAPACITY TOTAL NORMAL	10022967	38
PERSONALITY CHANGE	10034719	38
POLYDIPSIA	10036067	38
PRE-EXISTING CONDITION IMPROVED	10036495	38
RESPIRATORY DEPRESSION	10038678	38
HYSTEROSCOPY	10050125	38
SPUTUM CULTURE POSITIVE	10051612	38
VACCINATION SITE INFECTION	10068877	38
HELICOBACTER TEST NEGATIVE	10070409	38
PRODUCT RECONSTITUTION QUALITY ISSUE	10079007	38
RETINAL MIGRAINE	10052784	38
URINE PROTEIN/CREATININE RATIO	10053537	38
INTERVERTEBRAL DISC DISORDER	10061521	38
GENE MUTATION IDENTIFICATION TEST	10063477	38
NASOPHARYNGEAL SWAB	10085633	38
CAROTID ARTERY STENOSIS	10007687	37
CSF GLUCOSE NORMAL	10011538	37
HEPATIC FAILURE	10019663	37
INTRAOCULAR PRESSURE INCREASED	10022806	37
LOSS OF CONTROL OF LEGS	10024860	37
OSTEOMYELITIS	10031252	37
PERIPHERAL VASCULAR DISORDER	10034636	37
SLEEP TERROR	10041010	37
TONGUE MOVEMENT DISTURBANCE	10043963	37
VISUAL ACUITY REDUCED	10047531	37
WHITE BLOOD CELL COUNT ABNORMAL	10047940	37
C-REACTIVE PROTEIN DECREASED	10049220	37
EXTREMITY CONTRACTURE	10050732	37
DYSCHEZIA	10051244	37
INFLUENZA A VIRUS TEST POSITIVE	10070215	37
MYCOBACTERIUM TUBERCULOSIS COMPLEX TEST	10070472	37
HUMAN CHORIONIC GONADOTROPIN POSITIVE	10071335	37
AUTOIMMUNE HAEMOLYTIC ANAEMIA	10073785	37
COOLING THERAPY	10079482	37
LUNG HYPERINFLATION	10059487	37
ANTINEUTROPHIL CYTOPLASMIC ANTIBODY NEGATIVE	10060137	37
RENAL FUNCTION TEST ABNORMAL	10061480	37
ALLERGY TO CHEMICALS	10061626	37
BLADDER DISCOMFORT	10005034	36
BLOOD FIBRINOGEN INCREASED	10005521	36
CRANIOTOMY	10011322	36
DYSAESTHESIA	10013886	36
FOOD POISONING	10016952	36

HAIR TEXTURE ABNORMAL	10019049	36
LICHEN SCLEROSUS	10024434	36
NEPHROTIC SYNDROME	10029164	36
RETINAL HAEMORRHAGE	10038867	36
SKIN TEST POSITIVE	10040934	36
TONGUE COATED	10043945	36
OSTEOPENIA	10049088	36
PROTEIN C	10050416	36
TONIC CLONIC MOVEMENTS	10051171	36
EYE OEDEMA	10052139	36
BUTTERFLY RASH	10067982	36
BURNING MOUTH SYNDROME	10068065	36
MICROSCOPY	10069374	36
CLOSTRIDIUM TEST POSITIVE	10070027	36
GESTATIONAL HYPERTENSION	10070538	36
BREAST CONSERVING SURGERY	10076783	36
CEREBELLAR STROKE	10079062	36
ALTERED VISUAL DEPTH PERCEPTION	10053549	36
EYE INJURY	10061128	36
FACIAL NERVE DISORDER	10061457	36
PARKINSON'S DISEASE	10061536	36
ATRIAL TACHYCARDIA	10003668	35
AUDIOGRAM NORMAL	10003762	35
BIOPSY ENDOMETRIUM NORMAL	10004771	35
CHIROPRACTIC	10008534	35
CONVERSION DISORDER	10010893	35
DECUBITUS ULCER	10011985	35
FEELING OF RELAXATION	10016352	35
HEMIPLEGIC MIGRAINE	10019476	35
INTERNATIONAL NORMALISED RATIO DECREASED	10022594	35
MENINGITIS ASEPTIC	10027201	35
PERIORBITAL PAIN	10034546	35
PETIT MAL EPILEPSY	10034759	35
ULTRASOUND ANTENATAL SCREEN NORMAL	10045401	35
VIRAL RASH	10047476	35
VITAMIN B12 DEFICIENCY	10047609	35
VITH NERVE PARALYSIS	10047641	35
VENA CAVA FILTER INSERTION	10048932	35
VACCINATION SITE OEDEMA	10069617	35
MENISCUS INJURY	10072970	35
WRONG PATIENT	10084399	35
QUARANTINE	10084468	35
PROGESTERONE	10063291	35

PROCEDURAL PAIN	10064882	35
EXCESSIVE EYE BLINKING	10065166	35
INJECTION SITE DISCHARGE	10065600	35
EXTERNAL EAR PAIN	10065785	35
ANAPHYLACTOID REACTION	10002216	34
CEREBRAL ISCHAEMIA	10008120	34
CEREBRAL VENOUS THROMBOSIS	10008138	34
EPICONDYLITIS	10014971	34
IRIDOCYCLITIS	10022941	34
MULTIPLE ALLERGIES	10028164	34
NERVE BLOCK	10029173	34
ORAL LICHEN PLANUS	10030983	34
OVARIAN CYST RUPTURED	10033136	34
SALIVARY GLAND ENLARGEMENT	10039408	34
SENSATION OF BLOOD FLOW	10039996	34
TOOTH INJURY	10044043	34
IMPLANTABLE DEFIBRILLATOR INSERTION	10049442	34
ABSCESS LIMB	10050473	34
MEAN PLATELET VOLUME	10050539	34
HEARING AID USER	10050852	34
BRONCHIAL SECRETION RETENTION	10066820	34
HEPATITIS B VIRUS TEST	10068415	34
HELICOBACTER TEST	10070400	34
DRAIN PLACEMENT	10072795	34
DIAGNOSTIC ASPIRATION	10072924	34
IMPLANTABLE CARDIAC MONITOR INSERTION	10074731	34
CARDIOVASCULAR EXAMINATION	10074890	34
SWALLOW STUDY	10076217	34
TOTAL COMPLEMENT ACTIVITY TEST	10077882	34
CARDIAC DYSFUNCTION	10079751	34
INJECTION SITE PUSTULE	10054994	34
MEAN PLATELET VOLUME DECREASED	10055053	34
BLOOD 25-HYDROXYCHOLECALCIFEROL	10059936	34
CONNECTIVE TISSUE DISORDER	10061087	34
COMPLICATION OF PREGNANCY	10061452	34
ARTERIAL OCCLUSIVE DISEASE	10062599	34
FLOW CYTOMETRY	10065440	34
MAGNETIC RESONANCE CHOLANGIOPANCREATOGRAPHY	10066085	34
BACTERIAL VAGINOSIS	10004055	33
BLOOD PH INCREASED	10005708	33
CARDIAC TAMPONADE	10007610	33
DIET REFUSAL	10012775	33
ELECTROCARDIOGRAM QT INTERVAL	10014385	33

ENURESIS	10014928	33
GINGIVITIS	10018292	33
GLAUCOMA	10018304	33
HIGH DENSITY LIPOPROTEIN DECREASED	10020060	33
OESTRADIOL	10030227	33
PROTHROMBIN LEVEL NORMAL	10037052	33
SARCOIDOSIS	10039486	33
TENDON RUPTURE	10043248	33
TONGUE OEDEMA	10043967	33
TOTAL LUNG CAPACITY DECREASED	10044100	33
ULTRASOUND KIDNEY ABNORMAL	10045422	33
X-RAY OF PELVIS AND HIP ABNORMAL	10048160	33
LAZINESS	10051602	33
MYOCARDIAL STRAIN	10066954	33
MATERNAL EXPOSURE TIMING UNSPECIFIED	10071415	33
MAGNETIC RESONANCE IMAGING ABDOMINAL ABNORMAL	10083132	33
SCAN MYOCARDIAL PERFUSION	10054082	33
MENSTRUAL DISCOMFORT	10056344	33
CHRONIC INFLAMMATORY DEMYELINATING POLYRADICULONE	10057645	33
BIOPSY UTERUS NORMAL	10058404	33
CSF TEST ABNORMAL	10059703	33
FREEZING PHENOMENON	10060904	33
CRANIAL NERVE DISORDER	10061093	33
OESOPHAGEAL DISORDER	10061318	33
OPTIC NERVE DISORDER	10061322	33
CARDIAC VALVE DISEASE	10061406	33
POLYP	10061529	33
DIAGNOSTIC PROCEDURE	10061816	33
ORTHOSIS USER	10066194	33
AORTIC ANEURYSM	10002882	32
ATRIOVENTRICULAR BLOCK COMPLETE	10003673	32
ATRIOVENTRICULAR BLOCK FIRST DEGREE	10003674	32
BIOPSY KIDNEY ABNORMAL	10004783	32
BRONCHIECTASIS	10006445	32
CSF CULTURE NEGATIVE	10011528	32
GASTRIC EMPTYING STUDY	10017782	32
HEPATIC CYST	10019646	32
HEPATIC FUNCTION ABNORMAL	10019670	32
HEPATITIS C ANTIBODY	10019745	32
HILAR LYMPHADENOPATHY	10020094	32
RADICULOPATHY	10037779	32
SKIN HYPERTROPHY	10040867	32
SPLENIC INFARCTION	10041648	32

TRI-IODOTHYRONINE INCREASED	10044596	32
TUBERCULIN TEST	10044726	32
UPPER GASTROINTESTINAL HAEMORRHAGE	10046274	32
LARGE INTESTINE POLYP	10051589	32
C-REACTIVE PROTEIN ABNORMAL	10068559	32
VACCINATION SITE SCAR	10069619	32
VARICELLA VIRUS TEST NEGATIVE	10070445	32
MYCOBACTERIUM TUBERCULOSIS COMPLEX TEST NEGATIVE	10070471	32
CRANIOCEREBRAL INJURY	10070976	32
FOETAL EXPOSURE DURING PREGNANCY	10071404	32
AUSCULTATION	10076270	32
IMMATURE GRANULOCYTE COUNT INCREASED	10081727	32
REFLEX TEST	10082738	32
PARANASAL SINUS INFLAMMATION	10083836	32
TRI-IODOTHYRONINE FREE	10053792	32
THYROXINE FREE INCREASED	10055163	32
POST VIRAL FATIGUE SYNDROME	10057244	32
TROPONIN T INCREASED	10058269	32
SKIN TEST NEGATIVE	10060888	32
BRAIN OPERATION	10061732	32
ORTHOSTATIC INTOLERANCE	10063927	32
TONSILLAR INFLAMMATION	10065169	32
ABORTION MISSED	10000230	31
ACNE CYSTIC	10000503	31
BIOPSY SKIN NORMAL	10004875	31
BLOOD PRESSURE DIASTOLIC INCREASED	10005739	31
BRONCHOSCOPY ABNORMAL	10006480	31
CHOLECYSTITIS ACUTE	10008614	31
CHRONIC RESPIRATORY FAILURE	10009126	31
CREATININE RENAL CLEARANCE DECREASED	10011372	31
EPINEPHRINE	10015060	31
ESSENTIAL TREMOR	10015496	31
GRANULOMA	10018691	31
HAEMOLYSIS	10018910	31
HERNIA	10019909	31
HYPERCOAGULATION	10020608	31
PAROTID GLAND ENLARGEMENT	10034023	31
POST-TRAUMATIC STRESS DISORDER	10036316	31
PURULENT DISCHARGE	10037569	31
RETINAL TEAR	10038897	31
RHINITIS ALLERGIC	10039085	31
SPIROMETRY	10041629	31
TRI-IODOTHYRONINE NORMAL	10044598	31

URINARY TRACT DISORDER	10046566	31
VITAMIN B12 INCREASED	10047610	31
WOUND SECRETION	10048629	31
LIVE BIRTH	10049550	31
THIRST DECREASED	10050200	31
OVULATION DISORDER	10067490	31
CLOSTRIDIUM TEST NEGATIVE	10070271	31
TILT TABLE TEST POSITIVE	10072467	31
NONINFECTIVE ENCEPHALITIS	10074712	31
CSF RED BLOOD CELL COUNT POSITIVE	10075562	31
HERPES SIMPLEX TEST POSITIVE	10077969	31
NASAL INJURY	10078651	31
MAGNETIC RESONANCE IMAGING THORACIC ABNORMAL	10083143	31
INCISIONAL DRAINAGE	10052532	31
WALKING DISABILITY	10053204	31
HISTAMINE LEVEL	10060029	31
VOCAL CORD DISORDER	10061581	31
HEPATIC LESION	10061998	31
OVERSENSING	10063834	31
ABORTION INDUCED	10000220	30
ACUTE SINUSITIS	10001076	30
BEHAVIOUR DISORDER	10004207	30
BLOOD PROLACTIN	10005777	30
CULTURE THROAT NEGATIVE	10011633	30
DIVERTICULUM INTESTINAL	10013559	30
ENTERITIS	10014866	30
HAEMOLYTIC ANAEMIA	10018916	30
LEUKAEMIA	10024288	30
NARCOLEPSY	10028713	30
ON AND OFF PHENOMENON	10030312	30
PLASMA CELL MYELOMA	10035226	30
PLATELET DISORDER	10035532	30
SOMNAMBULISM	10041347	30
WOUND INFECTION	10048038	30
MOUTH INJURY	10049294	30
STAPHYLOCOCCAL BACTERAEMIA	10051017	30
MADAROSIS	10051235	30
POSTMENOPAUSE	10051775	30
VERTEBRAL FORAMINAL STENOSIS	10069690	30
INFLUENZA B VIRUS TEST POSITIVE	10070208	30
FUNGAL TEST NEGATIVE	10070458	30
RIGHT VENTRICULAR DILATATION	10074222	30
LANGUAGE DISORDER	10074869	30

RHEUMATOLOGICAL EXAMINATION	10084263	30
HAEMOFILTRATION	10053090	30
CARDIAC IMAGING PROCEDURE	10053451	30
CARDIAC IMAGING PROCEDURE NORMAL	10053452	30
TONSILLAR DISORDER	10053477	30
CSF WHITE BLOOD CELL COUNT INCREASED	10053805	30
NUCHAL RIGIDITY	10058483	30
HAND DERMATITIS	10058898	30
BLOOD SMEAR TEST	10059655	30
URINE OUTPUT INCREASED	10059896	30
TOXICOLOGIC TEST	10061384	30
HERPES OPHTHALMIC	10062004	30
AGONAL RESPIRATION	10085467	30
ACUTE MYELOID LEUKAEMIA	10000880	29
ADRENAL DISORDER	10001347	29
AMYOTROPHIC LATERAL SCLEROSIS	10002026	29
ANTIPHOSPHOLIPID SYNDROME	10002817	29
ATRIOVENTRICULAR BLOCK SECOND DEGREE	10003677	29
AUDITORY DISORDER	10003778	29
BREAST INDURATION	10006258	29
CHANGE OF BOWEL HABIT	10008399	29
CORONARY ARTERY BYPASS	10011077	29
HYPOCALCAEMIA	10020947	29
LARYNGOSCOPY	10023888	29
MEDICAL DIET	10027087	29
MELANOCYTIC NAEVUS	10027145	29
PREMATURE BABY	10036590	29
TRICHORRHEXIS	10044625	29
VULVAL DISORDER	10047754	29
BLADDER SPASM	10048994	29
CORRECTIVE LENS USER	10050858	29
CORONAVIRUS INFECTION	10051905	29
REGURGITATION	10067171	29
BRONCHIAL WALL THICKENING	10067182	29
PRODUCT LABEL CONFUSION	10069273	29
FUNGAL TEST	10070457	29
SENSITISATION	10070834	29
THYROID STIMULATING IMMUNOGLOBULIN	10071388	29
VACCINATION SITE JOINT MOVEMENT IMPAIRMENT	10076175	29
X-RAY DENTAL NORMAL	10079994	29
RECALLED PRODUCT ADMINISTERED	10081540	29
MUSCLE STRENGTH ABNORMAL	10082799	29
INJECTION SITE JOINT MOVEMENT IMPAIRMENT	10053979	29

BLOODY DISCHARGE	10057687	29
VITAMIN B1	10058766	29
COMPUTERISED TOMOGRAM CORONARY ARTERY	10060804	29
TRIGEMINAL NERVE DISORDER	10060890	29
FOETAL DISORDER	10061157	29
SKIN INJURY	10061364	29
EYE OPERATION	10061852	29
FLUID REPLACEMENT	10061858	29
NECK INJURY	10062211	29
SPIDER VEIN	10062696	29
ANTI-THYROID ANTIBODY INCREASED	10085912	29
ANGIOPLASTY	10002475	28
BLOOD URIC ACID INCREASED	10005861	28
ECTOPIC PREGNANCY	10014166	28
ELECTRONYSTAGMOGRAM	10014449	28
GASTRIC ULCER	10017822	28
NAIL DISORDER	10028694	28
NERVE STIMULATION TEST	10029191	28
OPTIC ISCHAEMIC NEUROPATHY	10030924	28
SCLERITIS	10039705	28
THYROID PAIN	10043757	28
UTERINE POLYP	10046811	28
TRANSFERRIN SATURATION DECREASED	10050770	28
BETA-2 GLYCOPROTEIN ANTIBODY	10066403	28
DEBRIDEMENT	10067806	28
ULTRASOUND UTERUS NORMAL	10074417	28
PRODUCT AVAILABILITY ISSUE	10077800	28
MUCOSAL HYPERTROPHY	10079674	28
CATHETER DIRECTED THROMBOLYSIS	10085325	28
OPTICAL COHERENCE TOMOGRAPHY	10057208	28
QRS AXIS ABNORMAL	10057624	28
HYPERTENSIVE EMERGENCY	10058179	28
BRAIN NEOPLASM	10061019	28
EMBOLISM	10061169	28
GASTROINTESTINAL MOTILITY DISORDER	10061173	28
MASS EXCISION	10061560	28
STEROID THERAPY	10062117	28
IMMUNOLOGY TEST NORMAL	10062502	28
THALAMIC INFARCTION	10064961	28
HEPATITIS B TEST NEGATIVE	10065002	28
APRAXIA	10003062	27
BILIRUBIN CONJUGATED	10004684	27
BLOOD IMMUNOGLOBULIN G DECREASED	10005595	27

BLOOD IMMUNOGLOBULIN G INCREASED	10005596	27
DECREASED INTEREST	10011971	27
FUNGAL SKIN INFECTION	10017543	27
HIGH DENSITY LIPOPROTEIN NORMAL	10020062	27
MITRAL VALVE PROLAPSE	10027730	27
OVULATION PAIN	10033314	27
PANCREATIC DISORDER	10033616	27
PELVIC VENOUS THROMBOSIS	10034272	27
PHYSIOTHERAPY	10034998	27
POSTPARTUM HAEMORRHAGE	10036417	27
PYURIA	10037686	27
RADICULITIS BRACHIAL	10037778	27
SLEEP PARALYSIS	10041002	27
SNORING	10041235	27
STATUS EPILEPTICUS	10041962	27
SUBCUTANEOUS ABSCESS	10042343	27
THROMBOCYTOSIS	10043563	27
THYROXINE INCREASED	10043818	27
TYMPANOMETRY	10045214	27
ONYCHOCLASIS	10048886	27
BRAIN DEATH	10049054	27
SUBCLAVIAN VEIN THROMBOSIS	10049446	27
EXPOSURE TO COMMUNICABLE DISEASE	10049711	27
ALDOLASE	10050286	27
ANTITHROMBIN III	10050640	27
ENDODONTIC PROCEDURE	10051085	27
ENDOMETRIAL ABLATION	10051908	27
BETA-2 GLYCOPROTEIN ANTIBODY NEGATIVE	10066405	27
VARICELLA VIRUS TEST POSITIVE	10070214	27
DIFFUSE ALOPECIA	10073736	27
COMPLICATION ASSOCIATED WITH DEVICE	10077107	27
INTRACRANIAL MASS	10077667	27
PROHORMONE BRAIN NATRIURETIC PEPTIDE	10077780	27
FOOD REFUSAL	10080283	27
CONTINUOUS POSITIVE AIRWAY PRESSURE	10052934	27
CARDIAC ASSISTANCE DEVICE USER	10053686	27
MINERAL SUPPLEMENTATION	10053963	27
POST PROCEDURAL COMPLICATION	10058046	27
PCO2 NORMAL	10058983	27
CSF TEST NORMAL	10059702	27
LIMB OPERATION	10061226	27
TOXICOLOGIC TEST NORMAL	10061383	27
EUSTACHIAN TUBE DISORDER	10061462	27

COLECTOMY	10061778	27
BLOOD HOMOCYSTEINE	10062994	27
INJECTION SITE INJURY	10066083	27
ACCIDENT	10000369	26
BIOPSY LUNG	10004794	26
BIOPSY THYROID GLAND	10004888	26
BLOOD OESTROGEN	10005684	26
BLOOD TESTOSTERONE	10005811	26
CHRONIC LYMPHOCYTIC LEUKAEMIA	10008958	26
COAGULATION TIME	10009790	26
ENDOSCOPY UPPER GASTROINTESTINAL TRACT	10014819	26
EOSINOPHILIA	10014950	26
EXTRAOCULAR MUSCLE PARESIS	10015829	26
FEBRILE NEUTROPENIA	10016288	26
HAIR DISORDER	10019037	26
HEPATITIS C ANTIBODY NEGATIVE	10019746	26
PEMPHIGUS	10034280	26
SMEAR CERVIX ABNORMAL	10041206	26
KNEE OPERATION	10049548	26
INJECTION SITE DRYNESS	10067252	26
RESPIRATORY SYNCYTIAL VIRUS TEST POSITIVE	10068563	26
VACCINATION SITE HYPERSENSITIVITY	10068880	26
PRODUCT QUALITY ISSUE	10069327	26
VACCINATION SITE SCAB	10069618	26
FAECAL CALPROTECTIN	10069670	26
MENSTRUATION NORMAL	10070933	26
HUMAN CHORIONIC GONADOTROPIN NORMAL	10071334	26
ENZYME LEVEL TEST	10072233	26
EYE COLOUR CHANGE	10072289	26
ORAL BLOOD BLISTER	10076590	26
EXCESSIVE CERUMEN PRODUCTION	10076897	26
ADULT FAILURE TO THRIVE	10077257	26
ATRIAL ENLARGEMENT	10079340	26
THERAPEUTIC PRODUCT EFFECT INCOMPLETE	10082200	26
CONVALESCENT PLASMA TRANSFUSION	10084817	26
DIAPHRAGMALGIA	10052749	26
CARDIAC TELEMETRY ABNORMAL	10053450	26
EAR, NOSE AND THROAT EXAMINATION NORMAL	10056847	26
ENDOCRINE OPHTHALMOPATHY	10060742	26
VENOUS THROMBOSIS LIMB	10061408	26
WHITE BLOOD CELL DISORDER	10061414	26
ORAL MUCOSAL EXFOLIATION	10064487	26
PERCUTANEOUS CORONARY INTERVENTION	10065608	26

VACCINATION SITE PAPULE	10066046	26
BRONCHIAL HYPERREACTIVITY	10066091	26
ACUTE DISSEMINATED ENCEPHALOMYELITIS	10000709	25
ACUTE HEPATIC FAILURE	10000804	25
ADRENAL INSUFFICIENCY	10001367	25
ALBUMIN GLOBULIN RATIO DECREASED	10001565	25
ASPARTATE AMINOTRANSFERASE DECREASED	10003479	25
ATROPHY	10003694	25
B-CELL LYMPHOMA	10003899	25
BLOOD FOLLICLE STIMULATING HORMONE NORMAL	10005535	25
BLOOD LUTEINISING HORMONE	10005646	25
BREAST DISORDER FEMALE	10006229	25
CARDIOLIPIN ANTIBODY NEGATIVE	10007629	25
CAROTID ARTERY THROMBOSIS	10007688	25
CATATONIA	10007776	25
CHILD MALTREATMENT SYNDROME	10008513	25
CSF PROTEIN NORMAL	10011576	25
DECREASED IMMUNE RESPONSIVENESS	10011968	25
GINGIVAL DISORDER	10018280	25
GRAM STAIN	10018654	25
HEART SOUNDS ABNORMAL	10019311	25
LAGOPHTHALMOS	10023683	25
MYELITIS	10028524	25
PHARYNGEAL ULCERATION	10034834	25
POLYCYSTIC OVARIES	10036049	25
RENAL INFARCT	10038470	25
SCLERODERMA	10039710	25
SERUM SICKNESS	10040400	25
SPUTUM INCREASED	10041812	25
SUPERIOR SAGITTAL SINUS THROMBOSIS	10042567	25
THROMBOLYSIS	10043568	25
TOOTH LOSS	10044044	25
VENOUS THROMBOSIS	10047249	25
WRIST FRACTURE	10048049	25
PHYSICAL DISABILITY	10048624	25
PROTEIN S	10050417	25
BLOOD UREA NITROGEN/CREATININE RATIO INCREASED	10050760	25
WOUND HAEMORRHAGE	10051373	25
INNER EAR INFLAMMATION	10066966	25
ADVERSE EVENT FOLLOWING IMMUNISATION	10069520	25
VIRAL TITRE	10070252	25
AMYLASE NORMAL	10072328	25
VEIN RUPTURE	10077110	25

MUCOSAL DISORDER	10082268	25
ELECTROCARDIOGRAM ST-T SEGMENT ABNORMAL	10052333	25
PATIENT ISOLATION	10053315	25
EXPLORATIVE LAPAROTOMY	10053361	25
ANTIPLATELET THERAPY	10053460	25
EPSTEIN-BARR VIRUS ANTIBODY NEGATIVE	10053818	25
EAR, NOSE AND THROAT EXAMINATION ABNORMAL	10056848	25
HEPATIC MASS	10057110	25
ANXIETY DISORDER	10057666	25
BIPOLAR DISORDER	10057667	25
PSYCHOGENIC SEIZURE	10058895	25
SPINAL CORD DISORDER	10061367	25
WOUND TREATMENT	10062932	25
EFFUSION	10063045	25
EAR INFLAMMATION	10085924	25
ADRENOCORTICAL INSUFFICIENCY ACUTE	10001389	24
ASPIRATION JOINT	10003517	24
BITE	10004966	24
BLOOD IMMUNOGLOBULIN E INCREASED	10005591	24
BLOOD PROLACTIN NORMAL	10005781	24
CLAUSTROPHOBIA	10009244	24
DERMAL CYST	10012426	24
DIZZINESS EXERTIONAL	10013576	24
EMBOLISM VENOUS	10014522	24
FEBRILE CONVULSION	10016284	24
FLUID OVERLOAD	10016803	24
GUTTATE PSORIASIS	10018797	24
HIGH DENSITY LIPOPROTEIN	10020050	24
HYPOALBUMINAEMIA	10020942	24
INFERTILITY FEMALE	10021928	24
INTESTINAL ISCHAEMIA	10022680	24
MASTECTOMY	10026878	24
MUCOSAL DRYNESS	10028111	24
NOCTURIA	10029446	24
OTITIS EXTERNA	10033072	24
PERIPHERAL SENSORY NEUROPATHY	10034620	24
PRURITUS GENITAL	10037093	24
STEVENS-JOHNSON SYNDROME	10042033	24
TORTICOLLIS	10044074	24
TYMPANIC MEMBRANE PERFORATION	10045210	24
VESTIBULAR FUNCTION TEST	10047389	24
CAROTID ARTERY OCCLUSION	10048964	24
CERVICAL RADICULOPATHY	10050217	24

LEFT ATRIAL DILATATION	10067286	24
ANTI-CYCLIC CITRULLINATED PEPTIDE ANTIBODY POSITIVE	10068798	24
HUMAN RHINOVIRUS TEST POSITIVE	10070249	24
RESPIRATORY TRACT OEDEMA	10070774	24
EGFR STATUS ASSAY	10071955	24
DERMAL FILLER INJECTION	10072349	24
INVASIVE DUCTAL BREAST CARCINOMA	10073095	24
THERAPY CHANGE	10074300	24
ADVERSE FOOD REACTION	10077759	24
DENTAL PARAESTHESIA	10078276	24
LYMPHATIC DISORDER	10052314	24
ADRENAL MASS	10053235	24
OFFICE VISIT	10053323	24
FAECALOMA	10056325	24
CONGESTIVE CARDIOMYOPATHY	10056370	24
FOOD CRAVING	10056465	24
PERIPHERAL ARTERY OCCLUSION	10057525	24
CARDIAC OPERATION	10061026	24
FINGER DEFORMITY	10061156	24
HAND DEFORMITY	10061194	24
INFARCTION	10061216	24
LICHENOID KERATOSIS	10064000	24
THROMBOEMBOLECTOMY	10064958	24
AMNIOCENTESIS	10001958	23
ANOVULATORY CYCLE	10002659	23
BLOOD CREATINE NORMAL	10005466	23
BLOOD FOLLICLE STIMULATING HORMONE INCREASED	10005534	23
BLOOD GROUPING	10005569	23
CEREBELLAR INFARCTION	10008034	23
CORONARY ARTERY THROMBOSIS	10011091	23
CSF CULTURE	10011527	23
CSF PROTEIN	10011572	23
EPSTEIN-BARR VIRUS INFECTION REACTIVATION	10015109	23
EYELID SENSORY DISORDER	10015999	23
HYPERCALCAEMIA	10020583	23
HYPERTONIC BLADDER	10020853	23
IMMUNOGLOBULINS	10021496	23
INFLAMMATORY BOWEL DISEASE	10021972	23
JOINT INJECTION	10023218	23
MACULAR DEGENERATION	10025409	23
MESENTERIC VEIN THROMBOSIS	10027402	23
MUCOSAL INFLAMMATION	10028116	23
NONSPECIFIC REACTION	10029719	23

PAROTITIS	10034038	23
RENAL TUBULAR NECROSIS	10038540	23
SUBMAXILLARY GLAND ENLARGEMENT	10042419	23
ULTRASOUND LIVER ABNORMAL	10045428	23
URINARY SYSTEM X-RAY	10046558	23
VIRAL UPPER RESPIRATORY TRACT INFECTION	10047482	23
DRUG SCREEN POSITIVE	10049177	23
HAPTOGLOBIN	10050744	23
TRANSFERRIN SATURATION	10051007	23
BASOPHIL PERCENTAGE INCREASED	10052220	23
PHARYNGEAL DISORDER	10067353	23
POTENTIATING DRUG INTERACTION	10068072	23
HYPERTRANSAMINASAEMIA	10068237	23
ANORECTAL DISCOMFORT	10068286	23
BODY TEMPERATURE NORMAL	10069600	23
STRESS ECHOCARDIOGRAM NORMAL	10070745	23
CARDIOVASCULAR SYMPTOM	10075534	23
HLA-B*27 ASSAY	10078439	23
REDUCED FACIAL EXPRESSION	10078576	23
HEMIPARAESTHESIA	10078746	23
ANEMBRYONIC GESTATION	10079814	23
TROPONIN ABNORMAL	10084979	23
FIRST TRIMESTER PREGNANCY	10052395	23
EPSTEIN-BARR VIRUS ANTIBODY	10053817	23
HELICOBACTER INFECTION	10054263	23
TOBACCO USER	10057581	23
ANTIACETYLCHOLINE RECEPTOR ANTIBODY	10059956	23
AUTOANTIBODY TEST	10060972	23
SCAN THYROID GLAND	10061508	23
OCCULT BLOOD	10061878	23
PHYSICAL EXAMINATION NORMAL	10062079	23
MULTIMORBIDITY	10063914	23
AMYLASE INCREASED	10002016	22
AZOTAEMIA	10003885	22
BIOPSY LYMPH GLAND ABNORMAL	10004798	22
BLOOD IMMUNOGLOBULIN M INCREASED	10005601	22
BRAIN HERNIATION	10006126	22
BREATH ODOUR	10006326	22
ENDOCARDITIS	10014665	22
GASTRIC DILATATION	10017779	22
GLOMERULAR FILTRATION RATE INCREASED	10018359	22
HYPOREFLEXIA	10021089	22
ILEUS	10021328	22

ILLUSION	10021403	22
ISCHAEMIC HEPATITIS	10023025	22
LARYNGEAL OEDEMA	10023845	22
MONONUCLEOSIS HETEROPHILE TEST POSITIVE	10027922	22
MORNING SICKNESS	10027975	22
NEUTROPHILIA	10029379	22
OSTEITIS	10031149	22
PANCREATIC CYST	10033615	22
PUPILLARY REFLEX IMPAIRED	10037532	22
RETINAL DISORDER	10038853	22
SIALOADENITIS	10040628	22
ULTRASOUND LIVER NORMAL	10045429	22
VULVOVAGINAL DRYNESS	10047791	22
CERUMEN REMOVAL	10050336	22
CYTOKINE STORM	10050685	22
HISTAMINE INTOLERANCE	10068652	22
HEPATITIS A VIRUS TEST	10068815	22
PRODUCT LOT NUMBER ISSUE	10069266	22
BLADDER DYSFUNCTION	10069632	22
DOUBLE STRANDED DNA ANTIBODY POSITIVE	10069907	22
KLEBSIELLA TEST POSITIVE	10070091	22
VARICELLA VIRUS TEST	10070444	22
CRYPTOCOCCUS TEST	10070456	22
COLD-STIMULUS HEADACHE	10071151	22
LOWER URINARY TRACT SYMPTOMS	10071289	22
NASAL DISCHARGE DISCOLOURATION	10071553	22
HAEMOPHAGOCYTIC LYMPHOHISTIOCYTOSIS	10071583	22
SKIN WOUND	10072170	22
HYPOOSMOLAR STATE	10074867	22
VACCINATION SITE JOINT PAIN	10076176	22
CRITICAL ILLNESS	10077264	22
NEUROMYELITIS OPTICA SPECTRUM DISORDER	10077875	22
INTRA-ABDOMINAL FLUID COLLECTION	10078659	22
PARAESTHESIA EAR	10052433	22
ACQUIRED DIAPHRAGMATIC EVENTRATION	10059185	22
STOOL ANALYSIS ABNORMAL	10059527	22
HISTAMINE LEVEL INCREASED	10060051	22
ABDOMINAL ABSCESS	10060921	22
ULTRASOUND THYROID NORMAL	10060988	22
CSF WHITE BLOOD CELL COUNT	10061098	22
CHEST INJURY	10061386	22
GENE MUTATION IDENTIFICATION TEST NEGATIVE	10063479	22
JOINT INSTABILITY	10064931	22

ORTHOSTATIC HYPERTENSION	10065508	22
ACUTE PULMONARY OEDEMA	10001029	21
ANAEMIA MACROCYTIC	10002064	21
APTYALISM	10003068	21
ARTERIAL THROMBOSIS	10003178	21
BIOPSY MUSCLE	10004802	21
BLOOD FIBRINOGEN DECREASED	10005520	21
BLOOD GASES NORMAL	10005540	21
COLON CANCER	10009944	21
CORTISOL NORMAL	10011209	21
DEAFNESS TRANSITORY	10011900	21
ENDOMETRIAL HYPERPLASIA	10014755	21
GASTROINTESTINAL INFECTION	10017964	21
GENERALISED ANXIETY DISORDER	10018075	21
LIP ULCERATION	10024572	21
MYELOPATHY	10028570	21
OBSESSIVE-COMPULSIVE DISORDER	10029898	21
PERICARDIAL EXCISION	10034475	21
PERIPHERAL ISCHAEMIA	10034576	21
PLACENTAL DISORDER	10035132	21
PO2 INCREASED	10035769	21
POLYNEUROPATHY	10036105	21
PROTHROMBIN TIME SHORTENED	10037070	21
SKIN ATROPHY	10040799	21
SPINAL FRACTURE	10041569	21
TRI-IODOTHYRONINE DECREASED	10044594	21
ULTRASOUND BLADDER	10045406	21
HYPERVIGILANCE	10048533	21
ALCOHOL USE	10048921	21
ANAL HAEMORRHAGE	10049555	21
URETERAL STENT INSERTION	10050307	21
MONOCYTE PERCENTAGE DECREASED	10052229	21
AMNIORRHOEA	10066470	21
SUPERFICIAL VEIN PROMINENCE	10068717	21
BASAL GANGLIA INFARCTION	10069020	21
PRODUCT EXPIRATION DATE ISSUE	10069271	21
EHRLICHIA TEST	10070399	21
VULVOVAGINAL RASH	10071588	21
VERTEBRAL ARTERY DISSECTION	10071716	21
OESOPHAGOGASTRODUODENOSCOPY NORMAL	10072164	21
PRONE POSITION	10074744	21
VACCINATION SITE DYSAESTHESIA	10076160	21
COMPRESSION GARMENT APPLICATION	10079209	21

WRONG PATIENT RECEIVED PRODUCT	10081577	21
PHANTOM LIMB SYNDROME	10082544	21
ORGAN FAILURE	10053159	21
TRI-IODOTHYRONINE FREE NORMAL	10053793	21
THYROXINE FREE DECREASED	10055162	21
EYE LASER SURGERY	10057105	21
AORTIC DILATATION	10057453	21
VIRAL TEST POSITIVE	10059424	21
FRACTION OF INSPIRED OXYGEN	10059883	21
PERIPHERAL EMBOLISM	10061340	21
RHINOVIRUS INFECTION	10061494	21
BIOPSY UTERUS	10061697	21
DEMYELINATING POLYNEUROPATHY	10061811	21
PULMONARY FUNCTION TEST DECREASED	10061922	21
BRAIN SCAN ABNORMAL	10061943	21
INCREASED BRONCHIAL SECRETION	10062530	21
INTERLEUKIN LEVEL	10063321	21
ULTRASOUND JOINT	10063568	21
DEVICE MALFUNCTION	10063829	21
BRONCHIAL DISORDER	10064913	21
LOOSE TOOTH	10065612	21
MYOCARDIAL INJURY	10085879	21
ABDOMINAL MASS	10000077	20
AORTIC THROMBOSIS	10002910	20
APLASTIC ANAEMIA	10002967	20
BEHCET'S SYNDROME	10004213	20
BLOOD COPPER	10005439	20
BLOOD IMMUNOGLOBULIN A NORMAL	10005587	20
BLOOD OESTROGEN DECREASED	10005687	20
BREAST ENGORGEMENT	10006240	20
CARDIOLIPIN ANTIBODY POSITIVE	10007630	20
CERVICAL SPINAL STENOSIS	10008313	20
CREATININE RENAL CLEARANCE	10011371	20
DEREALISATION	10012422	20
DREAMY STATE	10013634	20
ENDOSCOPY UPPER GASTROINTESTINAL TRACT ABNORMAL	10014820	20
GLUCOSE URINE PRESENT	10018478	20
HAEMANGIOMA	10018814	20
HEPATIC ENCEPHALOPATHY	10019660	20
HYPERKERATOSIS	10020649	20
INTRAVENTRICULAR HAEMORRHAGE	10022840	20
LARYNGOSPASM	10023891	20
MASSAGE	10026871	20

MENINGITIS VIRAL	10027260	20
NECROSIS	10028851	20
OESOPHAGEAL ACHALASIA	10030136	20
OPEN REDUCTION OF FRACTURE	10030682	20
OPHTHALMOPLEGIA	10030875	20
OVULATION DELAYED	10033310	20
PATHOGEN RESISTANCE	10034133	20
PREMATURE MENOPAUSE	10036601	20
RUPTURED CEREBRAL ANEURYSM	10039330	20
SKIN CANCER	10040808	20
SPLEEN DISORDER	10041633	20
URINARY CASTS	10046533	20
VASCULAR INJURY	10047080	20
VOCAL CORD PARALYSIS	10047674	20
TACHYARRHYTHMIA	10049447	20
INJECTION SITE COLDNESS	10050082	20
LIGAMENT PAIN	10068790	20
INTRADUCTAL PROLIFERATIVE BREAST LESION	10073094	20
VARICELLA ZOSTER VIRUS INFECTION	10075611	20
GLUTEN SENSITIVITY	10076493	20
ORAL CONTUSION	10078170	20
HERPES SIMPLEX REACTIVATION	10080137	20
SARS-COV-1 TEST POSITIVE	10084446	20
POLYPECTOMY	10053308	20
CARDIAC TELEMETRY NORMAL	10053449	20
CANALITH REPOSITIONING PROCEDURE	10053470	20
TYPE III IMMUNE COMPLEX MEDIATED REACTION	10053614	20
IMMUNOSUPPRESSANT DRUG THERAPY	10054980	20
MUSCLE MASS	10056720	20
HIV ANTIBODY NEGATIVE	10058762	20
BLUE TOE SYNDROME	10059126	20
GALACTOSTASIS	10060710	20
COMPLEMENT FACTOR	10061055	20
METASTATIC NEOPLASM	10061289	20
ABDOMEN SCAN	10061936	20
THYROIDECTOMY	10062127	20
SPINAL OPERATION	10062262	20
PERIPHERAL ARTERIAL OCCLUSIVE DISEASE	10062585	20
REACTION TO PRESERVATIVES	10064788	20
INFLAMMATORY PAIN	10065390	20
ANIMAL BITE	10002515	19
ARTERIOSPASM CORONARY	10003225	19
BARIUM SWALLOW	10004124	19

BASOPHIL COUNT INCREASED	10004169	19
BILIRUBIN CONJUGATED INCREASED	10004685	19
BLOOD CREATINE PHOSPHOKINASE MB INCREASED	10005474	19
BLOOD PRESSURE DIASTOLIC	10005735	19
BLOOD THYROID STIMULATING HORMONE ABNORMAL	10005830	19
CACHEXIA	10006895	19
CARTILAGE INJURY	10007710	19
CIRCULATORY COLLAPSE	10009192	19
CULTURE WOUND	10011641	19
DUPUYTREN'S CONTRACTURE	10013872	19
DYSPNOEA PAROXYSMAL NOCTURNAL	10013974	19
ECZEMA NUMMULAR	10014201	19
ERYTHROMELALGIA	10015284	19
GAMMA-GLUTAMYLTRANSFERASE	10017687	19
GRUNTING	10018762	19
HAEMORRHAGE SUBCUTANEOUS	10018999	19
HALLUCINATIONS, MIXED	10019079	19
HAND FRACTURE	10019114	19
HUMERUS FRACTURE	10020462	19
INTESTINAL PERFORATION	10022694	19
MICROCYTIC ANAEMIA	10027538	19
ORAL CONTRACEPTION	10030970	19
PULMONARY HAEMORRHAGE	10037394	19
PULMONARY VALVE INCOMPETENCE	10037448	19
PUPILLARY LIGHT REFLEX TESTS ABNORMAL	10037525	19
REPETITIVE SPEECH	10038583	19
RETICULOCYTE COUNT	10038787	19
SHOCK SYMPTOM	10040581	19
SYNOVITIS	10042868	19
ULTRASOUND EYE	10045416	19
VENTILATION/PERFUSION SCAN ABNORMAL	10047265	19
VENTILATION/PERFUSION SCAN NORMAL	10047266	19
ANTIPHOSPHOLIPID ANTIBODIES POSITIVE	10048678	19
PERICARDIAL FIBROSIS	10048724	19
THERMAL BURNS OF EYE	10049267	19
SHOCK HAEMORRHAGIC	10049771	19
HAEMODYNAMIC INSTABILITY	10052076	19
CAROTID ARTERIOSCLEROSIS	10067116	19
VACCINATION SITE HAEMATOMA	10069472	19
SUBSTANCE USE	10070964	19
PUPIL DILATION PROCEDURE	10072352	19
OCCUPATIONAL EXPOSURE TO PRODUCT	10073311	19
ABDOMINAL LYMPHADENOPATHY	10073485	19

INTENTIONAL PRODUCT MISUSE	10074903	19
CEREBRAL CONGESTION	10076929	19
LACUNAR STROKE	10076994	19
PRODUCT DISPENSING ISSUE	10081771	19
INSULIN THERAPY	10085221	19
DIURETIC THERAPY	10053073	19
SLEEP STUDY ABNORMAL	10053320	19
ESCHERICHIA BACTERAEMIA	10054258	19
NASOENDOSCOPY	10055050	19
ALLERGY TO ARTHROPOD BITE	10058285	19
OVARIAN MASS	10058823	19
THERAPEUTIC PRODUCT INEFFECTIVE	10060769	19
DNA ANTIBODY	10061131	19
DRUG LEVEL	10061823	19
OCCULT BLOOD NEGATIVE	10061879	19
GALLBLADDER ENLARGEMENT	10062693	19
IMMUNE COMPLEX ASSAY	10063326	19
CATARACT OPERATION	10063797	19
BONE CONTUSION	10066251	19
ULTRASOUND LYMPH NODES	10085635	19
AMMONIA NORMAL	10001947	18
ANGINA UNSTABLE	10002388	18
BIOPSY COLON ABNORMAL	10004760	18
BLOOD FIBRINOGEN NORMAL	10005522	18
BLOOD IRON INCREASED	10005620	18
BLOOD OSMOLARITY NORMAL	10005698	18
BRAIN STEM INFARCTION	10006147	18
BREECH PRESENTATION	10006356	18
BURNS SECOND DEGREE	10006802	18
CHRONIC SINUSITIS	10009137	18
COR PULMONALE ACUTE	10010969	18
CYSTOSCOPY ABNORMAL	10011815	18
DEATH OF RELATIVE	10011931	18
DENTAL CARIES	10012318	18
DISTRACTIBILITY	10013486	18
DYSHIDROTIC ECZEMA	10013913	18
FACE AND MOUTH X-RAY	10016018	18
FAECES HARD	10016101	18
GANGRENE	10017711	18
HYPOVITAMINOSIS	10021135	18
MIXED CONNECTIVE TISSUE DISEASE	10027754	18
MONOPARESIS	10027925	18
PROLONGED LABOUR	10036872	18

RESPIRATORY ACIDOSIS	10038661	18
RETICULOCYTE COUNT INCREASED	10038792	18
RETINAL EXUDATES	10038862	18
SICKLE CELL ANAEMIA WITH CRISIS	10040642	18
SUBDURAL HAEMORRHAGE	10042364	18
TIBIA FRACTURE	10043827	18
UTERINE DISORDER	10046779	18
VITAMIN B6	10047613	18
VOCAL CORD DYSFUNCTION	10047671	18
VAGINAL LESION	10048937	18
CREATININE URINE	10049696	18
SPINAL FUSION SURGERY	10050505	18
MITRAL VALVE CALCIFICATION	10050558	18
HIP SURGERY	10051060	18
CYTOMEGALOVIRUS TEST POSITIVE	10051620	18
BLOOD CREATINE PHOSPHOKINASE DECREASED	10067760	18
UTERINE DILATION AND EVACUATION	10067801	18
FASTING	10068315	18
USE OF ACCESSORY RESPIRATORY MUSCLES	10069555	18
CHLAMYDIA TEST	10070272	18
PARVOVIRUS B19 TEST NEGATIVE	10070365	18
PARVOVIRUS B19 TEST	10070366	18
STAPHYLOCOCCUS TEST	10070412	18
UROBILINOGEN URINE INCREASED	10070479	18
DRUG REACTION WITH EOSINOPHILIA AND SYSTEMIC SYMPTO	10073508	18
VACCINATION SITE COLDNESS	10076157	18
URINARY TRACT DISCOMFORT	10076568	18
HEMIANAESTHESIA	10077170	18
URETEROLITHIASIS	10077989	18
TONSILLAR ERYTHEMA	10078256	18
PERFUSION BRAIN SCAN NORMAL	10079704	18
LUNG ASSIST DEVICE THERAPY	10082527	18
GASTROINTESTINAL PATHOGEN PANEL	10082666	18
ULTRASOUND OVARY	10052602	18
INHALATION THERAPY	10052996	18
MEDICAL OBSERVATION	10053047	18
BILIARY DILATATION	10057202	18
FACTOR V LEIDEN MUTATION	10058279	18
SPINAL DEFORMITY	10058907	18
VENOUS OCCLUSION	10058990	18
URINE OUTPUT	10059894	18
CARDIOVASCULAR FUNCTION TEST	10060858	18
PH BODY FLUID	10061346	18

ANTIBODY TEST NORMAL	10061429	18
RENAL INJURY	10061481	18
PARACENTESIS	10061905	18
GENITAL LESION	10061978	18
RENAL MASS	10062104	18
PARASITE BLOOD TEST	10063195	18
PROGESTERONE DECREASED	10063271	18
ALLERGIC RESPIRATORY SYMPTOM	10063527	18
VITAL FUNCTIONS ABNORMAL	10063644	18
BLOOD COUNT ABNORMAL	10064198	18
ABORTION THREATENED	10000242	17
BIOPSY BONE MARROW NORMAL	10004739	17
BIOPSY LYMPH GLAND NORMAL	10004799	17
BLOOD ALBUMIN INCREASED	10005288	17
BLOOD PROLACTIN INCREASED	10005780	17
BLOOD TESTOSTERONE NORMAL	10005816	17
BRONCHITIS CHRONIC	10006458	17
CHALAZION	10008388	17
ELECTROPHORESIS PROTEIN NORMAL	10014469	17
ENDOSCOPY UPPER GASTROINTESTINAL TRACT NORMAL	10014821	17
FEMORAL NECK FRACTURE	10016450	17
FIBROSIS	10016642	17
FOREIGN TRAVEL	10017059	17
GRANULOCYTE COUNT	10018678	17
HAEMOGLOBIN ABNORMAL	10018879	17
HAEMORRHOIDS THROMBOSED	10019023	17
IDIOPATHIC URTICARIA	10021247	17
IGA NEPHROPATHY	10021263	17
LEG AMPUTATION	10024124	17
LOGORRHOEA	10024796	17
LUMBAR SPINAL STENOSIS	10025005	17
MYOPIA	10028651	17
NIPPLE DISORDER	10029417	17
PALMAR ERYTHEMA	10033551	17
PARASITE STOOL TEST NEGATIVE	10033904	17
PLEOCYTOSIS	10035551	17
PO2 NORMAL	10035770	17
POSTURING	10036437	17
PUPILS UNEQUAL	10037538	17
RIGHT VENTRICULAR FAILURE	10039163	17
SALIVARY GLAND PAIN	10039421	17
SALPINGECTOMY	10039449	17
SKIN DEPIGMENTATION	10040825	17

SLIT-LAMP EXAMINATION	10041031	17
SPEECH DISORDER DEVELOPMENTAL	10041467	17
TARDIVE DYSKINESIA	10043118	17
TENDON INJURY	10043242	17
UTERINE CANCER	10046766	17
VASOSPASM	10047163	17
X-RAY WITH CONTRAST UPPER GASTROINTESTINAL TRACT	10048210	17
SACRAL PAIN	10048710	17
WEIGHT FLUCTUATION	10049040	17
PNEUMOMEDIASTINUM	10050184	17
HEPATITIS B SURFACE ANTIGEN	10050529	17
BLOOD PRESSURE INADEQUATELY CONTROLLED	10051128	17
GENITAL DISCOMFORT	10066887	17
JOINT FLUID DRAINAGE	10066994	17
DEVICE ISSUE	10069868	17
MYCOPLASMA TEST	10070411	17
VULVOVAGINAL ERYTHEMA	10070437	17
UROBILINOGEN URINE DECREASED	10070480	17
SUBCHORIONIC HAEMORRHAGE	10071010	17
HUMAN CHORIONIC GONADOTROPIN ABNORMAL	10071330	17
OVARIAN VEIN THROMBOSIS	10072059	17
PRETERM PREMATURE RUPTURE OF MEMBRANES	10073024	17
IMMUNISATION ANXIETY RELATED REACTION	10075205	17
THUNDERCLAP HEADACHE	10075481	17
VACCINATION SITE INJURY	10076169	17
VACCINATION SITE ANAESTHESIA	10076192	17
STENOSIS	10076711	17
JAW CLICKING	10077089	17
REACTION TO EXCIPIENT	10079925	17
MAGNETIC RESONANCE IMAGING THORACIC NORMAL	10083141	17
PRODUCT DOSE OMISSION IN ERROR	10084404	17
SPLINT APPLICATION	10053312	17
INTESTINAL RESECTION	10054193	17
INTERVERTEBRAL DISC SPACE NARROWING	10055041	17
BREAST CANCER METASTATIC	10055113	17
DEHYDROEPIANDROSTERONE TEST	10056642	17
WOUND CLOSURE	10057679	17
REFLUX GASTRITIS	10057969	17
METABOLIC DISORDER	10058097	17
ULTRASOUND TESTES	10058847	17
OXYGEN CONSUMPTION	10059167	17
BLOOD SMEAR TEST ABNORMAL	10059665	17
CYTOGENETIC ANALYSIS	10059882	17

ANTINEUTROPHIL CYTOPLASMIC ANTIBODY POSITIVE	10060136	17
ELECTROCARDIOGRAM ST-T CHANGE	10061117	17
PELVIC FRACTURE	10061161	17
KLEBSIELLA INFECTION	10061259	17
OOPHORECTOMY	10061889	17
BLADDER SCAN	10061940	17
TUMOUR MARKER TEST	10062216	17
CYSTITIS NONINFECTIVE	10063057	17
EOSINOPHILIC OESOPHAGITIS	10064212	17
PELVIC DISCOMFORT	10064229	17
GENE MUTATION	10064571	17
CAPILLARY NAIL REFILL TEST	10065130	17
VISCERAL PAIN	10065385	17
PRODUCT AFTER TASTE	10086446	17
ALLERGY TO ANIMAL	10001742	16
AMBLYOPIA	10001906	16
ANGULAR CHEILITIS	10002509	16
AORTIC VALVE REPLACEMENT	10002916	16
ASPHYXIA	10003497	16
ASTHMA EXERCISE INDUCED	10003557	16
AXILLARY VEIN THROMBOSIS	10003880	16
CSF CELL COUNT	10011520	16
CULTURE WOUND POSITIVE	10011643	16
CYSTOSCOPY NORMAL	10011816	16
FAECES PALE	10016102	16
GAMMA-GLUTAMYLTRANSFERASE INCREASED	10017693	16
HALO VISION	10019099	16
HYDROCEPHALUS	10020508	16
HYPERREFLEXIA	10020745	16
HYPOVOLAEMIC SHOCK	10021138	16
LARGE INTESTINE PERFORATION	10023804	16
MEASLES	10027011	16
MENINGIOMA	10027191	16
METASTASES TO LYMPH NODES	10027459	16
MUSCLE RUPTURE	10028331	16
OESOPHAGEAL STENOSIS	10030194	16
OVARIAN CANCER	10033128	16
PO2	10035766	16
RENAL TRANSPLANT	10038533	16
SPINAL CORD COMPRESSION	10041549	16
SUICIDE ATTEMPT	10042464	16
TETANY	10043380	16
THYROXINE DECREASED	10043816	16

TUBERCULOSIS	10044755	16
VENTRICULAR HYPERTROPHY	10047295	16
URTICARIAL VASCULITIS	10048820	16
VISUAL BRIGHTNESS	10049155	16
RED BLOOD CELL SEDIMENTATION RATE ABNORMAL	10049186	16
GINGIVAL PRURITUS	10049306	16
LEFT VENTRICULAR DILATATION	10050043	16
LUMBAR RADICULOPATHY	10050219	16
LUPUS-LIKE SYNDROME	10050551	16
PULMONARY CALCIFICATION	10051200	16
INJECTION SITE MACULE	10067255	16
WITHDRAWAL OF LIFE SUPPORT	10067595	16
GENITAL SWELLING	10067639	16
BRAIN STEM STROKE	10068644	16
ENTEROCOCCUS TEST POSITIVE	10070024	16
ENTEROVIRUS TEST	10070396	16
LOSS OF BLADDER SENSATION	10070632	16
INTENTIONAL MEDICAL DEVICE REMOVAL BY PATIENT	10070773	16
BASAL GANGLIA STROKE	10071043	16
VIRAL SEPSIS	10071362	16
N-TERMINAL PROHORMONE BRAIN NATRIURETIC PEPTIDE NO	10071659	16
BOTULINUM TOXIN INJECTION	10073539	16
VACCINATION SITE EXTRAVASATION	10076163	16
NEISSERIA TEST	10082453	16
MAGNETIC RESONANCE IMAGING BREAST ABNORMAL	10083124	16
FOREIGN BODY IN THROAT	10083433	16
SARS-COV-2 TEST FALSE POSITIVE	10084602	16
PALATAL DISORDER	10052453	16
ENTERAL NUTRITION	10052591	16
VENTRICULAR DRAINAGE	10052947	16
FLUID INTAKE RESTRICTION	10053623	16
SHIFT TO THE LEFT	10056383	16
PERIORBITAL CELLULITIS	10057182	16
HAEMATOLOGY TEST NORMAL	10057756	16
ADMINISTRATION SITE PAIN	10058049	16
OXYGEN CONSUMPTION DECREASED	10059165	16
CYTOLOGY NORMAL	10059531	16
BASE EXCESS	10059961	16
PLATELET FACTOR 4	10060192	16
ANTI-THYROID ANTIBODY NEGATIVE	10060311	16
COMPUTERISED TOMOGRAM CORONARY ARTERY NORMAL	10060805	16
ABDOMINAL HERNIA	10060954	16
DIAPHRAGMATIC DISORDER	10061106	16

ENTEROCOCCAL INFECTION	10061124	16
PSYCHIATRIC SYMPTOM	10061472	16
DEFORMITY	10061619	16
CHONDROPATHY	10061762	16
PANCREATIC ENZYMES INCREASED	10061900	16
BREATH SOUNDS ABSENT	10062285	16
THYROID HORMONES INCREASED	10063161	16
DEVICE RELATED INFECTION	10064687	16
NIH STROKE SCALE	10065527	16
SARS-COV-2 RNA INCREASED	10085495	16
POLYMERS ALLERGY	10086347	16
ADENOCARCINOMA	10001141	15
ARTERIAL CATHETERISATION	10003148	15
BASAL CELL CARCINOMA	10004146	15
BLOOD OSMOLARITY	10005693	15
BLOOD PARATHYROID HORMONE	10005699	15
BLOOD PRESSURE IMMEASURABLE	10005748	15
BLOOD PRESSURE SYSTOLIC DECREASED	10005758	15
BRONCHIOLITIS	10006448	15
CARDIAC FAILURE CHRONIC	10007558	15
CARDIOSPASM	10007645	15
CEREBROSPINAL FLUID LEAKAGE	10008164	15
COLOSTOMY	10010041	15
CORONARY ARTERY STENOSIS	10011089	15
CSF OLIGOCLONAL BAND PRESENT	10011562	15
ENDOSCOPIC RETROGRADE CHOLANGIOPANCREATOGRAPHY	10014802	15
FLAT AFFECT	10016759	15
GASTRIC HAEMORRHAGE	10017788	15
GINGIVAL DISCOLOURATION	10018278	15
GLOMERULONEPHRITIS	10018364	15
GRAM STAIN NEGATIVE	10018655	15
HAEMANGIOMA OF SKIN	10018823	15
IIIRD NERVE PARALYSIS	10021283	15
IMPETIGO	10021531	15
INJECTION SITE NERVE DAMAGE	10022083	15
IVTH NERVE PARALYSIS	10023110	15
KERATITIS	10023332	15
KYPHOSIS	10023509	15
LACTOSE INTOLERANCE	10023681	15
MALIGNANT MELANOMA	10025650	15
METASTASES TO LIVER	10027457	15
MORPHOEA	10027982	15
MYOPATHY	10028641	15

OVARIAN ENLARGEMENT	10033157	15
PERIORAL DERMATITIS	10034541	15
PERITONITIS	10034674	15
PHARYNGEAL HAEMORRHAGE	10034827	15
PHLEBITIS SUPERFICIAL	10034902	15
POSTOPERATIVE WOUND INFECTION	10036410	15
PREGNANCY TEST URINE POSITIVE	10036578	15
PULMONARY GRANULOMA	10037391	15
RADIAL NERVE PALSY	10037751	15
SKULL X-RAY	10040964	15
TEETHING	10043183	15
THROMBOTIC STROKE	10043647	15
VASCULITIC RASH	10047111	15
RED BLOOD CELLS URINE NEGATIVE	10048546	15
SCAR PAIN	10049002	15
ANAPHYLAXIS PROPHYLAXIS	10049090	15
CARBOHYDRATE ANTIGEN 125	10049248	15
PHARYNGEAL MASS	10049270	15
ONYCHOMADESIS	10049274	15
SUDDEN ONSET OF SLEEP	10050014	15
ANTI-NEUTROPHIL CYTOPLASMIC ANTIBODY POSITIVE VASCUL	10050894	15
COMPLEMENT FACTOR C4 DECREASED	10050983	15
PREMENSTRUAL DYSPHORIC DISORDER	10051537	15
VASOGENIC CEREBRAL OEDEMA	10067275	15
REFLEX TEST NORMAL	10068272	15
COMA SCALE	10069708	15
BORRELIA TEST POSITIVE	10070011	15
WALKING DISTANCE TEST	10071033	15
INJECTION RELATED REACTION	10071152	15
SYSTOLIC DYSFUNCTION	10071436	15
TIME PERCEPTION ALTERED	10074073	15
TENDON DISCOMFORT	10074599	15
ADMINISTRATION SITE BRUISE	10075094	15
WEST NILE VIRUS TEST	10075808	15
SINUS NODE DYSFUNCTION	10075889	15
PRODUCT LEAKAGE	10076232	15
FEEDING TUBE USER	10076368	15
ADAMTS13 ACTIVITY ASSAY	10076768	15
CYTOKINE TEST	10077352	15
BILIRUBIN URINE PRESENT	10077356	15
NOTHING BY MOUTH ORDER	10080668	15
CHILDHOOD ASTHMA	10081274	15
INTRAUTERINE CONTRACEPTION	10082352	15

MAGNETIC RESONANCE IMAGING BREAST	10083122	15
ORBITAL SWELLING	10085251	15
STATUS MIGRAINOSUS	10052945	15
OESOPHAGEAL DISCOMFORT	10053634	15
FACET JOINT SYNDROME	10054813	15
MUCOSAL DISCOLOURATION	10054877	15
PHONOPHOBIA	10054956	15
TOXOPLASMA SEROLOGY NEGATIVE	10056299	15
ABDOMINAL INFECTION	10056519	15
DAYDREAMING	10057315	15
HIV ANTIBODY	10058761	15
HIV ANTIGEN	10058763	15
MYOGLOBIN BLOOD	10059884	15
HAEMORRHAGIC OVARIAN CYST	10060781	15
BLOOD ELECTROLYTES ABNORMAL	10061014	15
BREAST DISORDER	10061021	15
CSF WHITE BLOOD CELL COUNT NEGATIVE	10061099	15
HEART INJURY	10061200	15
TUMOUR EXCISION	10061392	15
BLOOD ELECTROLYTES DECREASED	10061715	15
HISTOLOGY	10062005	15
THYROID GLAND SCAN ABNORMAL	10062149	15
ACARODERMATITIS	10063409	15
SPONDYLOLISTHESIS	10063550	15
BLOOD COUNT NORMAL	10064197	15
ULCERATIVE KERATITIS	10064996	15
PHARYNGEAL INFLAMMATION	10065716	15
AORTIC DISSECTION	10002895	14
BILE DUCT STONE	10004637	14
BIOPSY BRAIN	10004741	14
BLOOD OESTROGEN INCREASED	10005688	14
BLOOD OSMOLARITY INCREASED	10005697	14
BLOOD ZINC	10005864	14
BREAST DISCOLOURATION	10006228	14
BREAST OEDEMA	10006294	14
CEREBRAL ARTERY EMBOLISM	10008088	14
CEREBRAL PALSY	10008129	14
CHOLANGITIS	10008604	14
CHONDROCALCINOSIS PYROPHOSPHATE	10008690	14
CONTRAINDICATION TO VACCINATION	10010835	14
CSF OLIGOCLONAL BAND	10011558	14
ELECTROCARDIOGRAM PR SHORTENED	10014374	14
EMBOLISM ARTERIAL	10014513	14

HAPTOGLOBIN DECREASED	10019150	14
HIV TEST FALSE POSITIVE	10020186	14
HODGKIN'S DISEASE	10020206	14
IMPULSIVE BEHAVIOUR	10021567	14
LACK OF SPONTANEOUS SPEECH	10023615	14
LOSS OF EMPLOYMENT	10024861	14
MOUTH BREATHING	10028017	14
NEPHROPATHY	10029151	14
OVERWEIGHT	10033307	14
POLYHYDRAMNIOS	10036079	14
SALIVA ALTERED	10039379	14
SLEEP TALKING	10041009	14
SQUAMOUS CELL CARCINOMA	10041823	14
TENOSYNOVITIS	10043261	14
TWIN PREGNANCY	10045188	14
VISUAL FIELD TESTS ABNORMAL	10047567	14
POSTPARTUM STATE	10048738	14
LUMBAR VERTEBRAL FRACTURE	10049947	14
WOUND DRAINAGE	10050325	14
CORONARY ANGIOPLASTY	10050329	14
HEPATITIS B SURFACE ANTIGEN NEGATIVE	10050542	14
WHITE BLOOD CELLS URINE	10050663	14
SUPRAPUBIC PAIN	10050822	14
POST PROCEDURAL HAEMORRHAGE	10051077	14
CONJUNCTIVAL HYPERAEMIA	10051625	14
STICKY SKIN	10051788	14
TREATMENT FAILURE	10066901	14
BASAL GANGLIA HAEMORRHAGE	10067057	14
ORGANISING PNEUMONIA	10067472	14
WEST NILE VIRUS TEST NEGATIVE	10068813	14
GASTROINTESTINAL EXAMINATION	10068978	14
GENE SEQUENCING	10069604	14
TOXICITY TO VARIOUS AGENTS	10070863	14
WALKING DISTANCE TEST ABNORMAL	10071032	14
EYELID SKIN DRYNESS	10071717	14
COMPUTERISED TOMOGRAM KIDNEY ABNORMAL	10072171	14
ENCEPHALITIS AUTOIMMUNE	10072378	14
INCENTIVE SPIROMETRY	10072425	14
SENSITIVITY TO WEATHER CHANGE	10072850	14
BARTONELLA TEST NEGATIVE	10075208	14
COATING IN MOUTH	10075366	14
GASTROINTESTINAL BACTERIAL OVERGROWTH	10078158	14
DEFAECATION DISORDER	10079938	14

AST/ALT RATIO	10079980	14
LICHEN PLANOPILARIS	10081142	14
NASAL MUCOSAL BLISTERING	10082185	14
CYST REMOVAL	10052938	14
PATIENT RESTRAINT	10053316	14
HEAT THERAPY	10053324	14
VASCULAR RUPTURE	10053649	14
CEREBRAL HAEMATOMA	10053942	14
TRANSAMINASES	10054888	14
IN VITRO FERTILISATION	10056204	14
POST CONCUSSION SYNDROME	10057230	14
MULTI-ORGAN DISORDER	10058092	14
ALLERGY TO ARTHROPOD STING	10058284	14
AORTIC DISORDER	10058648	14
LIGHT CHAIN ANALYSIS	10060083	14
ANALGESIC DRUG LEVEL	10060090	14
PARASITE STOOL TEST	10060812	14
APPENDIX DISORDER	10060960	14
PSEUDOMONAS INFECTION	10061471	14
ARTERY DISSECTION	10061660	14
BREAST OPERATION	10061734	14
CYTOLOGY	10061804	14
DISEASE PROGRESSION	10061818	14
WHOLE BODY SCAN	10062151	14
SHOULDER OPERATION	10062242	14
CHOLECYSTITIS INFECTIVE	10062631	14
LIGAMENT RUPTURE	10065433	14
MULTISYSTEM INFLAMMATORY SYNDROME	10086091	14
THYROID HORMONES TEST	10086270	14
ACUTE FEBRILE NEUTROPHILIC DERMATOSIS	10000748	13
AMMONIA INCREASED	10001946	13
BIOPSY LUNG ABNORMAL	10004795	13
BRAIN STEM HAEMORRHAGE	10006145	13
BREAST CELLULITIS	10006217	13
CAPILLARY DISORDER	10007189	13
CHROMATOPSIA	10008795	13
CHROMOSOME ANALYSIS NORMAL	10008818	13
CLONUS	10009346	13
CORTISOL INCREASED	10011207	13
CSF GLUCOSE	10011535	13
DYSLEXIA	10013932	13
EHLERS-DANLOS SYNDROME	10014316	13
ENDOMETRIAL DISORDER	10014753	13

EPHELIDES	10014970	13
GLOMERULONEPHRITIS MINIMAL LESION	10018374	13
HAIR GROWTH ABNORMAL	10019044	13
HAND-FOOT-AND-MOUTH DISEASE	10019113	13
HEPATITIS ACUTE	10019727	13
HEPATITIS C	10019744	13
HIGH DENSITY LIPOPROTEIN INCREASED	10020061	13
HYPERTHERMIA	10020843	13
HYPERTONIA	10020852	13
ILEOSTOMY	10021321	13
IMMUNOGLOBULINS NORMAL	10021501	13
LABILE BLOOD PRESSURE	10023533	13
LIPIDS INCREASED	10024592	13
LYMPHANGITIS	10025226	13
MACROCYTOSIS	10025382	13
MALABSORPTION	10025476	13
NEOPLASM SKIN	10029098	13
OLIGURIA	10030302	13
OVARIAN DISORDER	10033139	13
PARKINSONISM	10034010	13
PERTUSSIS	10034738	13
POSITRON EMISSION TOMOGRAM NORMAL	10036222	13
RADIUS FRACTURE	10037802	13
RENAL STONE REMOVAL	10038528	13
SCINTILLATING SCOTOMA	10039677	13
SEBORRHOEIC DERMATITIS	10039793	13
SMALL INTESTINAL RESECTION	10041105	13
SPINAL LAMINECTOMY	10041576	13
THYROID CYST	10043706	13
TONGUE GEOGRAPHIC	10043957	13
TOURETTE'S DISORDER	10044126	13
UPPER MOTOR NEURONE LESION	10046298	13
URETHRAL PAIN	10046461	13
THORACIC OUTLET SYNDROME	10048627	13
VERTEBRAL LESION	10048877	13
ALLERGIC SINUSITIS	10049153	13
INJECTION SITE JOINT SWELLING	10049260	13
BRONCHOALVEOLAR LAVAGE	10049413	13
TONGUE HAEMORRHAGE	10049870	13
SELF-INJURIOUS IDEATION	10051154	13
CENTRAL NERVOUS SYSTEM INFLAMMATION	10051288	13
WHITE COAT HYPERTENSION	10051581	13
ACUTE CORONARY SYNDROME	10051592	13

NUMB CHIN SYNDROME	10066930	13
INTRANASAL HYPOAESTHESIA	10067068	13
POST PROCEDURAL INFECTION	10067268	13
NEUROLOGICAL DECOMPENSATION	10068357	13
TRAUMATIC LUNG INJURY	10069363	13
ENTEROVIRUS TEST POSITIVE	10070386	13
EPIDURAL INJECTION	10072041	13
GRANULOMATOSIS WITH POLYANGIITIS	10072579	13
RENAL REPLACEMENT THERAPY	10074746	13
GENITAL BLISTER	10074995	13
CSF RED BLOOD CELL COUNT	10075561	13
VACCINATION SITE STREAKING	10076189	13
CEREBRAL ENDOVASCULAR ANEURYSM REPAIR	10077079	13
PREMATURE MENARCHE	10077320	13
FUNDUS AUTOFLUORESCENCE	10078792	13
WALL MOTION SCORE INDEX ABNORMAL	10079016	13
PANCREATIC FAILURE	10079281	13
HERPES ZOSTER REACTIVATION	10080516	13
SINONASAL OBSTRUCTION	10080545	13
ACUTE CARDIAC EVENT	10081099	13
INCORRECT PRODUCT ADMINISTRATION DURATION	10081581	13
GINGIVAL ABSCESS	10052359	13
HIGH RISK PREGNANCY	10052744	13
ALCOHOL WITHDRAWAL SYNDROME	10053164	13
HEARING DISABILITY	10053194	13
INAPPROPRIATE ANTIDIURETIC HORMONE SECRETION	10053198	13
FOETAL MONITORING	10053278	13
BLOOD PRESSURE ORTHOSTATIC	10053352	13
CARDIAC VENTRICULAR THROMBOSIS	10053994	13
GENITAL BURNING SENSATION	10054815	13
ELECTROLYTE SUBSTITUTION THERAPY	10054977	13
LOSS OF PROPRIOCEPTION	10057332	13
RIGHT ATRIAL ENLARGEMENT	10058227	13
CARDIAC FUNCTION TEST ABNORMAL	10058479	13
URINE KETONE BODY	10059222	13
RIGHT VENTRICULAR SYSTOLIC PRESSURE	10060199	13
NEOPLASM PROGRESSION	10061309	13
POISONING	10061355	13
UTERINE CONTRACTIONS ABNORMAL	10061400	13
PULMONARY VASCULAR DISORDER	10061474	13
ABNORMAL SLEEP-RELATED EVENT	10061613	13
CYST RUPTURE	10061803	13
DRUG INTOLERANCE	10061822	13

OBSTRUCTION	10061876	13
PHYSICAL EXAMINATION ABNORMAL	10062078	13
URINARY TRACT PAIN	10062225	13
SLEEP DISORDER DUE TO A GENERAL MEDICAL CONDITION	10063910	13
ONYCHALGIA	10064251	13
KERATOSIS PILARIS	10066295	13
COVID-19 SCREENING	10085530	13
AKINESIA	10001541	12
ALBUMIN URINE	10001575	12
ALCOHOL INTOLERANCE	10001598	12
ALOPECIA UNIVERSALIS	10001767	12
ANGIOGRAM RETINA	10002443	12
ANORECTAL DISORDER	10002644	12
ANURIA	10002847	12
BILIRUBIN CONJUGATED NORMAL	10004686	12
BIOPSY BREAST NORMAL	10004746	12
BLEEDING TIME PROLONGED	10005140	12
BLOOD IMMUNOGLOBULIN M NORMAL	10005602	12
BLOOD PARATHYROID HORMONE INCREASED	10005703	12
BREAST ABSCESS	10006171	12
BUNDLE BRANCH BLOCK	10006578	12
CEREBRAL ARTERY THROMBOSIS	10008092	12
CHANGE IN SUSTAINED ATTENTION	10008398	12
CHEST CRUSHING	10008468	12
CHRONIC GASTRITIS	10008882	12
COMPRESSION FRACTURE	10010214	12
CORNEAL ABRASION	10010984	12
CSF LYMPHOCYTE COUNT INCREASED	10011549	12
DIABETIC NEUROPATHY	10012680	12
DUODENITIS	10013864	12
ENDOMETRIAL CANCER	10014733	12
ENTEROCOLITIS	10014893	12
EYE ALLERGY	10015907	12
EYELID INFECTION	10015988	12
FIBROCYSTIC BREAST DISEASE	10016621	12
FIBULA FRACTURE	10016667	12
GLUCOSE URINE	10018436	12
GLYCOSYLATED HAEMOGLOBIN DECREASED	10018482	12
HAEMOPERITONEUM	10018935	12
HYPERBILIRUBINAEMIA	10020578	12
HYPERPHAGIA	10020710	12
HYPERTENSIVE HEART DISEASE	10020823	12
INFERTILITY	10021926	12

INJECTION SITE CYST	10022055	12
LUPUS NEPHRITIS	10025140	12
MEAN CELL HAEMOGLOBIN CONCENTRATION INCREASED	10026992	12
MENORRHAGIA	10027313	12
MILK ALLERGY	10027633	12
MYOCARDIAL FIBROSIS	10028594	12
NIGHT BLINDNESS	10029404	12
OBSTRUCTIVE SLEEP APNOEA SYNDROME	10029983	12
OPTIC NERVE INJURY	10030938	12
PANCREATIC CARCINOMA	10033609	12
PANNICULITIS	10033675	12
PH URINE	10034791	12
PITYRIASIS	10035110	12
PNEUMONIA KLEBSIELLA	10035717	12
PNEUMONIA STAPHYLOCOCCAL	10035734	12
PNEUMONIA STREPTOCOCCAL	10035735	12
SEBORRHOEA	10039792	12
SIGMOIDOSCOPY	10040669	12
SMOOTH MUSCLE ANTIBODY	10041227	12
TONGUE SPASM	10043981	12
TONIC CONVULSION	10043994	12
VAGINAL ODOUR	10046935	12
VOLVULUS	10047697	12
TERMINAL STATE	10048669	12
UROSEPSIS	10048709	12
VERTEBRAL ARTERY OCCLUSION	10048965	12
INTRA-UTERINE CONTRACEPTIVE DEVICE REMOVAL	10049481	12
SHOULDER ARTHROPLASTY	10049551	12
SPUTUM PURULENT	10050090	12
BLOOD PHOSPHORUS INCREASED	10050196	12
DENTAL EXAMINATION	10050316	12
CAROTID ARTERY DISSECTION	10050403	12
LYMPH GLAND INFECTION	10050823	12
PROTEIN S NORMAL	10051737	12
BONE MARROW OEDEMA	10051763	12
THYROID CANCER	10066474	12
ULTRASOUND OVARY ABNORMAL	10067168	12
RIGHT ATRIAL DILATATION	10067282	12
ABDOMINAL WALL HAEMATOMA	10067383	12
ANAL PRURITUS	10068172	12
TERMINAL INSOMNIA	10068932	12
INFERIOR VENA CAVA DILATATION	10069111	12
PRODUCT COLOUR ISSUE	10069221	12

PSEUDOMONAS TEST POSITIVE	10070135	12
STRESS ECHOCARDIOGRAM ABNORMAL	10070746	12
GASTROINTESTINAL TRACT IRRITATION	10070840	12
POSTERIOR REVERSIBLE ENCEPHALOPATHY SYNDROME	10071066	12
HUMAN CHORIONIC GONADOTROPIN INCREASED	10071332	12
MULTIPLE USE OF SINGLE-USE PRODUCT	10072342	12
RHEUMATIC DISORDER	10072736	12
FEMALE SEX HORMONE LEVEL	10072841	12
TRIPLE NEGATIVE BREAST CANCER	10075566	12
VACCINATION SITE JOINT ERYTHEMA	10076177	12
HELPLESSNESS	10077169	12
PROCALCITONIN DECREASED	10077830	12
HLA-B*27 POSITIVE	10078451	12
CERVICAL DILATATION	10079273	12
X-RAY DENTAL ABNORMAL	10079993	12
PRODUCT DISPENSING ERROR	10080359	12
TISSUE INJURY	10080903	12
LARGE INTESTINE INFECTION	10081959	12
ANAESTHESIA ORAL	10082548	12
PELVIC FLOOR DYSFUNCTION	10083246	12
URINARY OCCULT BLOOD NEGATIVE	10084961	12
OCULAR VASCULAR DISORDER	10052896	12
FOETAL NON-STRESS TEST	10053108	12
THORACIC CAVITY DRAINAGE	10053975	12
DEPRESSIVE SYMPTOM	10054089	12
THROAT LESION	10054830	12
TENOSYNOVITIS STENOSANS	10056698	12
DENTAL CARE	10056723	12
RECTAL TENESMUS	10057071	12
DYSLIPIDAEMIA	10058108	12
OCULAR ICTERUS	10058117	12
BIOPSY ARTERY NORMAL	10058393	12
NEGATIVE THOUGHTS	10058672	12
DERMATITIS PSORIASIFORM	10058675	12
SKIN OEDEMA	10058679	12
NIPPLE SWELLING	10058680	12
EARLY SATIETY	10059186	12
IMMUNOHISTOCHEMISTRY	10059656	12
CYTOGENETIC ANALYSIS NORMAL	10059886	12
POSITIVE END-EXPIRATORY PRESSURE	10059890	12
LEFT VENTRICULAR END-DIASTOLIC PRESSURE INCREASED	10060088	12
CALCIUM IONISED NORMAL	10060897	12
COMPLEMENT FACTOR NORMAL	10061056	12

SKULL FRACTURE	10061365	12
SOFT TISSUE DISORDER	10061366	12
BONE LESION	10061728	12
BONE MARROW DISORDER	10061729	12
CARDIAC ELECTROPHYSIOLOGIC STUDY	10061739	12
EATING DISORDER SYMPTOM	10061832	12
PURULENCE	10061926	12
VASCULAR TEST	10062172	12
CHEST WALL MASS	10063004	12
AMINO ACID LEVEL	10063258	12
CARBON DIOXIDE ABNORMAL	10064156	12
EPIPLOIC APPENDAGITIS	10064231	12
CEREBRAL CALCIFICATION	10066296	12
ANISOCYTOSIS	10002536	11
BARTHOLIN'S CYST	10004140	11
BIOPSY CERVIX	10004753	11
BIOPSY ENDOMETRIUM ABNORMAL	10004770	11
BLOOD CATECHOLAMINES	10005411	11
BLOOD CHOLESTEROL DECREASED	10005424	11
BLOOD CREATINE PHOSPHOKINASE MB NORMAL	10005475	11
BLOOD FOLATE INCREASED	10005528	11
BLOOD TESTOSTERONE INCREASED	10005815	11
BODY TINEA	10005913	11
CARDIAC ANEURYSM	10007513	11
CATAPLEXY	10007737	11
CAUTERY TO NOSE	10007829	11
CHEMICAL BURN	10008420	11
COOMBS DIRECT TEST	10010929	11
COR PULMONALE	10010968	11
CORNEAL OEDEMA	10011033	11
CORTISOL DECREASED	10011198	11
CRYSTAL URINE PRESENT	10011512	11
ELECTRIC SHOCK	10014357	11
ELECTROCARDIOGRAM QRS COMPLEX ABNORMAL	10014378	11
ELECTROPHORESIS	10014458	11
EUSTACHIAN TUBE OBSTRUCTION	10015544	11
FEAR OF DISEASE	10016278	11
FIBROADENOMA OF BREAST	10016613	11
FRACTURED SACRUM	10017308	11
FULL BLOOD COUNT DECREASED	10017413	11
GLOMERULONEPHRITIS RAPIDLY PROGRESSIVE	10018378	11
HAEMANGIOMA OF LIVER	10018821	11
HAIR COLOUR CHANGES	10019030	11

HALLUCINATION, OLFACTORY	10019072	11
HEPATITIS A ANTIBODY	10019721	11
HYPERTROPHY OF TONGUE PAPILLAE	10020893	11
HYPOPHOSPHATAEMIA	10021058	11
INJECTION SITE ULCER	10022105	11
INSPIRATORY CAPACITY DECREASED	10022448	11
INSULIN RESISTANCE	10022489	11
INTERMITTENT CLAUDICATION	10022562	11
LAPAROSCOPY	10023693	11
LYMPHOCYTOSIS	10025280	11
MACULAR OEDEMA	10025415	11
MEDIASTINAL MASS	10027076	11
METASTASES TO BONE	10027452	11
METRORRHAGIA	10027514	11
MICTURITION DISORDER	10027561	11
NEUROMYOPATHY	10029323	11
NON-HODGKIN'S LYMPHOMA	10029547	11
PELVIC ABSCESS	10034236	11
PEPTIC ULCER	10034341	11
PERFUME SENSITIVITY	10034434	11
PLACENTA PRAEVIA	10035119	11
PLATELET COUNT ABNORMAL	10035526	11
PROCTITIS	10036774	11
PULMONARY ARTERY THROMBOSIS	10037340	11
REBOUND EFFECT	10038001	11
RED BLOOD CELL COUNT ABNORMAL	10038151	11
SERUM SICKNESS-LIKE REACTION	10040402	11
SKIN NECROSIS	10040893	11
SKIN STRIAE	10040925	11
STUPOR	10042264	11
SUBCUTANEOUS EMPHYSEMA	10042344	11
TONGUE PARALYSIS	10043972	11
TONGUE ROUGH	10043977	11
TYPE I HYPERSENSITIVITY	10045240	11
UMBILICAL HERNIA	10045458	11
URINE FLOW DECREASED	10046640	11
WATER POLLUTION	10047838	11
WITHDRAWAL SYNDROME	10048010	11
TUBULOINTERSTITIAL NEPHRITIS	10048302	11
KIDNEY ENLARGEMENT	10048469	11
ATRIAL THROMBOSIS	10048632	11
RED BLOOD CELL SEDIMENTATION RATE DECREASED	10049188	11
INTRA-UTERINE CONTRACEPTIVE DEVICE INSERTION	10049480	11

BLOOD HOMOCYSTEINE NORMAL	10049736	11
CERVICAL VERTEBRAL FRACTURE	10049946	11
AMMONIA	10050287	11
CAST APPLICATION	10050305	11
CERULOPLASMIN	10050646	11
LOWER GASTROINTESTINAL HAEMORRHAGE	10050953	11
BIOPSY ARTERY	10051409	11
HYPOAESTHESIA TEETH	10051780	11
ALLERGY TO METALS	10066414	11
RETROPERITONEAL LYMPHADENOPATHY	10067015	11
TEMPERATURE DIFFERENCE OF EXTREMITIES	10068016	11
BONE DENSITY ABNORMAL	10068789	11
PRODUCT CONTAMINATION PHYSICAL	10069176	11
COMA SCALE ABNORMAL	10069709	11
HELICOBACTER TEST POSITIVE	10070101	11
BURNING FEET SYNDROME	10070237	11
MYCOBACTERIUM TEST NEGATIVE	10070408	11
NEOVASCULAR AGE-RELATED MACULAR DEGENERATION	10071129	11
GIARDIA TEST NEGATIVE	10072429	11
SUBCHORIONIC HAEMATOMA	10072596	11
OPTICAL COHERENCE TOMOGRAPHY ABNORMAL	10073561	11
SPONTANEOUS HAEMORRHAGE	10074557	11
NASAL HERPES	10074936	11
RHESUS ANTIGEN POSITIVE	10076520	11
INTRUSIVE THOUGHTS	10077275	11
SPEECH SOUND DISORDER	10077803	11
ORAL SOFT TISSUE BIOPSY	10078906	11
VISUAL SNOW SYNDROME	10079450	11
SENSORY OVERLOAD	10079780	11
PARANASAL SINUS HYPOSECRETION	10080046	11
COMPLICATED APPENDICITIS	10081534	11
PULMONARY IMAGING PROCEDURE ABNORMAL	10082582	11
FLUORESCENCE ANGIOGRAM ABNORMAL	10083087	11
FOLLICULAR LYMPHOMA	10085128	11
SECOND TRIMESTER PREGNANCY	10052396	11
SKIN BACTERIAL INFECTION	10052891	11
FRACTURE DISPLACEMENT	10053206	11
SLEEP STUDY NORMAL	10053321	11
VASCULAR OCCLUSION	10053648	11
TRI-IODOTHYRONINE FREE INCREASED	10053790	11
BURSA DISORDER	10056337	11
GYNAECOLOGICAL EXAMINATION NORMAL	10056829	11
INTERVERTEBRAL DISC OPERATION	10057322	11

CARDIAC VALVE VEGETATION	10057651	11
JUVENILE IDIOPATHIC ARTHRITIS	10059176	11
ENDOCRINE TEST	10059689	11
ANTICOAGULATION DRUG LEVEL ABOVE THERAPEUTIC	10060320	11
AUTOANTIBODY NEGATIVE	10060974	11
PAPILLOMA VIRAL INFECTION	10061331	11
PERINEAL PAIN	10061339	11
ARTERIOGRAM	10061658	11
BLOOD PH	10061724	11
GASTRECTOMY	10061965	11
C1 ESTERASE INHIBITOR TEST	10062730	11
MYASTHENIA GRAVIS CRISIS	10062758	11
GENE MUTATION IDENTIFICATION TEST POSITIVE	10063478	11
COAGULATION TEST ABNORMAL	10063557	11
CEREBRAL ARTERY STENOSIS	10063648	11
TONSILLOLITH	10063957	11
NON-HIGH-DENSITY LIPOPROTEIN CHOLESTEROL	10063984	11
HORMONE THERAPY	10065646	11
INJECTION SITE PALLOR	10066041	11
ABNORMAL WEIGHT GAIN	10000188	10
ABSCESS ORAL	10000311	10
ACUTE PSYCHOSIS	10001022	10
ALPHA 1 FOETOPROTEIN	10001772	10
AMYLASE DECREASED	10002014	10
ANGLE CLOSURE GLAUCOMA	10002500	10
AORTIC VALVE STENOSIS	10002918	10
APNOEIC ATTACK	10002977	10
APPLICATION SITE PAIN	10003051	10
BARIUM SWALLOW ABNORMAL	10004125	10
BENIGN BREAST NEOPLASM	10004243	10
BIOPSY COLON	10004759	10
BLOOD COPPER NORMAL	10005444	10
BLOOD CORTICOTROPHIN	10005450	10
BLOOD IMMUNOGLOBULIN E NORMAL	10005592	10
BLOOD LUTEINISING HORMONE DECREASED	10005648	10
BLOOD LUTEINISING HORMONE INCREASED	10005649	10
CEREBROVASCULAR DISORDER	10008196	10
COMPLEMENT FACTOR C3 INCREASED	10010126	10
COUGH DECREASED	10011225	10
CSF CELL COUNT NORMAL	10011523	10
CSF PRESSURE INCREASED	10011570	10
DISSOCIATIVE DISORDER	10013462	10
ENZYME ABNORMALITY	10014935	10

EPINEPHRINE INCREASED	10015064	10
HAEMORRHAGIC DISORDER	10019009	10
HAEMOTHORAX	10019027	10
HEPATIC VEIN THROMBOSIS	10019713	10
HEPATITIS B	10019731	10
HYPERMETROPIA	10020675	10
HYPOCHLORAEMIA	10020955	10
HYPOCHROMIC ANAEMIA	10020969	10
INTENTIONAL OVERDOSE	10022523	10
KETOACIDOSIS	10023379	10
LABIA ENLARGED	10023527	10
LIBIDO DECREASED	10024419	10
MASTOIDITIS	10026900	10
NEPHRITIS	10029117	10
OESTRADIOL INCREASED	10030231	10
OESTRADIOL NORMAL	10030233	10
ORBITAL OEDEMA	10031051	10
OXYGEN SATURATION INCREASED	10033320	10
PATELLA FRACTURE	10034122	10
PCO2	10034180	10
PERNICIOUS ANAEMIA	10034695	10
PLEURAL THICKENING	10035616	10
PNEUMONIA PSEUDOMONAL	10035731	10
POLYCHROMASIA	10036040	10
POLYCYTHAEMIA	10036051	10
REFLEXES ABNORMAL	10038254	10
RENAL VEIN THROMBOSIS	10038548	10
RETINAL OEDEMA	10038886	10
SCLERAL DISCOLOURATION	10039696	10
SPLENECTOMY	10041642	10
STILL'S DISEASE	10042061	10
SUTURE REMOVAL	10042640	10
TACHYCARDIA FOETAL	10043074	10
TELANGIECTASIA	10043189	10
THYROTOXIC CRISIS	10043786	10
TOBACCO ABUSE	10043903	10
VENTRICULAR ARRHYTHMIA	10047281	10
VESTIBULAR FUNCTION TEST NORMAL	10047391	10
VIRAL MYOCARDITIS	10047470	10
VISUAL ACUITY TESTS ABNORMAL	10047534	10
ANEURYSM RUPTURED	10048380	10
ORAL INFECTION	10048685	10
UNDERWEIGHT	10048828	10

UTERINE CYST	10048931	10
ANTIACETYLCHOLINE RECEPTOR ANTIBODY POSITIVE	10049144	10
SUDDEN CARDIAC DEATH	10049418	10
ABNORMAL CLOTTING FACTOR	10049862	10
THORACIC VERTEBRAL FRACTURE	10049948	10
ELECTROCARDIOGRAM PR INTERVAL	10050656	10
SPECIFIC GRAVITY URINE INCREASED	10050773	10
MACULAR HOLE	10051058	10
ENCEPHALOMALACIA	10051818	10
FOETAL CARDIAC DISORDER	10052088	10
CONTRAST MEDIA ALLERGY	10066973	10
HAEMOSTASIS	10067439	10
HOSPICE CARE	10067973	10
AXILLARY LYMPHADENECTOMY	10068477	10
ANISOMASTIA	10068732	10
MANUAL LYMPHATIC DRAINAGE	10068967	10
LIQUID PRODUCT PHYSICAL ISSUE	10069224	10
VACCINATION SITE ABSCESS	10069556	10
PROTEUS TEST POSITIVE	10070134	10
HYPOXIC-ISCHAEMIC ENCEPHALOPATHY	10070511	10
HEPARIN-INDUCED THROMBOCYTOPENIA TEST POSITIVE	10070664	10
MEDICAL INDUCTION OF COMA	10070677	10
HEPATITIS B CORE ANTIBODY NEGATIVE	10071345	10
BANDAEMIA	10072081	10
DRUG-INDUCED LIVER INJURY	10072268	10
FAECAL CALPROTECTIN INCREASED	10072482	10
HEPATIC CANCER	10073069	10
JOINT VIBRATION	10074327	10
SCALLOPED TONGUE	10074686	10
EYELID CONTUSION	10075018	10
BARTONELLA TEST	10075209	10
NONINFECTIVE SIALOADENITIS	10075243	10
QUANTITATIVE SUDOMOTOR AXON REFLEX TEST	10075351	10
SWALLOW STUDY ABNORMAL	10076037	10
CHA2DS2-VASC-SCORE	10076965	10
MULTIPATHOGEN PCR TEST	10077018	10
INFLAMMATORY MARKER DECREASED	10077676	10
PATIENT UNCOOPERATIVE	10077706	10
DECOMPRESSIVE CRANIECTOMY	10078092	10
SKIN CULTURE	10078401	10
HOUSEBOUND	10079226	10
MITRAL VALVE THICKENING	10079336	10
PERFUSION BRAIN SCAN	10079705	10

SALMONELLA TEST NEGATIVE	10079858	10
ALCOHOL TEST NEGATIVE	10081298	10
ROBOTIC SURGERY	10082954	10
MAGNETIC RESONANCE IMAGING ABDOMINAL NORMAL	10083135	10
COGNITIVE TEST	10083267	10
ULTRASOUND SPLEEN	10083327	10
EPSTEIN-BARR VIRUS ANTIGEN POSITIVE	10052363	10
ELECTROCARDIOGRAM REPOLARISATION ABNORMALITY	10052464	10
VASCULAR IMAGING	10052677	10
MYCOTIC ALLERGY	10052758	10
MEDICAL DEVICE REMOVAL	10052971	10
BRONCHITIS VIRAL	10053160	10
ARTHRITIS BACTERIAL	10053555	10
WOUND COMPLICATION	10053692	10
URINARY TRACT INFECTION BACTERIAL	10054088	10
EPIDURAL BLOOD PATCH	10055034	10
HAEMORRHAGIC TRANSFORMATION STROKE	10055677	10
ADENOMYOSIS	10056268	10
ASYMPTOMATIC BACTERIURIA	10056396	10
MONOCLONAL ANTIBODY UNCONJUGATED THERAPY	10056417	10
CUTANEOUS LUPUS ERYTHEMATOSUS	10056509	10
ORAL PUSTULE	10056674	10
SKIN LAXITY	10057064	10
VOCAL CORD INFLAMMATION	10057295	10
INCISION SITE PAIN	10058043	10
GASTROINTESTINAL OEDEMA	10058061	10
PULMONARY ARTERY DILATATION	10058491	10
INFREQUENT BOWEL MOVEMENTS	10059158	10
NITRITE URINE	10059220	10
SEDATIVE THERAPY	10059283	10
BAND NEUTROPHIL PERCENTAGE	10059468	10
RUSSELL'S VIPER VENOM TIME	10059775	10
TONGUE INJURY	10059924	10
PARASITIC BLOOD TEST NEGATIVE	10059992	10
COMPUTERISED TOMOGRAM CORONARY ARTERY ABNORMAL	10060806	10
ARTERIAL STENOSIS	10060965	10
AUTOANTIBODY POSITIVE	10060973	10
SPUTUM ABNORMAL	10060991	10
ORAL FUNGAL INFECTION	10061324	10
VAGINAL DISORDER	10061402	10
AUTONOMIC NEUROPATHY	10061666	10
SALIVARY GLAND DISORDER	10061935	10
GALLBLADDER OPERATION	10061962	10

HERNIA REPAIR	10062003	10
LYMPHOCYTE MORPHOLOGY ABNORMAL	10062047	10
METASTASIS	10062194	10
TONGUE EXFOLIATION	10064488	10
PULMONARY ARTERIAL HYPERTENSION	10064911	10
URINARY TRACT INFLAMMATION	10064921	10
PERIVASCULAR DERMATITIS	10064986	10
PELVIC FLUID COLLECTION	10065388	10
BRACHIAL PLEXOPATHY	10065417	10
HEPATOBILIARY SCAN ABNORMAL	10066195	10
ACCELERATED HYPERTENSION	10000358	9
ACUTE LYMPHOCYTIC LEUKAEMIA	10000846	9
ADRENAL ADENOMA	10001323	9
ALDOLASE NORMAL	10001643	9
ANAESTHESIA	10002091	9
ARNOLD-CHIARI MALFORMATION	10003101	9
BIOPSY THYROID GLAND NORMAL	10004890	9
BLOOD ALDOSTERONE	10005293	9
BLOOD IMMUNOGLOBULIN A INCREASED	10005586	9
BLOOD LUTEINISING HORMONE NORMAL	10005650	9
BLOOD POTASSIUM ABNORMAL	10005722	9
BLOOD PRESSURE DIASTOLIC DECREASED	10005737	9
BREAST NEOPLASM	10006279	9
BREATH HOLDING	10006322	9
CALCULUS URINARY	10007027	9
CHRONIC MYELOID LEUKAEMIA	10009013	9
CROUP INFECTIOUS	10011416	9
CSF GLUCOSE DECREASED	10011537	9
CSF OLIGOCLONAL BAND ABSENT	10011560	9
CULTURE STOOL POSITIVE	10011631	9
CULTURE WOUND NEGATIVE	10011642	9
CYTOMEGALOVIRUS INFECTION	10011831	9
DAIRY INTOLERANCE	10011852	9
DEFECT CONDUCTION INTRAVENTRICULAR	10012118	9
DERMATOCHALASIS	10012493	9
DRUG ABUSE	10013654	9
ENDOCRINE DISORDER	10014695	9
EXERCISE ELECTROCARDIOGRAM ABNORMAL	10015645	9
GRANULOCYTE COUNT INCREASED	10018683	9
HAEMOCHROMATOSIS	10018872	9
HAPTOGLOBIN NORMAL	10019154	9
HYDROPS FOETALIS	10020529	9
INTENTIONAL SELF-INJURY	10022524	9

IRON BINDING CAPACITY TOTAL DECREASED	10022963	9
JUDGEMENT IMPAIRED	10023236	9
LAPAROTOMY	10023696	9
LARGE INTESTINAL ULCER	10023799	9
LIPASE DECREASED	10024573	9
LIPIDS ABNORMAL	10024588	9
MENARCHE	10027182	9
MITRAL VALVE STENOSIS	10027733	9
MYELODYSPLASTIC SYNDROME	10028533	9
NASAL POLYPS	10028756	9
OBSTRUCTION GASTRIC	10029957	9
OESOPHAGEAL DILATATION	10030164	9
OESOPHAGEAL ULCER	10030201	9
OLIGOHYDRAMNIOS	10030289	9
ONYCHOLYSIS	10030337	9
OROPHARYNGEAL SWELLING	10031118	9
OSTEONECROSIS	10031264	9
PARAPLEGIA	10033892	9
PARESIS	10033985	9
PELVIC INFLAMMATORY DISEASE	10034254	9
PLICATED TONGUE	10035630	9
PNEUMONIA MYCOPLASMAL	10035724	9
PNEUMONIA PNEUMOCOCCAL	10035728	9
PULMONARY ALVEOLAR HAEMORRHAGE	10037313	9
SACROILIITIS	10039361	9
SEXUALLY TRANSMITTED DISEASE	10040490	9
SINUS ARREST	10040738	9
SKIN HYPOPIGMENTATION	10040868	9
SPECIFIC GRAVITY URINE	10041438	9
SPINAL CORD INJURY CERVICAL	10041554	9
SQUAMOUS CELL CARCINOMA OF SKIN	10041834	9
TORSADE DE POINTES	10044066	9
TRANSPLANT REJECTION	10044439	9
TYMPANOMETRY ABNORMAL	10045215	9
UTERINE ENLARGEMENT	10046782	9
UTERINE INFLAMMATION	10046793	9
VENTRICULAR SEPTAL DEFECT	10047298	9
VERY LOW DENSITY LIPOPROTEIN	10047351	9
PNEUMOPERITONEUM	10048299	9
ISCHAEMIC CARDIOMYOPATHY	10048858	9
VASCULAR PSEUDOANEURYSM	10048975	9
HELLP SYNDROME	10049058	9
PANCREATIC MASS	10049082	9

PSYCHOMOTOR SKILLS IMPAIRED	10049215	9
GINGIVAL ULCERATION	10049398	9
PUBIC PAIN	10049747	9
MEDICAL DEVICE IMPLANTATION	10049812	9
IMPATIENCE	10049976	9
ENEMA ADMINISTRATION	10050314	9
RIGHT VENTRICULAR ENLARGEMENT	10050582	9
CSF GLUCOSE INCREASED	10050763	9
COMPLEMENT FACTOR C4 INCREASED	10050982	9
STREPTOCOCCAL BACTERAEMIA	10051018	9
CARBOHYDRATE ANTIGEN 125 INCREASED	10051414	9
BONE EROSION	10051728	9
REFLUX LARYNGITIS	10067869	9
SALT CRAVING	10067951	9
CAPILLARY NAIL REFILL TEST ABNORMAL	10069381	9
BED SHARING	10069595	9
COXSACKIE VIRUS TEST	10070394	9
CANDIDA TEST POSITIVE	10070451	9
BAND SENSATION	10070714	9
SOFT TISSUE MASS	10071051	9
SMEAR VAGINAL NORMAL	10071320	9
ANTI-TRANSGLUTAMINASE ANTIBODY	10071472	9
FOETAL NON-STRESS TEST ABNORMAL	10071516	9
ENZYME LEVEL INCREASED	10072234	9
BENZODIAZEPINE DRUG LEVEL	10072337	9
ANTI-TRANSGLUTAMINASE ANTIBODY NEGATIVE	10072526	9
CHRONIC PIGMENTED PURPURA	10072726	9
HYSTEROSCOPY NORMAL	10074342	9
CANCER SURGERY	10074431	9
SPLENIC THROMBOSIS	10074601	9
VACCINATION SITE DRYNESS	10076159	9
COMPUTERISED TOMOGRAM LIVER	10076215	9
RHESUS ANTIGEN NEGATIVE	10076521	9
EYE PARAESTHESIA	10076569	9
COMPUTERISED TOMOGRAM INTESTINE	10077359	9
FOETAL GROWTH ABNORMALITY	10077582	9
LIVER FUNCTION TEST DECREASED	10077677	9
SOMATIC SYMPTOM DISORDER	10078076	9
OPHTHALMIC SCAN	10078104	9
BUTTOCK INJURY	10078480	9
PERSISTENT POSTURAL-PERCEPTUAL DIZZINESS	10079170	9
VULVOVAGINAL INFLAMMATION	10079372	9
FOCAL DYSCOGNITIVE SEIZURES	10079424	9

OBSTRUCTIVE PANCREATITIS	10079822	9
EXPLODING HEAD SYNDROME	10080684	9
URINARY SEDIMENT	10080828	9
CENTRAL VISION LOSS	10081186	9
SUBACUTE INFLAMMATORY DEMYELINATING POLYNEUROPAT	10081726	9
IMMATURE GRANULOCYTE PERCENTAGE INCREASED	10082033	9
ORAL MUCOSAL ROUGHENING	10084009	9
ABNORMAL CORD INSERTION	10084854	9
THIRD TRIMESTER PREGNANCY	10052397	9
SALINE INFUSION SONOGRAM	10052604	9
EMERGENCY CARE EXAMINATION	10053069	9
PHYSICAL BREAST EXAMINATION	10053478	9
URINE ALBUMIN/CREATININE RATIO	10053542	9
LIMB DEFORMITY	10053652	9
HAEMORRHOIDAL HAEMORRHAGE	10054787	9
LYMPH NODE CALCIFICATION	10056284	9
INTRA-ABDOMINAL HAEMATOMA	10056457	9
DENTAL EXAMINATION NORMAL	10056827	9
GYNAECOLOGICAL EXAMINATION ABNORMAL	10056830	9
THERAPEUTIC EMBOLISATION	10057335	9
BLOOD KETONE BODY INCREASED	10057594	9
QRS AXIS	10057622	9
TOTAL CHOLESTEROL/HDL RATIO	10058632	9
BREAST MILK DISCOLOURATION	10058665	9
HIV ANTIGEN NEGATIVE	10058764	9
ADNEXA UTERI MASS	10058897	9
INTESTINAL HAEMORRHAGE	10059175	9
METASTASES TO CENTRAL NERVOUS SYSTEM	10059282	9
RADICULAR PAIN	10059604	9
PLASMINOGEN ACTIVATOR INHIBITOR	10059620	9
BLOOD SMEAR TEST NORMAL	10059664	9
COAGULATION FACTOR	10059744	9
LEFT VENTRICULAR END-DIASTOLIC PRESSURE	10060065	9
RED BLOOD CELL MORPHOLOGY NORMAL	10060197	9
INTERVERTEBRAL DISCITIS	10060738	9
DUODENOGASTRIC REFLUX	10060865	9
CALCIUM IONISED DECREASED	10060898	9
DNA ANTIBODY NEGATIVE	10061109	9
ECONOMIC PROBLEM	10061114	9
ELECTROCARDIOGRAM CHANGE	10061116	9
MASTOID DISORDER	10061277	9
VAGUS NERVE DISORDER	10061403	9
CORNEAL DISORDER	10061453	9

ULCER HAEMORRHAGE	10061577	9
FACIAL NEURALGIA	10061594	9
ARTERIAL STENT INSERTION	10061657	9
CAROTID ARTERY DISEASE	10061744	9
DENTAL OPERATION	10061812	9
LITHOTRIPSY	10062039	9
LARGE INTESTINAL OBSTRUCTION	10062062	9
TYMPANIC MEMBRANE DISORDER	10062218	9
GRIP STRENGTH	10062557	9
UTERINE MASS	10062662	9
ANKLE BRACHIAL INDEX	10062762	9
PROGESTERONE NORMAL	10063273	9
ENDOTRACHEAL INTUBATION COMPLICATION	10063349	9
EYELID EXFOLIATION	10064580	9
MEIBOMIAN GLAND DYSFUNCTION	10065062	9
EXTENSIVE SWELLING OF VACCINATED LIMB	10065106	9
MEDIAL TIBIAL STRESS SYNDROME	10065303	9
NIH STROKE SCALE ABNORMAL	10065531	9
EXTERNAL EAR INFLAMMATION	10065837	9
STRESS URINARY INCONTINENCE	10066218	9
MULTISYSTEM INFLAMMATORY SYNDROME IN ADULTS	10085850	9
SPONTANEOUS RUPTURE OF MEMBRANES	10087390	9
ALANINE AMINOTRANSFERASE DECREASED	10001549	8
ALDOLASE INCREASED	10001642	8
AMNIOCENTESIS NORMAL	10001963	8
AMYLOIDOSIS	10002022	8
ANTIMICROBIAL SUSCEPTIBILITY TEST	10002791	8
AORTIC VALVE SCLEROSIS	10002917	8
ASTIGMATISM	10003569	8
BACTERIURIA	10004056	8
BARIUM SWALLOW NORMAL	10004126	8
BARRETT'S OESOPHAGUS	10004137	8
BIOPSY HEART	10004779	8
BIOPSY SITE UNSPECIFIED ABNORMAL	10004871	8
BIPOLAR I DISORDER	10004939	8
BLADDER DILATATION	10005033	8
BLADDER IRRITATION	10005052	8
BLISTER INFECTED	10005192	8
BLOOD ETHANOL NORMAL	10005516	8
BLOOD PARATHYROID HORMONE NORMAL	10005704	8
BRONCHIAL IRRITATION	10006438	8
BRONCHOSCOPY NORMAL	10006481	8
BURNING SENSATION MUCOSAL	10006789	8

BURNS THIRD DEGREE	10006803	8
CALCINOSIS	10006938	8
CEREBELLAR HAEMORRHAGE	10008030	8
CHOREA	10008748	8
CIRCADIAN RHYTHM SLEEP DISORDER	10009191	8
COAGULATION FACTOR V LEVEL	10009752	8
COLD URTICARIA	10009869	8
COMPARTMENT SYNDROME	10010121	8
COMPLETED SUICIDE	10010144	8
CONJUNCTIVAL OEDEMA	10010726	8
CSF LYMPHOCYTE COUNT NORMAL	10011550	8
DERMATITIS DIAPER	10012444	8
DIAPHRAGMATIC PARALYSIS	10012725	8
DRUG SPECIFIC ANTIBODY ABSENT	10013744	8
ELECTROCARDIOGRAM QRS COMPLEX	10014377	8
EMOTIONAL POVERTY	10014557	8
ENDOSCOPY GASTROINTESTINAL	10014809	8
ERYTHEMA ANNULARE	10015153	8
ESSENTIAL THROMBOCYTHAEMIA	10015493	8
EYE INFECTION BACTERIAL	10015930	8
FEELINGS OF WORTHLESSNESS	10016374	8
GLOMERULONEPHRITIS MEMBRANOUS	10018372	8
GLUCOSE TOLERANCE TEST NORMAL	10018435	8
HEAT EXHAUSTION	10019332	8
HIV TEST POSITIVE	10020188	8
HYPERAEMIA	10020565	8
HYPERCHLORHYDRIA	10020601	8
HYPOHIDROSIS	10021013	8
INCREASED INSULIN REQUIREMENT	10021664	8
INJECTION SITE THROMBOSIS	10022104	8
INSULIN C-PEPTIDE	10022474	8
INTESTINAL DILATATION	10022642	8
INTUSSUSCEPTION	10022863	8
JOB DISSATISFACTION	10023193	8
LARYNGOSCOPY ABNORMAL	10023889	8
MALIGNANT HYPERTENSION	10025600	8
MEAN CELL VOLUME ABNORMAL	10027000	8
MULTIPLE FRACTURES	10028200	8
NASAL SEPTUM DEVIATION	10028762	8
NON-CONSUMMATION	10029540	8
OEDEMA MOUTH	10030110	8
ORAL MUCOSAL DISCOLOURATION	10030996	8
OTITIS MEDIA ACUTE	10033079	8

PANCREATITIS CHRONIC	10033649	8
PANCREATITIS NECROTISING	10033654	8
PAPILLARY THYROID CANCER	10033701	8
PATHOLOGICAL FRACTURE	10034156	8
PELVIC MASS	10034260	8
PERITONSILLAR ABSCESS	10034686	8
PNEUMOTHORAX SPONTANEOUS	10035763	8
POLYMYOSITIS	10036102	8
PUPILLARY DISORDER	10037521	8
RESPIRATORY ALKALOSIS	10038664	8
RETINAL VEIN THROMBOSIS	10038908	8
RETROPERITONEAL HAEMORRHAGE	10038980	8
RUBBER SENSITIVITY	10039251	8
SOCIAL AVOIDANT BEHAVIOUR	10041243	8
SOFT TISSUE INFLAMMATION	10041290	8
SPINAL CORD INJURY	10041552	8
SPIROMETRY NORMAL	10041631	8
SPLENIC RUPTURE	10041658	8
SPLENIC VEIN THROMBOSIS	10041659	8
SPLINTER HAEMORRHAGES	10041663	8
STARVATION	10041954	8
SUDDEN VISUAL LOSS	10042441	8
SYSTEMIC LUPUS ERYTHEMATOSUS RASH	10042946	8
THERAPEUTIC RESPONSE DECREASED	10043414	8
ULTRASOUND BLADDER NORMAL	10045408	8
URINARY HESITATION	10046542	8
VASOCONSTRICTION	10047139	8
VENA CAVA THROMBOSIS	10047195	8
VERTEBRAL ARTERY STENOSIS	10047330	8
VITAMIN B6 NORMAL	10047619	8
VITREOUS HAEMORRHAGE	10047655	8
TUMOUR MARKER INCREASED	10048621	8
FEAR OF FALLING	10048744	8
MYOFASCIAL PAIN SYNDROME	10048780	8
BREAST CALCIFICATIONS	10048782	8
CHOLESTASIS OF PREGNANCY	10049055	8
VASCULAR OPERATION	10049071	8
MILLER FISHER SYNDROME	10049567	8
LUNG CANCER METASTATIC	10050017	8
CERUMEN IMPACTION	10050337	8
PARENTERAL NUTRITION	10051284	8
STERNOTOMY	10051501	8
OSCILLOPSIA	10052087	8

ACTIVATED PROTEIN C RESISTANCE TEST	10067642	8
DISEASE COMPLICATION	10067671	8
ANTIBIOTIC THERAPY	10067768	8
PRODUCT ODOUR ABNORMAL	10069226	8
PRODUCT CONTAINER ISSUE	10069293	8
MAY-THURNER SYNDROME	10069727	8
MYCOBACTERIUM TUBERCULOSIS COMPLEX TEST POSITIVE	10070325	8
MYCOBACTERIUM TEST	10070407	8
CANDIDA TEST NEGATIVE	10070452	8
DISTRIBUTIVE SHOCK	10070559	8
BODY FLUID ANALYSIS	10070900	8
PROCEDURAL HAEMORRHAGE	10071229	8
POST-TRAUMATIC NECK SYNDROME	10071366	8
THYROID STIMULATING IMMUNOGLOBULIN INCREASED	10071389	8
RESTING TREMOR	10071390	8
UNDIFFERENTIATED CONNECTIVE TISSUE DISEASE	10071575	8
TYPICAL AURA WITHOUT HEADACHE	10071669	8
GIARDIA TEST	10072433	8
INJECTION SITE PLAQUE	10073174	8
ASPERGILLUS INFECTION	10074171	8
NEUROMUSCULAR PAIN	10074313	8
VEIN COLLAPSE	10074621	8
SUPINE POSITION	10074742	8
METHYLENETETRAHYDROFOLATE REDUCTASE GENE MUTATIO	10074753	8
BURN ORAL CAVITY	10075532	8
RESPIROVIRUS TEST	10075548	8
FACTOR V LEIDEN CARRIER	10075652	8
THYROID HORMONES DECREASED	10075803	8
VACCINATION SITE MACULE	10076181	8
ALLERGIC REACTION TO EXCIPIENT	10078853	8
AUTOIMMUNE THYROID DISORDER	10079165	8
RED EAR SYNDROME	10079225	8
JOINT SPACE NARROWING	10079942	8
DRUG SPECIFIC ANTIBODY	10080179	8
TRICHOGLOSSIA	10080276	8
SMALL AIRWAYS DISEASE	10080547	8
PULMONARY IMAGING PROCEDURE	10082579	8
PERIPHERAL VEIN OCCLUSION	10083103	8
SKIN DISCHARGE	10083144	8
SARS-COV-1 TEST NEGATIVE	10084445	8
INJECTION SITE MUSCLE ATROPHY	10084809	8
TROPONIN DECREASED	10084968	8
THORACIC OPERATION	10052703	8

PSYCHIATRIC EVALUATION	10053336	8
RED CELL DISTRIBUTION WIDTH DECREASED	10053921	8
COMPUTERISED TOMOGRAM KIDNEY	10054004	8
EXPLORATORY OPERATION	10056589	8
GASTRIC INFECTION	10056663	8
PALATAL OEDEMA	10056998	8
DEPENDENCE ON RESPIRATOR	10057482	8
EAR IRRIGATION	10058074	8
BIOPSY ARTERY ABNORMAL	10058364	8
DERMATOLOGIC EXAMINATION ABNORMAL	10058380	8
HYPOPERFUSION	10058558	8
GENERAL PHYSICAL CONDITION	10058912	8
METANEPHRINE URINE	10059217	8
FUNGAL TEST POSITIVE	10059423	8
ANTICONVULSANT DRUG LEVEL	10059466	8
CYTOLOGY ABNORMAL	10059523	8
RETINOGRAM	10059661	8
T-LYMPHOCYTE COUNT	10059741	8
RUSSELL'S VIPER VENOM TIME NORMAL	10059758	8
RED BLOOD CELL MORPHOLOGY	10059907	8
BASE EXCESS INCREASED	10059993	8
BASE EXCESS DECREASED	10059994	8
BLOOD ALCOHOL INCREASED	10060013	8
RIGHT VENTRICULAR SYSTOLIC PRESSURE INCREASED	10060236	8
TINEA INFECTION	10060889	8
ANALGESIC DRUG LEVEL THERAPEUTIC	10060943	8
BILIARY TRACT DISORDER	10061008	8
COOMBS TEST POSITIVE	10061090	8
LIGAMENT DISORDER	10061222	8
LIGAMENT INJURY	10061223	8
PROTEUS INFECTION	10061470	8
SCAN MYOCARDIAL PERFUSION NORMAL	10061514	8
MITRAL VALVE DISEASE	10061532	8
CARDIAC FIBRILLATION	10061592	8
ABDOMEN SCAN NORMAL	10061937	8
FRACTURE TREATMENT	10061959	8
GRIMACING	10061991	8
HLA MARKER STUDY	10062006	8
RETINAL OPERATION	10062107	8
SYPHILIS	10062120	8
VIRAL LOAD	10062178	8
SKIN GRAFT	10062247	8
PARANASAL CYST	10062321	8

HEPARIN-INDUCED THROMBOCYTOPENIA	10062506	8
RED BLOOD CELL ANALYSIS	10062748	8
BLOOD ALCOHOL NORMAL	10062897	8
BRAIN STEM SYNDROME	10063292	8
BRACHIOCEPHALIC VEIN THROMBOSIS	10063363	8
FOETAL CHROMOSOME ABNORMALITY	10064041	8
CSF IMMUNOGLOBULIN INCREASED	10064338	8
DEVICE DISLOCATION	10064684	8
MYOCARDIAL OEDEMA	10064966	8
FACTOR II MUTATION	10065003	8
THERAPY CESSATION	10065154	8
POLYCHONDRITIS	10065159	8
NIH STROKE SCALE SCORE INCREASED	10065528	8
VACCINATION SITE PUSTULE	10066047	8
EMERGENCY CARE	10066400	8
SARS-COV-2 RNA UNDETECTABLE	10085494	8
ACUTE MACULAR NEURORETINOPATHY	10086444	8
ABORTION	10000210	7
AGORAPHOBIA	10001502	7
AKATHISIA	10001540	7
ANTIMITOCHONDRIAL ANTIBODY	10002795	7
APALLIC SYNDROME	10002941	7
ARTERIOVENOUS FISTULA	10003226	7
ARTHROSCOPY	10003411	7
BENIGN NEOPLASM OF THYROID GLAND	10004412	7
BIOPSY VAGINA	10004900	7
BLOOD CALCIUM ABNORMAL	10005393	7
BLOOD ETHANOL	10005513	7
BLOOD IMMUNOGLOBULIN M DECREASED	10005600	7
BLOOD SODIUM ABNORMAL	10005800	7
BLOOD UREA ABNORMAL	10005846	7
BONE CYST	10005952	7
BRADYCARDIA FOETAL	10006094	7
BREAST CANCER RECURRENT	10006198	7
BREAST HAEMORRHAGE	10006254	7
BREAST HYPERPLASIA	10006256	7
BURN OESOPHAGEAL	10006707	7
CARCINOEMBRYONIC ANTIGEN	10007262	7
CAROTID ARTERY ANEURYSM	10007686	7
CARPAL TUNNEL DECOMPRESSION	10007695	7
CERVICAL DISCHARGE	10008260	7
CERVIX DISORDER	10008351	7
CHOLESTASIS	10008635	7

CLAVICLE FRACTURE	10009245	7
COAGULATION FACTOR VIII LEVEL	10009766	7
COAGULATION TIME PROLONGED	10009799	7
COLD TYPE HAEMOLYTIC ANAEMIA	10009868	7
COLLAGEN DISORDER	10009903	7
COLPOSCOPY	10010069	7
CONDUCTIVE DEAFNESS	10010280	7
CONJUNCTIVITIS ALLERGIC	10010744	7
CONJUNCTIVITIS VIRAL	10010755	7
COOMBS DIRECT TEST POSITIVE	10010933	7
CREUTZFELDT-JAKOB DISEASE	10011384	7
DEPRESSION SUICIDAL	10012397	7
DIFFUSE LARGE B-CELL LYMPHOMA	10012818	7
DROP ATTACKS	10013643	7
DUODENAL ULCER	10013836	7
DYSMETRIA	10013936	7
ECZEMA EYELIDS	10014194	7
ELECTRONYSTAGMOGRAM ABNORMAL	10014450	7
EMPYEMA	10014568	7
EPIGLOTTITIS	10015030	7
EXERCISE LACK OF	10015650	7
FACE AND MOUTH X-RAY NORMAL	10016020	7
FUNDOSCOPY	10017519	7
GLIOSIS	10018341	7
GRANULOCYTES ABNORMAL	10018685	7
HEPATIC NEOPLASM	10019695	7
HEPATITIS B ANTIBODY	10019732	7
HEPATITIS B ANTIBODY NEGATIVE	10019734	7
HORMONE REPLACEMENT THERAPY	10020388	7
HYDROURETER	10020533	7
HYPERCHOLESTEROLAEMIA	10020603	7
HYPEROSMOLAR STATE	10020697	7
HYPERTROPHIC CARDIOMYOPATHY	10020871	7
HYPOCOMPLEMENTAEMIA	10020974	7
HYPOMANIA	10021030	7
INFLAMMATION SCAN	10021966	7
INJECTION SITE ATROPHY	10022048	7
INJECTION SITE DERMATITIS	10022056	7
INTERNATIONAL NORMALISED RATIO ABNORMAL	10022592	7
LIVER TRANSPLANT	10024714	7
MENINGISM	10027197	7
METASTASES TO LUNG	10027458	7
MONOCLONAL ANTIBODY IMMUNOCONJUGATE THERAPY	10027859	7

MONOCYTOSIS	10027906	7
MUMPS	10028257	7
MUTISM	10028403	7
NASAL ULCER	10028780	7
NEUROLOGIC NEGLECT SYNDROME	10029289	7
NODAL RHYTHM	10029470	7
OEDEMA MUCOSAL	10030111	7
OPISTHOTONUS	10030899	7
OVARIAN FAILURE	10033165	7
PALINDROMIC RHEUMATISM	10033534	7
PEAK EXPIRATORY FLOW RATE	10034192	7
PEAK EXPIRATORY FLOW RATE DECREASED	10034195	7
PICA	10035001	7
PORTAL HYPERTENSION	10036200	7
PRIMIGRAVIDA	10036757	7
PSEUDOLYMPHOMA	10037127	7
PULMONARY SARCOIDOSIS	10037430	7
PULPITIS DENTAL	10037464	7
RENAL SCAN	10038520	7
RETINAL VASCULITIS	10038905	7
RUBELLA ANTIBODY TEST	10039259	7
SCHIZOPHRENIA	10039626	7
SMOOTH MUSCLE ANTIBODY POSITIVE	10041229	7
SPLENIC ARTERY ANEURYSM	10041645	7
STRESS FRACTURE	10042212	7
SWEATING FEVER	10042666	7
TOE AMPUTATION	10043913	7
TOOTH DISCOLOURATION	10044032	7
TOTAL LUNG CAPACITY	10044097	7
TRANSFERRIN	10044354	7
VISUAL ACUITY TESTS NORMAL	10047537	7
VITAMIN C	10047620	7
VULVAL ABSCESS	10047732	7
UMBILICAL CORD ABNORMALITY	10048596	7
ANKLE OPERATION	10049012	7
LABILE HYPERTENSION	10049079	7
RECTAL DISCHARGE	10049101	7
PIRIFORMIS SYNDROME	10049433	7
BRAIN STEM AUDITORY EVOKED RESPONSE	10049507	7
PRECANCEROUS CELLS PRESENT	10049673	7
GALLBLADDER POLYP	10049704	7
BLOOD HOMOCYSTEINE INCREASED	10049733	7
APPENDICEAL ABSCESS	10049764	7

ROTATOR CUFF REPAIR	10050324	7
AORTIC VALVE CALCIFICATION	10050559	7
LEFT VENTRICULAR ENLARGEMENT	10050581	7
TRANSIENT ACANTHOLYTIC DERMATOSIS	10051446	7
VASCULAR CALCIFICATION	10051753	7
INFUSION RELATED REACTION	10051792	7
BETA HAEMOLYTIC STREPTOCOCCAL INFECTION	10052100	7
GASTROINTESTINAL HYPOMOTILITY	10052105	7
FLUORESCENT IN SITU HYBRIDISATION	10066931	7
CYTOGENETIC ABNORMALITY	10067477	7
PHARYNGEAL ABSCESS	10067781	7
PLEURAL FLUID ANALYSIS	10068055	7
VENOUS OXYGEN SATURATION DECREASED	10068427	7
CAPSULAR CONTRACTURE ASSOCIATED WITH BREAST IMPLAN	10068514	7
DRAIN REMOVAL	10068616	7
INJECTION SITE EXFOLIATION	10068689	7
LYSOZYME	10068708	7
INCORRECT DOSE ADMINISTERED BY DEVICE	10068945	7
VACCINATION SITE EXFOLIATION	10069489	7
EXPOSURE TO VACCINATED PERSON	10069603	7
BILE ACID MALABSORPTION	10069703	7
CHLAMYDIA TEST POSITIVE	10070159	7
VIRAL TITRE INCREASED	10070256	7
TYPHUS RICKETTSIA TEST	10070404	7
FALSE POSITIVE INVESTIGATION RESULT	10070748	7
FOETAL MONITORING NORMAL	10071506	7
TILT TABLE TEST NORMAL	10072466	7
MICROVASCULAR CORONARY ARTERY DISEASE	10072685	7
BURSAL FLUID ACCUMULATION	10072833	7
PERINEAL INJURY	10073329	7
GENITAL HERPES SIMPLEX	10073931	7
OPHTHALMIC HERPES SIMPLEX	10073938	7
DIAPHRAGMATIC SPASM	10074312	7
HYSTEROSCOPY ABNORMAL	10074341	7
POOR FEEDING INFANT	10075316	7
RIGHT VENTRICULAR EJECTION FRACTION DECREASED	10075337	7
HEARING AID THERAPY	10075385	7
EYE SYMPTOM	10075536	7
GENITOURINARY SYMPTOM	10075537	7
AORTIC VALVE THICKENING	10075851	7
VACCINATION SITE JOINT SWELLING	10076178	7
PRODUCT USE IN UNAPPROVED INDICATION	10076476	7
BREATH SOUNDS NORMAL	10076579	7

PERFORATION	10076705	7
LUNG HYPOINFLATION	10076905	7
INFECTED BITE	10076911	7
DEVICE USE ISSUE	10077812	7
VENOUS ANGIOPLASTY	10077826	7
INSURANCE ISSUE	10078156	7
SYSTEMIC SCLERODERMA	10078638	7
VENTRICULAR ENLARGEMENT	10079339	7
PERFUSION BRAIN SCAN ABNORMAL	10079703	7
CONTINUOUS GLUCOSE MONITORING	10079764	7
EYE HAEMATOMA	10079891	7
ULTRASOUND PANCREAS	10080632	7
SKIN LESION INFLAMMATION	10081154	7
STIFF TONGUE	10081491	7
SPINAL RETROLISTHESIS	10081602	7
HEART RATE VARIABILITY TEST	10082112	7
ANTI-RNA POLYMERASE III ANTIBODY	10082281	7
INVESTIGATION NORMAL	10082416	7
REFUSAL OF VACCINATION	10082811	7
PERIORBITAL DISCOMFORT	10082879	7
EYE INFARCTION	10083006	7
RESTLESS ARM SYNDROME	10083534	7
PRECANCEROUS CONDITION	10083553	7
ANAL RASH	10084123	7
BLOOD DONATION	10084199	7
BILIARY OBSTRUCTION	10084247	7
DISCOURAGEMENT	10084257	7
COVID-19 TREATMENT	10084460	7
TRANSVERSE SINUS STENOSIS	10084532	7
PATIENT ELOPEMENT	10084569	7
TELEMEDICINE	10084643	7
CIRCUMORAL OEDEMA	10052250	7
MIGRAINE WITHOUT AURA	10052787	7
INGROWN HAIR	10052899	7
OESOPHAGRAM	10053059	7
MICROBIOLOGY TEST	10053075	7
NON-ALCOHOLIC STEATOHEPATITIS	10053219	7
INSULIN-REQUIRING TYPE 2 DIABETES MELLITUS	10053247	7
EXPOSURE TO TOXIC AGENT	10053487	7
NEURODEGENERATIVE DISORDER	10053643	7
CARDIOVASCULAR AUTONOMIC FUNCTION TEST	10053689	7
HISTONE ANTIBODY POSITIVE	10053833	7
VITAMIN SUPPLEMENTATION	10054128	7

CARDIAC INFECTION	10054212	7
FIBROMUSCULAR DYSPLASIA	10054794	7
VERTEBRAL OSTEOPHYTE	10055042	7
COLON CANCER METASTATIC	10055114	7
BURSA INJURY	10057602	7
ULTRASOUND BILIARY TRACT NORMAL	10057850	7
EAR TUBE INSERTION	10057900	7
SUBACUTE CUTANEOUS LUPUS ERYTHEMATOSUS	10057903	7
INFECTED CYST	10058015	7
AORTIC OCCLUSION	10058178	7
PULMONARY HILUM MASS	10058563	7
VITAMIN E	10058765	7
MYCOBACTERIUM AVIUM COMPLEX INFECTION	10058806	7
INTESTINAL MASS	10059017	7
HAEMORRHAGIC CYST	10059189	7
BAND NEUTROPHIL COUNT	10059477	7
SALIVA ANALYSIS	10059532	7
BLOOD ALKALINE PHOSPHATASE DECREASED	10059569	7
RETINOGRAM ABNORMAL	10059663	7
ANTI-GAD ANTIBODY POSITIVE	10059728	7
ADHESION	10059837	7
ALPHA TUMOUR NECROSIS FACTOR	10059981	7
HAEMATOMA EVACUATION	10060733	7
ISCHAEMIC CEREBRAL INFARCTION	10060840	7
SINUSITIS BACTERIAL	10060841	7
MONOCLONAL GAMMOPATHY	10060880	7
ARTERIAL DISORDER	10060963	7
ARTHRITIS INFECTIVE	10060968	7
FOOT OPERATION	10061160	7
IMMUNOLOGY TEST ABNORMAL	10061214	7
GASTROINTESTINAL ULCER	10061459	7
LACRIMAL DISORDER	10061525	7
ABDOMINAL OPERATION	10061609	7
CHRONIC RESPIRATORY DISEASE	10061768	7
CSF VIRUS NO ORGANISMS OBSERVED	10061801	7
PANCREATIC ENZYMES	10061898	7
MONONEUROPATHY	10062203	7
SOCIAL PROBLEM	10062254	7
JOINT HYPEREXTENSION	10062310	7
BRAIN STEM THROMBOSIS	10062573	7
CYCLIC VOMITING SYNDROME	10062937	7
BRONCHOALVEOLAR LAVAGE ABNORMAL	10063078	7
OPIATES NEGATIVE	10063225	7

INTERLEUKIN LEVEL INCREASED	10063322	7
TRYPTASE INCREASED	10063342	7
AMNIOTIC FLUID VOLUME DECREASED	10063356	7
SINGLE FUNCTIONAL KIDNEY	10063530	7
AUTISM SPECTRUM DISORDER	10063844	7
FALLOPIAN TUBE OPERATION	10064510	7
HUMAN PAPILLOMA VIRUS TEST NEGATIVE	10064543	7
BREATH SOUNDS	10064779	7
PANCREATIC DUCT DILATATION	10064858	7
EX-TOBACCO USER	10065386	7
NEUROPSYCHOLOGICAL TEST	10065432	7
GASTROINTESTINAL STOMA COMPLICATION	10065718	7
SOFT TISSUE NECROSIS	10065769	7
THERAPY INTERRUPTED	10066377	7
LATENT AUTOIMMUNE DIABETES IN ADULTS	10066389	7
SCAR INFLAMMATION	10085373	7
CYTOKINE INCREASED	10085573	7
THROMBOSIS WITH THROMBOCYTOPENIA SYNDROME	10086158	7
MYOCARDIAL STRAIN IMAGING ABNORMAL	10086295	7
NEISSERIA TEST NEGATIVE	10086416	7
BREAKTHROUGH COVID-19	10086861	7
ABNORMAL MENSTRUAL CLOTS	10087591	7
ABDOMINAL ADHESIONS	10000050	6
ADDISON'S DISEASE	10001130	6
ADRENAL HAEMORRHAGE	10001361	6
ALBUMIN GLOBULIN RATIO INCREASED	10001567	6
ALCOHOL ABUSE	10001584	6
ALCOHOL POISONING	10001605	6
AMAUROSIS FUGAX	10001903	6
ANAEMIA OF CHRONIC DISEASE	10002073	6
ANISOCORIA	10002535	6
ANTIMICROBIAL SUSCEPTIBILITY TEST SENSITIVE	10002794	6
APPLICATION SITE ERYTHEMA	10003041	6
BIOPSY BONE ABNORMAL	10004736	6
BIOPSY PERIPHERAL NERVE	10004845	6
BLADDER CANCER	10005003	6
BLOOD CHOLESTEROL ABNORMAL	10005423	6
BLOOD ETHANOL INCREASED	10005515	6
BLOOD MAGNESIUM ABNORMAL	10005652	6
BLOOD TESTOSTERONE DECREASED	10005814	6
BLOOD ZINC NORMAL	10005869	6
BONE CANCER	10005949	6
BRAIN COMPRESSION	10006112	6

BRAIN HYPOXIA	10006127	6
BREAST ATROPHY	10006179	6
BREAST RECONSTRUCTION	10006305	6
CAPILLARY LEAK SYNDROME	10007196	6
CAROTID ENDARTERECTOMY	10007692	6
CAUDA EQUINA SYNDROME	10007821	6
CD4 LYMPHOCYTES	10007838	6
CD4/CD8 RATIO	10007842	6
CHLOASMA	10008570	6
CHOLANGIOGRAM	10008598	6
CHORIORETINITIS	10008769	6
COAGULATION FACTOR V LEVEL NORMAL	10009758	6
COCCIDIOIDOMYCOSIS	10009825	6
COLOUR BLINDNESS	10010050	6
CORNEAL GRAFT REJECTION	10011017	6
CREATININE RENAL CLEARANCE INCREASED	10011364	6
CRYOGLOBULINS	10011476	6
CRYOGLOBULINS ABSENT	10011477	6
CRYOTHERAPY	10011483	6
CYST DRAINAGE	10011737	6
DANDRUFF	10011859	6
DEVICE BREAKAGE	10012575	6
DYSPAREUNIA	10013941	6
DYSPHORIA	10013954	6
ECLAMPSIA	10014129	6
EDUCATIONAL PROBLEM	10014270	6
ELECTROCARDIOGRAM ST SEGMENT	10014389	6
ELECTROCARDIOGRAM ST SEGMENT NORMAL	10014393	6
ELECTROCARDIOGRAM T WAVE AMPLITUDE DECREASED	10014394	6
ENTEROCOCCAL BACTERAEMIA	10014885	6
ERYSIPELAS	10015145	6
EYE INFECTION VIRAL	10015940	6
FIBRIN D DIMER DECREASED	10016579	6
FISTULA	10016717	6
FROSTBITE	10017382	6
GALACTORRHOEA	10017600	6
GASTROENTERITIS ESCHERICHIA COLI	10017903	6
HAEMARTHROSIS	10018829	6
HAEMOGLOBIN URINE PRESENT	10018909	6
HAPTOGLOBIN INCREASED	10019152	6
HEAD BANGING	10019191	6
HEART DISEASE CONGENITAL	10019273	6
HOMELESS	10020361	6

HORNER'S SYNDROME	10020392	6
HYPEREMESIS GRAVIDARUM	10020614	6
HYPERMOBILITY SYNDROME	10020677	6
HYPOGAMMAGLOBULINAEMIA	10020983	6
HYPOVENTILATION	10021133	6
ILLITERACY	10021401	6
INFECTION PARASITIC	10021857	6
INFLAMMATORY CARCINOMA OF THE BREAST	10021980	6
INTENTION TREMOR	10022520	6
INTERTRIGO	10022622	6
JAW FRACTURE	10023149	6
KAWASAKI'S DISEASE	10023320	6
KETONURIA	10023388	6
LIBIDO INCREASED	10024421	6
LIVER SCAN	10024707	6
LUNG ABSCESS	10025028	6
LYMPHOCYTE COUNT ABNORMAL	10025252	6
MEAN ARTERIAL PRESSURE	10026982	6
MESENTERIC ARTERY THROMBOSIS	10027397	6
MICROALBUMINURIA	10027525	6
MOTOR NEURONE DISEASE	10028003	6
MUCOSAL ULCERATION	10028124	6
MULTIGRAVIDA	10028160	6
NASAL CAVITY PACKING	10028733	6
NERVE STIMULATION TEST ABNORMAL	10029192	6
NEURODERMATITIS	10029263	6
NEUROGENIC BLADDER	10029279	6
NEUROMA	10029308	6
NIPPLE EXUDATE BLOODY	10029418	6
NODAL ARRHYTHMIA	10029458	6
OCULAR HYPERTENSION	10030043	6
PALMAR-PLANTAR ERYTHRODYSAESTHESIA SYNDROME	10033553	6
PANCREATIC ATROPHY	10033603	6
PARONYCHIA	10034016	6
PERICARDITIS CONSTRICTIVE	10034487	6
PERIPHERAL NERVE INJURY	10034586	6
PHOBIA	10034912	6
POIKILOCYTOSIS	10035774	6
POLYCYTHAEMIA VERA	10036057	6
POLYMORPHIC LIGHT ERUPTION	10036087	6
PROCEDURAL SITE REACTION	10036769	6
PROCTITIS ULCERATIVE	10036783	6
PROTHROMBIN LEVEL INCREASED	10037051	6

PROTRUSION TONGUE	10037076	6
PYELONEPHRITIS ACUTE	10037597	6
RENAL CANCER	10038389	6
RENAL COLIC	10038419	6
RENAL HAEMORRHAGE	10038460	6
RETAINED PLACENTA OR MEMBRANES	10038758	6
RETICULOCYTE COUNT NORMAL	10038794	6
RETINAL FUNCTION TEST ABNORMAL	10038866	6
RHESUS ANTIBODIES	10039033	6
ROCKY MOUNTAIN SPOTTED FEVER	10039207	6
SCIATIC NERVE INJURY	10039670	6
SEBORRHOEIC KERATOSIS	10039796	6
SEROMA	10040102	6
SEROTONIN SYNDROME	10040108	6
SEXUAL DYSFUNCTION	10040477	6
SIGMOIDOSCOPY ABNORMAL	10040670	6
SKULL X-RAY ABNORMAL	10040965	6
SPINAL CORD INJURY THORACIC	10041558	6
SPLENIC HAEMORRHAGE	10041647	6
STASIS DERMATITIS	10041955	6
STATUS ASTHMATICUS	10041961	6
THORACOTOMY	10043491	6
THYROID NEOPLASM	10043744	6
TRANSFERRIN NORMAL	10044358	6
TUBERCULIN TEST NEGATIVE	10044727	6
ULNA FRACTURE	10045375	6
ULTRASOUND EYE ABNORMAL	10045417	6
URGE INCONTINENCE	10046494	6
URINARY BLADDER HAEMORRHAGE	10046528	6
URTICARIA CHOLINERGIC	10046740	6
URTICARIA PAPULAR	10046750	6
UTERINE HYPERTONUS	10046790	6
VAGINAL CYST	10046900	6
VERY LOW DENSITY LIPOPROTEIN NORMAL	10047362	6
VESTIBULAR FUNCTION TEST ABNORMAL	10047390	6
VIOLENCE-RELATED SYMPTOM	10047426	6
VITAMIN B12 ABSORPTION TEST	10047605	6
VOLUME BLOOD DECREASED	10047689	6
VON WILLEBRAND'S DISEASE	10047715	6
VULVOVAGINAL CANDIDIASIS	10047784	6
WEIGHT GAIN POOR	10047897	6
WISDOM TEETH REMOVAL	10047991	6
RESTRICTIVE PULMONARY DISEASE	10048667	6

SCIATIC NERVE NEUROPATHY	10048950	6
ARACHNOID CYST	10049005	6
HAEMATOCRIT ABNORMAL	10049221	6
VOCAL CORD PARESIS	10049234	6
NOCTURNAL DYSPNOEA	10049235	6
RIGHT VENTRICULAR HYPERTROPHY	10050326	6
SCLERAL HAEMORRHAGE	10050508	6
COLD AGGLUTININS	10050523	6
FOETAL HEART RATE DECREASED	10051136	6
BILE DUCT STENT INSERTION	10051773	6
LYMPHOID TISSUE HYPERPLASIA	10051808	6
UVULITIS	10051962	6
OVARIAN OPERATION	10052089	6
IODINE ALLERGY	10052098	6
ESCHERICHIA URINARY TRACT INFECTION	10052238	6
BETA-2 GLYCOPROTEIN ANTIBODY POSITIVE	10066404	6
STAPHYLOCOCCAL SKIN INFECTION	10066409	6
OROPHARYNGEAL PLAQUE	10067721	6
HLA MARKER STUDY POSITIVE	10067937	6
INTELLECTUAL DISABILITY	10067989	6
PARASITIC TEST	10068096	6
HYPERDYNAMIC LEFT VENTRICLE	10068359	6
ANTIVIRAL TREATMENT	10068724	6
LUNG CYST	10068749	6
RESPIRATORY TRACT INFLAMMATION	10068956	6
PRENATAL SCREENING TEST ABNORMAL	10069151	6
ELECTROCAUTERISATION	10069204	6
CSF WHITE BLOOD CELL DIFFERENTIAL	10069602	6
PRODUCT COMPLAINT	10069902	6
HAEMOPHILUS TEST POSITIVE	10070100	6
EXECUTIVE DYSFUNCTION	10070246	6
ADENOVIRUS TEST POSITIVE	10070369	6
TREPONEMA TEST FALSE POSITIVE	10070402	6
CSF IMMUNOGLOBULIN	10070420	6
HEAVY EXPOSURE TO ULTRAVIOLET LIGHT	10070687	6
RESPIRATORY MUSCLE WEAKNESS	10070833	6
WALKING DISTANCE TEST NORMAL	10071031	6
CHEST TUBE REMOVAL	10071381	6
EXPOSURE VIA DIRECT CONTACT	10071411	6
ANTI-MUSCLE SPECIFIC KINASE ANTIBODY	10071475	6
INFECTIOUS PLEURAL EFFUSION	10071699	6
MUSCLE OEDEMA	10071759	6
AUTOINFLAMMATORY DISEASE	10072220	6

OESOPHAGEAL MOTILITY DISORDER	10072419	6
PERIPHERAL ARTERY BYPASS	10072561	6
LID SULCUS DEEPENED	10072716	6
REVERSIBLE CEREBRAL VASOCONSTRICTION SYNDROME	10073240	6
ESCHERICHIA TEST NEGATIVE	10073291	6
EYE ULCER	10073423	6
INCORRECT DOSAGE ADMINISTERED	10073768	6
LARGE INTESTINAL STENOSIS	10074061	6
JOINT RANGE OF MOTION MEASUREMENT	10075631	6
AUTOIMMUNE ENCEPHALOPATHY	10075691	6
LISTERIA TEST	10075707	6
INVASIVE BREAST CARCINOMA	10075713	6
ADMINISTRATION SITE WARMTH	10075971	6
INTERVENTIONAL PROCEDURE	10075978	6
FEEDING INTOLERANCE	10076042	6
PULMONARY PHYSICAL EXAMINATION	10076199	6
CHRONIC DISEASE	10076311	6
NASAL CRUSTING	10076524	6
PRODUCT SUBSTITUTION	10076753	6
PROCTECTOMY	10077252	6
MITE ALLERGY	10077290	6
COMPUTERISED TOMOGRAM LIVER ABNORMAL	10078360	6
NEUROPSYCHIATRIC SYMPTOMS	10078497	6
ULTRASOUND PANCREAS NORMAL	10078674	6
OROPHARYNGEAL OEDEMA	10078783	6
HYPERINTENSITY IN BRAIN DEEP NUCLEI	10079392	6
HAEMOPHILUS TEST	10080946	6
C1 ESTERASE INHIBITOR TEST NORMAL	10080954	6
PROCEDURAL FAILURE	10081594	6
DYSBIOSIS	10082129	6
NASOLARYNGOSCOPY	10082566	6
AST/ALT RATIO ABNORMAL	10082832	6
EXPOSURE TO FUNGUS	10083061	6
TRIPLE POSITIVE BREAST CANCER	10083233	6
TRANSCUTANEOUS PACING	10083519	6
NON-PITTING OEDEMA	10083903	6
TRACHEAL ASPIRATE CULTURE	10084653	6
CAFFEINE CONSUMPTION	10052308	6
VENTRICULAR ASSIST DEVICE INSERTION	10052371	6
CATHETER REMOVAL	10052916	6
CHOLELITHOTOMY	10052925	6
CRANIECTOMY	10052937	6
JOINT DISLOCATION REDUCTION	10052988	6

INTESTINAL ADHESION LYSIS	10052993	6
IRON BINDING CAPACITY UNSATURATED	10053226	6
CARDIAC PACEMAKER EVALUATION	10053441	6
ABSCESS JAW	10053573	6
ABSCESS NECK	10053576	6
EXTRAOCULAR MUSCLE DISORDER	10053635	6
THERAPEUTIC PROCEDURE	10053757	6
ALLERGIC COUGH	10053779	6
CSF MONOCYTE COUNT DECREASED	10053800	6
EVANS SYNDROME	10053873	6
DACRYOSTENOSIS ACQUIRED	10053990	6
LIPOPROTEIN (A)	10054020	6
PERIPHERAL PARALYSIS	10054808	6
SPLENIC LESION	10054829	6
ALLERGY TO PLANTS	10054928	6
BRAIN CANCER METASTATIC	10055093	6
OLFACTORY NERVE DISORDER	10056388	6
STAPHYLOCOCCAL SEPSIS	10056430	6
DENTAL EXAMINATION ABNORMAL	10056828	6
PALPABLE PURPURA	10056872	6
INTESTINAL ANASTOMOSIS	10057146	6
BLOOD METHAEMOGLOBIN	10057157	6
OESOPHAGEAL DILATION PROCEDURE	10057305	6
EAR INJURY	10057446	6
ANGIOGRAM PERIPHERAL ABNORMAL	10057517	6
ULTRASOUND BILIARY TRACT ABNORMAL	10057851	6
CYSTOID MACULAR OEDEMA	10058202	6
BIOPSY INTESTINE ABNORMAL	10058371	6
BIOPSY UTERUS ABNORMAL	10058376	6
SPINAL CORD INFARCTION	10058571	6
PSEUDOMONAL BACTERAEMIA	10058923	6
LABOUR PAIN	10059204	6
EXTREMITY NECROSIS	10059385	6
THERAPEUTIC HYPOTHERMIA	10059485	6
ABDOMINAL CAVITY DRAINAGE	10059486	6
HAEMORRHOID OPERATION	10059624	6
X-RAY WITH CONTRAST LOWER GASTROINTESTINAL TRACT	10059691	6
B-LYMPHOCYTE COUNT	10059966	6
URINE AMPHETAMINE NEGATIVE	10059986	6
RHEUMATOID FACTOR QUANTITATIVE	10060198	6
PLATELET MORPHOLOGY NORMAL	10060221	6
TYPE V HYPERLIPIDAEMIA	10060755	6
JOINT STABILISATION	10060788	6

EMBOLIC CEREBRAL INFARCTION	10060839	6
BENIGN LYMPH NODE NEOPLASM	10060997	6
BINOCULAR EYE MOVEMENT DISORDER	10061010	6
CARDIOACTIVE DRUG LEVEL	10061028	6
THYROGLOBULIN	10061136	6
NEUTROPHIL COUNT ABNORMAL	10061313	6
OPTIC NEUROPATHY	10061323	6
SKELETAL INJURY	10061363	6
COLORECTAL CANCER	10061451	6
SALIVARY GLAND NEOPLASM	10061497	6
SCLERAL DISORDER	10061510	6
X-RAY GASTROINTESTINAL TRACT ABNORMAL	10061582	6
X-RAY GASTROINTESTINAL TRACT NORMAL	10061584	6
BLAST CELLS	10061700	6
BRONCHITIS BACTERIAL	10061736	6
COLON OPERATION	10061779	6
CONJUNCTIVITIS BACTERIAL	10061784	6
EAR INFECTION VIRAL	10061830	6
RETINAL VASCULAR THROMBOSIS	10062108	6
VITAMIN D INCREASED	10062134	6
VITAMIN B12 ABNORMAL	10062187	6
SPINAL CORD NEOPLASM	10062261	6
NAIL RIDGING	10062283	6
HERPES DERMATITIS	10062639	6
HAEMORRHAGIC DIATHESIS	10062713	6
MESENTERIC PANNICULITIS	10063031	6
SPINAL CORD OEDEMA	10063036	6
HEPATITIS B SURFACE ANTIBODY	10063051	6
FAECAL DISIMPACTION	10063056	6
BARBITURATES NEGATIVE	10063224	6
HISTAMINE ABNORMAL	10063302	6
BLADDER HYPERTROPHY	10063408	6
HYPERGLYCAEMIC HYPEROSMOLAR NONKETOTIC SYNDROME	10063554	6
EXCESSIVE GRANULATION TISSUE	10063560	6
VERTEBROPLASTY	10063565	6
EXPOSURE TO EXTREME TEMPERATURE	10063601	6
BIOCHEMICAL PREGNANCY	10063639	6
BLOOD PRESSURE MANAGEMENT	10063926	6
VASOPRESSIVE THERAPY	10064148	6
CELL MARKER	10064496	6
ILIAC ARTERY OCCLUSION	10064601	6
NAIL GROWTH ABNORMAL	10064764	6
LIGAMENTITIS	10064804	6

CANCER IN REMISSION	10065305	6
BONE MARROW FAILURE	10065553	6
TRACHEAL INFLAMMATION	10065899	6
CARDIOVASCULAR INSUFFICIENCY	10065929	6
ULTRASOUND HEAD	10085915	6
BRIEF RESOLVED UNEXPLAINED EVENT	10086181	6
SICK LEAVE	10086390	6
ANGIOTENSIN CONVERTING ENZYME NORMAL	10087039	6
ABSCESS INTESTINAL	10000285	5
ACOUSTIC NEUROMA	10000523	5
ACROCHORDON	10000591	5
ADENOMA BENIGN	10001233	5
ALBUMIN URINE ABSENT	10001581	5
ALBUMIN URINE PRESENT	10001582	5
ALCOHOLISM	10001639	5
ALPHA 1 FOETOPROTEIN NORMAL	10001782	5
ANHEDONIA	10002511	5
AORTITIS	10002921	5
ARRHYTHMIA SUPRAVENTRICULAR	10003130	5
ARTERIOVENOUS MALFORMATION	10003193	5
ASPIRATION BONE MARROW	10003505	5
BIOPSY BONE	10004735	5
BIOPSY STOMACH	10004879	5
BIOPSY STOMACH NORMAL	10004881	5
BIOPSY VULVA ABNORMAL	10004904	5
BLOOD BILIRUBIN UNCONJUGATED	10005369	5
BLOOD CATECHOLAMINES NORMAL	10005415	5
BLOOD CREATINE PHOSPHOKINASE ABNORMAL	10005468	5
BLOOD CREATININE ABNORMAL	10005481	5
BLOOD DONOR	10005494	5
BLOOD FOLATE DECREASED	10005527	5
BLOOD INSULIN	10005605	5
BLOOD LACTIC ACID ABNORMAL	10005633	5
BRACHIAL PLEXUS INJURY	10006074	5
BREAST CANCER STAGE II	10006200	5
BREAST CANCER STAGE III	10006201	5
CARDIAC OUTPUT	10007594	5
CARDIAC SARCOIDOSIS	10007604	5
CAVERNOUS SINUS THROMBOSIS	10007830	5
CD8 LYMPHOCYTES	10007845	5
CERVICAL CYST	10008254	5
CERVICAL POLYP	10008297	5
CHOLANGIOCARCINOMA	10008593	5

CHROMOSOMAL ANALYSIS	10008811	5
CHRONIC MYELOMONOCYTIC LEUKAEMIA	10009018	5
CORNEAL OPACITY	10011035	5
CULTURE TISSUE SPECIMEN	10011635	5
CYSTOGRAM	10011804	5
DERMATITIS EXFOLIATIVE GENERALISED	10012456	5
DEVICE EXPULSION	10012578	5
DEVICE LEAKAGE	10012587	5
DYSPRAXIA	10013976	5
ELECTROCARDIOGRAM QRS COMPLEX NORMAL	10014379	5
ELECTROCARDIOGRAM T WAVE PEAKED	10014396	5
ELECTROCONVULSIVE THERAPY	10014404	5
ELECTROLYTE DEPLETION	10014415	5
ELECTRONYSTAGMOGRAM NORMAL	10014451	5
ELLIPTOCYTOSIS	10014489	5
ENCEPHALITIS VIRAL	10014612	5
ENCEPHALOMYELITIS	10014619	5
ENDARTERECTOMY	10014648	5
ENTEROVIRUS INFECTION	10014909	5
ENTHESOPATHY	10014910	5
ERYTHEMA INDURATUM	10015213	5
ERYTHROPSIA	10015290	5
ESCHERICHIA SEPSIS	10015296	5
EXTERNAL FIXATION OF FRACTURE	10015741	5
EXTRAPYRAMIDAL DISORDER	10015832	5
EXTRAVASATION	10015866	5
FACE AND MOUTH X-RAY ABNORMAL	10016019	5
FASCIOTOMY	10016237	5
FAT TISSUE INCREASED	10016251	5
FEMALE ORGASMIC DISORDER	10016405	5
FIXED ERUPTION	10016741	5
FLIGHT OF IDEAS	10016777	5
FUNGAEMIA	10017523	5
GALLBLADDER OEDEMA	10017637	5
GASTRITIS EROSIVE	10017865	5
GASTROINTESTINAL NECROSIS	10017982	5
GINGIVAL RECESSION	10018290	5
GLIOBLASTOMA	10018336	5
GONORRHOEA	10018612	5
HEMIANOPIA	10019452	5
HEMIANOPIA HOMONYMOUS	10019456	5
HEPATITIS A ANTIBODY NEGATIVE	10019723	5
HEPATOCELLULAR INJURY	10019837	5

HEPATOSPLENOMEGALY	10019847	5
HIV INFECTION	10020161	5
HOSTILITY	10020400	5
HYPERAMMONAEMIA	10020575	5
HYPERMETABOLISM	10020674	5
HYPERPARATHYROIDISM	10020705	5
HYPERSENSITIVITY VASCULITIS	10020764	5
HYPERTENSIVE ENCEPHALOPATHY	10020803	5
HYSTEROTOMY	10021171	5
INJECTION SITE EROSION	10022059	5
IRIS ADHESIONS	10022945	5
IRON BINDING CAPACITY TOTAL INCREASED	10022965	5
LACRIMATION DECREASED	10023642	5
LARYNGEAL PAIN	10023848	5
LEUKOPLAKIA ORAL	10024396	5
LOCKED-IN SYNDROME	10024792	5
MASTOCYTOSIS	10026891	5
MENINGITIS BACTERIAL	10027202	5
MENINGITIS CHEMICAL	10027206	5
MITRAL VALVE REPLACEMENT	10027732	5
MOLE EXCISION	10027805	5
MOLLUSCUM CONTAGIOSUM	10027807	5
MONOCLONAL IMMUNOGLOBULIN PRESENT	10027864	5
MYELOSUPPRESSION	10028584	5
MYOKYMIA	10028632	5
MYOPERICARDITIS	10028650	5
NASAL OBSTRUCTION	10028748	5
NECROTISING FASCIITIS	10028885	5
NERVE STIMULATION TEST NORMAL	10029193	5
OBSESSIVE THOUGHTS	10029897	5
OROPHARYNGEAL SPASM	10031111	5
OSTEOMYELITIS ACUTE	10031253	5
PARAPSORIASIS	10033898	5
PARATRACHEAL LYMPHADENOPATHY	10033969	5
PELVIC CONGESTION	10034240	5
PERITONEAL DIALYSIS	10034660	5
PERSONALITY DISORDER	10034721	5
PLEURAL FIBROSIS	10035600	5
PLEURODESIS	10035625	5
POLYARTERITIS NODOSA	10036024	5
POST VACCINATION SYNDROME	10036242	5
PREALBUMIN	10036508	5
PRESSURE OF SPEECH	10036649	5

PROTEIN C DECREASED	10037005	5
PROTEIN TOTAL ABNORMAL	10037012	5
PULSE PRESSURE INCREASED	10037482	5
PYODERMA GANGRENOSUM	10037635	5
RED BLOOD CELL NUCLEATED MORPHOLOGY PRESENT	10038165	5
RED BLOOD CELL SCHISTOCYTES PRESENT	10038169	5
RENAL ATROPHY	10038381	5
RETINAL ISCHAEMIA	10038871	5
RETINAL VASCULAR OCCLUSION	10038903	5
SALPINGO-OOPHORECTOMY BILATERAL	10039465	5
SIMPLE PARTIAL SEIZURES	10040703	5
SLEEP DISORDER DUE TO GENERAL MEDICAL CONDITION, INSC	10040986	5
SMEAR SITE UNSPECIFIED NORMAL	10041209	5
SMEAR VAGINA	10041214	5
SOFT TISSUE INJURY	10041291	5
THROMBOCYTOPENIC PURPURA	10043561	5
TRACHEITIS	10044302	5
TUMOUR PAIN	10045171	5
VERTICAL INFECTION TRANSMISSION	10047337	5
VIIITH NERVE INJURY	10047409	5
VIRAL PHARYNGITIS	10047473	5
VITAMIN B6 INCREASED	10047618	5
VITRECTOMY	10047644	5
VITREOUS DEGENERATION	10047649	5
X-RAY THERAPY TO LUNG	10048202	5
XANTHOPSIA	10048216	5
CEREBRAL VENTRICLE DILATATION	10048824	5
NAIL BED BLEEDING	10048891	5
ANAL ABSCESS	10048946	5
BASILAR ARTERY OCCLUSION	10048963	5
CARBOHYDRATE ANTIGEN 19-9	10049073	5
INJECTION SITE JOINT WARMTH	10049262	5
BONE DENSITY DECREASED	10049470	5
ANTITHROMBIN III DECREASED	10049547	5
FEELING GUILTY	10049708	5
URETHRAL HAEMORRHAGE	10049710	5
DYSACUSIS	10049712	5
PEAU D'ORANGE	10049752	5
BLOOD HIV RNA BELOW ASSAY LIMIT	10049829	5
DRY GANGRENE	10049927	5
ENDOLYMPHATIC HYDROPS	10049934	5
FAECAL VOLUME INCREASED	10049939	5
IDIOSYNCRATIC DRUG REACTION	10049998	5

VULVOVAGINAL ULCERATION	10050181	5
TONSILLAR ULCER	10050211	5
SEROLOGY NEGATIVE	10050410	5
SEROLOGY NORMAL	10050411	5
INABILITY TO CRAWL	10050740	5
FREE THYROXINE INDEX	10050742	5
SPECIFIC GRAVITY URINE DECREASED	10050772	5
ITCHING SCAR	10050818	5
TOXOPLASMA SEROLOGY	10050941	5
METAMYELOCYTE COUNT	10050965	5
COMPLEMENT FACTOR C3 DECREASED	10050981	5
ORAL SURGERY	10051059	5
WRIST SURGERY	10051062	5
ELECTROCARDIOGRAM Q WAVE ABNORMAL	10051177	5
DYSENTERY	10051402	5
TOLOSA-HUNT SYNDROME	10051526	5
METASTASES TO PERITONEUM	10051676	5
COSTOVERTEBRAL ANGLE TENDERNESS	10051704	5
ENTEROBACTER INFECTION	10051910	5
FOETAL CYSTIC HYGROMA	10052011	5
CYTOKINE RELEASE SYNDROME	10052015	5
GLARE	10052128	5
OESOPHAGEAL RUPTURE	10052211	5
EXAGGERATED STARTLE RESPONSE	10066482	5
STOMACH MASS	10066792	5
ENDOSCOPIC ULTRASOUND	10067016	5
ELBOW OPERATION	10067100	5
CEREBRAL MICROANGIOPATHY	10067466	5
PERIPHERAL NERVE LESION	10067633	5
SHORTENED CERVIX	10067726	5
FOCAL SEGMENTAL GLOMERULOSCLEROSIS	10067757	5
UPPER AIRWAY OBSTRUCTION	10067775	5
ORAL ALLERGY SYNDROME	10068355	5
DEVICE INTOLERANCE	10068444	5
SPLENIC CALCIFICATION	10068608	5
BLOOD BETA-D-GLUCAN	10068725	5
BLOOD CHROMOGRANIN A	10068766	5
VON WILLEBRAND'S FACTOR ACTIVITY TEST	10068985	5
VON WILLEBRAND'S FACTOR ANTIGEN TEST	10068988	5
MASTOID EFFUSION	10069008	5
GASTROINTESTINAL VIRAL INFECTION	10069049	5
INTRAMEDULLARY ROD INSERTION	10069066	5
GROSS MOTOR DELAY	10069118	5

PERIPROSTHETIC FRACTURE	10069135	5
SEPTIC ENCEPHALOPATHY	10069141	5
EYELID INJURY	10069200	5
EYE PATCH APPLICATION	10069647	5
SPINAL COLUMN INJURY	10069689	5
EYEGLASSES THERAPY	10069772	5
TREPONEMA TEST POSITIVE	10070158	5
BORDETELLA TEST	10070277	5
COXSACKIE VIRUS TEST NEGATIVE	10070393	5
ASPERGILLUS TEST NEGATIVE	10070449	5
NAIL BED DISORDER	10070533	5
ADMINISTRATION SITE RASH	10071156	5
VULVOVAGINAL INJURY	10071212	5
HEPATITIS B CORE ANTIBODY	10071342	5
SEIZURE CLUSTER	10071350	5
CAPSULE ENDOSCOPY	10071360	5
INFECTED DERMAL CYST	10071367	5
FOETAL NON-STRESS TEST NORMAL	10071517	5
VASCULAR ENDOTHELIAL GROWTH FACTOR ASSAY	10071597	5
PERIORBITAL HAEMORRHAGE	10071697	5
CEREBRAL CAVERNOUS MALFORMATION	10071747	5
ENZYME ACTIVITY NORMAL	10072022	5
OCULAR ROSACEA	10072139	5
COMPUTERISED TOMOGRAM KIDNEY NORMAL	10072169	5
NECK SURGERY	10072227	5
FOETAL PLACENTAL THROMBOSIS	10072240	5
PERIPHERAL ARTERY STENOSIS	10072563	5
HAEMANGIOMA OF BONE	10072572	5
EYELID THICKENING	10072719	5
NON-TOBACCO USER	10072840	5
HUMAN METAPNEUMOVIRUS TEST POSITIVE	10072859	5
HEPATOCELLULAR CARCINOMA	10073071	5
INVASIVE LOBULAR BREAST CARCINOMA	10073096	5
ROMBERG TEST POSITIVE	10073242	5
ESCHERICHIA TEST	10073290	5
BILIRUBIN CONJUGATED DECREASED	10073405	5
COXIELLA TEST	10073454	5
COLONIC ABSCESS	10073573	5
VISUAL PERSEVERATION	10073653	5
OBSTRUCTIVE SHOCK	10073708	5
MICROEMBOLISM	10073734	5
REVERSED HOT-COLD SENSATION	10073738	5
PNEUMOCYSTIS JIROVECII PNEUMONIA	10073755	5

VASCULAR CATHETERISATION	10074169	5
CARDIOPULMONARY EXERCISE TEST	10074357	5
AIRWAY SECRETION CLEARANCE THERAPY	10074363	5
FOETAL HEART RATE DECELERATION ABNORMALITY	10074636	5
MONOCLONAL B-CELL LYMPHOCYTOSIS	10074668	5
ADJUSTED CALCIUM	10074756	5
HAIR INJURY	10074924	5
ADMINISTRATION SITE SWELLING	10075107	5
ROOT CANAL INFECTION	10075136	5
ANTI-MUELLERIAN HORMONE LEVEL	10075156	5
NODULAR RASH	10075807	5
VACCINATION SITE HYPERAESTHESIA	10076165	5
VACCINATION SITE NERVE DAMAGE	10076184	5
OVERGROWTH FUNGAL	10076272	5
PRODUCT SELECTION ERROR	10076542	5
ACUTE MOTOR-SENSORY AXONAL NEUROPATHY	10076657	5
DECREASED NASOLABIAL FOLD	10076861	5
CYTOKINE ABNORMAL	10077351	5
HYPOBARISM	10077606	5
TOTAL COMPLEMENT ACTIVITY INCREASED	10077884	5
NON-24-HOUR SLEEP-WAKE DISORDER	10078086	5
THYROID CALCIFICATION	10078379	5
AMNIOTIC MEMBRANE RUPTURE TEST POSITIVE	10078834	5
TRANSIENT LINGUAL PAPILLITIS	10079293	5
MICROVASCULAR CRANIAL NERVE PALSY	10079491	5
ORTHOSTATIC HEART RATE TEST	10079786	5
PALATE INJURY	10079812	5
PRODUCT COMMUNICATION ISSUE	10080099	5
IMAGING PROCEDURE ARTIFACT	10080358	5
PORTOGRAM	10080520	5
ANTIGLIADIN ANTIBODY	10080857	5
GALLBLADDER HYPOFUNCTION	10080936	5
LEPTOSPIRA TEST	10080944	5
RED BLOOD CELL SCHISTOCYTES	10080983	5
DEGENERATIVE BONE DISEASE	10081730	5
PRODUCT PRESCRIBING ERROR	10081770	5
CENTRAL NERVOUS SYSTEM VASCULITIS	10081778	5
HYPERSENSITIVITY PNEUMONITIS	10081988	5
MAGNETIC RESONANCE ELASTOGRAPHY	10082070	5
AUTOMATIC POSITIVE AIRWAY PRESSURE	10082223	5
LIP SCAB	10082767	5
PITUITARY SCAN	10082813	5
PERIPHERAL VEIN THROMBUS EXTENSION	10082853	5

COMPUTERISED TOMOGRAM PANCREAS ABNORMAL	10082936	5
SEPTIC PULMONARY EMBOLISM	10083093	5
MAGNETIC RESONANCE IMAGING BREAST NORMAL	10083125	5
CONGESTIVE HEPATOPATHY	10084058	5
IMPERCEPTION	10084197	5
DISSEMINATED VARICELLA ZOSTER VIRUS INFECTION	10084396	5
WRONG ROUTE	10084398	5
FOCAL PERITONITIS	10084697	5
OXYGENATION INDEX	10084783	5
LEFT ATRIAL APPENDAGE CLOSURE IMPLANT	10085044	5
MORTON'S NEURALGIA	10052288	5
VASCULAR CAUTERISATION	10052679	5
URODYNAMICS MEASUREMENT	10052685	5
INTRA-AORTIC BALLOON PLACEMENT	10052989	5
CRYSTAL URINE	10053114	5
THERAPEUTIC RESPONSE DELAYED	10053181	5
INGUINAL MASS	10053217	5
INFUSION SITE PAIN	10053483	5
TRACHEAL DISORDER	10053490	5
URINE PROTEIN/CREATININE RATIO INCREASED	10053538	5
ELECTROCARDIOGRAM PR PROLONGATION	10053657	5
CSF MONOCYTE COUNT	10053799	5
CSF NEUTROPHIL COUNT INCREASED	10053803	5
CAROTID PULSE ABNORMAL	10053928	5
RADIAL PULSE ABNORMAL	10053937	5
CARDIAC PHARMACOLOGIC STRESS TEST	10054017	5
SKIN CANDIDA	10054152	5
INCREASED VISCOSITY OF BRONCHIAL SECRETION	10054761	5
BONE TUBERCULOSIS	10056377	5
BILIARY DYSKINESIA	10056529	5
PERIPHERAL SENSORIMOTOR NEUROPATHY	10056673	5
INTRA-OCULAR INJECTION	10057098	5
BIFASCICULAR BLOCK	10057393	5
CARDIAC VENTRICULAR DISORDER	10057455	5
NASAL MUCOSAL DISCOLOURATION	10057537	5
SPINAL MYELOGRAM	10057563	5
VASCULAR DEMENTIA	10057678	5
MUSCLE ENZYME	10057951	5
PANEL-REACTIVE ANTIBODY	10058280	5
DYSPLASIA	10058314	5
BLOOD BILIRUBIN ABNORMAL	10058477	5
ANTI-PLATELET ANTIBODY	10058542	5
VITAMIN B2	10058767	5

GENITAL TRACT INFLAMMATION	10058821	5
REFUSAL OF EXAMINATION	10058840	5
BICYTOPENIA	10058956	5
INCISION SITE COMPLICATION	10059048	5
CSF PRESSURE	10059213	5
VENIPUNCTURE	10059445	5
METAMYELOCYTE PERCENTAGE	10059469	5
LDL/HDL RATIO	10059693	5
JUGULAR VEIN DISTENSION	10059865	5
BLOOD UREA NITROGEN/CREATININE RATIO DECREASED	10059937	5
LE CELLS	10059939	5
BLOOD ALCOHOL	10059963	5
LIGHT CHAIN ANALYSIS INCREASED	10060084	5
HUMAN HERPES VIRUS 6 SEROLOGY	10060114	5
ANTIPSYCHOTIC DRUG LEVEL	10060142	5
PULMONARY ARTERIAL PRESSURE ABNORMAL	10060233	5
ANTICOAGULATION DRUG LEVEL BELOW THERAPEUTIC	10060321	5
RETENTION CYST	10060721	5
ILIOTIBIAL BAND SYNDROME	10060813	5
LIGHT CHAIN ANALYSIS NORMAL	10060866	5
INTRAOCULAR PRESSURE TEST ABNORMAL	10060951	5
BENIGN NEOPLASM	10060999	5
HAEMOPHILUS INFECTION	10061190	5
INTRA-ABDOMINAL HAEMORRHAGE	10061249	5
MIDDLE EAR DISORDER	10061290	5
PNEUMONIA FUNGAL	10061354	5
TOXICOLOGIC TEST ABNORMAL	10061382	5
SENSORY LEVEL ABNORMAL	10061567	5
VITAMIN B COMPLEX DEFICIENCY	10061580	5
AORTIC VALVE DISEASE	10061589	5
PARAINFLUENZAE VIRUS INFECTION	10061907	5
BONE SCAN ABNORMAL	10061941	5
BONE SCAN NORMAL	10061942	5
GASTROINTESTINAL SCAN	10061945	5
SEVERE ACUTE RESPIRATORY SYNDROME	10061982	5
HEPATECTOMY	10061997	5
LUNG OPERATION	10062043	5
KNEE DEFORMITY	10062061	5
RESPIRATORY TRACT INFECTION VIRAL	10062106	5
SENSORIMOTOR DISORDER	10062162	5
VITAMIN D ABNORMAL	10062188	5
MICROANGIOPATHY	10062198	5
URINE CANNABINOIDS INCREASED	10062228	5

UTERINE OPERATION	10062234	5
SHOULDER DEFORMITY	10062241	5
SKIN OPERATION	10062251	5
COUGH VARIANT ASTHMA	10063076	5
CENTRAL PAIN SYNDROME	10064012	5
CARDIAC SEPTAL DEFECT	10064021	5
GASTROINTESTINAL TUBE REMOVAL	10064069	5
HUMAN PAPILLOMA VIRUS TEST POSITIVE	10064328	5
HUMAN PAPILLOMA VIRUS TEST	10064542	5
BREAKTHROUGH PAIN	10064556	5
MICROGRAPHIC SKIN SURGERY	10064836	5
COITAL BLEEDING	10065019	5
UROGRAM	10065096	5
PULMONARY HILAR ENLARGEMENT	10065291	5
CEREBRAL HYPOPERFUSION	10065384	5
HER2 POSITIVE BREAST CANCER	10065430	5
CEREBRAL ARTERIOSCLEROSIS	10065559	5
OESOPHAGEAL FOOD IMPACTION	10065567	5
MIDDLE EAR INFLAMMATION	10065838	5
ANEURYSM REPAIR	10066050	5
INTRACARDIAC MASS	10066087	5
PLASMA CELL MASTITIS	10066170	5
TOOTH RESTORATION	10085637	5
HEAVY METAL TEST	10086051	5
RESPIRATORY PATHOGEN PANEL	10086457	5
ABSTAINS FROM ALCOHOL	10000340	4
ACCIDENT AT HOME	10000370	4
ACCIDENT AT WORK	10000372	4
ACTINIC KERATOSIS	10000614	4
ACUTE PROMYELOCYTIC LEUKAEMIA	10001019	4
ADMINISTRATION SITE REACTION	10001315	4
ALPHA 1 FOETOPROTEIN INCREASED	10001781	4
ALPHA 1 GLOBULIN	10001783	4
ANAL FISSURE	10002153	4
ANTIMITOCHONDRIAL ANTIBODY NORMAL	10002798	4
ANTIMITOCHONDRIAL ANTIBODY POSITIVE	10002799	4
ARTERIAL CATHETERISATION ABNORMAL	10003149	4
ARTERIAL INJURY	10003162	4
ARTHROGRAM	10003278	4
ATROPHIC VULVOVAGINITIS	10003693	4
BENIGN HYDATIDIFORM MOLE	10004272	4
BIOPSY BRAIN ABNORMAL	10004742	4
BIOPSY LIVER NORMAL	10004793	4

BIOPSY TONGUE ABNORMAL	10004892	4
BLOOD BILIRUBIN UNCONJUGATED INCREASED	10005370	4
BLOOD BILIRUBIN UNCONJUGATED NORMAL	10005371	4
BLOOD CREATINE ABNORMAL	10005462	4
BLOOD CREATINE DECREASED	10005463	4
BLOOD FOLLICLE STIMULATING HORMONE ABNORMAL	10005532	4
BLOOD FOLLICLE STIMULATING HORMONE DECREASED	10005533	4
BLOOD IMMUNOGLOBULIN A DECREASED	10005585	4
BLOOD INSULIN INCREASED	10005614	4
BLOOD IRON ABNORMAL	10005617	4
BLOOD LEAD	10005639	4
BLOOD OESTROGEN NORMAL	10005689	4
BRAIN ABSCESS	10006105	4
BRAIN NEOPLASM MALIGNANT	10006131	4
BREAST CANCER STAGE I	10006199	4
BREAST CANCER STAGE IV	10006202	4
BUNION OPERATION	10006586	4
CARCINOID TUMOUR	10007275	4
CARDIAC OUTPUT DECREASED	10007595	4
CERVICAL CORD COMPRESSION	10008252	4
CERVICAL DYSPLASIA	10008263	4
CHOLECYSTOSTOMY	10008624	4
CHONDRITIS	10008685	4
CLUBBING	10009691	4
COAGULATION FACTOR VIII LEVEL INCREASED	10009770	4
COLOUR VISION TESTS	10010055	4
CONDUCTION DISORDER	10010276	4
CONTACT LENS INTOLERANCE	10010804	4
CONTRAST MEDIA REACTION	10010836	4
COOMBS DIRECT TEST NEGATIVE	10010931	4
COWS MILK FREE DIET	10011244	4
COXSACKIE VIRAL INFECTION	10011261	4
CREATININE RENAL CLEARANCE NORMAL	10011374	4
CSF CELL COUNT INCREASED	10011522	4
DEAFNESS PERMANENT	10011894	4
DELUSIONAL DISORDER, UNSPECIFIED TYPE	10012255	4
DELUSIONAL PERCEPTION	10012258	4
DERMATITIS INFECTED	10012470	4
DIABETES INSIPIDUS	10012599	4
DIABETIC COMA	10012650	4
DIABETIC RETINOPATHY	10012689	4
DIAPHRAGM MUSCLE WEAKNESS	10012708	4
DIRECTIONAL DOPPLER FLOW TESTS	10013047	4

DISSOCIATIVE AMNESIA	10013461	4
DRUG DEPENDENCE	10013663	4
DRUG LEVEL DECREASED	10013718	4
ECTOPIC PREGNANCY TERMINATION	10014168	4
ELECTROCARDIOGRAM QRS COMPLEX PROLONGED	10014380	4
ENANTHEMA	10014579	4
ENDOCARDITIS BACTERIAL	10014666	4
FOETAL DISTRESS SYNDROME	10016855	4
FORCEPS DELIVERY	10016991	4
FRONTAL SINUS OPERATION	10017379	4
GLOMERULAR FILTRATION RATE ABNORMAL	10018356	4
GLOSSOPHARYNGEAL NEURALGIA	10018391	4
GLUCOSE-6-PHOSPHATE DEHYDROGENASE DEFICIENCY	10018444	4
GLYCOSYLATED HAEMOGLOBIN ABNORMAL	10018481	4
GRANULOCYTE COUNT DECREASED	10018681	4
HAEMORRHAGE SUBEPIDERMAL	10019001	4
HEPATIC NECROSIS	10019692	4
HEPATITIS ALCOHOLIC	10019728	4
HETEROPHORIA	10020015	4
HYDROSALPINX	10020531	4
HYPERCHLORAEMIA	10020597	4
HYPERPLASIA	10020718	4
HYPERTRIGLYCERIDAEMIA	10020869	4
HYPERTROPHY	10020880	4
HYPERURICAEMIA	10020903	4
HYPOKALAEMIC SYNDROME	10021017	4
HYSTEROSALPINGOGRAM	10021167	4
ILEUS PARALYTIC	10021333	4
IMMUNOGLOBULINS DECREASED	10021499	4
IMMUNOGLOBULINS INCREASED	10021500	4
INDIFFERENCE	10021703	4
INGUINAL HERNIA	10022016	4
INTERNAL FIXATION OF FRACTURE	10022576	4
INTRA-CEREBRAL ANEURYSM OPERATION	10022736	4
IODINE UPTAKE INCREASED	10022922	4
IRON BINDING CAPACITY TOTAL ABNORMAL	10022961	4
KELOID SCAR	10023330	4
LARYNGOSCOPY NORMAL	10023890	4
LEIOMYOSARCOMA	10024189	4
LICHENIFICATION	10024438	4
LIPOATROPHY	10024604	4
LUNG LOBECTOMY	10024741	4
LOOSE BODY IN JOINT	10024829	4

LUNG ADENOCARCINOMA	10025031	4
LUNG CARCINOMA CELL TYPE UNSPECIFIED STAGE IV	10025070	4
MACULOPATHY	10025425	4
MALIGNANT ASCITES	10025538	4
MALIGNANT PLEURAL EFFUSION	10026673	4
METABOLIC ALKALOSIS	10027423	4
MUCOSA VESICLE	10028103	4
MULTIPLE INJURIES	10028224	4
MULTIPLE PREGNANCY	10028243	4
MUSCULAR DYSTROPHY	10028356	4
MYASTHENIC SYNDROME	10028424	4
NEPHRECTOMY	10029116	4
NORMOCHROMIC NORMOCYTIC ANAEMIA	10029783	4
OESTRADIOL DECREASED	10030229	4
OMENTECTOMY	10030305	4
OPTIC ATROPHY	10030910	4
ORAL DISCHARGE	10030972	4
OVARIAN CYSTECTOMY	10033137	4
OVERGROWTH BACTERIAL	10033302	4
PERIODONTITIS	10034539	4
PERIPHERAL MOTOR NEUROPATHY	10034580	4
PICKWICKIAN SYNDROME	10035004	4
PITYRIASIS RUBRA PILARIS	10035116	4
PLACENTA PRAEVIA HAEMORRHAGE	10035121	4
PLEURAL DISORDER	10035595	4
PNEUMONIA LEGIONELLA	10035718	4
POVERTY OF SPEECH	10036467	4
PUSTULAR PSORIASIS	10037575	4
QUADRIPLEGIA	10037714	4
RADIAL NERVE INJURY	10037749	4
RASH SCARLATINIFORM	10037890	4
RECURRENT CANCER	10038111	4
RENAL ABSCESS	10038351	4
RENAL ARTERY STENOSIS	10038378	4
RENAL ARTERY THROMBOSIS	10038380	4
RENIN	10038555	4
RESPIRATORY TRACT HAEMORRHAGE	10038727	4
RETAINED PRODUCTS OF CONCEPTION	10038773	4
RETICULOCYTE COUNT DECREASED	10038790	4
RETINOPATHY	10038923	4
SALMONELLOSIS	10039447	4
SCARLET FEVER	10039587	4
SINGLE PHOTON EMISSION COMPUTERISED TOMOGRAM	10040722	4

SKULL X-RAY NORMAL	10040966	4
SMOOTH MUSCLE ANTIBODY NEGATIVE	10041228	4
SOLILOQUY	10041308	4
SPINAL DECOMPRESSION	10041563	4
SPIROMETRY ABNORMAL	10041630	4
STEATORRHOEA	10041969	4
STRESS AT WORK	10042210	4
SUBCUTANEOUS HAEMATOMA	10042345	4
SUBDURAL HAEMATOMA EVACUATION	10042363	4
SURGICAL PROCEDURE REPEATED	10042618	4
SYNOVIAL RUPTURE	10042862	4
SYRINGOMYELIA	10042928	4
TEMPORAL LOBE EPILEPSY	10043209	4
THOUGHT BLOCKING	10043495	4
TINEA PEDIS	10043873	4
TONSILLECTOMY	10044006	4
TOXIC EPIDERMAL NECROLYSIS	10044223	4
TRICHOMONIASIS	10044620	4
TYMPANOMETRY NORMAL	10045216	4
ULNAR NERVE INJURY	10045378	4
ULTRASOUND BLADDER ABNORMAL	10045407	4
URETHRAL DISORDER	10046445	4
URETHRAL SPASM	10046464	4
URETHRITIS	10046480	4
VACUUM EXTRACTOR DELIVERY	10046868	4
VENOUS INJURY	10047228	4
VENTRICULO-PERITONEAL SHUNT	10047304	4
VERTEBROBASILAR INSUFFICIENCY	10047334	4
VERTIGO CNS ORIGIN	10047343	4
WHITE BLOOD CELLS STOOL POSITIVE	10047963	4
WOUND DEHISCENCE	10048031	4
BIOPSY CHORIONIC VILLOUS	10048536	4
PERIPHERAL PULSE DECREASED	10048718	4
MULTIPLE-DRUG RESISTANCE	10048723	4
COLON ADENOMA	10048832	4
RECTAL EXAMINATION	10048892	4
VAGINAL MUCOSAL BLISTERING	10048904	4
PERFORMANCE STATUS DECREASED	10048919	4
OTOSCOPY	10048929	4
URINARY TRACT INFECTION FUNGAL	10049059	4
VASCULAR GRAFT OCCLUSION	10049060	4
SALIVA DISCOLOURATION	10049069	4
OCULAR MYASTHENIA	10049168	4

GINGIVAL INJURY	10049300	4
SPINAL CORD INFECTION	10049654	4
HOMICIDAL IDEATION	10049666	4
LHERMITTE'S SIGN	10049690	4
PERICARDIAL RUB	10049759	4
CALCIFICATION OF MUSCLE	10049797	4
SINGLE UMBILICAL ARTERY	10049807	4
CARDIAC DEATH	10049993	4
APPLICATION SITE BRUISE	10050114	4
LYMPH NODE ABSCESS	10050167	4
MACROCEPHALY	10050183	4
SELF ESTEEM DECREASED	10050280	4
OTIC EXAMINATION	10050319	4
PROSTATIC SPECIFIC ANTIGEN	10050704	4
BAND NEUTROPHIL COUNT INCREASED	10050759	4
HYPOCHROMASIA	10050789	4
ORAL DYSAESTHESIA	10050820	4
BREAST PROSTHESIS USER	10050854	4
DENTAL CLEANING	10051047	4
CARDIOPULMONARY FAILURE	10051093	4
INCISION SITE HAEMORRHAGE	10051100	4
FOETAL HEART RATE NORMAL	10051140	4
DIABETIC GASTROPARESIS	10051153	4
FLUID IMBALANCE	10051283	4
DIABETES MELLITUS MANAGEMENT	10051599	4
INTRANASAL PARAESTHESIA	10051660	4
ESCHAR	10051814	4
ASTHMA PROPHYLAXIS	10066654	4
LICHEN STRIATUS	10066945	4
RETINOPEXY	10066985	4
TONGUE ABSCESS	10066991	4
MENSTRUAL CYCLE MANAGEMENT	10067029	4
CENTRAL VENOUS CATHETER REMOVAL	10067098	4
INJECTION SITE LACERATION	10067253	4
THROMBOTIC CEREBRAL INFARCTION	10067347	4
DECREASED VIBRATORY SENSE	10067502	4
LOW BIRTH WEIGHT BABY	10067508	4
NEUROSENSORY HYPOACUSIS	10067587	4
WINGED SCAPULA	10067628	4
PREMATURE OVULATION	10068042	4
CEREBRAL AMYLOID ANGIOPATHY	10068044	4
INHIBITORY DRUG INTERACTION	10068071	4
FOREIGN BODY REACTION	10068159	4

ANDROGENETIC ALOPECIA	10068168	4
CARDIORENAL SYNDROME	10068230	4
REFLEX TEST ABNORMAL	10068273	4
MAMMOPLASTY	10068647	4
VIRAL CARDIOMYOPATHY	10068767	4
PERIPORTAL OEDEMA	10068821	4
CEREBROSPINAL FLUID DRAINAGE	10068892	4
TONGUE PIGMENTATION	10069164	4
SMOKE SENSITIVITY	10069201	4
PRODUCT FORMULATION ISSUE	10069228	4
PRODUCT SUBSTITUTION ISSUE	10069326	4
VALSALVA MANEUVER	10069420	4
ORTHOSTATIC HEART RATE RESPONSE INCREASED	10069431	4
CSF IMMUNOGLOBULIN G INDEX	10069597	4
SUBCLAVIAN ARTERY OCCLUSION	10069695	4
BIOPSY SOFT TISSUE	10069752	4
H1N1 INFLUENZA	10069767	4
OMENTAL INFARCTION	10069886	4
BORDETELLA TEST POSITIVE	10070010	4
ENTEROBACTER TEST POSITIVE	10070023	4
MYCOPLASMA TEST POSITIVE	10070160	4
HEPATITIS B VIRUS TEST POSITIVE	10070217	4
HEPATITIS C VIRUS TEST POSITIVE	10070218	4
STREPTOCOCCAL URINARY TRACT INFECTION	10070300	4
PARVOVIRUS B19 TEST POSITIVE	10070338	4
ASPERGILLUS TEST	10070450	4
WRONG DEVICE USED	10070468	4
OESOPHAGEAL IRRITATION	10070818	4
FEMOROACETABULAR IMPINGEMENT	10070899	4
HUMAN ANAPLASMOSIS	10071038	4
CLINICALLY ISOLATED SYNDROME	10071068	4
IDIOPATHIC GENERALISED EPILEPSY	10071081	4
AUTOIMMUNE ARTHRITIS	10071155	4
IMPAIRED REASONING	10071176	4
HEPATITIS B SURFACE ANTIBODY NEGATIVE	10071347	4
MACULAR FIBROSIS	10071392	4
ANTI-AQUAPORIN-4 ANTIBODY	10071466	4
PSEUDOHYPONATRAEMIA	10072126	4
PRERENAL FAILURE	10072370	4
ANTI-AQUAPORIN-4 ANTIBODY NEGATIVE	10072507	4
ANTI-GANGLIOSIDE ANTIBODY POSITIVE	10072516	4
PERIPHERAL ENDARTERECTOMY	10072560	4
PERIPHERAL ARTERY STENT INSERTION	10072562	4

CONTRACEPTIVE IMPLANT	10072571	4
KLEIHAUER-BETKE TEST NEGATIVE	10072576	4
ORTHOPAEDIC EXAMINATION	10072624	4
ORTHOPAEDIC EXAMINATION ABNORMAL	10072625	4
HAND-EYE COORDINATION IMPAIRED	10072649	4
HUMIDITY INTOLERANCE	10072791	4
MACROPHAGE COUNT	10072857	4
ADNEXAL TORSION	10072943	4
TERM BIRTH	10072953	4
LOBULAR BREAST CARCINOMA IN SITU	10073099	4
ROMBERG TEST	10073238	4
EXPOSURE VIA EYE CONTACT	10073336	4
DECEREBRATE POSTURE	10073346	4
INJECTION SITE JOINT DISCOMFORT	10073459	4
INSTILLATION SITE PARAESTHESIA	10073558	4
PHARYNGEAL ENANTHEMA	10073741	4
TRIGEMINAL NEURITIS	10074054	4
T-CELL RECEPTOR GENE REARRANGEMENT TEST	10074077	4
AXILLARY WEB SYNDROME	10074387	4
VENA CAVA FILTER REMOVAL	10074397	4
CATHETER MANAGEMENT	10074414	4
DURAL ARTERIOVENOUS FISTULA	10074462	4
ADAMTS13 ACTIVITY NORMAL	10074492	4
LARGE INTESTINAL POLYPECTOMY	10074625	4
INCISION SITE SWELLING	10074758	4
CAFFEINE ALLERGY	10074895	4
TONIC POSTURING	10075125	4
NEUROPATHIC MUSCULAR ATROPHY	10075469	4
STOMA SITE DISCHARGE	10075512	4
AUTOIMMUNE DEMYELINATING DISEASE	10075688	4
ADMINISTRATION SITE NODULE	10075765	4
AMNIOTIC FLUID INDEX DECREASED	10075866	4
VACCINATION SITE ECZEMA	10076161	4
TRANSCRIPTION MEDICATION ERROR	10076245	4
INJECTION SITE JOINT ERYTHEMA	10076327	4
TUMOUR MARKER DECREASED	10076385	4
LUMBOSACRAL RADICULOPATHY	10076578	4
ACUTE MOTOR AXONAL NEUROPATHY	10076658	4
EXTERNAL COMPRESSION HEADACHE	10076669	4
ALLERGY TO SYNTHETIC FABRIC	10076764	4
TRANSCATHETER AORTIC VALVE IMPLANTATION	10077015	4
VASCULAR STENT STENOSIS	10077144	4
MYELOPROLIFERATIVE NEOPLASM	10077465	4

SENSORY PROCESSING DISORDER	10077516	4
PALATAL ULCER	10077519	4
TENOPLASTY	10077558	4
PRODUCT SUPPLY ISSUE	10077801	4
OBSESSIVE-COMPULSIVE SYMPTOM	10077894	4
ILLNESS ANXIETY DISORDER	10078077	4
GENITO-PELVIC PAIN/PENETRATION DISORDER	10078087	4
FOETAL BIOPHYSICAL PROFILE SCORE ABNORMAL	10078124	4
PULMONARY ARTERY OCCLUSION	10078201	4
ABDOMINAL WALL WOUND	10078479	4
CONTRAINDICATED PRODUCT ADMINISTERED	10078504	4
ULTRASOUND PANCREAS ABNORMAL	10078673	4
AUDITORY NERVE DISORDER	10078794	4
DECREASED EMBRYO VIABILITY	10078930	4
RENAL TUBULAR INJURY	10078933	4
DISEASE RISK FACTOR	10078950	4
DENTAL RESTORATION FAILURE	10078978	4
FOETAL RENAL IMPAIRMENT	10078987	4
PSYCHOTIC SYMPTOM	10079254	4
STIFF LEG SYNDROME	10079359	4
PRODUCT USE COMPLAINT	10079400	4
DRAIN SITE COMPLICATION	10079626	4
SERUM SEROTONIN	10079787	4
MANDIBULAR MASS	10079878	4
TRANSIENT APHASIA	10080106	4
ENTEROCOCCUS TEST	10080177	4
IRREGULAR SLEEP WAKE RHYTHM DISORDER	10080301	4
WRONG SCHEDULE	10080303	4
ANTI-HLA ANTIBODY TEST	10080511	4
PERIORBITAL INFLAMMATION	10080722	4
NOROVIRUS TEST	10080822	4
RHESUS ANTIGEN	10080952	4
WRONG DOSAGE FORMULATION	10080975	4
LITHIASIS	10081111	4
OPHTHALMIC ARTERY THROMBOSIS	10081144	4
BACILLE CALMETTE-GUERIN SCAR REACTIVATION	10081210	4
INTRAOSSEOUS ACCESS PLACEMENT	10081231	4
ALCOHOL TEST	10081296	4
MUSCLE TENSION DYSPHONIA	10081496	4
ANTI-GLOMERULAR BASEMENT MEMBRANE DISEASE	10081981	4
NONALCOHOLIC FATTY LIVER DISEASE	10082249	4
ANTI-RNA POLYMERASE III ANTIBODY NEGATIVE	10082282	4
SKIN TEMPERATURE	10082526	4

LYMPH NODE RUPTURE	10082725	4
FLUORESCENCE ANGIOGRAM	10083089	4
HORMONE RECEPTOR POSITIVE BREAST CANCER	10083234	4
INTERNAL CAPSULE INFARCTION	10083408	4
ADENOCARCINOMA METASTATIC	10083456	4
COMPUTERISED TOMOGRAM AORTA	10083572	4
CARDIAC PERFUSION DEFECT	10083602	4
PHARYNGEAL PUSTULE	10084148	4
NEONATAL DYSPNOEA	10084238	4
SARS-COV-2 TEST FALSE NEGATIVE	10084480	4
TROPONIN I ABNORMAL	10084969	4
TROPONIN I DECREASED	10084972	4
MAGNETIC RESONANCE IMAGING HEPATOBILIARY	10085120	4
MAGNETIC RESONANCE IMAGING HEPATOBILIARY ABNORMAI	10085121	4
QUANTITATIVE SENSORY TESTING	10085180	4
LYMPHATIC OBSTRUCTION	10052315	4
BRAIN CONTUSION	10052346	4
GASTROINTESTINAL HYPERMOTILITY	10052402	4
LARGE INTESTINAL HAEMORRHAGE	10052534	4
DRUG TOLERANCE DECREASED	10052805	4
ACQUIRED OESOPHAGEAL WEB	10052820	4
ALLEN'S TEST	10053463	4
INFUSION SITE WARMTH	10053484	4
VENOUS ANGIOMA OF BRAIN	10053485	4
BLOOD TESTOSTERONE FREE INCREASED	10053785	4
BACTERIAL SEPSIS	10053840	4
RADIAL PULSE	10053935	4
POSTOPERATIVE ILEUS	10054048	4
GENITAL ERYTHEMA	10054816	4
MYOCLONIC EPILEPSY	10054859	4
INTERVERTEBRAL DISC COMPRESSION	10055039	4
THYROXINE FREE ABNORMAL	10055158	4
PNEUMONIA NECROTISING	10055672	4
HAEMATOMA MUSCLE	10055890	4
TINEA VERSICOLOUR	10056131	4
PAIN MANAGEMENT	10056350	4
TRACHEOMALACIA	10056397	4
DEHYDROEPIANDROSTERONE DECREASED	10056629	4
PSEUDOCYST	10056658	4
WEIGHT ABNORMAL	10056814	4
SALIVARY GLAND MASS	10057002	4
BLAST CELLS PRESENT	10057107	4
IRRITABILITY POSTVACCINAL	10057224	4

CONNECTIVE TISSUE INFLAMMATION	10057254	4
VASCULAR TEST ABNORMAL	10057458	4
VASCULAR TEST NORMAL	10057523	4
CARDIAC SEPTAL HYPERTROPHY	10057576	4
PROCEDURAL COMPLICATION	10057765	4
VENTRICULAR INTERNAL DIAMETER	10057921	4
CHEMICAL BURN OF SKIN	10057941	4
MUSCLE ENZYME INCREASED	10057945	4
RHEUMATOID FACTOR DECREASED	10057961	4
GLOBULINS DECREASED	10058001	4
HYPEREXPLEXIA	10058271	4
MENOPAUSAL DISORDER	10058825	4
DISTURBANCE IN SEXUAL AROUSAL	10058929	4
THALAMUS HAEMORRHAGE	10058939	4
ILIAC VEIN OCCLUSION	10058992	4
RETINAL WHITE DOTS SYNDROME	10059081	4
INFLUENZA IMMUNISATION	10059429	4
SYNOVIAL FLUID ANALYSIS ABNORMAL	10059526	4
ENDOCRINE TEST NORMAL	10059700	4
ANTI-GAD ANTIBODY	10059729	4
CSF MYELIN BASIC PROTEIN	10059773	4
PACEMAKER GENERATED RHYTHM	10059831	4
DRUG RESISTANCE	10059866	4
PLATELET MORPHOLOGY	10059906	4
RETICULOCYTE PERCENTAGE INCREASED	10059920	4
GASTRIC PH DECREASED	10060041	4
ANTIBIOTIC LEVEL	10060317	4
ANTICOAGULATION DRUG LEVEL	10060318	4
ANTICOAGULATION DRUG LEVEL THERAPEUTIC	10060319	4
DIABETIC FOOT INFECTION	10060803	4
CALCIUM IONISED	10060900	4
ABDOMINAL NEOPLASM	10060925	4
AMNIOTIC CAVITY INFECTION	10060937	4
BASOPHIL COUNT ABNORMAL	10060978	4
LABOUR COMPLICATION	10061050	4
CRANIAL NERVE INJURY	10061094	4
DISTURBANCE IN SOCIAL BEHAVIOUR	10061108	4
GASTROINTESTINAL INJURY	10061172	4
IMPULSE-CONTROL DISORDER	10061215	4
LYMPHOPROLIFERATIVE DISORDER	10061232	4
INVESTIGATION ABNORMAL	10061253	4
MUCOSAL HAEMORRHAGE	10061298	4
POSTPARTUM DISORDER	10061469	4

SCAN ABDOMEN ABNORMAL	10061502	4
SCAN LYMPH NODES	10061504	4
SCAN SPLEEN	10061506	4
LIP AND/OR ORAL CAVITY CANCER	10061523	4
PITUITARY TUMOUR BENIGN	10061538	4
URINARY TRACT OBSTRUCTION	10061574	4
AMPUTATION	10061627	4
ARTERIOGRAM ABNORMAL	10061659	4
CHONDROCALCINOSIS	10061761	4
COOMBS TEST	10061786	4
DIVERTICULAR PERFORATION	10061820	4
EXTRADURAL ABSCESS	10061846	4
PYELOCALIECTASIS	10061927	4
HEART VALVE REPLACEMENT	10061995	4
INTESTINAL OPERATION	10062024	4
PH BODY FLUID ABNORMAL	10062071	4
RED BLOOD CELL MORPHOLOGY ABNORMAL	10062100	4
STENT REMOVAL	10062115	4
THYROID OPERATION	10062126	4
URINARY SYSTEM X-RAY ABNORMAL	10062138	4
MITRAL VALVE REPAIR	10062202	4
NASAL OPERATION	10062210	4
URINE ELECTROLYTES NORMAL	10062230	4
UTERINE INFECTION	10062233	4
SOFT TISSUE INFECTION	10062255	4
GENITAL ABSCESS	10062316	4
VULVAR EROSION	10062559	4
URETHRITIS NONINFECTIVE	10062903	4
OPIATES POSITIVE	10063232	4
CD4 LYMPHOCYTES INCREASED	10063294	4
DNA TEST FOR FRAGILE X	10063296	4
AMNIOTIC FLUID VOLUME INCREASED	10063357	4
VENOUS STENT INSERTION	10063389	4
RELAPSING-REMITTING MULTIPLE SCLEROSIS	10063399	4
PRURITUS ALLERGIC	10063438	4
PREMATURE AGEING	10063493	4
EROSIVE OESOPHAGITIS	10063655	4
EYELID CYST	10063692	4
IMPLANT SITE PAIN	10063782	4
NON-HIGH-DENSITY LIPOPROTEIN CHOLESTEROL INCREASED	10063967	4
ANTIALLERGIC THERAPY	10064059	4
SOLAR LENTIGO	10064127	4
DEVICE OCCLUSION	10064685	4

DIABETIC DIET	10064725	4
RENAL ARTERY STENT PLACEMENT	10064727	4
NUTRITIONAL CONDITION ABNORMAL	10065026	4
APPARENT LIFE THREATENING EVENT	10065044	4
LATENT TUBERCULOSIS	10065048	4
BIOPSY GINGIVAL	10065076	4
UROGRAM NORMAL	10065098	4
FISTULOGRAM	10065149	4
SUICIDAL BEHAVIOUR	10065604	4
DRUG DISPENSED TO WRONG PATIENT	10065634	4
ACTH STIMULATION TEST	10065665	4
BONE LOSS	10065687	4
VAGINAL FISTULA	10065813	4
POST-TUSSIVE VOMITING	10066220	4
CYTOPENIA	10066274	4
PULMONARY ARTERY COMPRESSION	10085390	4
FOETAL VASCULAR MALPERFUSION	10085689	4
HEAVY METAL NORMAL	10086047	4
DERMAL FILLER REACTION	10086476	4
ACETABULUM FRACTURE	10000397	3
ACHLORHYDRIA	10000451	3
ACNE PUSTULAR	10000513	3
ACUTE ABDOMEN	10000647	3
ACUTE LEUKAEMIA	10000830	3
ACUTE STRESS DISORDER	10001084	3
ALANINE AMINOTRANSFERASE ABNORMAL	10001547	3
ALICE IN WONDERLAND SYNDROME	10001666	3
ALKALOSIS	10001680	3
ALOPECIA TOTALIS	10001766	3
ALPHA 1 GLOBULIN INCREASED	10001787	3
AMMONIA DECREASED	10001945	3
AMNIOCENTESIS ABNORMAL	10001959	3
ANIMAL SCRATCH	10002519	3
ARTERIAL RUPTURE	10003173	3
ARTERIAL SPASM	10003175	3
ARTIFICIAL RUPTURE OF MEMBRANES	10003440	3
ASPIRATION JOINT ABNORMAL	10003518	3
ATOPY	10003645	3
ATRIAL SEPTAL DEFECT REPAIR	10003667	3
AURICULAR SWELLING	10003800	3
AXONAL NEUROPATHY	10003882	3
BABESIOSIS	10003965	3
BACTERIAL DISEASE CARRIER	10004017	3

BARTHOLIN'S ABSCESS	10004138	3
BENIGN HEPATIC NEOPLASM	10004269	3
BIOPSY BLADDER	10004730	3
BIOPSY BLADDER ABNORMAL	10004731	3
BIOPSY CERVIX NORMAL	10004755	3
BIOPSY COLON NORMAL	10004761	3
BIOPSY HEART ABNORMAL	10004780	3
BIOPSY KIDNEY NORMAL	10004784	3
BIOPSY LIP	10004788	3
BIOPSY MUSCLE ABNORMAL	10004803	3
BIOPSY PHARYNX	10004851	3
BIOPSY SALIVARY GLAND	10004865	3
BIOPSY SITE UNSPECIFIED NORMAL	10004872	3
BIOPSY STOMACH ABNORMAL	10004880	3
BIOPSY THYROID GLAND ABNORMAL	10004889	3
BLASTOMYCOSIS	10005098	3
BLOOD ALBUMIN ABNORMAL	10005286	3
BLOOD ALDOSTERONE NORMAL	10005297	3
BLOOD COPPER INCREASED	10005443	3
BLOOD CORTICOTROPHIN NORMAL	10005454	3
BLOOD GROUP B	10005567	3
BLOOD LACTATE DEHYDROGENASE ABNORMAL	10005627	3
BLOOD LACTATE DEHYDROGENASE DECREASED	10005629	3
BLOOD LEAD NORMAL	10005643	3
BLOOD PARATHYROID HORMONE DECREASED	10005702	3
BLOOD PRESSURE SYSTOLIC ABNORMAL	10005757	3
BLOOD THROMBOPLASTIN	10005823	3
BLOOD ZINC DECREASED	10005867	3
BLUNTED AFFECT	10005885	3
BODY MASS INDEX INCREASED	10005897	3
BORDERLINE PERSONALITY DISORDER	10006034	3
BRADYPNOEA	10006102	3
BRAIN NEOPLASM BENIGN	10006129	3
BRAIN STEM ISCHAEMIA	10006148	3
BREAST NECROSIS	10006277	3
BREATHING-RELATED SLEEP DISORDER	10006344	3
BRONCHOSTENOSIS	10006487	3
BURN OF INTERNAL ORGANS	10006712	3
CALCULUS BLADDER	10006987	3
CARBOXYHAEMOGLOBIN	10007239	3
CARBUNCLE	10007247	3
CARCINOEMBRYONIC ANTIGEN INCREASED	10007266	3
CARCINOEMBRYONIC ANTIGEN NORMAL	10007267	3

CARDIAC HYPERTROPHY	10007572	3
CEREBELLAR ATROPHY	10008027	3
CERVIX CARCINOMA	10008342	3
CHOLANGITIS SCLEROSING	10008609	3
CHOLECYSTITIS CHRONIC	10008617	3
CHOREOATHETOSIS	10008754	3
CHRONIC HEPATITIS	10008909	3
CHRONIC TONSILLITIS	10009152	3
CIRRHOSIS ALCOHOLIC	10009208	3
CLAUDICATION OF JAW MUSCLES	10009243	3
CLOT RETRACTION	10009668	3
COCHLEA IMPLANT	10009830	3
COLD AGGLUTININS POSITIVE	10009854	3
CONSTRICTED AFFECT	10010778	3
CORNEAL LESION	10011026	3
CRYOGLOBULINS PRESENT	10011478	3
CRYSTAL URINE ABSENT	10011510	3
CSF LYMPHOCYTE COUNT	10011547	3
CSF LYMPHOCYTE COUNT ABNORMAL	10011548	3
CSF PRESSURE NORMAL	10011571	3
CULTURE TISSUE SPECIMEN NEGATIVE	10011636	3
CYST ASPIRATION	10011734	3
CYSTITIS HAEMORRHAGIC	10011793	3
DEVELOPMENTAL DELAY	10012559	3
DIASTOLIC HYPERTENSION	10012758	3
DISLOCATION OF VERTEBRA	10013183	3
DROWNING	10013647	3
DRUG SPECIFIC ANTIBODY PRESENT	10013745	3
ECHOENCEPHALOGRAM	10014119	3
ECHOLALIA	10014127	3
ECZEMA HERPETICUM	10014197	3
ELECTROCARDIOGRAM Q WAVES	10014375	3
ELECTROCOCHLEOGRAM	10014398	3
ENDOMETRITIS	10014791	3
ENDOSCOPIC RETROGRADE CHOLANGIOPANCREATOGRAPHY A	10014803	3
ENDOSCOPY SMALL INTESTINE	10014816	3
EOSINOPHILIC FASCIITIS	10014954	3
EPIGLOTTIC OEDEMA	10015029	3
EXERCISE ELECTROCARDIOGRAM NORMAL	10015646	3
EXERCISE TEST	10015652	3
EXTENSOR PLANTAR RESPONSE	10015727	3
EXTRADURAL HAEMATOMA	10015769	3
EYELID RETRACTION	10015997	3

FAILED INDUCTION OF LABOUR	10016123	3
FALLOT'S TETRALOGY	10016193	3
FAMILY STRESS	10016215	3
FASCIITIS	10016228	3
FIBRIN DEGRADATION PRODUCTS	10016585	3
FORCED EXPIRATORY VOLUME	10016984	3
FUNDOSCOPY NORMAL	10017521	3
GAMMA-GLUTAMYLTRANSFERASE NORMAL	10017694	3
GASTRIC CANCER	10017758	3
GASTRIC PERFORATION	10017815	3
GASTROENTEROSTOMY	10017873	3
GENETIC COUNSELLING	10018138	3
GIARDIASIS	10018262	3
GLUCOSE TOLERANCE TEST ABNORMAL	10018433	3
GLUTEN FREE DIET	10018459	3
GROWING PAINS	10018745	3
HAEMORRHAGIC INFARCTION	10019013	3
HEART SOUNDS NORMAL	10019312	3
HEAT STROKE	10019345	3
HEPATIC INFARCTION	10019680	3
HEPATITIS A	10019719	3
HEPATITIS C ANTIBODY POSITIVE	10019747	3
HEREDITARY ANGIOEDEMA	10019860	3
HERPANGINA	10019936	3
HERPES GESTATIONIS	10019939	3
HISTIOCYTOSIS	10020117	3
HISTOPLASMOSIS	10020141	3
HOLMES-ADIE PUPIL	10020352	3
HUMAN EHRLICHIOSIS	10020429	3
HYPERADRENALISM	10020562	3
HYPERPROTEINAEMIA	10020740	3
HYPNOPOMPIC HALLUCINATION	10020928	3
HYPOTHALAMO-PITUITARY DISORDER	10021111	3
IMMUNODEFICIENCY COMMON VARIABLE	10021449	3
IMMUNOELECTROPHORESIS	10021464	3
INFECTED SKIN ULCER	10021784	3
INFECTION SUSCEPTIBILITY INCREASED	10021866	3
INFERTILITY TESTS	10021931	3
INGROWING NAIL	10022013	3
INJECTION SITE GRANULOMA	10022065	3
INJURY CORNEAL	10022120	3
INTRADUCTAL PAPILLOMA OF BREAST	10022781	3
INTRINSIC FACTOR ANTIBODY POSITIVE	10022855	3

IODINE UPTAKE	10022917	3
IODINE UPTAKE DECREASED	10022920	3
IRREGULAR SLEEP PHASE	10022995	3
KETOSIS	10023391	3
LACTATION PUERPERAL INCREASED	10023671	3
LARGE INTESTINE ANASTOMOSIS	10023800	3
LARYNGEAL STENOSIS	10023862	3
LIVING ALONE	10024729	3
LONG QT SYNDROME	10024803	3
LORDOSIS	10024842	3
LOSS OF LIBIDO	10024870	3
LUNG ADENOCARCINOMA STAGE IV	10025038	3
MALLORY-WEISS SYNDROME	10026712	3
MAMMARY DUCT ECTASIA	10026730	3
MARROW HYPERPLASIA	10026851	3
MEAN ARTERIAL PRESSURE DECREASED	10026983	3
MEDIASTINOSCOPY	10027083	3
MERALGIA PARAESTHETICA	10027385	3
METAL POISONING	10027439	3
METASTASES TO SPINE	10027468	3
MICROANGIOPATHIC HAEMOLYTIC ANAEMIA	10027527	3
MILIA	10027626	3
MYELOID LEUKAEMIA	10028549	3
MYOCARDIAL NECROSIS	10028602	3
MYOCARDIAL RUPTURE	10028604	3
MYOGLOBIN BLOOD INCREASED	10028625	3
MYRINGOTOMY	10028662	3
NAIL PITTING	10028702	3
NAIL PSORIASIS	10028703	3
NEEDLE TRACK MARKS	10028896	3
NEONATAL DISORDER	10028934	3
NEPHROCALCINOSIS	10029146	3
NEURITIS CRANIAL	10029244	3
NEUROSIS	10029333	3
NON-ALCOHOLIC FATTY LIVER	10029530	3
NORMOCHROMIC ANAEMIA	10029782	3
OESOPHAGEAL VARICES HAEMORRHAGE	10030210	3
OESOPHAGOSCOPY	10030222	3
OMPHALITIS	10030306	3
ONYCHOMYCOSIS	10030338	3
OPTIC NERVE SHEATH HAEMORRHAGE	10030941	3
ORGASM ABNORMAL	10031085	3
OSTEOMA	10031249	3

PANCREATITIS RELAPSING	10033657	3
PARAPARESIS	10033885	3
PAROTID DUCT OBSTRUCTION	10034021	3
PAROXYSMAL NOCTURNAL HAEMOGLOBINURIA	10034042	3
PECTUS EXCAVATUM	10034204	3
PELVI-URETERIC OBSTRUCTION	10034232	3
PERICARDIAL HAEMORRHAGE	10034476	3
PERIORBITAL HAEMATOMA	10034544	3
PH URINE INCREASED	10034795	3
PHYSICAL ASSAULT	10034983	3
PLACENTAL INSUFFICIENCY	10035138	3
PLAGUE	10035148	3
PLANTAR FASCIAL FIBROMATOSIS	10035154	3
PNEUMONIA RESPIRATORY SYNCYTIAL VIRAL	10035732	3
PREGNANCY WITH ADVANCED MATERNAL AGE	10036582	3
PRESBYOPIA	10036628	3
PRINZMETAL ANGINA	10036759	3
PROTHROMBIN TIME ABNORMAL	10037057	3
PROTHROMBIN TIME RATIO	10037064	3
PRURIGO	10037083	3
PULMONARY ARTERIAL PRESSURE INCREASED	10037324	3
PULMONARY VASCULITIS	10037457	3
PULMONARY VENOUS THROMBOSIS	10037459	3
PURGING	10037544	3
RAPID EYE MOVEMENTS SLEEP ABNORMAL	10037841	3
RECTAL CANCER	10038038	3
RED BLOOD CELL MICROCYTES PRESENT	10038163	3
RENIN NORMAL	10038561	3
RESORPTION BONE INCREASED	10038642	3
RETINAL ARTERY THROMBOSIS	10038831	3
RETINAL SCAR	10038895	3
RETINAL VASCULAR DISORDER	10038901	3
RHINOPHYMA	10039098	3
ROSEOLA	10039222	3
RUBELLA	10039252	3
SCHIZOAFFECTIVE DISORDER	10039621	3
SENSE OF OPPRESSION	10040007	3
SMALL CELL LUNG CANCER	10041067	3
SPECIFIC GRAVITY URINE ABNORMAL	10041439	3
SPINAL CLAUDICATION	10041539	3
SPINAL CORD DRAINAGE	10041551	3
SQUAMOUS CELL CARCINOMA OF LUNG	10041826	3
STERNAL FRACTURE	10042015	3

STRANGULATED HERNIA	10042166	3
STROKE VOLUME	10042245	3
T-CELL TYPE ACUTE LEUKAEMIA	10042987	3
TATTOO	10043143	3
TEETH BRITTLE	10043173	3
THROMBOPHLEBITIS SEPTIC	10043593	3
THROMBOTIC MICROANGIOPATHY	10043645	3
THYROIDITIS ACUTE	10043780	3
TONGUE HAEMATOMA	10043959	3
TOOTH RESORPTION	10044052	3
TOPOGRAPHY CORNEAL	10044059	3
TOXIC NODULAR GOITRE	10044242	3
TOXIC SHOCK SYNDROME	10044248	3
TOXOPLASMOSIS	10044272	3
TRACHEOBRONCHITIS	10044314	3
TRAUMATIC HAEMATOMA	10044522	3
TRI-IODOTHYRONINE UPTAKE	10044599	3
TUBERCULIN TEST POSITIVE	10044728	3
TYPE IIA HYPERLIPIDAEMIA	10045261	3
ULNAR NEURITIS	10045380	3
ULTRASOUND SKULL	10045441	3
UMBILICAL CORD PROLAPSE	10045452	3
UNEMPLOYMENT	10045520	3
URETERIC STENOSIS	10046411	3
URETEROSCOPY	10046413	3
URINARY TRACT INFECTION ENTEROCOCCAL	10046572	3
URINE OSMOLARITY DECREASED	10046653	3
URINE OSMOLARITY INCREASED	10046654	3
URINE SODIUM DECREASED	10046671	3
UV LIGHT THERAPY	10046850	3
VESICOURETERIC REFLUX	10047370	3
VIRAL PERICARDITIS	10047472	3
VISUAL FIELD TESTS NORMAL	10047568	3
VITAL CAPACITY	10047579	3
VITREOUS DISORDER	10047651	3
VITRITIS	10047663	3
VOCAL CORD THICKENING	10047676	3
VULVOVAGINITIS	10047794	3
WITHDRAWAL BLEED	10047998	3
PREALBUMIN DECREASED	10048436	3
BLADDER PROLAPSE	10048475	3
ENDOTHELIAL DYSFUNCTION	10048554	3
SELF-INDUCED VOMITING	10048636	3

LYMPHOCELE	10048642	3
NEUROGENIC BOWEL	10048657	3
VENOUS STENOSIS	10048671	3
ACUTE GENERALISED EXANTHEMATOUS PUSTULOSIS	10048799	3
ANAEMIA POSTOPERATIVE	10048861	3
HERNIA PAIN	10049077	3
WEIGHT LOSS POOR	10049084	3
PATELLOFEMORAL PAIN SYNDROME	10049143	3
FRACTURED COCCYX	10049164	3
SKIN TURGOR DECREASED	10049428	3
PERIPARTUM CARDIOMYOPATHY	10049430	3
URINE SODIUM	10049510	3
VAGINAL ABSCESS	10049573	3
QUADRIPARESIS	10049680	3
BRADYARRHYTHMIA	10049765	3
CARDIO-RESPIRATORY DISTRESS	10049874	3
NEPHROSTOMY	10050001	3
SPINAL EPIDURAL HAEMATOMA	10050162	3
SUBCLAVIAN ARTERY STENOSIS	10050180	3
GROIN ABSCESS	10050269	3
5-HYDROXYINDOLACETIC ACID	10050342	3
FEAR OF EATING	10050366	3
SKIN ULCER HAEMORRHAGE	10050377	3
ELECTROCARDIOGRAM T WAVE NORMAL	10050381	3
HAEMATOSALPINX	10050468	3
VULVA CYST	10050475	3
TRACTION	10050498	3
ECHOVIRUS TEST	10050680	3
VITAMIN A	10050712	3
BETA 2 MICROGLOBULIN	10050745	3
SYNOVIAL FLUID WHITE BLOOD CELLS POSITIVE	10050769	3
TRANSFERRIN SATURATION INCREASED	10050771	3
TRACHEAL STENOSIS	10050816	3
POSTOPERATIVE THROMBOSIS	10050902	3
MYELOCYTE COUNT	10050966	3
PLASMA CELL COUNT	10051006	3
GASTRIC VARICES	10051012	3
CATHETER SITE HAEMORRHAGE	10051099	3
FOETAL HEART RATE	10051137	3
ELECTROCARDIOGRAM T WAVE AMPLITUDE INCREASED	10051159	3
SWOLLEN TEAR DUCT	10051209	3
B-LYMPHOCYTE COUNT DECREASED	10051313	3
BILE DUCT STENOSIS	10051341	3

BILE OUTPUT ABNORMAL	10051344	3
CARBOHYDRATE ANTIGEN 19-9 INCREASED	10051418	3
ENTEROCUTANEOUS FISTULA	10051425	3
PHLEBOLITH	10051474	3
STRAWBERRY TONGUE	10051495	3
COMPLEMENT FACTOR C2	10051556	3
HAEMATOMA INFECTION	10051564	3
PULMONARY SEPSIS	10051739	3
CYANOPSIA	10051819	3
CITROBACTER INFECTION	10051904	3
TRACHEAL PAIN	10051955	3
METABOLIC SYNDROME	10052066	3
EAR CANAL ERYTHEMA	10052135	3
TYMPANIC MEMBRANE HYPERAEMIA	10052154	3
INCLUSION BODY MYOSITIS	10066407	3
HAEMATOLOGICAL MALIGNANCY	10066476	3
WEANING FAILURE	10066829	3
PAIN THRESHOLD DECREASED	10066956	3
ENDOSCOPIC ULTRASOUND ABNORMAL	10067020	3
EAR INFECTION BACTERIAL	10067046	3
CYTOTOXIC OEDEMA	10067276	3
URINARY CASTS ABSENT	10067533	3
SYSTEMIC LUPUS ERYTHEMATOSUS DISEASE ACTIVITY INDEX A	10067659	3
SPASMODIC DYSPHONIA	10067672	3
VASCULAR GRAFT	10067740	3
PUDENDAL CANAL SYNDROME	10067773	3
BABINSKI REFLEX TEST	10067804	3
KERATOMILEUSIS	10067903	3
MICROBIOLOGY TEST NORMAL	10067935	3
RENAL CELL CARCINOMA	10067946	3
OROMANDIBULAR DYSTONIA	10067954	3
RENAL FUSION ANOMALY	10068033	3
FEMORAL ARTERY EMBOLISM	10068365	3
SUPPORTIVE CARE	10068369	3
CERVIX INFLAMMATION	10068402	3
VENOUS OXYGEN SATURATION ABNORMAL	10068428	3
VENOUS OXYGEN SATURATION NORMAL	10068474	3
VENOUS RECANALISATION	10068605	3
EAR INFECTION FUNGAL	10068630	3
PINEAL GLAND CYST	10068650	3
RESPIRATORY FATIGUE	10068733	3
DECIDUAL CAST	10068735	3
VON WILLEBRAND'S FACTOR ACTIVITY NORMAL	10068984	3

HEART SOUNDS	10069168	3
PRODUCT LABEL ISSUE	10069289	3
PRODUCT PACKAGING QUANTITY ISSUE	10069299	3
PRODUCT MEASURED POTENCY ISSUE	10069325	3
DECREASED EYE CONTACT	10069633	3
DEVICE DIFFICULT TO USE	10069853	3
DEVICE PHYSICAL PROPERTY ISSUE	10069880	3
BACILLUS TEST POSITIVE	10069961	3
LACTOBACILLUS TEST POSITIVE	10070000	3
STREPTOBACILLUS TEST POSITIVE	10070054	3
SERRATIA TEST POSITIVE	10070128	3
BRUCELLA TEST	10070281	3
AUTOIMMUNE NEUROPATHY	10070439	3
VACCINE VIRUS SHEDDING	10070583	3
DEVICE INFUSION ISSUE	10070617	3
MUSCLE CONTUSION	10070757	3
UPPER RESPIRATORY TRACT IRRITATION	10070841	3
INFECTION REACTIVATION	10070891	3
JUVENILE MYOCLONIC EPILEPSY	10071082	3
BREAST SCAN	10071224	3
AORTIC STENT INSERTION	10071256	3
HEPATITIS B CORE ANTIBODY POSITIVE	10071344	3
METASTATIC LYMPHOMA	10071541	3
RETROPERITONEAL MASS	10071732	3
RADIAL NERVE COMPRESSION	10071930	3
DIALYSIS RELATED COMPLICATION	10071946	3
FRACTURE PAIN	10072132	3
JANUS KINASE 2 MUTATION	10072206	3
WRITER'S CRAMP	10072249	3
PSYCHOGENIC MOVEMENT DISORDER	10072376	3
SUBSTANCE-INDUCED PSYCHOTIC DISORDER	10072388	3
DROPPED HEAD SYNDROME	10072442	3
HYDROGEN BREATH TEST NORMAL	10072484	3
MONONUCLEAR CELL COUNT	10072854	3
UNINTENTIONAL MEDICAL DEVICE REMOVAL	10072931	3
PROTEIN DEFICIENCY	10072956	3
SOMATOTROPIN STIMULATION TEST	10073222	3
ALVEOLAR LUNG DISEASE	10073344	3
POST PROCEDURAL CONTUSION	10073353	3
URINE OSMOLARITY NORMAL	10073410	3
GENITAL PARAESTHESIA	10073420	3
INTERLEUKIN-2 RECEPTOR ASSAY	10073452	3
HAEMOPHOBIA	10073458	3

OPTICAL COHERENCE TOMOGRAPHY NORMAL	10073560	3
INTENTIONAL UNDERDOSE	10073954	3
FORCED VITAL CAPACITY	10074072	3
CIRCUMSTANCE OR INFORMATION CAPABLE OF LEADING TO [illegible]	10074266	3
CARDIOPULMONARY EXERCISE TEST NORMAL	10074358	3
GASTROINTESTINAL POLYP HAEMORRHAGE	10074437	3
APPENDICOLITH	10074458	3
STOMA SITE HAEMORRHAGE	10074508	3
ANORECTAL SWELLING	10074526	3
BEHAVIOURAL THERAPY	10074786	3
DISEASE SUSCEPTIBILITY	10074870	3
GRANULOMATOUS DERMATITIS	10074954	3
HYPERFERRITINAEMIA	10075046	3
ANTI-MUELLERIAN HORMONE LEVEL DECREASED	10075158	3
MUCOSAL PAIN	10075187	3
PERINEAL RASH	10075364	3
DISABILITY ASSESSMENT SCALE	10075368	3
LUMBOSACRAL PLEXOPATHY	10075478	3
ANAL HYPOAESTHESIA	10075521	3
DRY AGE-RELATED MACULAR DEGENERATION	10075567	3
CD30 EXPRESSION	10075644	3
ADMINISTRATION SITE MOVEMENT IMPAIRMENT	10075955	3
PERIODIC ACID SCHIFF STAIN	10076035	3
MEDICAL DEVICE SITE PAIN	10076133	3
VACCINATION SITE ATROPHY	10076155	3
VACCINATION SITE CYST	10076158	3
CSF VOLUME	10076216	3
ANTI FACTOR V ANTIBODY	10076271	3
OPTIC NERVE COMPRESSION	10076302	3
CLINOMANIA	10076760	3
ANTI FACTOR VIII ANTIBODY INCREASED	10076777	3
JUGULAR VEIN OCCLUSION	10076835	3
DOSE CALCULATION ERROR	10076874	3
JOINT DEBRIDEMENT	10076971	3
ANTIENDOMYSIAL ANTIBODY TEST	10076977	3
VASCULAR STENT OCCLUSION	10077143	3
INTENSIVE CARE UNIT ACQUIRED WEAKNESS	10077255	3
HEREDITARY MOTOR AND SENSORY NEUROPATHY	10077306	3
HEPATOBILIARY SCAN NORMAL	10077444	3
IMPAIRED ABILITY TO USE MACHINERY	10077474	3
OROPHARYNGEAL COBBLE STONE MUCOSA	10077523	3
MEDICAL PROCEDURE	10077673	3
PSEUDOMONAS TEST	10077708	3

PERINEAL DISORDER	10077853	3
EOSINOPHILIC GRANULOMATOSIS WITH POLYANGIITIS	10078117	3
GENITAL ULCER SYNDROME	10078121	3
RETAINED PLACENTA OPERATION	10078243	3
TONSILLAR EXUDATE	10078257	3
INCORRECT DISPOSAL OF PRODUCT	10078390	3
SKIN CULTURE POSITIVE	10078402	3
TUMEFACTIVE MULTIPLE SCLEROSIS	10078556	3
INAPPROPRIATE RELEASE OF PRODUCT FOR DISTRIBUTION	10078608	3
HERPES VIRUS TEST ABNORMAL	10078969	3
COGNITIVE LINGUISTIC DEFICIT	10078970	3
WALL MOTION SCORE INDEX NORMAL	10079015	3
SUBCLAVIAN VEIN OCCLUSION	10079164	3
ADMINISTRATION SITE INDENTATION	10079278	3
HAIR GROWTH RATE ABNORMAL	10079335	3
ACHENBACH SYNDROME	10079562	3
DEPENDENCE ON OXYGEN THERAPY	10079637	3
DROOPING SHOULDER SYNDROME	10079746	3
SALMONELLA TEST	10079854	3
ALCOHOL USE DISORDER	10080021	3
SKIN LESION REMOVAL	10080131	3
FEAR-RELATED AVOIDANCE OF ACTIVITIES	10080136	3
PRIMARY BILIARY CHOLANGITIS	10080429	3
DRUG DOSE OMISSION BY DEVICE	10080648	3
TERM BABY	10080681	3
VASCULAR DEVICE INFECTION	10080714	3
TISSUE IRRITATION	10080906	3
ARTIFICIAL INSEMINATION	10080948	3
NECK DISSECTION	10081204	3
STEM CELL THERAPY	10081224	3
VESSEL HARVESTING	10081419	3
ELECTROCARDIOGRAM PR SEGMENT ELEVATION	10081494	3
GASTROINTESTINAL SCARRING	10081656	3
CRAMP-FASCICULATION SYNDROME	10081974	3
TARGET SKIN LESION	10081998	3
VOGT-KOYANAGI-HARADA DISEASE	10082001	3
GASTROINTESTINAL DECOMPRESSION	10082064	3
HEART RATE VARIABILITY INCREASED	10082110	3
INFANT IRRITABILITY	10082189	3
CANCER FATIGUE	10082239	3
INTERNAL CAROTID ARTERY DEFORMITY	10082308	3
OSTOMY BAG PLACEMENT	10082415	3
BREAST SCAN ABNORMAL	10082620	3

PERIORBITAL IRRITATION	10082774	3
MUSCLE STRENGTH NORMAL	10082796	3
MONOCLONAL IMMUNOGLOBULIN INCREASED	10082825	3
CHRONIC ACTIVE EPSTEIN-BARR VIRUS INFECTION	10082848	3
FEMALE REPRODUCTIVE TRACT DISORDER	10082971	3
GASTROINTESTINAL TRACT BIOPSY	10082977	3
FLUORESCENCE ANGIOGRAM NORMAL	10083088	3
MAGNETIC RESONANCE IMAGING RENAL	10083131	3
VACUUM ASPIRATION	10083276	3
RETICULAR CELL COUNT	10083485	3
BLOOD BETA-D-GLUCAN NEGATIVE	10083489	3
FOREIGN BODY INGESTION	10083512	3
IRIS DISCOLOURATION	10083516	3
PRECANCEROUS LESION OF DIGESTIVE TRACT	10083666	3
ALPHA-1 ANTITRYPSIN DEFICIENCY	10083869	3
PLEURAL FLUID ANALYSIS ABNORMAL	10083928	3
REVASCULARISATION PROCEDURE	10084091	3
AXONAL AND DEMYELINATING POLYNEUROPATHY	10084153	3
LOSS OF THERAPEUTIC RESPONSE	10084221	3
OPTIC PERINEURITIS	10084264	3
FOETAL CARDIAC ARREST	10084280	3
GARDNERELLA TEST NEGATIVE	10084362	3
PRODUCT ADMINISTERED BY WRONG PERSON	10084371	3
MALARIA ANTIGEN TEST	10084385	3
AEROCOCCUS URINAE INFECTION	10084826	3
NOROVIRUS INFECTION	10085018	3
COMPUTERISED TOMOGRAM OF GALLBLADDER	10085278	3
URINARY OCCULT BLOOD POSITIVE	10052287	3
MYOCARDIAL BRIDGING	10052289	3
POSTICTAL PARALYSIS	10052469	3
ABDOMINAL REBOUND TENDERNESS	10052489	3
TINEL'S SIGN	10052492	3
OESOPHAGEAL MOTILITY TEST	10053061	3
POST PROCEDURAL DRAINAGE	10053175	3
BIOPSY SMALL INTESTINE	10053281	3
BIOPSY SMALL INTESTINE NORMAL	10053282	3
EYE IRRIGATION	10053300	3
SPINAL CORD OPERATION	10053348	3
BLOOD PRESSURE ORTHOSTATIC NORMAL	10053353	3
BLOOD PRESSURE ORTHOSTATIC DECREASED	10053356	3
THERMOMETRY	10053359	3
BRAIN NATRIURETIC PEPTIDE DECREASED	10053407	3
APNOEA TEST	10053464	3

SURGICAL STAPLING	10053465	3
GROUP B STREPTOCOCCUS NEONATAL SEPSIS	10053588	3
CEREBELLAR ARTERY OCCLUSION	10053633	3
ACQUIRED HAEMOPHILIA	10053745	3
GROWTH RETARDATION	10053759	3
EOSINOPHILIC CELLULITIS	10053776	3
BLOOD TESTOSTERONE FREE	10053787	3
CSF WHITE BLOOD CELL COUNT DECREASED	10053806	3
GIANOTTI-CROSTI SYNDROME	10053842	3
RADIAL PULSE DECREASED	10053903	3
RADIAL PULSE INCREASED	10053904	3
OESTROGEN RECEPTOR ASSAY	10054053	3
LARYNGEAL DISCOMFORT	10054115	3
BREAST PROSTHESIS REMOVAL	10054831	3
CORNEAL TRANSPLANT	10054846	3
INFLAMMATION OF WOUND	10054923	3
PELVIC HAEMATOMA	10054974	3
URINE PHOSPHORUS NORMAL	10055066	3
VARICES OESOPHAGEAL	10056091	3
FEMALE STERILISATION	10056199	3
ADHESIOLYSIS	10056269	3
EMPHYSEMATOUS CYSTITIS	10056351	3
PARKINSONIAN REST TREMOR	10056437	3
CORNEAL IRRITATION	10056476	3
HEPATIC INFECTION	10056522	3
URINE BILIRUBIN DECREASED	10056614	3
BRONCHIAL OEDEMA	10056695	3
VARICOPHLEBITIS	10056717	3
OTIC EXAMINATION NORMAL	10056833	3
DEVICE FAILURE	10056871	3
ALCOHOLIC PANCREATITIS	10056977	3
URINE HOMOCYSTINE	10057159	3
SCLERAL OEDEMA	10057431	3
TONSILLAR HAEMORRHAGE	10057450	3
TONSILLAR CYST	10057451	3
CARDIAC PROCEDURE COMPLICATION	10057461	3
PERIPHERAL ARTERY ANEURYSM	10057521	3
SPINAL MYELOGRAM NORMAL	10057564	3
FRACTURE REDUCTION	10057609	3
PERIPHERAL NERVE DECOMPRESSION	10057610	3
COLON INJURY	10057669	3
TRANSPLANT	10057677	3
JOINT ARTHROPLASTY	10057681	3

UPPER RESPIRATORY TRACT INFECTION BACTERIAL	10057868	3
HEAD LAG	10057870	3
CHRONIC CUTANEOUS LUPUS ERYTHEMATOSUS	10057929	3
BLADDER MASS	10058320	3
ANTI-PLATELET ANTIBODY POSITIVE	10058529	3
SEROSITIS	10058556	3
LARYNX IRRITATION	10058670	3
VITAMIN K	10058768	3
VASODILATION PROCEDURE	10058794	3
PULMONARY NECROSIS	10058824	3
ENTERITIS INFECTIOUS	10058839	3
BACTEROIDES BACTERAEMIA	10058853	3
CYTOMEGALOVIRUS VIRAEMIA	10058854	3
VIRAEMIA	10058874	3
GENERAL PHYSICAL CONDITION NORMAL	10058910	3
APPLICATION SITE DISCOLOURATION	10059005	3
DIALYSIS DEVICE INSERTION	10059015	3
VENTRICULAR DYSFUNCTION	10059056	3
SUTURE RELATED COMPLICATION	10059059	3
STOMA SITE RASH	10059071	3
STOMA SITE IRRITATION	10059072	3
CEREBRAL VASOCONSTRICTION	10059109	3
BAND NEUTROPHIL PERCENTAGE INCREASED	10059129	3
VENTRICULAR DYSKINESIA	10059162	3
OXYGEN CONSUMPTION INCREASED	10059168	3
ANOGENITAL WARTS	10059313	3
MITOGEN STIMULATION TEST	10059463	3
INTRACRANIAL HAEMATOMA	10059491	3
NON-SMALL CELL LUNG CANCER METASTATIC	10059515	3
METHYLMALONIC ACIDURIA	10059521	3
SYNOVIAL FLUID ANALYSIS	10059534	3
BETA GLOBULIN	10059561	3
URINE BARBITURATES	10059578	3
PHLEBOTOMY	10059648	3
SEX HORMONE BINDING GLOBULIN	10059654	3
SPUTUM RETENTION	10059685	3
BLOOD HEAVY METAL TEST	10059692	3
GLUCOSE-6-PHOSPHATE DEHYDROGENASE	10059900	3
RETICULOCYTE PERCENTAGE NORMAL	10059918	3
RED BLOOD CELL MICROCYTES	10060122	3
ANTINEUTROPHIL CYTOPLASMIC ANTIBODY INCREASED	10060138	3
PULMONARY ARTERIAL PRESSURE	10060196	3
ANTIDEPRESSANT DRUG LEVEL	10060312	3

MESENTERIC HAEMORRHAGE	10060717	3
DIABETIC FOOT	10060734	3
PLASMINOGEN	10060824	3
POST LUMBAR PUNCTURE SYNDROME	10060854	3
SALIVARY GLAND MUCOCOELE	10060870	3
ABDOMINAL SYMPTOM	10060926	3
ANAESTHETIC COMPLICATION	10060938	3
ARTERIAL HAEMORRHAGE	10060964	3
CHLAMYDIAL INFECTION	10061041	3
COMPLEMENT FACTOR DECREASED	10061048	3
COMPLEMENT FACTOR INCREASED	10061049	3
CONGENITAL UTERINE ANOMALY	10061079	3
COOMBS TEST NEGATIVE	10061089	3
DIABETIC COMPLICATION	10061104	3
DNA ANTIBODY POSITIVE	10061110	3
EOSINOPHIL COUNT ABNORMAL	10061125	3
MALOCCLUSION	10061274	3
MINERAL DEFICIENCY	10061291	3
NASAL MUCOSAL DISORDER	10061305	3
PERIPHERAL NERVE OPERATION	10061341	3
PERITONEAL DISORDER	10061343	3
CEREBRAL CYST	10061445	3
CENTRAL NERVOUS SYSTEM FUNCTION TEST NORMAL	10061464	3
POSTOPERATIVE WOUND COMPLICATION	10061468	3
SCAN MYOCARDIAL PERFUSION ABNORMAL	10061501	3
SERRATIA INFECTION	10061512	3
SENSORY LEVEL	10061558	3
X-RAY GASTROINTESTINAL TRACT	10061583	3
ADRENALECTOMY	10061622	3
ARTHRODESIS	10061683	3
BLOOD HEAVY METAL NORMAL	10061719	3
CARDIAC ELECTROPHYSIOLOGIC STUDY ABNORMAL	10061808	3
DIABETIC NEPHROPATHY	10061835	3
ELECTROPHORESIS NORMAL	10061836	3
FAT NECROSIS	10061857	3
ENDOSCOPY GASTROINTESTINAL ABNORMAL	10061860	3
NOSE DEFORMITY	10061875	3
PANCREATIC NEOPLASM	10061902	3
CRANIAL NERVE PARALYSIS	10061908	3
LYMPH NODES SCAN ABNORMAL	10061949	3
GASTRIC BYPASS	10061966	3
LASER THERAPY	10062035	3
LEUKOPLAKIA	10062037	3

LYMPHOCYTIC INFILTRATION	10062049	3
MANIPULATION	10062053	3
RED BLOOD CELL ELLIPTOCYTES PRESENT	10062055	3
PERICHONDRITIS	10062067	3
PERITONITIS BACTERIAL	10062070	3
STREPTOBACILLUS INFECTION	10062118	3
STOMACH SCAN	10062144	3
SERONEGATIVE ARTHRITIS	10062164	3
CATECHOLAMINES URINE	10062277	3
HABITUAL ABORTION	10062935	3
ANTI-GLOMERULAR BASEMENT MEMBRANE ANTIBODY	10063038	3
BRONCHOALVEOLAR LAVAGE NORMAL	10063077	3
BASILAR ARTERY THROMBOSIS	10063093	3
CHORIORETINOPATHY	10063118	3
AMPHETAMINES NEGATIVE	10063223	3
AMINO ACID LEVEL INCREASED	10063259	3
HISTAMINE NORMAL	10063303	3
ARTERIOSCLEROTIC RETINOPATHY	10063452	3
MEDICATION DILUTION	10063482	3
STEM CELL TRANSPLANT	10063581	3
EXPOSURE TO NOISE	10063602	3
AVERSION	10063659	3
PELVIC HAEMORRHAGE	10063678	3
PHOTOTHERAPY	10063925	3
VASCULAR STENT THROMBOSIS	10063934	3
NIPPLE INFLAMMATION	10064043	3
PASSIVE SMOKING	10064066	3
FAMILIAL RISK FACTOR	10064117	3
BETA 2 GLOBULIN	10064139	3
BONE FRAGMENTATION	10064211	3
SMEAR VAGINAL ABNORMAL	10064247	3
SPLENIC GRANULOMA	10064249	3
OESOPHAGEAL OEDEMA	10064342	3
COMPLICATION OF DEVICE INSERTION	10064538	3
TOTAL BILE ACIDS INCREASED	10064558	3
PLACENTAL INFARCTION	10064620	3
DACTYLITIS	10064769	3
ASTHMATIC CRISIS	10064823	3
WAIST CIRCUMFERENCE INCREASED	10064863	3
CERVICOGENIC HEADACHE	10064888	3
HIGH FREQUENCY ABLATION	10064979	3
CYSTITIS BACTERIAL	10065198	3
ORAL VIRAL INFECTION	10065234	3

HEPATIC CALCIFICATION	10065274	3
SPONTANEOUS HAEMATOMA	10065304	3
POST PROCEDURAL HYPOTHYROIDISM	10065306	3
MULTIFOCAL MOTOR NEUROPATHY	10065579	3
DERMATILLOMANIA	10065701	3
BIOPSY PLACENTA	10065916	3
AUTOIMMUNE INNER EAR DISEASE	10065996	3
VACCINATION SITE PALLOR	10066045	3
CAROTID ARTERY STENT INSERTION	10066102	3
AVULSION FRACTURE	10066184	3
VISCERAL CONGESTION	10066397	3
CARDIAC THERAPEUTIC PROCEDURE	10085366	3
INAPPROPRIATE SCHEDULE OF PRODUCT DISCONTINUATION	10085426	3
BLOOD LOSS ASSESSMENT	10085463	3
SUPRACLAVICULAR FOSSA PAIN	10085665	3
INDETERMINATE INVESTIGATION RESULT	10085741	3
CAMPYLOBACTER TEST	10086167	3
HEAD IMPULSE TEST	10086191	3
SIGMOID SINUS THROMBOSIS	10087208	3
JOINT IMPINGEMENT	10087218	3
ADENOIDAL HYPERTROPHY	10001229	2
ADIPOSIS DOLOROSA	10001294	2
ADJUSTMENT DISORDER WITH DEPRESSED MOOD	10001297	2
ADRENERGIC SYNDROME	10001387	2
ALCOHOL INTERACTION	10001597	2
ALPHA 1 FOETOPROTEIN AMNIOTIC FLUID	10001775	2
ALPHA 1 GLOBULIN NORMAL	10001789	2
ALPHA 2 GLOBULIN	10001792	2
ALPHA 2 GLOBULIN INCREASED	10001796	2
ALPHA-1 ANTI-TRYPSIN	10001803	2
ANAEMIA FOLATE DEFICIENCY	10002043	2
ANAPHYLAXIS TREATMENT	10002222	2
ANENCEPHALY	10002320	2
ANGIOGRAM RETINA ABNORMAL	10002444	2
ANGIOSARCOMA	10002476	2
ANION GAP ABNORMAL	10002523	2
ANOREXIA NERVOSA	10002649	2
ANTEROGRADE AMNESIA	10002711	2
ANTICIPATORY ANXIETY	10002758	2
ANTITHROMBIN III DEFICIENCY	10002832	2
AORTIC ANEURYSM REPAIR	10002885	2
AORTIC ANEURYSM RUPTURE	10002886	2
AORTIC EMBOLUS	10002897	2

APLASIA PURE RED CELL	10002965	2
APPLICATION SITE RASH	10003054	2
ARRESTED LABOUR	10003118	2
ARTERIOVENOUS FISTULA THROMBOSIS	10003192	2
ARTERITIS	10003230	2
ARTHRITIS ENTEROPATHIC	10003253	2
ASPIRATION BONE MARROW ABNORMAL	10003506	2
ASPIRATION BURSA	10003508	2
ASPIRATION JOINT NORMAL	10003519	2
ATONIC URINARY BLADDER	10003629	2
B-CELL SMALL LYMPHOCYTIC LYMPHOMA	10003908	2
BASILAR ARTERY STENOSIS	10004163	2
BENIGN NEOPLASM OF CERVIX UTERI	10004312	2
BENIGN OVARIAN TUMOUR	10004433	2
BINGE EATING	10004716	2
BIOPSY ABDOMINAL WALL NORMAL	10004723	2
BIOPSY BRAIN NORMAL	10004743	2
BIOPSY BRONCHUS	10004747	2
BIOPSY CERVIX ABNORMAL	10004754	2
BIOPSY LUNG NORMAL	10004796	2
BIOPSY OESOPHAGUS ABNORMAL	10004809	2
BIOPSY OESOPHAGUS NORMAL	10004810	2
BIOPSY RECTUM	10004859	2
BIOPSY SCLERA ABNORMAL	10004869	2
BIPOLAR II DISORDER	10004940	2
BLADDER CATHETER REMOVAL	10005025	2
BLEEDING ANOVULATORY	10005104	2
BLEEDING TIME	10005136	2
BLEEDING VARICOSE VEIN	10005144	2
BLINDNESS CORTICAL	10005177	2
BLOOD ARSENIC NORMAL	10005343	2
BLOOD BICARBONATE ABNORMAL	10005358	2
BLOOD CAFFEINE DECREASED	10005383	2
BLOOD CHROMIUM INCREASED	10005435	2
BLOOD CORTICOTROPHIN ABNORMAL	10005451	2
BLOOD CORTICOTROPHIN INCREASED	10005453	2
BLOOD FIBRINOGEN ABNORMAL	10005518	2
BLOOD FOLATE ABNORMAL	10005525	2
BLOOD INSULIN NORMAL	10005615	2
BLOOD OESTROGEN ABNORMAL	10005685	2
BLOOD PRESSURE SYSTOLIC NORMAL	10005764	2
BLOOD PROLACTIN ABNORMAL	10005778	2
BLOOD THROMBOPLASTIN DECREASED	10005826	2

BLOOD TRIGLYCERIDES DECREASED	10005838	2
BLOOD ZINC ABNORMAL	10005865	2
BONE GRAFT	10005971	2
BONE NEOPLASM	10005995	2
BREAST CANCER IN SITU	10006189	2
BREAST FIBROSIS	10006253	2
BRONCHOGRAM ABNORMAL	10006466	2
BUNDLE BRANCH BLOCK BILATERAL	10006579	2
CARBOXYHAEMOGLOBIN NORMAL	10007245	2
CARDIAC AMYLOIDOSIS	10007509	2
CARDIOVASCULAR FUNCTION TEST NORMAL	10007652	2
CAREGIVER	10007664	2
CATARRH	10007774	2
CELLULITIS ORBITAL	10007918	2
CENTRAL NERVOUS SYSTEM LYMPHOMA	10007953	2
CEREBELLAR ATAXIA	10008025	2
CERULOPLASMIN NORMAL	10008221	2
CERVICAL INCOMPETENCE	10008267	2
CERVICITIS	10008323	2
CERVICOBRACHIAL SYNDROME	10008334	2
CERVIX NEOPLASM	10008354	2
CHOLANGITIS ACUTE	10008605	2
CHOLESTEATOMA	10008642	2
CHORDAE TENDINAE RUPTURE	10008745	2
CHOROIDITIS	10008792	2
CHROMOSOME ANALYSIS ABNORMAL	10008817	2
CHRONIC HEPATITIS C	10008912	2
CLEFT PALATE	10009269	2
COAGULATION FACTOR INCREASED	10009743	2
COAGULATION FACTOR IX LEVEL	10009744	2
COAGULATION FACTOR VII LEVEL	10009759	2
COAGULATION TIME ABNORMAL	10009791	2
COAGULATION TIME SHORTENED	10009800	2
COELIAC ARTERY COMPRESSION SYNDROME	10009838	2
COLECTOMY TOTAL	10009879	2
COLOSTOMY CLOSURE	10010045	2
COMBINED IMMUNODEFICIENCY	10010099	2
CONFABULATION	10010297	2
CONGENITAL ANOMALY	10010356	2
CONVALESCENT	10010888	2
COOMBS NEGATIVE HAEMOLYTIC ANAEMIA	10010940	2
CORNEAL DYSTROPHY	10011005	2
CORNEAL EPITHELIUM DEFECT	10011010	2

CORNEAL SCAR	10011044	2
CORONARY ARTERY EMBOLISM	10011084	2
CORONARY OSTIAL STENOSIS	10011105	2
CORTISOL ABNORMAL	10011196	2
CORTISOL FREE URINE	10011199	2
CRANIAL NERVE PALSIES MULTIPLE	10011314	2
CREATINE URINE INCREASED	10011356	2
CREST SYNDROME	10011380	2
CROSSMATCH	10011412	2
CRYOGLOBULINAEMIA	10011474	2
CSF CULTURE POSITIVE	10011529	2
CSF GLUCOSE ABNORMAL	10011536	2
CSF NEUTROPHIL COUNT	10011555	2
CSF PROTEIN ABNORMAL	10011573	2
CSF PROTEIN DECREASED	10011574	2
CULTURE CERVIX	10011621	2
CULTURE CERVIX NEGATIVE	10011622	2
CUTANEOUS T-CELL LYMPHOMA	10011677	2
CYSTIC FIBROSIS	10011762	2
CYSTITIS ESCHERICHIA	10011790	2
CYSTOCELE	10011803	2
DEJA VU	10012177	2
DERMATITIS HERPETIFORMIS	10012468	2
DERMOID CYST	10012522	2
DIABETIC HYPEROSMOLAR COMA	10012669	2
DIAPHRAGMATIC HERNIA	10012713	2
DIARRHOEA INFECTIOUS	10012742	2
DILATATION ATRIAL	10013002	2
DIRECTIONAL DOPPLER FLOW TESTS ABNORMAL	10013048	2
DRUG LEVEL INCREASED	10013722	2
DRUG WITHDRAWAL SYNDROME	10013754	2
DUMPING SYNDROME	10013810	2
DUODENAL ULCER HAEMORRHAGE	10013839	2
EARLY RETIREMENT	10014055	2
ECZEMA ASTEATOTIC	10014190	2
ECZEMA INFECTED	10014199	2
EJECTION FRACTION ABNORMAL	10014331	2
ELDERLY	10014348	2
ELECTROPHORESIS ABNORMAL	10014459	2
ELECTROPHORESIS PROTEIN ABNORMAL	10014468	2
ENAMEL ANOMALY	10014576	2
ENDOMETRIAL ADENOCARCINOMA	10014720	2
ENDOMETRIAL CANCER STAGE I	10014738	2

ENLARGED CLITORIS	10014838	2
EOSINOPHILIC MYOCARDITIS	10014961	2
EOSINOPHILIC PNEUMONIA	10014962	2
EPIDURAL ANAESTHESIA	10015011	2
EPILEPTIC AURA	10015049	2
EPISIOTOMY	10015087	2
ERYTHRODERMIC PSORIASIS	10015278	2
EVACUATION OF RETAINED PRODUCTS OF CONCEPTION	10015550	2
EWING'S SARCOMA	10015560	2
EXERCISE ADEQUATE	10015636	2
EXTERNAL EAR CELLULITIS	10015729	2
EYE EXCISION	10015923	2
EYE INFECTION STAPHYLOCOCCAL	10015937	2
FACTOR VIII DEFICIENCY	10016080	2
FALSE LABOUR	10016194	2
FAMILIAL MEDITERRANEAN FEVER	10016207	2
FERTILITY INCREASED	10016467	2
FIBRIN	10016574	2
FIBRIN DEGRADATION PRODUCTS NORMAL	10016589	2
FINGER AMPUTATION	10016678	2
FLASHBACK	10016754	2
FOETAL ARRHYTHMIA	10016847	2
FOLATE DEFICIENCY	10016880	2
FORCED EXPIRATORY VOLUME DECREASED	10016987	2
FOREIGN BODY IN EYE	10017012	2
GAIT SPASTIC	10017585	2
GASTRIC POLYPS	10017817	2
GASTRIC ULCER PERFORATION	10017835	2
GASTROINTESTINAL FISTULA	10017877	2
GASTROINTESTINAL ARTERIOVENOUS MALFORMATION	10017932	2
GIANT PAPILLARY CONJUNCTIVITIS	10018258	2
GLIOBLASTOMA MULTIFORME	10018337	2
GLOSSECTOMY	10018383	2
GLYCOSURIA	10018473	2
GRANULOMA SKIN	10018698	2
GRANULOMATOUS LIVER DISEASE	10018704	2
GRAVITATIONAL OEDEMA	10018713	2
GRIEF REACTION	10018724	2
GUN SHOT WOUND	10018794	2
HAEMOGLOBINURIA	10018906	2
HAEMOGLOBIN URINE ABSENT	10018907	2
HAEMORRHAGIC CEREBRAL INFARCTION	10019005	2
HALLUCINATION, TACTILE	10019074	2

HEART TRANSPLANT	10019314	2
HEPATITIS A ANTIBODY NORMAL	10019724	2
HEPATITIS A ANTIBODY POSITIVE	10019725	2
HEPATITIS C RNA	10019748	2
HEPATITIS VIRAL	10019799	2
HEPATOTOXICITY	10019851	2
HERNIA HIATUS REPAIR	10019915	2
HERPES SIMPLEX ENCEPHALITIS	10019953	2
HIRSUTISM	10020112	2
HYPERALDOSTERONISM	10020571	2
HYPERGAMMAGLOBULINAEMIA BENIGN MONOCLONAL	10020631	2
HYPERLACTACIDAEMIA	10020660	2
HYPERMAGNESAEMIA	10020669	2
HYPERPARATHYROIDISM SECONDARY	10020708	2
HYPERPHOSPHATAEMIA	10020711	2
HYPERPLASIA ADRENAL	10020719	2
HYPERTRICHOSIS	10020864	2
HYPHAEMA	10020923	2
HYPOCOAGULABLE STATE	10020973	2
HYPONATRAEMIC SYNDROME	10021037	2
HYPOTRICHOSIS	10021126	2
ICHTHYOSIS	10021198	2
IDIOPATHIC PULMONARY FIBROSIS	10021240	2
IMMUNOGLOBULINS ABNORMAL	10021497	2
INADEQUATE DIET	10021577	2
INCARCERATED HERNIA	10021610	2
INCISIONAL HERNIA	10021619	2
INCISIONAL HERNIA REPAIR	10021623	2
INJECTION SITE FIBROSIS	10022064	2
INJECTION SITE HYPERTROPHY	10022072	2
INJECTION SITE NECROSIS	10022082	2
INSPIRATORY CAPACITY	10022445	2
INSTILLATION SITE PAIN	10022459	2
INSULIN C-PEPTIDE DECREASED	10022476	2
INSULIN RESISTANT DIABETES	10022491	2
INTERFERON GAMMA NORMAL	10022538	2
INTERVERTEBRAL DISC DISPLACEMENT	10022636	2
INTESTINAL INFARCTION	10022657	2
INTRAOCULAR PRESSURE DECREASED	10022804	2
IRIDOTOMY	10022944	2
JAUNDICE CHOLESTATIC	10023129	2
JAW CYST	10023146	2
KARYOTYPE ANALYSIS	10023302	2

KERATOCONUS	10023353	2
KIDNEY SMALL	10023435	2
KYPHOSCOLIOSIS	10023506	2
LACRIMAL GLAND ENLARGEMENT	10023632	2
LACRIMATION DISORDER	10023643	2
LAPAROSCOPY ABNORMAL	10023694	2
LARYNGITIS VIRAL	10023880	2
LE CELLS PRESENT	10024062	2
LEARNING DISABILITY	10024092	2
LERICHE SYNDROME	10024242	2
LICHEN NITIDUS	10024428	2
LID LAG	10024443	2
LINEAR IGA DISEASE	10024515	2
LIPIDS DECREASED	10024591	2
LIPOMA EXCISION	10024614	2
LIVER ABSCESS	10024652	2
LOCAL ANAESTHESIA	10024758	2
LOW DENSITY LIPOPROTEIN DECREASED	10024909	2
LYMPHOCYTIC LEUKAEMIA	10025270	2
MACROGLOSSIA	10025391	2
MACROSOMIA	10025394	2
MAGNESIUM DEFICIENCY	10025433	2
MALARIA	10025487	2
MALFORMATION VENOUS	10025532	2
MALLET FINGER	10026710	2
MAXIMAL VOLUNTARY VENTILATION	10026958	2
MEIGE'S SYNDROME	10027138	2
MENOPAUSE DELAYED	10027310	2
MENTAL DISABILITY	10027353	2
MESENTERIC ARTERIAL OCCLUSION	10027394	2
MESENTERIC ARTERY STENOSIS	10027396	2
METANEPHRINE URINE NORMAL	10027447	2
METASTATIC MALIGNANT MELANOMA	10027480	2
MONONEUROPATHY MULTIPLEX	10027918	2
MOUTH CYST	10028020	2
MULTIPLE GATED ACQUISITION SCAN	10028211	2
MUSCLE HAEMORRHAGE	10028309	2
MUSCLE HYPERTROPHY	10028311	2
MYALGIA INTERCOSTAL	10028413	2
MYCETOMA MYCOTIC	10028426	2
MYCOTOXICOSIS	10028520	2
MYELOFIBROSIS	10028537	2
MYOGLOBIN URINE	10028626	2

MYOGLOBINURIA	10028629	2
NAIL DISCOMFORT	10028693	2
NAIL HYPERTROPHY	10028700	2
NASAL ABSCESS	10028720	2
NASAL SEPTUM DISORDER	10028763	2
NASAL SEPTUM PERFORATION	10028765	2
NASAL VESTIBULITIS	10028781	2
NECROSIS ISCHAEMIC	10028862	2
NEPHROSCLEROSIS	10029159	2
NEUTROPHIL TOXIC GRANULATION PRESENT	10029378	2
NODAL OSTEOARTHRITIS	10029469	2
NON-SMALL CELL LUNG CANCER STAGE IV	10029522	2
NOREPINEPHRINE INCREASED	10029752	2
NOSOCOMIAL INFECTION	10029803	2
OESOPHAGEAL CARCINOMA	10030155	2
OESOPHAGEAL HAEMORRHAGE	10030172	2
OESOPHAGECTOMY	10030215	2
OPEN ANGLE GLAUCOMA	10030348	2
OPEN FRACTURE	10030527	2
OPTICOKINETIC NYSTAGMUS TESTS ABNORMAL	10030956	2
ORBITAL MYOSITIS	10031050	2
OSTEOMYELITIS CHRONIC	10031256	2
OSTEOSCLEROSIS	10031298	2
OTOSALPINGITIS	10033102	2
OTOTOXICITY	10033109	2
PANCREATECTOMY	10033596	2
PANCREATIC CARCINOMA METASTATIC	10033610	2
PANCREATIC ENZYME ABNORMALITY	10033619	2
PANCREATITIS HAEMORRHAGIC	10033650	2
PANOPHTHALMITIS	10033683	2
PAPILLOMA	10033713	2
PARAESTHESIA MUCOSAL	10033780	2
PARATHYROIDECTOMY	10033968	2
PARIETAL CELL ANTIBODY POSITIVE	10033996	2
PAROTIDECTOMY	10034036	2
PEPTIC ULCER HAEMORRHAGE	10034344	2
PERFORATION BILE DUCT	10034405	2
PERICORONITIS	10034504	2
PERIODONTAL DISEASE	10034536	2
PERIPHERAL NERVE NEUROSTIMULATION	10034587	2
PERIPHERAL NERVOUS SYSTEM FUNCTION TEST ABNORMAL	10034591	2
PERIPHERAL T-CELL LYMPHOMA UNSPECIFIED	10034623	2
PH URINE ABNORMAL	10034792	2

PHAEOCHROMOCYTOMA	10034800	2
PITUITARY TUMOUR	10035104	2
PLASMA VISCOSITY	10035467	2
PLASMINOGEN NORMAL	10035496	2
PLASTIC SURGERY TO THE FACE	10035509	2
PLATELET MORPHOLOGY ABNORMAL	10035538	2
PNEUMONIA CHLAMYDIAL	10035673	2
PNEUMONIA HAEMOPHILUS	10035702	2
PNEUMONITIS ASPIRATION	10035744	2
POLIOMYELITIS	10036012	2
POLYNEUROPATHY IDIOPATHIC PROGRESSIVE	10036111	2
PORENCEPHALY	10036172	2
PORPHYRIA ACUTE	10036182	2
POST POLIO SYNDROME	10036239	2
POSTERIOR CAPSULE OPACIFICATION	10036346	2
PREAURICULAR CYST	10036509	2
PRECIPITATE LABOUR	10036519	2
PREGNANCY TEST FALSE POSITIVE	10036573	2
PRESBYACUSIS	10036626	2
PROGRESSIVE SUPRANUCLEAR PALSY	10036813	2
PROLONGED PREGNANCY	10036877	2
PROPHYLAXIS	10036898	2
PROTHROMBIN LEVEL ABNORMAL	10037048	2
PROTHROMBIN LEVEL DECREASED	10037050	2
PSYCHOMOTOR RETARDATION	10037213	2
PSYCHOTHERAPY	10037247	2
PSYCHOTIC BEHAVIOUR	10037249	2
PULMONARY ARTERIAL WEDGE PRESSURE	10037325	2
PULMONARY HAEMOSIDEROSIS	10037396	2
RADIATION ASSOCIATED PAIN	10037755	2
RADIOACTIVE IODINE THERAPY	10037784	2
RADIOALLERGOSORBENT TEST	10037787	2
RASH PAPULOSQUAMOUS	10037879	2
REACTION TO FOOD ADDITIVE	10037977	2
RECTAL CANCER STAGE IV	10038051	2
RECTAL PROLAPSE REPAIR	10038078	2
RED BLOOD CELL HYPOCHROMIC MORPHOLOGY PRESENT	10038161	2
RENAL TUBULAR DISORDER	10038537	2
RENAL VASCULITIS	10038546	2
RETINAL ARTERY EMBOLISM	10038826	2
RETINAL DEGENERATION	10038845	2
RETINAL PIGMENT EPITHELIOPATHY	10038893	2
RETINITIS	10038910	2

RETRACTED NIPPLE	10038944	2
RHESUS ANTIBODIES POSITIVE	10039036	2
RHEUMATIC FEVER	10039054	2
RUBELLA IMMUNITY CONFIRMED	10039262	2
SALIVARY GLAND CALCULUS	10039394	2
SALPINGITIS	10039453	2
SCLERODERMA-LIKE REACTION	10039714	2
SEASONAL AFFECTIVE DISORDER	10039775	2
SELECTIVE IGA IMMUNODEFICIENCY	10039915	2
SENILE DEMENTIA	10039966	2
SENSORY NEUROPATHY HEREDITARY	10040037	2
SEPTIC EMBOLUS	10040067	2
SHOCK HYPOGLYCAEMIC	10040576	2
SICKLE CELL DISEASE	10040644	2
SIGMOIDOSCOPY NORMAL	10040671	2
SINUS POLYP	10040749	2
SKIN FRAGILITY	10040851	2
SKULL FRACTURED BASE	10040960	2
SLIT-LAMP TESTS ABNORMAL	10041033	2
SMALL INTESTINAL ANASTOMOSIS	10041094	2
SMALL INTESTINAL PERFORATION	10041103	2
SOCIAL ALCOHOL DRINKER	10041241	2
SOCIAL ANXIETY DISORDER	10041242	2
SPINAL CORD INJURY LUMBAR	10041555	2
SPINAL MUSCULAR ATROPHY	10041582	2
SPLENIC HAEMATOMA	10041646	2
SPLENIC INJURY	10041649	2
SPORTS INJURY	10041738	2
SUBCLAVIAN ARTERY THROMBOSIS	10042334	2
SUSPICIOUSNESS	10042635	2
SYMPTOM MASKED	10042750	2
SYSTEMIC CANDIDA	10042938	2
T-CELL LYMPHOMA	10042971	2
TACHYCARDIA PAROXYSMAL	10043079	2
TANNING	10043115	2
TARSAL TUNNEL SYNDROME	10043121	2
TENDON SHEATH INCISION	10043250	2
TERMINAL ILEITIS	10043280	2
TESTICULAR DISORDER	10043306	2
TESTICULAR PAIN	10043345	2
THROAT CANCER	10043515	2
THROMBIN TIME	10043539	2
THYMECTOMY	10043661	2

THYROID ATROPHY	10043693	2
THYROID RELEASING HORMONE CHALLENGE TEST	10043763	2
TINEA CAPITIS	10043866	2
TOOTH EROSION	10044038	2
TRACHEAL OBSTRUCTION	10044291	2
TRANCE	10044334	2
TRANSFERRIN DECREASED	10044356	2
TRANSFERRIN INCREASED	10044357	2
TRI-IODOTHYRONINE UPTAKE INCREASED	10044602	2
TRISOMY 21	10044688	2
TRISOMY 22	10044689	2
TUMOUR LYSIS SYNDROME	10045170	2
TURNER'S SYNDROME	10045181	2
UMBILICAL CORD AROUND NECK	10045447	2
UNINTENDED PREGNANCY	10045542	2
URETERIC CALCULUS REMOVAL	10046391	2
URETERIC DILATATION	10046399	2
URETERIC OBSTRUCTION	10046406	2
URINE ELECTROPHORESIS	10046635	2
URINE OSMOLARITY	10046652	2
URINE PORPHOBILINOGEN INCREASED	10046658	2
URINE SODIUM NORMAL	10046673	2
URTICARIA CONTACT	10046742	2
URTICARIA PHYSICAL	10046751	2
UTERINE NEOPLASM	10046803	2
UTERINE PROLAPSE	10046814	2
UTERINE RUPTURE	10046820	2
UTERINE TENDERNESS	10046827	2
VAGINITIS GARDNERELLA	10046957	2
VARICOSE VEIN RUPTURED	10046999	2
VASCULAR HEADACHE	10047077	2
VENA CAVA EMBOLISM	10047193	2
VENOUS PRESSURE JUGULAR INCREASED	10047240	2
VIRAL LABYRINTHITIS	10047466	2
VITAL CAPACITY NORMAL	10047583	2
VITAMIN B6 DEFICIENCY	10047617	2
VULVAL OEDEMA	10047763	2
VULVITIS	10047780	2
WALDENSTROM'S MACROGLOBULINAEMIA	10047801	2
WARM TYPE HAEMOLYTIC ANAEMIA	10047822	2
WHITE BLOOD CELL AGGLUTINATION PRESENT	10047937	2
XANTHOCHROMIA	10048213	2
XEROSIS	10048222	2

CARDIOMYOPATHY ACUTE	10048377	2
GROWTH OF EYELASHES	10048462	2
SUTURE RUPTURE	10048547	2
HYDROTHORAX	10048612	2
SKIN MACERATION	10048625	2
SUBDURAL HYGROMA	10048626	2
INFUSION SITE ERYTHEMA	10048634	2
POOR VENOUS ACCESS	10048737	2
OESOPHAGOGASTRIC FUNDOPLASTY	10048852	2
PITYRIASIS LICHENOIDES ET VARIOLIFORMIS ACUTA	10048895	2
BLADDER IRRIGATION	10048923	2
RECTAL ABSCESS	10048947	2
CYTOMEGALOVIRUS COLITIS	10048983	2
RENAL ARTERY OCCLUSION	10048988	2
HYSTEROSALPINGO-OOPHORECTOMY	10049149	2
RED BLOOD CELL AGGLUTINATION	10049190	2
BLEEDING TIME ABNORMAL	10049227	2
CARBOHYDRATE ANTIGEN 15-3	10049249	2
CARBOHYDRATE ANTIGEN 27.29	10049250	2
SHORT-BOWEL SYNDROME	10049416	2
CARDIAC PACEMAKER REPLACEMENT	10049434	2
LYMPHANGIOLEIOMYOMATOSIS	10049459	2
DEHYDROEPIANDROSTERONE INCREASED	10049482	2
BRAIN STEM AUDITORY EVOKED RESPONSE NORMAL	10049508	2
TRAUMATIC FRACTURE	10049514	2
ANTI-ERYTHROCYTE ANTIBODY	10049523	2
ATRIAL PRESSURE INCREASED	10049785	2
COLORECTOSTOMY	10049804	2
ANAL SPHINCTER ATONY	10049823	2
ABULIA	10050013	2
APPLICATION SITE NODULE	10050101	2
POSTUROGRAPHY	10050120	2
OPHTHALMOPLEGIC MIGRAINE	10050122	2
CEREBRAL HAEMORRHAGE FOETAL	10050157	2
VOCAL CORD ATROPHY	10050201	2
ANAL INFLAMMATION	10050247	2
CARDIOVASCULAR DECONDITIONING	10050257	2
TUMOUR ULCERATION	10050283	2
ELECTROCARDIOGRAM P WAVE ABNORMAL	10050384	2
SEROLOGY ABNORMAL	10050408	2
SEROLOGY POSITIVE	10050409	2
ANASTOMOTIC LEAK	10050456	2
MORBID THOUGHTS	10050464	2

SINOSCOPY	10050499	2
ANTI-ISLET CELL ANTIBODY	10050516	2
URINE CALCIUM	10050520	2
ATRIAL PRESSURE	10050641	2
LEUKONYCHIA	10050658	2
CYSTITIS-LIKE SYMPTOM	10050687	2
OESOPHAGEAL MANOMETRY	10050703	2
RED BLOOD CELL BURR CELLS PRESENT	10050761	2
METAMYELOCYTE COUNT INCREASED	10050765	2
MYELOCYTE COUNT INCREASED	10050985	2
GASTROINTESTINAL STROMAL TUMOUR	10051066	2
PRESCRIBED OVERDOSE	10051076	2
PROTEIN S DECREASED	10051120	2
ACQUIRED DYSFIBRINOGENAEMIA	10051122	2
MECONIUM IN AMNIOTIC FLUID	10051133	2
SPLENIC CYST	10051169	2
PLANTAR ERYTHEMA	10051170	2
NASAL ODOUR	10051181	2
POSTOPERATIVE ABSCESS	10051182	2
CAMPYLOBACTER INFECTION	10051226	2
PHOTODERMATOSIS	10051246	2
SPONDYLOARTHROPATHY	10051265	2
BLOOD VISCOSITY INCREASED	10051293	2
NEUROLOGICAL INFECTION	10051295	2
SOFT TISSUE HAEMORRHAGE	10051297	2
T-LYMPHOCYTE COUNT DECREASED	10051318	2
THYROGLOSSAL CYST	10051320	2
BILE OUTPUT	10051345	2
THROMBIN TIME NORMAL	10051389	2
MALIGNANT NEOPLASM PROGRESSION	10051398	2
BIOPSY CHEST WALL	10051412	2
VIRAL MYOSITIS	10051512	2
LUNG TRANSPLANT REJECTION	10051604	2
PARATHYROID GLAND ENLARGEMENT	10051653	2
PITUITARY CYST	10051687	2
METASTASES TO MENINGES	10051696	2
PLATELETPHERESIS	10051719	2
PROTEIN S INCREASED	10051735	2
PULMONARY CAVITATION	10051738	2
AORTIC BRUIT	10051832	2
EXPOSURE TO ALLERGEN	10051841	2
TONGUE CYST	10051879	2
ENDOMETRIAL ATROPHY	10051909	2

GLOMERULONEPHROPATHY	10051920	2
FEBRILE INFECTION	10051998	2
PANCREATIC ISLETS HYPERPLASIA	10052031	2
HYDROMETRA	10052096	2
OCCUPATIONAL THERAPY	10066401	2
POST PROCEDURAL SWELLING	10066415	2
ANAEMIA OF PREGNANCY	10066468	2
ACUTE POLYNEUROPATHY	10066699	2
ACUTE INTERSTITIAL PNEUMONITIS	10066728	2
SCLERAL BUCKLING SURGERY	10066785	2
MECHANICAL VENTILATION COMPLICATION	10066821	2
HUMAN HERPESVIRUS 6 INFECTION REACTIVATION	10066845	2
SURGICAL FAILURE	10066902	2
BLADDER HYDRODISTENSION	10066923	2
BOWEL PREPARATION	10066943	2
FISTULA DISCHARGE	10067143	2
CEREBELLAR EMBOLISM	10067167	2
AIRWAY BURNS	10067204	2
MINI-TRACHEOSTOMY	10067205	2
MONOCYTE PERCENTAGE ABNORMAL	10067238	2
SLIPPING RIB SYNDROME	10067349	2
INTRAOCULAR LENS IMPLANT	10067434	2
MYELOMALACIA	10067441	2
CONFUSIONAL AROUSAL	10067494	2
TRENDELENBURG POSITION	10067496	2
URINARY CASTS PRESENT	10067534	2
ACTIVATED PROTEIN C RESISTANCE TEST POSITIVE	10067648	2
END-TIDAL CO2 DECREASED	10067665	2
PARASITIC GASTROENTERITIS	10067720	2
PULMONARY AIR LEAKAGE	10067826	2
DEMENTIA WITH LEWY BODIES	10067889	2
PHOTOREFRACTIVE KERATECTOMY	10067902	2
INTERSTITIAL GRANULOMATOUS DERMATITIS	10067972	2
SWEAT TEST	10068025	2
SWEAT TEST ABNORMAL	10068027	2
GASTROINTESTINAL SURGERY	10068093	2
ENDOVENOUS ABLATION	10068094	2
VESTIBULAR NYSTAGMUS	10068101	2
METASTATIC CARCINOID TUMOUR	10068115	2
HEAT ILLNESS	10068188	2
ANTI FACTOR VIII ANTIBODY TEST	10068229	2
GASTROINTESTINAL BACTERIAL INFECTION	10068306	2
HEPATITIS B DNA INCREASED	10068379	2

DRUG DELIVERY SYSTEM MALFUNCTION	10068383	2
CAPILLARITIS	10068406	2
VENOUS OXYGEN SATURATION	10068425	2
NATURAL KILLER CELL COUNT	10068495	2
EXPOSURE TO CONTAMINATED DEVICE	10068515	2
INTRA-ABDOMINAL PRESSURE INCREASED	10068611	2
EXERTIONAL HEADACHE	10068614	2
POST PROCEDURAL CONSTIPATION	10068620	2
EMPHYSEMATOUS PYELONEPHRITIS	10068822	2
SCHIZOAFFECTIVE DISORDER BIPOLAR TYPE	10068889	2
GASTROINTESTINAL EXAMINATION NORMAL	10068976	2
VON WILLEBRAND'S FACTOR ANTIGEN NORMAL	10068982	2
AUTOIMMUNE PANCREATITIS	10069002	2
MAMMARY DUCTECTOMY	10069117	2
HYPERGEUSIA	10069147	2
PRODUCT CONTAMINATION	10069173	2
PRODUCT TASTE ABNORMAL	10069227	2
SUBRETINAL FLUID	10069356	2
VACCINATION SITE DERMATITIS	10069477	2
MAST CELL DEGRANULATION TEST	10069536	2
MONKEYPOX	10069538	2
VACCINATION SITE GRANULOMA	10069562	2
ROBUST TAKE FOLLOWING EXPOSURE TO VACCINIA VIRUS	10069576	2
DISEASE PRODROMAL STAGE	10069581	2
VACCINATION SITE NECROSIS	10069625	2
BONE MARROW RETICULIN FIBROSIS	10069678	2
THROMBOPOIETIN LEVEL ABNORMAL	10069685	2
COMA SCALE NORMAL	10069707	2
LOW CARBOHYDRATE DIET	10069751	2
ADMINISTRATION RELATED REACTION	10069773	2
PANCREATIC STENT PLACEMENT	10069809	2
EYE COMPLICATION ASSOCIATED WITH DEVICE	10069837	2
DEVICE INFORMATION OUTPUT ISSUE	10069842	2
DEVICE MATERIAL ISSUE	10069871	2
CSF VOLUME INCREASED	10069903	2
LACRIMAL HAEMORRHAGE	10069930	2
CITROBACTER TEST POSITIVE	10069963	2
MORAXELLA TEST POSITIVE	10070095	2
NEISSERIA TEST POSITIVE	10070096	2
GARDNERELLA TEST POSITIVE	10070099	2
SALMONELLA TEST POSITIVE	10070127	2
BARTONELLA TEST POSITIVE	10070157	2
EHRLICHIA TEST POSITIVE	10070195	2

HEPATITIS A VIRUS TEST POSITIVE	10070216	2
FOREIGN BODY	10070245	2
RUBIVIRUS TEST POSITIVE	10070250	2
BRUCELLA TEST NEGATIVE	10070269	2
MYCOBACTERIUM TEST POSITIVE	10070323	2
RUBULAVIRUS TEST POSITIVE	10070347	2
NOROVIRUS TEST POSITIVE	10070379	2
COXSACKIE VIRUS TEST POSITIVE	10070384	2
ASPERGILLUS TEST POSITIVE	10070448	2
CANDIDA TEST	10070453	2
IMMUNE TOLERANCE INDUCTION	10070581	2
EXTERNAL CEPHALIC VERSION	10070636	2
KNUCKLE PADS	10070650	2
LIMB ASYMMETRY	10070670	2
MALARIA ANTIBODY TEST NEGATIVE	10070880	2
OVARIAN CANCER STAGE IV	10070908	2
BONE DEFORMITY	10070918	2
REYNOLD'S SYNDROME	10070953	2
PRODUCT PHYSICAL ISSUE	10071134	2
VENTRICULAR DYSSYNCHRONY	10071186	2
BINGE DRINKING	10071238	2
PRODUCT ORIGIN UNKNOWN	10071242	2
HEPATITIS B SURFACE ANTIBODY POSITIVE	10071346	2
EXPOSURE VIA PARTNER	10071412	2
ANTI-GANGLIOSIDE ANTIBODY	10071473	2
ANTI-VGCC ANTIBODY	10071476	2
ANTI-COMPLEMENT ANTIBODY	10071477	2
IMMUNOPHENOTYPING	10071534	2
IL-2 RECEPTOR ASSAY	10071599	2
UMBILICAL CORD THROMBOSIS	10071652	2
CARBONIC ANHYDRASE GENE MUTATION ASSAY	10071964	2
SMALL SIZE PLACENTA	10072038	2
MEDICAL COUNSELLING	10072071	2
ENVIRONMENTAL EXPOSURE	10072082	2
STIFF PERSON SYNDROME	10072148	2
RENAL VASCULAR THROMBOSIS	10072226	2
CHRONIC VILLITIS OF UNKNOWN ETIOLOGY	10072271	2
NEUROMYOTONIA	10072359	2
ACTION TREMOR	10072413	2
FAECAL CALPROTECTIN NORMAL	10072480	2
HYDROGEN BREATH TEST ABNORMAL	10072485	2
ANTI-NMDA ANTIBODY NEGATIVE	10072499	2
ANTI-NMDA ANTIBODY POSITIVE	10072500	2

ANTI-VGCC ANTIBODY NEGATIVE	10072501	2
ANTI-AQUAPORIN-4 ANTIBODY POSITIVE	10072508	2
ANTI-GANGLIOSIDE ANTIBODY NEGATIVE	10072515	2
ANTI-MUSCLE SPECIFIC KINASE ANTIBODY NEGATIVE	10072521	2
ANTIRIBOSOMAL P ANTIBODY	10072533	2
VIRAL PAROTITIS	10072589	2
ORTHOPAEDIC PROCEDURE	10072627	2
POSTOPERATIVE RESPIRATORY FAILURE	10072651	2
PELVIC CYST	10072715	2
PHARYNGO-OESOPHAGEAL DIVERTICULUM	10072785	2
PREGNANCY OF UNKNOWN LOCATION	10072811	2
INTERFERON GAMMA RELEASE ASSAY POSITIVE	10072866	2
PULMONARY ENDARTERECTOMY	10072893	2
CORTICOSTEROID BINDING GLOBULIN TEST	10072932	2
BIRDSHOT CHORIORETINOPATHY	10072959	2
GASTROSTOMY TUBE REMOVAL	10072964	2
CONGENITAL SKIN DIMPLES	10072978	2
INVASIVE PAPILLARY BREAST CARCINOMA	10073098	2
FAT TISSUE DECREASED	10073170	2
GASTROINTESTINAL DILATION PROCEDURE	10073232	2
OVARIAN CLEAR CELL CARCINOMA	10073268	2
UPPER RESPIRATORY TRACT ENDOSCOPY	10073332	2
PLEOMORPHIC ADENOMA	10073372	2
OEDEMATOUS KIDNEY	10073381	2
TRANSFERRIN RECEPTOR ASSAY	10073450	2
SPLEEN CONTUSION	10073533	2
EPIDURAL HAEMORRHAGE	10073681	2
MUSCULOSKELETAL INJURY	10073713	2
LACK OF INJECTION SITE ROTATION	10073752	2
EPIDURAL CATHETER PLACEMENT	10074181	2
HERPES ZOSTER CUTANEOUS DISSEMINATED	10074297	2
OPHTHALMIC VEIN THROMBOSIS	10074349	2
DRUG PROVOCATION TEST	10074350	2
POSITIVE EXPIRATORY PRESSURE THERAPY	10074372	2
NONINFECTIVE OOPHORITIS	10074436	2
TENDON LAXITY	10074443	2
HEPATIC VASCULAR THROMBOSIS	10074494	2
SPLENIC ARTERY THROMBOSIS	10074600	2
NONINFECTIVE CONJUNCTIVITIS	10074701	2
ADMINISTRATION SITE INFLAMMATION	10074704	2
COLONIC LAVAGE	10074741	2
FOWLER'S POSITION	10074743	2
INFECTED SEROMA	10074755	2

PRODUCT PACKAGING CONFUSION	10074776	2
ORAL MUCOSA HAEMATOMA	10074779	2
THECAL SAC COMPRESSION	10074801	2
SECONDARY CEREBELLAR DEGENERATION	10074943	2
MULTIPLE DRUG THERAPY	10074950	2
RIGHT HEMISPHERE DEFICIT SYNDROME	10075037	2
ALTERED PITCH PERCEPTION	10075083	2
ADMINISTRATION SITE DISCHARGE	10075097	2
ADMINISTRATION SITE DISCOLOURATION	10075098	2
ENZYME LEVEL ABNORMAL	10075119	2
DIET FAILURE	10075213	2
FOETAL BIOPHYSICAL PROFILE SCORE	10075216	2
ECG SIGNS OF MYOCARDIAL INFARCTION	10075299	2
ELECTROCARDIOGRAM J WAVE ABNORMAL	10075347	2
REACTIVE GASTROPATHY	10075425	2
MEDICAL CANNABIS THERAPY	10075530	2
CUTANEOUS SYMPTOM	10075531	2
GOMORI METHENAMINE SILVER STAIN	10075549	2
CHANGE IN SEIZURE PRESENTATION	10075606	2
INTESTINAL SEPSIS	10075622	2
ACUTE HAEMORRHAGIC ULCERATIVE COLITIS	10075634	2
CARDIAC PACEMAKER ADJUSTMENT	10075685	2
AUTOIMMUNE DERMATITIS	10075689	2
VASCULITIC ULCER	10075714	2
AORTIC PERFORATION	10075729	2
MODIFIED RANKIN SCORE	10075864	2
MACULAR DETACHMENT	10075873	2
ADMINISTRATION SITE DISCOMFORT	10075928	2
ADMINISTRATION SITE IRRITATION	10075941	2
IDIOPATHIC ORBITAL INFLAMMATION	10075997	2
GASTROSTOMY TUBE SITE COMPLICATION	10076053	2
PLANNING TO BECOME PREGNANT	10076056	2
INFUSION SITE JOINT MOVEMENT IMPAIRMENT	10076075	2
VACCINATION SITE CALCIFICATION	10076156	2
VACCINATION SITE JOINT INFLAMMATION	10076174	2
VACCINATION SITE PLAQUE	10076187	2
DILATED PORES	10076249	2
FLUID BALANCE POSITIVE	10076267	2
VASCULAR DEVICE USER	10076273	2
ANTI-SACCHAROMYCES CEREVISIAE ANTIBODY	10076310	2
SPINAL INSTABILITY	10076339	2
DEVICE LOOSENING	10076364	2
VASCULAR COMPRESSION THERAPY	10076482	2

TIDAL VOLUME	10076535	2
MARGINAL ZONE LYMPHOMA	10076596	2
CHEST WALL HAEMATOMA	10076597	2
ATHEROSCLEROTIC PLAQUE RUPTURE	10076604	2
NECROTISING SOFT TISSUE INFECTION	10076637	2
PRIMARY COUGH HEADACHE	10076707	2
PROLAPSE	10076708	2
PELVIC OPERATION	10076776	2
CEREBRAL VASCULAR OCCLUSION	10076895	2
SYSTEMIC IMMUNE ACTIVATION	10076970	2
TUMOUR MARKER ABNORMAL	10077029	2
HEPATITIS C CORE ANTIBODY NEGATIVE	10077054	2
PENETRATING AORTIC ULCER	10077118	2
NEURITIC PLAQUES	10077174	2
ALLERGY TO SURGICAL SUTURES	10077279	2
ISCHAEMIC SKIN ULCER	10077408	2
DUST ALLERGY	10077439	2
PULMONARY RESECTION	10077488	2
VERTEBRAL ARTERY ANEURYSM	10077498	2
ORAL PIGMENTATION	10077552	2
GASTRORRHAPHY	10077591	2
BASILAR ARTERY ANEURYSM	10077607	2
PORTOSPLENOMESENTERIC VENOUS THROMBOSIS	10077623	2
MANUFACTURING ISSUE	10077796	2
PERSISTENT DEPRESSIVE DISORDER	10077804	2
C3 GLOMERULOPATHY	10077827	2
OBSTRUCTIVE NEPHROPATHY	10077862	2
METABOLIC SURGERY	10078004	2
LIMBIC ENCEPHALITIS	10078012	2
SOMATIC SYMPTOM DISORDER OF PREGNANCY	10078085	2
FLAVIVIRUS TEST NEGATIVE	10078301	2
ANTI-MYELIN-ASSOCIATED GLYCOPROTEIN ANTIBODIES POSIT	10078318	2
PERINATAL DEPRESSION	10078366	2
CISTERNOGRAM	10078378	2
GRANULOMATOUS LYMPHADENITIS	10078494	2
VASCULAR ACCESS SITE THROMBOSIS	10078675	2
TENSOR FASCIAE LATAE SYNDROME	10078698	2
LARYNGEAL TREMOR	10078751	2
CYSTIC LUNG DISEASE	10078811	2
PRIMARY FAMILIAL BRAIN CALCIFICATION	10078822	2
NONINFECTIVE MYRINGITIS	10078830	2
PERSISTENT GENITAL AROUSAL DISORDER	10078966	2
SILENT THYROIDITIS	10079012	2

GASTROINTESTINAL POLYP	10079055	2
SUSPECTED PRODUCT CONTAMINATION	10079078	2
NASOPHARYNGOSCOPY	10079094	2
UTERINE IRRITABILITY	10079224	2
RADIOLOGICALLY ISOLATED SYNDROME	10079292	2
DRUG EFFECTIVE FOR UNAPPROVED INDICATION	10079317	2
DRUG USE DISORDER	10079381	2
MISOPHONIA	10079388	2
HYPERTENSIVE END-ORGAN DAMAGE	10079496	2
BACTERIAL VULVOVAGINITIS	10079528	2
ANTI FACTOR XA ASSAY NORMAL	10079648	2
DIFFUSION-WEIGHTED BRAIN MRI ABNORMAL	10079717	2
DIFFUSION-WEIGHTED BRAIN MRI NORMAL	10079719	2
LABOUR AUGMENTATION	10079807	2
LIP COSMETIC PROCEDURE	10079830	2
HYPOSPLENISM	10079831	2
NUTRITIONAL ASSESSMENT	10079875	2
FOETAL HEART RATE INDETERMINATE	10079882	2
INTENSIVE CARE UNIT DELIRIUM	10080066	2
PARANASAL SINUS HAEMORRHAGE	10080108	2
FRANCISELLA TEST	10080178	2
RECALLED PRODUCT	10080231	2
WRONG DOSE	10080304	2
SEPTIC SCREEN	10080310	2
FUNGAL DISEASE CARRIER	10080548	2
DRUG TROUGH LEVEL	10080597	2
RELAPSING MULTIPLE SCLEROSIS	10080700	2
COUNTERFEIT PRODUCT ADMINISTERED	10080754	2
INTRAVESICAL IMMUNOTHERAPY	10080771	2
INCORRECT DOSE ADMINISTERED BY PRODUCT	10080804	2
VON WILLEBRAND'S FACTOR ANTIBODY	10080829	2
HYPERAROUSAL	10080831	2
HTLV TEST	10080854	2
PHYSICAL CAPACITY EVALUATION	10080885	2
DEHISCENCE	10080904	2
KLEBSIELLA TEST	10080943	2
TISSUE RUPTURE	10080972	2
RED BLOOD CELL SPHEROCYTES	10080980	2
DENGUE VIRUS TEST NEGATIVE	10081043	2
OZONE THERAPY	10081102	2
INTENTIONAL REMOVAL OF DRUG DELIVERY SYSTEM BY PATIE	10081134	2
GALLBLADDER RUPTURE	10081151	2
DIGESTIVE ENZYME TEST	10081157	2

DIGESTIVE ENZYME ABNORMAL	10081159	2
AIRWAY PATENCY DEVICE INSERTION	10081227	2
NEUROLOGICAL PROCEDURAL COMPLICATION	10081338	2
PRODUCT RESIDUE PRESENT	10081359	2
HELIOTROPE RASH	10081454	2
ELECTROCARDIOGRAM PR SEGMENT DEPRESSION	10081493	2
VERTEBRAL LATERAL RECESS STENOSIS	10081612	2
CHEMOKINE INCREASED	10081663	2
EYE PH TEST	10081702	2
ARTERIAL ANGIOPLASTY	10081731	2
JUGULAR VEIN EMBOLISM	10081850	2
GROWTH DISORDER	10081945	2
NEUROPROSTHESIS IMPLANTATION	10081968	2
COLD BURN	10081985	2
SPINAL STROKE	10082031	2
SIGMOID-SHAPED VENTRICULAR SEPTUM	10082038	2
GREY MATTER HETEROTOPIA	10082084	2
ACUTE FLACCID MYELITIS	10082097	2
HEART RATE VARIABILITY DECREASED	10082111	2
ACQUIRED BLASCHKOID DERMATITIS	10082113	2
INCISION SITE DISCHARGE	10082296	2
EXTERNAL VAGAL NERVE STIMULATION	10082451	2
STARVATION KETOACIDOSIS	10082528	2
TONGUE THRUST	10082545	2
PLASMINOGEN ACTIVATOR INHIBITOR TYPE 1 DEFICIENCY	10082567	2
INFUSION RELATED HYPERSENSITIVITY REACTION	10082742	2
PHRENIC NERVE INJURY	10082761	2
ACHROMOBACTER INFECTION	10082866	2
FAECAL ELASTASE TEST	10082867	2
BILIARY CATHETER INSERTION	10082920	2
COMPUTERISED TOMOGRAM PANCREAS NORMAL	10082935	2
POST PROCEDURAL ERYTHEMA	10082944	2
MUCOCUTANEOUS DISORDER	10082980	2
EYELID SCAR	10082987	2
IMMUNE-MEDIATED MYOSITIS	10083073	2
ORBITAL SPACE OCCUPYING LESION	10083097	2
URINARY TRACT CANDIDIASIS	10083162	2
CORNEAL PACHYMETRY	10083168	2
HEMIDYSAESTHESIA	10083174	2
HER2 NEGATIVE BREAST CANCER	10083232	2
FIBRONECTIN	10083290	2
FIBRONECTIN NORMAL	10083291	2
PELVIC GIRDLE PAIN	10083336	2

DRUG EFFECT LESS THAN EXPECTED	10083365	2
SUBCAPSULAR SPLENIC HAEMATOMA	10083384	2
OUT OF SPECIFICATION PRODUCT USE	10083420	2
ELECTROCARDIOGRAM RR INTERVAL	10083424	2
VULVOVAGINAL EXFOLIATION	10083435	2
BLOOD BETA-D-GLUCAN POSITIVE	10083491	2
ILEOCAECAL RESECTION	10083501	2
ORAL PURPURA	10083533	2
ADMINISTRATION SITE WOUND	10083594	2
SUNSCREEN SENSITIVITY	10083629	2
MENINGEAL THICKENING	10083667	2
DEVICE TEMPERATURE ISSUE	10083711	2
COINFECTION	10083750	2
BULLOUS HAEMORRHAGIC DERMATOSIS	10083809	2
ORAL LICHENOID REACTION	10083833	2
FOREIGN BODY IN SKIN OR SUBCUTANEOUS TISSUE	10083982	2
DEVICE PLACEMENT ISSUE	10083995	2
NUTRITIONAL SUPPLEMENT ALLERGY	10084049	2
MANIC SYMPTOM	10084119	2
ANAL ERYTHEMA	10084122	2
ANTI-THYROID ANTIBODY DECREASED	10084158	2
MONTREAL COGNITIVE ASSESSMENT ABNORMAL	10084205	2
COMPARTMENT PRESSURE TEST	10084233	2
PLATELET AGGREGATION TEST	10084286	2
DIVERTICULITIS INTESTINAL PERFORATED	10084304	2
IMMOBILISATION SYNDROME	10084349	2
CORONAVIRUS PNEUMONIA	10084381	2
PRODUCT DELIVERY MECHANISM ISSUE	10084427	2
SACROILIAC JOINT DYSFUNCTION	10084449	2
EYELID CYST REMOVAL	10084552	2
SARS-COV-2 SEPSIS	10084639	2
INTRATYMPANIC INJECTION	10084734	2
TEMPORARY MECHANICAL CIRCULATORY SUPPORT	10084745	2
PROCEDURE ABORTED	10084762	2
SUPERIMPOSED PRE-ECLAMPSIA	10084825	2
PHANTOM VIBRATION SYNDROME	10085176	2
TEMPORARY TRANSVENOUS PACING	10085182	2
FULL BLOOD COUNT INCREASED	10052239	2
LDL/HDL RATIO DECREASED	10052338	2
BILIARY SPHINCTEROTOMY	10052378	2
DILATATION INTRAHEPATIC DUCT ACQUIRED	10052383	2
NEUROENDOCRINE TUMOUR	10052399	2
OEDEMATOUS PANCREATITIS	10052400	2

SMALL INTESTINAL HAEMORRHAGE	10052535	2
COMMINUTED FRACTURE	10052614	2
CANCER GENE CARRIER	10052648	2
TRABECULOPLASTY	10052697	2
CATHETERISATION VENOUS	10052698	2
AEROPHAGIA	10052813	2
EOSINOPHILIC PNEUMONIA ACUTE	10052832	2
EOSINOPHILIC PNEUMONIA CHRONIC	10052833	2
LABOUR STIMULATION	10052855	2
DENTAL IMPRESSION PROCEDURE	10052943	2
PHYSIOTHERAPY CHEST	10052961	2
LOOP ELECTROSURGICAL EXCISION PROCEDURE	10052976	2
LESION EXCISION	10052979	2
PERICARDIAL REPAIR	10053066	2
SIGHT DISABILITY	10053154	2
GENITAL CYST	10053178	2
ILIAC ARTERY STENOSIS	10053216	2
RHINOSCOPY	10053344	2
BLOOD PRESSURE ORTHOSTATIC ABNORMAL	10053354	2
BLOOD PRESSURE ORTHOSTATIC INCREASED	10053355	2
PROGRESSIVE MULTIPLE SCLEROSIS	10053395	2
CLONIC CONVULSION	10053398	2
BENIGN TUMOUR EXCISION	10053435	2
CARDIAC VENTRICULOGRAM	10053445	2
ANALGESIC THERAPY	10053469	2
TRAUMATIC HAEMORRHAGE	10053476	2
PHYSICAL BREAST EXAMINATION ABNORMAL	10053480	2
BRONCHOPLEURAL FISTULA	10053481	2
PACEMAKER GENERATED ARRHYTHMIA	10053486	2
CARDIAC VENTRICULOGRAM LEFT NORMAL	10053498	2
SCAN BONE MARROW ABNORMAL	10053504	2
URINE PROTEIN/CREATININE RATIO NORMAL	10053540	2
URINE ALBUMIN/CREATININE RATIO INCREASED	10053541	2
FOETAL CEREBROVASCULAR DISORDER	10053601	2
FREE HAEMOGLOBIN	10053626	2
IIIRD NERVE DISORDER	10053644	2
ARTIFICIAL HEART DEVICE USER	10053667	2
KINESITHERAPY	10053688	2
CARDIOVASCULAR AUTONOMIC FUNCTION TEST ABNORMAL	10053691	2
SACCADIC EYE MOVEMENT	10053694	2
FOETAL MACROSOMIA	10053700	2
SPINAL NERVE STIMULATOR IMPLANTATION	10053714	2
SIGMOIDECTOMY	10053770	2

TRI-IODOTHYRONINE FREE DECREASED	10053791	2
TRI-IODOTHYRONINE FREE ABNORMAL	10053794	2
BLOOD 1,25-DIHYDROXYCHOLECALCIFEROL DECREASED	10053811	2
BLOOD 25-HYDROXYCHOLECALCIFEROL DECREASED	10053813	2
AGONAL RHYTHM	10054015	2
LIPOPROTEIN (A) NORMAL	10054022	2
PNEUMOCOCCAL SEPSIS	10054047	2
OESTROGEN RECEPTOR ASSAY POSITIVE	10054054	2
OESTROGEN RECEPTOR ASSAY NEGATIVE	10054055	2
PERIHEPATIC DISCOMFORT	10054125	2
TETANUS IMMUNISATION	10054131	2
SUPERIOR MESENTERIC ARTERY SYNDROME	10054156	2
ACID FAST BACILLI INFECTION	10054204	2
ENTEROBACTER PNEUMONIA	10054218	2
ALPHA HAEMOLYTIC STREPTOCOCCAL INFECTION	10054265	2
HELICOBACTER GASTRITIS	10054272	2
DISORDER OF ORBIT	10054717	2
REGRESSIVE BEHAVIOUR	10054720	2
CORNEAL THINNING	10054760	2
MAXILLOFACIAL OPERATION	10054806	2
LIPASE ABNORMAL	10054821	2
TUBO-OVARIAN ABSCESS	10054824	2
MORGANELLA INFECTION	10054876	2
VASCULAR INSUFFICIENCY	10054880	2
METAPLASIA	10054949	2
PANCREATIC ENLARGEMENT	10055024	2
SWEAT GLAND INFECTION	10055027	2
INTERVERTEBRAL DISC INJURY	10055040	2
THYROID CANCER METASTATIC	10055107	2
HEPATIC CANCER METASTATIC	10055110	2
ECZEMA WEEPING	10055182	2
HEPATIC EMBOLISATION	10056203	2
RED BLOOD CELL VACUOLISATION	10056210	2
WEAN FROM VENTILATOR	10056353	2
CUBITAL TUNNEL SYNDROME	10056473	2
PRE-EXISTING DISEASE	10056486	2
THERAPEUTIC ASPIRATION	10056553	2
URINE ALCOHOL TEST	10056564	2
URINE ALCOHOL TEST NEGATIVE	10056565	2
VESTIBULITIS	10056651	2
LYMPHOMATOID PAPULOSIS	10056670	2
GENITAL DISCHARGE	10056740	2
RECTAL EXAMINATION ABNORMAL	10056824	2

HYDROCHOLECYSTIS	10056972	2
RECTAL SPASM	10057011	2
PNEUMATOSIS INTESTINALIS	10057030	2
URINE URIC ACID DECREASED	10057139	2
T-LYMPHOCYTE COUNT INCREASED	10057264	2
PARVOVIRUS INFECTION	10057343	2
REHABILITATION THERAPY	10057374	2
RETINAL INJURY	10057430	2
VASCULAR PROCEDURE COMPLICATION	10057462	2
HYPERBARIC OXYGEN THERAPY	10057480	2
VISUAL TRACKING TEST ABNORMAL	10057515	2
PERIPHERAL ARTERY ANGIOPLASTY	10057518	2
PURPURA NON-THROMBOCYTOPENIC	10057739	2
HAEMATOLOGY TEST ABNORMAL	10057755	2
VERTEBRAL ARTERY THROMBOSIS	10057777	2
PERITONEAL LAVAGE	10057837	2
NICOTINE DEPENDENCE	10057852	2
ANTICONVULSANT DRUG LEVEL DECREASED	10057857	2
SELECTIVE IGG SUBCLASS DEFICIENCY	10057863	2
PHARYNGITIS BACTERIAL	10057869	2
VENTRICULAR INTERNAL DIAMETER ABNORMAL	10057922	2
BAND NEUTROPHIL COUNT DECREASED	10057950	2
INFECTIVE THROMBOSIS	10058016	2
WOUND SEPSIS	10058041	2
WOUND ABSCESS	10058042	2
NEUTROPHILIC DERMATOSIS	10058105	2
NEPHROGENIC ANAEMIA	10058116	2
NEUTROPHIL PERCENTAGE ABNORMAL	10058134	2
HEART ALTERNATION	10058155	2
GALLBLADDER ADENOCARCINOMA	10058286	2
RETROPERITONEAL HAEMATOMA	10058360	2
BIOPSY INTESTINE NORMAL	10058399	2
DERMATOLOGIC EXAMINATION NORMAL	10058407	2
ARTIFICIAL CROWN PROCEDURE	10058456	2
OESOPHAGEAL INJURY	10058522	2
TOTAL CHOLESTEROL/HDL RATIO NORMAL	10058634	2
CYTOMEGALOVIRUS INFECTION REACTIVATION	10058666	2
PELVIC INFECTION	10058674	2
UTERINE CERVICAL PAIN	10058704	2
CEREBROVASCULAR INSUFFICIENCY	10058842	2
PNEUMOCOCCAL BACTERAEMIA	10058859	2
HEART VALVE CALCIFICATION	10058968	2
JOINT ABSCESS	10058981	2

ORGAN DONOR	10058985	2
RIB DEFORMITY	10059011	2
NEOPLASM SWELLING	10059049	2
MEDICAL DEVICE PAIN	10059057	2
VIBRATION TEST ABNORMAL	10059101	2
INFUSION SITE BRUISING	10059203	2
JAW OPERATION	10059242	2
URINE CYTOLOGY	10059263	2
URINE CYTOLOGY NORMAL	10059264	2
EPIDERMAL NECROSIS	10059284	2
INCISION SITE PRURITUS	10059386	2
BREAST INJURY	10059391	2
ACANTHOMA	10059394	2
PAROPHTHALMIA	10059407	2
PROLONGED EXPIRATION	10059411	2
PARANEOPLASTIC SYNDROME	10059420	2
SKIN NEOPLASM EXCISION	10059443	2
MYELOCYTE PERCENTAGE	10059470	2
PALLIATIVE CARE	10059513	2
RETINOGRAM NORMAL	10059662	2
HISTONE ANTIBODY	10059695	2
CSF MYELIN BASIC PROTEIN INCREASED	10059754	2
RUSSELL'S VIPER VENOM TIME ABNORMAL	10059759	2
PNEUMATIC COMPRESSION THERAPY	10059829	2
CARDIAC RESYNCHRONISATION THERAPY	10059862	2
CYTOGENETIC ANALYSIS ABNORMAL	10059887	2
BLOOD 1,25-DIHYDROXYCHOLECALCIFEROL	10059897	2
GLUCOSE-6-PHOSPHATE DEHYDROGENASE NORMAL	10059901	2
PORPHYRINS URINE NORMAL	10059911	2
RED BLOOD CELL ANISOCYTES PRESENT	10059915	2
RED BLOOD CELL POIKILOCYTES PRESENT	10059947	2
ALBUMIN CSF	10059953	2
TOTAL BILE ACIDS	10059962	2
5-HYDROXYINDOLACETIC ACID IN URINE	10059972	2
ALBUMIN CSF NORMAL	10059977	2
ALBUMIN CSF INCREASED	10059978	2
PLASMIN INHIBITOR	10059980	2
B-LYMPHOCYTE COUNT INCREASED	10059998	2
MEASLES ANTIBODY	10060066	2
BLOOD MERCURY	10060067	2
BLOOD MERCURY NORMAL	10060092	2
BLOOD MERCURY ABNORMAL	10060093	2
PARASITE BLOOD TEST POSITIVE	10060124	2

ANTIPSYCHOTIC DRUG LEVEL BELOW THERAPEUTIC	10060145	2
PROTEIN C INCREASED	10060230	2
PULMONARY ARTERIAL PRESSURE NORMAL	10060232	2
GASTROINTESTINAL EROSION	10060709	2
LYMPHOCYTE STIMULATION TEST NEGATIVE	10060759	2
HEPATIC ENZYME DECREASED	10060794	2
WHITE BLOOD CELL MORPHOLOGY NORMAL	10060798	2
CHOROIDAL NEOVASCULARISATION	10060823	2
HUMAN HERPES VIRUS 6 SEROLOGY POSITIVE	10060827	2
RECALL PHENOMENON	10060856	2
LIGHT CHAIN ANALYSIS ABNORMAL	10060867	2
DIFFUSE ALVEOLAR DAMAGE	10060902	2
BASE EXCESS NEGATIVE	10060915	2
FOETAL MALFORMATION	10060919	2
INTRAOCULAR PRESSURE TEST NORMAL	10060952	2
BENIGN UTERINE NEOPLASM	10061007	2
CEREBELLAR HAEMATOMA	10061038	2
COLON NEOPLASM	10061045	2
CSF WHITE BLOOD CELL COUNT POSITIVE	10061100	2
DRUG ABUSER	10061111	2
DRUG LEVEL THERAPEUTIC	10061134	2
SPONTANEOUS BACTERIAL PERITONITIS	10061135	2
GENITAL HAEMORRHAGE	10061178	2
HAEMODYNAMIC TEST	10061189	2
HEAD DEFORMITY	10061199	2
HELMINTHIC INFECTION	10061201	2
LARGE INTESTINAL ULCER HAEMORRHAGE	10061262	2
MANTLE CELL LYMPHOMA	10061275	2
MEDIASTINAL DISORDER	10061280	2
MENINGEAL DISORDER	10061281	2
MENTAL DISORDER DUE TO A GENERAL MEDICAL CONDITION	10061285	2
MYRINGITIS	10061302	2
OPTIC DISC DISORDER	10061321	2
PERICARDIAL DISEASE	10061338	2
PNEUMOCOCCAL INFECTION	10061353	2
TRAUMATIC INTRACRANIAL HAEMORRHAGE	10061387	2
URTICARIA THERMAL	10061399	2
WRIST DEFORMITY	10061415	2
CYST ASPIRATION ABNORMAL	10061420	2
RETINAL FUNCTION TEST NORMAL	10061463	2
RADIOISOTOPE SCAN	10061478	2
REPRODUCTIVE TRACT DISORDER	10061483	2
PLATELET FUNCTION TEST ABNORMAL	10061539	2

PLATELET FUNCTION TEST NORMAL	10061540	2
FACTITIOUS DISORDER	10061595	2
ABDOMINAL WALL OPERATION	10061611	2
ABORTION SPONTANEOUS INCOMPLETE	10061617	2
PLATELET FUNCTION TEST	10061684	2
BLOOD URIC ACID ABNORMAL	10061727	2
BONE MARROW TRANSPLANT	10061730	2
CATECHOLAMINES URINE NORMAL	10061749	2
COMPLICATION OF DELIVERY	10061781	2
DETOXIFICATION	10061814	2
EAR OPERATION	10061831	2
EXTRANODAL MARGINAL ZONE B-CELL LYMPHOMA (MALT TYP	10061850	2
EYELID OPERATION	10061853	2
NEOPLASM RECURRENCE	10061864	2
NON-SMALL CELL LUNG CANCER	10061873	2
PANCREATIC ENZYMES NORMAL	10061901	2
SALIVARY GLAND CANCER	10061934	2
LIVER SCAN NORMAL	10061948	2
RENAL SCAN NORMAL	10061954	2
GASTRITIS BACTERIAL	10061971	2
LYMPHATIC SYSTEM NEOPLASM	10062044	2
HISTOLOGY NORMAL	10062058	2
REDUCTION OF INCREASED INTRACRANIAL PRESSURE	10062101	2
RESPIRATORY GAS EXCHANGE DISORDER	10062105	2
WHITE BLOOD CELL MORPHOLOGY ABNORMAL	10062137	2
URINARY SYSTEM X-RAY NORMAL	10062139	2
SPLEEN SCAN NORMAL	10062143	2
THYROID GLAND SCAN NORMAL	10062150	2
VENOUS ANEURYSM	10062174	2
VIRAL LOAD INCREASED	10062180	2
URINE ELECTROLYTES	10062229	2
ELECTROCARDIOGRAM LOW VOLTAGE	10062311	2
LIPOHYPERTROPHY	10062315	2
CONGENITAL CEREBROVASCULAR ANOMALY	10062327	2
LEUKAEMIA RECURRENT	10062489	2
TRACHEAL HAEMORRHAGE	10062543	2
THROMBOSIS IN DEVICE	10062546	2
ENTEROVESICAL FISTULA	10062570	2
ENDOCARDITIS NONINFECTIVE	10062608	2
ISCHAEMIC LIMB PAIN	10062610	2
METAMYELOCYTE PERCENTAGE INCREASED	10062649	2
ERYTHROCYTE OSMOTIC FRAGILITY TEST	10062724	2
ULTRASOUND THERAPY	10062749	2

CAECECTOMY	10062859	2
PLACENTA ACCRETA	10062936	2
ABDOMINAL WALL MASS	10063005	2
PROGESTERONE ABNORMAL	10063270	2
LYMPHOCYTE PERCENTAGE ABNORMAL	10063337	2
INFANTILE SPITTING UP	10063338	2
INTERNATIONAL NORMALISED RATIO FLUCTUATION	10063351	2
VASCULAR STENT INSERTION	10063382	2
HEPATITIS B ANTIGEN POSITIVE	10063411	2
HEPATITIS B ANTIGEN	10063414	2
ROTAVIRUS TEST	10063459	2
ROTAVIRUS TEST NEGATIVE	10063460	2
VARICELLA POST VACCINE	10063522	2
ALLERGIC RESPIRATORY DISEASE	10063532	2
RENAL EMBOLISM	10063544	2
PULMONARY ARTERY WALL HYPERTROPHY	10063561	2
OTOACOUSTIC EMISSIONS TEST	10063567	2
EXPOSURE TO CONTAMINATED AIR	10063600	2
LIFE EXPECTANCY SHORTENED	10063750	2
IMPLANT SITE PRURITUS	10063785	2
PELVIC FLOOR MUSCLE WEAKNESS	10064026	2
LIMB CRUSHING INJURY	10064031	2
BIOPSY TONSIL	10064045	2
POLYMENORRHAGIA	10064050	2
PUNCTAL PLUG INSERTION	10064120	2
HAND-ARM VIBRATION SYNDROME	10064140	2
DENTAL IMPLANTATION	10064143	2
FIBRONECTIN INCREASED	10064166	2
FIBRONECTIN DECREASED	10064167	2
ELECTIVE PROCEDURE	10064263	2
BLADDER NEOPLASM SURGERY	10064272	2
INCORRECT DRUG ADMINISTRATION RATE	10064306	2
INTERCEPTED MEDICATION ERROR	10064382	2
MONOFILAMENT PRESSURE PERCEPTION TEST	10064511	2
PERIODIC LIMB MOVEMENT DISORDER	10064600	2
MACROPHAGES INCREASED	10064665	2
FAECAL VOMITING	10064670	2
EPIRETINAL MEMBRANE	10064697	2
INFUSION SITE EXTRAVASATION	10064774	2
PLATELETCRIT	10064782	2
ABSCESS RUPTURE	10064878	2
CYSTATIN C	10064988	2
NECROTISING RETINITIS	10064997	2

UTERINE CERVIX DILATION PROCEDURE	10064999	2
IMMUNE RECONSTITUTION INFLAMMATORY SYNDROME	10065042	2
UROGRAM ABNORMAL	10065097	2
EPSTEIN-BARR VIRAEMIA	10065110	2
LIGAMENT OPERATION	10065122	2
VERTEBRAL WEDGING	10065317	2
MAGNETIC THERAPY	10065343	2
BURNOUT SYNDROME	10065369	2
VON WILLEBRAND'S FACTOR ANTIGEN INCREASED	10065374	2
CORONARY ARTERY DILATATION	10065420	2
MINI MENTAL STATUS EXAMINATION ABNORMAL	10065424	2
NIH STROKE SCALE SCORE DECREASED	10065529	2
BLOOD PRODUCT TRANSFUSION DEPENDENT	10065535	2
MESENTERIC ARTERIOSCLEROSIS	10065560	2
RHEGMATOGENOUS RETINAL DETACHMENT	10065569	2
FEMALE SEX HORMONE LEVEL ABNORMAL	10065596	2
INTESTINAL CONGESTION	10065611	2
INCISION SITE ERYTHEMA	10065615	2
LIP INFECTION	10065755	2
GASTROINTESTINAL ANASTOMOTIC LEAK	10065879	2
PREHYPERTENSION	10065918	2
LEFT VENTRICLE OUTFLOW TRACT OBSTRUCTION	10065930	2
PNEUMOBILIA	10066004	2
PHALEN'S TEST POSITIVE	10066039	2
DYSMORPHISM	10066054	2
PARADOXICAL EMBOLISM	10066059	2
SUBSTANCE ABUSE	10066169	2
INJECTION SITE ECZEMA	10066221	2
COMPULSIVE LIP BITING	10066241	2
INTERLEUKIN-2 RECEPTOR INCREASED	10066315	2
PLEURAL DECORTICATION	10066322	2
RECTAL TUBE INSERTION	10066323	2
CONTINUOUS HAEMODIAFILTRATION	10066338	2
HYPERSEXUALITY	10066364	2
MATERNAL DEATH DURING CHILDBIRTH	10066376	2
INFRARED THERAPY	10066385	2
PULMONARY VENOUS HYPERTENSION	10085364	2
MYXOMA	10085372	2
NECROTIC LYMPHADENOPATHY	10085419	2
SARS-COV-2 RNA FLUCTUATION	10085497	2
CEREBELLAR TONSILLAR ECTOPIA	10085525	2
CUPPING THERAPY	10085708	2
UTERINE ARTERY EMBOLISATION	10085781	2

MYELIN OLIGODENDROCYTE GLYCOPROTEIN ANTIBODY-ASSOC	10085806	2
LOW LUNG COMPLIANCE	10086117	2
ANTI-PLATELET FACTOR 4 ANTIBODY TEST	10086159	2
17-HYDROXYPROGESTERONE	10086164	2
RUBULAVIRUS TEST	10086209	2
ORAL CAVITY EXAMINATION	10086211	2
MYOCARDIAL STRAIN IMAGING	10086297	2
PELVIC ORGAN PROLAPSE	10086381	2
OPHTHALMIC ARTERY ANEURYSM	10086406	2
BOWEL OBSTRUCTION SURGERY	10086417	2
PRODUCT DOSE CONFUSION	10086483	2
CANCER SCREENING	10086509	2
ASIA SYNDROME	10086562	2
PULMONARY SEPTAL THICKENING	10086984	2
WARM AUTOIMMUNE HAEMOLYTIC ANAEMIA	10087091	2
VIBRATION SYNDROME	10087138	2
SUPERFICIAL INFLAMMATORY DERMATOSIS	10087164	2
URINE ANTIGEN TEST	10087202	2
CONTRAST ECHOCARDIOGRAM	10087512	2
ABDOMEN CRUSHING	10000044	1
ABNORMAL LABOUR	10000153	1
ABNORMAL PALMAR/PLANTAR CREASES	10000162	1
ABORTION INCOMPLETE	10000217	1
ABSCESS OF EYELID	10000297	1
ABSCESS STERILE	10000317	1
ACID BASE BALANCE	10000456	1
ACIDOSIS HYPERCHLORAEMIC	10000489	1
ACRODYNIA	10000598	1
ACTIVATED PARTIAL THROMBOPLASTIN TIME ABNORMAL	10000631	1
ACUTE FATTY LIVER OF PREGNANCY	10000746	1
ACUTE HIV INFECTION	10000807	1
ACUTE MEGAKARYOCYTIC LEUKAEMIA	10000860	1
ACUTE MONOCYTIC LEUKAEMIA	10000871	1
ACUTE MYELOMONOCYTIC LEUKAEMIA	10000890	1
ADENOCARCINOMA GASTRIC	10001150	1
ADENOCARCINOMA OF COLON	10001167	1
ADENOIDECTOMY	10001230	1
AGRANULOCYTOSIS	10001507	1
AIR EMBOLISM	10001526	1
ALBUMIN GLOBULIN RATIO ABNORMAL	10001563	1
ALBUMINURIA	10001580	1
ALCOHOL PROBLEM	10001606	1
ALCOHOLIC	10001612	1

ALCOHOLIC HANGOVER	10001623	1
ALCOHOLIC LIVER DISEASE	10001627	1
ALDOLASE DECREASED	10001641	1
ALLERGY TO VENOM	10001751	1
ALOPECIA SCARRING	10001764	1
ALPHA 2 GLOBULIN DECREASED	10001794	1
ALPHA-1 ANTI-TRYPSIN DECREASED	10001805	1
ALPHA-1 ANTI-TRYPSIN INCREASED	10001808	1
ALPHA-2 MACROGLOBULIN INCREASED	10001814	1
ALVEOLITIS	10001889	1
AMYOTROPHY	10002027	1
ANAEMIA MEGALOBLASTIC	10002065	1
ANAEMIA VITAMIN B12 DEFICIENCY	10002080	1
ANAL ATRESIA	10002120	1
ANAL CANDIDIASIS	10002140	1
ANAL FISTULA	10002156	1
ANAL SKIN TAGS	10002172	1
ANAL SPHINCTEROTOMY	10002175	1
ANAL STENOSIS	10002176	1
ANAL ULCER	10002180	1
ANAPLASTIC THYROID CANCER	10002240	1
ANASTOMOTIC ULCER	10002243	1
ANDROGENS INCREASED	10002265	1
ANEURYSMAL BONE CYST	10002362	1
ANGIOGRAM RETINA NORMAL	10002445	1
ANHIDROSIS	10002512	1
ANORGASMIA	10002652	1
ANOXIA	10002660	1
ANTIEMETIC SUPPORTIVE CARE	10002781	1
ANTIMICROBIAL SUSCEPTIBILITY TEST RESISTANT	10002793	1
ANTISOCIAL BEHAVIOUR	10002820	1
AORTIC INJURY	10002899	1
APHAKIA	10002945	1
APLASIA	10002961	1
APPLICATION SITE OEDEMA	10003050	1
APPLICATION SITE WARMTH	10003060	1
ARACHNOIDITIS	10003074	1
ARTERIOVENOUS FISTULA OPERATION	10003190	1
ARTERITIS CORONARY	10003232	1
ARTHRITIS VIRAL	10003274	1
ASBESTOSIS	10003441	1
ASPARTATE AMINOTRANSFERASE ABNORMAL	10003477	1
ASPIRATION TRACHEAL NORMAL	10003532	1

ASTERIXIS	10003547	1
AXILLARY NERVE INJURY	10003879	1
B-CELL LYMPHOMA STAGE II	10003905	1
BACTERIAL PERICARDITIS	10004050	1
BARIUM ENEMA NORMAL	10004117	1
BARTHOLIN'S CYST REMOVAL	10004141	1
BARTONELLOSIS	10004145	1
BASOSQUAMOUS CARCINOMA	10004178	1
BELLIGERENCE	10004224	1
BENIGN CARDIAC NEOPLASM	10004245	1
BENIGN NEOPLASM OF BLADDER	10004302	1
BENIGN NEOPLASM OF SKIN	10004398	1
BENIGN PROSTATIC HYPERPLASIA	10004446	1
BETA 2 MICROGLOBULIN NORMAL	10004498	1
BILIRUBINURIA	10004710	1
BIOPSY ABDOMINAL WALL	10004721	1
BIOPSY ADRENAL GLAND	10004724	1
BIOPSY ADRENAL GLAND ABNORMAL	10004725	1
BIOPSY BLADDER NORMAL	10004732	1
BIOPSY BONE NORMAL	10004740	1
BIOPSY BRONCHUS ABNORMAL	10004748	1
BIOPSY HEART NORMAL	10004781	1
BIOPSY MUCOSA ABNORMAL	10004800	1
BIOPSY MUCOSA NORMAL	10004801	1
BIOPSY MUSCLE NORMAL	10004804	1
BIOPSY OESOPHAGUS	10004808	1
BIOPSY PERICARDIUM	10004842	1
BIOPSY PERICARDIUM ABNORMAL	10004843	1
BIOPSY PERIPHERAL NERVE ABNORMAL	10004846	1
BIOPSY PERIPHERAL NERVE NORMAL	10004847	1
BIOPSY PERITONEUM	10004848	1
BIOPSY RECTUM ABNORMAL	10004860	1
BIOPSY SPINAL CORD	10004876	1
BIOPSY TESTES	10004882	1
BIOPSY TRACHEA	10004894	1
BIOPSY TRACHEA ABNORMAL	10004895	1
BIOPSY VAGINA ABNORMAL	10004901	1
BIOPSY VULVA	10004903	1
BIOPSY VULVA NORMAL	10004905	1
BIRTH MARK	10004950	1
BIRTH TRAUMA	10004954	1
BLADDER CANCER RECURRENT	10005005	1
BLADDER CATHETER REPLACEMENT	10005026	1

BLADDER DIVERTICULUM	10005038	1
BLADDER NEOPLASM	10005056	1
BLEEDING TIME SHORTENED	10005141	1
BLOOD ALDOSTERONE INCREASED	10005296	1
BLOOD ANTIDIURETIC HORMONE NORMAL	10005336	1
BLOOD ARSENIC INCREASED	10005342	1
BLOOD BACTERICIDAL ACTIVITY	10005344	1
BLOOD CADMIUM	10005378	1
BLOOD CAFFEINE INCREASED	10005384	1
BLOOD CALCITONIN NORMAL	10005391	1
BLOOD CARBON MONOXIDE INCREASED	10005409	1
BLOOD CATECHOLAMINES INCREASED	10005414	1
BLOOD CHLORIDE ABNORMAL	10005417	1
BLOOD CHOLINESTERASE	10005428	1
BLOOD CHROMIUM	10005433	1
BLOOD CHROMIUM NORMAL	10005436	1
BLOOD COPPER ABNORMAL	10005440	1
BLOOD CORTICOTROPHIN DECREASED	10005452	1
BLOOD ETHANAL INCREASED	10005512	1
BLOOD ETHANOL DECREASED	10005514	1
BLOOD GASTRIN DECREASED	10005544	1
BLOOD GONADOTROPHIN NORMAL	10005564	1
BLOOD GROUP A	10005565	1
BLOOD INSULIN DECREASED	10005613	1
BLOOD LEAD INCREASED	10005642	1
BLOOD METHANOL	10005663	1
BLOOD PARATHYROID HORMONE ABNORMAL	10005700	1
BLOOD PRESSURE AMBULATORY INCREASED	10005732	1
BLOOD PRESSURE DIASTOLIC ABNORMAL	10005736	1
BLOOD THROMBIN	10005817	1
BLOOD THROMBIN INCREASED	10005821	1
BLOOD TRIGLYCERIDES ABNORMAL	10005837	1
BLOOD URIC ACID DECREASED	10005860	1
BONE DEVELOPMENT ABNORMAL	10005954	1
BONE HYPERTROPHY	10005973	1
BRAIN STEM GLIOMA	10006143	1
BRANCHIAL CYST	10006164	1
BREAST CYST DRAINAGE	10006221	1
BREAST MALFORMATION	10006271	1
BREECH DELIVERY	10006346	1
BRONCHIAL OBSTRUCTION	10006440	1
BRONCHOGRAM	10006465	1
BRONCHOPULMONARY ASPERGILLOSIS	10006473	1

BROWN-SEQUARD SYNDROME	10006491	1
BUDD-CHIARI SYNDROME	10006537	1
BULBAR PALSY	10006542	1
BULLOUS IMPETIGO	10006563	1
BURKITT'S LYMPHOMA	10006595	1
BURNS FIRST DEGREE	10006797	1
BURSA REMOVAL	10006807	1
CAECOPEXY	10006920	1
CAMPYLOBACTER GASTROENTERITIS	10007048	1
CARBON MONOXIDE POISONING	10007238	1
CARBOXYHAEMOGLOBIN DECREASED	10007241	1
CARCINOGENICITY	10007269	1
CARCINOID TUMOUR PULMONARY	10007282	1
CARDIAC ARREST NEONATAL	10007516	1
CARDIAC FAILURE HIGH OUTPUT	10007560	1
CARDIAC INDEX	10007575	1
CARDIAC INDEX DECREASED	10007577	1
CARDIAC MALPOSITION	10007585	1
CARDIOVASCULAR FUNCTION TEST ABNORMAL	10007651	1
CARNITINE NORMAL	10007670	1
CD4 LYMPHOCYTES DECREASED	10007839	1
CENTRAL NERVOUS SYSTEM FUNCTION TEST ABNORMAL	10007947	1
CENTRAL VENOUS PRESSURE INCREASED	10007980	1
CEREBELLAR ARTERY THROMBOSIS	10008023	1
CEREBRAL ARTERIOVENOUS MALFORMATION HAEMORRHAGI	10008086	1
CERULOPLASMIN DECREASED	10008217	1
CERVICAL NEURITIS	10008293	1
CERVICITIS TRICHOMONAL	10008332	1
CHEYNE-STOKES RESPIRATION	10008501	1
CHLOROPSIA	10008585	1
CHOLANGIOGRAM NORMAL	10008600	1
CHOLERA	10008631	1
CHONDROMALACIA	10008729	1
CHOROID MELANOMA	10008773	1
CHRONIC HEPATITIS B	10008910	1
CHRONIC LYMPHOCYTIC LEUKAEMIA STAGE 0	10008963	1
CHRONIC PAROXYSMAL HEMICRANIA	10009094	1
CIRCUMSTANTIALITY	10009207	1
CLEFT LIP AND PALATE	10009260	1
CLOTTING FACTOR TRANSFUSION	10009678	1
CNS VENTRICULITIS	10009719	1
COAGULATION FACTOR V LEVEL DECREASED	10009754	1
COAGULATION FACTOR X LEVEL	10009773	1

COAGULATION FACTOR XI LEVEL	10009777	1
COAGULATION FACTOR XII LEVEL	10009781	1
COAGULATION TIME NORMAL	10009794	1
COGWHEEL RIGIDITY	10009848	1
COLD AGGLUTININS NEGATIVE	10009853	1
COLLAGEN-VASCULAR DISEASE	10009905	1
COLON CANCER STAGE IV	10009956	1
COLON GANGRENE	10009971	1
COLONIC FISTULA	10009995	1
COLOSTOMY INFECTION	10010046	1
COLOUR BLINDNESS ACQUIRED	10010051	1
COLOUR VISION TESTS ABNORMAL	10010056	1
COMPLEMENT FIXATION TEST POSITIVE	10010134	1
COMPLICATED FRACTURE	10010149	1
COMPLICATIONS OF TRANSPLANT SURGERY	10010183	1
COMPLICATIONS OF TRANSPLANTED KIDNEY	10010185	1
COMPLICATIONS OF TRANSPLANTED LUNG	10010187	1
COMPULSIONS	10010219	1
CONDOM	10010274	1
CONGENITAL CYSTIC KIDNEY DISEASE	10010428	1
CONGENITAL DIAPHRAGMATIC HERNIA	10010439	1
CONGENITAL HYDROCEPHALUS	10010506	1
CONGENITAL MEGAURETER	10010540	1
CONGENITAL SPINAL FUSION	10010629	1
CONJOINED TWINS	10010688	1
CONJUNCTIVAL CYST	10010703	1
CONJUNCTIVAL DISCOLOURATION	10010712	1
CONJUNCTIVAL SCAR	10010733	1
CONTRACEPTIVE DIAPHRAGM	10010811	1
CONTRAINDICATION TO MEDICAL TREATMENT	10010833	1
COPPER DEFICIENCY	10010957	1
COPROLALIA	10010964	1
CORNEAL DEGENERATION	10010996	1
CORONARY ARTERY ANEURYSM	10011071	1
CORONARY ARTERY SURGERY	10011090	1
CORTISOL FREE URINE NORMAL	10011205	1
COW POX	10011238	1
CREATINE URINE	10011353	1
CROSS SENSITIVITY REACTION	10011411	1
CSF ELECTROPHORESIS	10011530	1
CSF LACTATE DEHYDROGENASE NORMAL	10011545	1
CULTURE THROAT POSITIVE	10011634	1
CUSHING'S SYNDROME	10011652	1

CUSHINGOID	10011655	1
CUTANEOUS SARCOIDOSIS	10011674	1
CYCLOTHYMIC DISORDER	10011724	1
CYSTOGRAM ABNORMAL	10011805	1
CYSTOGRAM NORMAL	10011806	1
DACRYOCANALICULITIS	10011843	1
DACRYOCYSTITIS	10011844	1
DEAFNESS TRAUMATIC	10011903	1
DEATH OF PET	10011930	1
DELAYED DELIVERY	10012186	1
DELIRIUM TREMENS	10012225	1
DELUSION OF REPLACEMENT	10012245	1
DENGUE FEVER	10012310	1
DEPILATION	10012364	1
DEPOSIT EYE	10012369	1
DEVICE EXTRUSION	10012579	1
DIABETIC RETINAL OEDEMA	10012688	1
DIFFUSE LARGE B-CELL LYMPHOMA RECURRENT	10012821	1
DIFFUSE LARGE B-CELL LYMPHOMA STAGE IV	10012826	1
DIFFUSE VASCULITIS	10012978	1
DIGEORGE'S SYNDROME	10012979	1
DILATATION VENTRICULAR	10013012	1
DIPHTHERIA	10013023	1
DIPLACUSIS	10013032	1
DISSEMINATED TUBERCULOSIS	10013453	1
DISSOCIATIVE IDENTITY DISORDER	10013468	1
DIVERTICULITIS INTESTINAL HAEMORRHAGIC	10013541	1
DIVERTICULUM GASTRIC	10013558	1
DIVERTICULUM INTESTINAL HAEMORRHAGIC	10013560	1
DRUG WITHDRAWAL CONVULSIONS	10013752	1
DUODENAL OBSTRUCTION	10013830	1
DUODENAL PERFORATION	10013832	1
DUODENAL ULCER PERFORATION	10013849	1
DYSDIADOCHOKINESIS	10013896	1
EAR DEFORMITY ACQUIRED	10014002	1
EBOLA DISEASE	10014071	1
ECTHYMA	10014141	1
ECTROPION	10014179	1
ELECTROCARDIOGRAM DELTA WAVES ABNORMAL	10014372	1
ELECTROCARDIOGRAM QT INTERVAL NORMAL	10014386	1
ELECTROCOCHLEOGRAM ABNORMAL	10014399	1
ELEPHANTIASIS	10014472	1
EMPTY SELLA SYNDROME	10014567	1

EMPYEMA DRAINAGE	10014569	1
ENCEPHALITIS MENINGOCOCCAL	10014597	1
ENDOCARDITIS ENTEROCOCCAL	10014671	1
ENDOMETRIAL CANCER STAGE II	10014739	1
ENDOMETRIAL CANCER STAGE III	10014740	1
ENDOMETRIAL NEOPLASM	10014759	1
ENDOPHTHALMITIS	10014801	1
ENDOSCOPIC RETROGRADE CHOLANGIOPANCREATOGRAPHY N	10014804	1
ENDOSCOPY SMALL INTESTINE ABNORMAL	10014817	1
ENTEROBIASIS	10014881	1
ENTEROCOLITIS HAEMORRHAGIC	10014896	1
EOSINOPHILIA MYALGIA SYNDROME	10014952	1
EPENDYMOMA	10014967	1
EPIPHYSIOLYSIS	10015079	1
EPITHELIOID SARCOMA	10015099	1
ERYTHEMA INFECTIOSUM	10015214	1
ERYTHEMA MARGINATUM	10015216	1
EUSTACHIAN TUBE PATULOUS	10015546	1
EUTHYROID SICK SYNDROME	10015549	1
EXCESSIVE EXERCISE	10015599	1
EXERCISE TEST NORMAL	10015654	1
EXHIBITIONISM	10015670	1
EXOMPHALOS	10015677	1
EXOSTOSIS OF JAW	10015690	1
EXSANGUINATION	10015719	1
EYE INFECTION FUNGAL	10015933	1
EYE MUSCLE RECESSION	10015954	1
EYELID BOIL	10015980	1
EYELID PTOSIS CONGENITAL	10015996	1
FACE CRUSHING	10016023	1
FALLOPIAN TUBE CANCER STAGE III	10016186	1
FAMILIAL TREMOR	10016212	1
FASCIA RELEASE	10016223	1
FASCIECTOMY	10016227	1
FAT EMBOLISM	10016246	1
FEAR OF OPEN SPACES	10016279	1
FEMORAL NERVE INJURY	10016451	1
FEVER NEONATAL	10016562	1
FIBRINOLYSIS INCREASED	10016607	1
FIBRINOLYSIS NORMAL	10016610	1
FINGER REPAIR OPERATION	10016687	1
FLAT CHEST	10016761	1
FLEA INFESTATION	10016773	1

FOETAL-MATERNAL HAEMORRHAGE	10016871	1
FOOD INTERACTION	10016948	1
FOOT AMPUTATION	10016960	1
FOOT AND MOUTH DISEASE	10016961	1
FOREIGN BODY ASPIRATION	10017000	1
FOREIGN BODY IN EAR	10017010	1
FRACTURE NONUNION	10017088	1
FRAGILE X SYNDROME	10017324	1
FRUCTOSAMINE	10017393	1
FUMBLING	10017472	1
FUNCTIONAL RESIDUAL CAPACITY ABNORMAL	10017505	1
FUNCTIONAL RESIDUAL CAPACITY DECREASED	10017507	1
FUNDOSCOPY ABNORMAL	10017520	1
GALLBLADDER CANCER	10017614	1
GAMMA RADIATION THERAPY TO BRAIN	10017676	1
GAMMA-GLUTAMYLTRANSFERASE DECREASED	10017690	1
GAS GANGRENE	10017737	1
GASTRIC LAVAGE	10017792	1
GASTRIC MUCOSAL HYPERTROPHY	10017807	1
GASTRIC ULCER HAEMORRHAGE	10017826	1
GASTROENTERITIS PROTEUS	10017910	1
GASTROENTERITIS SALMONELLA	10017914	1
GASTROINTESTINAL CARCINOMA	10017940	1
GENERAL ANAESTHESIA	10018060	1
GINGIVAL EROSION	10018282	1
GINGIVITIS ULCERATIVE	10018296	1
GLIOMA	10018338	1
GLOMERULONEPHRITIS ACUTE	10018366	1
GLOMERULONEPHRITIS PROLIFERATIVE	10018376	1
GLUCOSE TOLERANCE IMPAIRED IN PREGNANCY	10018430	1
GONADOTROPHIN RELEASING HORMONE STIMULATION TEST	10018519	1
GOODPASTURE'S SYNDROME	10018620	1
GOUTY TOPHUS	10018641	1
GROWTH ACCELERATED	10018746	1
HAEMANGIOBLASTOMA	10018813	1
HAEMANGIOMA OF BREAST	10018820	1
HAEMOCONCENTRATION	10018873	1
HAEMOGLOBIN A PRESENT	10018878	1
HALLUCINATION, GUSTATORY	10019071	1
HAND REPAIR OPERATION	10019118	1
HEART TRANSPLANT REJECTION	10019315	1
HEAT OEDEMA	10019340	1
HEINZ BODIES	10019372	1

HEPATIC ADENOMA	10019629	1
HEPATIC ARTERY THROMBOSIS	10019636	1
HEPATIC ATROPHY	10019637	1
HEPATIC HAEMATOMA	10019676	1
HEPATIC RUPTURE	10019707	1
HEPATITIS B ANTIBODY NORMAL	10019735	1
HEPATITIS B SURFACE ANTIGEN POSITIVE	10019742	1
HEPATITIS C RNA NEGATIVE	10019749	1
HEPATITIS C RNA POSITIVE	10019750	1
HEPATITIS D	10019762	1
HEPATITIS FULMINANT	10019772	1
HEPATITIS INFECTIOUS MONONUCLEOSIS	10019781	1
HEPATITIS TOXIC	10019795	1
HEPATORENAL SYNDROME	10019846	1
HEREDITARY OPTIC ATROPHY	10019895	1
HEREDITARY SPHEROCYTOSIS	10019904	1
HIP DISARTICULATION	10020098	1
HISTOPLASMOSIS DISSEMINATED	10020144	1
HODGKIN'S DISEASE NODULAR SCLEROSIS STAGE III	10020252	1
HODGKIN'S DISEASE STAGE II	10020269	1
HUMAN HERPESVIRUS 6 INFECTION	10020431	1
HYALOSIS ASTEROID	10020478	1
HYPERBILIRUBINAEMIA NEONATAL	10020580	1
HYPERKINESIA	10020651	1
HYPERPARATHYROIDISM PRIMARY	10020707	1
HYPERSPLENISM	10020769	1
HYPERTROPHIC SCAR	10020879	1
HYPERVITAMINOSIS	10020915	1
HYPNAGOGIC HALLUCINATION	10020927	1
HYPOLIPIDAEMIA	10021024	1
HYPOMETABOLISM	10021034	1
HYPOPARATHYROIDISM	10021041	1
HYPOPITUITARISM	10021067	1
HYPOPLASTIC LEFT HEART SYNDROME	10021076	1
HYPOPROTEINAEMIA	10021083	1
IIIRD NERVE INJURY	10021281	1
ILIAC ARTERY EMBOLISM	10021338	1
ILLOGICAL THINKING	10021402	1
IMPLANTATION COMPLICATION	10021547	1
IMPRISONMENT	10021554	1
INCISIONAL HERNIA, OBSTRUCTIVE	10021626	1
INFANTILE SPASMS	10021750	1
INFECTED BUNION	10021770	1

INFECTION IN AN IMMUNOCOMPROMISED HOST	10021818	1
INFECTION PROTOZOAL	10021859	1
INFECTIVE MYOSITIS	10021918	1
INJECTION SITE PHLEBITIS	10022090	1
INSPIRATORY CAPACITY ABNORMAL	10022446	1
INSULIN C-PEPTIDE NORMAL	10022480	1
INTERCAPILLARY GLOMERULOSCLEROSIS	10022530	1
INTERLEUKIN THERAPY	10022539	1
INTERMITTENT EXPLOSIVE DISORDER	10022568	1
INTERVENTRICULAR SEPTUM RUPTURE	10022626	1
INTESTINAL PSEUDO-OBSTRUCTION	10022698	1
INTESTINAL STENOSIS	10022699	1
INTESTINAL ULCER	10022714	1
INTRINSIC FACTOR ANTIBODY	10022851	1
INTRINSIC FACTOR ANTIBODY NEGATIVE	10022853	1
IODINE UPTAKE ABNORMAL	10022918	1
IODINE UPTAKE NORMAL	10022924	1
IRON METABOLISM DISORDER	10022983	1
IVTH NERVE INJURY	10023108	1
JAUNDICE NEONATAL	10023138	1
JEJUNOSTOMY	10023180	1
JOINT MANIPULATION	10023220	1
KARYOTYPE ANALYSIS NORMAL	10023304	1
KERATOACANTHOMA	10023347	1
KIDNEY CONTUSION	10023413	1
KIDNEY DUPLEX	10023416	1
KIDNEY FIBROSIS	10023421	1
KIDNEY TRANSPLANT REJECTION	10023439	1
LABORATORY TEST INTERFERENCE	10023550	1
LACRIMAL GLAND OPERATION	10023633	1
LAPAROSCOPY NORMAL	10023695	1
LARGE INTESTINAL OBSTRUCTION REDUCTION	10023794	1
LARYNGEAL PAPILLOMA	10023849	1
LEAD URINE INCREASED	10024088	1
LEIOMYOMA	10024184	1
LENS DISLOCATION	10024203	1
LENTICULAR OPACITIES	10024214	1
LEPTOSPIROSIS	10024238	1
LEUKODERMA	10024380	1
LEUKOENCEPHALOPATHY	10024382	1
LICE INFESTATION	10024424	1
LIGAMENT LAXITY	10024452	1
LIP LESION EXCISION	10024555	1

LIP NEOPLASM MALIGNANT STAGE UNSPECIFIED	10024557	1
LISTERIOSIS	10024641	1
LIVER TENDERNESS	10024712	1
LIVIDITY	10024728	1
LUNG ADENOCARCINOMA STAGE III	10025037	1
LUNG CARCINOMA CELL TYPE UNSPECIFIED RECURRENT	10025065	1
LUNG CARCINOMA CELL TYPE UNSPECIFIED STAGE 0	10025066	1
LUNG CARCINOMA CELL TYPE UNSPECIFIED STAGE I	10025067	1
LUNG TRANSPLANT	10025127	1
LYMPHANGIOGRAM	10025215	1
LYMPHANGIOMA	10025219	1
MALIGNANT MELANOMA IN SITU	10025652	1
MALIGNANT NEOPLASM OF EYE	10025910	1
MASTITIS POSTPARTUM	10026889	1
MEAN ARTERIAL PRESSURE INCREASED	10026985	1
MEASLES ANTIBODY NEGATIVE	10027012	1
MEDIAN NERVE INJURY	10027067	1
MEGACOLON	10027110	1
MEGALOBLASTS INCREASED	10027131	1
MEIBOMIANITIS	10027137	1
MELANODERMA	10027146	1
MENINGIOMA BENIGN	10027192	1
MENINGITIS HERPES	10027242	1
MENINGITIS PNEUMOCOCCAL	10027253	1
MENINGOCOCCAL SEPSIS	10027280	1
MENINGOENCEPHALITIS HERPETIC	10027285	1
MESENTERIC VENOUS OCCLUSION	10027403	1
MESOTHELIOMA	10027406	1
METABOLIC ABNORMALITY MANAGEMENT	10027415	1
METANEPHRINE URINE INCREASED	10027445	1
METASTASES TO ADRENALS	10027451	1
METASTASES TO THE MEDIASTINUM	10027469	1
METHAEMOGLOBIN URINE ABSENT	10027499	1
MICROCEPHALY	10027534	1
MICROCYTOSIS	10027540	1
MICROTIA	10027555	1
MIXED DEAFNESS	10027757	1
MONONUCLEOSIS SYNDROME	10027924	1
MOOD DISORDER DUE TO A GENERAL MEDICAL CONDITION	10027944	1
MOROSE	10027977	1
MOYAMOYA DISEASE	10028047	1
MULTI-VITAMIN DEFICIENCY	10028155	1
MULTIPAROUS	10028163	1

MULTIPLE CONGENITAL ABNORMALITIES	10028182	1
MURINE TYPHUS	10028282	1
MUSCLE REATTACHMENT	10028325	1
MYOGLOBIN URINE PRESENT	10028631	1
MYOMECTOMY	10028634	1
NAIL AVULSION	10028686	1
NAIL DYSTROPHY	10028698	1
NASAL SEPTAL OPERATION	10028760	1
NECK CRUSHING	10028829	1
NEGLECT OF PERSONAL APPEARANCE	10028901	1
NEONATAL ASPHYXIA	10028923	1
NERVE ROOT INJURY LUMBAR	10029184	1
NEURILEMMOMA BENIGN	10029235	1
NEUROFIBROMATOSIS	10029268	1
NEUROMUSCULAR BLOCKADE	10029315	1
NEUROPATHIC ARTHROPATHY	10029326	1
NEUROTOXICITY	10029350	1
NIPPLE INFECTION	10029419	1
NON-SMALL CELL LUNG CANCER STAGE III	10029519	1
NON-CARDIOGENIC PULMONARY OEDEMA	10029538	1
NON-HODGKIN'S LYMPHOMA RECURRENT	10029600	1
NON-HODGKIN'S LYMPHOMA STAGE III	10029604	1
NORMAL PRESSURE HYDROCEPHALUS	10029773	1
NULLIPAROUS	10029827	1
OCCUPATIONAL PROBLEM ENVIRONMENTAL	10030028	1
OCULOGYRIC CRISIS	10030071	1
OCULOMOTOR STUDY ABNORMAL	10030078	1
OEDEMA GENITAL	10030104	1
OESOPHAGEAL ADENOCARCINOMA	10030137	1
OESOPHAGEAL CANDIDIASIS	10030154	1
OESOPHAGEAL PERFORATION	10030181	1
OESOPHAGEAL ULCER HAEMORRHAGE	10030202	1
OESOPHAGEAL VARICEAL LIGATION	10030208	1
OESOPHAGOSCOPY ABNORMAL	10030223	1
OESTROGEN THERAPY	10030252	1
OLIGODENDROGLIOMA	10030286	1
OPTIC DISC HAEMORRHAGE	10030919	1
OPTICOKINETIC NYSTAGMUS TESTS	10030955	1
ORAL PAPULE	10031010	1
ORGANIC BRAIN SYNDROME	10031077	1
OSTEOLYSIS	10031248	1
OSTEOTOMY	10031300	1
OTITIS MEDIA CHRONIC	10033081	1

OTOSCLEROSIS	10033103	1
OVARIAN GERM CELL TERATOMA BENIGN	10033236	1
OVARIAN NECROSIS	10033270	1
OVARIAN RUPTURE	10033279	1
OVERWORK	10033308	1
OVULATION INDUCTION	10033312	1
PANCREATICODUODENECTOMY	10033644	1
PAPILLITIS	10033708	1
PAPILLOMA EXCISION	10033714	1
PARADOXICAL PRESSOR RESPONSE	10033771	1
PARALYTIC LAGOPHTHALMOS	10033842	1
PARANOID PERSONALITY DISORDER	10033869	1
PARASITE STOOL TEST POSITIVE	10033905	1
PARASITE URINE TEST NEGATIVE	10033908	1
PARATHYROID DISORDER	10033942	1
PARENT-CHILD PROBLEM	10033981	1
PARITY	10033997	1
PAROTID ABSCESS	10034019	1
PAST-POINTING	10034104	1
PATENT DUCTUS ARTERIOSUS	10034130	1
PELVIC PROLAPSE	10034268	1
PERICARDITIS ADHESIVE	10034486	1
PERINEAL HAEMATOMA	10034520	1
PERINEPHRIC ABSCESS	10034531	1
PERIPHERAL CIRCULATORY FAILURE	10034567	1
PERITONEAL ADHESIONS	10034650	1
PERSEVERATION	10034703	1
PETROLEUM DISTILLATE POISONING	10034761	1
PHILADELPHIA CHROMOSOME POSITIVE	10034877	1
PHIMOSIS	10034878	1
PIGMENTATION LIP	10035025	1
PILONIDAL CYST	10035043	1
PILONIDAL SINUS REPAIR	10035047	1
PINGUECULA	10035060	1
PITUITARY-DEPENDENT CUSHING'S SYNDROME	10035109	1
PLACENTAL TRANSFUSION SYNDROME	10035146	1
PLASMA CELL LEUKAEMIA	10035222	1
PLASMACYTOMA	10035484	1
PLASMACYTOSIS	10035485	1
PLASMODIUM VIVAX INFECTION	10035503	1
PLATELET DESTRUCTION INCREASED	10035531	1
PLEURAL CALCIFICATION	10035592	1
PLEURAL RUB	10035615	1

PNEUMONECTOMY	10035663	1
PNEUMONIA ADENOVIRAL	10035665	1
PNEUMONIA CYTOMEGALOVIRAL	10035676	1
PNEUMONIA ESCHERICHIA	10035699	1
PNEUMONIA INFLUENZAL	10035714	1
PNEUMOTHORAX TRAUMATIC	10035765	1
POLYDACTYLY	10036063	1
POLYGLANDULAR AUTOIMMUNE SYNDROME TYPE I	10036072	1
PORPHYRIA	10036181	1
PORTAL VEIN PHLEBITIS	10036205	1
POST INFLAMMATORY PIGMENTATION CHANGE	10036229	1
POST ABORTION HAEMORRHAGE	10036246	1
POSTPARTUM VENOUS THROMBOSIS	10036300	1
POTASSIUM HYDROXIDE PREPARATION NEGATIVE	10036448	1
POTASSIUM HYDROXIDE PREPARATION POSITIVE	10036449	1
POUCHITIS	10036463	1
PREMEDICATION	10036500	1
PRESENILE DEMENTIA	10036631	1
PRIAPISM	10036661	1
PRIMARY HYPOTHYROIDISM	10036697	1
PRIMARY MEDIASTINAL LARGE B-CELL LYMPHOMA	10036710	1
PRIMIPAROUS	10036758	1
PROGESTIN THERAPY	10036797	1
PROGRESSIVE BULBAR PALSY	10036800	1
PROGRESSIVE MULTIFOCAL LEUKOENCEPHALOPATHY	10036807	1
PROSTATIC SPECIFIC ANTIGEN DECREASED	10036972	1
PROTEIN ALBUMIN RATIO	10036989	1
PROTEIN ALBUMIN RATIO ABNORMAL	10036990	1
PSEUDOHYPOPARATHYROIDISM	10037126	1
PTERYGIUM	10037263	1
PULMONARY ARTERY ANEURYSM	10037336	1
PULMONARY ARTERY STENOSIS	10037338	1
PULMONARY CONTUSION	10037370	1
PULMONARY VALVE REPLACEMENT	10037449	1
PULMONARY VALVE STENOSIS	10037450	1
PULSE PRESSURE ABNORMAL	10037478	1
PULSE PRESSURE DECREASED	10037480	1
PUNCTATE KERATITIS	10037508	1
PUPILLARY LIGHT REFLEX TESTS	10037524	1
PURPURA SENILE	10037560	1
PYELOPLASTY	10037607	1
PYOGENIC GRANULOMA	10037649	1
RADIATION PNEUMONITIS	10037765	1

RADICAL HYSTERECTOMY	10037771	1
RADICULOTOMY	10037781	1
RANULA	10037838	1
REACTION TO COLOURING	10037974	1
RECTAL POLYPECTOMY	10038075	1
RECTAL PROLAPSE	10038077	1
RECTAL ULCER	10038080	1
RECTOCELE	10038084	1
RED BLOOD CELL MACROCYTES PRESENT	10038162	1
RED BLOOD CELL ROULEAUX FORMATION PRESENT	10038168	1
REFEEDING SYNDROME	10038236	1
REFRACTION DISORDER	10038264	1
RENAL ANEURYSM	10038366	1
RENAL CELL CARCINOMA RECURRENT	10038410	1
RENAL CORTICAL NECROSIS	10038422	1
RENAL DYSPLASIA	10038433	1
RENAL HAEMATOMA	10038459	1
RENAL TUBULAR ATROPHY	10038536	1
RENIN ABNORMAL	10038556	1
RETINAL CYST	10038839	1
RETINAL DEPOSITS	10038847	1
RETINITIS PIGMENTOSA	10038914	1
RETINOPATHY PROLIFERATIVE	10038934	1
RETIREMENT	10038942	1
RETROGRADE AMNESIA	10038965	1
RETROPERITONEAL ABSCESS	10038975	1
REVERSAL OF OPIATE ACTIVITY	10039004	1
RHABDOMYOMA	10039021	1
RHABDOMYOSARCOMA	10039022	1
RHINITIS PERENNIAL	10039094	1
RIB EXCISION	10039116	1
RINNE TUNING FORK TEST	10039190	1
ROULEAUX FORMATION	10039238	1
RUBELLA ANTIBODY NEGATIVE	10039257	1
RUBELLA ANTIBODY POSITIVE	10039258	1
SALIVARY DUCT STENOSIS	10039388	1
SALIVARY GLAND CANCER STAGE III	10039402	1
SALPINGO-OOPHORECTOMY	10039464	1
SALPINGO-OOPHORECTOMY UNILATERAL	10039467	1
SARCOMA	10039491	1
SARCOMA EXCISION	10039493	1
SCAR EXCISION	10039583	1
SCHISTOSOMIASIS	10039603	1

SCHOOL REFUSAL	10039660	1
SCHWANNOMA	10039667	1
SCLEROTHERAPY	10039721	1
SCROTAL SWELLING	10039759	1
SEBACEOUS CYST EXCISION	10039779	1
SELECTIVE MUTISM	10039917	1
SHOULDER DYSTOCIA	10040613	1
SICKLE CELL ANAEMIA	10040641	1
SICKLE CELL TRAIT	10040650	1
SINGLE PHOTON EMISSION COMPUTERISED TOMOGRAM NOR	10040724	1
SKIN DYSTROPHY	10040836	1
SKIN ULCER EXCISION	10040944	1
SLE ARTHRITIS	10040968	1
SLIT-LAMP TESTS NORMAL	10041034	1
SMALL CELL LUNG CANCER EXTENSIVE STAGE	10041068	1
SMALL INTESTINE GANGRENE	10041126	1
SNAKE BITE	10041230	1
SOLAR URTICARIA	10041307	1
SPINA BIFIDA	10041524	1
SPINAL ANAESTHESIA	10041536	1
SPINAL CORD INJURY CAUDA EQUINA	10041553	1
SPINE MALFORMATION	10041611	1
SPLINTER	10041662	1
SQUAMOUS CELL CARCINOMA OF THE ORAL CAVITY	10041857	1
SQUAMOUS CELL CARCINOMA OF THE TONGUE	10041865	1
STAB WOUND	10041899	1
STAPHYLOCOCCAL ABSCESS	10041917	1
STERILE PYURIA	10042010	1
STOMATITIS HAEMORRHAGIC	10042132	1
SUBCLAVIAN ARTERY EMBOLISM	10042332	1
SUPERIOR VENA CAVA SYNDROME	10042569	1
SUPEROVULATION	10042573	1
SWEAT DISCOLOURATION	10042652	1
SWEAT GLAND DISORDER	10042653	1
SYMPHYSIOLYSIS	10042747	1
SYSTEMIC MASTOCYTOSIS	10042949	1
SYSTOLIC HYPERTENSION	10042957	1
TAKAYASU'S ARTERITIS	10043097	1
TANGENTIALITY	10043114	1
TARSAL TUNNEL DECOMPRESSION	10043120	1
TATTOO EXCISION	10043145	1
TERATOMA	10043276	1
TETANUS	10043376	1

THALASSAEMIA	10043388	1
THALASSAEMIA ALPHA	10043390	1
THALASSAEMIA MINOR	10043393	1
THERAPEUTIC RESPONSE INCREASED	10043415	1
THERMOGRAM	10043420	1
THIRD STAGE POSTPARTUM HAEMORRHAGE	10043449	1
THREATENED LABOUR	10043508	1
THROMBOANGIITIS OBLITERANS	10043540	1
THROMBOSIS PROPHYLAXIS	10043634	1
THYMOMA	10043670	1
THYROID NODULE REMOVAL	10043754	1
THYROXINE ABNORMAL	10043814	1
TINEA CRURIS	10043868	1
TOE OPERATION	10043920	1
TONGUE NEOPLASM MALIGNANT STAGE UNSPECIFIED	10043966	1
TOOTH DEVELOPMENT DISORDER	10044030	1
TOOTH IMPACTED	10044042	1
TOTAL LUNG CAPACITY ABNORMAL	10044098	1
TRACHEAL CANCER	10044285	1
TRACHEAL DEVIATION	10044287	1
TRACHEAL OEDEMA	10044296	1
TRACHEOSTOMY MALFUNCTION	10044322	1
TRANSITIONAL CELL CARCINOMA	10044412	1
TRAUMATIC DELIVERY	10044520	1
TRAUMATIC SPINAL CORD COMPRESSION	10044542	1
TRI-IODOTHYRONINE UPTAKE DECREASED	10044601	1
TRICUSPID VALVE REPLACEMENT	10044641	1
TRICUSPID VALVE STENOSIS	10044642	1
TRIGEMINAL NERVE INJECTION	10044651	1
TRISOMY 13	10044686	1
TRUNCUS ARTERIOSUS PERSISTENT	10044703	1
TULARAEMIA	10045146	1
TUMOUR FLARE	10045169	1
TYMPANOPLASTY	10045217	1
ULTRASOUND EYE NORMAL	10045418	1
ULTRASOUND PROSTATE	10045431	1
ULTRASOUND SKULL NORMAL	10045444	1
UMBILICAL CORD SHORT	10045453	1
UMBILICAL HERNIA REPAIR	10045462	1
URETERIC CANCER	10046392	1
URETERIC INJURY	10046405	1
URETEROLITHOTOMY	10046412	1
URETEROSCOPY ABNORMAL	10046414	1

URETHRAL INJURY	10046454	1
URETHRAL OBSTRUCTION	10046459	1
URETHRAL SYNDROME	10046477	1
URINARY BLADDER RUPTURE	10046530	1
URINARY NITROGEN INCREASED	10046547	1
URINARY TRACT INFECTION NEONATAL	10046573	1
URINE DELTA AMINOLEVULINATE NORMAL	10046627	1
URINE ELECTROLYTES ABNORMAL	10046631	1
URINE ELECTROPHORESIS ABNORMAL	10046636	1
URINE POTASSIUM	10046660	1
URINE POTASSIUM DECREASED	10046662	1
URINE SODIUM INCREASED	10046672	1
UROGENITAL FISTULA	10046696	1
UROSTOMY	10046713	1
URTICARIA PIGMENTOSA	10046752	1
UTERINE ATONY	10046763	1
UTERINE ATROPHY	10046764	1
UTERINE INVERSION	10046796	1
UTERINE MALPOSITION	10046800	1
UTERINE POLYPECTOMY	10046813	1
VAGINAL PROLAPSE	10046940	1
VASCULAR RESISTANCE SYSTEMIC	10047105	1
VASCULAR RESISTANCE SYSTEMIC DECREASED	10047106	1
VASCULAR RESISTANCE SYSTEMIC INCREASED	10047107	1
VASOMOTOR RHINITIS	10047145	1
VENA CAVA INJURY	10047194	1
VENOOCCLUSIVE LIVER DISEASE	10047216	1
VENOUS PRESSURE JUGULAR	10047237	1
VENOUS VALVE RUPTURED	10047261	1
VENTRICULAR CISTERNOSTOMY	10047286	1
VIRAL TONSILLITIS	10047480	1
VISUAL ACUITY REDUCED TRANSIENTLY	10047532	1
VISUAL EVOKED POTENTIALS	10047547	1
VISUAL EVOKED POTENTIALS ABNORMAL	10047549	1
VITAL CAPACITY ABNORMAL	10047580	1
VITAMIN B1 DEFICIENCY	10047601	1
VITAMIN B6 ABNORMAL	10047614	1
VITAMIN B6 DECREASED	10047616	1
VITAMIN C INCREASED	10047624	1
VITAMIN C NORMAL	10047625	1
VITAMIN K DEFICIENCY	10047634	1
VITH NERVE INJURY	10047639	1
VITREOUS OPACITIES	10047658	1

VOCAL CORD POLYP	10047675	1
VOLUME BLOOD INCREASED	10047690	1
VOMITING PSYCHOGENIC	10047709	1
VTH NERVE INJURY	10047723	1
VULVAL CANCER	10047741	1
VULVAL CELLULITIS	10047752	1
VULVOVAGINITIS TRICHOMONAL	10047799	1
WANDERING PACEMAKER	10047818	1
WEBER TUNING FORK TEST	10047877	1
WERNICKE'S ENCEPHALOPATHY	10047911	1
WHITE BLOOD CELLS STOOL NEGATIVE	10047962	1
WITHDRAWAL HYPERTENSION	10048007	1
WOLFF-PARKINSON-WHITE SYNDROME	10048015	1
XANTHOMATOSIS	10048215	1
ZINC DEFICIENCY	10048259	1
VASCULITIS GASTROINTESTINAL	10048319	1
RUPTURED ECTOPIC PREGNANCY	10048407	1
GENITAL INFECTION	10048461	1
BIOPSY CHORIONIC VILLOUS ABNORMAL	10048537	1
POST THROMBOTIC SYNDROME	10048591	1
PSEUDARTHROSIS	10048617	1
FACTOR VIII INHIBITION	10048619	1
HYPEREOSINOPHILIC SYNDROME	10048643	1
AGNOSIA	10048663	1
TEAR DISCOLOURATION	10048673	1
ANTI-ERYTHROPOIETIN ANTIBODY POSITIVE	10048681	1
RHEUMATOID NODULE	10048694	1
LIPOSUCTION	10048701	1
PNEUMOCEPHALUS	10048736	1
DERMATOSIS	10048768	1
CEREBRAL HAEMANGIOMA	10048788	1
BILIARY ADENOMA	10048798	1
HYPOGLYCAEMIC SEIZURE	10048803	1
LARYNGEAL VENTRICLE PROLAPSE	10048805	1
NECROTISING SCLERITIS	10048851	1
OSTEOPATHIC TREATMENT	10048881	1
RADIATION OESOPHAGITIS	10048899	1
BOREDOM	10048909	1
FACTOR V DEFICIENCY	10048930	1
ANGIOLIPOMA	10048945	1
PARADOXICAL DRUG REACTION	10048958	1
STREPTOCOCCAL SEPSIS	10048960	1
PANCREATIC ABSCESS	10048984	1

SPINAL CORD HAEMORRHAGE	10048992	1
ENCEPHALITIS BRAIN STEM	10048997	1
ACUTE PHASE REACTION	10048998	1
ACCELERATED IDIOVENTRICULAR RHYTHM	10049003	1
ANGELMAN'S SYNDROME	10049004	1
ANTI FACTOR VIII ANTIBODY POSITIVE	10049013	1
DENTURE WEARER	10049029	1
LDL/HDL RATIO INCREASED	10049030	1
GENOTYPE DRUG RESISTANCE TEST	10049031	1
COMA ACIDOTIC	10049037	1
APPLICATION SITE INFLAMMATION	10049042	1
SPINDLE CELL SARCOMA	10049067	1
NORMAL FOETUS	10049081	1
OESOPHAGITIS ULCERATIVE	10049098	1
PREMENSTRUAL HEADACHE	10049122	1
NEUTROPENIC SEPSIS	10049151	1
RED BLOOD CELL ENZYMES ABNORMAL	10049191	1
ELECTROCARDIOGRAM ST-T SEGMENT DEPRESSION	10049224	1
ANKYLOGLOSSIA CONGENITAL	10049244	1
WOLFF-PARKINSON-WHITE SYNDROME CONGENITAL	10049291	1
GINGIVAL OEDEMA	10049305	1
PRECANCEROUS SKIN LESION	10049422	1
ANTI-ISLET CELL ANTIBODY POSITIVE	10049439	1
FIBROMATOSIS	10049444	1
URETHRAL ULCER	10049448	1
ERYTHROPOIESIS ABNORMAL	10049467	1
ERYTHROID SERIES ABNORMAL	10049472	1
URETHRAL CYST	10049502	1
BRAIN STEM AUDITORY EVOKED RESPONSE ABNORMAL	10049509	1
STOMATOCYTES PRESENT	10049536	1
AFTERBIRTH PAIN	10049589	1
UPPER RESPIRATORY TRACT INFLAMMATION	10049590	1
FUNGAL OESOPHAGITIS	10049656	1
ABDOMINAL MIGRAINE	10049714	1
METASTASES TO STOMACH	10049718	1
BLOOD HOMOCYSTEINE DECREASED	10049734	1
BLOOD HOMOCYSTEINE ABNORMAL	10049735	1
TUMOUR HAEMORRHAGE	10049750	1
PITUITARY HAEMORRHAGE	10049760	1
VENTRICULAR PRE-EXCITATION	10049761	1
NEONATAL TACHYCARDIA	10049775	1
URINARY BLADDER POLYP	10049794	1
EXTRASKELETAL OSSIFICATION	10049811	1

BONE INFARCTION	10049824	1
JOINT DISLOCATION POSTOPERATIVE	10049912	1
MEDIASTINAL HAEMATOMA	10049941	1
INTERCOSTAL NEURALGIA	10049949	1
INTRACRANIAL HYPOTENSION	10049977	1
CERVIX HAEMATOMA UTERINE	10050020	1
CERVIX HAEMORRHAGE UTERINE	10050022	1
NEONATAL HYPOXIA	10050081	1
ELECTRONEUROMYOGRAPHY	10050116	1
TEMPOROMANDIBULAR JOINT SURGERY	10050123	1
ELECTRONEUROGRAPHY	10050127	1
ADNEXA UTERI CYST	10050203	1
SKIN FIBROSIS	10050207	1
PAGET-SCHROETTER SYNDROME	10050216	1
FAECAL VOLUME DECREASED	10050248	1
CASTLEMAN'S DISEASE	10050251	1
URINE CALCIUM INCREASED	10050278	1
BODY SURFACE AREA	10050311	1
MAXIMUM HEART RATE	10050312	1
GINGIVAL OPERATION	10050338	1
OROPHARYNGEAL CANDIDIASIS	10050346	1
ELECTROCARDIOGRAM T WAVE BIPHASIC	10050382	1
ELECTROCARDIOGRAM P WAVE NORMAL	10050383	1
PERIPANCREATIC FLUID COLLECTION	10050466	1
EYELID TUMOUR	10050497	1
NEUROPATHIC ULCER	10050502	1
METASTATIC RENAL CELL CARCINOMA	10050513	1
EDENTULOUS	10050585	1
ANORECTAL OPERATION	10050622	1
BLOOD ELASTASE	10050642	1
BLOOD ERYTHROPOIETIN	10050648	1
BLOOD ERYTHROPOIETIN NORMAL	10050670	1
ANTI-INSULIN ANTIBODY	10050692	1
FALLOPIAN TUBE CYST	10050697	1
TOTAL FLUID OUTPUT	10050709	1
SELF-MEDICATION	10050729	1
ATRIAL NATRIURETIC PEPTIDE	10050734	1
BLOOD GASTRIN	10050751	1
LEUKOCYTURIA	10050791	1
GROIN INFECTION	10050821	1
CARDIOACTIVE DRUG LEVEL INCREASED	10050845	1
LIMB PROSTHESIS USER	10050861	1
FOOT PROSTHESIS USER	10050863	1

PORTAL HYPERTENSIVE GASTROPATHY	10050897	1
DECREASED VENTRICULAR AFTERLOAD	10050906	1
PROMYELOCYTE COUNT	10050968	1
URINE NITROGEN	10050970	1
LYMPHORRHOEA	10050979	1
FIBRINOUS BRONCHITIS	10051011	1
HOMANS' SIGN POSITIVE	10051031	1
EYE NAEVUS	10051045	1
RENAL SURGERY	10051061	1
PURULENT PERICARDITIS	10051071	1
PUNCTURE SITE HAEMORRHAGE	10051101	1
CD4/CD8 RATIO INCREASED	10051121	1
HYPOFIBRINOGENAEMIA	10051125	1
SCLERITIS ALLERGIC	10051126	1
FOETAL HEART RATE INCREASED	10051138	1
HYPERVISCOSITY SYNDROME	10051151	1
ELECTROCARDIOGRAM QRS COMPLEX SHORTENED	10051158	1
HEPATITIS B CORE ANTIGEN	10051160	1
OXYGEN SATURATION IMMEASURABLE	10051197	1
SPEECH REHABILITATION	10051212	1
ADENOIDITIS	10051223	1
URETERITIS	10051250	1
XANTHOGRANULOMA	10051251	1
ABDOMINAL WALL INFECTION	10051254	1
PROTEIN S DEFICIENCY	10051292	1
PROTEIN C DEFICIENCY	10051298	1
LIGAMENT CALCIFICATION	10051326	1
POST TRANSPLANT LYMPHOPROLIFERATIVE DISORDER	10051358	1
MYOFASCIAL SPASM	10051378	1
THROMBIN TIME PROLONGED	10051390	1
URINE AMPHETAMINE POSITIVE	10051400	1
CARBOHYDRATE ANTIGEN 15-3 INCREASED	10051415	1
CARBOHYDRATE ANTIGEN 27.29 INCREASED	10051419	1
THYROXIN BINDING GLOBULIN	10051424	1
BIOPSY PANCREAS	10051470	1
PROSTATOMEGALY	10051482	1
CHONDROMATOSIS	10051503	1
TRANSCRANIAL MAGNETIC STIMULATION	10051505	1
VIRAL SINUSITIS	10051513	1
VOCAL CORD CYST	10051515	1
CHVOSTEK'S SIGN	10051518	1
LEUKAPHERESIS	10051524	1
CAROTIDYNIA	10051550	1

CORNEAL BLEEDING	10051558	1
CORNEAL DEFECT	10051559	1
CHEST WALL ABSCESS	10051568	1
ABDOMINAL WALL CYST	10051634	1
AMNIORRHEXIS	10051641	1
OVARIAN FIBROSIS	10051657	1
METASTASES TO OESOPHAGUS	10051671	1
METASTASES TO TRACHEA	10051685	1
CALCIPHYLAXIS	10051714	1
FLUCTUANCE	10051723	1
WHITE BLOOD CELL SCAN	10051726	1
PERICARDIAL CYST	10051730	1
PANCREAS INFECTION	10051741	1
GALACTOGRAPHY	10051743	1
GASTRITIS VIRAL	10051791	1
BLOOD BETA-D-GLUCAN ABNORMAL	10051795	1
TRACHEAL INJURY	10051867	1
ACUTE CHEST SYNDROME	10051895	1
ADRENOMEGALY	10051896	1
PROMOTION OF PERIPHERAL CIRCULATION	10051897	1
CEREBROVASCULAR OPERATION	10051902	1
CHOLESTEROSIS	10051914	1
OESOPHAGEAL POLYP	10051935	1
OOCYTE HARVEST	10051936	1
OVARIOCENTESIS	10051939	1
PNEUMATOSIS	10051986	1
INTERNAL HERNIA	10051989	1
CORTICAL LAMINAR NECROSIS	10051990	1
LIP EROSION	10051992	1
PACEMAKER SYNDROME	10051994	1
SPONDYLOLYSIS	10052013	1
FACET JOINT BLOCK	10052018	1
NEURAL TUBE DEFECT	10052046	1
VAGAL NERVE STIMULATOR IMPLANTATION	10052077	1
VAGAL NERVE STIMULATOR REMOVAL	10052078	1
FALLOPIAN TUBE DISORDER	10052094	1
CONJUNCTIVAL BLEB	10052114	1
MYELOBLAST PERCENTAGE INCREASED	10052226	1
ALLERGY PROPHYLAXIS	10066536	1
DRY SKIN PROPHYLAXIS	10066596	1
OVARIAN CANCER RECURRENT	10066697	1
BLADDER TRANSITIONAL CELL CARCINOMA STAGE IV	10066752	1
INJECTION SITE RECALL REACTION	10066797	1

N-TELOPEPTIDE	10066811	1
ACTIVATION SYNDROME	10066817	1
EAGLE'S SYNDROME	10066835	1
TACITURNITY	10066855	1
TRICUSPID VALVE PROLAPSE	10066862	1
GALLBLADDER CANCER METASTATIC	10066879	1
EPIDEMIC POLYARTHRITIS	10066919	1
MYXOFIBROSARCOMA	10066948	1
PROCEDURAL NAUSEA	10066962	1
PROCEDURAL VOMITING	10066963	1
SUPERINFECTION BACTERIAL	10066972	1
ANAPHYLACTOID SYNDROME OF PREGNANCY	10067010	1
ENDOSCOPIC ULTRASOUND NORMAL	10067021	1
AMNIOTIC FLUID INDEX	10067078	1
HYPOGLOSSAL NERVE PARESIS	10067129	1
CONGENITAL ABDOMINAL HERNIA	10067183	1
NAEVUS FLAMMEUS	10067193	1
REBOUND TACHYCARDIA	10067207	1
UTERINE FIBROSIS	10067269	1
CEREBRAL MICROHAEMORRHAGE	10067277	1
SLEEP-RELATED EATING DISORDER	10067315	1
ARRHYTHMIC STORM	10067339	1
SPINAL MENINGEAL CYST	10067375	1
HEPATIC ANGIOSARCOMA	10067388	1
HUMAN SEMINAL PLASMA HYPERSENSITIVITY	10067432	1
ARTERIAL COMPRESSION THERAPY	10067438	1
ROTAVIRUS INFECTION	10067470	1
UHTHOFF'S PHENOMENON	10067485	1
SLEEP INERTIA	10067493	1
SEGMENTED HYALINISING VASCULITIS	10067527	1
BLOOD CREATINE PHOSPHOKINASE MB DECREASED	10067537	1
CEREBRAL SALT-WASTING SYNDROME	10067548	1
PNEUMONIA CRYPTOCOCCAL	10067565	1
NEUROGENIC HYPERTENSION	10067598	1
EPIDURAL LIPOMATOSIS	10067599	1
CARTILAGE ATROPHY	10067632	1
SYSTEMIC LUPUS ERYTHEMATOSUS DISEASE ACTIVITY INDEX I	10067657	1
PREGNANCY ON CONTRACEPTIVE	10067667	1
IRIS BOMBE	10067684	1
EX-DRUG ABUSER	10067689	1
OPHTHALMIC FLUID-AIR EXCHANGE PROCEDURE	10067701	1
INTRAPARTUM HAEMORRHAGE	10067703	1
HYPERLIPASAEMIA	10067725	1

UMBILICAL GRANULOMA	10067731	1
UTERINE CERVIX STENOSIS	10067732	1
COAGULATION FACTOR DEFICIENCY	10067787	1
ABDOMINAL WALL HAEMORRHAGE	10067788	1
COMMUNITY ACQUIRED INFECTION	10067822	1
SPLENIC VARICES	10067823	1
ENDOBRONCHIAL VALVE IMPLANTATION	10067851	1
GASTRIC OCCULT BLOOD POSITIVE	10067855	1
NEUROSURGERY	10067908	1
LONG THORACIC NERVE PALSY	10067925	1
EASTERN COOPERATIVE ONCOLOGY GROUP PERFORMANCE ST	10067961	1
AORTIC INTRAMURAL HAEMATOMA	10067975	1
INJECTION SITE VASCULITIS	10067995	1
COMPULSIVE HOARDING	10068007	1
TRIGEMINAL NERVE PARESIS	10068008	1
THERMOHYPOAESTHESIA	10068009	1
TEMPERATURE PERCEPTION TEST ABNORMAL	10068013	1
TEMPERATURE PERCEPTION TEST INCREASED	10068014	1
PROBIOTIC THERAPY	10068048	1
DEPENDENT RUBOR	10068057	1
ACCESSORY SPLEEN	10068059	1
THYROID HORMONE REPLACEMENT THERAPY	10068076	1
UVEITIS-GLAUCOMA-HYPHAEMA SYNDROME	10068148	1
ATRIOVENTRICULAR CONDUCTION TIME SHORTENED	10068180	1
AUTONOMIC DYSREFLEXIA	10068196	1
ANTIANGIOGENIC THERAPY	10068301	1
URETHRAL ATROPHY	10068313	1
CARDIOTHORACIC RATIO	10068336	1
HEPATITIS C RNA INCREASED	10068377	1
VENOUS OXYGEN PARTIAL PRESSURE	10068421	1
VENOUS OXYGEN SATURATION INCREASED	10068426	1
IDIOPATHIC GUTTATE HYPOMELANOSIS	10068489	1
NATURAL KILLER CELL COUNT INCREASED	10068496	1
NATURAL KILLER T CELL COUNT	10068498	1
NATURAL KILLER T CELL COUNT INCREASED	10068499	1
SOFT TISSUE ATROPHY	10068548	1
ANAL FUNGAL INFECTION	10068556	1
BREAST SARCOMA	10068582	1
PERITONEAL FLUID ANALYSIS NORMAL	10068624	1
CHRONOTROPIC INCOMPETENCE	10068627	1
PROSTHETIC VESSEL IMPLANTATION	10068628	1
DOWLING-DEGOS DISEASE	10068651	1
NAIL INJURY	10068655	1

LIVER SARCOIDOSIS	10068664	1
PULMONARY VEIN OCCLUSION	10068690	1
UMBILICAL HAEMATOMA	10068712	1
HEPATITIS D VIRUS TEST	10068787	1
ANTISYNTHETASE SYNDROME	10068801	1
WEST NILE VIRUS TEST POSITIVE	10068812	1
CONJUNCTIVAL PALLOR	10068819	1
PANCREATIC CALCIFICATION	10068823	1
FUNGATING WOUND	10068834	1
AIRWAY PEAK PRESSURE INCREASED	10068853	1
URINARY STONE ANALYSIS	10068860	1
ANGIOSCOPY	10068866	1
GENITAL HYPOAESTHESIA	10068912	1
ANORECTAL VARICES	10068924	1
GASTROINTESTINAL EXAMINATION ABNORMAL	10068977	1
VON WILLEBRAND'S FACTOR ACTIVITY INCREASED	10068987	1
VON WILLEBRAND'S FACTOR ANTIGEN ABNORMAL	10068989	1
TUBULOINTERSTITIAL NEPHRITIS AND UVEITIS SYNDROME	10069034	1
HISTIOCYTIC NECROTISING LYMPHADENITIS	10069070	1
ATROPHIC GLOSSITIS	10069085	1
HEPATIC VEIN DILATATION	10069112	1
TRACHEOBRONCHIAL STENT INSERTION	10069120	1
MYOCARDIAL DEPRESSION	10069140	1
ALDOLASE ABNORMAL	10069154	1
ABNORMAL WITHDRAWAL BLEEDING	10069195	1
PRODUCT IDENTIFICATION NUMBER ISSUE	10069267	1
PHYSICAL PRODUCT LABEL ISSUE	10069272	1
PRODUCT CLOSURE ISSUE	10069295	1
ACUTE LUNG INJURY	10069351	1
PRODUCT PACKAGING ISSUE	10069405	1
LYMPHATIC FISTULA	10069421	1
HASHIMOTO'S ENCEPHALOPATHY	10069432	1
BONE GRAFT REMOVAL	10069436	1
HENOCH-SCHONLEIN PURPURA NEPHRITIS	10069440	1
LYMPHATIC DUCT INJURY	10069449	1
HYPOGLOSSAL NERVE PARALYSIS	10069450	1
ACQUIRED VON WILLEBRAND'S DISEASE	10069495	1
INTRAPERICARDIAL THROMBOSIS	10069550	1
BRAIN MIDLINE SHIFT	10069551	1
ATRIOVENTRICULAR DISSOCIATION	10069571	1
PNEUMOCOCCAL IMMUNISATION	10069578	1
VACCINATION SITE ULCER	10069621	1
CAMPTOCORMIA	10069646	1

URINARY STRAINING	10069648	1
MESENTERITIS	10069665	1
HTLV-1 CARRIER	10069712	1
NEUROTROPHIC KERATOPATHY	10069732	1
INTERNAL FIXATION OF SPINE	10069749	1
THYROID DERMATOPATHY	10069771	1
RESPIRATORY SYNCYTIAL VIRUS BRONCHITIS	10069811	1
NEUROLOGICAL COMPLICATION ASSOCIATED WITH DEVICE	10069836	1
STENT MALFUNCTION	10069862	1
ANTIRIBOSOMAL P ANTIBODY POSITIVE	10069910	1
RENAL ARTERY STENT REMOVAL	10069948	1
BILE DUCT STENT REMOVAL	10069951	1
ACTINOMYCES TEST POSITIVE	10069956	1
BACTEROIDES TEST POSITIVE	10069958	1
MICROCOCCUS TEST POSITIVE	10070001	1
PEPTOSTREPTOCOCCUS TEST POSITIVE	10070003	1
PROPIONIBACTERIUM TEST POSITIVE	10070004	1
STENOTROPHOMONAS TEST POSITIVE	10070006	1
PANTOEA AGGLOMERANS TEST POSITIVE	10070019	1
BRUCELLA TEST POSITIVE	10070021	1
CAMPYLOBACTER TEST POSITIVE	10070025	1
CORYNEBACTERIUM TEST POSITIVE	10070028	1
LEGIONELLA TEST POSITIVE	10070092	1
LISTERIA TEST POSITIVE	10070094	1
SHIGELLA TEST POSITIVE	10070129	1
DENGUE VIRUS TEST POSITIVE	10070225	1
VIRAL TITRE DECREASED	10070251	1
METHYLENETETRAHYDROFOLATE REDUCTASE DEFICIENCY	10070309	1
AMINOPYRINE BREATHING TEST	10070311	1
TRANSIENT ELASTOGRAPHY	10070321	1
APHTHOVIRUS TEST POSITIVE	10070340	1
PNEUMOVIRUS TEST POSITIVE	10070345	1
UNEVALUABLE INVESTIGATION	10070435	1
PNEUMOCYSTIS TEST POSITIVE	10070454	1
ARTHROTOMY	10070463	1
DRUG ADMINISTERED IN WRONG DEVICE	10070470	1
AQUEOUS HUMOUR LEAKAGE	10070497	1
TRAUMATIC RENAL INJURY	10070510	1
POST STROKE DEPRESSION	10070606	1
VESSEL PUNCTURE SITE INFLAMMATION	10070647	1
CORTICAL DYSPLASIA	10070666	1
HUNTINGTON'S DISEASE	10070668	1
FALSE NEGATIVE INVESTIGATION RESULT	10070747	1

THERAPEUTIC NERVE ABLATION	10070755	1
BONE DECALCIFICATION	10070817	1
REACTIVE AIRWAYS DYSFUNCTION SYNDROME	10070832	1
PULMONARY SENSITISATION	10070838	1
UTERINE LEIOMYOMA EMBOLISATION	10070867	1
JOINT LAXITY	10070874	1
MALARIA ANTIBODY TEST POSITIVE	10070881	1
OVARIAN CANCER STAGE III	10070907	1
METASTASES TO PELVIS	10070913	1
PULMONARY PHYSICAL EXAMINATION ABNORMAL	10070936	1
PULMONARY PHYSICAL EXAMINATION NORMAL	10070937	1
ANAL EXAMINATION ABNORMAL	10070942	1
METASTATIC CUTANEOUS CROHN'S DISEASE	10070949	1
COLPOSCOPY NORMAL	10070980	1
VASCULAR COMPRESSION	10070995	1
INTRADUCTAL PAPILLARY MUCINOUS NEOPLASM	10070999	1
CREATININE URINE NORMAL	10071020	1
CREATININE URINE ABNORMAL	10071021	1
VITH NERVE PARESIS	10071044	1
ANKLE DEFORMITY	10071050	1
EYE HAEMANGIOMA	10071053	1
ENDOBRONCHIAL ULTRASOUND	10071063	1
OVARIAN CALCIFICATION	10071064	1
AUTOPHOBIA	10071070	1
SUBCUTANEOUS BIOPSY ABNORMAL	10071140	1
OPTIC DISC HYPERAEMIA	10071196	1
TROUSSEAU'S SIGN	10071284	1
SUSPECTED COUNTERFEIT PRODUCT	10071287	1
ULTRASOUND URINARY SYSTEM	10071295	1
MELANOCYTIC HYPERPLASIA	10071308	1
SPINAL ARTERY THROMBOSIS	10071316	1
CENTRAL NERVOUS SYSTEM FUNCTION TEST	10071317	1
CARBOHYDRATE ANTIGEN 125 NORMAL	10071318	1
NEUROPSYCHIATRIC SYNDROME	10071323	1
URINE MAGNESIUM	10071357	1
SALIVARY GLAND INDURATION	10071363	1
PSYCHOLOGICAL TRAUMA	10071368	1
LENS EXTRACTION	10071370	1
EXPANDED DISABILITY STATUS SCALE	10071383	1
RETINAL FIBROSIS	10071391	1
VANISHING TWIN SYNDROME	10071398	1
AXIAL SPONDYLOARTHRITIS	10071400	1
MYCOBACTERIUM CHELONAE INFECTION	10071401	1

EXPOSURE VIA FATHER	10071403	1
MATERNAL EXPOSURE DURING DELIVERY	10071407	1
BRACHIORADIAL PRURITUS	10071443	1
ANTI-NEURONAL ANTIBODY	10071463	1
ANTI-THROMBIN ANTIBODY	10071465	1
ANTI-NMDA ANTIBODY	10071468	1
ANTI-ZINC TRANSPORTER 8 ANTIBODY	10071471	1
CRYSTAL NEPHROPATHY	10071503	1
FOETAL MONITORING ABNORMAL	10071507	1
CEREBRAL REVASCULARISATION	10071508	1
MESENTERIC HAEMATOMA	10071557	1
FLUID WAVE TEST	10071565	1
SUSAC'S SYNDROME	10071573	1
ANAPLASTIC LYMPHOMA RECEPTOR TYROSINE KINASE ASSAY	10071593	1
TRANSMEMBRANE RECEPTOR TYROSINE KINASE ASSAY	10071598	1
GENETIC POLYMORPHISM	10071602	1
FAECAL ELASTASE CONCENTRATION DECREASED	10071656	1
N-TERMINAL PROHORMONE BRAIN NATRIURETIC PEPTIDE AB	10071660	1
DENTAL LEAKAGE	10071694	1
FOWLER'S SYNDROME	10071718	1
DEVICE FUNCTION TEST	10071743	1
MILK SOY PROTEIN INTOLERANCE	10071754	1
VIRAL MYELITIS	10071763	1
NEURONE-SPECIFIC ENOLASE INCREASED	10071929	1
SUBRETINAL HAEMATOMA	10071935	1
CYP2C19 GENE STATUS ASSAY	10071965	1
BRCA2 GENE MUTATION ASSAY	10071970	1
ENZYME ACTIVITY INCREASED	10072021	1
MESENTERIC NEOPLASM	10072025	1
CENTRAL NERVOUS SYSTEM HAEMORRHAGE	10072043	1
PERINEPHRITIS	10072058	1
ARTIFICIAL SKIN GRAFT	10072067	1
GLUCOCORTICOID DEFICIENCY	10072079	1
BLADDER WALL CALCIFICATION	10072140	1
PAEDIATRIC AUTOIMMUNE NEUROPSYCHIATRIC DISORDERS A	10072147	1
MENISCUS CYST	10072157	1
PUNCTURE SITE SWELLING	10072161	1
MYOCARDIAL STUNNING	10072186	1
BLADDER INSTILLATION PROCEDURE	10072199	1
FASCIAL RUPTURE	10072209	1
GENITAL HERPES ZOSTER	10072210	1
POSTERIOR FOSSA DECOMPRESSION	10072283	1
X-RAY WITH CONTRAST	10072302	1

AMYLASE ABNORMAL	10072327	1
LEUKOTRIENE TEST	10072335	1
END-TIDAL CO2	10072374	1
ASYMMETRIC DI-METHYLARGININE INCREASED	10072403	1
RED BLOOD CELL ANALYSIS ABNORMAL	10072410	1
DELIVERY OUTSIDE HEALTH FACILITY	10072446	1
FLUORESCENT IN SITU HYBRIDISATION POSITIVE	10072468	1
GESTATIONAL AGE TEST ABNORMAL	10072479	1
NASOGASTRIC OUTPUT ABNORMAL	10072487	1
ANTI-IA2 ANTIBODY POSITIVE	10072498	1
ANTI-VGCC ANTIBODY POSITIVE	10072502	1
ANTI-ACTIN ANTIBODY POSITIVE	10072506	1
ANTI-GLOMERULAR BASEMENT MEMBRANE ANTIBODY NEGA	10072517	1
ANTI-ISLET CELL ANTIBODY NEGATIVE	10072520	1
ANTI-ZINC TRANSPORTER 8 ANTIBODY NEGATIVE	10072529	1
ANTI-ZINC TRANSPORTER 8 ANTIBODY POSITIVE	10072530	1
CAROTID REVASCULARISATION	10072559	1
RECTAL INJURY	10072587	1
UMBILICAL DISCHARGE	10072595	1
INADEQUATE ASEPTIC TECHNIQUE IN USE OF PRODUCT	10072608	1
ORTHOPAEDIC EXAMINATION NORMAL	10072626	1
INTRAHEPATIC PORTAL HEPATIC VENOUS FISTULA	10072629	1
NOTALGIA PARAESTHETICA	10072643	1
VERY LONG-CHAIN ACYL-COENZYME A DEHYDROGENASE DEFI	10072656	1
TOOTH DEMINERALISATION	10072665	1
FIBROBLAST GROWTH FACTOR 23 INCREASED	10072680	1
UVEITIC GLAUCOMA	10072686	1
HEPATITIS VIRAL TEST POSITIVE	10072748	1
CD4 LYMPHOCYTE PERCENTAGE DECREASED	10072798	1
BREAST CYST RUPTURE	10072812	1
MONONUCLEAR CELL COUNT ABNORMAL	10072856	1
LYMPHATIC MAPPING	10072871	1
DEVICE USE ERROR	10072878	1
URINARY BLADDER TOXICITY	10072996	1
PROGRESSIVE FACIAL HEMIATROPHY	10073006	1
MALIGNANT NEOPLASM OF UNKNOWN PRIMARY SITE	10073059	1
GALLBLADDER NEOPLASM	10073072	1
TUBULAR BREAST CARCINOMA	10073104	1
PLASMA CELL MYELOMA RECURRENT	10073133	1
DEDIFFERENTIATED LIPOSARCOMA	10073135	1
SOLUBLE FIBRIN MONOMER COMPLEX	10073165	1
PYLORIC SPHINCTER INSUFFICIENCY	10073166	1
INDUCTION OF CERVIX RIPENING	10073175	1

CORTICOTROPIN-RELEASING HORMONE STIMULATION TEST	10073178	1
ADRENOCORTICOTROPIC HORMONE DEFICIENCY	10073179	1
BOOSTER DOSE MISSED	10073205	1
DYSTONIC TREMOR	10073210	1
POSTURAL TREMOR	10073211	1
PERIPANCREATIC VARICES	10073215	1
HEPATITIS A ANTIGEN NEGATIVE	10073225	1
IDIOPATHIC ANGIOEDEMA	10073257	1
BRENNER TUMOUR	10073258	1
OVARIAN GRANULOSA CELL TUMOUR	10073260	1
EXPOSURE VIA BODY FLUID	10073300	1
EXPOSURE VIA CONTAMINATED DEVICE	10073301	1
EXPOSURE TO RADIATION	10073306	1
EXPOSURE TO CONTAMINATED WATER	10073309	1
ACCIDENTAL EXPOSURE TO PRODUCT BY CHILD	10073318	1
WHITE BLOOD CELL ANALYSIS NORMAL	10073322	1
DECORTICATE POSTURE	10073345	1
GENITAL CONTUSION	10073355	1
ADENOCARCINOMA OF APPENDIX	10073359	1
APPENDIX CANCER	10073360	1
SMALL INTESTINE ADENOCARCINOMA	10073373	1
LARGE INTESTINE BENIGN NEOPLASM	10073394	1
CD4 LYMPHOCYTES NORMAL	10073408	1
INCISION SITE RASH	10073411	1
INJECTION SITE HYPERAESTHESIA	10073418	1
BLOOD TRYPSIN	10073447	1
INFLUENZA C VIRUS TEST	10073451	1
APPLICATION SITE ACNE	10073457	1
PROSTHETIC CARDIAC VALVE MALFUNCTION	10073511	1
INTERFERON GAMMA RELEASE ASSAY	10073542	1
SPINAL SUBARACHNOID HAEMORRHAGE	10073564	1
EXPIRED DEVICE USED	10073594	1
INSTILLATION SITE EXFOLIATION	10073601	1
INSTILLATION SITE HAEMORRHAGE	10073610	1
INSTILLATION SITE INDURATION	10073613	1
INSTILLATION SITE WARMTH	10073629	1
FOETAL MEGACYSTIS	10073660	1
CEREBRAL VENTRICLE COLLAPSE	10073706	1
ADENOSINE DEAMINASE INCREASED	10073725	1
HORMONAL CONTRACEPTION	10073728	1
GENITALS ENLARGED	10073740	1
THUMB SUCKING	10073746	1
INHIBITING ANTIBODIES POSITIVE	10073771	1

STROKE-LIKE MIGRAINE ATTACKS AFTER RADIATION THERAPY	10073775	1
APPLICATION SITE LACERATION	10073779	1
OSTEOCHONDRAL FRACTURE	10073853	1
PREICTAL STATE	10073854	1
KETOGENIC DIET	10073855	1
RADIOEMBOLISATION	10073946	1
PROSOPAGNOSIA	10073980	1
ARTERIAL SEGMENTAL PRESSURE TEST	10073986	1
PROCEDURAL INTESTINAL PERFORATION	10074065	1
PUNCTURE SITE OEDEMA	10074069	1
OESOPHAGEAL COMPRESSION	10074074	1
BILIARY ASCITES	10074150	1
DRY LUNG SYNDROME	10074163	1
CRANIOFACIAL DEFORMITY	10074180	1
TISSUE EXPANSION PROCEDURE	10074186	1
VOLVULUS REPAIR	10074224	1
HERPES SIMPLEX GASTRITIS	10074240	1
HERPES SIMPLEX PHARYNGITIS	10074244	1
HERPES ZOSTER PHARYNGITIS	10074245	1
HERPES SIMPLEX MENINGOENCEPHALITIS	10074247	1
HERPES ZOSTER MENINGOENCEPHALITIS	10074248	1
HERPES ZOSTER MENINGOMYELITIS	10074251	1
HERPES ZOSTER MENINGITIS	10074259	1
LYMPH NODE HAEMORRHAGE	10074270	1
RIGHT VENTRICULAR HYPERTENSION	10074301	1
ANTI B ANTIBODY	10074304	1
FEV1/FVC RATIO	10074315	1
FORCED EXPIRATORY FLOW	10074316	1
CARDIOPULMONARY EXERCISE TEST ABNORMAL	10074359	1
SACROILIAC FRACTURE	10074362	1
RED BREAST SYNDROME	10074453	1
UTERINE MYOMA EXPULSION	10074467	1
TRAUMATIC HAEMOTHORAX	10074487	1
ADAMTS13 ACTIVITY DECREASED	10074491	1
ECTOPIC PREGNANCY WITH CONTRACEPTIVE DEVICE	10074497	1
BODY MASS INDEX ABNORMAL	10074506	1
STOMA SITE ERYTHEMA	10074514	1
UTERINE SCAR	10074527	1
ANTICOAGULATION DRUG LEVEL INCREASED	10074528	1
HEPATIC FIBROSIS MARKER TEST	10074593	1
COLLOID BRAIN CYST	10074613	1
NONREASSURING FOETAL HEART RATE PATTERN	10074641	1
FOETAL HEART RATE ACCELERATION ABNORMALITY	10074642	1

NONINFECTIVE CHORIORETINITIS	10074696	1
INFECTIVE IRITIS	10074698	1
FORCED VITAL CAPACITY DECREASED	10074722	1
PORTAL FIBROSIS	10074726	1
BLOOD VISCOSITY DECREASED	10074727	1
BLOOD VISCOSITY ABNORMAL	10074728	1
NECROTISING MYOSITIS	10074769	1
TONGUE POLYP	10074778	1
ADMINISTRATION SITE ERYTHEMA	10074796	1
UNHEALTHY DIET	10074834	1
TRANSPLANT DYSFUNCTION	10074860	1
PREVERTEBRAL SOFT TISSUE SWELLING OF CERVICAL SPACE	10074880	1
EXPULSION OF MEDICATION	10074905	1
APNOEA TEST ABNORMAL	10074913	1
THERAPEUTIC RESPONSE CHANGED	10074941	1
URINARY TRACT STOMA COMPLICATION	10074942	1
VASCULAR MALFORMATION	10074979	1
WAIST CIRCUMFERENCE	10074998	1
ABNORMAL INVOLUNTARY MOVEMENT SCALE	10075002	1
ATYPICAL MYCOBACTERIAL LOWER RESPIRATORY TRACT INFEC	10075026	1
POLYOMAVIRUS TEST	10075038	1
BK POLYOMAVIRUS TEST	10075041	1
ASPIRIN-EXACERBATED RESPIRATORY DISEASE	10075084	1
HEPATITIS E VIRUS TEST	10075085	1
ADMINISTRATION SITE CELLULITIS	10075095	1
ADMINISTRATION SITE HYPERSENSITIVITY	10075102	1
ADMINISTRATION SITE ODOUR	10075103	1
ADMINISTRATION SITE PRURITUS	10075106	1
MODEL FOR END STAGE LIVER DISEASE SCORE	10075139	1
FRACTIONAL EXCRETION OF SODIUM	10075142	1
ANTI-MUELLERIAN HORMONE LEVEL INCREASED	10075157	1
ANTI-MUELLERIAN HORMONE LEVEL NORMAL	10075159	1
VISUAL ANALOGUE SCALE	10075166	1
PROCEDURAL ANXIETY	10075204	1
CEREBRAL VENTRICULAR RUPTURE	10075249	1
TENDON SHEATH DISORDER	10075252	1
ACTIVATED PARTIAL THROMBOPLASTIN TIME RATIO	10075283	1
ACTIVATED PARTIAL THROMBOPLASTIN TIME RATIO NORMAL	10075288	1
VENTRICULAR REMODELLING	10075291	1
TARGETED CANCER THERAPY	10075304	1
BLOOD BILIRUBIN UNCONJUGATED DECREASED	10075327	1
PORTAL TRACT INFLAMMATION	10075331	1
SOFT TISSUE SARCOMA	10075333	1

CHOLANGITIS INFECTIVE	10075445	1
BRACHIOCEPHALIC ARTERY STENOSIS	10075450	1
STOMA SITE EXTRAVASATION	10075511	1
ACINETOBACTER TEST	10075547	1
MEDICAL DEVICE SITE ERYTHEMA	10075576	1
MEDICAL DEVICE SITE INFLAMMATION	10075581	1
MEDICAL DEVICE SITE SWELLING	10075586	1
ANTRAL FOLLICLE COUNT	10075595	1
COLUMBIA SUICIDE SEVERITY RATING SCALE	10075600	1
ALLERGY ALERT TEST NEGATIVE	10075601	1
CEREBROSPINAL FLUID CIRCULATION DISORDER	10075604	1
CD25 ANTIGEN POSITIVE	10075649	1
CEREBRAL ARTERY PERFORATION	10075734	1
BASOPHILOPENIA	10075813	1
HUMAN HERPES VIRUS 8 TEST	10075833	1
NASAL ADHESIONS	10075834	1
TONGUE FUNGAL INFECTION	10075845	1
AMNIOTIC FLUID INDEX INCREASED	10075867	1
FIDUCIAL MARKER PLACEMENT	10075872	1
ADMINISTRATION SITE CALCIFICATION	10075925	1
ADMINISTRATION SITE DYSAESTHESIA	10075930	1
ADMINISTRATION SITE INDURATION	10075939	1
ADMINISTRATION SITE NERVE DAMAGE	10075957	1
ADMINISTRATION SITE PAPULE	10075959	1
NEOBLADDER SURGERY	10075996	1
APPLICATION SITE COLDNESS	10076001	1
APPLICATION SITE MOVEMENT IMPAIRMENT	10076020	1
APPLICATION SITE NERVE DAMAGE	10076021	1
APPLICATION SITE PLAQUE	10076023	1
SUSTAINED VIRAL RESPONSE	10076041	1
COMPUTED TOMOGRAPHIC ABSCESSOGRAM	10076045	1
SPINAL CORD HAEMATOMA	10076051	1
CLINICAL TRIAL PARTICIPANT	10076055	1
EYELID MYOCLONUS	10076060	1
INFUSION SITE JOINT SWELLING	10076078	1
INFUSION SITE STREAKING	10076086	1
MEDICAL DEVICE SITE EROSION	10076104	1
MEDICAL DEVICE SITE JOINT INFECTION	10076118	1
MEDICAL DEVICE SITE JOINT PAIN	10076121	1
MEDICAL DEVICE SITE JOINT SWELLING	10076123	1
MEDICAL DEVICE SITE THROMBOSIS	10076145	1
MEDICAL DEVICE SITE WARMTH	10076148	1
VACCINATION SITE EROSION	10076162	1

VACCINATION SITE ISCHAEMIA	10076170	1
VACCINATION SITE JOINT DISCOMFORT	10076171	1
VACCINATION SITE JOINT EFFUSION	10076172	1
VACCINATION SITE JOINT WARMTH	10076179	1
VACCINATION SITE LACERATION	10076180	1
VACCINATION SITE PHLEBITIS	10076185	1
VACCINATION SITE PHOTOSENSITIVITY REACTION	10076186	1
VACCINATION SITE THROMBOSIS	10076190	1
INTESTINAL ANGIOEDEMA	10076229	1
FLUID BALANCE ASSESSMENT	10076266	1
FLUID BALANCE NEGATIVE	10076268	1
SYNOVIAL BIOPSY ABNORMAL	10076280	1
CENTRAL NERVOUS SYSTEM LUPUS	10076328	1
STEATOHEPATITIS	10076331	1
ENDOSCOPIC SWALLOWING EVALUATION	10076372	1
INCARCERATED INCISIONAL HERNIA	10076387	1
ORAL HYPERAESTHESIA	10076390	1
CHRONIC PAPILLOMATOUS DERMATITIS	10076454	1
HYPERACTIVE PHARYNGEAL REFLEX	10076460	1
DOCUMENTED HYPERSENSITIVITY TO ADMINISTERED PRODUC	10076470	1
INABILITY TO AFFORD MEDICATION	10076503	1
COMPULSIVE CHEEK BITING	10076510	1
FUNGAL PHARYNGITIS	10076516	1
SCHISTOCYTOSIS	10076534	1
ERYTHEMATOTELANGIECTATIC ROSACEA	10076536	1
PAPULOPUSTULAR ROSACEA	10076537	1
PNEUMOCYSTIS TEST NEGATIVE	10076540	1
DERMAL FILLER OVERCORRECTION	10076572	1
LASER BRAIN ABLATION	10076587	1
ULCERATIVE GASTRITIS	10076599	1
MAST CELL DEGRANULATION PRESENT	10076606	1
PRESCRIPTION DRUG USED WITHOUT A PRESCRIPTION	10076639	1
AUTOIMMUNE HYPOTHYROIDISM	10076644	1
DISSEMINATED VARICELLA ZOSTER VACCINE VIRUS INFECTION	10076667	1
MIGRAINE-TRIGGERED SEIZURE	10076676	1
PREMATURE BABY DEATH	10076700	1
SUBARACHNOID HAEMATOMA	10076701	1
DEVELOPMENTAL REGRESSION	10076709	1
URETERIC COMPRESSION	10076715	1
OPEN GLOBE INJURY	10076717	1
AMEGAKARYOCYTIC THROMBOCYTOPENIA	10076744	1
ADAMTS13 INHIBITOR SCREEN ASSAY	10076767	1
POST-TRAUMATIC NEURALGIA	10076781	1

NASAL SINUS IRRIGATION	10076904	1
SEZARY CELL COUNT	10076961	1
CHA2DS2-VASC ANNUAL STROKE RISK HIGH	10076968	1
SYSTOLIC ANTERIOR MOTION OF MITRAL VALVE	10076976	1
JC POLYOMAVIRUS TEST NEGATIVE	10076979	1
BICKERSTAFF'S ENCEPHALITIS	10076985	1
NEUROENDOSCOPY	10076990	1
HYPERTENSIVE CEREBROVASCULAR DISEASE	10077000	1
POSTPARTUM THROMBOSIS	10077022	1
MASTICATORY PAIN	10077030	1
ILIAC ARTERY DISEASE	10077115	1
SYSTEMIC INFECTION	10077116	1
FAILED IN VITRO FERTILISATION	10077207	1
DEFIANT BEHAVIOUR	10077244	1
SURGICAL FIXATION OF RIB FRACTURE	10077270	1
RAPID EYE MOVEMENT SLEEP BEHAVIOUR DISORDER	10077299	1
ACUTE ON CHRONIC LIVER FAILURE	10077305	1
PELVIC FLOOR MUSCLE TRAINING	10077309	1
DOPAMINE TRANSPORTER SCINTIGRAPHY	10077317	1
INFANTILE APNOEA	10077321	1
TICK PARALYSIS	10077336	1
EPILEPTIC ENCEPHALOPATHY	10077380	1
SUICIDE THREAT	10077417	1
HAEMORRHAGIC BREAST CYST	10077443	1
PORTAL SHUNT PROCEDURE	10077479	1
PSEUDOFOLLICULITIS	10077520	1
SOMATIC DYSFUNCTION	10077554	1
FOETAL MOVEMENT DISORDER	10077576	1
FOETAL ANAEMIA	10077577	1
FOETAL GASTROINTESTINAL TRACT IMAGING ABNORMAL	10077579	1
FOETAL RENAL IMAGING ABNORMAL	10077581	1
BLOCH-SULZBERGER SYNDROME	10077624	1
ANTI FACTOR X ACTIVITY INCREASED	10077671	1
PAO2/FIO2 RATIO	10077710	1
SPINAL FLATTENING	10077756	1
VASCULAR ACCESS SITE BRUISING	10077767	1
VASCULAR ACCESS PLACEMENT	10077814	1
QUADRANTANOPIA	10077820	1
OESOPHAGEAL MUCOSAL TEAR	10077822	1
OMENTAL NECROSIS	10077828	1
VISCERAL VENOUS THROMBOSIS	10077829	1
VASCULAR ACCESS MALFUNCTION	10077832	1
LEFT-TO-RIGHT CARDIAC SHUNT	10077834	1

TOTAL COMPLEMENT ACTIVITY DECREASED	10077883	1
CARDIAC STEATOSIS	10077905	1
ARACHNOID WEB	10077932	1
ABDOMINAL FAT APRON	10077983	1
NEUROSARCOIDOSIS	10078011	1
INTERSPINOUS OSTEOARTHRITIS	10078014	1
SELECTIVE EATING DISORDER	10078069	1
GAMBLING DISORDER	10078070	1
GENDER DYSPHORIA	10078075	1
PSEUDOSTROKE	10078090	1
THERAPY PARTIAL RESPONDER	10078115	1
HAEMOSIDERIN STAIN	10078154	1
HIV-ASSOCIATED NEUROCOGNITIVE DISORDER	10078164	1
PEAK NASAL INSPIRATORY FLOW TEST	10078180	1
ZIKA VIRUS INFECTION	10078205	1
LIGAMENTUM FLAVUM HYPERTROPHY	10078213	1
RENAL GRAFT INFECTION	10078229	1
DENTAL DYSAESTHESIA	10078275	1
FLAVIVIRUS TEST	10078302	1
BIOPSY GALLBLADDER ABNORMAL	10078309	1
CEREBRAL MICROEMBOLISM	10078311	1
SYMMETRICAL DRUG-RELATED INTERTRIGINOUS AND FLEXUR	10078325	1
CHOLELITHOTRIPSY	10078327	1
NIPPLE ENLARGEMENT	10078337	1
RISK OF FUTURE PREGNANCY MISCARRIAGE	10078342	1
B-CELL DEPLETION THERAPY	10078393	1
BENIGN ENLARGEMENT OF THE SUBARACHNOID SPACES	10078477	1
DISCONTINUED PRODUCT ADMINISTERED	10078505	1
B-LYMPHOCYTE COUNT ABNORMAL	10078589	1
CD19 LYMPHOCYTE COUNT ABNORMAL	10078591	1
BACTERIAL ABDOMINAL INFECTION	10078660	1
VOCAL CORD AUGMENTATION	10078752	1
EXPOSURE VIA UNKNOWN ROUTE	10078762	1
MYSOPHOBIA	10078769	1
TUMOUR INFLAMMATION	10078786	1
PERINEPHRIC OEDEMA	10078818	1
AMNIOTIC MEMBRANE RUPTURE TEST NEGATIVE	10078835	1
AMNIOTIC MEMBRANE RUPTURE TEST	10078836	1
HAEMORRHAGIC VARICELLA SYNDROME	10078873	1
ARTIFICIAL BLOOD VESSEL OCCLUSION	10078895	1
SWOLLEN JOINT COUNT	10078912	1
SUPINE HYPERTENSION	10078932	1
EXPOSURE KERATITIS	10078951	1

BREAST PROLIFERATIVE CHANGES	10078952	1
IMMUNE-MEDIATED NEUROPATHY	10078963	1
INVESTIGATION NONCOMPLIANCE	10078982	1
PRODUCT SUBSTITUTION ERROR	10078984	1
PRIMARY HEADACHE ASSOCIATED WITH SEXUAL ACTIVITY	10079056	1
AUTOIMMUNE PERICARDITIS	10079058	1
PEDANTIC SPEECH	10079059	1
OROPHARYNGEAL DISCOLOURATION	10079096	1
RESPIRATORY SINUS ARRHYTHMIA MAGNITUDE ABNORMAL	10079117	1
TRANSVALVULAR PRESSURE GRADIENT ABNORMAL	10079127	1
SEGMENTAL DIVERTICULAR COLITIS	10079166	1
MEDICAL DEVICE SITE BURN	10079169	1
MYOPIA CORRECTION	10079195	1
SHORT INTERPREGNANCY INTERVAL	10079272	1
CSF EOSINOPHIL COUNT	10079290	1
URINE ORGANIC ACID TEST	10079357	1
ACUTE MACULAR OUTER RETINOPATHY	10079367	1
EPSTEIN BARR VIRUS POSITIVE MUCOCUTANEOUS ULCER	10079386	1
ELECTROGASTROGRAM	10079538	1
TENDON DISLOCATION	10079547	1
MESENTERIC ARTERY ANEURYSM	10079556	1
ORAL DYSPLASIA	10079559	1
THYMIC CYST	10079570	1
PERICARDIAL MASS	10079578	1
CARDIAC VALVE THICKENING	10079587	1
WIG WEARER	10079603	1
SUBCUTANEOUS DRUG ABSORPTION IMPAIRED	10079629	1
LUPUS MYOSITIS	10079642	1
FRACTIONAL EXHALED NITRIC OXIDE	10079700	1
DIFFUSION-WEIGHTED BRAIN MRI	10079718	1
ATRIAL APPENDAGE CLOSURE	10079735	1
EXPOSURE VIA TRANSPLANT	10079736	1
ATYPICAL HAEMOLYTIC URAEMIC SYNDROME	10079840	1
FOREIGN BODY IN GASTROINTESTINAL TRACT	10079846	1
TRICHODYNIA	10079852	1
SUBCHONDRAL INSUFFICIENCY FRACTURE	10079864	1
PNEUMONIA ACINETOBACTER	10079866	1
PNEUMONIA SERRATIA	10079868	1
SUPERIOR SEMICIRCULAR CANAL DEHISCENCE	10079888	1
CRYPTITIS	10079893	1
BENZODIAZEPINE DRUG LEVEL INCREASED	10079903	1
INTRACARDIAC PRESSURE INCREASED	10079904	1
MINIMAL RESIDUAL DISEASE	10079987	1

URINARY BLADDER SUSPENSION	10079992	1
PRODUCT APPEARANCE CONFUSION	10080000	1
OEDEMA BLISTER	10080039	1
EUGLYCAEMIC DIABETIC KETOACIDOSIS	10080061	1
PROSTHETIC CARDIAC VALVE STENOSIS	10080073	1
PATERNAL EXPOSURE BEFORE PREGNANCY	10080093	1
SITOPHOBIA	10080170	1
ARTERIAL TORTUOSITY SYNDROME	10080250	1
ARTERIAL DOLICHOECTASIA	10080307	1
DEVICE DISPENSING ERROR	10080357	1
DUPLICATE THERAPY ERROR	10080422	1
ANIMAL ATTACK	10080456	1
VIBRIO VULNIFICUS INFECTION	10080490	1
REMOVAL OF EXTERNAL FIXATION	10080546	1
HYPERTHERMIC CHEMOTHERAPY	10080557	1
POST PROCEDURAL FEVER	10080559	1
GASTROINTESTINAL VASCULAR MALFORMATION HAEMORRHA	10080561	1
HISTAMINE RELEASE TEST	10080590	1
GUT FERMENTATION SYNDROME	10080594	1
RENAL ARTERY RESTENOSIS	10080626	1
IMPLANTABLE CARDIAC MONITOR REMOVAL	10080633	1
AUTOIMMUNE LUNG DISEASE	10080701	1
STOMA CREATION	10080707	1
UTERINE CERVIX HYPERPLASIA	10080728	1
JC POLYOMAVIRUS TEST	10080768	1
FINKELSTEIN TEST	10080810	1
AGGLUTINATION TEST	10080821	1
SNAPPING HIP SYNDROME	10080832	1
TARTRATE-RESISTANT ACID PHOSPHATASE INCREASED	10080834	1
MIXED ANXIETY AND DEPRESSIVE DISORDER	10080836	1
ENTERIC NEUROPATHY	10080846	1
ISOCITRATE DEHYDROGENASE GENE MUTATION	10080848	1
HTLV-1 TEST	10080855	1
ADENOSINE DEAMINASE	10080858	1
VISUOSPATIAL DEFICIT	10080880	1
HYPERGLYCINAEMIA	10080883	1
BRAIN STENT INSERTION	10080887	1
PROCEDURAL SHOCK	10080894	1
TISSUE INFILTRATION	10080901	1
ASSISTED REPRODUCTIVE TECHNOLOGY	10080951	1
PARANEOPLASTIC MYELOPATHY	10080965	1
SINGLE COMPONENT OF A TWO-COMPONENT PRODUCT ADM	10080977	1
CUTIS VERTICIS GYRATA	10080996	1

OBESITY CARDIOMYOPATHY	10081007	1
TRANSILLUMINATION	10081057	1
PERIPHERAL ARTERY HAEMATOMA	10081077	1
AUTOIMMUNE EYE DISORDER	10081123	1
DIGESTIVE ENZYME DECREASED	10081158	1
DIGESTIVE ENZYME NORMAL	10081160	1
EYE DROP INSTILLATION	10081161	1
EPILEPSY WITH MYOCLONIC-ATONIC SEIZURES	10081179	1
PLATELET RICH PLASMA THERAPY	10081187	1
JOINT POSITION SENSE DECREASED	10081223	1
PIK3CA RELATED OVERGROWTH SPECTRUM	10081236	1
PITUITARY GLAND OPERATION	10081273	1
ALCOHOL TEST POSITIVE	10081297	1
MAISONNEUVE FRACTURE	10081343	1
EAR AND HEARING DISORDER PROPHYLAXIS	10081384	1
SPINAL SEGMENTAL DYSFUNCTION	10081395	1
SPLENIC FLEXURE MOBILISATION	10081418	1
TRACHEAL ASPIRATION PROCEDURE	10081462	1
SUSPECTED PRODUCT QUALITY ISSUE	10081480	1
ACUTE MYELOID LEUKAEMIA REFRACTORY	10081513	1
NATURAL KILLER CELL ACTIVITY TEST	10081529	1
DIABETIC WOUND	10081558	1
INTERCEPTED PRODUCT ADMINISTRATION ERROR	10081573	1
PRODUCT MONITORING ERROR	10081575	1
OTOLITHIASIS	10081585	1
ELECTROCARDIOGRAM S1-S2-S3 PATTERN	10081593	1
SEIZURE PROPHYLAXIS	10081601	1
SPINAL SYNOVIAL CYST	10081613	1
DUROTOMY PROCEDURE	10081615	1
SERPIGINOUS CHOROIDITIS	10081652	1
GENITAL DISCOLOURATION	10081697	1
INTERCEPTED PRODUCT PRESCRIBING ERROR	10081743	1
CONTRAINDICATED PRODUCT PRESCRIBED	10081744	1
MURPHY'S SIGN TEST	10081748	1
CATECHOLAMINE CRISIS	10081751	1
STEROID DIABETES	10081755	1
ABSENCE OF IMMEDIATE TREATMENT RESPONSE	10081766	1
CARDIAC DEVICE REPROGRAMMING	10081886	1
CEREBRAL ARTERY STENT INSERTION	10081893	1
CHECKPOINT KINASE 2 GENE MUTATION	10081927	1
LARYNGEAL STROBOSCOPY	10081946	1
PERNIO-LIKE ERYTHEMA	10081993	1
PLACENTAL CALCIFICATION	10082008	1

RETINOSCOPY	10082015	1
INCISION SITE IMPAIRED HEALING	10082028	1
PORTAL VEIN EMBOLISM	10082030	1
NEONATAL SEIZURE	10082067	1
UMBILICAL CORD BLOOD PH	10082071	1
PLEURAL MASS	10082101	1
GASTRIC ELECTRICAL STIMULATION	10082102	1
THERAPEUTIC PRODUCT EFFECT INCREASED	10082203	1
ENZYME ACTIVITY ASSAY	10082222	1
POSTPARTUM ANXIETY	10082233	1
ANTI-RNA POLYMERASE III ANTIBODY POSITIVE	10082283	1
EXPOSURE TO TOBACCO	10082288	1
URTICARIAL DERMATITIS	10082290	1
DRUG TITRATION	10082292	1
DORSAL RAMUS SYNDROME	10082295	1
ENDOCERVICAL MUCOSAL THICKENING	10082304	1
LEFT ATRIAL VOLUME INCREASED	10082369	1
AMPLIFIED MUSCULOSKELETAL PAIN SYNDROME	10082375	1
ABERRANT AORTIC ARCH	10082380	1
PROGRESSIVE MACULAR HYPOMELANOSIS	10082386	1
NON-SCARRING ALOPECIA	10082395	1
ENTERORRHAPHY	10082396	1
INCREASED LIVER STIFFNESS	10082444	1
PROTHROMBIN FRAGMENT 1.2	10082446	1
VERTEBROBASILAR STROKE	10082484	1
HYPERRESPONSIVE TO STIMULI	10082489	1
PANCREATIC CYST DRAINAGE	10082531	1
CAPNOGRAM	10082532	1
PULMONARY IMAGING PROCEDURE NORMAL	10082581	1
STRONGYLOIDES TEST POSITIVE	10082598	1
ABDOMINAL WALL OEDEMA	10082609	1
POSITRON EMISSION TOMOGRAM BREAST ABNORMAL	10082622	1
DECREASED GAIT VELOCITY	10082636	1
BIOPSY NASOPHARYNX ABNORMAL	10082658	1
ANAL BLISTER	10082677	1
ADMINISTRATION SITE ACNE	10082766	1
VAGINAL DILATION PROCEDURE	10082791	1
VERTEBRAL END PLATE INFLAMMATION	10082831	1
COMPUTED TOMOGRAPHIC GASTROGRAPHY	10082840	1
NASAL POTENTIAL DIFFERENCE TEST	10082865	1
HABIT COUGH	10082913	1
VESTIBULAR PAROXYSMIA	10082929	1
COMPUTERISED TOMOGRAM PANCREAS	10082934	1

STEROID DEPENDENCE	10082986	1
INJECTION SITE PANNICULITIS	10083040	1
MACROPHAGE INFLAMMATORY PROTEIN-1 ALPHA INCREASED	10083049	1
IMMUNE-MEDIATED ENCEPHALITIS	10083074	1
LIPOPROTEIN-ASSOCIATED PHOSPHOLIPASE A2	10083082	1
SUBDURAL ABSCESS	10083083	1
LUNG DIFFUSION TEST DECREASED	10083094	1
LUNG DIFFUSION DISORDER	10083095	1
ILIAC VEIN STENOSIS	10083102	1
PERIPHERAL VEIN STENOSIS	10083104	1
AURICULAR CHONDRITIS	10083111	1
MAGNETIC RESONANCE IMAGING PANCREAS	10083121	1
DEVICE USE CONFUSION	10083151	1
PHARYNGEAL CONTUSION	10083176	1
PLACENTAL CYST	10083196	1
ELECTROCARDIOGRAM P WAVE	10083198	1
NASOPHARYNGEAL TUMOUR	10083253	1
BRONCHIOLITIS OBLITERANS SYNDROME	10083303	1
GENERALISED ONSET NON-MOTOR SEIZURE	10083376	1
INTESTINAL MUCOSAL TEAR	10083542	1
FOLLICULAR CYSTITIS	10083549	1
ORBITAL HAEMATOMA	10083565	1
AUTOIMMUNE CHOLANGITIS	10083636	1
HYPOTENSIVE CRISIS	10083659	1
CEREBRAL MICROINFARCTION	10083668	1
SPLENIC ARTERY EMBOLISATION	10083795	1
INTERNAL DEVICE EXPOSED	10083797	1
HEPATIC PERFUSION DISORDER	10083840	1
ABLEPHARON MACROSTOMIA SYNDROME	10083860	1
PARADOXICAL PSORIASIS	10083929	1
OCCUPATIONAL EXPOSURE TO SUNLIGHT	10083930	1
DIALYSIS HYPOTENSION	10084012	1
MENINGITIS ESCHERICHIA	10084029	1
EPIGLOTTIC CANCER	10084056	1
VASCULAR GRAFT INFECTION	10084057	1
EMBOLIC CEREBELLAR INFARCTION	10084072	1
ATRIOVENTRICULAR NODE DYSFUNCTION	10084085	1
AST TO PLATELET RATIO INDEX INCREASED	10084175	1
FACIOBRACHIAL DYSTONIC SEIZURE	10084187	1
PLATELET STORAGE POOL DEFICIENCY	10084190	1
POOR MILK EJECTION REFLEX	10084213	1
VERTEBRAL ARTERY ARTERIOSCLEROSIS	10084347	1
GARDNERELLA TEST	10084361	1

SULPHUR DIOXIDE TEST	10084384	1
OCCUPATIONAL EXPOSURE TO COMMUNICABLE DISEASE	10084393	1
DISSEMINATED VARICELLA	10084395	1
INTENTIONAL PRODUCT MISUSE TO CHILD	10084403	1
MERS-COV TEST	10084443	1
SARS-COV-2 CARRIER	10084461	1
TONGUE INDURATION	10084548	1
GALLBLADDER MASS	10084568	1
STOOL DNA TEST NEGATIVE	10084583	1
SRSF2 GENE MUTATION	10084608	1
ASXL1 GENE MUTATION	10084611	1
BRONCHOSCOPIC LUNG VOLUME REDUCTION	10084618	1
CHRONIC RHINOSINUSITIS WITHOUT NASAL POLYPS	10084742	1
ARTERIOVENOUS FISTULA SITE PSEUDOANEURYSM	10084750	1
REACTIVE CAPILLARY ENDOTHELIAL PROLIFERATION	10084761	1
VOCAL CORD ERYTHEMA	10084781	1
VOCAL CORD BOWING	10084782	1
OTOENDOSCOPY	10084853	1
DEPRESSION RATING SCALE SCORE	10084952	1
URINARY OCCULT BLOOD	10084960	1
VACCINE ASSOCIATED ENHANCED RESPIRATORY DISEASE	10085001	1
ENDOBRONCHIAL ULTRASOUND TRANSBRONCHIAL NEEDLE AS	10085040	1
PARANASAL SINUS MASS	10085055	1
HCOV-OC43 INFECTION	10085077	1
RECURRENT SUBAREOLAR BREAST ABSCESS	10085084	1
TREATMENT DELAYED	10085087	1
SEROMA DRAINAGE	10085114	1
FOLLICULAR LYMPHOMA STAGE I	10085123	1
NEUTROPHIL/LYMPHOCYTE RATIO	10085124	1
OPTOMETRIC THERAPY	10085142	1
DENTAL IMPLANT REMOVAL	10085172	1
CONFIRMED E-CIGARETTE OR VAPING PRODUCT USE ASSOCIA	10085189	1
FOLLICULAR ECZEMA	10085198	1
THYMOMA BENIGN	10085199	1
LUMBAR ARTERY EMBOLISATION	10085311	1
BRONCHIAL NEOPLASM	10052245	1
SALIVARY GLAND CYST	10052248	1
CATHETER SITE PAIN	10052268	1
DYSTROPHIC CALCIFICATION	10052273	1
HIGH-PITCHED CRYING	10052286	1
KERATOLYSIS EXFOLIATIVA ACQUIRED	10052291	1
VAGINAL CELLULITIS	10052301	1
NASAL FLARING	10052319	1

PRIMARY ADRENAL INSUFFICIENCY	10052381	1
BILIARY CYST	10052384	1
GASTRIC HYPOMOTILITY	10052405	1
MYOFASCITIS	10052443	1
OCULAR NEOPLASM	10052448	1
FACTOR X DEFICIENCY	10052474	1
GASTRIC VOLVULUS	10052541	1
OTITIS EXTERNA FUNGAL	10052557	1
URTICARIA PRESSURE	10052572	1
SKIN COSMETIC PROCEDURE	10052608	1
ALLERGIC BRONCHITIS	10052613	1
POSTOPERATIVE CARE	10052617	1
CYSTIC FIBROSIS CARRIER	10052656	1
BIOPSY BLOOD VESSEL	10052663	1
VASCULAR SHUNT	10052664	1
VALVULOPLASTY CARDIAC	10052681	1
UROLOGICAL EXAMINATION	10052684	1
TRICUSPID VALVE REPAIR	10052690	1
TRANSURETHRAL BLADDER RESECTION	10052693	1
TRACHEOSCOPY	10052694	1
ADENOCARCINOMA PANCREAS	10052747	1
FIBROUS CORTICAL DEFECT	10052753	1
BODY DYSMORPHIC DISORDER	10052793	1
DRUG TOLERANCE INCREASED	10052806	1
PERIRECTAL ABSCESS	10052814	1
EOSINOPHILIC PUSTULAR FOLLICULITIS	10052834	1
PRENATAL CARE	10052859	1
X-RAY THERAPY TO BLOOD	10052869	1
MURPHY'S SIGN POSITIVE	10052900	1
CARDIAC SEPTAL DEFECT REPAIR	10052910	1
SPINAL SUPPORT	10052920	1
CLAMPING OF BLOOD VESSEL	10052928	1
ARTERIAL THERAPEUTIC PROCEDURE	10052949	1
DRAIN OF CEREBRAL SUBDURAL SPACE	10052957	1
IMPLANTABLE DEFIBRILLATOR REMOVAL	10052958	1
INTERMITTENT POSITIVE PRESSURE BREATHING	10052959	1
VENOUS REPAIR	10052964	1
MUSCLE ELECTROSTIMULATION THERAPY	10052969	1
DRUG WITHDRAWAL MAINTENANCE THERAPY	10052970	1
LIP OPERATION	10052977	1
JOINT IRRIGATION	10052987	1
ILEOCOLOSTOMY	10052997	1
HYSTEROSALPINGECTOMY	10052998	1

BIOPSY FOETAL	10053139	1
INDUCED ABORTION FAILED	10053191	1
EYELID BLEEDING	10053196	1
LEUKOERYTHROBLASTIC ANAEMIA	10053199	1
PARALYTIC DISABILITY	10053201	1
WOUND DECOMPOSITION	10053208	1
MIXED INCONTINENCE	10053236	1
BIOPSY SMALL INTESTINE ABNORMAL	10053283	1
OPHTHALMIC FLUID DRAINAGE	10053299	1
ABDOMINAL EXPLORATION	10053309	1
PSYCHIATRIC EVALUATION ABNORMAL	10053338	1
ROUTINE HEALTH MAINTENANCE	10053339	1
ENDOTHELIN ABNORMAL	10053403	1
BRAIN NATRIURETIC PEPTIDE ABNORMAL	10053408	1
APPLICATION SITE SWELLING	10053424	1
CARDIAC VENTRICULOGRAM ABNORMAL	10053447	1
ANTACID THERAPY	10053458	1
ANTIINFLAMMATORY THERAPY	10053467	1
PHYSICAL BREAST EXAMINATION NORMAL	10053479	1
CARDIAC VENTRICULOGRAM LEFT	10053497	1
CARDIAC VENTRICULOGRAM LEFT ABNORMAL	10053499	1
SCAN BONE MARROW NORMAL	10053503	1
INFUSION SITE SWELLING	10053505	1
CLEFT UVULA	10053507	1
URINE PROTEIN/CREATININE RATIO DECREASED	10053536	1
URINE PROTEIN/CREATININE RATIO ABNORMAL	10053539	1
URINE ALBUMIN/CREATININE RATIO NORMAL	10053543	1
VITH NERVE DISORDER	10053646	1
PROSTHESIS USER	10053669	1
CARDIOVASCULAR AUTONOMIC FUNCTION TEST NORMAL	10053690	1
EXCESSIVE OCULAR CONVERGENCE	10053693	1
PULMONARY FUNCTION CHALLENGE TEST	10053709	1
PULMONARY FUNCTION CHALLENGE TEST ABNORMAL	10053711	1
WOUND NECROSIS	10053716	1
FIBROUS HISTIOCYTOMA	10053717	1
BRAIN TUMOUR OPERATION	10053749	1
ABDOMINOPLASTY	10053774	1
BLOOD TESTOSTERONE FREE NORMAL	10053788	1
BLOOD ERYTHROPOIETIN INCREASED	10053795	1
BLOOD ANDROSTENEDIONE INCREASED	10053808	1
BLOOD 1,25-DIHYDROXYCHOLECALCIFEROL INCREASED	10053810	1
SNEDDON'S SYNDROME	10053841	1
MELAS SYNDROME	10053872	1

SEPSIS SYNDROME	10053879	1
TRISOMY 18	10053884	1
CAROTID PULSE INCREASED	10053898	1
POPLITEAL PULSE DECREASED	10053905	1
POPLITEAL PULSE INCREASED	10053906	1
RED CELL DISTRIBUTION WIDTH ABNORMAL	10053923	1
CAROTID PULSE	10053926	1
THROMBIN-ANTITHROMBIN III COMPLEX	10053970	1
THROMBIN-ANTITHROMBIN III COMPLEX NORMAL	10053971	1
IMPLANT SITE HAEMORRHAGE	10053995	1
THYROGLOBULIN INCREASED	10054010	1
THYROGLOBULIN PRESENT	10054024	1
PROGESTERONE RECEPTOR ASSAY	10054056	1
PROGESTERONE RECEPTOR ASSAY NEGATIVE	10054058	1
VESSEL PUNCTURE SITE HAEMORRHAGE	10054092	1
MICROCOCCUS INFECTION	10054120	1
POST PROCEDURAL DISCOMFORT	10054126	1
HEPATITIS B IMMUNISATION	10054130	1
STENOTROPHOMONAS INFECTION	10054138	1
SALIVA ALCOHOL TEST POSITIVE	10054176	1
SMALL INTESTINE CARCINOMA	10054184	1
GAIT DEVIATION	10054197	1
IIIRD NERVE PARESIS	10054202	1
AEROMONAS INFECTION	10054205	1
ALCOHOL REHABILITATION	10054206	1
ENTEROCOCCAL SEPSIS	10054221	1
SCLERAL THINNING	10054763	1
DECREASED BRONCHIAL SECRETION	10054764	1
ANTERIOR CHAMBER INFLAMMATION	10054765	1
RETROPLACENTAL HAEMATOMA	10054798	1
INJECTION SITE CALCIFICATION	10054812	1
BLOOD PHOSPHORUS ABNORMAL	10054823	1
RECTAL LESION	10054828	1
EXTERNAL AUDITORY CANAL ATRESIA	10054875	1
MENISCUS OPERATION	10054948	1
PULMONARY HAEMATOMA	10054991	1
CERVIX CERCLAGE PROCEDURE	10054992	1
BROMHIDROSIS	10055000	1
EAR NEOPLASM	10055016	1
GINGIVAL CYST	10055018	1
ANAL INJURY	10055023	1
UMBILICAL ERYTHEMA	10055029	1
ERYTHEMA DYSCHROMICUM PERSTANS	10055035	1

RECTAL CANCER METASTATIC	10055097	1
BONE CANCER METASTATIC	10055101	1
PITUITARY CANCER METASTATIC	10055106	1
TONGUE CANCER METASTATIC	10055109	1
ARTERIOVENOUS FISTULA SITE COMPLICATION	10055122	1
ARTERIOVENOUS FISTULA SITE HAEMORRHAGE	10055123	1
AUTOIMMUNE NEUTROPENIA	10055128	1
CATHETER SITE HAEMATOMA	10055662	1
CORNEAL NEOVASCULARISATION	10055665	1
NECROTISING ULCERATIVE GINGIVOSTOMATITIS	10055670	1
PARKINSONIAN GAIT	10056242	1
EOSINOPHILIC CYSTITIS	10056246	1
ANTICONVULSANT DRUG LEVEL THERAPEUTIC	10056282	1
CD8 LYMPHOCYTES DECREASED	10056283	1
STERNAL INJURY	10056287	1
GLUTATHIONE DECREASED	10056290	1
NEOPLASM OF THYMUS	10056296	1
ANTICONVULSANT DRUG LEVEL BELOW THERAPEUTIC	10056327	1
ALCOHOLIC SEIZURE	10056347	1
REFUSAL OF TREATMENT BY RELATIVE	10056406	1
BLADDER NECK OPERATION	10056416	1
ARTERIAL BYPASS OPERATION	10056418	1
SWAN GANZ CATHETER PLACEMENT	10056442	1
PERICARDIAL DRAINAGE TEST NORMAL	10056463	1
CORONARY ARTERY RESTENOSIS	10056489	1
BENIGN BILIARY NEOPLASM	10056514	1
PSOAS ABSCESS	10056517	1
CATHETER SITE INFECTION	10056520	1
PERITONEAL CANDIDIASIS	10056562	1
LIPECTOMY	10056588	1
APOLIPOPROTEIN	10056630	1
MIDDLE LOBE SYNDROME	10056652	1
RETROPERITONEAL NEOPLASM	10056664	1
PHOBIA OF DRIVING	10056676	1
MENISCUS REMOVAL	10056722	1
GASTROINTESTINAL ULCER HAEMORRHAGE	10056743	1
EGOBRONCHOPHONY	10056744	1
BODY HEIGHT ABNORMAL	10056822	1
RECTAL EXAMINATION NORMAL	10056823	1
OTOSCOPY NORMAL	10056825	1
OTOSCOPY ABNORMAL	10056826	1
UROLOGICAL EXAMINATION ABNORMAL	10056842	1
MEDICAL OBSERVATION NORMAL	10056843	1

EMERGENCY CARE EXAMINATION NORMAL	10056845	1
APGAR SCORE NORMAL	10056849	1
FACIAL WASTING	10056866	1
ACROCHORDON EXCISION	10056875	1
NEAR DROWNING	10056905	1
NECROBIOSIS LIPOIDICA DIABETICORUM	10056969	1
OESOPHAGEAL MASS	10057003	1
MECONIUM STAIN	10057028	1
POIKILODERMA	10057041	1
MULTIFOCAL MICRONODULAR PNEUMOCYTE HYPERPLASIA	10057109	1
SYNOVIAL FLUID RED BLOOD CELLS POSITIVE	10057111	1
SYNOVIAL FLUID CRYSTAL PRESENT	10057112	1
URINE URIC ACID INCREASED	10057120	1
URINE TRANSITIONAL CELLS PRESENT	10057121	1
URINE OXALATE	10057138	1
URINE URIC ACID NORMAL	10057140	1
FRACTURE DEBRIDEMENT	10057147	1
POST LAMINECTOMY SYNDROME	10057239	1
CELL-MEDIATED CYTOTOXICITY	10057250	1
LOWER RESPIRATORY TRACT INFLAMMATION	10057260	1
LUNG INDURATION	10057261	1
NEUROENDOCRINE CARCINOMA	10057270	1
WEST NILE VIRAL INFECTION	10057293	1
MICROGRAPHIA	10057333	1
ONYCHOPHAGIA	10057342	1
BLOOD BRAIN BARRIER DEFECT	10057361	1
IRIS INJURY	10057413	1
CILIARY BODY DISORDER	10057415	1
PERIORBITAL DISORDER	10057425	1
VITREOUS ADHESIONS	10057435	1
VITREOUS INJURY	10057436	1
ADENOIDAL DISORDER	10057449	1
ANGIOGRAM PERIPHERAL	10057452	1
PERICARDIAL OPERATION	10057463	1
TRICUSPID VALVE CALCIFICATION	10057466	1
CORNEAL LIGHT REFLEX TEST ABNORMAL	10057512	1
HAEMODYNAMIC TEST NORMAL	10057522	1
ECG P WAVE INVERTED	10057526	1
BONE MARROW MYELOGRAM NORMAL	10057527	1
BONE MARROW MYELOGRAM ABNORMAL	10057528	1
ILIAC ARTERY DISSECTION	10057554	1
SPINAL MYELOGRAM ABNORMAL	10057565	1
CHRONIC HEPATIC FAILURE	10057573	1

POST PROCEDURAL DIARRHOEA	10057585	1
POST PROCEDURAL BILE LEAK	10057586	1
POST PROCEDURAL URINE LEAK	10057587	1
BLOOD KETONE BODY DECREASED	10057595	1
BLOOD KETONE BODY PRESENT	10057598	1
BLOOD KETONE BODY ABSENT	10057600	1
CHOLEDOCHOLITHOTOMY	10057601	1
URINARY TRACT OPERATION	10057611	1
AORTIC BYPASS	10057617	1
COCKROACH ALLERGY	10057643	1
CYTOREDUCTIVE SURGERY	10057647	1
FEMALE SEXUAL DYSFUNCTION	10057671	1
BIOPSY CHEST WALL ABNORMAL	10057684	1
ACQUIRED CLAW TOE	10057689	1
POST PROCEDURAL DISCHARGE	10057751	1
AORTOGRAM	10057775	1
BILIARY SEPSIS	10057847	1
ANTICONVULSANT DRUG LEVEL INCREASED	10057856	1
BONE DENSITY INCREASED	10057864	1
TENDON CALCIFICATION	10057888	1
ENDOMETRIOSIS ABLATION	10057902	1
ELECTROCARDIOGRAM U WAVE PRESENT	10057913	1
TRANSPLANT EVALUATION	10057925	1
IRON BINDING CAPACITY UNSATURATED DECREASED	10057966	1
TOXIC SKIN ERUPTION	10057970	1
HEPATITIS E ANTIBODY	10057992	1
INFECTED NEOPLASM	10058014	1
CANCER PAIN	10058019	1
PROSTATIC SPECIFIC ANTIGEN NORMAL	10058022	1
JOINT ADHESION	10058031	1
CYSTATIN C INCREASED	10058037	1
ABDOMINAL SEPSIS	10058040	1
GRAFT COMPLICATION	10058060	1
NEUTRALISING ANTIBODIES	10058063	1
PRECOCIOUS PUBERTY	10058084	1
HANGNAIL	10058109	1
EOSINOPHIL PERCENTAGE ABNORMAL	10058133	1
SUBENDOCARDIAL ISCHAEMIA	10058145	1
REPERFUSION ARRHYTHMIA	10058156	1
LUPUS ENDOCARDITIS	10058225	1
ECG SIGNS OF MYOCARDIAL ISCHAEMIA	10058317	1
PARAKERATOSIS	10058318	1
MATERNAL THERAPY TO ENHANCE FOETAL LUNG MATURITY	10058340	1

HYPOGONADISM	10058359	1
EOSINOPHILS URINE PRESENT	10058363	1
BIOPSY PANCREAS ABNORMAL	10058374	1
MEDIASTINAL BIOPSY ABNORMAL	10058378	1
EOSINOPHILS URINE	10058391	1
BIOPSY FALLOPIAN TUBE NORMAL	10058397	1
BIOPSY BLOOD VESSEL ABNORMAL	10058410	1
NAIL PIGMENTATION	10058455	1
HYPEROXIA	10058490	1
NASAL ASPIRATION	10058525	1
BILE OUTPUT INCREASED	10058528	1
PERIPHERAL NERVE PALSY	10058530	1
ANTI-PLATELET ANTIBODY NEGATIVE	10058541	1
POOR SUCKING REFLEX	10058605	1
URINE CALCIUM/CREATININE RATIO	10058624	1
TOTAL CHOLESTEROL/HDL RATIO INCREASED	10058630	1
TOTAL CHOLESTEROL/HDL RATIO DECREASED	10058631	1
EMBOLIA CUTIS MEDICAMENTOSA	10058729	1
MIGRAINE PROPHYLAXIS	10058734	1
HIV ANTIBODY POSITIVE	10058736	1
HIV ANTIGEN POSITIVE	10058737	1
EYELASH DISCOLOURATION	10058809	1
HEPATITIS B REACTIVATION	10058827	1
ELECTIVE SURGERY	10058829	1
KLEBSIELLA BACTERAEMIA	10058856	1
ENTEROBACTER BACTERAEMIA	10058857	1
ULTRASOUND TESTES NORMAL	10058864	1
CONGENITAL THROMBOCYTE DISORDER	10058896	1
SALMONELLA BACTERAEMIA	10058924	1
HAEMOBILIA	10058947	1
CYSTIC LYMPHANGIOMA	10058949	1
VICTIM OF ABUSE	10058984	1
HEPATIC VEIN OCCLUSION	10058991	1
ACUTE HAEMORRHAGIC LEUKOENCEPHALITIS	10058994	1
GASTROINTESTINAL ISCHAEMIA	10059028	1
ACUTE MYELOID LEUKAEMIA RECURRENT	10059034	1
POSTPRANDIAL HYPOGLYCAEMIA	10059035	1
SCEDOSPORIUM INFECTION	10059045	1
BILOMA	10059118	1
CEREBROSCLEROSIS	10059150	1
CARDIAC MASSAGE	10059163	1
ACUTE HEPATITIS B	10059193	1
PERINEPHRIC COLLECTION	10059209	1

TISSUE POLYPEPTIDE ANTIGEN	10059212	1
BIOPSY SPLEEN ABNORMAL	10059227	1
HAEMOGLOBIN URINE	10059228	1
INCISION SITE HAEMATOMA	10059241	1
DIALYSIS DISEQUILIBRIUM SYNDROME	10059256	1
DELIRIUM FEBRILE	10059267	1
PANCREATIC CARCINOMA STAGE IV	10059326	1
DESMOID TUMOUR	10059352	1
PARASITIC TEST POSITIVE	10059422	1
GALLBLADDER NECROSIS	10059446	1
HAEMODILUTION	10059484	1
MUCOSAL EXCORIATION	10059490	1
HAEMORRHAGIC URTICARIA	10059499	1
APOPTOSIS	10059512	1
PERITONEAL FLUID ANALYSIS ABNORMAL	10059524	1
SALIVA ANALYSIS NORMAL	10059533	1
BETA GLOBULIN DECREASED	10059546	1
OTOPLASTY	10059588	1
NECROBIOSIS	10059605	1
STROKE IN EVOLUTION	10059613	1
ABDOMINAL WALL DISORDER	10059625	1
MUMPS ANTIBODY TEST	10059638	1
LARYNGEAL OBSTRUCTION	10059639	1
MUMPS ANTIBODY TEST NEGATIVE	10059645	1
SEROCONVERSION TEST NEGATIVE	10059647	1
SEX HORMONE BINDING GLOBULIN INCREASED	10059659	1
REPRODUCTIVE HORMONE	10059686	1
HISTONE ANTIBODY NEGATIVE	10059708	1
ANTI-GAD ANTIBODY NEGATIVE	10059730	1
CSF MYELIN BASIC PROTEIN NORMAL	10059752	1
DEXAMETHASONE SUPPRESSION TEST	10059774	1
ULNAR NERVE PALSY	10059790	1
GLUCOSE-6-PHOSPHATE DEHYDROGENASE ABNORMAL	10059902	1
RED BLOOD CELL SPHEROCYTES PRESENT	10059916	1
MYOGLOBIN BLOOD DECREASED	10059940	1
CARNITINE	10059952	1
ALBUMIN CSF ABNORMAL	10059976	1
ALBUMIN CSF DECREASED	10059979	1
BLOOD ALCOHOL ABNORMAL	10059995	1
BLOOD GALACTOSE	10060022	1
INTERFERON GAMMA LEVEL	10060023	1
GASTRIC PH	10060024	1
URINE LACTIC ACID	10060064	1

URINE MERCURY ABNORMAL	10060095	1
POTASSIUM CHLORIDE SENSITIVITY TEST	10060194	1
PLATELET FACTOR 4 INCREASED	10060219	1
PLATELET FACTOR 4 DECREASED	10060220	1
POTASSIUM CHLORIDE SENSITIVITY TEST ABNORMAL	10060225	1
RHEUMATOID FACTOR QUANTITATIVE INCREASED	10060234	1
RIGHT VENTRICULAR SYSTOLIC PRESSURE DECREASED	10060237	1
REVERSE TRI-IODOTHYRONINE	10060292	1
RESPIRATORY TRACT INFECTION BACTERIAL	10060693	1
PRESBYOESOPHAGUS	10060696	1
GASTRIC BANDING	10060785	1
LARYNGOMALACIA	10060786	1
ABDOMINAL HERNIA REPAIR	10060802	1
MYXOEDEMA COMA	10060819	1
NECROLYTIC MIGRATORY ERYTHEMA	10060821	1
HUMAN HERPES VIRUS 6 SEROLOGY NEGATIVE	10060828	1
PROSTATE CANCER	10060862	1
TRANSPLANT FAILURE	10060872	1
AORTIC RUPTURE	10060874	1
BENIGN FAMILIAL HAEMATURIA	10060876	1
HEREDITARY HAEMOLYTIC ANAEMIA	10060893	1
CALCIUM IONISED ABNORMAL	10060896	1
CALCIUM IONISED INCREASED	10060899	1
VOLUME BLOOD	10060917	1
ABDOMINAL HERNIA OBSTRUCTIVE	10060923	1
ABDOMINAL INJURY	10060924	1
ADENOVIRUS INFECTION	10060931	1
ANALGESIC DRUG LEVEL DECREASED	10060941	1
ANALGESIC DRUG LEVEL INCREASED	10060942	1
ARTHROPOD-BORNE DISEASE	10060970	1
APICAL GRANULOMA	10060983	1
SPUTUM NORMAL	10060989	1
SPUTUM TEST	10060990	1
BENIGN SOFT TISSUE NEOPLASM	10061004	1
BLOOD CANNABINOIDS	10061012	1
BRACHYTHERAPY	10061018	1
BURSITIS INFECTIVE	10061022	1
CATECHOLAMINES URINE INCREASED	10061034	1
CENTRAL NERVOUS SYSTEM INFECTION	10061036	1
CESTODE INFECTION	10061039	1
CLOSTRIDIAL INFECTION	10061043	1
CONGENITAL CARDIOVASCULAR ANOMALY	10061054	1
SPUTUM DECREASED	10061081	1

ENDOCARDIAL DISEASE	10061120	1
FEMALE GENITAL TRACT FISTULA	10061149	1
GASTROENTERITIS BACTERIAL	10061166	1
GASTROINTESTINAL NEOPLASM	10061174	1
GENITAL INFECTION FUNGAL	10061180	1
HAEMATOPOIETIC NEOPLASM	10061187	1
HAMARTOMA	10061193	1
LEUKAEMIA IN REMISSION	10061220	1
MALIGNANT LYMPHOID NEOPLASM	10061240	1
LARYNGEAL DISORDER	10061263	1
LEARNING DISORDER	10061265	1
MALIGNANT NERVOUS SYSTEM NEOPLASM	10061268	1
MALIGNANT PERITONEAL NEOPLASM	10061269	1
MENINGEAL NEOPLASM	10061282	1
METASTASES TO NERVOUS SYSTEM	10061287	1
MUCOSAL EROSION	10061297	1
NAIL INFECTION	10061304	1
NECK DEFORMITY	10061307	1
NERVE ROOT INJURY	10061310	1
NEUROLOGICAL EYELID DISORDER	10061312	1
PARAGANGLION NEOPLASM	10061332	1
PARAPROTEINAEMIA	10061333	1
SPIROCHAETAL INFECTION	10061370	1
THERAPEUTIC REACTION TIME DECREASED	10061380	1
TRICUSPID VALVE DISEASE	10061389	1
TYPHUS	10061393	1
VICTIM OF CRIME	10061409	1
VISUAL PATHWAY DISORDER	10061411	1
CRYSTAL ARTHROPATHY	10061419	1
AMNESTIC DISORDER	10061423	1
URINE RETINOL BINDING PROTEIN INCREASED	10061432	1
CENTRAL NERVOUS SYSTEM STIMULATION	10061444	1
DENTOFACIAL FUNCTIONAL DISORDER	10061454	1
EXTERNAL EAR DISORDER	10061456	1
ERECTILE DYSFUNCTION	10061461	1
PULSE WAVEFORM ABNORMAL	10061475	1
RENAL NEOPLASM	10061482	1
PULSE WAVEFORM	10061488	1
POST PROCEDURAL FISTULA	10061489	1
RETINOSCHISIS	10061492	1
RICKETTSIOSIS	10061495	1
SCAN ADRENAL GLAND	10061503	1
SCAN PARATHYROID	10061507	1

MYOTONIA	10061533	1
OVARIAN NEOPLASM	10061535	1
PULMONARY VALVE DISEASE	10061541	1
SMALL INTESTINAL ULCER HAEMORRHAGE	10061550	1
URINE ELECTROLYTES DECREASED	10061579	1
ADRENAL NEOPLASM	10061588	1
ABORTION COMPLETE	10061614	1
ABORTION SPONTANEOUS COMPLETE	10061616	1
ANALGESIC INTERVENTION SUPPORTIVE THERAPY	10061631	1
ANGIODYSPLASIA	10061636	1
AORTIC SURGERY	10061651	1
AORTIC VALVE REPAIR	10061652	1
ARTERIAL GRAFT	10061655	1
ATYPICAL MYCOBACTERIAL INFECTION	10061663	1
ARTERIOGRAM NORMAL	10061682	1
BLADDER INJURY	10061698	1
BLADDER OPERATION	10061699	1
BLOOD CANNABINOIDS DECREASED	10061712	1
BLOOD CANNABINOIDS NORMAL	10061714	1
CEREBELLAR TUMOUR	10061750	1
CEREBROVASCULAR STENOSIS	10061751	1
CERVIX OPERATION	10061752	1
CHORIORETINAL DISORDER	10061763	1
COAGULATION FACTOR V LEVEL ABNORMAL	10061771	1
CORNEAL INFECTION	10061788	1
CSF PRESSURE ABNORMAL	10061798	1
CARDIAC ELECTROPHYSIOLOGIC STUDY NORMAL	10061807	1
DEFORMITY THORAX	10061810	1
DUODENAL OPERATION	10061826	1
ENTROPION	10061842	1
EYE EXERCISES	10061851	1
FACIAL OPERATION	10061854	1
FALLOPIAN TUBE NEOPLASM	10061855	1
FASCIAL OPERATION	10061856	1
TREMATODE INFECTION	10061859	1
ENDOSCOPY GASTROINTESTINAL NORMAL	10061861	1
OROPHARYNGITIS FUNGAL	10061891	1
OVARIAN GERM CELL CANCER	10061893	1
PANCREATIC ENZYMES ABNORMAL	10061899	1
PANCREATIC OPERATION	10061903	1
PARANASAL SINUS AND NASAL CAVITY MALIGNANT NEOPLASI	10061909	1
PARASOMNIA	10061910	1
PARATHYROID SCAN ABNORMAL	10061951	1

GASTRIC STENOSIS	10061970	1
GASTROINTESTINAL OBSTRUCTION	10061974	1
GENITAL INFECTION FEMALE	10061977	1
GLOMERULOSCLEROSIS	10061989	1
HAEMOPHILIA	10061992	1
HEART VALVE OPERATION	10061994	1
HEART VALVE STENOSIS	10061996	1
HEREDITARY ATAXIA	10062002	1
HUMAN T-CELL LYMPHOTROPIC VIRUS INFECTION	10062007	1
IMMUNE AGGLUTININS	10062011	1
IMMUNOSUPPRESSANT DRUG LEVEL	10062013	1
IMMUNOSUPPRESSANT DRUG LEVEL DECREASED	10062014	1
KERATITIS VIRAL	10062029	1
LACTOBACILLUS INFECTION	10062031	1
LUNG NEOPLASM	10062042	1
LYMPHOCYTE MORPHOLOGY	10062046	1
HISTOLOGY ABNORMAL	10062059	1
PERFORATED ULCER	10062065	1
PERINEAL OPERATION	10062068	1
RADIOTHERAPY TO BREAST	10062090	1
RHEUMATIC HEART DISEASE	10062110	1
SPLENIC INFECTION	10062112	1
TENDON OPERATION	10062121	1
TONGUE NEOPLASM	10062129	1
TONSILLAR NEOPLASM	10062131	1
TRACHEAL NEOPLASM	10062133	1
STOMACH SCAN NORMAL	10062146	1
VARICOSE VEIN OPERATION	10062168	1
VENOUS OPERATION	10062175	1
METASTASES TO SOFT TISSUE	10062197	1
MORAXELLA INFECTION	10062204	1
MUCOSAL BIOPSY	10062205	1
MYCOBACTERIAL INFECTION	10062207	1
URETERAL DISORDER	10062220	1
URINE CANNABINOIDS DECREASED	10062227	1
SKELETAL MUSCLE ENZYMES	10062246	1
SKIN IMPLANT	10062248	1
SMALL INTESTINE OPERATION	10062252	1
SPECIFIC GRAVITY BODY FLUID	10062256	1
SPECIFIC GRAVITY BODY FLUID NORMAL	10062260	1
BLAST CELL COUNT INCREASED	10062274	1
CONGENITAL CYST	10062328	1
CONGENITAL FOOT MALFORMATION	10062332	1

TRUNCUS COELIACUS THROMBOSIS	10062363	1
ERYTHEMA MIGRANS	10062488	1
EXOCRINE PANCREATIC FUNCTION TEST NORMAL	10062518	1
EROSIVE DUODENITIS	10062532	1
CONGENITAL MYOPATHY	10062547	1
VAGINAL EROSION	10062558	1
INJECTABLE CONTRACEPTION	10062577	1
ORAL ADMINISTRATION COMPLICATION	10062611	1
CORNEAL ENDOTHELIITIS	10062621	1
PANCREATIC ENZYMES DECREASED	10062646	1
CAPILLARY FRAGILITY TEST	10062658	1
NEMATODIASIS	10062701	1
HAEMOGLOBIN A2	10062734	1
VASOACTIVE INTESTINAL POLYPEPTIDE TEST	10062736	1
TRISOMY 15	10062757	1
ANKLE BRACHIAL INDEX NORMAL	10062761	1
HYPOPHYSITIS	10062767	1
NASAL CAVITY MASS	10062771	1
PINGUECULITIS	10062889	1
OLFACTORY TEST ABNORMAL	10062927	1
GASTRIC HYPERTONIA	10062931	1
INNER EAR OPERATION	10062990	1
PEDAL PULSE DECREASED	10062993	1
HAEMOGLOBIN ELECTROPHORESIS	10063002	1
HAEMATOTYMPANUM	10063013	1
ATHERECTOMY	10063025	1
FALSE POSITIVE TUBERCULOSIS TEST	10063050	1
PERITONEAL FLUID ANALYSIS	10063062	1
APPENDICITIS NONINFECTIVE	10063063	1
APPLICATION SITE DISCOMFORT	10063072	1
PREGNANCY WITH CONTRACEPTIVE DEVICE	10063130	1
ACOUSTIC NEURITIS	10063162	1
MACULAR SCAR	10063185	1
PARASITE DNA TEST	10063194	1
CHRONIC ALLOGRAFT NEPHROPATHY	10063209	1
AMPHETAMINES	10063227	1
AMPHETAMINES POSITIVE	10063228	1
BARBITURATES POSITIVE	10063230	1
OESTRADIOL ABNORMAL	10063266	1
MICROSCOPIC POLYANGIITIS	10063344	1
SYNOVIAL FLUID CELL COUNT	10063367	1
SYNOVIAL FLUID PROTEIN	10063368	1
DRUG THERAPY	10063370	1

POLYPOIDAL CHOROIDAL VASCULOPATHY	10063381	1
ROSAI-DORFMAN SYNDROME	10063397	1
DIHYDROTESTOSTERONE LEVEL	10063535	1
METASTATIC SQUAMOUS CELL CARCINOMA	10063569	1
EXPOSURE TO CHEMICAL POLLUTION	10063599	1
RADIOTHERAPY TO LYMPH NODES	10063616	1
HIPPOCAMPAL SCLEROSIS	10063629	1
GENITAL INJURY	10063630	1
PYRAMIDAL TRACT SYNDROME	10063636	1
KLEIHAUER-BETKE TEST	10063652	1
LYMPHOCYTIC HYPOPHYSITIS	10063685	1
ANTIPSYCHOTIC DRUG LEVEL INCREASED	10063686	1
URAEMIC GASTROPATHY	10063709	1
HEPATIC AMOEBIASIS	10063741	1
ELECTROCARDIOGRAM QT INTERVAL ABNORMAL	10063748	1
IMPLANT SITE INFLAMMATION	10063781	1
VULVAL HAEMORRHAGE	10063816	1
UNDERSENSING	10063835	1
ATRIOVENTRICULAR SEPTAL DEFECT	10063836	1
APPLICATION SITE INDURATION	10063839	1
VISCEROPTOSIS	10063840	1
IMPLANT SITE EXTRAVASATION	10063856	1
IMPLANT SITE INDURATION	10063860	1
IMPLANT SITE PUSTULES	10063866	1
IMPLANT SITE SWELLING	10063867	1
LOWER RESPIRATORY TRACT INFECTION BACTERIAL	10063890	1
RENAL ISCHAEMIA	10063897	1
DOLICHOCOLON	10063917	1
KABUKI MAKE-UP SYNDROME	10063935	1
ABDOMINAL BRUIT	10063943	1
PULMONARY AMYLOIDOSIS	10063945	1
ILEOCOLECTOMY	10063950	1
BRUDZINSKI'S SIGN	10064010	1
TUBERCULIN TEST FALSE POSITIVE	10064057	1
MULTIPLE SYSTEM ATROPHY	10064060	1
FACIOSCAPULOHUMERAL MUSCULAR DYSTROPHY	10064087	1
NAIL BED INFLAMMATION	10064088	1
SUGGESTIBILITY	10064093	1
AMNIOTIC BAND SYNDROME	10064100	1
INJECTION SITE JOINT INFLAMMATION	10064111	1
CONJUNCTIVOCHALASIS	10064132	1
RETINAL ANEURYSM	10064145	1
LAMINAPLASTY	10064161	1

BONE MARROW OEDEMA SYNDROME	10064269	1
MALIGNANT ATROPHIC PAPULOSIS	10064281	1
LABELLED DRUG-FOOD INTERACTION MEDICATION ERROR	10064374	1
CARDIAC VEIN DISSECTION	10064408	1
CD19 LYMPHOCYTES DECREASED	10064428	1
COLPOSCOPY ABNORMAL	10064457	1
MUCOSAL EXFOLIATION	10064486	1
INCARCERATED UMBILICAL HERNIA	10064492	1
EXCESSIVE SKIN	10064503	1
AUTOIMMUNE MYOCARDITIS	10064539	1
MARCUS GUNN SYNDROME	10064583	1
ORAL MUCOSA EROSION	10064594	1
NASAL CAVITY PACKING REMOVAL	10064609	1
SERUM AMYLOID A PROTEIN INCREASED	10064649	1
OSTEONECROSIS OF JAW	10064658	1
RED BLOOD CELL ACANTHOCYTES PRESENT	10064662	1
GRAFT VERSUS HOST DISEASE IN LIVER	10064676	1
PORTAL VENOUS GAS	10064711	1
ABSCESS MANAGEMENT	10064718	1
THANATOPHOBIA	10064723	1
CSF MONONUCLEAR CELL COUNT INCREASED	10064741	1
BREAST HAEMATOMA	10064753	1
PERIARTHRITIS CALCAREA	10064754	1
SALIVARY GLAND OPERATION	10064759	1
CARDIAC VALVE PROSTHESIS USER	10064776	1
URINARY TRACT INFECTION VIRAL	10064825	1
PUS IN STOOL	10064830	1
CHEMICAL PEEL OF SKIN	10064840	1
INCISION SITE INFLAMMATION	10064903	1
AGE-RELATED MACULAR DEGENERATION	10064930	1
ACINETOBACTER BACTERAEMIA	10064965	1
EYELID HAEMATOMA	10064976	1
NEUTRALISING ANTIBODIES NEGATIVE	10064981	1
RECTAL FISSURE	10064993	1
DRUG EXPOSURE BEFORE PREGNANCY	10064998	1
POST-TRAUMATIC PAIN	10065016	1
PARTNER STRESS	10065022	1
HERPES ZOSTER DISSEMINATED	10065038	1
LOSS OF DREAMING	10065085	1
EYE MUSCLE ENTRAPMENT	10065088	1
DISCOGRAM ABNORMAL	10065094	1
GASTROINTESTINAL ENDOSCOPIC THERAPY	10065100	1
REACTOGENICITY EVENT	10065109	1

GLOBULIN ABNORMAL	10065125	1
VIRAL SKIN INFECTION	10065173	1
LYMPHADENITIS VIRAL	10065209	1
TONSILLITIS BACTERIAL	10065235	1
OBTURATOR NEUROPATHY	10065292	1
INTERVERTEBRAL DISC ANNULAR TEAR	10065294	1
MACULAR RUPTURE	10065319	1
CONTACT LENS THERAPY	10065344	1
MINI MENTAL STATUS EXAMINATION	10065423	1
NEUROPSYCHOLOGICAL TEST ABNORMAL	10065426	1
HYDROGEN BREATH TEST	10065439	1
VENOUS HAEMORRHAGE	10065441	1
ANTI-GLOMERULAR BASEMENT MEMBRANE ANTIBODY POSIT	10065513	1
ANTIENDOMYSIAL ANTIBODY POSITIVE	10065514	1
ANTIMYOCARDIAL ANTIBODY POSITIVE	10065515	1
NASAL MUCOSAL ULCER	10065546	1
RENAL ARTERY ARTERIOSCLEROSIS	10065561	1
THYMUS ENLARGEMENT	10065588	1
PUNCTURE SITE PAIN	10065599	1
STOMA SITE INFLAMMATION	10065669	1
ACTH STIMULATION TEST NORMAL	10065674	1
ACTH STIMULATION TEST ABNORMAL	10065675	1
OCULAR SARCOIDOSIS	10065700	1
PANCREATIC DUCT STENOSIS	10065703	1
RECTAL OBSTRUCTION	10065707	1
LARYNGEAL INFLAMMATION	10065735	1
MUCOSAL INFECTION	10065764	1
CRANIAL NERVE INFECTION	10065765	1
MUSCULOSKELETAL DEFORMITY	10065783	1
FALLOPIAN TUBE OBSTRUCTION	10065789	1
FALLOPIAN TUBE PERFORATION	10065790	1
FISTULA OF SMALL INTESTINE	10065850	1
CENTRAL OBESITY	10065941	1
TOOTH AVULSION	10065953	1
DEVICE ELECTRICAL FINDING	10065963	1
URETHRAL BULKING AGENT INJECTION	10066000	1
CARDIAC AUTONOMIC NEUROPATHY	10066001	1
VACTERL SYNDROME	10066022	1
POST VACCINATION AUTOINOCULATION	10066043	1
PERINEAL CYST	10066058	1
FINE MOTOR DELAY	10066088	1
POLYMORPHIC ERUPTION OF PREGNANCY	10066100	1
IMMOBILISATION PROLONGED	10066112	1

CARDIOPULMONARY BYPASS	10066123	1
EVIDENCE BASED TREATMENT	10066150	1
DIFFUSE AXONAL INJURY	10066213	1
METAPNEUMOVIRUS INFECTION	10066226	1
MEDIASTINAL SHIFT	10066239	1
ABORTION OF ECTOPIC PREGNANCY	10066266	1
PSEUDOHALLUCINATION	10066297	1
LIP HAEMATOMA	10066304	1
GENOTYPE DRUG RESISTANCE TEST POSITIVE	10066368	1
NATUROPATHY	10066398	1
NAPRAPATHY	10066399	1
RENAL-LIMITED THROMBOTIC MICROANGIOPATHY	10085346	1
ENDOCRINE SYSTEM EXAMINATION	10085389	1
VACCINE ASSOCIATED ENHANCED DISEASE	10085491	1
HORMONE RECEPTOR NEGATIVE HER2 POSITIVE BREAST CAN(	10085561	1
HIGH-RESOLUTION COMPUTERISED TOMOGRAM OF LUNG	10085782	1
SOMNIPHOBIA	10085856	1
EAR, NOSE AND THROAT DISORDER	10085881	1
TRAUMATIC HEART INJURY	10085907	1
ULTRASOUND HEAD ABNORMAL	10085916	1
DENGUE VIRUS TEST	10085983	1
NEUROPATHIC PRURITUS	10085997	1
RADIOIMMUNOTHERAPY	10086030	1
GENITAL SCARRING	10086046	1
ANTI-PLATELET FACTOR 4 ANTIBODY NEGATIVE	10086160	1
DERMOSCOPY	10086166	1
CHRONIC INFLAMMATORY RESPONSE SYNDROME	10086201	1
COMBINED TIBIA-FIBULA FRACTURE	10086207	1
ASTROVIRUS TEST	10086214	1
DELAYED UMBILICAL CORD CLAMPING	10086215	1
ANTIPSYCHOTIC THERAPY	10086273	1
VACCINE INDUCED ANTIBODY ABSENT	10086346	1
ROTATOR CUFF TEAR ARTHROPATHY	10086355	1
COMPLETE DECONGESTIVE THERAPY	10086420	1
BONE DEMINERALISATION	10086421	1
AMNIOTIC FLUID INDEX NORMAL	10086422	1
TRIGEMINAL NEUROPATHY	10086511	1
FACTOR VIII ACTIVITY TEST	10086559	1
OLFACTORY DYSFUNCTION	10086567	1
ORAL HERPES ZOSTER	10086594	1
FOLLICULAR DISORDER	10086657	1
IDIOPATHIC ENVIRONMENTAL INTOLERANCE	10086717	1
TENDON SHEATH EFFUSION	10086724	1

PULMONARY BLASTOMYCOSIS	10086752	1
BLOOD PRODUCT REFUSAL	10086776	1
CORNEAL REFLEX TEST	10086820	1
PERIPHERAL VEIN THROMBOSIS	10087102	1
PERIORBITAL DERMATITIS	10087203	1
OROPHARYNGEAL SUCTIONING	10087274	1
MAXILLOFACIAL PAIN	10087335	1
PELVIC FLUID COLLECTION DRAINAGE	10087353	1
COMPUTERISED TOMOGRAM VENOGRAPHY HEAD	10087355	1
INTESTINAL BARRIER DYSFUNCTION	10087367	1
NEUROGENIC COUGH	10087605	1
THALAMIC STROKE	10087626	1
VERTEBROBASILAR ARTERY DISSECTION	10087672	1

Supplementary Table 6. Menstrual adverse events by vaccine type reported in the VAERS database as of 22 November 2022.

Vaccine Type	Vaccine Type Code	Events Reported
		35386
COVID19 VACCINE	COVID19	26714
HUMAN PAPILLOMAVIRUS (TYPES 6, 11, 16, 18) RECOMBINANT VACCINE	HPV4	1198
HEPATITIS B VACCINE	HEP	1013
INFLUENZA VIRUS VACCINE, TRIVALENT (INJECTED)	FLU3(SEASONAL)	581
ZOSTER VACCINE	VARZOS	566
UNKNOWN VACCINES	UNK	394
TETANUS AND DIPHTHERIA TOXOIDS AND ACELLULAR PERTUSSIS VACCINE (BOOSTRIX/ADACEL)	TDAP	368
INFLUENZA VIRUS VACCINE, QUADRIVALENT (INJECTED)	FLU4(SEASONAL)	303
MEASLES, MUMPS AND RUBELLA VIRUS VACCINE, LIVE	MMR	299
HEPATITIS A	HEPA	261
TYPHOID VACCINE	TYP	258
MENINGOCOCCAL VACCINE (MENACTRA)	MNQ	236
INFLUENZA VIRUS VACCINE, NO BRAND NAME	FLUX(SEASONAL)	233
HUMAN PAPILLOMAVIRUS (TYPES 6, 11,16, 18, 31, 33, 45, 52, 58) RECOMBINANT VACCINE	HPV9	216
VARIVAX-VARICELLA VIRUS LIVE	VARCEL	201
PNEUMOCOCCAL VACCINE, POLYVALENT	PPV	188
TETANUS AND DIPHTHERIA TOXOIDS, ADULT	TD	176
COVID19-2	COVID19-2	152
DIPHTHERIA AND TETANUS TOXOIDS AND ACELLULAR PERTUSSIS VACCINE	DTAP	152
RABIES VIRUS VACCINE	RAB	131
HAEMOPHILUS B CONJUGATE VACCINE	HIBV	124
POLIOVIRUS VACCINE INACTIVATED	IPV	124
ANTHRAX VACCINE	ANTH	112
ROTAVIRUS VACCINE, LIVE, ORAL, PENTAVALENT	RV5	97

PNEUMOCOCCAL, 13-VALENT VACCINE (PREVNAR13)	PNC13	95
INFLUENZA (H1N1) MONOVALENT (INJECTED)	FLU(H1N1)	80
POLIOVIRUS VACCINE TRIVALENT, LIVE, ORAL	OPV	78
INFLUENZA VIRUS VACCINE, TRIVALENT (INTRANASAL SPRAY)	FLUN3(SEASONAL)	75
MENINGOCOCCAL B VACCINE	MENB	65
YELLOW FEVER VACCINE	YF	65
DIPHTHERIA AND TETANUS TOXOIDS AND ACELLULAR PERTUSSIS VACCINE + HEPATITIS B + INACTIVATED POLIOVIRUS VACCINE	DTAPHEPBIP	61
DIPHTHERIA AND TETANUS TOXOIDS AND PERTUSSIS VACCINE	DTP	61
PNEUMOCOCCAL, 7-VALENT VACCINE (PREVNAR)	PNC	52
MENINGOCOCCAL POLYSACCHARIDE VACCINE	MEN	48
INFLUENZA VIRUS VACCINE, QUADRIVALENT, CELL-CULTURE-DERIVED (INJECTED)	FLUC4(SEASONAL)	45
TETANUS TOXOID	TTOX	43
INFLUENZA (H1N1) MONOVALENT, (INTRANASAL SPRAY)	FLUN(H1N1)	38
HEPATITIS A AND HEPATITIS B VACCINE	HEPAB	37
LYME VACCINE (LYMERIX)	LYME	37
ROTAVIRUS VACCINE (ROTASHIELD)	RV	36
INFLUENZA(H1N1) MONOVALENT, UNKNOWN MANUFACTURER	FLUX(H1N1)	35
SMALLPOX VACCINE	SMALL	35
DIPHTHERIA AND TETANUS TOXOIDS AND ACELLULAR PERTUSSIS VACCINE + INACTIVATED POLIOVIRUS VACCINE + HAEMOPHILUS B CONJUGATE VACCINE	DTAPIPVHIB	25
DIPHTHERIA AND TETANUS TOXOIDS AND ACELLULAR PERTUSSIS VACCINE + INACTIVATED POLIOVIRUS VACCINE	DTAPIPV	24
HUMAN PAPILLOMAVIRUS VACCINE	HPVX	24

DIPHTHERIA AND TETANUS TOXOIDS, PEDIATRIC	DT	23
INFLUENZA VIRUS VACCINE, QUADRIVALENT (INTRANASAL SPRAY)	FLUN4(SEASONAL)	23
INFLUENZA VIRUS VACCINE, QUADRIVALENT, ADJUVANT (INJECTED)	FLUA4(SEASONAL)	21
MEASLES, MUMPS, RUBELLA, AND VARICELLA VACCINE (PROQUAD)	MMRV	20
INFLUENZA VIRUS VACCINE, TRIVALENT, ADJUVANT (INJECTED)	FLUA3(SEASONAL)	14
ROTAVIRUS VACCINE, LIVE, ORAL	RV1	14
JAPANESE ENCEPHALITIS VIRUS VACCINE	JEV	12
HUMAN PAPILLOVAVIRUS BIVALENT	HPV2	11
INFLUENZA VIRUS VACCINE, QUADRIVALENT, RECOMBINANT (INJECTED)	FLUR4(SEASONAL)	10
JAPANESE ENCEPHALITIS VIRUS VACCINE, INACTIVATED, ADSORBED	JEV1	8
COMVAX	HBHEPB	7
RUBELLA VACCINE	RUB	7
CHOLERA VACCINE	CHOL	6
INFLUENZA VIRUS VACCINE, TRIVALENT, CELL-CULTURE-DERIVED (INJECTED)	FLUC3(SEASONAL)	5
DIPHTHERIA/PERTUSSIS/POLIO (ORAL [LIVE] OR INACTIVATED NOT NOTED)	DPP	4
INFLUENZA VIRUS VACCINE, TRIVALENT, RECOMBINANT (INJECTED)	FLUR3(SEASONAL)	4
MEASLES AND RUBELLA VACCINE	MER	4
ROTAVIRUS (NO BRAND NAME)	RVX	4
SMALLMNK	SMALLMNK	4
ADENOVIRUS TYPE 4 &7 VACCINE, LIVE ORAL	ADEN_4_7	3
EBOLA ZAIRE VACCINE	EBZR	3
MEASLES VACCINE	MEA	3
MUMPS VIRUS VACCINE, LIVE	MU	3
TETRAMUNE	DTAPH	3
DIPHTHERIA AND TETANUS TOXOIDS ACELLULAR PERTUSSIS POLIOVIRUS INACTIVATED HAEMOPHILUS INFLUENZA B AND HEPATITIS B VACCINE (HEXAVAX)	6VAX-F	2

DIPHTHERIA AND TETANUS TOXOIDS PERTUSSIS AND HAEMOPHILUS INFLUENZA B VACCINE (HEXAVAX)	DTPHIB	2
JAPANESE ENCEPHALITIS VIRUS VACCINE (NO BRAND NAME)	JEVX	2
PNEUMOCOCCAL, 20-VALENT VACCINE (PREVNAR20)	PNC20	2
BACILLUS CALMETTE-GUERIN VACCINE	BCG	1
DENGUE TETRAVALENT VACCINE (DENGVAXIA)	DF	1
HAEMOPHILUS B POLYSACCHARIDE VACCINE	HBPV	1
MEASLES AND MUMPS VIRUS VACCINE, LIVE	MM	1
PERTUSSIS, ADSORBED VACCINE	PER	1
TETANUS, DIPHTHERIA AND ACELLULAR PERTUSSIS, AND INACTIVATED POLIO VIRUS	TDAPIPV	1

Supplementary Table 7. Literature search results from PubMed Abstract Sifter.

Landscape View						
		Subject queries:		Menstruation Disturbances OR "menstrual irregularities" OR "menstrual abnormalities" OR (heavy menstrual bleeding OR menstruation irregular OR menstrual disorder OR dysmenorrhoea OR intermenstrual bleeding OR amenorrhoea OR postmenopausal haemorrhage OR premenstrual syndrome OR premenstrual pain OR menstrual discomfort OR menstruation normal OR premenstrual dysphoric disorder OR menstrual cycle management OR premenstrual headache OR retrograde menstruation)	menstrual cycle OR menstruation	menstrual cycle OR menstruation OR Menstruation Disturbances OR "menstrual irregularities" OR "menstrual abnormalities" OR (heavy menstrual bleeding OR menstruation irregular OR menstrual disorder OR dysmenorrhoea OR intermenstrual bleeding OR amenorrhoea OR postmenopausal haemorrhage OR premenstrual syndrome OR premenstrual pain OR menstrual discomfort OR menstruation normal OR premenstrual dysphoric disorder OR menstrual cycle management OR premenstrual headache OR retrograde
		Summary heading		abn menstruation		
**id**	**Preferred Name**	**Chemical / Entity query**	**any**	**Abnormal menses**	**Any menstruation**	**any plus**
IRF1	interferon regulatory factor 1	IRF1 OR interferon regulatory factor 1	3121	0	4	4

STAT1	signal transducer and activator of transcriptio n 1	STAT1 OR signal transducer and activator of transcriptio n 1	11078	6	9	13
RelA	RELA proto-oncogene, NF-kB subunit	RelA OR RELA proto-oncogene, NF-kB subunit OR Transcriptio n Factor RelA	11286	2	13	13
STAT2	signal transducer and activator of transcriptio n 2	STAT2 OR signal transducer and activator of transcriptio n 2 OR STAT2 Transcriptio n Factor	1390	0	1	1
IRF3	interferon regulatory factor 3	IRF3 OR interferon regulatory factor 3	4299	0	0	0

Landscape View

		Subject queries:		Abnormal menses	Any menstruation	any plus
		Subject queries:		Menstruation Disturbances OR "menstrual irregularities" OR "menstrual abnormalities" OR (heavy menstrual bleeding OR menstruation irregular OR menstrual disorder OR dysmenorrhoea OR intermenstrual bleeding OR amenorrhoea OR postmenopausal haemorrhage OR premenstrual syndrome OR premenstrual pain OR menstrual discomfort OR menstruation normal OR premenstrual dysphoric disorder OR menstrual cycle management OR premenstrual headache OR retrograde menstruation)	menstrual cycle OR menstruation	menstrual cycle OR menstruation OR Menstruation Disturbances OR "menstrual irregularities" OR "menstrual abnormalities" OR (heavy menstrual bleeding OR menstruation irregular OR menstrual disorder OR dysmenorrhoea OR intermenstrual bleeding OR amenorrhoea OR postmenopausal haemorrhage OR premenstrual syndrome OR premenstrual pain OR menstrual discomfort OR menstruation normal OR premenstrual dysphoric disorder OR menstrual cycle management OR premenstrual headache OR
		Summary heading		abn menstruation		
**id**	**Preferred Name**	**Chemical / Entity query**	**any**	**Abnormal menses**	**Any menstruation**	**any plus**
TNF	tumor necrosis factor	TNF OR tumor necrosis factor	299917	141	333	421
IL6R	interleukin 6 receptor	IL6R OR interleukin 6 receptor	17710	6	51	54
IL6ST	interleukin 6 cytokine family signal transducer	IL6ST OR interleukin 6 cytokine family signal transducer	1558	1	12	12

LIF	LIF interleukin 6 family cytokine	LIF OR LIF interleukin 6 family cytokine	10794	11	98	101
BIRC3	baculoviral IAP repeat containing 3	BIRC3 OR baculoviral IAP repeat containing 3	743	0	0	0
FGF2	fibroblast growth factor 2	FGF2 OR fibroblast growth factor 2	18497	12	45	47
ARHGDIB	Rho GDP dissociation inhibitor beta	ARHGDIB OR Rho GDP dissociation inhibitor beta	266	0	0	0
RPS3	ribosomal protein S3	RPS3 OR ribosomal protein S3	525	0	0	0
RHOU	ras homolog family member U	RHOU OR ras homolog family member U	656	0	1	1
MIF	macrophage migration inhibitory factor	MIF OR macrophage migration inhibitory factor	7577	10	24	29
STAT4	signal transducer and activator of transcription 4	STAT4 OR signal transducer and activator of transcription 4	1762	0	0	0
TEK	TEK receptor tyrosine kinase	TEK OR TEK receptor tyrosine kinase	3113	4	18	18
CXCR4	C-X-C motif chemokine receptor 4	CXCR4 OR C-X-C motif chemokine receptor 4	15571	4	19	20

GAK	cyclin G associated kinase	GAK OR cyclin G associated kinase	435	2	1	2
ACTN1	actinin alpha 1	ACTN1 OR actinin alpha 1	2035	0	1	1
PGR	progesterone receptor	PGR OR progesterone receptor	45560	796	1927	2286
MFN2	mitofusin 2	MFN2 OR mitofusin 2	2204	0	0	0
EZH2	enhancer of zeste 2 polycomb repressive complex 2 subunit	EZH2 OR enhancer of zeste 2 polycomb repressive complex 2 subunit	5750	1	2	2
AXL	AXL receptor tyrosine kinase	AXL OR AXL receptor tyrosine kinase	2334	2	2	3
IGFBP2	insulin like growth factor binding protein 2	IGFBP2 OR insulin like growth factor binding protein 2	2071	1	23	23
NUB1	negative regulator of ubiquitin like proteins 1	NUB1 OR negative regulator of ubiquitin like proteins 1	382	0	0	0
ICAM1	intercellular adhesion molecule 1	ICAM1 OR intercellular adhesion molecule 1	24759	10	35	35
PSME2	proteasome activator subunit 2	PSME2 OR proteasome activator subunit 2	294	0	1	1

ADM	adrenomedu llin	ADM OR adrenomedu llin	31290	16	23	30
IL1B	interleukin 1 beta	IL1B OR interleukin 1 beta	56099	43	109	128
HIF1A	hypoxia inducible factor 1 subunit alpha	HIF1A OR hypoxia inducible factor 1 subunit alpha	17674	8	31	36
GDI2	GDP dissociation inhibitor 2	GDI2 OR GDP dissociation inhibitor 2	674	0	0	0
PHF19	PHD finger protein 19	PHF19 OR PHD finger protein 19	66	1	0	1
CD1C	CD1c molecule	CD1C OR CD1c molecule	801	2	4	4
CTSW	cathepsin W	CTSW OR cathepsin W	45	0	0	0
KISS1R	KISS1 receptor	KISS1R OR KISS1 receptor	1235	22	16	34
DLK2	delta like non-canonical Notch ligand 2	DLK2 OR delta like non-canonical Notch ligand 2	50	0	0	0
CCL5	C-C motif chemokine ligand 5	CCL5 OR C-C motif chemokine ligand 5	7899	10	21	23

Landscape View Results

		Subject queries:		Menstruation Disturbances OR "menstrual irregularities" OR "menstrual abnormalities" OR (heavy menstrual bleeding OR menstruation irregular OR menstrual disorder OR dysmenorrhoea OR intermenstrual bleeding OR amenorrhoea OR postmenopausal haemorrhage OR premenstrual syndrome OR premenstrual pain OR menstrual discomfort OR menstruation normal OR premenstrual dysphoric disorder OR menstrual cycle management OR premenstrual headache OR retrograde menstruation)	menstrual cycle OR menstruation	menstrual cycle OR menstruation OR Menstruation Disturbances OR "menstrual irregularities" OR "menstrual abnormalities" OR (heavy menstrual bleeding OR menstruation irregular OR menstrual disorder OR dysmenorrhoea OR intermenstrual bleeding OR amenorrhoea OR postmenopausal haemorrhage OR premenstrual syndrome OR premenstrual pain OR menstrual discomfort OR menstruation normal OR premenstrual dysphoric disorder OR menstrual cycle management OR premenstrual headache OR retrograde
		Summary heading		abn menstruation		
**id**	**Preferred Name**	**Chemical / Entity query**	**any**	**Abnormal menses**	**Any menstruation**	**any plus**
TNF	tumor necrosis factor	TNF OR tumor necrosis factor	299917	141	333	421
VEGFA	vascular endothelial growth factor A	VEGFA OR vascular endothelial growth factor A	60492	84	281	319
IL1B	interleukin 1 beta	IL1B OR interleukin 1 beta	56093	43	109	128
CASP3	caspase 3	CASP3 OR caspase 3	79456	19	53	64

IL6R	interleukin 6 receptor	IL6R OR interleukin 6 receptor	17707	6	51	54
NOS2	nitric oxide synthase 2	NOS2 OR nitric oxide synthase 2	55806	31	49	71
CDH1	cadherin 1	CDH1 OR cadherin 1	28976	16	47	51
TERT	telomerase reverse transcriptase	TERT OR telomerase reverse transcriptase	52555	11	41	45
CCL2	C-C motif chemokine ligand 2	CCL2 OR C-C motif chemokine ligand 2	19554	13	41	44
MIF	macrophage migration inhibitory factor	MIF OR macrophage migration inhibitory factor	7577	10	24	29
FLT1	fms related receptor tyrosine kinase 1	FLT1 OR fms related receptor tyrosine kinase 1	2774	2	17	17
PPARA	peroxisome proliferator activated receptor alpha	PPARA OR peroxisome proliferator activated receptor alpha	20505	2	8	9
AKT1	AKT serine/threonine kinase 1	AKT1 OR AKT serine/threonine kinase 1	11251	4	8	10
CREB1	cAMP responsive element binding protein 1	CREB1 OR cAMP responsive element binding protein 1	8688	1	7	8

DRD1	dopamine receptor D1	DRD1 OR dopamine receptor D1	13266	3	5	6
E2F1	E2F transcription factor 1	E2F1 OR E2F transcription factor 1	6463	0	5	5
BMPR2	bone morphogenetic protein receptor type 2	BMPR2 OR bone morphogenetic protein receptor type 2	3247	4	3	7
PARP1	poly(ADP-ribose) polymerase 1	PARP1 OR poly(ADP-ribose) polymerase 1	13191	2	3	4
NFE2L2	NFE2 like bZIP transcription factor 2	NFE2L2 OR NFE2 like bZIP transcription factor 2	9260	1	2	2
S100A6	S100 calcium binding protein A6	S100A6 OR S100 calcium binding protein A6	599	0	2	2
MX2	MX dynamin like GTPase 2	MX2 OR MX dynamin like GTPase 2	902	0	2	2
TXNIP	thioredoxin interacting protein	TXNIP OR thioredoxin interacting protein	1840	1	1	2
GNAS	GNAS complex locus	GNAS OR GNAS complex locus	1730	3	1	4
GNAI2	G protein subunit alpha i2	GNAI2 OR G protein subunit alpha i2	627	0	1	1

TPT1	tumor protein, translationally-controlled 1	TPT1 OR tumor protein, translationally-controlled 1	770	0	1	1
MIR576	microRNA 576	MIR576 OR microRNA 576	150	0	1	1
FTO	FTO alpha-ketoglutarate dependent dioxygenase	FTO OR FTO alpha-ketoglutarate dependent dioxygenase	4000	2	1	2
CD274	CD274 molecule	CD274 OR CD274 molecule	8284	0	1	1
MAPK14	mitogen-activated protein kinase 14	MAPK14 OR mitogen-activated protein kinase 14	1373	1	0	1
MIR516A2	microRNA 516a-2	MIR516A2 OR microRNA 516a-2	2	0	0	0
MIR516A1	microRNA 516a-1	MIR516A1 OR microRNA 516a-1	1	0	0	0
MIR488	microRNA 488	MIR488 OR microRNA 488	229	0	0	0
AHSG	alpha 2-HS glycoprotein	AHSG OR alpha 2-HS glycoprotein	1408	0	0	0
IFI44	interferon induced protein 44	IFI44 OR interferon induced protein 44	1374	0	0	0
SERPINF2	serpin family F member 2	SERPINF2 OR serpin family F member 2	55	0	0	0

Supplementary Table 8. Differentially expressed genes in response to treatment with COVID-19 vaccine BNT162b2.

Gene	Log2FC	FDR
IDO1	8.44	1.26E-05
LAMP3	8.36	1.26E-05
CCR5AS	8.20	5.35E-05
TNFAIP6	7.91	7.82E-05
CXCL10	7.15	4.46E-05
CCRL2	7.02	9.17E-05
GRIN3A	6.98	1.75E-04
RSPH9	6.60	1.88E-05
CCL2	6.55	2.11E-04
MDK	6.49	3.35E-04
OAS2	6.37	3.39E-04
SEPTIN4	6.35	3.55E-04
SOCS1	6.31	3.87E-04
IFIT5	6.28	3.39E-04
USP18	6.20	4.32E-04
P2RY14	6.14	3.77E-04
PDCD1LG2	6.08	2.73E-04
MSR1	5.92	6.35E-04
EFCAB2	5.91	6.38E-04
DDX58	5.90	6.11E-04
APOL4	5.86	1.74E-04
ANKRD22	5.77	1.75E-04
CD274	5.75	5.03E-04
DDX60	5.75	6.65E-04
GBP1P1	5.74	1.49E-04
RSAD2	5.72	3.39E-04
TOR1B	5.71	5.53E-05
NCOA7	5.70	1.80E-06
TNFSF10	5.68	6.82E-04
PLSCR1	5.67	8.33E-04
CMPK2	5.66	6.80E-04
RTP4	5.62	7.83E-04
CALHM6	5.61	7.14E-04
ATF3	5.53	5.96E-04
DHX58	5.50	7.66E-04
H4C8	5.43	5.35E-05
TIFA	5.35	9.40E-04
GCH1	5.34	9.35E-04
HERC5	5.32	1.16E-03
RGL1	5.27	3.89E-04
TMEM268	5.27	3.34E-04

CLEC6A	5.24	1.34E-03
AIM2	5.22	1.23E-03
IFITM3P2	5.21	7.49E-04
GBP6	5.18	9.38E-04
TDRD7	5.18	5.64E-04
IFIT1	5.17	1.34E-03
RMI2	5.15	1.22E-03
MOV10	5.15	4.93E-04
IFIH1	5.13	1.29E-03
TRIM22	5.11	1.46E-03
CEACAM1	5.11	1.55E-03
ISG15	5.09	1.38E-03
TFEC	5.08	1.13E-03
OASL	5.02	1.85E-03
PNPT1	5.00	6.09E-05
IFIT3	5.00	1.77E-03
IFIT2	4.97	1.96E-03
SAT1	4.93	3.34E-04
GAS6	4.92	3.39E-04
FAS	4.91	1.75E-03
OAS1	4.90	2.15E-03
LHFPL2	4.87	2.10E-03
IFI44L	4.86	1.59E-03
MX1	4.84	2.30E-03
TRIM6	4.84	2.30E-03
MRPL44	4.84	2.65E-04
SMTNL1	4.83	2.30E-03
DDX60L	4.79	1.28E-03
KIAA1958	4.79	3.89E-04
IFITM3	4.76	2.55E-03
GALM	4.76	1.91E-04
TMEM252	4.74	1.22E-03
GBP5	4.72	2.25E-03
IL1RN	4.72	5.12E-04
VAMP5	4.71	2.56E-03
MICB	4.71	7.49E-04
SPATS2L	4.67	2.30E-03
OAS3	4.67	2.50E-03
SERPING1	4.66	1.59E-03
SAMD9	4.66	8.14E-04
SAMD9L	4.65	2.77E-03
STAT2	4.65	2.42E-03
EIF2AK2	4.64	2.11E-03
PARP12	4.63	1.54E-03
HORMAD1	4.62	2.13E-03

LAP3	4.61	2.91E-03
G0S2	4.58	2.17E-03
CARD17	4.56	3.02E-03
IFI6	4.55	3.17E-03
FBXO6	4.55	3.06E-03
STK3	4.55	2.23E-03
TNFSF13B	4.55	2.30E-03
IFI44	4.54	3.25E-03
CD38	4.54	3.04E-04
WDFY1	4.53	8.98E-04
PARP11	4.52	1.09E-04
WARS1	4.48	3.45E-03
TMEM123	4.47	2.90E-04
IRF7	4.46	3.17E-03
ZBP1	4.43	2.42E-03
XAF1	4.41	3.87E-03
TANK	4.35	1.18E-04
EPSTI1	4.35	4.24E-03
TRIM5	4.33	2.30E-03
DAPP1	4.33	1.11E-03
PARP9	4.32	3.81E-03
GRAMD1B	4.32	2.22E-03
IRAK2	4.31	5.12E-04
BISPR	4.31	1.82E-03
MASTL	4.30	5.78E-04
CCL8	4.29	1.11E-03
GBP1	4.26	3.98E-03
ISG20	4.26	2.68E-03
MX2	4.26	8.33E-04
IFI16	4.25	2.36E-03
CHMP5	4.25	3.68E-03
CLEC5A	4.24	1.54E-03
METTL7B	4.20	4.45E-03
FCGR1CP	4.20	3.21E-03
GK	4.18	4.89E-03
DDIAS	4.17	2.41E-03
APOBEC3F	4.13	1.56E-04
RIPK2	4.13	2.55E-03
SCARF1	4.11	6.06E-03
NBN	4.10	1.43E-04
LYSMD2	4.10	1.02E-03
CMTR1	4.10	2.42E-03
BCL2L14	4.08	3.55E-03
DYNLT1	4.08	2.42E-03
IFI27	4.06	5.28E-03

GPR84	4.06	6.21E-03
PPP1R2P1	4.05	4.45E-03
DOCK4	4.03	6.00E-03
PML	4.02	6.41E-03
PHF11	4.02	6.44E-04
STAT1	4.01	6.76E-03
STX11	4.01	5.10E-03
XRN1	4.00	4.16E-03
HNRNPLL	3.99	6.09E-05
MFSD14B	3.98	2.17E-03
PARP14	3.94	7.79E-03
HCAR3	3.93	6.25E-03
LPCAT2	3.91	7.08E-03
BATF2	3.91	5.04E-03
GADD45B	3.90	3.21E-03
KLF5	3.89	7.56E-03
VAMP3	3.89	1.11E-03
ASPRV1	3.88	3.46E-03
ALMS1P1	3.88	4.40E-03
UBE2L6	3.86	8.54E-03
LACTB	3.86	4.45E-03
EXOC3L1	3.86	8.56E-03
CD300LD	3.86	8.43E-03
STOM	3.85	8.94E-04
VCPIP1	3.84	2.73E-03
SAMD4A	3.84	8.86E-03
SCML1	3.84	3.35E-04
IFITM1	3.84	5.04E-03
POLB	3.84	2.85E-03
H2BC21	3.84	2.74E-04
GALNT3	3.83	1.20E-03
ANKFY1	3.83	1.25E-03
TAP1	3.83	7.88E-03
ETV7	3.83	4.65E-03
KPTN	3.82	3.66E-03
GM2A	3.81	1.87E-03
GBP4	3.81	9.08E-03
IFI35	3.81	9.55E-03
ASPHD2	3.81	4.53E-03
CD59	3.78	3.80E-03
AIG1	3.78	3.35E-04
NUB1	3.77	3.21E-03
H2BP2	3.76	3.18E-03
LYRM1	3.75	5.12E-04
APOBEC3B	3.75	1.01E-02

APOL6	3.75	1.02E-02
RUFY4	3.74	1.05E-02
RAB33B	3.74	3.21E-03
TASL	3.74	1.22E-03
SLC26A8	3.72	7.36E-03
CSRNP1	3.71	2.30E-03
FANCA	3.70	6.37E-03
H2BC4	3.70	1.01E-03
NRIR	3.70	6.95E-03
ICAM1	3.70	9.08E-03
H3C6	3.70	5.42E-03
EPB41L5	3.68	4.65E-03
MMP8	3.68	1.15E-02
RBCK1	3.68	5.59E-03
RASGRP3	3.67	4.93E-04
SLAMF8	3.67	1.17E-02
ATG3	3.66	2.80E-03
APOBEC3A	3.66	1.05E-02
RALB	3.66	4.06E-03
SP140	3.66	4.00E-03
APOL1	3.65	1.17E-02
PSTPIP2	3.65	1.14E-02
DTX3L	3.65	7.96E-03
SP100	3.65	3.87E-04
NCF1B	3.64	3.75E-03
ARL5B	3.63	1.01E-03
PRRG4	3.63	9.56E-03
CNDP2	3.63	2.95E-03
SOD2	3.62	8.07E-03
SELL	3.62	3.41E-03
MR1	3.62	6.38E-04
PLAAT4	3.61	8.86E-03
CREG1	3.61	4.07E-03
USP25	3.60	3.47E-04
SRGAP2C	3.60	4.48E-04
SRBD1	3.59	1.19E-04
SQOR	3.57	3.80E-03
SAMHD1	3.57	2.14E-03
BLZF1	3.56	1.75E-04
SEC24D	3.56	3.14E-03
FCGR1A	3.56	9.56E-03
GBP3	3.56	8.53E-03
MTHFD2	3.55	3.55E-03
MIA3	3.55	9.34E-04
TENT5A	3.55	1.21E-03

TRIM69	3.55	1.96E-03
PI4K2B	3.53	3.87E-03
ZNF200	3.49	2.30E-03
C1GALT1	3.48	1.01E-03
IL27	3.47	1.61E-02
CALCOCO2	3.46	2.73E-03
UBE2S	3.46	3.23E-03
ERAP2	3.45	3.05E-03
NCF1C	3.44	6.86E-03
CASP4	3.44	4.18E-03
TTC21A	3.44	2.06E-03
IL15RA	3.44	5.60E-03
SIRPD	3.43	6.23E-03
HCAR2	3.43	1.14E-02
PSME2P2	3.43	1.50E-02
PSME2	3.42	1.48E-02
CASP1	3.42	4.32E-03
C3AR1	3.42	4.06E-03
SMCO4	3.42	1.38E-02
SNX10	3.42	4.80E-03
CCR1	3.42	5.58E-03
TRAFD1	3.41	1.45E-02
B3GNT5	3.41	4.25E-03
ZC3HAV1	3.41	1.53E-03
PELI1	3.39	3.58E-03
PIK3AP1	3.38	7.70E-03
ODF3B	3.37	1.42E-02
CASP5	3.37	1.91E-02
RHBDF2	3.37	1.48E-02
SLAMF7	3.36	2.77E-03
PLAUR	3.36	5.13E-03
SORT1	3.36	1.63E-02
DCUN1D3	3.36	6.37E-03
GNB4	3.36	5.29E-03
MYOF	3.35	1.98E-02
CD40	3.35	3.98E-03
TRIM38	3.34	1.04E-03
PSME2P6	3.33	2.01E-02
C3orf14	3.33	5.46E-03
SCARB2	3.33	8.18E-04
ACTA2	3.33	9.99E-03
FCGR1B	3.32	1.91E-02
VRK2	3.32	4.06E-03
SECTM1	3.32	2.10E-02
TMEM140	3.31	1.31E-02

PLAC8	3.30	8.33E-04
KCNJ15	3.30	1.46E-02
H4C14	3.29	4.37E-03
CD46	3.29	2.51E-03
PDE4B	3.28	4.16E-03
DISC1	3.28	4.48E-03
FCGR3B	3.28	5.07E-03
SRGAP2B	3.28	3.66E-03
C9orf72	3.28	6.97E-03
CDK17	3.27	1.02E-03
GPD2	3.27	3.31E-03
JAK2	3.26	6.95E-03
ZCCHC2	3.26	1.36E-02
MAD2L1BP	3.26	5.85E-05
FGL2	3.25	4.50E-03
ZNF684	3.25	4.50E-03
TAP2	3.24	1.42E-02
FLVCR2	3.24	1.04E-02
C5orf15	3.24	3.55E-03
KIAA0040	3.24	5.46E-03
FCGR2B	3.23	1.30E-03
IRF2	3.23	2.95E-03
HES4	3.22	1.99E-02
C2	3.21	2.51E-02
IGFLR1	3.21	1.02E-02
LGALS3BP	3.21	1.46E-02
HERC6	3.20	5.17E-03
LMO2	3.20	3.51E-03
KARS1	3.20	5.76E-03
PPA1	3.20	1.72E-03
AFF1	3.19	1.03E-02
FAR2	3.19	3.51E-03
DUSP3	3.19	5.05E-03
RUBCN	3.19	1.87E-03
ERP44	3.19	1.30E-03
PIM2	3.18	4.74E-03
CYB5R4	3.18	1.61E-03
H2BC18	3.18	1.18E-02
SLC6A12	3.17	2.46E-02
PRPS2	3.17	2.51E-03
ADAR	3.16	6.37E-03
ERI1	3.16	2.68E-03
PSME2P1	3.16	2.69E-02
ACOT9	3.15	7.66E-04
SPPL2A	3.14	6.65E-04

BAZ1A	3.14	7.46E-03
ADM	3.14	7.11E-03
CASP10	3.14	3.47E-04
PLA2G7	3.14	9.86E-03
IRF1	3.13	2.01E-02
EIF4E3	3.13	1.43E-03
SESTD1	3.13	4.10E-03
ACO1	3.12	6.25E-03
HSH2D	3.12	4.07E-03
C18orf25	3.12	8.35E-03
ELOVL5	3.11	5.90E-03
FRMD3	3.11	2.48E-02
P2RY6	3.10	1.93E-02
NPC2	3.10	1.34E-02
GLRX	3.10	9.08E-03
LONRF1	3.09	4.57E-03
ARFIP1	3.09	2.06E-03
DRAM1	3.09	3.70E-03
TNF	3.07	2.57E-03
ANXA3	3.07	2.86E-02
VPS9D1	3.07	2.87E-02
PLA2G4A	3.06	1.42E-02
TCN2	3.06	2.69E-02
PATL1	3.06	2.98E-03
DPYD	3.06	2.17E-03
ALPK1	3.05	1.10E-02
SCO2	3.05	2.59E-02
B2M	3.05	4.96E-03
MLKL	3.04	1.25E-02
NOD2	3.04	2.50E-02
STX3	3.04	3.79E-03
ZNF366	3.04	2.81E-02
KCNK13	3.04	1.42E-02
ITPRID2	3.04	9.34E-03
ARSB	3.03	2.77E-03
PLEK	3.03	8.00E-03
SHISA5	3.03	1.03E-02
DHRS9	3.03	2.12E-02
GNS	3.02	7.74E-03
DHRS12	3.02	1.41E-02
TESK2	3.02	1.51E-02
RGS1	3.02	3.51E-02
MTMR11	3.02	4.50E-03
SNX20	3.02	8.90E-03
LRRK2	3.01	9.52E-03

C1QB	3.01	3.59E-02
HAPLN3	3.01	6.09E-03
SETP11	3.01	3.46E-02
APOBEC3G	3.01	2.15E-03
GBP2	3.01	3.08E-02
CARD16	2.99	1.81E-02
P2RY12	2.99	2.62E-02
ADCY3	2.99	2.76E-03
CFAP58	2.98	2.98E-02
ZNF117	2.98	1.63E-02
UNC93B1	2.98	5.58E-03
KIAA0319L	2.97	6.38E-04
MOB1A	2.97	9.38E-04
OSM	2.97	1.05E-02
USP6NL	2.97	3.73E-03
PSME1	2.97	1.40E-02
HINT3	2.96	2.30E-03
NMI	2.96	1.71E-02
TRPV4	2.96	3.88E-02
SHFL	2.96	1.84E-03
CLIC4	2.95	5.76E-03
GTF2B	2.95	6.75E-04
MAFF	2.95	2.84E-02
MT2A	2.94	3.51E-02
GRASLND	2.93	3.34E-02
CD2AP	2.93	6.95E-03
KIF23	2.92	2.15E-02
TRIM21	2.92	2.16E-02
FFAR2	2.92	1.59E-02
MCTP1	2.91	1.94E-03
ACSL4	2.91	1.50E-02
CAPZA2	2.91	1.01E-02
TLR7	2.91	1.65E-02
C3orf38	2.90	5.78E-03
WSB2	2.90	5.59E-03
ZNF496	2.90	3.41E-03
ZNF702P	2.90	3.38E-02
FGF13	2.90	4.38E-02
PANK2	2.89	1.15E-03
ZNF267	2.89	2.03E-02
STN1	2.89	1.68E-02
BST2	2.88	3.21E-02
NRN1	2.88	3.21E-02
SHOX2	2.87	4.50E-02
AZI2	2.87	5.03E-04

PSMB8	2.87	1.85E-02
CYBB	2.87	2.12E-02
GOLM1	2.87	7.36E-03
NCF1	2.87	3.46E-02
GIMAP2	2.86	1.58E-02
GPR65	2.86	8.86E-03
ATP6V1G1	2.86	4.79E-03
FYTTD1	2.86	1.23E-03
CAMK2D	2.86	2.11E-03
CEP162	2.86	4.73E-03
CLEC7A	2.85	4.24E-03
SRGN	2.85	4.85E-03
HELZ2	2.85	3.24E-02
SLC31A2	2.85	8.72E-03
NUCB1	2.85	3.21E-02
MBOAT2	2.84	1.30E-02
CASP7	2.83	2.01E-02
ITPRIPL2	2.83	2.60E-02
PRR11	2.83	1.52E-02
H3P14	2.83	3.10E-02
TMEM60	2.83	1.39E-03
SLK	2.82	1.46E-02
TAGAP	2.82	8.56E-03
GIMAP4	2.82	5.28E-03
NUDT16L2P	2.81	1.70E-02
ATP1B2	2.81	3.42E-02
TOR1A	2.81	6.38E-04
DUSP5	2.81	2.81E-02
CD53	2.81	4.80E-03
RBM7	2.81	7.85E-04
PSMA4	2.81	2.40E-02
ZDHHC19	2.80	4.61E-02
IL31RA	2.80	3.81E-02
EIF4G3	2.80	1.62E-02
SMIM14	2.80	1.55E-04
ZNF438	2.80	1.63E-02
CXCL9	2.79	2.97E-02
NFIL3	2.78	3.63E-03
MICALL1	2.78	6.37E-03
CD36	2.78	1.03E-02
UBE2D1	2.78	1.22E-02
TLR8	2.78	1.42E-02
LY6E	2.77	4.65E-02
ZNFX1	2.77	2.19E-02
RHOT1	2.77	3.41E-03

SNX6	2.77	2.90E-04
NLRC5	2.77	1.91E-02
CNP	2.77	6.75E-04
CCL4L2	2.77	2.22E-02
RAB20	2.77	4.54E-02
APOL2	2.76	4.73E-02
IL1B	2.76	1.10E-02
DUSP6	2.76	6.78E-03
CBWD2	2.75	1.10E-02
C5	2.75	4.14E-02
IL15	2.74	2.86E-02
SP110	2.74	1.52E-02
MAT2B	2.74	1.63E-02
RICTOR	2.74	2.82E-03
TAPBP	2.73	3.14E-03
C1GALT1C1	2.73	1.80E-02
SUCNR1	2.73	5.22E-02
CXCL16	2.73	3.57E-02
RAB8B	2.73	2.52E-03
SLC22A4	2.73	3.79E-03
CLEC4E	2.72	1.80E-02
CGAS	2.72	1.17E-02
CACNA1A	2.71	4.88E-02
KYNU	2.71	1.36E-02
CCDC13	2.71	2.43E-02
ARMH1	2.70	3.41E-03
ABHD16A	2.70	4.32E-03
PSMB9	2.70	4.43E-02
CYRIB	2.70	1.96E-03
FYB1	2.69	1.71E-02
LYPLA1	2.69	1.67E-03
COQ10B	2.69	6.10E-03
SLC22A15	2.69	1.13E-02
RRM2B	2.69	9.97E-03
MYD88	2.68	1.59E-02
FOLR2	2.68	2.96E-02
SRGAP2	2.68	1.01E-02
CPEB2	2.68	3.23E-03
BTN3A1	2.68	5.72E-03
EFR3A	2.68	1.12E-03
SSB	2.68	3.82E-03
RP2	2.67	1.78E-02
LMNB1	2.67	3.72E-02
TLDC2	2.66	3.88E-02
N4BP2L1	2.66	2.42E-03

MAN1A1	2.66	9.97E-03
PARP10	2.66	3.62E-02
PMAIP1	2.65	1.36E-02
CMAHP	2.65	1.21E-02
CBWD3	2.65	3.65E-02
ECE1	2.65	5.04E-03
SCIMP	2.65	1.39E-02
LYN	2.65	1.20E-02
GTPBP1	2.65	1.30E-02
MITD1	2.64	1.34E-02
IL18BP	2.64	9.72E-03
SIPA1L1	2.63	3.08E-02
CD164	2.63	7.79E-03
H3P6	2.62	1.37E-02
UBE2D3	2.61	1.01E-03
RAB24	2.61	1.53E-02
TNFAIP3	2.60	3.87E-03
N4BP1	2.60	1.72E-03
USP30	2.60	7.96E-03
ITM2B	2.59	5.46E-03
CTSS	2.59	3.58E-03
PRKAG2	2.59	3.06E-04
C12orf4	2.59	2.83E-02
LGALS9	2.58	4.62E-02
FIG4	2.58	4.45E-03
EDEM2	2.58	8.27E-03
BTN2A2	2.58	9.35E-03
RAB5A	2.58	4.89E-03
SMCHD1	2.58	1.40E-02
PUS3	2.58	1.27E-02
CAPZA1	2.57	9.13E-03
LCP2	2.57	1.79E-02
STAM2	2.57	3.82E-03
CDS2	2.56	1.44E-02
UBE2D3P1	2.56	2.28E-02
SLC35A5	2.56	1.50E-02
SERPINB9	2.55	1.64E-02
SLFN5	2.55	2.52E-03
HERPUD2	2.55	5.16E-04
RAB1A	2.55	1.67E-03
GNA13	2.54	8.45E-03
FCGR2A	2.54	1.95E-02
SEPHS2	2.54	1.39E-02
LGALS8	2.54	8.60E-03
PTK2B	2.54	3.54E-03

MAPK14	2.54	1.37E-02
HEXD	2.53	1.11E-03
TUT7	2.53	1.43E-02
PFKFB2	2.53	3.43E-02
FBXL5	2.52	1.40E-02
NT5C2	2.52	3.16E-02
CMTM6	2.52	2.07E-02
AP1M2	2.52	4.51E-02
ETV6	2.51	2.58E-02
IMPA1	2.51	3.26E-03
CTSL	2.51	5.48E-02
SH3GLB1	2.51	1.52E-03
SLC49A3	2.50	4.85E-02
USP15	2.50	1.52E-02
COP1	2.50	4.90E-03
C4orf3	2.50	7.06E-03
TLR1	2.49	2.46E-02
NT5C3A	2.49	2.36E-03
SERPINB1	2.49	9.52E-03
HPSE	2.48	1.51E-02
RAP2C	2.48	3.72E-03
ARL11	2.48	4.16E-03
ATP6V1B2	2.48	2.43E-02
MXD1	2.48	1.49E-02
IRF9	2.48	3.77E-02
MOB3C	2.47	3.66E-03
LMTK2	2.47	4.60E-02
RASGEF1B	2.47	1.20E-02
APOL3	2.47	4.40E-02
C19orf12	2.47	2.73E-03
MED12L	2.47	2.86E-02
ACTR3C	2.46	3.04E-02
OSGIN2	2.46	2.10E-02
GCNT2	2.46	1.46E-02
AP1AR	2.46	2.89E-02
PLAGL1	2.46	3.61E-02
FCER1G	2.46	2.85E-02
DESI1	2.46	6.62E-03
ARRDC3	2.45	1.54E-02
TRANK1	2.45	4.97E-02
GALC	2.45	1.91E-02
CD300A	2.45	2.17E-02
PRR5L	2.45	5.27E-02
KDSR	2.45	1.92E-02
UBQLN2	2.45	1.76E-02

TVP23B	2.44	3.59E-02
PLGRKT	2.44	1.00E-02
MORC3	2.44	2.02E-03
UBE2W	2.44	1.68E-02
MYL12A	2.44	7.18E-04
ITPRIP	2.44	2.65E-02
SLC25A28	2.44	1.70E-02
DTNBP1	2.44	8.36E-03
IFNGR2	2.44	5.01E-03
PTGS2	2.43	4.19E-02
DMXL2	2.43	3.16E-02
GSTK1	2.43	1.42E-02
C1D	2.43	1.89E-02
ELF1	2.43	3.48E-04
HIF1A	2.43	8.00E-03
CCDC146	2.43	3.48E-02
TM9SF2	2.42	1.44E-02
HTATIP2	2.42	2.91E-03
HEBP1	2.42	1.42E-02
CIR1	2.42	2.91E-02
IRF5	2.41	2.22E-02
ZNF396	2.41	3.12E-02
DEGS1	2.41	7.56E-03
BTN2A1	2.41	7.56E-03
ANKRD13A	2.41	6.96E-03
CUL1	2.41	1.82E-02
FCAR	2.41	2.70E-02
GLS	2.41	2.21E-02
TPMT	2.40	1.37E-02
GSTO1	2.40	2.13E-02
CARS2	2.40	1.79E-02
ZFP36	2.40	2.48E-02
GCNT1	2.40	1.20E-02
TLR4	2.40	1.68E-02
PILRA	2.40	3.43E-02
TMEM59	2.40	6.37E-03
NFKBIE	2.39	1.81E-02
POMP	2.39	1.42E-02
NECAP1	2.39	2.60E-03
CXCR2P1	2.39	5.30E-02
WSB1	2.38	3.79E-03
CYSTM1	2.38	2.15E-02
SCRN3	2.38	3.03E-03
H2AC6	2.37	3.22E-03
PIGB	2.37	2.23E-03

ECT2	2.37	5.15E-02
TOR1AIP1	2.37	8.59E-03
TRIM14	2.37	5.47E-03
TLR2	2.37	2.88E-02
NXT2	2.37	3.93E-02
TBK1	2.37	4.44E-02
GSDMD	2.36	3.54E-02
MED28	2.36	9.38E-04
TMX1	2.36	3.85E-02
MEFV	2.36	3.90E-02
CCR2	2.36	2.60E-02
RAB5IF	2.36	4.38E-02
BLOC1S6	2.36	1.10E-02
SCLT1	2.36	9.30E-03
EDEM1	2.36	1.17E-02
PRKCD	2.36	2.94E-02
ELL2	2.36	3.55E-03
STARD3NL	2.35	1.15E-02
JUNB	2.35	1.91E-02
GPR141	2.35	1.46E-02
LILRA5	2.35	2.78E-02
HSD17B11	2.35	1.12E-02
RBM43	2.35	2.48E-02
ATP1B3	2.34	1.61E-02
MEF2A	2.34	3.86E-03
C4orf33	2.34	1.99E-02
RBM23	2.34	1.23E-02
TMEM30A	2.34	3.72E-02
SDE2	2.34	2.76E-02
BCL10	2.34	2.15E-03
SPTLC2	2.34	4.94E-03
BID	2.34	1.55E-02
STK24	2.34	1.70E-02
FGFR1OP2	2.34	3.45E-03
ZFYVE26	2.34	1.43E-02
MAP3K8	2.34	2.30E-03
FEM1C	2.34	3.21E-02
RELB	2.34	3.15E-02
GPBP1	2.33	1.86E-02
TMEM33	2.33	2.26E-03
MAP2K6	2.33	2.14E-03
GPN3	2.33	1.12E-02
LAMP2	2.32	1.49E-02
CTBS	2.32	1.18E-02
TSC22D1	2.32	1.63E-02

CHMP2B	2.32	5.71E-03
VWA3B	2.32	5.29E-02
RFX5	2.32	2.17E-02
SRP54	2.32	1.14E-03
LIMK1	2.32	3.59E-02
RAB8A	2.31	5.03E-03
RAB31	2.31	2.78E-02
NFE2L2	2.31	6.95E-03
NDUFB6	2.31	2.43E-02
IL12RB1	2.31	1.08E-02
RAB10	2.31	1.88E-03
SLC7A7	2.31	2.36E-02
QKI	2.31	1.92E-03
GMPR2	2.31	6.67E-03
USP33	2.31	5.32E-03
TOP1	2.31	4.68E-02
NOD1	2.30	2.62E-02
ZNF273	2.30	4.26E-02
NIPSNAP3A	2.29	3.71E-02
DRAP1	2.29	4.02E-02
ATXN7	2.29	3.21E-02
NFKBIA	2.29	3.08E-02
SKIL	2.29	1.83E-02
JPX	2.29	2.19E-02
EPHB1	2.29	2.96E-02
DENND1A	2.29	5.24E-02
TFG	2.29	7.96E-03
SIGLEC5	2.29	4.54E-02
H2BC5	2.29	2.62E-02
TMEM165	2.28	4.32E-03
CREM	2.28	1.92E-02
PTPRC	2.28	6.67E-03
TMEM154	2.28	7.56E-03
SCYL2	2.28	1.16E-02
CBR1	2.28	2.08E-02
RIPK1	2.28	1.13E-02
BMPR2	2.28	6.36E-03
SFT2D1	2.28	5.46E-03
FNDC3A	2.28	1.38E-02
FRMD4B	2.27	3.15E-02
RPAP3	2.27	8.59E-03
ABCA1	2.27	3.21E-02
CNIH4	2.27	4.00E-02
DENND1B	2.27	2.37E-03
MTF1	2.27	1.80E-02

NTNG2	2.27	5.43E-02
ZC3H12A	2.27	1.66E-02
LILRB3	2.27	5.24E-02
MMADHC	2.26	3.80E-03
CLEC4A	2.26	7.98E-03
GCA	2.26	5.06E-02
HAUS4	2.26	2.41E-02
DGLUCY	2.26	1.38E-02
DECR1	2.26	1.06E-02
ELF4	2.26	3.36E-02
DIP2B	2.26	5.03E-03
ELK1	2.25	9.63E-03
AP1S2	2.25	3.71E-02
NRBF2	2.25	1.76E-02
C21orf91	2.25	1.83E-02
PAK1	2.25	3.48E-02
PRKAR1A	2.25	3.59E-02
LAT2	2.25	1.83E-02
C6orf62	2.24	5.03E-04
HDAC4	2.23	4.53E-02
LCP1	2.23	4.00E-02
PARP3	2.23	3.92E-02
LILRB1	2.23	3.90E-02
TIMM10	2.23	1.85E-02
OSTM1	2.22	1.62E-02
NDC80	2.22	4.17E-02
KMO	2.22	3.11E-02
C1orf52	2.22	2.30E-03
STX17	2.22	8.57E-03
SLC40A1	2.22	3.40E-02
FCGR3A	2.21	3.04E-02
ATP6V1C1	2.21	8.72E-03
IRF8	2.21	1.71E-02
CD74	2.21	3.04E-02
MTMR6	2.21	1.02E-02
EXT1	2.21	9.45E-03
PHACTR2	2.20	8.21E-03
ARL8B	2.20	1.06E-03
SLC30A7	2.20	1.53E-02
MILR1	2.20	1.86E-02
TATDN3	2.20	3.41E-03
CLIC1	2.20	1.96E-02
FMR1	2.20	4.32E-02
CDC42SE2	2.20	1.70E-02
RHOQ	2.19	2.85E-02

SERTAD3	2.19	1.01E-02
BROX	2.19	5.03E-03
GVINP1	2.19	3.23E-02
TMED7	2.18	3.17E-02
TSPAN2	2.18	2.69E-02
CARD8	2.18	4.07E-03
CD47	2.18	3.99E-03
SBF2	2.17	1.82E-02
PPP1R11	2.17	2.31E-02
ACSL5	2.17	1.26E-02
PNPLA8	2.17	2.66E-02
SRF	2.17	1.96E-02
SNTB1	2.17	4.38E-02
AKIRIN2	2.17	1.20E-02
CPPED1	2.17	5.29E-02
GLUL	2.17	5.08E-02
BST1	2.17	1.98E-02
STAP1	2.17	3.45E-02
CTSB	2.17	3.59E-02
USPL1	2.17	7.13E-03
ZNRF2	2.17	7.03E-03
SCYL3	2.17	3.13E-02
METTL4	2.17	2.38E-02
SLFN12	2.17	4.67E-02
GDI2	2.16	1.69E-03
ZDHHC13	2.16	1.66E-02
RESF1	2.16	5.03E-03
KIF5B	2.16	9.89E-03
MAX	2.16	6.78E-03
NCSTN	2.16	4.47E-02
CXorf38	2.16	2.76E-03
GLIPR2	2.16	5.24E-02
ABHD3	2.16	2.93E-02
AMN1	2.16	1.98E-02
ABI1	2.16	2.86E-02
PRKD2	2.16	3.51E-02
GIMAP8	2.15	3.57E-02
THEMIS2	2.15	4.55E-02
RB1	2.15	1.44E-02
DHRSX	2.15	2.12E-02
PSMA3	2.14	5.46E-03
TBC1D2B	2.14	2.88E-02
MFSD14A	2.14	2.60E-02
CNEP1R1	2.14	3.29E-02
ZNF230	2.13	2.63E-02

PIP4P2	2.13	2.66E-02
GSAP	2.13	2.12E-02
MS4A6A	2.13	3.25E-02
KDM6A	2.13	2.58E-02
TTC32	2.13	4.31E-02
SDHD	2.12	1.46E-02
GBA	2.11	3.96E-02
ADPGK	2.11	2.42E-03
PPP2R2A	2.11	1.00E-02
MTMR4	2.11	1.63E-02
SNX2	2.11	2.19E-02
CYLD	2.10	2.10E-02
WASHC4	2.10	1.54E-02
TMEM167B	2.10	1.71E-02
ENTPD1	2.10	2.05E-02
TXNIP	2.10	1.05E-02
ST3GAL5	2.10	1.47E-02
NPTN	2.10	2.80E-02
NUP58	2.10	1.36E-02
CCNL1	2.10	1.13E-02
TRIQK	2.09	3.98E-02
ANXA2R	2.09	3.11E-02
SPCS3	2.09	1.23E-02
PPCDC	2.08	3.93E-02
LAPTM4A	2.08	1.90E-02
DPH3	2.08	6.95E-03
TXNL4B	2.08	7.19E-03
RIOK3	2.08	2.96E-02
SLC37A1	2.08	3.06E-02
KPNB1	2.08	3.85E-02
TRIM27	2.07	3.94E-02
CDC73	2.07	4.30E-03
PPM1K	2.07	9.07E-03
LRRC61	2.07	4.55E-02
ACTR3	2.07	3.86E-02
PSMA5	2.06	1.99E-02
KCMF1	2.06	1.13E-03
RMC1	2.06	1.80E-02
BTG2	2.06	2.25E-02
VTA1	2.06	3.21E-02
GTPBP2	2.06	2.22E-02
SZRD1	2.05	8.45E-03
BNIP3L	2.05	5.11E-02
SPART	2.05	2.65E-02
SETD2	2.05	1.51E-02

SAMD8	2.04	2.86E-02
UBR1	2.04	6.84E-03
ZNF619	2.04	2.11E-02
GALNS	2.04	1.95E-02
SFT2D2	2.04	1.93E-02
PSEN1	2.04	2.19E-02
PRPF40A	2.04	5.16E-04
SHOC2	2.04	2.39E-02
TRMT1L	2.04	1.65E-02
CSTF3	2.03	1.31E-02
ZNF350	2.03	1.93E-02
NFYA	2.03	1.80E-02
CCNDBP1	2.03	9.47E-03
STX12	2.03	1.37E-02
BBX	2.03	1.38E-02
JKAMP	2.02	4.74E-02
MANBAL	2.02	9.80E-03
TMUB2	2.02	7.99E-03
RAB14	2.02	5.03E-03
ANXA5	2.02	3.66E-02
HBP1	2.02	4.82E-02
SNX13	2.02	2.66E-02
MARCHF5	2.02	2.65E-02
NFKBIZ	2.02	3.23E-02
DCAF11	2.02	1.85E-02
TOPORS	2.01	5.03E-02
SLU7	2.01	1.16E-02
ATP6V1D	2.01	1.59E-03
CFLAR	2.01	4.27E-02
GNAI3	2.00	7.56E-03
CALM2	2.00	1.55E-02
YME1L1	2.00	1.20E-03
YIPF6	2.00	5.46E-03
PI4K2A	-2.00	9.13E-03
SARM1	-2.00	3.88E-02
TEDC1	-2.00	4.31E-02
PDCD7	-2.00	3.20E-02
SNRNP70	-2.00	1.01E-03
TRIM47	-2.00	4.57E-02
TAB1	-2.00	1.19E-02
MRI1	-2.00	1.78E-02
PRPS1	-2.00	1.02E-02
HCST	-2.00	3.87E-02
DUS2	-2.01	5.46E-03
IL32	-2.01	2.39E-02

ZNF34	-2.01	5.33E-02
CUTA	-2.01	2.48E-02
RAB33A	-2.01	4.78E-02
YLPM1	-2.01	2.48E-02
CPSF4	-2.01	2.95E-03
PABPN1	-2.01	3.83E-02
GIMAP1	-2.02	9.44E-03
CD3G	-2.02	2.06E-02
C16orf86	-2.02	2.27E-02
FOXO1	-2.02	1.76E-02
ATM	-2.03	5.29E-02
TRIM65	-2.03	1.82E-02
LCK	-2.03	1.38E-02
SCRN2	-2.03	2.65E-02
ZNF219	-2.03	4.93E-02
ZBTB24	-2.03	8.20E-03
ZFYVE19	-2.03	2.41E-03
MPRIP	-2.03	1.68E-02
SIGLEC10	-2.04	3.91E-02
NLRC3	-2.04	3.18E-02
MFHAS1	-2.04	3.52E-02
CLEC2D	-2.04	4.78E-02
EVI5L	-2.04	1.75E-02
SEPTIN11	-2.04	8.58E-03
GIPC1	-2.04	2.00E-02
RHOBTB2	-2.05	6.74E-03
BIN1	-2.05	4.27E-03
PCED1A	-2.05	2.41E-03
QTRT1	-2.05	4.52E-03
CD81	-2.05	1.52E-02
PFAS	-2.05	2.10E-02
HSD17B8	-2.05	2.71E-02
CPSF1	-2.05	5.15E-03
UBASH3A	-2.05	7.51E-03
PDCD11	-2.05	3.44E-02
POP1	-2.05	4.31E-02
ENDOV	-2.05	1.46E-02
MORC2	-2.05	1.85E-02
PGAP2	-2.06	4.26E-03
DNMT1	-2.06	2.30E-03
DBN1	-2.06	4.29E-02
CD99	-2.06	6.55E-03
NME3	-2.06	1.80E-02
TRBC2	-2.06	1.20E-02
SMG6	-2.06	3.11E-02

CAMKK1	-2.06	3.18E-02
DTD1	-2.06	1.35E-03
RBM15	-2.06	7.34E-03
DHRS4L2	-2.06	3.47E-02
STMN3	-2.07	1.80E-02
MDN1	-2.07	3.92E-02
PIGP	-2.07	3.46E-02
HMOX2	-2.07	6.73E-03
WDR54	-2.07	1.96E-02
D2HGDH	-2.07	3.48E-02
PGAP3	-2.07	1.62E-02
MIEF2	-2.07	2.07E-02
ANKZF1	-2.07	4.77E-03
WDR59	-2.07	7.17E-03
KAT14	-2.07	2.16E-02
PARP16	-2.08	2.65E-02
LIG1	-2.08	1.38E-02
CNTROB	-2.08	3.45E-02
RCAN1	-2.08	4.32E-02
NBPF11	-2.08	3.57E-02
SFI1	-2.08	1.49E-03
CMTM8	-2.08	5.45E-02
POMT1	-2.08	2.56E-02
HRAS	-2.08	3.15E-02
FANCE	-2.09	2.46E-02
HNRNPU	-2.09	5.29E-02
KHK	-2.09	5.09E-02
KRBA2	-2.09	5.43E-02
ALDOC	-2.09	3.59E-02
BAG3	-2.09	2.81E-02
TOP1MT	-2.09	3.17E-02
DAG1	-2.09	5.27E-02
MTR	-2.09	1.40E-02
PLCB2	-2.09	7.41E-03
BCL2	-2.09	2.17E-02
CAPS	-2.10	1.71E-02
FCSK	-2.10	5.09E-03
APBA2	-2.10	3.34E-02
ECHDC2	-2.10	2.38E-02
ZNF227	-2.10	3.43E-02
NDUFA13	-2.10	3.97E-02
GPR89A	-2.11	3.88E-02
DYRK2	-2.11	1.61E-02
SAC3D1	-2.11	1.62E-02
DDX46	-2.11	2.72E-02

LTBP4	-2.11	1.26E-02
AKR1B1	-2.11	5.82E-03
ACBD4	-2.11	9.72E-03
TRMT44	-2.11	4.27E-03
PHF19	-2.12	7.56E-03
VIPR1	-2.12	1.57E-02
MAPK13	-2.12	9.79E-03
POLE	-2.12	3.83E-02
ZNF48	-2.12	4.21E-02
MFSD3	-2.13	2.55E-02
CENPV	-2.13	2.85E-02
AFG3L1P	-2.13	2.35E-02
LTBP3	-2.13	2.54E-02
ENDOG	-2.13	3.15E-02
CD96	-2.13	1.32E-02
ANKRD55	-2.13	2.86E-02
KLHDC4	-2.13	1.59E-02
ZFPM1	-2.13	3.69E-02
PIGL	-2.14	4.37E-02
HDGFL2	-2.14	5.58E-03
HPGD	-2.14	5.08E-02
ZNF696	-2.14	6.61E-03
PARD6A	-2.14	2.17E-02
CD1C	-2.14	2.17E-02
ZNF783	-2.14	1.51E-02
ATP1A1	-2.14	3.54E-03
ADK	-2.14	8.33E-03
TRGV2	-2.14	4.84E-02
TRBC1	-2.14	8.17E-03
GOLGA8A	-2.14	4.98E-02
SNHG11	-2.15	5.76E-03
ZNF784	-2.15	4.36E-02
DDX27	-2.15	1.21E-03
CBX5	-2.15	1.20E-02
ZNF589	-2.15	1.47E-02
RBM28	-2.15	1.30E-02
GPATCH4	-2.15	1.71E-02
SLC35D1	-2.15	2.62E-02
R3HDM1	-2.15	3.65E-03
TRERF1	-2.16	6.37E-03
ZNF512B	-2.16	2.11E-02
USP28	-2.16	1.51E-02
ZNF444	-2.16	7.03E-03
PYCR3	-2.16	1.43E-02
FLNA	-2.16	1.55E-02

RBFA	-2.16	1.04E-02
ZFP14	-2.16	4.13E-02
PARP1	-2.16	6.70E-03
MIB2	-2.16	2.42E-03
ATRN	-2.17	4.12E-02
FLYWCH1	-2.17	4.89E-03
GZF1	-2.17	3.20E-02
KLF12	-2.17	1.13E-02
IL21R	-2.17	5.57E-03
SAMD3	-2.17	4.83E-02
WDR91	-2.17	3.80E-03
TMEM107	-2.17	5.46E-03
RPAP1	-2.18	9.07E-03
KANTR	-2.18	3.14E-02
AMPD3	-2.18	1.27E-02
DIP2A	-2.18	1.51E-02
ATG9B	-2.18	3.76E-02
ABHD15	-2.18	9.08E-03
HLCS	-2.18	3.48E-02
EME2	-2.19	9.34E-03
ZNF2	-2.19	2.60E-02
KLC2	-2.19	1.80E-02
TAF4	-2.19	5.21E-02
AASDH	-2.19	6.58E-03
DOHH	-2.19	2.52E-02
LBH	-2.19	3.70E-03
RTN4R	-2.20	4.94E-02
CCDC61	-2.20	5.46E-03
FAHD2A	-2.20	4.07E-03
HTT	-2.20	1.13E-02
ZBTB38	-2.20	2.56E-03
TRAPPC6A	-2.20	8.33E-03
TRIM52	-2.20	6.95E-03
DHODH	-2.20	6.53E-03
ZAP70	-2.20	7.36E-03
PLCD1	-2.20	2.03E-02
LGALS12	-2.20	3.80E-02
CLUH	-2.20	1.27E-02
THBD	-2.20	4.83E-02
NIBAN3	-2.20	2.96E-02
SFXN5	-2.20	6.50E-03
NOXA1	-2.21	3.34E-02
CHD6	-2.21	1.99E-02
NUMA1	-2.21	5.98E-03
CBLB	-2.21	1.10E-02

PEX5	-2.21	2.07E-02
C12orf57	-2.21	4.24E-02
CCDC28B	-2.21	2.33E-02
TCFL5	-2.22	3.01E-02
FBXW9	-2.22	3.11E-02
TBC1D19	-2.22	5.28E-02
NOL9	-2.22	1.35E-02
SIGIRR	-2.22	3.41E-03
KAT6B	-2.22	1.95E-02
DOP1B	-2.22	1.11E-02
PILRB	-2.22	1.95E-02
NUDC	-2.22	7.16E-03
SPN	-2.22	5.40E-03
PLEKHF1	-2.23	2.69E-02
SLC2A4RG	-2.23	2.74E-02
SNRNP25	-2.23	1.22E-02
LRRC45	-2.23	1.09E-02
CD6	-2.23	4.27E-03
IL7R	-2.24	2.48E-02
ANO9	-2.24	1.19E-02
PRH1	-2.24	4.96E-02
RAD18	-2.24	2.29E-02
HS6ST1	-2.24	4.90E-03
USF2	-2.24	5.17E-02
FBLN7	-2.24	1.76E-02
PBX4	-2.24	2.13E-02
SLC2A8	-2.25	1.59E-02
ABCA3	-2.25	3.57E-02
TCHP	-2.25	1.56E-02
CREB3L4	-2.25	3.59E-02
SEPTIN9	-2.25	2.73E-03
PIK3IP1	-2.25	2.20E-02
SLC25A29	-2.25	2.96E-02
CCDC57	-2.25	5.87E-03
PELI3	-2.26	8.88E-03
BACH2	-2.26	6.04E-03
ZBTB39	-2.26	1.68E-02
ESYT1	-2.26	7.11E-03
SLC25A4	-2.26	1.87E-02
SELENOM	-2.26	2.63E-02
USP45	-2.26	4.04E-02
CTSK	-2.26	4.58E-02
FCMR	-2.26	6.88E-03
PDE6G	-2.26	2.45E-02
EIF2B4	-2.26	9.49E-04

CEP290	-2.26	5.46E-02
PDE4DIP	-2.26	1.19E-02
MAZ	-2.27	1.64E-02
ZNF827	-2.27	4.10E-02
SUPT3H	-2.27	3.89E-02
LARGE2	-2.27	2.88E-02
SH3BP1	-2.27	4.24E-02
RPTOR	-2.27	2.78E-03
SH2D2A	-2.27	1.14E-02
ID2	-2.28	7.46E-03
COQ8A	-2.28	7.27E-03
ZDHHC8	-2.28	2.95E-03
ZNF677	-2.28	4.01E-02
DSTYK	-2.28	5.01E-04
RASA4CP	-2.28	2.82E-02
CD247	-2.28	7.41E-03
HNRNPH1	-2.28	3.58E-03
XYLT1	-2.29	1.75E-02
SWAP70	-2.29	3.14E-03
CAMKMT	-2.29	2.26E-03
ANKRD36C	-2.29	1.43E-02
BCL9	-2.29	4.25E-02
TBC1D9	-2.29	1.70E-02
NBPF15	-2.29	4.17E-02
SLC4A7	-2.29	2.12E-02
ERCC2	-2.29	2.01E-02
CABIN1	-2.30	4.30E-03
TSNARE1	-2.30	6.42E-03
MAPK8IP3	-2.30	8.94E-04
TNIK	-2.30	6.61E-03
NT5E	-2.30	4.75E-02
PRKCSH	-2.31	2.66E-02
TCF7	-2.32	5.49E-03
ZNF362	-2.32	1.30E-03
VEGFB	-2.32	1.17E-02
RAB44	-2.32	4.96E-02
GCN1	-2.32	5.44E-04
TSPYL4	-2.32	3.02E-02
MIS18A	-2.32	1.35E-02
TLE2	-2.32	1.74E-02
FDXR	-2.32	2.40E-02
SKAP1	-2.33	8.33E-04
C9orf139	-2.33	1.34E-02
IL11RA	-2.33	1.05E-02
SNRNP200	-2.33	1.09E-04

REX1BD	-2.33	4.77E-03
NMRAL1	-2.33	1.34E-03
SMARCA4	-2.33	1.20E-03
CFAP410	-2.33	5.73E-03
HGH1	-2.33	3.79E-03
BRF1	-2.33	2.51E-03
CDIP1	-2.34	3.06E-03
CTF1	-2.34	5.37E-02
IFT74	-2.34	4.29E-02
MISP3	-2.34	2.90E-02
BRI3BP	-2.35	3.51E-03
ZNF767P	-2.35	1.54E-03
PLCH2	-2.35	4.94E-02
ZBTB40	-2.35	2.51E-03
S1PR4	-2.35	5.03E-03
CARD11	-2.35	2.01E-03
ALKBH6	-2.35	3.11E-02
AUTS2	-2.35	3.67E-02
SPON2	-2.35	5.25E-02
TRGV4	-2.36	4.74E-02
ZNF428	-2.36	4.80E-02
SCAND2P	-2.36	3.86E-02
EHMT2	-2.36	1.80E-02
ZNF74	-2.36	1.17E-02
ABHD14B	-2.36	5.19E-04
GTPBP3	-2.37	8.71E-03
PPP2R5D	-2.37	1.02E-02
RORA	-2.37	9.72E-03
ZNF440	-2.38	2.58E-02
HNRNPD	-2.38	3.78E-03
SPTBN1	-2.38	8.59E-03
KLHL22	-2.38	1.55E-03
PPT2	-2.38	1.11E-02
CEP78	-2.38	1.76E-02
PLCB3	-2.38	2.05E-02
NEURL1	-2.39	2.92E-02
KCNE1	-2.39	4.37E-02
LSM14B	-2.39	2.74E-02
MAP3K12	-2.39	1.42E-02
TBX21	-2.39	3.72E-02
PLXDC1	-2.39	3.27E-02
IRS2	-2.39	3.91E-02
TTC3	-2.39	3.75E-03
SLC41A1	-2.39	8.27E-03
KLC4	-2.39	3.72E-03

PGPEP1	-2.39	2.62E-02
ERN1	-2.40	2.47E-03
RUBCNL	-2.40	3.16E-02
FNDC10	-2.40	1.45E-02
DHRS13	-2.40	2.77E-02
NUDT13	-2.40	5.10E-02
CEP192	-2.40	1.12E-02
TLE1	-2.40	1.11E-02
ZNHIT6	-2.40	1.85E-02
EIF2AK3	-2.41	2.62E-02
CEP128	-2.41	3.11E-02
MYO9A	-2.41	1.41E-02
CCDC134	-2.41	2.83E-02
PI4KAP2	-2.41	2.81E-02
CNR2	-2.41	7.82E-03
C1orf35	-2.41	6.70E-03
STK39	-2.42	2.11E-03
UBN2	-2.42	1.49E-02
RTL6	-2.42	3.51E-03
LFNG	-2.42	6.64E-03
PTGDR	-2.42	3.43E-02
AFAP1L2	-2.42	4.37E-02
ETS1	-2.43	1.63E-02
BCL7C	-2.43	5.46E-03
ZNF664	-2.43	4.84E-02
CD27	-2.43	5.03E-03
FMO4	-2.43	4.64E-02
SMAD7	-2.43	4.26E-03
DCLRE1B	-2.44	1.11E-02
HOMER3	-2.44	1.31E-02
SPIN4	-2.44	3.09E-02
DDX42	-2.44	1.02E-02
KLHL3	-2.44	3.11E-02
PLEC	-2.45	6.73E-03
FUT8	-2.45	1.51E-02
NKG7	-2.45	2.85E-02
ARHGEF9	-2.45	5.20E-03
PHACTR1	-2.45	6.70E-03
DPEP2	-2.45	4.54E-03
ACAP3	-2.45	1.78E-03
PPP1R3E	-2.45	1.05E-02
ZBED3	-2.45	1.64E-02
VPS13A	-2.46	1.51E-02
GPM6B	-2.46	4.27E-02
CD93	-2.46	1.50E-02

PLLP	-2.46	3.62E-02
APBB1	-2.46	1.69E-02
AK5	-2.46	1.35E-02
WRN	-2.46	2.82E-02
CX3CR1	-2.46	1.66E-02
SLC27A1	-2.47	1.04E-02
SYTL2	-2.47	1.90E-02
ZNF517	-2.47	3.11E-02
PABPC4	-2.47	6.73E-03
AKAP1	-2.47	2.86E-03
ADCY9	-2.47	1.90E-02
UPF3A	-2.47	8.52E-03
ZNF282	-2.48	1.36E-02
WDR6	-2.48	1.27E-02
SERTAD2	-2.48	4.28E-03
FANCI	-2.48	5.36E-02
DDX54	-2.48	1.25E-03
MTA1	-2.48	2.24E-03
DTX4	-2.49	9.86E-03
ARL4C	-2.49	8.31E-03
TCF19	-2.49	1.93E-02
HRH2	-2.49	8.21E-03
GOLGA2P5	-2.49	3.59E-02
PLCXD1	-2.49	1.42E-02
MACROD1	-2.49	4.06E-02
CRIP2	-2.49	2.56E-02
GOLGA7B	-2.49	2.50E-02
XRCC3	-2.49	2.15E-03
AFF3	-2.49	6.70E-03
NLRX1	-2.49	1.70E-02
PRRT3	-2.49	9.96E-03
TBC1D10A	-2.50	4.40E-05
TNFRSF8	-2.50	3.11E-03
PTGDR2	-2.50	4.37E-02
CHPF	-2.50	2.14E-02
TMEM204	-2.50	1.23E-02
SPATC1L	-2.51	1.36E-02
PIK3R1	-2.51	9.91E-04
LRRC23	-2.51	3.16E-02
NCALD	-2.51	1.81E-02
MVK	-2.52	7.07E-03
ST6GALNAC6	-2.52	4.53E-02
BICDL1	-2.52	7.05E-03
SPECC1L	-2.53	1.54E-02
AHI1	-2.53	4.42E-02

TSPOAP1	-2.53	4.05E-02
CENPB	-2.53	3.21E-02
LPCAT4	-2.53	8.16E-04
PARP15	-2.53	1.02E-03
ISYNA1	-2.53	5.24E-03
SPINT1	-2.53	1.37E-02
RABL6	-2.53	8.68E-03
MDS2	-2.54	1.27E-02
TMEM25	-2.54	1.36E-02
MFGE8	-2.54	2.43E-02
CTSW	-2.54	2.25E-02
MYBBP1A	-2.54	5.60E-03
PA2G4P4	-2.54	3.62E-02
ANTXRLP1	-2.54	1.96E-02
SPNS2	-2.55	3.92E-02
CUL7	-2.55	1.85E-02
TTC16	-2.55	2.06E-02
B4GAT1	-2.55	9.97E-03
MBD3	-2.55	8.86E-03
GOLIM4	-2.55	3.64E-02
PUS1	-2.55	2.41E-03
CD160	-2.56	4.54E-02
CHD3	-2.56	8.45E-03
NELL2	-2.56	2.50E-02
TRDJ1	-2.56	5.24E-02
PITPNC1	-2.56	9.33E-04
CROCCP2	-2.56	9.22E-03
MHENCR	-2.56	2.81E-03
PLCG1	-2.56	9.13E-03
STXBP5	-2.56	5.65E-03
NT5C3AP2	-2.56	4.55E-02
SETD1A	-2.56	2.70E-02
MAP3K9	-2.57	4.74E-02
UBQLN4	-2.57	8.33E-04
ZNF563	-2.57	3.54E-02
NMT2	-2.58	4.79E-03
DUSP16	-2.58	3.41E-03
C9orf40	-2.58	1.61E-03
AGO1	-2.58	8.62E-04
CXCR3	-2.59	1.09E-02
MTSS1	-2.59	6.14E-04
SIK2	-2.59	2.87E-02
EPHA1	-2.59	8.86E-03
CLCF1	-2.59	2.30E-02
MYO15B	-2.59	1.98E-03

KLRD1	-2.60	2.49E-02
MARCHF9	-2.60	6.35E-03
FAHD2CP	-2.60	3.46E-02
KIF19	-2.60	4.92E-02
ZNF891	-2.60	3.42E-02
FSD1	-2.60	4.55E-02
DNMBP	-2.60	2.56E-02
CHRNG	-2.61	7.05E-03
RTL10	-2.61	2.15E-04
DGKD	-2.61	8.53E-05
TEF	-2.61	6.77E-03
DHX35	-2.61	2.04E-02
CD320	-2.62	3.14E-03
NFATC2	-2.62	2.56E-03
PPARA	-2.62	3.54E-03
ZNF628	-2.62	5.29E-02
TMEM177	-2.62	1.67E-03
CD7	-2.62	4.29E-03
SYNGR1	-2.63	5.42E-03
SH2B1	-2.63	3.94E-03
NSD2	-2.63	4.50E-03
HID1	-2.63	4.22E-02
RASL11A	-2.63	5.34E-02
ARL10	-2.63	1.85E-02
TMEM238	-2.64	2.30E-02
SYNE1	-2.64	2.06E-03
ACTL10	-2.64	9.26E-03
EML3	-2.64	1.75E-04
MEGF6	-2.64	4.95E-03
FBRSL1	-2.64	6.37E-03
DEF6	-2.65	6.44E-04
KISS1R	-2.65	5.15E-02
HAVCR1	-2.65	4.13E-02
POLR1E	-2.65	2.52E-03
ZBED2	-2.66	3.03E-02
ZSWIM5	-2.66	2.86E-02
LYPD3	-2.66	3.42E-02
NR2F6	-2.66	9.31E-03
PPP1R13B	-2.67	8.00E-03
CICP14	-2.67	3.64E-02
SEMA6A	-2.67	2.85E-02
CLIP2	-2.67	6.32E-03
NECTIN1	-2.68	3.13E-02
TGFBR3	-2.68	9.97E-03
QSOX2	-2.68	4.50E-03

TSTD3	-2.68	3.42E-02
NCOR2	-2.68	1.99E-03
RAB19	-2.68	3.94E-02
HIP1R	-2.68	2.91E-03
TMEM120B	-2.69	3.51E-03
ATP2B4	-2.69	7.66E-04
CRTC1	-2.69	2.77E-03
NOP53	-2.69	2.51E-03
CCDC102A	-2.69	4.79E-03
CACNA2D2	-2.69	2.43E-02
AGPAT4	-2.69	2.73E-03
TJP3	-2.69	1.31E-02
VMO1	-2.69	5.50E-02
ZC3H12B	-2.69	3.35E-02
IKZF2	-2.69	1.43E-02
ENO3	-2.69	2.50E-02
TSPYL2	-2.70	1.11E-03
ZNF683	-2.70	4.29E-02
CHCHD6	-2.70	2.29E-03
LRRC56	-2.70	3.44E-02
TMIGD2	-2.71	5.13E-03
GPR55	-2.71	4.38E-02
CACNB3	-2.71	4.88E-02
DOK7	-2.71	3.08E-02
PRSS23	-2.71	3.57E-02
MORC4	-2.72	1.58E-02
TLCD3A	-2.72	5.20E-02
BCKDHB	-2.72	7.06E-03
LUNAR1	-2.72	5.12E-02
DLGAP3	-2.72	5.24E-02
ALKBH2	-2.72	2.01E-03
TPM2	-2.72	3.98E-02
ASRGL1	-2.72	7.83E-04
POLR1G	-2.73	4.23E-02
TSEN54	-2.73	1.01E-03
TNFRSF18	-2.73	1.24E-02
BDH1	-2.73	1.94E-03
KLRG1	-2.73	4.73E-03
NCR1	-2.73	2.60E-02
DBNDD1	-2.74	1.69E-02
NRBP2	-2.74	1.48E-02
NSG1	-2.74	1.85E-02
TRGV10	-2.74	2.02E-02
LRRC75A	-2.74	1.39E-02
EPN2	-2.74	7.27E-03

IL24	-2.75	2.94E-02
ANKRD34A	-2.75	4.38E-02
FBXO2	-2.75	8.29E-03
YPEL1	-2.75	7.23E-03
VSIG1	-2.75	9.13E-03
CDC25B	-2.75	4.33E-04
PLCL1	-2.75	5.04E-03
NCR3	-2.75	2.91E-03
ADAM19	-2.75	2.79E-03
ITGB4	-2.76	5.49E-02
CDHR2	-2.76	2.59E-02
UBE2Q2P1	-2.76	4.37E-02
LDOC1	-2.76	5.60E-03
RAD54L2	-2.76	5.43E-04
RFLNB	-2.76	1.79E-02
AMDHD2	-2.76	8.33E-04
BHLHE40	-2.76	5.03E-03
KCTD7	-2.76	8.33E-04
CARNS1	-2.77	1.30E-02
ZNF286A	-2.77	2.57E-02
C1orf216	-2.77	1.00E-02
RRBP1	-2.78	6.14E-03
EPHX1	-2.78	6.62E-03
DLK2	-2.78	4.73E-02
ZFP69	-2.78	1.36E-02
AXIN1	-2.78	2.11E-04
CAMSAP2	-2.78	5.15E-02
ARL4D	-2.79	1.03E-02
KCNQ5	-2.79	3.85E-02
EMILIN1	-2.79	1.72E-02
ACACB	-2.79	1.75E-02
TSHZ1	-2.80	1.58E-03
UICLM	-2.80	4.44E-02
FABP3	-2.80	4.12E-02
ARHGEF39	-2.80	3.16E-02
ARHGAP10	-2.81	3.00E-03
IL23R	-2.81	5.30E-02
SLC45A1	-2.81	5.40E-02
TRAM2	-2.81	5.08E-04
CA5B	-2.81	3.29E-03
INCENP	-2.81	3.25E-02
ZNF568	-2.81	1.63E-02
TCEAL3	-2.81	1.75E-02
SLC39A14	-2.81	2.64E-02
ALKBH8	-2.82	1.48E-02

GZMM	-2.82	3.77E-03
DGCR8	-2.82	2.95E-03
HHAT	-2.82	3.57E-02
ZNF37BP	-2.82	5.62E-03
CKB	-2.83	1.63E-02
TRAJ54	-2.83	4.30E-02
BCL7A	-2.84	3.25E-03
CXXC5	-2.84	1.29E-03
DHRS11	-2.84	1.27E-02
FFAR4	-2.85	5.00E-02
KCTD15	-2.85	1.63E-02
PLXNA3	-2.85	5.04E-03
ZFP30	-2.85	3.17E-02
DMPK	-2.85	2.16E-02
SAMD10	-2.85	3.63E-03
PLCB1	-2.85	3.43E-03
SLC29A1	-2.85	1.37E-02
RRP1	-2.85	2.11E-04
PORCN	-2.86	1.66E-03
CFL1P1	-2.86	2.86E-02
ADAMTS1	-2.86	4.61E-02
LAT	-2.86	2.47E-02
ZFP92	-2.86	4.82E-02
DPYSL4	-2.87	4.77E-02
LAX1	-2.87	4.31E-03
AZIN2	-2.87	1.81E-02
TRIP10	-2.88	7.08E-03
AGFG2	-2.88	3.35E-04
TKTL1	-2.88	1.02E-02
THRA	-2.88	3.95E-03
PCNT	-2.89	1.59E-03
NLRP7	-2.89	3.83E-02
NHLRC4	-2.89	1.25E-02
IQCK	-2.89	4.49E-02
MSC	-2.89	1.20E-02
HEATR9	-2.90	4.56E-02
ZNF70	-2.90	2.26E-02
BMP8A	-2.90	3.77E-02
DPEP3	-2.91	9.97E-03
LLGL2	-2.91	1.28E-03
NECTIN3	-2.91	4.25E-02
NBPF1	-2.92	4.79E-03
LAS1L	-2.92	2.42E-03
CCNB1IP1	-2.93	5.58E-03
LKAAEAR1	-2.94	4.31E-02

PELP1	-2.94	1.30E-03
ITGA10	-2.94	4.19E-02
GAN	-2.94	2.47E-02
DDX11	-2.94	1.81E-02
CHST14	-2.94	4.16E-03
PPP2R3B	-2.94	3.34E-04
FRMD5	-2.95	3.90E-02
CELSR2	-2.95	4.05E-02
PRAG1	-2.95	1.02E-03
OXER1	-2.95	5.58E-03
ACER1	-2.95	4.03E-02
CRIP3	-2.95	3.51E-02
CD248	-2.95	1.56E-02
TNK1	-2.95	2.47E-03
RADX	-2.96	2.31E-02
SCAI	-2.96	8.10E-03
CCDC65	-2.96	2.15E-02
IFFO2	-2.96	2.95E-03
C1QTNF3	-2.96	4.08E-02
ADAMTS5	-2.96	3.78E-02
EPHB3	-2.97	3.92E-02
CST7	-2.97	2.13E-03
ADGRG1	-2.97	1.54E-02
JMY	-2.97	1.35E-04
TULP4	-2.97	4.11E-04
BMP8B	-2.97	3.57E-02
PCBP4	-2.97	2.52E-03
FBF1	-2.97	6.23E-03
PLB1	-2.98	2.78E-03
DDX12P	-2.98	3.81E-02
ZNF615	-2.98	1.85E-02
JUP	-2.98	8.36E-03
CCDC106	-2.99	4.06E-03
PDP2	-2.99	1.00E-02
LIM2	-2.99	3.76E-02
WDR86	-2.99	1.52E-02
ZDHHC1	-3.00	1.42E-02
HIRIP3	-3.00	2.25E-03
DBP	-3.00	1.02E-03
HAGHL	-3.00	5.24E-03
MC1R	-3.01	1.54E-02
TEAD2	-3.01	3.30E-02
PID1	-3.01	2.37E-03
DEGS2	-3.01	1.71E-02
CAVIN3	-3.01	3.42E-02

PFN1P2	-3.02	3.78E-02
SCUBE3	-3.02	2.21E-02
CCL5	-3.02	2.17E-03
ZCCHC18	-3.02	2.48E-02
CHTF18	-3.04	1.21E-03
A2M	-3.04	1.85E-02
CRIP1	-3.05	2.07E-03
ESRP2	-3.05	2.70E-02
KCNH3	-3.05	3.15E-03
NMUR1	-3.05	1.51E-02
RASSF1	-3.05	1.49E-03
ZBTB16	-3.05	4.29E-03
ZNF831	-3.06	5.55E-04
OLIG2	-3.06	3.01E-02
DYNC2I1	-3.06	6.69E-03
FALEC	-3.06	3.53E-02
GRAP	-3.06	6.14E-03
IL23A	-3.06	6.60E-03
TOX2	-3.06	1.17E-02
PCYT1B	-3.07	3.12E-02
MLC1	-3.07	7.08E-03
CDAN1	-3.08	8.33E-04
RIMBP3	-3.08	3.38E-02
IGIP	-3.08	2.73E-02
KCNK12	-3.08	2.21E-02
AKT3	-3.08	5.04E-03
KIAA1671	-3.08	3.04E-02
SBK1	-3.08	1.49E-03
NXPH4	-3.09	3.11E-02
GNB1L	-3.09	2.09E-04
L3MBTL4	-3.10	2.88E-02
FGFBP2	-3.10	1.37E-02
OTUD7B	-3.10	1.97E-02
ATOH8	-3.10	3.17E-02
A2MP1	-3.11	3.06E-02
PAK6	-3.11	2.91E-02
CERS4	-3.11	7.66E-04
PLD4	-3.11	3.16E-03
BNC2	-3.11	3.08E-02
SERPINF1	-3.11	2.77E-03
SOX12	-3.12	3.51E-03
ZNF395	-3.12	2.77E-03
ADORA3	-3.12	1.28E-02
ZNF569	-3.12	5.46E-03
CEP250	-3.13	1.59E-03

AMIGO1	-3.13	3.95E-03
AGAP9	-3.13	3.14E-02
MYRF	-3.14	2.96E-02
TRAV23DV6	-3.14	2.28E-03
ZBTB4	-3.15	2.48E-05
CA14	-3.15	2.96E-02
EOMES	-3.15	1.72E-03
GPR19	-3.16	2.11E-02
AHNAK	-3.16	1.09E-03
ADGRE4P	-3.16	2.38E-02
NDRG2	-3.16	8.85E-04
FGF17	-3.16	2.92E-02
ALKAL2	-3.19	2.86E-02
OVGP1	-3.19	2.60E-02
KLRB1	-3.20	1.02E-02
PIF1	-3.20	2.60E-02
NINL	-3.20	2.66E-02
AEBP1	-3.20	2.04E-02
BOK	-3.21	1.96E-02
CACNA1I	-3.21	8.54E-03
NBPF3	-3.22	6.89E-03
PYROXD2	-3.22	1.72E-03
RAMP1	-3.22	9.02E-03
ZFP41	-3.23	3.48E-04
TMEM8B	-3.23	2.42E-03
C17orf50	-3.23	2.59E-02
ZNF316	-3.23	8.70E-03
SMPD3	-3.23	2.98E-03
SNAPC4	-3.24	8.33E-04
CHD1L	-3.24	2.32E-02
FBXW4P1	-3.24	8.36E-03
FOCAD	-3.25	6.09E-05
CD163	-3.26	4.53E-03
ABCA17P	-3.26	1.96E-02
SLC16A11	-3.26	1.37E-02
SGSM1	-3.27	2.20E-02
HES1	-3.27	1.14E-02
SLC4A4	-3.27	1.50E-02
CNFN	-3.27	1.35E-02
MAMSTR	-3.28	1.21E-02
ZP3	-3.28	7.56E-03
REEP6	-3.28	4.66E-03
TAGLN	-3.28	3.57E-03
KRT8P34	-3.29	1.68E-02
ZHX3	-3.29	2.73E-03

GOLGA8B	-3.30	1.67E-03
MIAT	-3.30	4.27E-03
HYAL1	-3.31	8.06E-03
GTF3C1	-3.32	1.63E-05
PMP22	-3.32	1.81E-02
WNT5B	-3.33	1.17E-02
ALOX15	-3.33	1.73E-02
CPAMD8	-3.34	3.81E-03
S1PR5	-3.34	3.99E-03
SLC25A15	-3.34	2.95E-03
SIGLEC17P	-3.34	5.03E-03
SALL2	-3.34	2.12E-02
NOG	-3.35	9.66E-03
C13orf46	-3.35	8.71E-03
ENGASE	-3.35	7.17E-05
PRSS57	-3.37	1.17E-02
UNC119B	-3.38	2.36E-04
CROCC	-3.38	6.74E-03
SORBS3	-3.38	1.49E-04
ALS2CL	-3.38	1.57E-03
ANO8	-3.38	1.79E-02
PPP1R37	-3.39	8.21E-03
BAIAP2	-3.39	8.06E-04
PIK3C2B	-3.41	3.41E-03
CASC19	-3.41	1.78E-02
SLC9A5	-3.41	1.51E-02
TIPIN	-3.41	1.09E-02
GLB1L2	-3.41	1.76E-02
FBL	-3.41	7.14E-04
GIPR	-3.42	1.87E-02
NTHL1	-3.42	4.56E-04
AMOTL1	-3.43	1.80E-02
ASB2	-3.43	2.91E-03
CABP4	-3.43	1.69E-02
BAIAP3	-3.44	3.55E-03
TRAJ49	-3.44	1.60E-02
SLFN12L	-3.44	6.20E-03
ASPH	-3.46	2.67E-04
LYNX1	-3.46	1.76E-02
SPEF2	-3.46	6.91E-03
TFAP4	-3.46	7.36E-03
LRFN3	-3.47	3.66E-03
PALLD	-3.48	2.55E-03
MRPL45P2	-3.49	5.62E-03
PARTICL	-3.49	1.51E-02

DUSP8	-3.49	7.56E-03
SPON1	-3.50	1.39E-02
PKD1P6	-3.50	9.99E-03
ABLIM2	-3.51	1.37E-02
BMP1	-3.51	9.35E-03
SFRP5	-3.51	1.42E-02
SLC25A10	-3.52	6.14E-03
SLC29A2	-3.52	2.75E-03
EBF4	-3.52	1.45E-02
LGR6	-3.53	6.67E-03
TDRD9	-3.54	1.51E-02
ACYP1	-3.55	8.43E-03
C11orf95	-3.56	1.51E-02
MKS1	-3.57	9.17E-05
CXCR6	-3.57	2.95E-03
PIGZ	-3.58	1.21E-02
ENG	-3.58	1.27E-04
CLIC3	-3.58	4.26E-03
PHLDB3	-3.58	4.07E-03
C15orf62	-3.59	1.46E-02
MATK	-3.59	1.02E-03
SPRY1	-3.60	4.28E-03
GPRASP1	-3.61	8.69E-03
GPA33	-3.62	9.96E-04
GNLY	-3.64	2.66E-03
C9orf106	-3.64	1.66E-03
C16orf74	-3.64	2.66E-03
DOC2GP	-3.65	1.00E-02
SDK2	-3.65	8.29E-03
TPPP3	-3.66	2.30E-03
GSDME	-3.66	8.29E-03
EEF2K	-3.66	5.78E-04
SKI	-3.67	3.70E-03
SERPINF2	-3.67	8.27E-03
CALY	-3.67	1.25E-02
NPHP4	-3.68	2.90E-03
PLXND1	-3.68	4.69E-04
AMOT	-3.69	1.20E-02
ETNK2	-3.70	1.06E-02
GSTM2	-3.70	1.41E-03
PWWP3A	-3.70	6.60E-07
TRAJ41	-3.71	9.99E-03
TAMALIN	-3.72	1.16E-03
AXIN2	-3.73	4.70E-03
HABP4	-3.73	2.36E-04

EVA1C	-3.74	4.00E-04
FOXO3B	-3.74	1.14E-03
SCRT2	-3.74	1.08E-02
PZP	-3.74	7.79E-03
TRAF3IP1	-3.75	1.01E-03
IQCC	-3.75	9.55E-03
COL6A2	-3.76	1.29E-03
RDH10	-3.76	7.13E-03
LZTS3	-3.77	1.03E-02
PAIP2B	-3.77	6.70E-03
SDC2	-3.77	7.96E-03
KANK3	-3.80	2.93E-03
SPTAN1	-3.80	1.14E-04
KLHL32	-3.80	9.69E-03
MPP2	-3.81	8.76E-03
SPOCK2	-3.83	1.26E-05
ABCA2	-3.83	1.09E-04
ITPRIPL1	-3.83	1.06E-03
L2HGDH	-3.84	4.32E-03
WNT10B	-3.87	8.95E-03
PRSS33	-3.87	6.73E-03
PODXL2	-3.88	3.92E-03
SCNN1D	-3.92	7.36E-03
ZNF233	-3.92	7.36E-03
CERCAM	-3.92	4.89E-03
KRI1	-3.92	1.14E-03
LYPD2	-3.96	6.20E-03
GALNT12	-3.97	8.33E-04
CCDC136	-3.98	7.36E-03
FGFBP3	-4.00	2.75E-03
FEZ1	-4.01	3.70E-03
FANCB	-4.02	7.09E-03
ZSCAN12	-4.07	3.66E-03
CAND2	-4.08	5.58E-03
CCR12P	-4.08	3.91E-03
AKAP5	-4.11	6.08E-03
TUBB4A	-4.12	2.61E-03
NRG1	-4.13	4.22E-03
ZNF470	-4.13	4.66E-03
ZNF575	-4.14	3.55E-04
SPIRE1	-4.14	4.49E-03
KBTBD11	-4.15	1.09E-04
SCD5	-4.17	5.03E-03
RPH3A	-4.19	2.96E-03
DCHS1	-4.20	1.59E-03

ADAMTS7P1	-4.21	2.90E-03
SLC26A11	-4.23	5.88E-05
DLG5	-4.25	4.73E-03
ICA1L	-4.26	2.73E-03
CROCC2	-4.26	4.77E-03
ABCC5	-4.27	1.68E-04
PDE4DIPP2	-4.29	1.01E-03
SOWAHC	-4.29	4.64E-03
IGFBP6	-4.29	3.55E-03
IL5RA	-4.29	1.01E-03
WDR27	-4.30	8.58E-04
CSNK1E	-4.31	1.51E-04
PHLDB2	-4.35	2.30E-03
OLFM2	-4.44	1.18E-04
LTC4S	-4.44	1.06E-03
COL19A1	-4.45	6.44E-04
ZNF571	-4.45	2.42E-03
ALMS1	-4.46	3.82E-04
IER5L	-4.50	8.93E-04
HERC2	-4.53	2.65E-04
CERNA1	-4.54	2.73E-03
CA6	-4.54	2.30E-03
RAB3A	-4.55	1.99E-03
TENM1	-4.57	7.90E-04
C12orf60	-4.57	2.56E-03
THBS4	-4.63	2.91E-03
SAMD1	-4.65	1.06E-03
NMB	-4.71	3.35E-04
CEP126	-4.73	1.88E-03
ZNF8	-4.77	4.33E-04
ZGRF1	-4.84	1.81E-03
ADGRG5	-4.85	5.85E-05
MMP11	-4.86	3.55E-04
CCDC163	-4.86	1.36E-03
ENHO	-4.89	9.34E-04
DOK4	-4.91	5.01E-04
ZNF771	-4.91	1.04E-03
MXRA8	-4.91	1.99E-03
VENTX	-5.12	8.33E-04
SH3RF3	-5.22	5.74E-04
SIGLEC8	-5.25	9.35E-04
FXYD7	-5.28	9.34E-04
EXTL2	-5.28	9.38E-04
S100B	-5.41	9.38E-04
OLIG1	-5.45	2.11E-04

PLCD3	-5.55	8.33E-04
GPSM1	-5.59	5.03E-04
CHIC1	-5.81	6.22E-04
CTSF	-5.83	4.23E-05
PTCH1	-5.91	4.73E-05
PEG13	-5.97	4.85E-04
PDZD4	-6.07	8.53E-05
WNT7A	-6.32	1.18E-04
JAKMIP2	-6.37	1.09E-04
LMLN	-6.48	2.65E-04
SPNS3	-6.55	1.26E-05
SEMA4C	-7.06	6.60E-07
